# Supplementary material for: GOLPH3 and GOLPH3L maintain Golgi localization of LYSET and a functional mannose 6-phosphate transport pathway
Source: EMBO J. 2024 Nov 25;43(24):6264–90. doi: 10.1038/s44318-024-00305-z (PMC11649813; doi:10.1038/s44318-024-00305-z)
Supplement: Supplementary file 1 — Appendix [file 44318_2024_305_MOESM1_ESM.pdf]

# GOLPH3 and GOLPH3L maintain Golgi localization of LYSET and a functional mannose 6-phosphate transport pathway

Berit K. Brauer[1,‡], Zilei Chen[2,‡], Felix Beirow[1], Jiaran Li[3], Daniel Meisinger[1], Emanuela Capriotti[4], Michaela Schweizer[5], Lea Wagner[1], Jascha Wienberg[1], Laura Hobohm[1], Lukas Heintz[1,8], Wenjie Qiao[6], Yoshiki Narimatsu[7], Jan E. Carette[6], Henrik Clausen[7], Dominic Winter[3], Thomas Braulke[4], Sabrina Jabs[2,*] and Matthias Voss[1,*]

[1] Institute of Biochemistry, Kiel University, Kiel, Germany

[2] Institute of Clinical Molecular Biology, Kiel University, Kiel, Germany & University Medical Center Schleswig-Holstein, Campus Kiel, Kiel, Germany

[3] Institute for Biochemistry and Molecular Biology, Medical Faculty, Rheinische Friedrich-Wilhelms-University of Bonn, Bonn, Germany

[4] Department of Osteology and Biomechanics, Cell Biology of Rare Diseases, University Medical Center Hamburg-Eppendorf, Hamburg, Germany

[5] Morphology and Electron Microscopy, University Medical Center Hamburg-Eppendorf, Center for Molecular Neurobiology (ZMNH), Hamburg, Germany

[6] Department of Microbiology and Immunology, Stanford University School of Medicine, Stanford, CA, USA

[7] Faculty of Health Sciences, Centre for Glycomics, Department of Cellular and Molecular Medicine, University of Copenhagen, Copenhagen, Denmark

‡ Shared first authorship

* To whom correspondence should be addressed:

mvoss@biochem.uni-kiel.de or s.jabs@ikmb.uni-kiel.de

## Appendix Figures (this file)

Fig. S1: Generation of a SPPL3 activity-based rescue model in SPPL3-deficient Flp-In T-REx 293 cells. **p. 2**

Fig. S2: Genome editing in *SPPL3* and B4GALT5 secretion from hTERT RPE-1 cells. **p. 3**

Fig. S3: Generation of GOLPH3/GOLPH3L-deficient HEK293 cells. **p. 4**

Fig. S4: Whole-cell proteome and secretome analyses of *GOLPH3/GOLPH3L* KO and parental HEK293 cells. **p. 6**

Fig. S5: Generation of GOLPH3/GOLPH3L-deficient HAP1 cells. **p. 7**

Fig. S6: Ultrastructural analysis of GOLPH3/GOLPH3L-deficient cells. **p. 8**

Fig. S7: Lysosomal proteins in *GOLPH3/GOLPH3L* KO cells. **p. 10**

Fig. S8: Generation of *GOLPH3* and *GOLPH3/GOLPH3L* KO hTERT RPE-1 cells. **p. 12**

Fig. S9: Immunofluorescence data related to Fig. 5. **p. 13**

Fig. S10: Genotyping of newly established *LYSET* KO HEK293 and hTERT RPE-1 cells. **p. 14**

## Appendix Tables (this file)

Tab. S1: crRNAs and oligos used for CRISPR/Cas9-mediated genome editing and genotyping at target loci. **p. 15**

Tab. S2: Oligos used for molecular cloning. **p. 16**

Tab. S3: Small molecule inhibitors used. **p. 18**

Tab. S4: Primary antibodies used in this study and purchased from commercial sources. **p. 19**

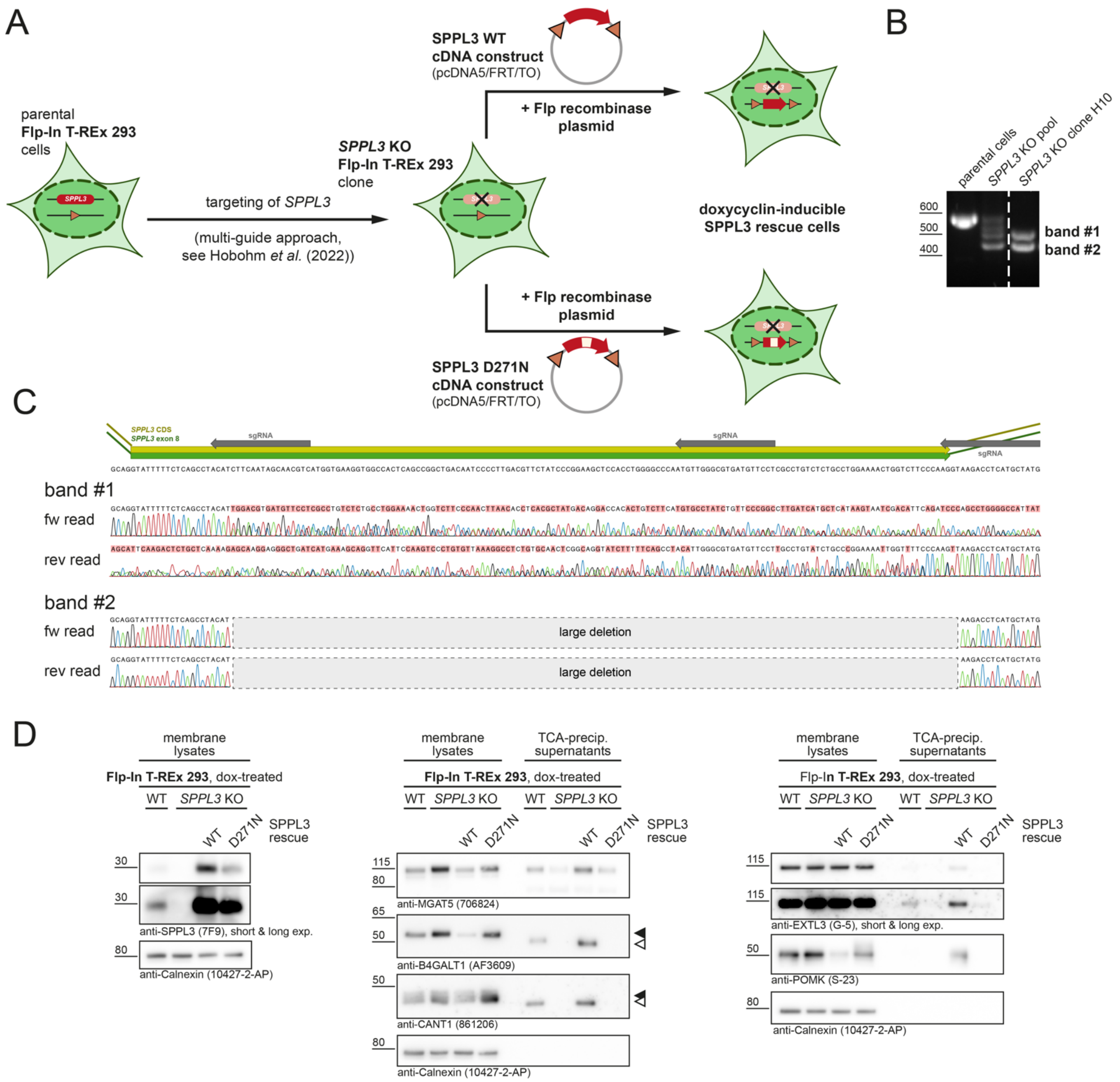


**Appendix Fig. S1**: **Generation of a SPPL3 activity-based rescue model in SPPL3-deficient Flp-In T-REx 293 cells.** (**A**) Schematic overview of the generation of SPPL3-deficient Flp-In T-REx 293 cells with a doxycycline-inducible re-expression of WT human SPPL3 or an inactive variant (D271N). Flp-In T-REx 293 cells were first edited in *SPPL3* using three sgRNAs binding in close proximity to each other in *SPPL3* exon 8 as detailed elsewhere (Hobohm *et al*, 2022). Next, cDNA constructs encoding human SPPL3 WT (top) and catalytically inactive SPPL3 D271N (bottom) were integrated into the validated SPPL3 KO clone (H10) using Flp-mediated integration. (**B**) PCR amplification of a region comprising the sgRNA binding sites in *SPPL3* exon 8 from clone H10. (**C**) Alignment of Sanger sequencing reads to the *SPPL3* reference sequence. Reads were obtained following purification of the two distinctive bands observed for H10 genomic DNA. (**D**) Immunoblot analyses of membrane lysates and TCA-precipitated conditioned supernatants of clones derived from parental (WT) Flp-In T-REx 293 cells as depicted schematically in (**A**). Cells were induced with doxycycline (dox; 100 ng/ml) for 48 hrs before medium was changed to doxycycline-supplemented OptiMEM to collect secreted proteins overnight. Membrane lysates and TCA-precipitated conditioned supernatants were probed for abundance of the indicated proteins.

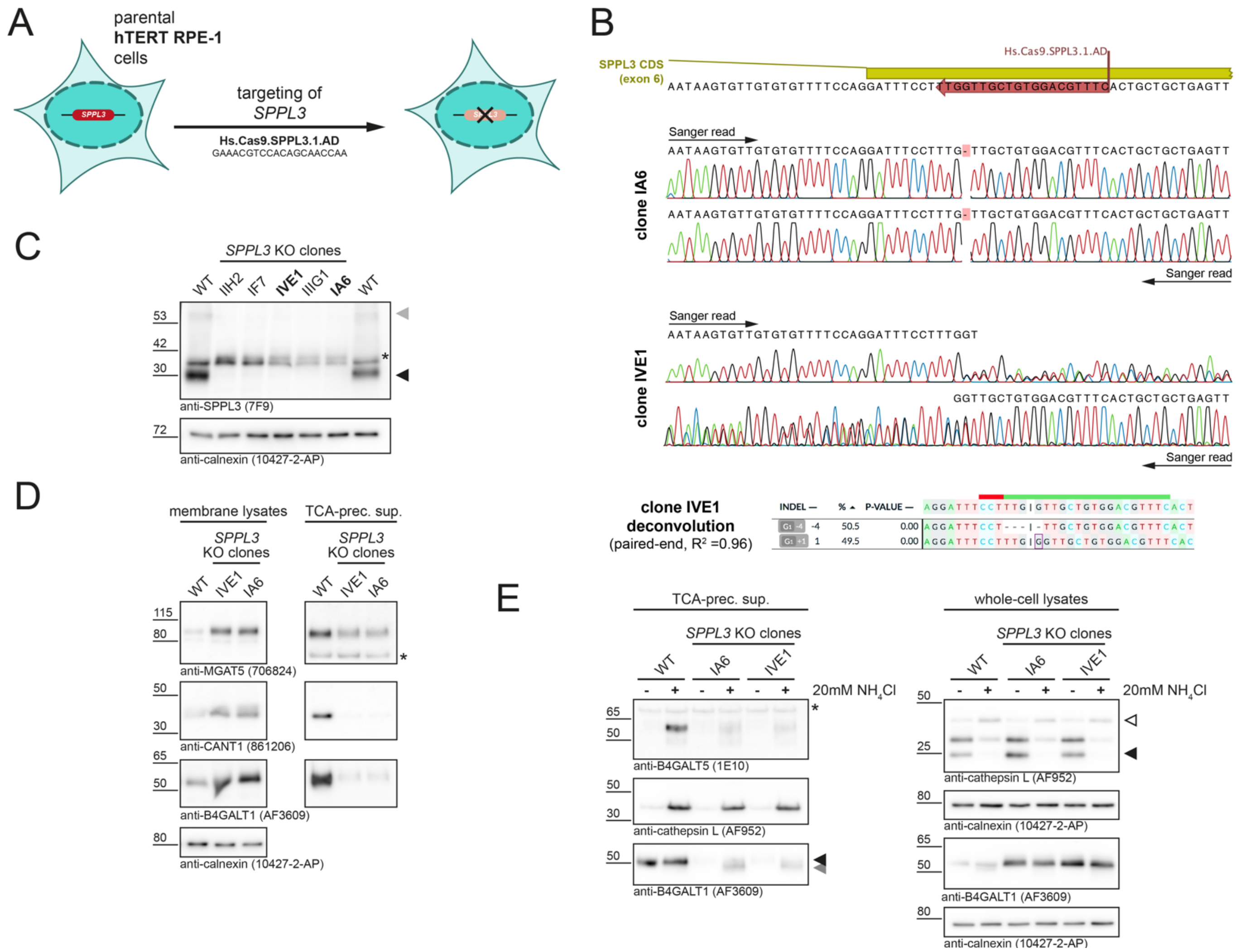


**Appendix Fig. S2**: **Genome editing in *SPPL3* and B4GALT5 secretion from hTERT RPE-1 cells.** (**A**) Schematic overview of the SPPL3 targeting strategy pursued. (**B**) Sanger sequencing results of two hTERT RPE-1 clones successfully edited in *SPPL3*. The reads cover the target region and the crRNA binding site is indicated. Clone IA6 was homozygous for a deletion (top reads). Clone IVE1 was heterozygous based on the Sanger reads (lower reads) and read deconvolution using DECODR predicted two distinct frame-shifting alleles. (**C**) SPPL3 immunoblots of membrane lysates. Clones IVE1 and IA6 used for further experiments are highlighted in bold. Calnexin was used as a loading control. Black arrowheads denote the expected full-length protein bands, the grey arrowhead points to a likely SPPL3 dimer. (**D**) Golgi enzyme levels in membrane lysates and TCA-precipitated conditioned supernatants of parental (WT) or *SPPL3* KO hTERT RPE-1 clones. Note the reduced secretion and intracellular accumulation of SPPL3 substrates in the *SPPL3* KO clones. (**E**) Ammonium chloride-induced secretion of B4GALT5. Cells were treated as indicated and TCA-precipitated conditioned supernatants were analysed by immunoblotting using the indicated antibodies.

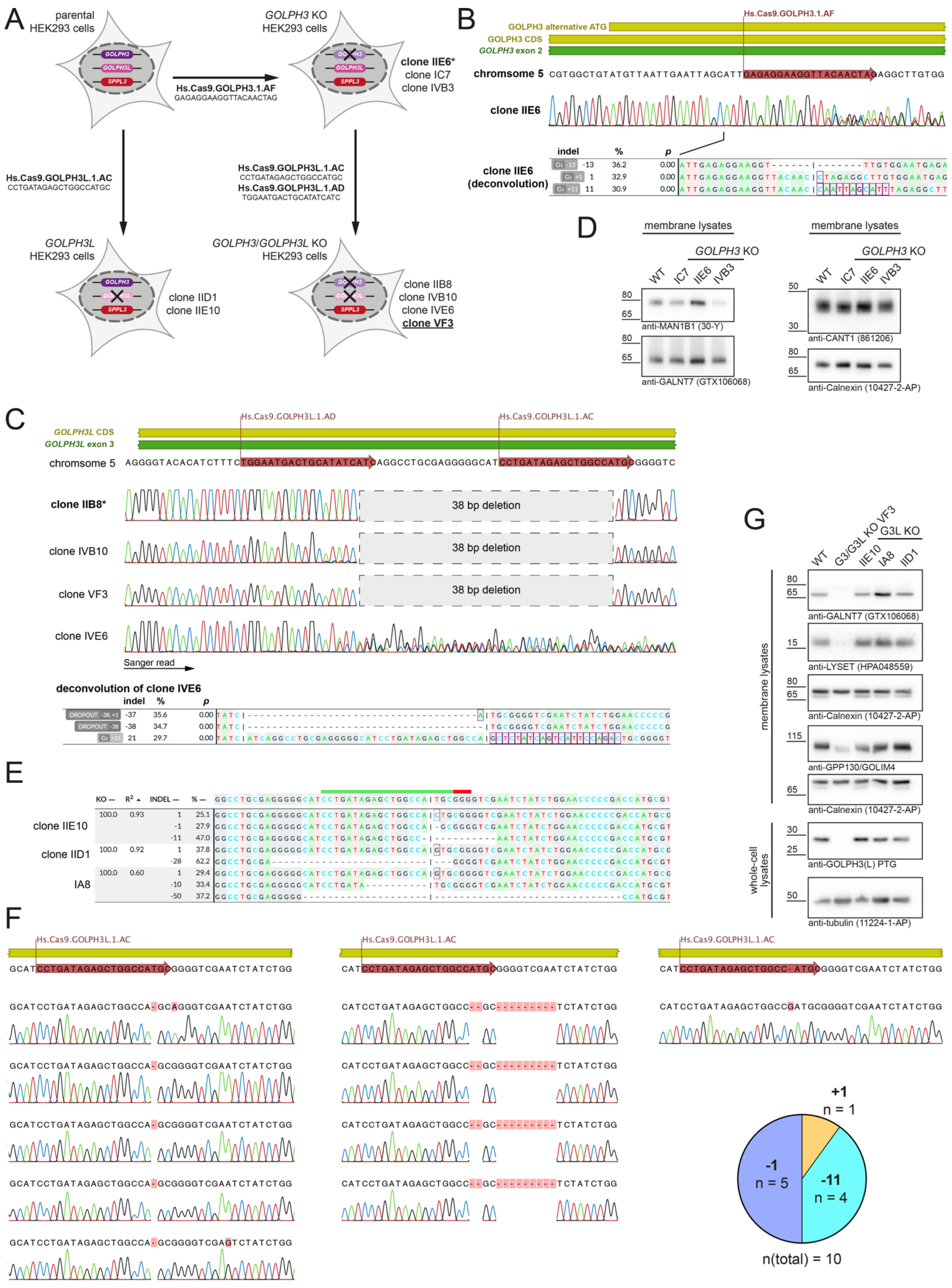


**Suppl. Fig. 3:** *legend on next page*

**Appendix Fig. S3**: **Generation of GOLPH3/GOLPH3L-deficient HEK293 cells.** (**A**) Overview of the genome editing strategy for successive depletion of *GOLPH3* and *GOLPH3L* with indicated targeting sequences. Clones validated and used in experiments in this study are shown. Clones in bold and labelled with an asterisk are the clones subjected to the second round of genome editing. The *GOLPH3/GOLPH3L* KO clone VF3 (bold and underlined) was used to establish stable cell pools with a cumate-inducible GOLPH3 rescue. (**B**) Sanger read obtained from clone IIE6 following genome editing in *GOLPH3* aligned to the Cas9-targeted region in *GOLPH3* (top). Deconvolution of the same read conducted with DECODR (Bloh *et al*, 2021) (bottom). Absence of GOLPH3 protein was confirmed by immunoblotting (Fig. 3E) and clone IIE6 was used to subsequently knock-out *GOLPH3L*. (**C**) Alignment of Sanger reads obtained from the indicated HEK293 clones after the second round of genome editing to the *GOLPH3L* target region. Clone IVE6 was not homozygous for the 38bp deletion and was analysed by deconvolution (bottom). (**D**) Cell-endogenous levels of select established type II membrane protein GOLPH3/GOLPH3L clients in membrane lysates of three *GOLPH3* KO clones and parental HEK293 cells. Calnexin was used as loading control. (**E**) Results of the paired-end deconvolution of forward and reverse Sanger reads of the *GOLPH3L* target region PCR-amplified from DNA of the indicated clones conducted with DECODR (Bloh *et al*, 2021). (**F**) Confirmation of the deconvolution results of *GOLPH3L* KO clone IIE10. The target locus was amplified using primers extended with restriction enzyme cleavage sites and was subsequently cloned into a pcDNA3-derived plasmid. Plasmid DNA was isolated from individual *E. coli* clones and subjected to Sanger sequencing. Only reads of frameshifting indel alleles were observed. (**G**) Abundance of LYSET and select Golgi type II membrane proteins in HEK293 clones edited in only *GOLPH3L* and compared to parental and *GOLPH3/GOLPH3L* KO HEK293 cells. Calnexin was used as loading control.

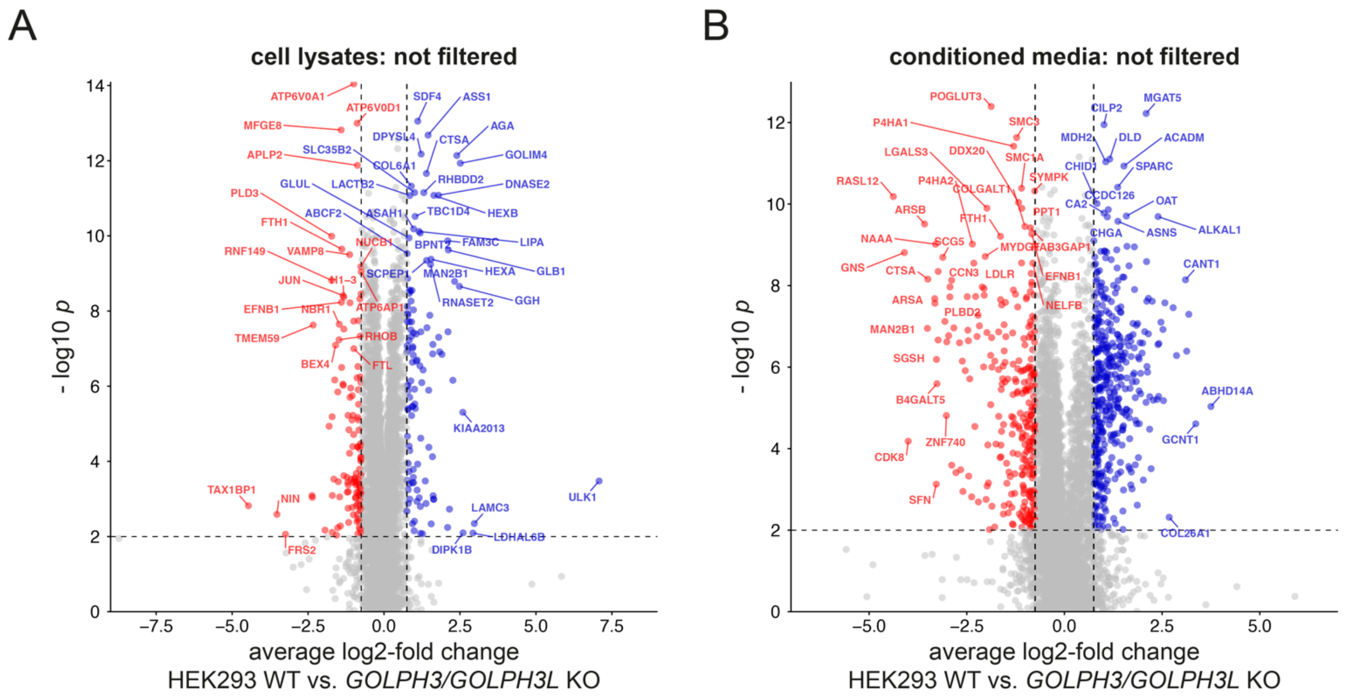


**Appendix Fig. S4**: **Whole-cell proteome and secretome analyses of *GOLPH3/GOLPH3L* KO and parental HEK293 cells.** Four technical replicates of *GOLPH3/GOLPH3L* KO clone VF3 and parental HEK293 cells were analysed. Volcano plots are depicting changes in protein abundance for all proteins quantified in whole-cell lysates (**A**) and conditioned cell culture supernatants (**B**). Significantly ($p < 0.01$) changed (log2-fold change < -0.75 and > 0.75) proteins are coloured in red and blue, respectively, and select data points are labelled with gene names.

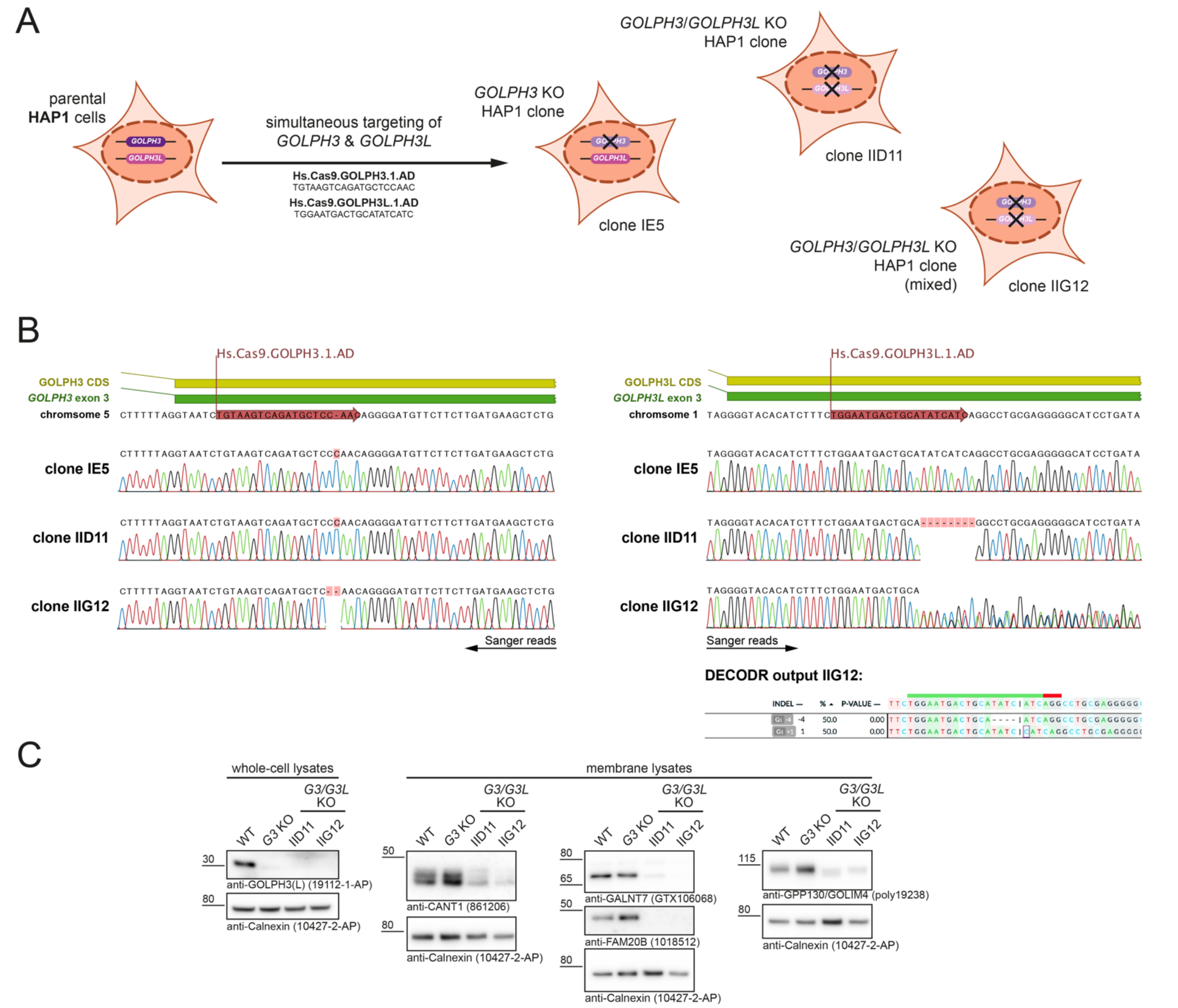


**Appendix Fig. S5**: **Generation of GOLPH3/GOLPH3L-deficient HAP1 cells.** (**A**) Overview of the genome editing strategy in HAP1 cells. *GOLPH3* and *GOLPH3L* were targeted simultaneously (crRNA binding sites are indicated). (**B**) Genotyping of obtained HAP1 clones using PCR amplification and Sanger sequencing demonstrated the presence of frame-shifting +1, +1 and -2 indels in *GOLPH3* in clones IE5, IID11 and IIG12 and no indel mutation in IE5 and a frame-shifting 8-bp deletion in IID11 in *GOLPH3L*. The *GOLPH3L* read for clone IIG12 was ambiguous and deconvolution suggests equal abundance of two distinct indels. IIG12 may thus represent a mixed clone or may have lost haploidy. Both indels detected are frame-shifting and immunoblotting supported absence of GOLPH3L protein (Fig. 3F). (**C**) Levels of select established type II membrane protein GOLPH3/GOLPH3L clients in membrane lysates of WT, *GOLPH3* KO and *GOLPH3/GOLPH3L* KO HAP1 cells. Calnexin was used as loading control.

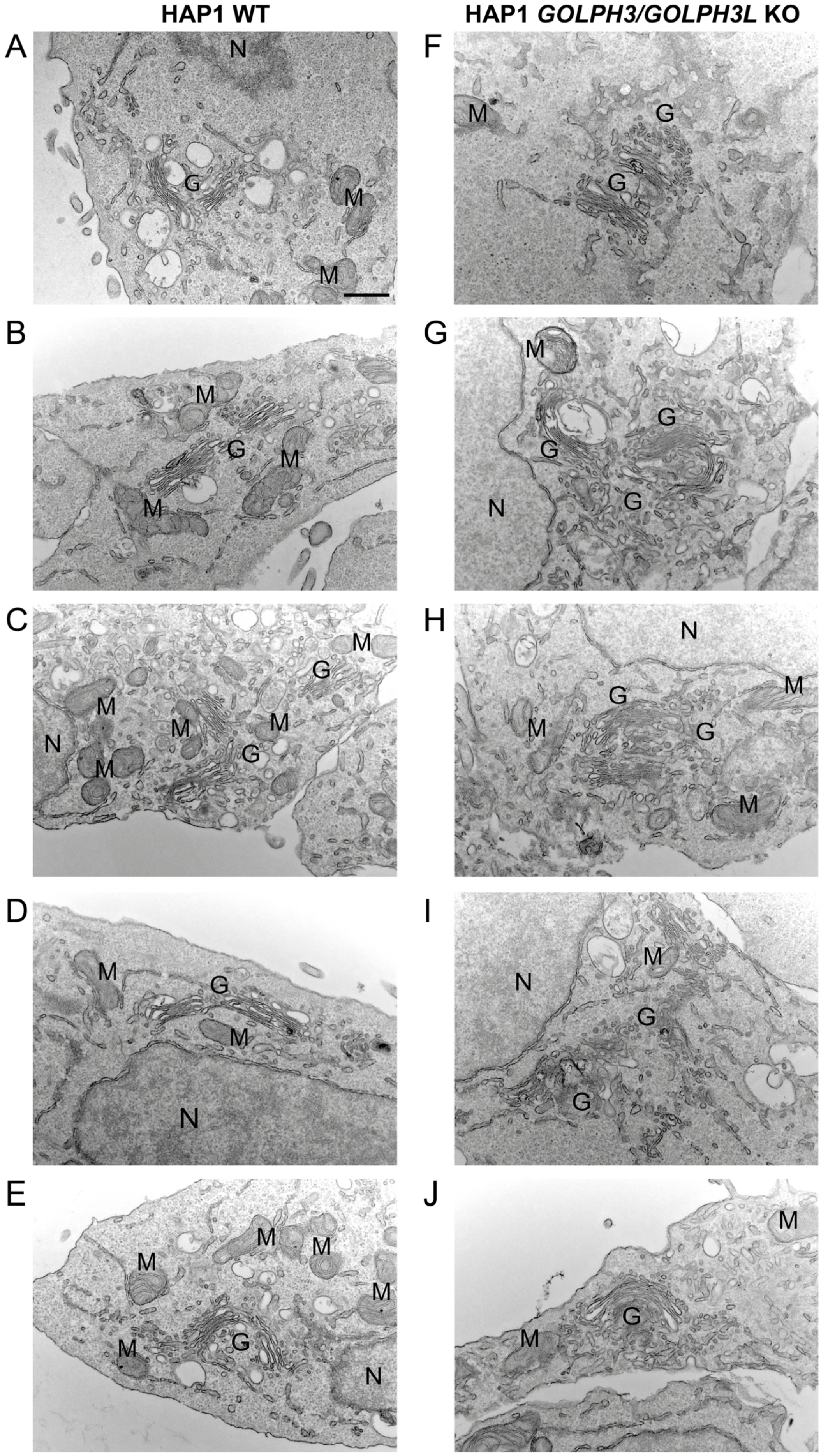


**Suppl. Fig. 6:** *legend on next page*

**Appendix Fig. S6: Ultrastructural analysis of GOLPH3/GOLPH3L-deficient cells.** Representative high-resolution electron micrographs of Golgi complexes in WT and *GOLPH3/GOLPH3L* KO (clone IID11) HAP1 cells. In HAP1 WT cells, flattened membrane stacks (cisternae) and associated transport vesicles as well as larger secretory vesicles are well preserved. In *GOLPH3/GOLPH3L* KO cells the Golgi stacks are dissolved, almost not discernible and surrounded by large numbers of vesicles. G, Golgi apparatus; N, nucleus; M, mitochondrium; scale bar = 500 nm.

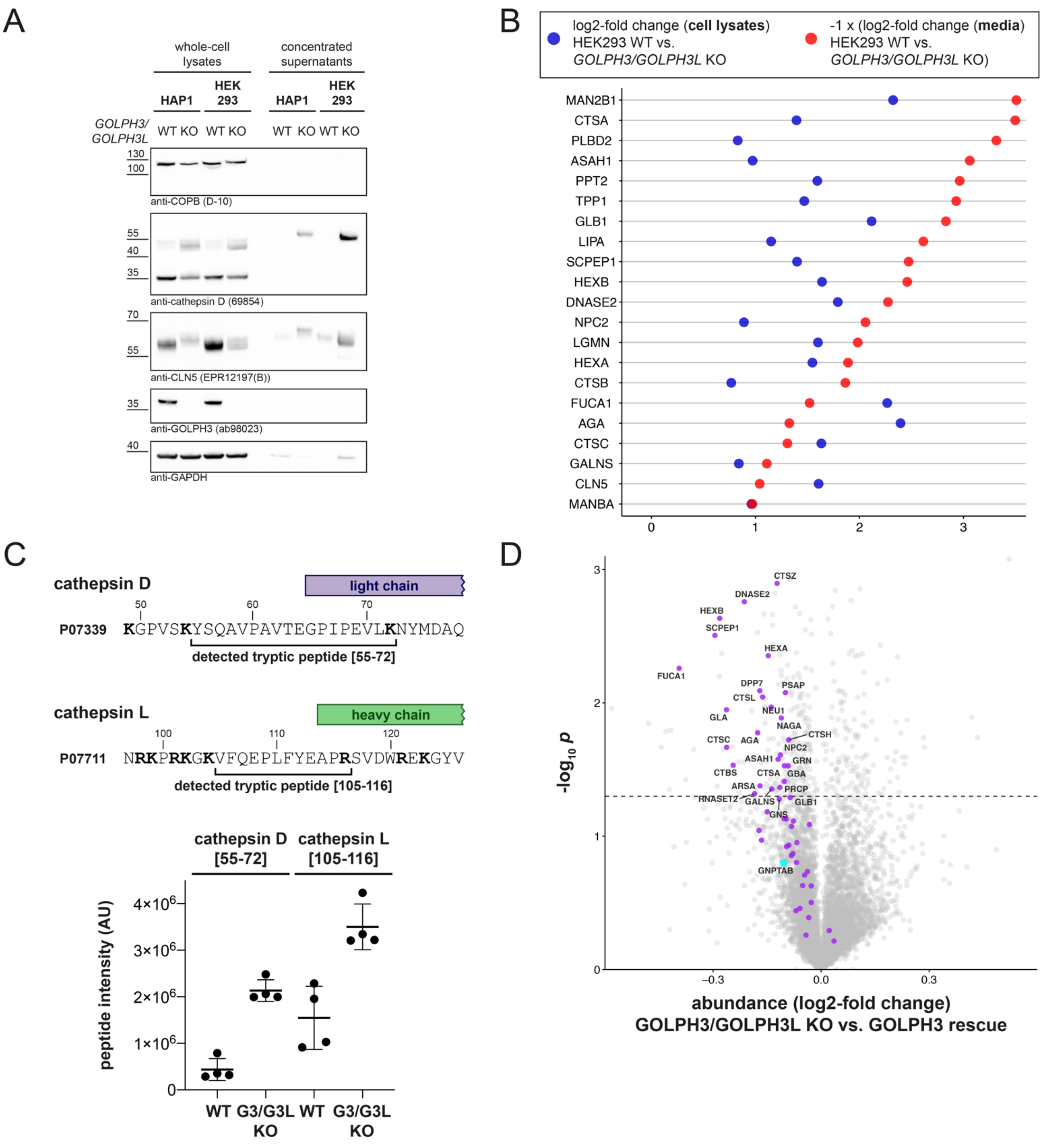


**Appendix Fig. S7: Lysosomal proteins in *GOLPH3/GOLPH3L* KO cells.** (**A**) CLN5 and cathepsin D levels in whole-cell lysates (left) and concentrated conditioned supernatants (right) of WT and *GOLPH3/GOLPH3L* KO HAP1 and HEK293 cells. (**B**) Comparison of absolute log2-fold changes for luminal lysosomal enzymes (labelled by gene name) significantly changed in both proteome (Fig. 4C) and secretome (Fig. 4D) datasets obtained for GOLPH3/GOLPH3L-deficient HEK293 cells. (**C**) Tryptic cathepsin-derived peptides quantified in the secretome dataset (Suppl. Fig. 4B) supporting the predominant secretion of immature lysosomal enzymes from *GOLPH3/GOLPH3L* KO cells. Quantified cathepsin-derived tryptic peptides were manually searched for tryptic peptides spanning regions known to undergo (auto-)proteolytic cleavage events during maturation. Two peptides, mapping to such regions in cathepsin D (Richo & Conner, 1994) and cathepsin L (Ménard *et al*, 1998) were identified and intensity values obtained for these peptides in conditioned media of HEK293 WT and *GOLPH3/GOLPH3L* KO cells are plotted (± SD). (**D**) Depletion of lysosomal proteins from GOLPH3/GOLPH3L-deficient U2-OS cells. Published proteome data were retrieved from (Welch *et al*,

2021). Well-characterized soluble lysosomal proteins (Markmann *et al*, 2017) are shown in purple and labelled with the corresponding gene name in case of a p-value < 0.05 (dashed black line). The α/β subunit of the GNPT complex (GNPTAB) is labelled in cyan. Note that LYSET/TMEM251 was not present in the dataset.

**References (not included in main reference list)**

Ménard R, Carmona E, Takebe S, Dufour É, Plouffe C, Mason P & Mort JS (1998) Autocatalytic Processing of Recombinant Human Procathepsin L: Contribution of both intermolecular and unimolecular events in the processing of procathepsin L in vitro. *J Biol Chem* 273: 4478–4484

Richo GR & Conner GE (1994) Structural requirements of procathepsin D activation and maturation. *J Biol Chem* 269: 14806–14812

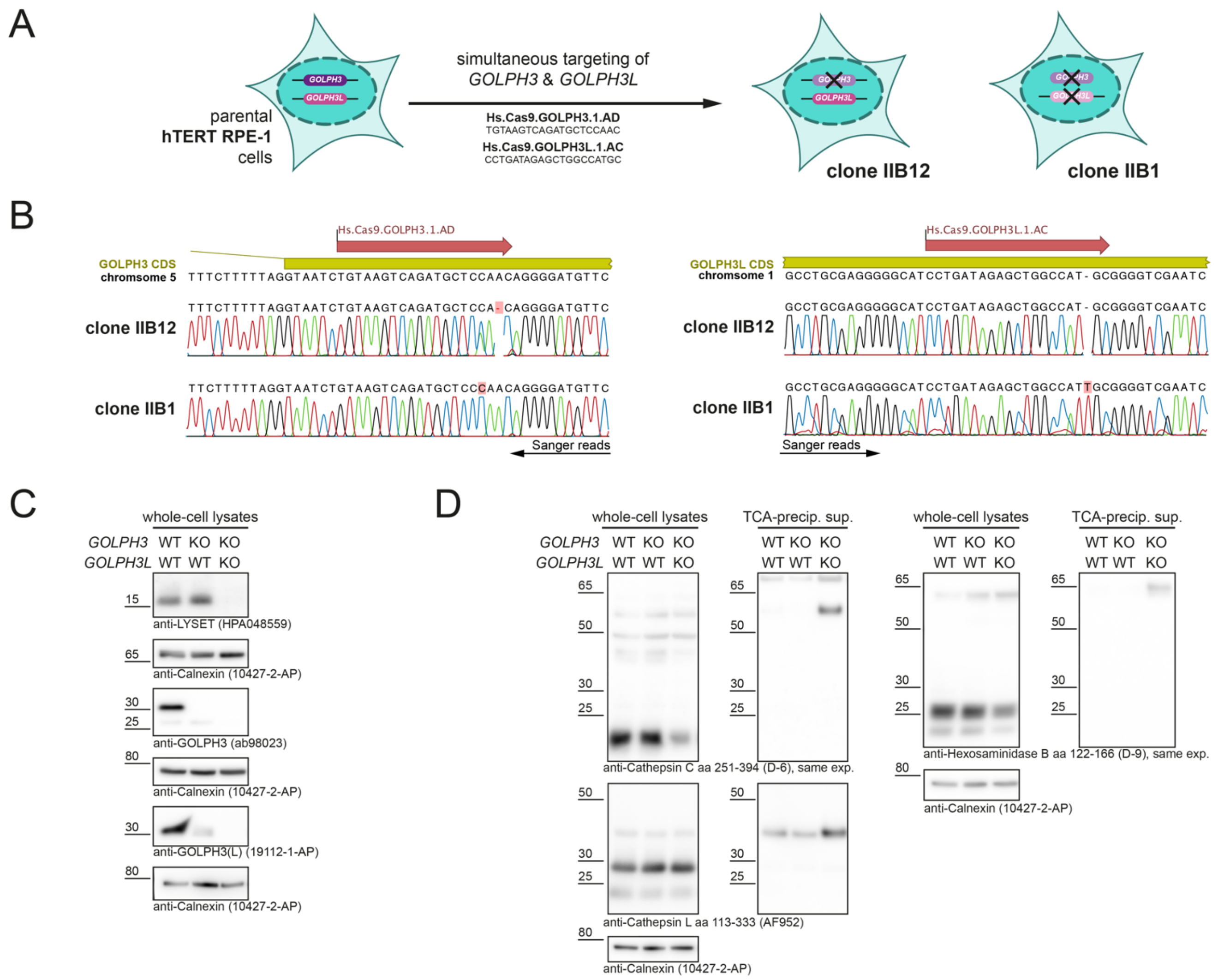


**Appendix Fig. S8: Generation of *GOLPH3* and *GOLPH3/GOLPH3L* KO hTERT RPE-1 cells.** (**A**) Genome editing strategy used to simultaneously target *GOLPH3* and *GOLPH3L*. The schematically shown single-cell-derived clones IIB12 and IIB1 were selected and were found to carry biallelic frame-shifting indels in *GOLPH3* only and in both *GOLPH3* and *GOLPH3L*, respectively. (**B**) Sanger sequencing reads obtained from clones IIB12 and IIB1 aligned to the *GOLPH3* (top) and *GOLPH3L* (bottom) target regions. (**C**) Immunoblot analysis of LYSET and GOLPH3/GOLPH3L levels in whole-cell lysates of parental, *GOLPH3* KO and *GOLPH3/GOLPH3L* KO hTert RPE-1 cells. Calnexin was used as loading control. (**D**) Immunoblot analysis abundance and maturation of cathepsin C, cathepsin L and hexosaminidase B in whole-cell lysates and TCA-precipitated conditioned cell culture supernatants of parental, *GOLPH3* KO and *GOLPH3/GOLPH3L* KO hTert RPE-1 cells. Calnexin was used as loading control.

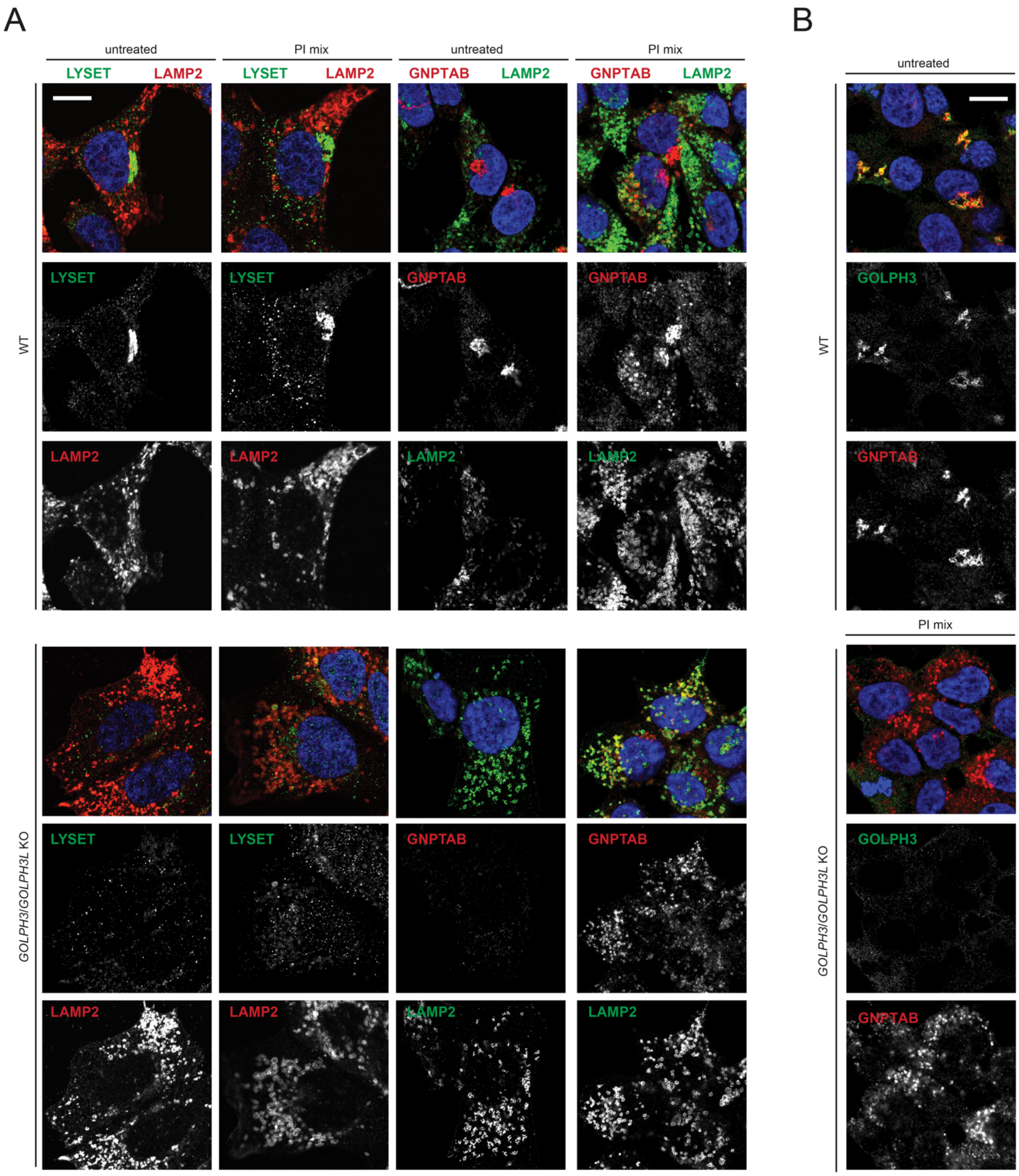


**Appendix Fig. S9: Immunofluorescence data related to Fig. 5.** Single-channel images corresponding to overlay images shown in Fig. 5G (**A**) and Fig. 5H (**B**). DNA staining (Hoechst 33342) is shown in blue; scale bar = 10 µm.

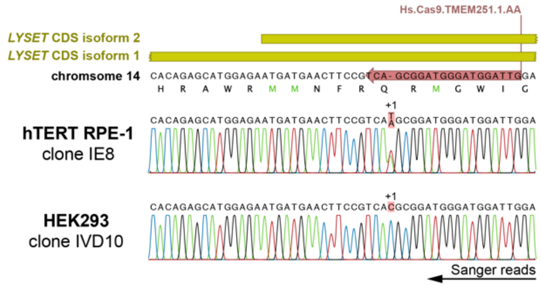


**Appendix Fig. S10: Genotyping of newly established *LYSET* KO hTERT RPE-1 and HEK293 cells.** The *LYSET* target region was PCR-amplified from genomic DNA isolated from the indicated clones and subjected to Sanger sequencing. Obtained reads were aligned to the corresponding region in the human reference genome. Frame-shifting +1 insertions in both clones are highlighted.

**Appendix Tab. S1: crRNAs and oligos used for CRISPR/Cas9-mediated genome editing and genotyping at target loci.** All crRNAs were purchased from Integrated DNA Technologies and the manufacturer's reference ID is provided. All sequences provided are given in 5'>3' orientation. *, Binding site of the two GOLPH3L crRNAs are in close proximity and the very same genotyping strategy was pursued.

| Target locus | crRNA ID | crRNA target sequence<br>genotyping oligo #1 sequence<br>genotyping oligo #2 sequence |
|---|---|---|
| ***GOLPH3*** | Hs.Cas9.GOLPH3.1.AF | GAGAGGAAGGTTACAACTAG<br>TGGAAAGATAAGCCCCCGAAAA<br>TGGCCGGTTACCTTAACAATCA |
| ***GOLPH3*** | Hs.Cas9.GOLPH3.1.AD | TGTAAGTCAGATGCTCCAAC<br>GATACTACATGAAAGCTTGAGGC<br>ACCATTACTTCCCCACTCTATGAC |
| ***GOLPH3L*** | Hs.Cas9.GOLPH3L.1.AC | CCTGATAGAGCTGGCCATGC<br>ACAGCAAACCATGGAAATTCACT*<br>ACCTGTTTAGACAATGGAACTGC* |
| ***GOLPH3L*** | Hs.Cas9.GOLPH3L.1.AD | TGGAATGACTGCATATCATC<br>ACAGCAAACCATGGAAATTCACT*<br>ACCTGTTTAGACAATGGAACTGC* |
| ***SPPL3*** | Hs.Cas9.SPPL3.1.AB | ACACTTGACTGGAATCCACC<br>CCTCATCACCGAGCAAAACCA<br>AGAAGTGTTTAACAGCAGCCAAT |
| ***SPPL3*** | Hs.Cas9.SPPL3.1.AD | GAAACGTCCACAGCAACCAA<br>ATGGAGATGCTGAACGCACTTA<br>AGGTGGGTAAGGGGAATGACC |
| ***LYSET*** | Hs.Cas9.TMEM251.1.AA | CAATCCATCCCATCCGCTGA<br>TTGGAGTAGTTGCCGTGACAG<br>CAGTAGCCCACTGTTTTGGGA<br>**HDR donor:**<br>TTTAATTTGAAGGCACAGAGCATGGAGAATGATGAACTTCGCTCAGGCCATGGGATGGATTGGAGTGGGATTGTATCT<br>GTTAGCCAGTG |

**Appendix Tab. S2: Oligos used for molecular cloning.** In case of oligos used for mutagenesis, bases in lower case correspond to introduced mutations.

| Gene | Purpose of PCR amplification | Oligonucleotide sequences (forward/reverse), 5'>3' |
|---|---|---|
| ***SPPL3*** | Site-directed mutagenesis to introduce the D271N active site mutant | GGGCATCGGAaACATCGTTAT |
| | | AACATGGAGAAGTGGCTG |
| ***GOLPH3*** | Amplification of CDS without C-terminal tag, introduction of HindIII and XhoI sites for insertion into pcDNA3.1 backbone | CTTAAGCTTACCATGACCTCGCTGACCCAGC |
| | | GACTCGAGTCACTTGGTGAACGCCGCCAC |
| | Site-directed mutagenesis to introduce the R14A/R15A mutant | CCTGGTGCAGgctgcCACCGAGGCCTCCCGC |
| | | CCGGAGCTGCGCTGGGTC |
| | Site-directed mutagenesis to introduce the R90L mutant | TCTGGATTACtgGGCTGTATGTTAATTG |
| | | TGATATACAGTCATTCCAAAATG |
| | Amplification of CDS without C-terminal tag, introduction of NheI and NotI sites for insertion into pBQM812A backbone | AGAGCTAGCACCATGACCTCGCTGACCCAGCGCAG |
| | | ATAGCGGCCGCTCACTTGGTGAACGCCGCCAC |
| ***LYSET* isoform 1** | Amplification of CDS, introduction of HindIII and BamHI sites for insertion into pcDNA6 myc/His backbone | CCCAAGCTTATGCCAAAGCCACCCGATTATTC |
| | | CGCGGATCCTTACGTGTCAATCAGTTGTAGTC |
| | Side-directed mutagenesis: R22A | GGGAACAGGAgcgTTTTCGGGACCATTG |
| | | ACGGCAAGCGTTAAAGAGTCAC |
| | Side-directed mutagenesis: R29A | ACCATTGCACgcgGCATGGAGAATG |
| | | CATTCTCCATGCcgcGTGCAATGGT |
| | Side-directed mutagenesis: R32A | CAGAGCATGGGCGATGATGAACTTCC |
| | | TGCAATGGTCCCGAAAATCTTCC |
| | Side-directed mutagenesis: R37A | GATGAACTTCgcTCAGCGGATGGGATG |
| | | ATTCTCCATGCTCTGTGC |
| | Side-directed mutagenesis: R39A | CTTCCGTCAGGCGATGGGATGGATTGG |
| | | TTCATCATTCTCCATGCTCTGTGC |
| | Side-directed mutagenesis: R39W | CTTCCGTCAGtGGATGGGATG |
| | | TTCATCATTCTCCATGCTCTG |
| | Side-directed mutagenesis: R37A/R39A | ggctATGGGATGGATTGGAGTG |
| | | tgagcGAAGTTCATCATTCTCCATG |
| | Side-directed mutagenesis: R37K/R39K | gaagATGGGATGGATTGGAGTG |
| | | tgcttGAAGTTCATCATTCTCCATG |
| | Site-directed mutagenesis: R22A/R37A | GATGAACTTCGCGCAGCGGATGGG |

| | | |
|---|---|---|
| | | ATTCTCCATGCTCTGTGCAATGG |
| | Site-directed mutagenesis: R29A/R37A | GATGAACTTCGCGCAGCGGATGGG |
| | | ATTCTCCATGCTCTGTGCAATGG |
| | Site-directed mutagenesis: R32A/R37A | GATGAACTTCGCGCAGCGGATGGG |
| | | ATCGCCCATGCTCTGTGCAATGG |
| | Site-directed mutagenesis: R22A/R29A | ACCATTGCACgcgGCATGGAGAATG |
| | | CCCGAAAACGCTCCTGTTCCCAC |
| | Site-directed mutagenesis: R22A/R32A | CAGAGCATGGGCGATGATGAACTTCC |
| | | TGCAATGGTCCCGAAAATCTTCC |
| | Site-directed mutagenesis: R29A/R32A | CGCGGCATGGGCGATGATGAACTTC |
| | | CATTCTCCATGCcgcGTGCAATGGT |
| ***LYSET* isoform 2** | Amplification of CDS, introduction of HindIII and BamHI sites for insertion into pcDNA6 myc/His backbone | CCCAAGCTTATGATGAACTTCCGTCAGCGG |
| | | CGCGGATCCTTACGTGTCAATCAGTTGTAGTC |
| ***B4GALT5*** | Amplification of CDS without C-terminal stop codon, introduction into pcDNA3.1-derived backbone with an in-frame C-terminal V5 tag via HindIII and BamHI | CTTAAGCTTACCATGCGCGCCCGCCGGGGGCTG |
| | | AGAGGATCCGTACTCGTTCACCTGAGC |
| | Amplification of CDS without C-terminal stop codon, to insert in-frame into Str-KDEL_ST-SBP-mCherry | GAGGCGCGCCATGCGGGCCCGCCGGGGGCTGCTG |
| | | CATGGAATTCCCGTACTCGTTCACCTGAGCCAGCT |
| ***B4GALT1*** | Amplification of CDS without C-terminal stop codon, introduction into pcDNA3.1-derived backbone with an in-frame C-terminal V5 tag via HindIII and BamHI | AACTTAAGCTTACCATGAGGCTTCGGGAGCCGCTC |
| | | GCCGGATCCGCTCGGTGTCCCGATGTC |
| ***GNPTAB*** | Amplification of CDS without C-terminal stop codon, introduction of 20bp overlapping ends with EcoRV-digested pLenti_CMVTRE3G-Puro-DEST and the synthesized myc-IRES-LYSET-FLAG fragment for Gibson Assembly | TGTGGTGGAATTCTGCAGATACCATGCTGTTCAAGCTCCTGCAGAGAC |
| | | TCTGAGATGAGTTTTTGTTCTACTCTGATTCGATTGGGACTAGCTTCTTTG |

**Appendix Tab. S3: Small molecule inhibitors used.**

| Inhibitor | Target(s)/target process | Source, catalogue number |
|---|---|---|
| Ammonium chloride | Lysosomal acidification, M6P/M6P receptor interaction | Carl Roth |
| Bafilomycin A1 | Lysosomal acidification | Cayman Chemical |
| E-64d | Cysteine proteases | Cayman Chemical<br>Sigma-Aldrich |
| Leupeptin (hemisulfate) | Cystein/serine/threonine proteases | Carl Roth<br>Sigma-Aldrich |
| Pepstatin A | Aspartyl proteases | Carl Roth<br>Roche |

**Appendix Tab. S4: Primary antibodies used in this study and purchased from commercial sources.** For monoclonal antibodies clone name/number are provided, for polyclonal antibodies the vendor's respective catalogue number. WB, Western blot; IF, immunofluorescence.

| Antigen | Source | Clone / Catalogue # | Host species | Application & dilution |
|---|---|---|---|---|
| Actin | Sigma-Aldrich | A5316 | mouse | WB: 1:5000 |
| B4GAT1 | R&D systems | 724057 | mouse | WB: 1:1,000 |
| B4GALT1 | R&D systems | AF3609 | goat | WB: 1:2,000 |
| Calnexin | Protein Tech Group | 10427-2 | rabbit | WB: 1:25,000 |
| CANT1 | R&D systems | 861206 | mouse | WB: 1:1,000 |
| cathepsin B | Biolegend | W20149K | rat | WB: 1:1,000 |
| cathepsin C | Santa Cruz Biotechnologies | D-6 | mouse | WB: 1:1,000 |
| Cathepsin D | Cell Signaling Technology | 69854 | rabbit | WB: 1:1,000 |
| Cathepsin L | R&D systems | AF952 | goat | WB: 1:1,000 |
| CLN5 | Abcam | EPR12197(B) | rabbit | WB: 1:1,000 |
| COPB | Santa Cruz Biotechnologies | D-10 | mouse | WB: 1:1,000 |
| COG3 | Protein Tech Group | 11130-1-AP | rabbit | WB: 1:2,000 |
| EXTL3 | Santa Cruz Biotechnologies | G-5 | mouse | WB: 1:1,000 |
| FLAG tag | Sigma-Aldrich | M2 / F1804 | mouse | WB: 1:1,000 |
| GALNT2 | Sigma-Aldrich | HPA011222 | rabbit | WB: 1:1,000 |
| GALNT7 | Genetex | GTX106068 | rabbit | WB: 1:3,000 |
| GM130 | BD Biosciences | 35 | mouse | WB: 1:1,000<br>IF: 1:100 |
| GM130 | Cell Signaling Technology | 12480 | rabbit | WB: 1:1,000<br>IF: 1:100 |
| GOLGA3 | Protein Tech Group | 21193-1-AP | rabbit | WB: 1:3,000 |
| GOLGA5 | Genetex | GT2726 | mouse | WB: 1:1,000 |
| GOLPH3 | Abcam | ab98023 | rabbit | WB: 1:1,000<br>IF: 1:100 |
| GOLPH3/GOLPH3L | Protein Tech Group | 19112-1-AP | rabbit | WB: 1:2,000<br>IP: see methods section |
| Golgin 97 | Thermo Fisher Scientific | A21270 | mouse | WB: 1:500 |
| GPP130/GOLIM4 | Biolegend | poly19238 / #923801 | rabbit | WB: 1:1,000 |
| GRASP55 | Protein Tech Group | 10598-1-AP | rabbit | WB: 1:1,000 |

| | | | | |
|---|---|---|---|---|
| GRASP65 | Santa Cruz Biotechnologies | D12 | mouse | WB: 1:1,000 |
| GS27 | BD Biosciences | 25 | mouse | WB: 1:1,000 |
| GS28 | BD Biosciences | 1 | mouse | WB: 1:1,000 |
| HEXB | Santa Cruz Biotechnologies | D-9 | mouse | WB: 1:1,000 |
| LAMP1 | Developmental Studies Hybridoma Bank | H4A3 | mouse | WB: 1:1,000 |
| LAMP1 | Abcam | ab24170 | rabbit | WB 1:1,000 |
| LAMP2 | Developmental Studies Hybridoma Bank | H4B4 | mouse | WB: 1:1,000 |
| LYSET (TMEM251) | Thermo Fisher Scientific | PA5-61769 | rabbit | WB: 1:1,000<br>IF: 1:100 |
| LYSET (TMEM251) | Human Proteome Atlas | HPA048559 | rabbit | WB: 1:1,000 |
| MAN1B1 | Santa Cruz Biotechnologies | 30-Y | mouse | WB: 1:500 |
| mCherry | Chromotek | 6G6 | mouse | WB: 1:1,500 |
| MGAT5 | R&D systems | 706824 | mouse | WB: 1:1,000 |
| myc tag | Cell Signaling Technology | 9B11 / #2276 | mouse | WB: 1:1,000 |
| POMK | Santa Cruz Biotechnologies | S-23 | mouse | WB: 1:1,000 |
| SPPL3 | Sigma-Aldrich | 7F9 | mouse | WB: 1:1,000 |
| TGN46 | Abcam | ab50595 | rabbit | WB: 1:1,000 |
| TM9SF2 | Thermo Fisher Scientific | PA5-48517 | rabbit | WB: 1:1,000 |
| TM9SF4 | Protein Tech Group | 25595-1-AP | rabbit | WB: 1:1,000 |
| tubulin | Protein Tech Group | 11224-1-AP | rabbit | WB: 1:20,000 |
| V5 tag | Thermo Fisher Scientific | R960-25 | rabbit | WB: 1:5,000 |
