# Supplementary figures and images for: GOLPH3 and GOLPH3L maintain Golgi localization of LYSET and a functional mannose 6-phosphate transport pathway (part 1 of 4)
Source: EMBO J. 2024 Nov 25;43(24):6264–90. doi: 10.1038/s44318-024-00305-z (PMC11649813; doi:10.1038/s44318-024-00305-z)

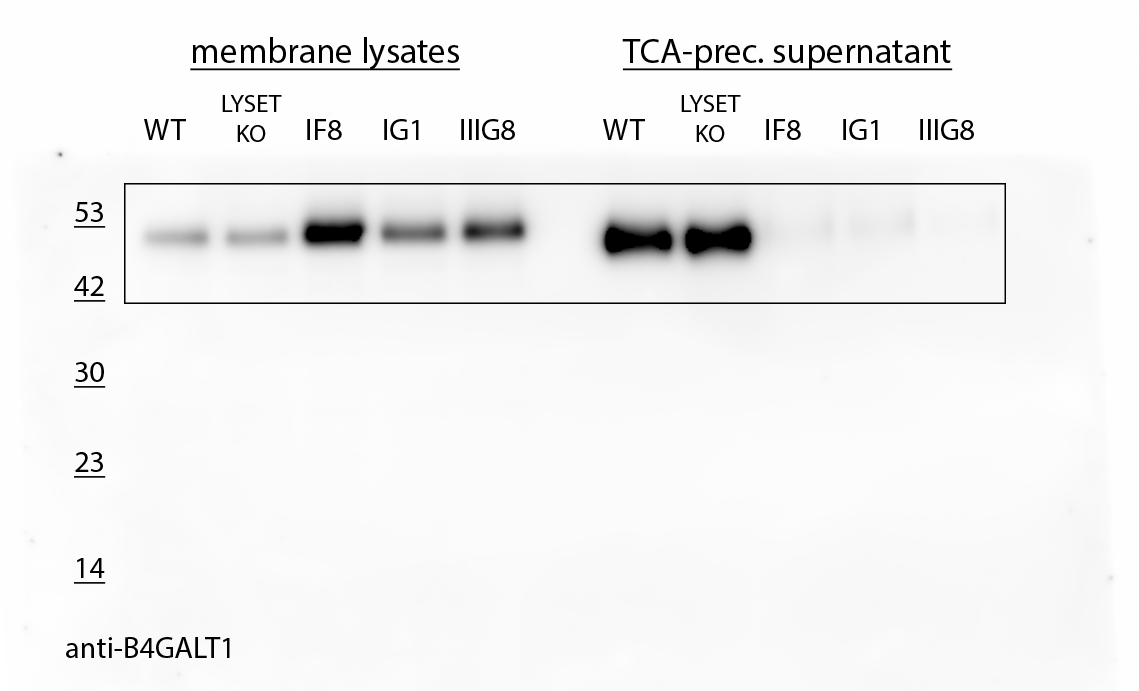

Supplement: Supplementary file 4 — Source data Fig. 1 [file 44318_2024_305_MOESM4_ESM.zip › Figure 1/1F/source data B4GALT1 time series 20230525_150829-06_Ch_Chemi.tif]

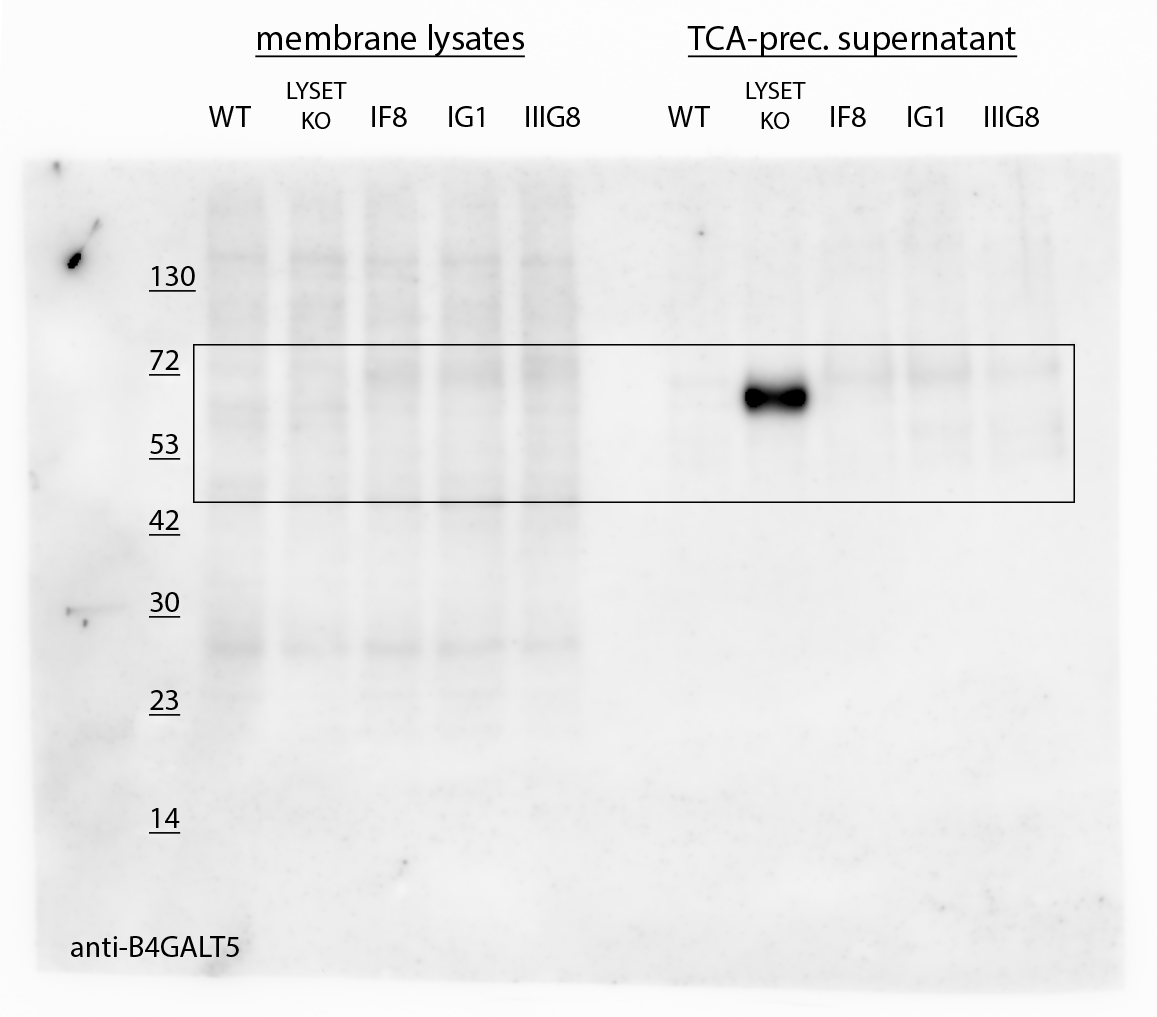

Supplement: Supplementary file 4 — Source data Fig. 1 [file 44318_2024_305_MOESM4_ESM.zip › Figure 1/1F/source data B4GALT5 time series 20230525_150016-20_Ch_Chemi.tif]

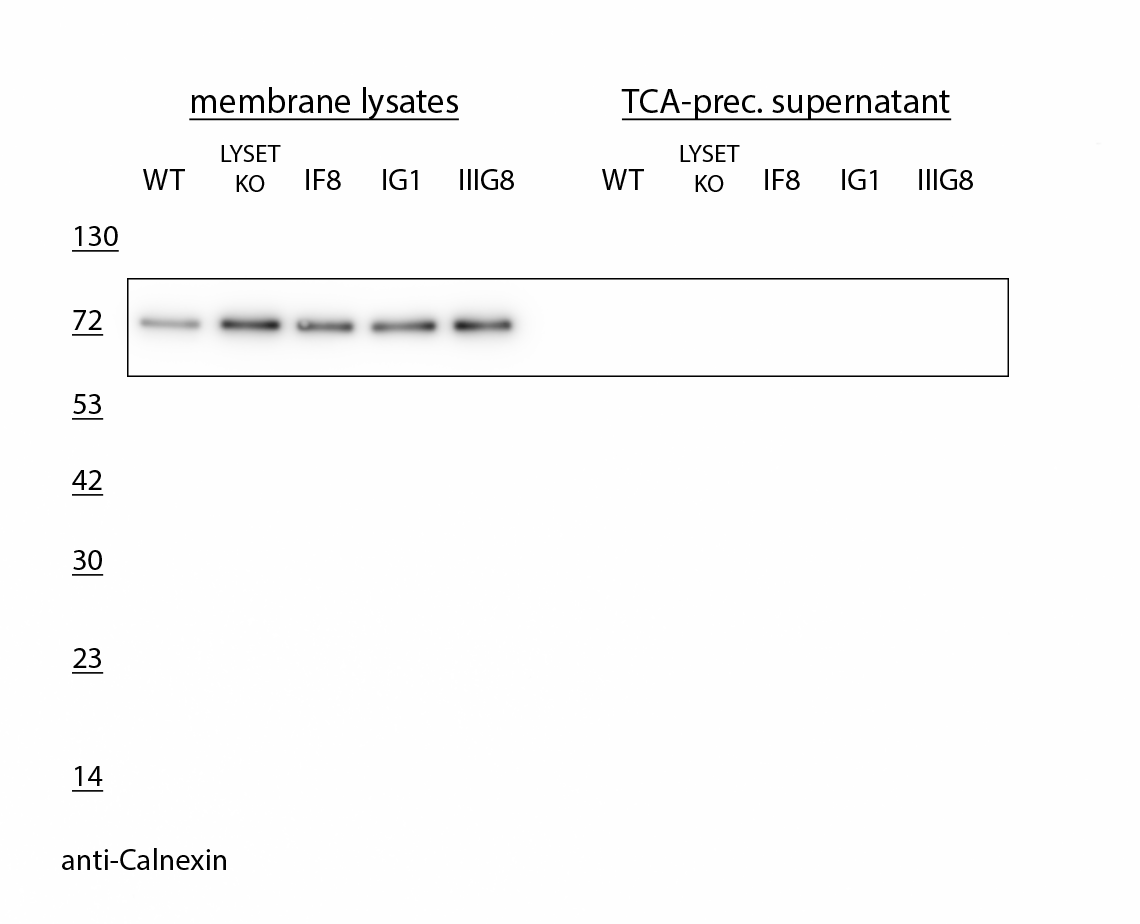

Supplement: Supplementary file 4 — Source data Fig. 1 [file 44318_2024_305_MOESM4_ESM.zip › Figure 1/1F/source data Calnexin for B4GALT5 20240229_142800-05_Ch_Chemi.tif]

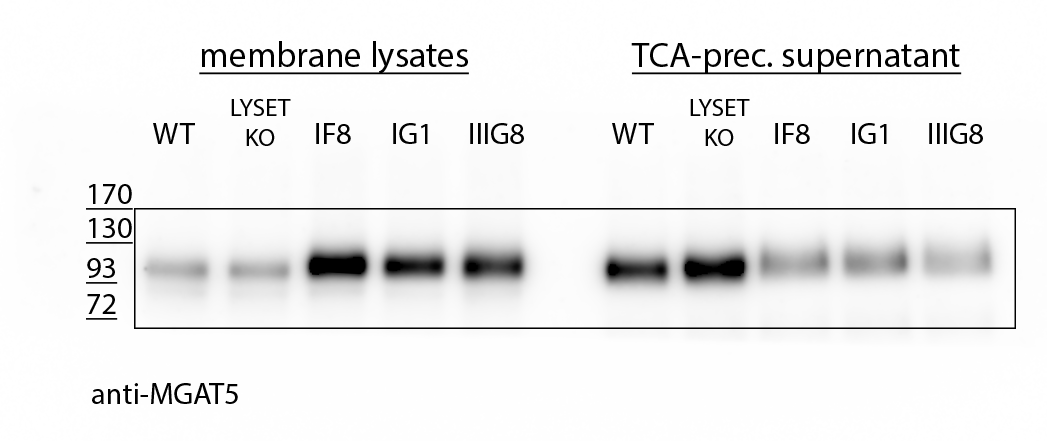

Supplement: Supplementary file 4 — Source data Fig. 1 [file 44318_2024_305_MOESM4_ESM.zip › Figure 1/1F/source data MGAT5 time series 20230525_151647-05_Ch_Chemi.tif]

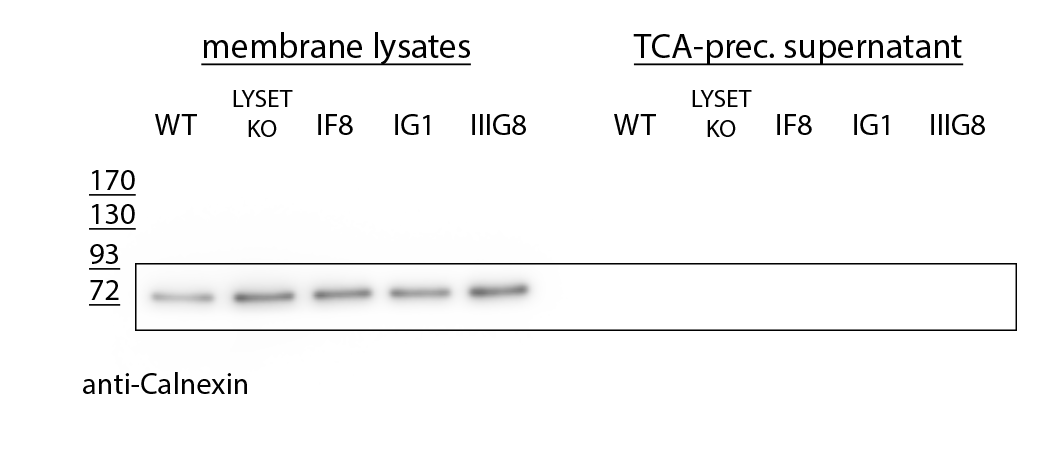

Supplement: Supplementary file 4 — Source data Fig. 1 [file 44318_2024_305_MOESM4_ESM.zip › Figure 1/1F/source data Calnexin for MGAT5, B4GALT1, GALNT2 20240327_164058-01_Ch_Chemi.tif]

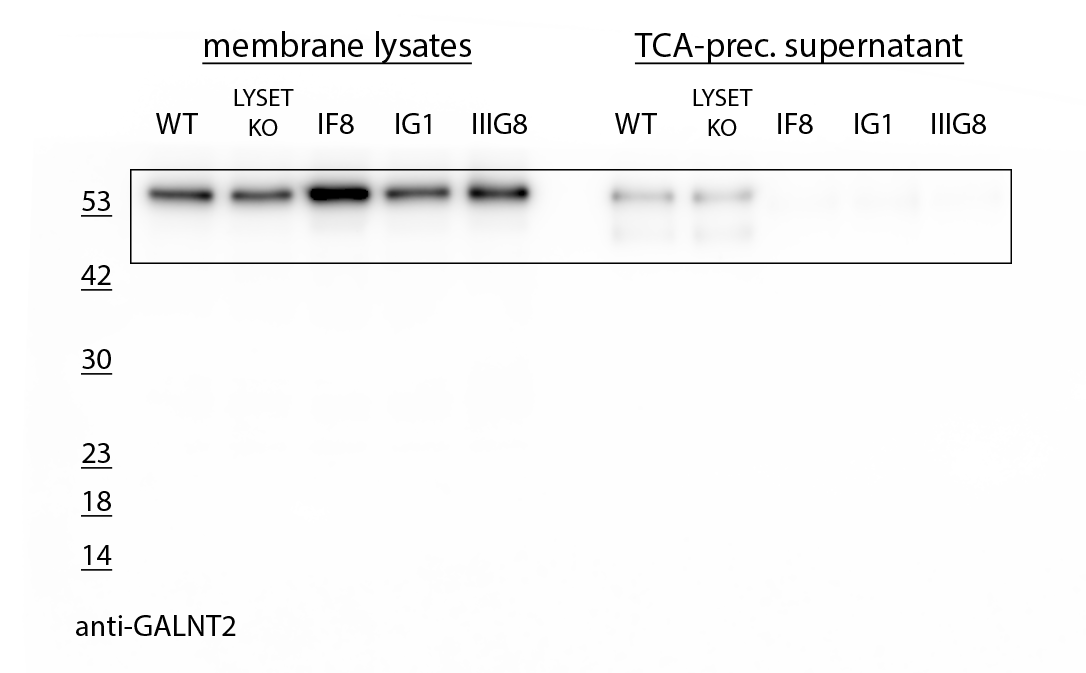

Supplement: Supplementary file 4 — Source data Fig. 1 [file 44318_2024_305_MOESM4_ESM.zip › Figure 1/1F/source data GALNT2 time series 20230530_132850-01_Ch_Chemi.tif]

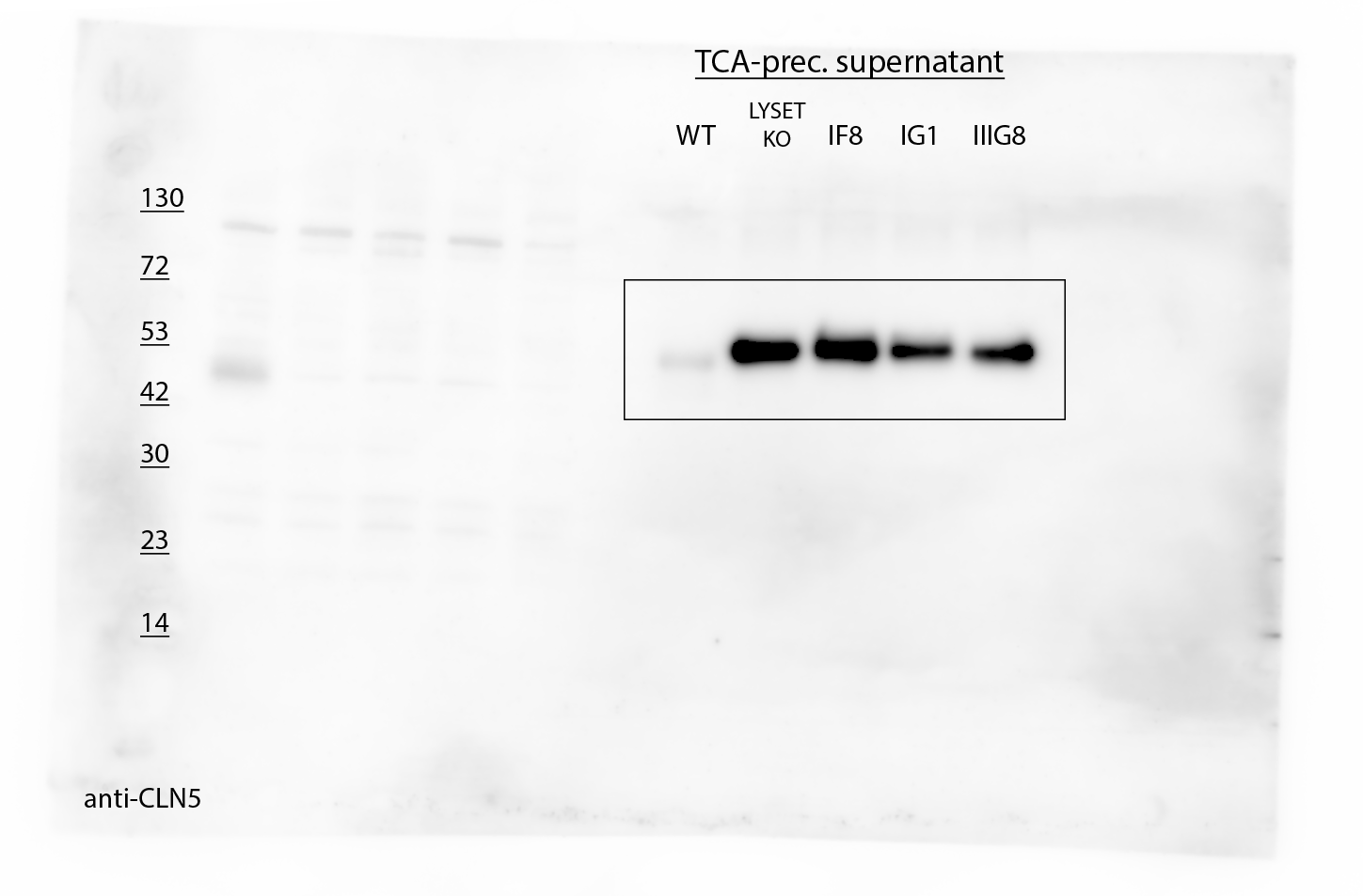

Supplement: Supplementary file 4 — Source data Fig. 1 [file 44318_2024_305_MOESM4_ESM.zip › Figure 1/1G/source data CLN5 time series 20230629_150354-06_Ch_Chemi.tif]

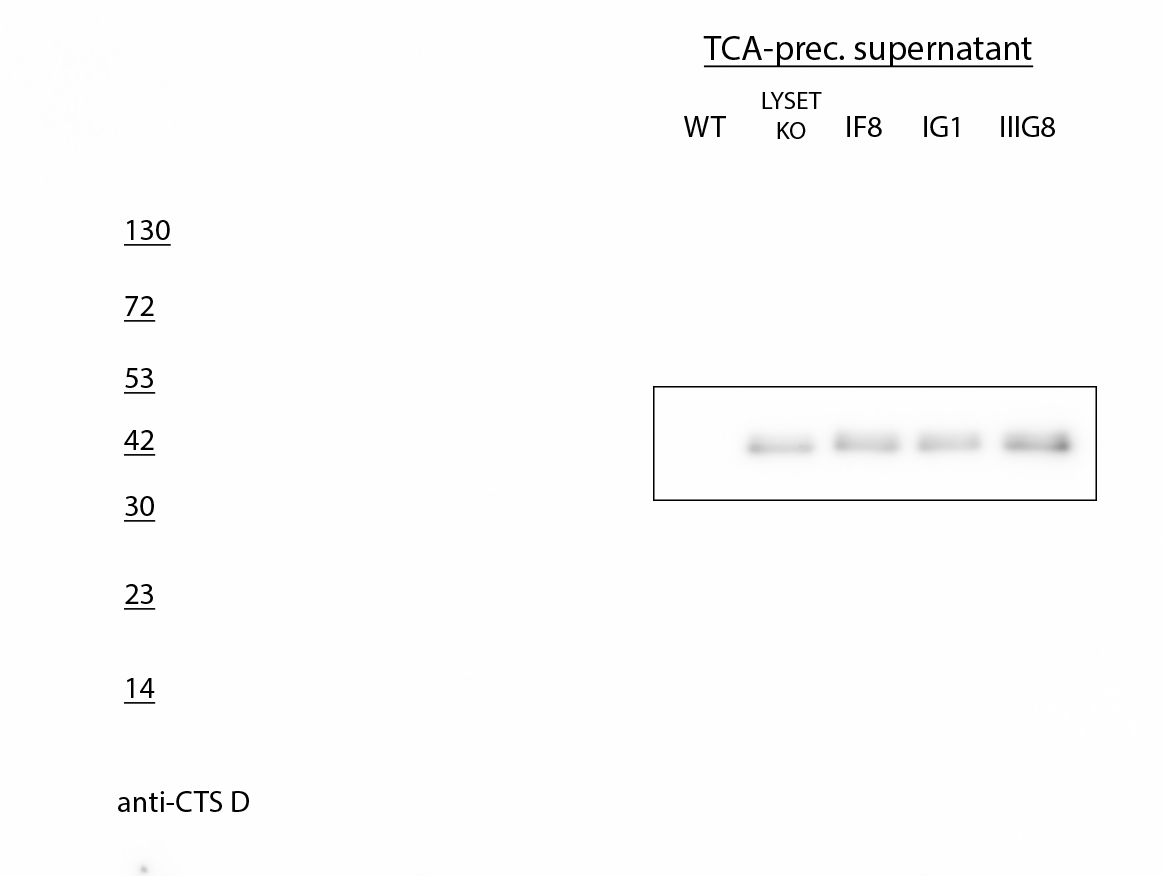

Supplement: Supplementary file 4 — Source data Fig. 1 [file 44318_2024_305_MOESM4_ESM.zip › Figure 1/1G/source data CTS D time series 20230705_144158-18_Ch_Chemi.tif]

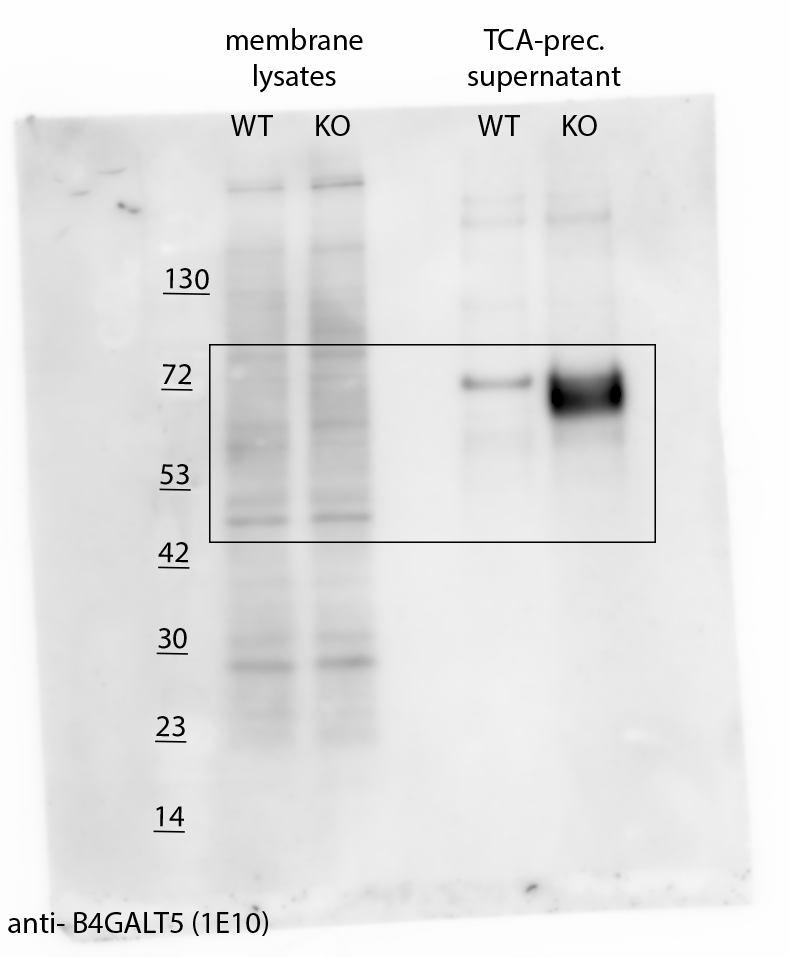

Supplement: Supplementary file 4 — Source data Fig. 1 [file 44318_2024_305_MOESM4_ESM.zip › Figure 1/1B/source data B4GALT5 1E10 time series 20230414_142523-06_Ch_Chemi.tif]

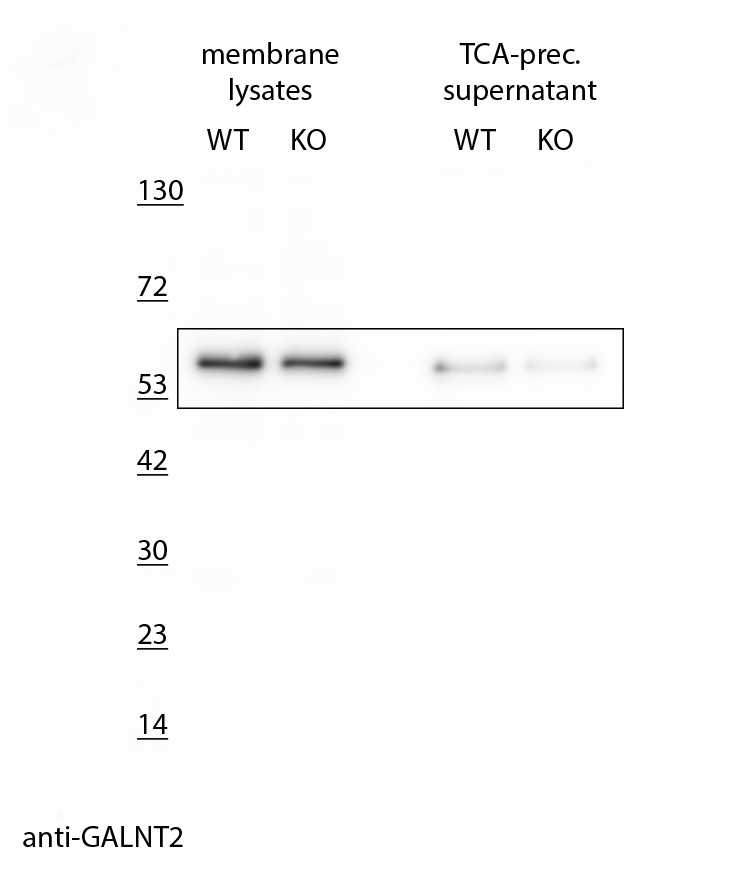

Supplement: Supplementary file 4 — Source data Fig. 1 [file 44318_2024_305_MOESM4_ESM.zip › Figure 1/1B/source data GALNT2 time series 20230426_132009-02_Ch_Chemi.tif]

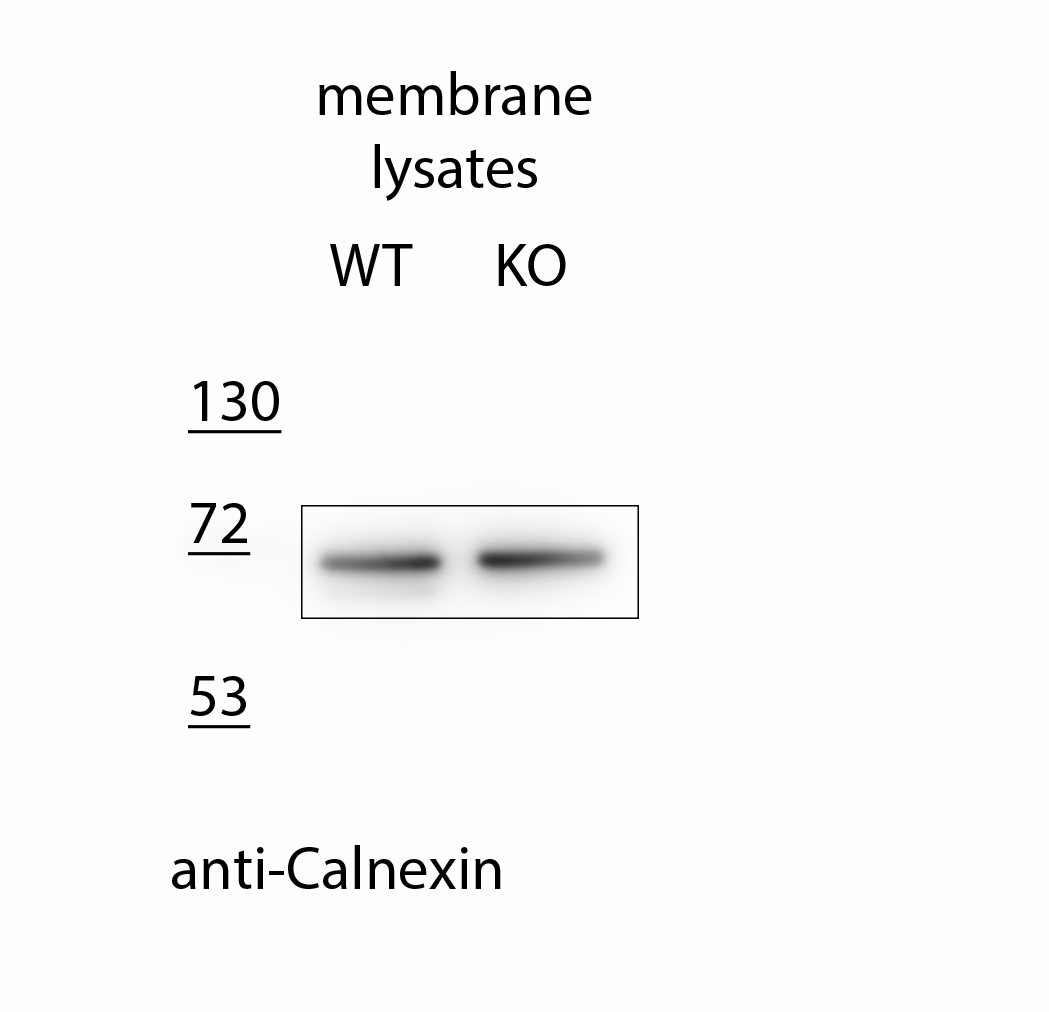

Supplement: Supplementary file 4 — Source data Fig. 1 [file 44318_2024_305_MOESM4_ESM.zip › Figure 1/1B/source data Calnexin for LYSET 8s 20230414_145554_Ch_Chemi.tif]

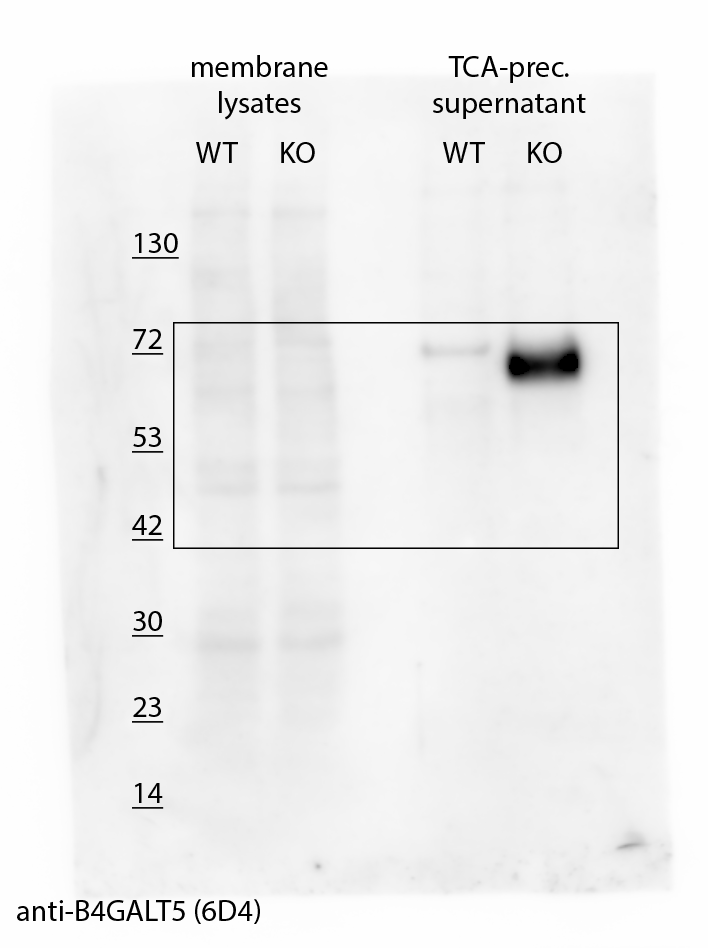

Supplement: Supplementary file 4 — Source data Fig. 1 [file 44318_2024_305_MOESM4_ESM.zip › Figure 1/1B/source Data B4GALT5 6D4 time series 20230414_141731-06_Ch_Chemi.tif]

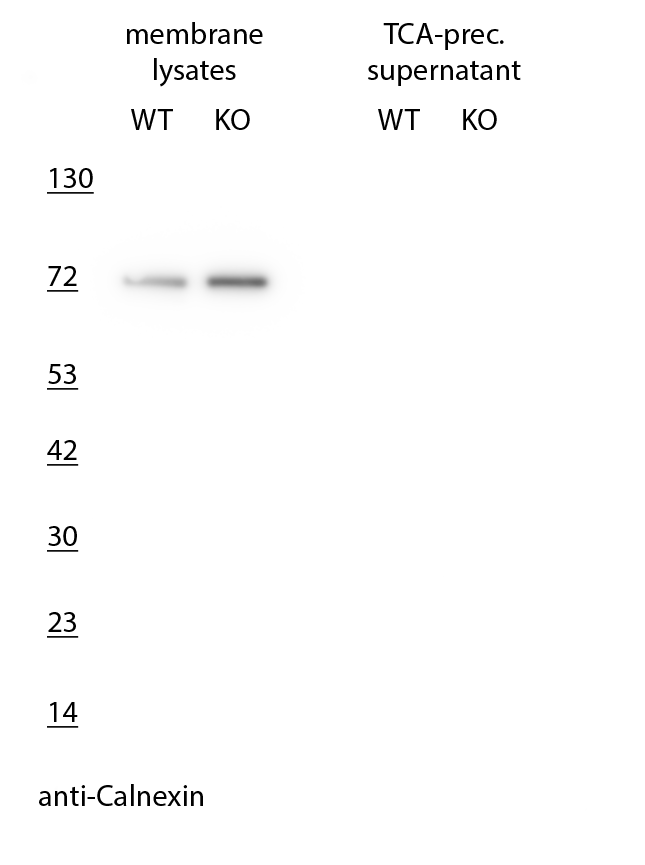

Supplement: Supplementary file 4 — Source data Fig. 1 [file 44318_2024_305_MOESM4_ESM.zip › Figure 1/1B/source data Calnexin for B4GALT5 (1E10), GALNT2 20240227_162812-02_Ch_Chemi.tif]

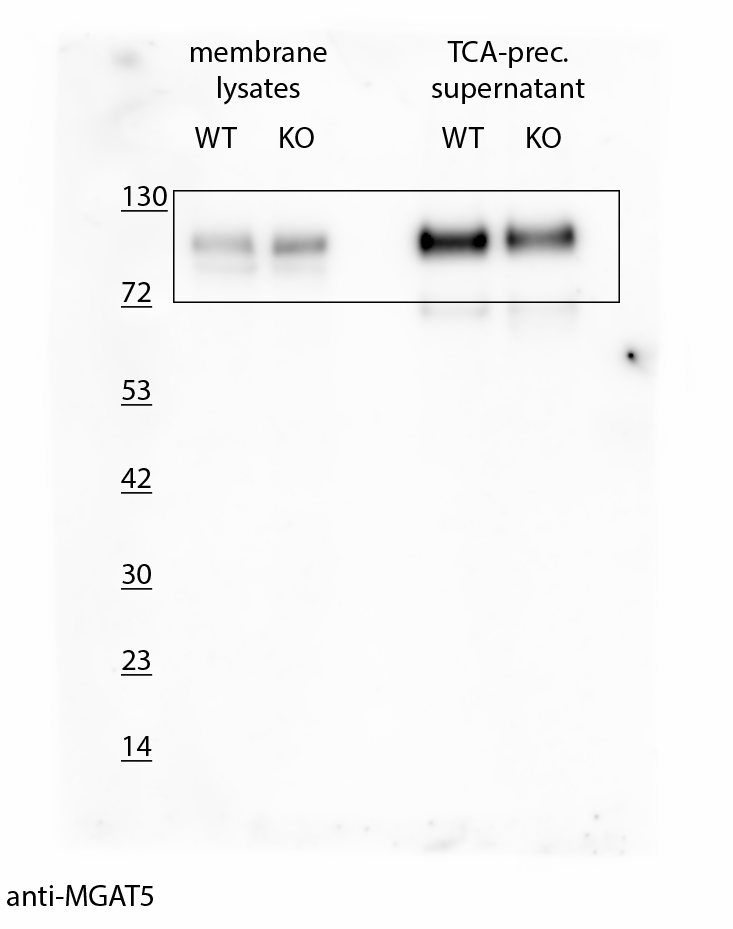

Supplement: Supplementary file 4 — Source data Fig. 1 [file 44318_2024_305_MOESM4_ESM.zip › Figure 1/1B/source data MGAT5 time series 20230418_114414-05_Ch_Chemi.tif]

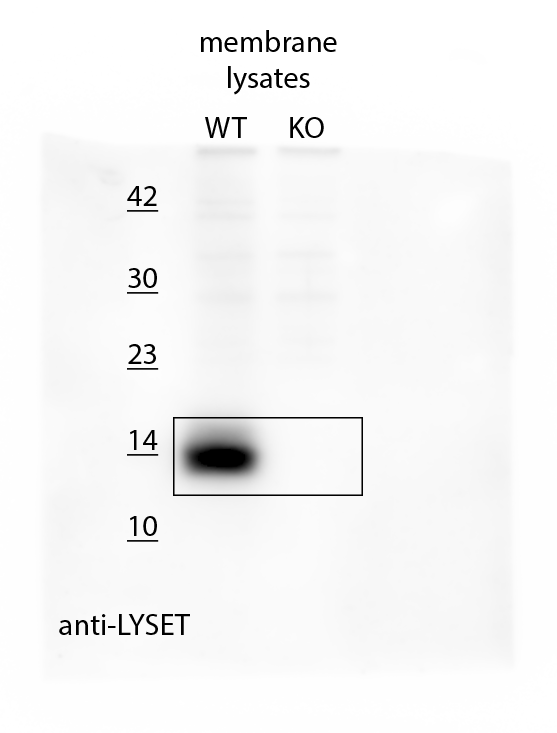

Supplement: Supplementary file 4 — Source data Fig. 1 [file 44318_2024_305_MOESM4_ESM.zip › Figure 1/1B/source data LYSET time series 20230414_144511-03_Ch_Chemi.tif]

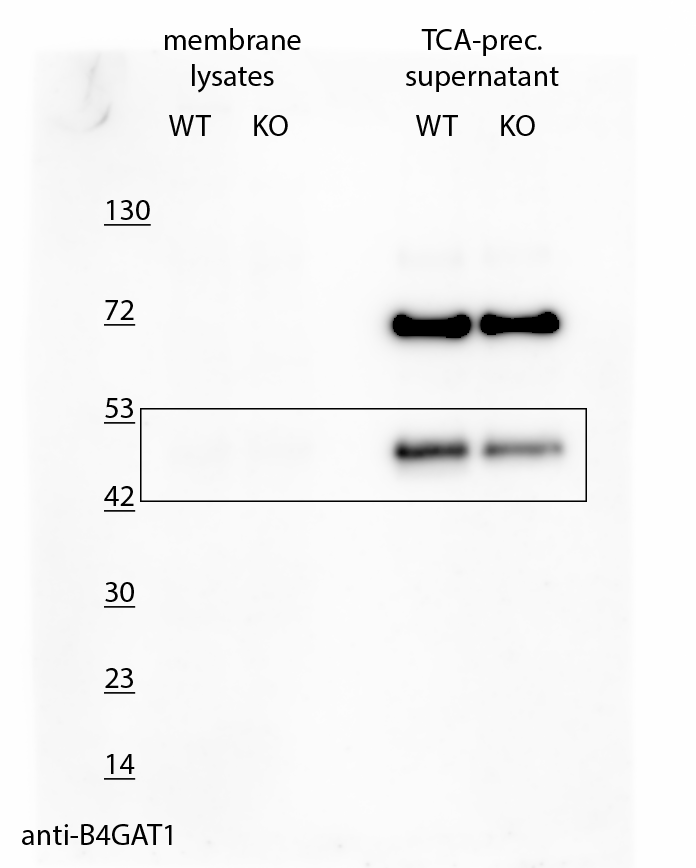

Supplement: Supplementary file 4 — Source data Fig. 1 [file 44318_2024_305_MOESM4_ESM.zip › Figure 1/1B/source data B4GAT1 time series 20230426_124259-05_Ch_Chemi.tif]

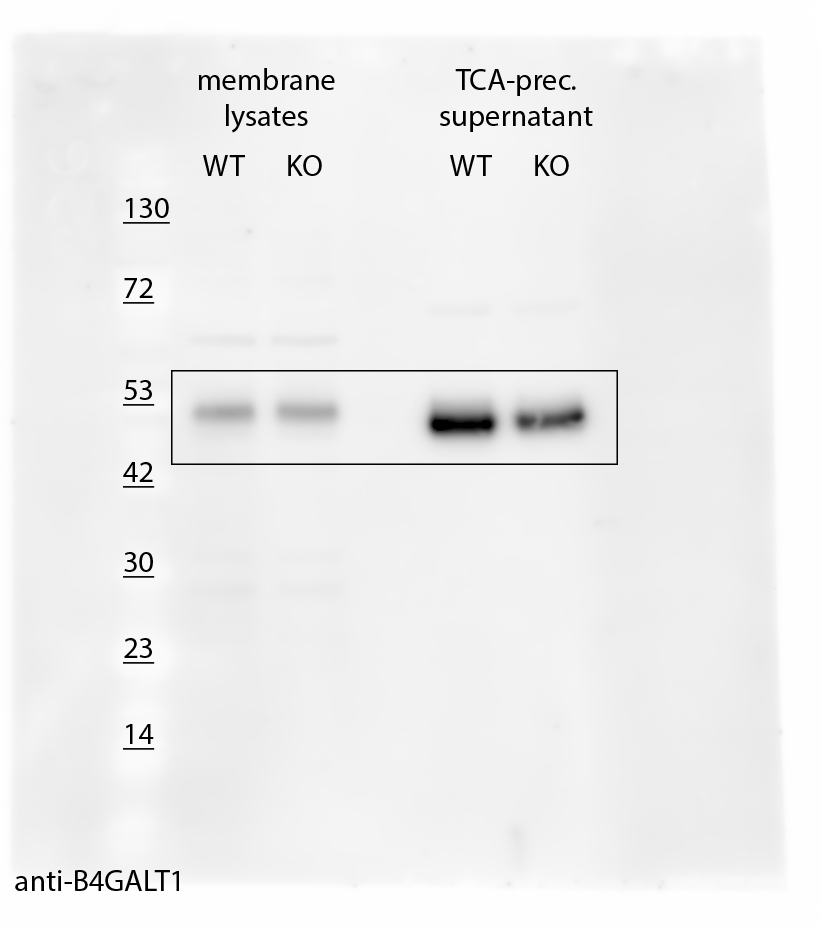

Supplement: Supplementary file 4 — Source data Fig. 1 [file 44318_2024_305_MOESM4_ESM.zip › Figure 1/1B/source data B4GALT1 time series 20230516_115719-04_Ch_Chemi.tif]

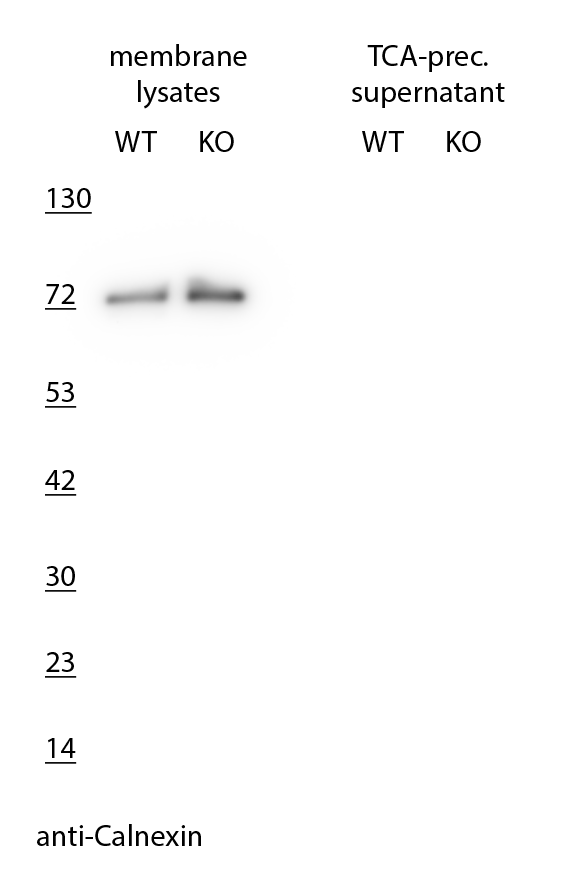

Supplement: Supplementary file 4 — Source data Fig. 1 [file 44318_2024_305_MOESM4_ESM.zip › Figure 1/1B/source data Calnexin for B4GALT5 (6D4), MGAT5, B4GAT1 20240227_161507-01_Ch_Chemi.tif]

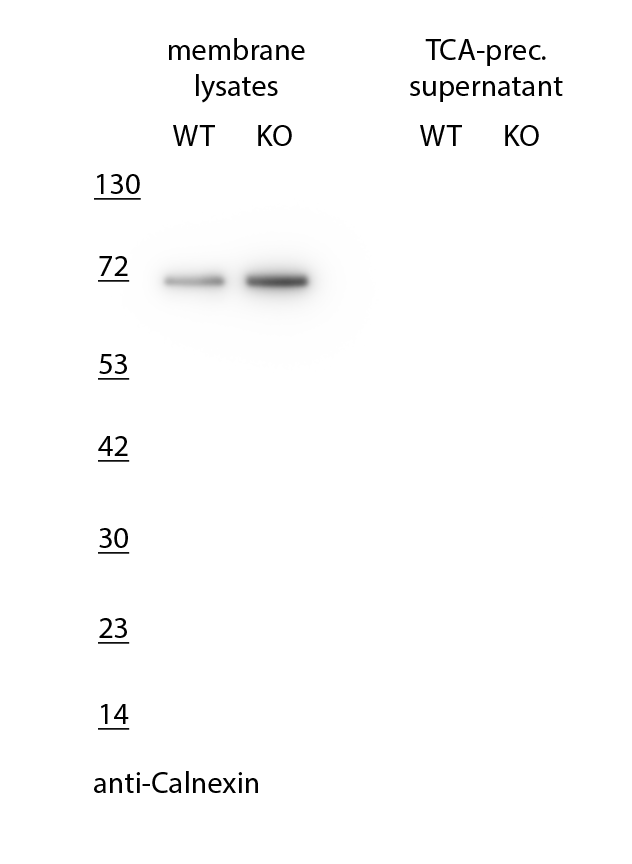

Supplement: Supplementary file 4 — Source data Fig. 1 [file 44318_2024_305_MOESM4_ESM.zip › Figure 1/1B/source data Calnexin for B4GALT1 20240227_162354-01_Ch_Chemi.tif]

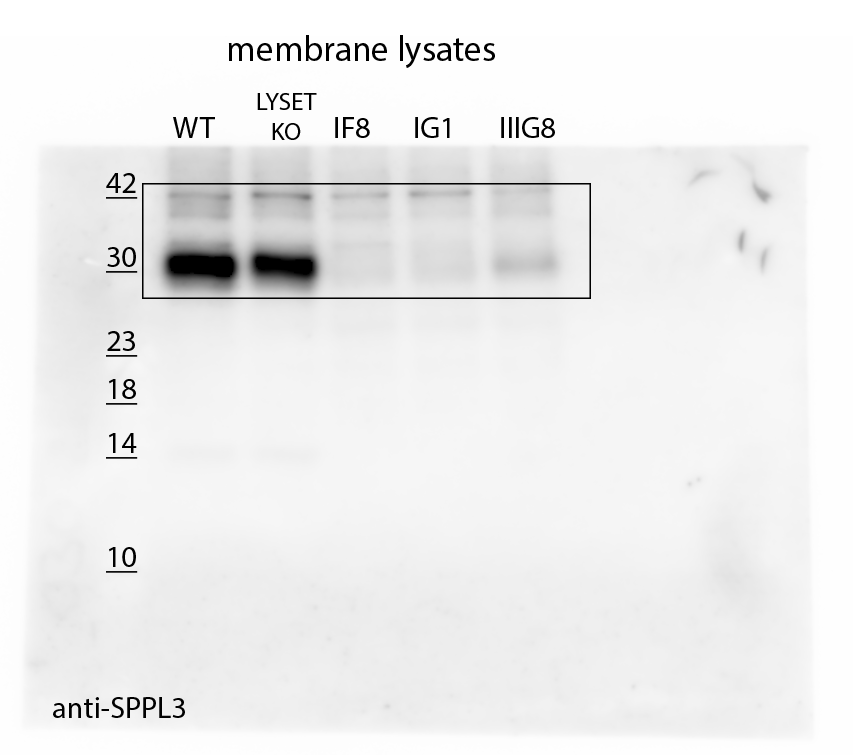

Supplement: Supplementary file 4 — Source data Fig. 1 [file 44318_2024_305_MOESM4_ESM.zip › Figure 1/1E/source data SPPL3 time series 20230601_125458-06_Ch_Chemi.tif]

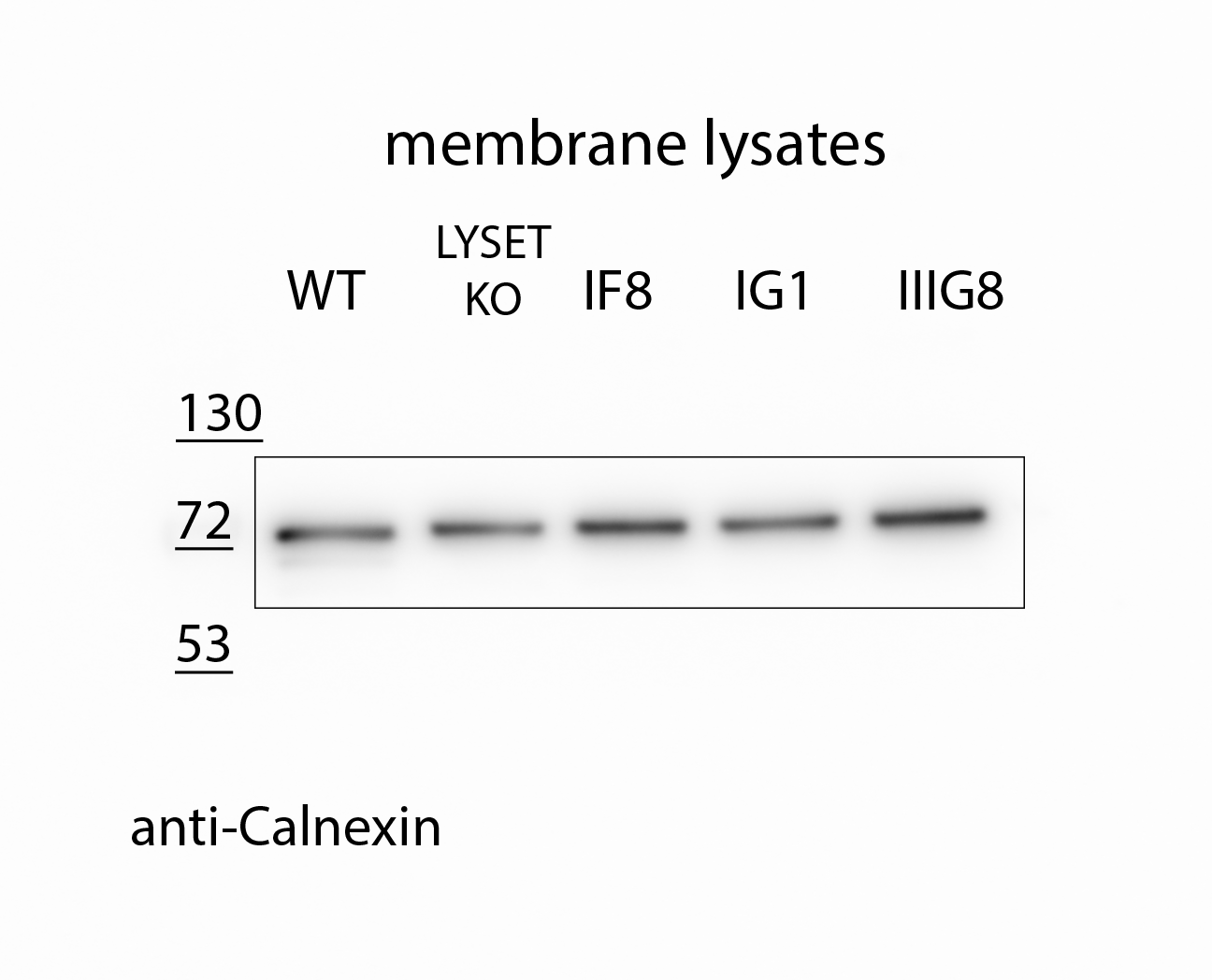

Supplement: Supplementary file 4 — Source data Fig. 1 [file 44318_2024_305_MOESM4_ESM.zip › Figure 1/1E/source data Calnexin time series 20230530_124938-12_Ch_Chemi.tif]

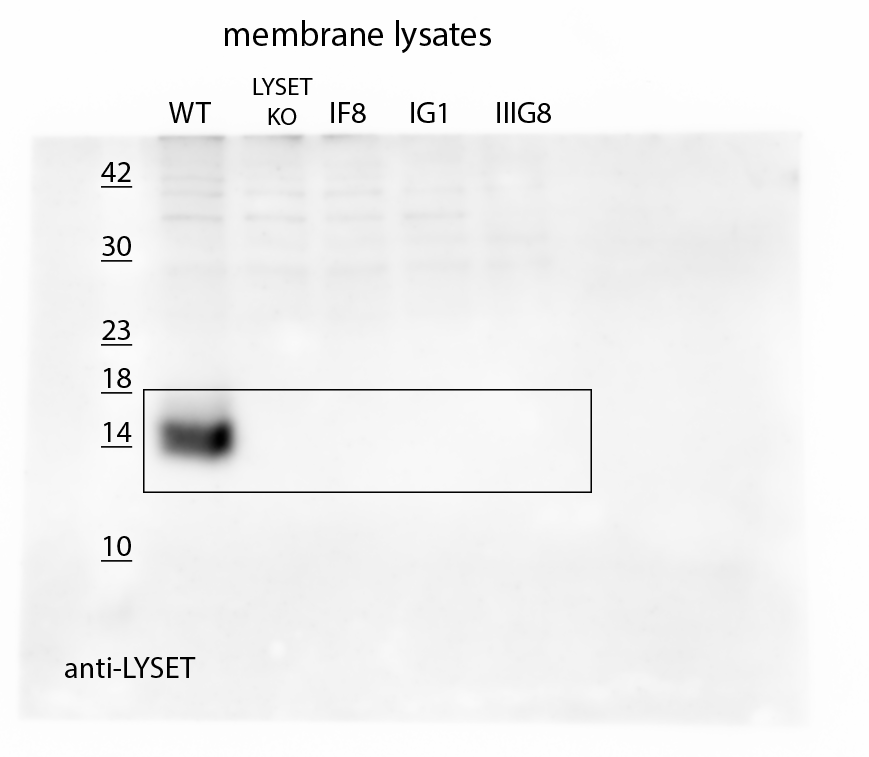

Supplement: Supplementary file 4 — Source data Fig. 1 [file 44318_2024_305_MOESM4_ESM.zip › Figure 1/1E/source data LYSET time series 20230530_123645-09_Ch_Chemi.tif]

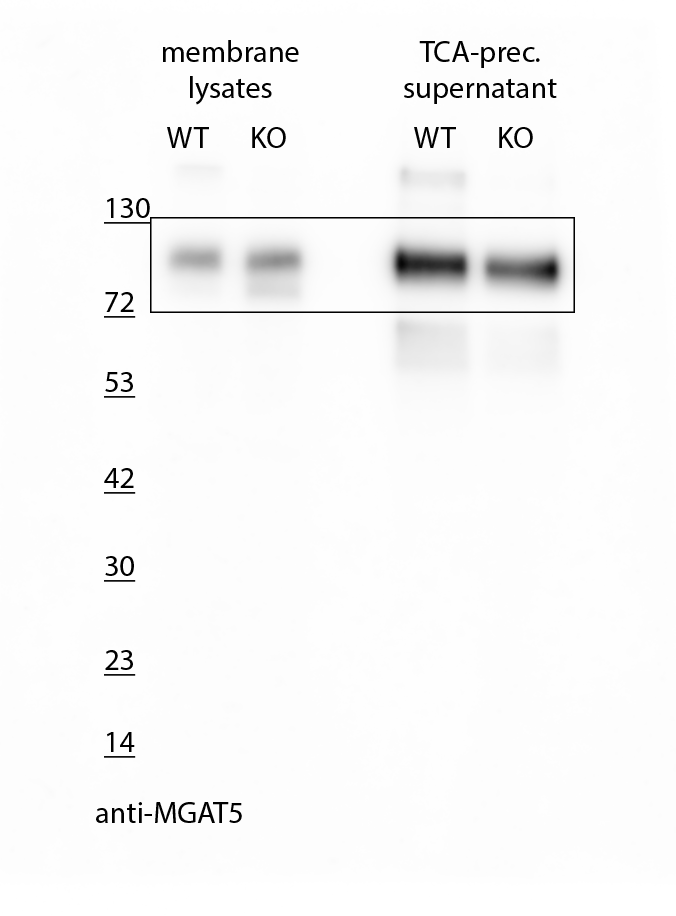

Supplement: Supplementary file 4 — Source data Fig. 1 [file 44318_2024_305_MOESM4_ESM.zip › Figure 1/1C/source data MGAT5 time series 20230817_131850-03_Ch_Chemi.tif]

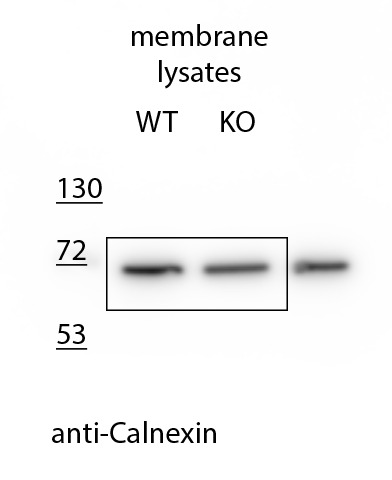

Supplement: Supplementary file 4 — Source data Fig. 1 [file 44318_2024_305_MOESM4_ESM.zip › Figure 1/1C/source data Calnexin for LYSET time series 20230807_152722-01_Ch_Chemi.tif]

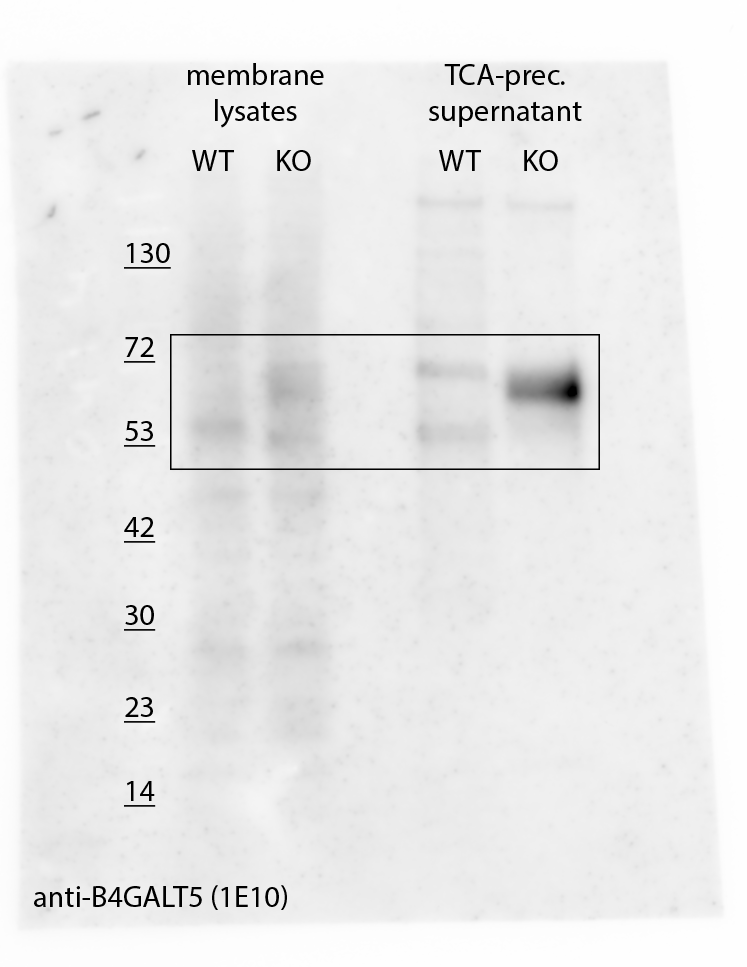

Supplement: Supplementary file 4 — Source data Fig. 1 [file 44318_2024_305_MOESM4_ESM.zip › Figure 1/1C/source data B4GALT5 (1E10) time series 20230816_140058-20_Ch_Chemi.tif]

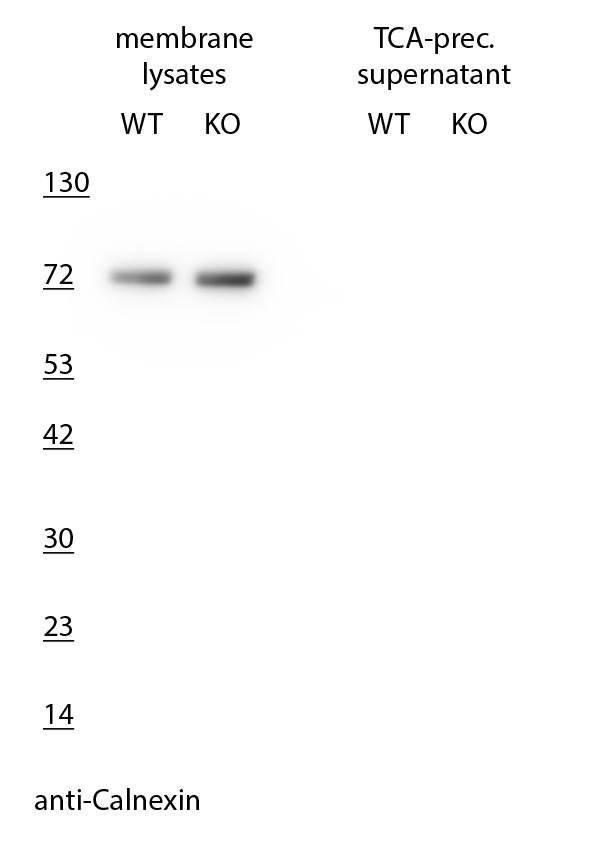

Supplement: Supplementary file 4 — Source data Fig. 1 [file 44318_2024_305_MOESM4_ESM.zip › Figure 1/1C/source data Calnexin for GALNT2, EXTL3 20240229_140920-02_Ch_Chemi.tif]

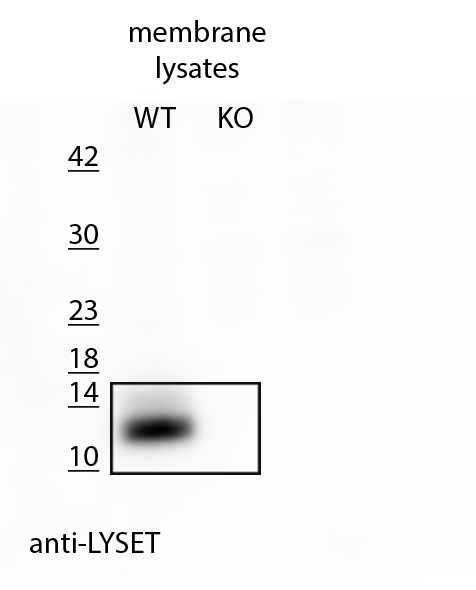

Supplement: Supplementary file 4 — Source data Fig. 1 [file 44318_2024_305_MOESM4_ESM.zip › Figure 1/1C/source data LYSET time series 20230807_153236-01_Ch_Chemi.tif]

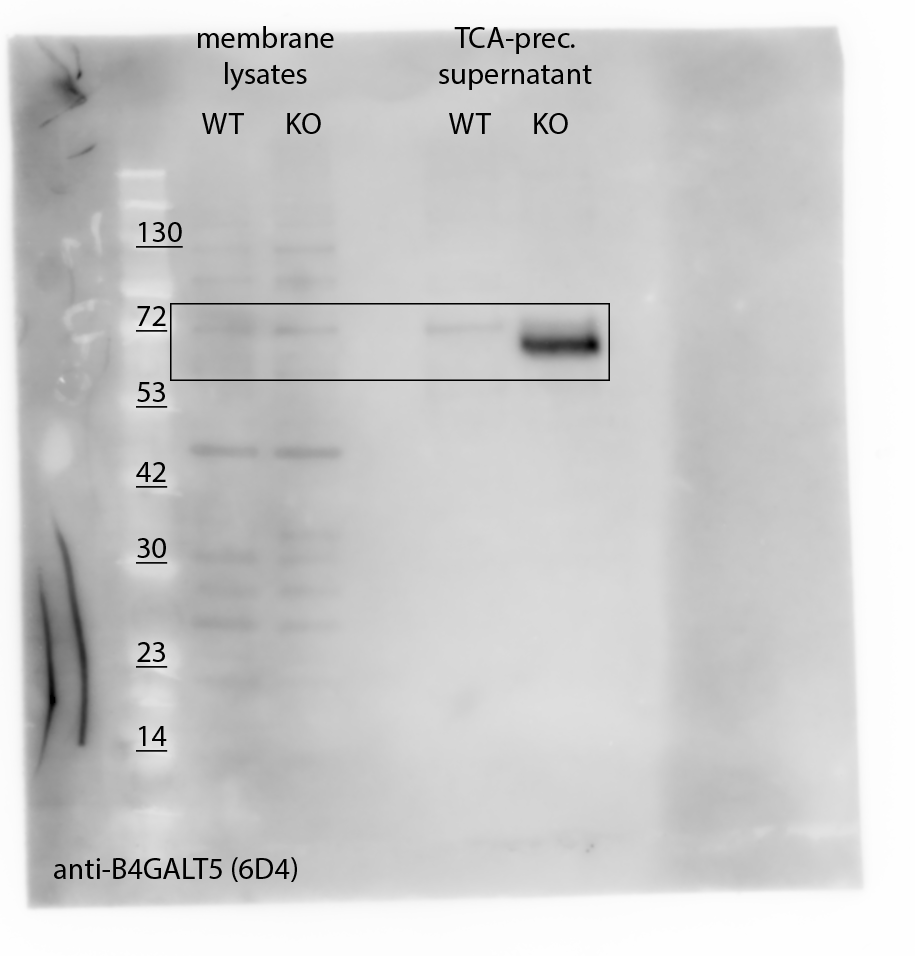

Supplement: Supplementary file 4 — Source data Fig. 1 [file 44318_2024_305_MOESM4_ESM.zip › Figure 1/1C/source data B4GALT5 (6D4) time series 20230815_141554-05_Ch_Chemi.tif]

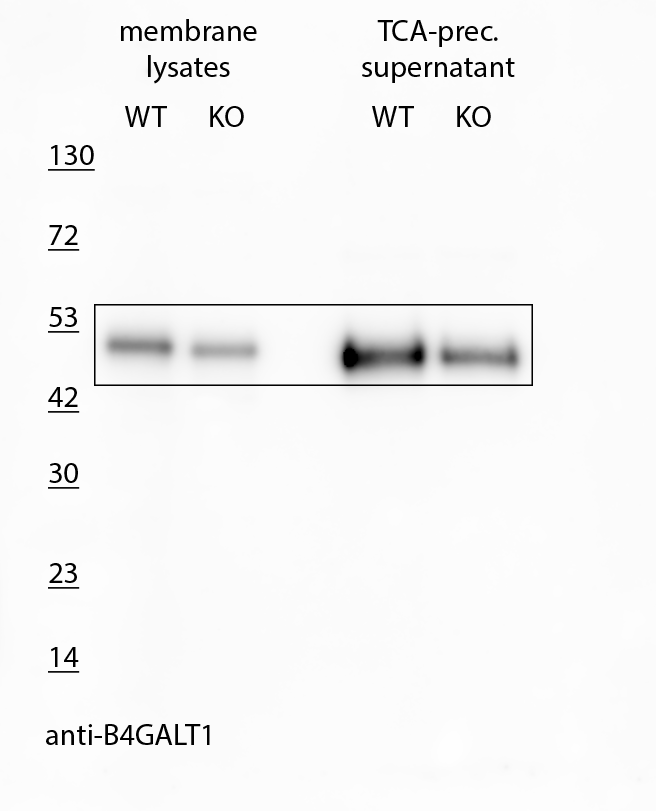

Supplement: Supplementary file 4 — Source data Fig. 1 [file 44318_2024_305_MOESM4_ESM.zip › Figure 1/1C/source data B4GALT1 time series 20230816_135538-02_Ch_Chemi.tif]

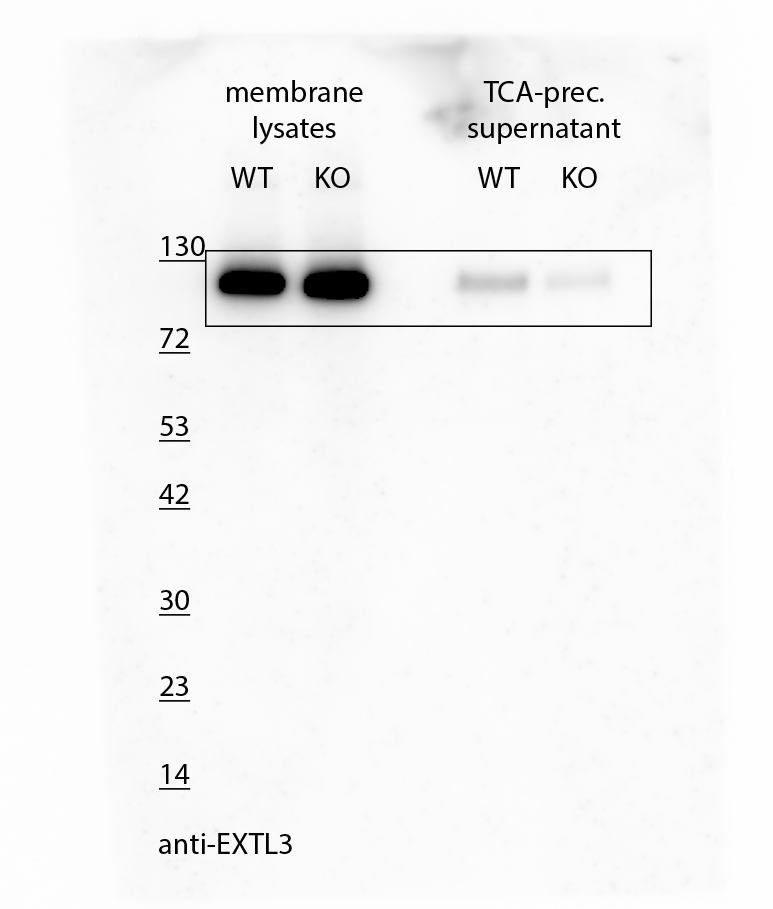

Supplement: Supplementary file 4 — Source data Fig. 1 [file 44318_2024_305_MOESM4_ESM.zip › Figure 1/1C/source data EXTL3 time series 20230817_115604-20_Ch_Chemi.tif]

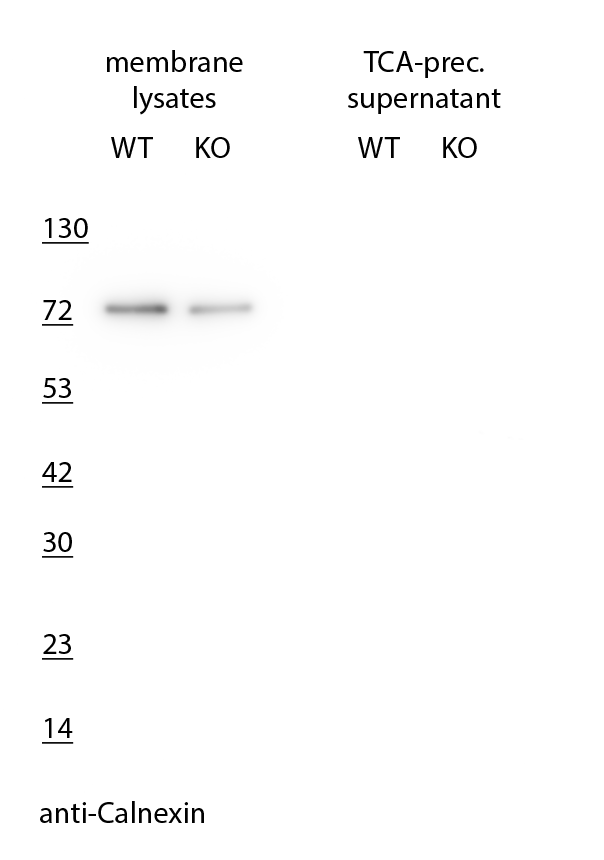

Supplement: Supplementary file 4 — Source data Fig. 1 [file 44318_2024_305_MOESM4_ESM.zip › Figure 1/1C/source data Calnexin for B4GALT5 (6D4), B4GALT1 20240229_140351-02_Ch_Chemi.tif]

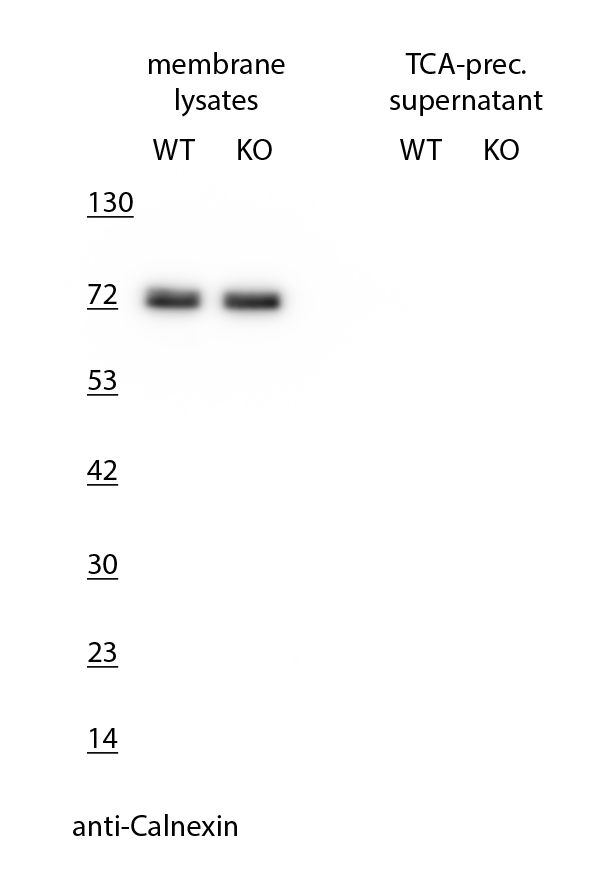

Supplement: Supplementary file 4 — Source data Fig. 1 [file 44318_2024_305_MOESM4_ESM.zip › Figure 1/1C/source data Calnexin for B4GALT5 (1E10), MGAT5 20240229_144921-03_Ch_Chemi.tif]

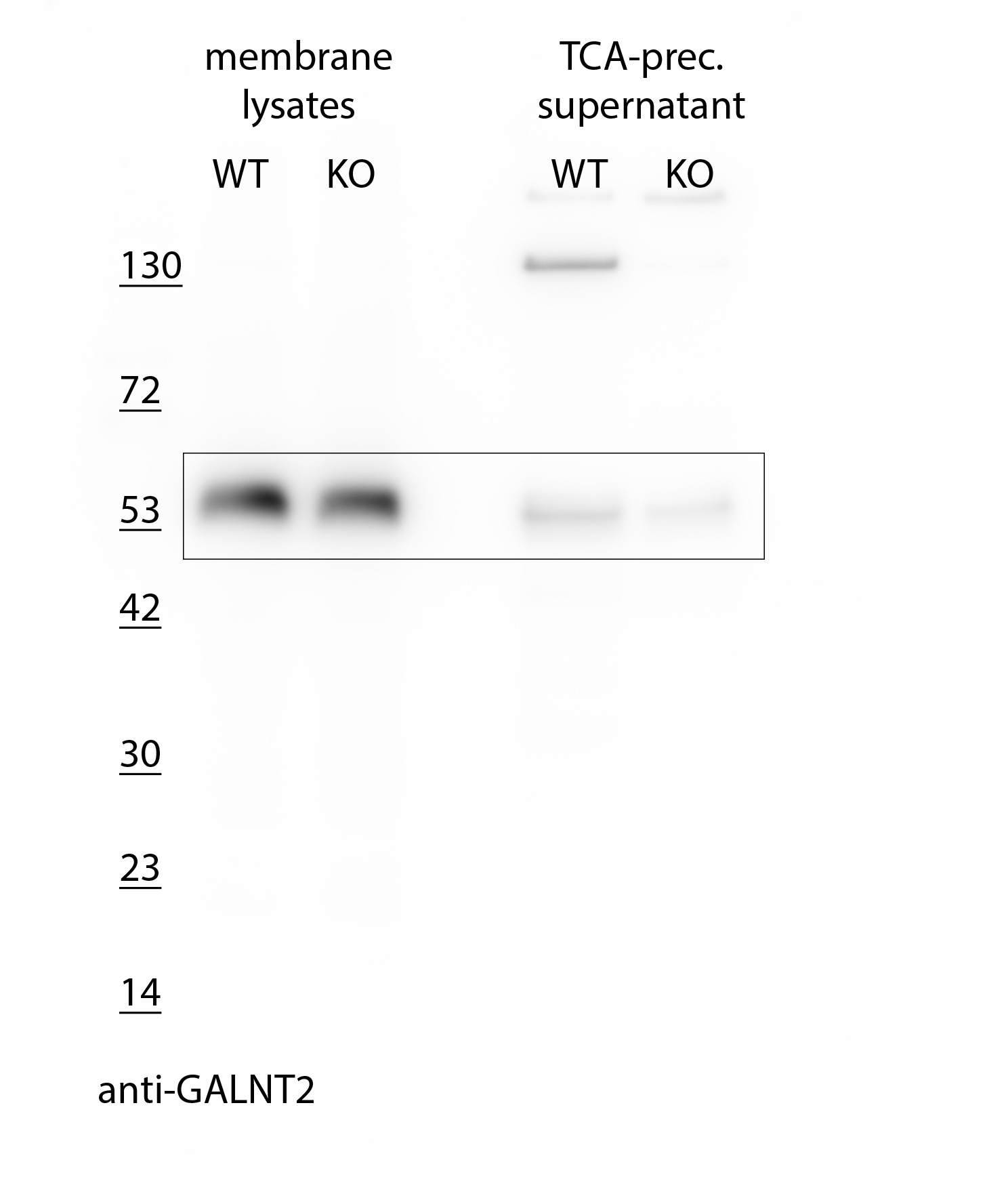

Supplement: Supplementary file 4 — Source data Fig. 1 [file 44318_2024_305_MOESM4_ESM.zip › Figure 1/1C/source data GALNT2 time series 20230816_141322-01_Ch_Chemi.tif]

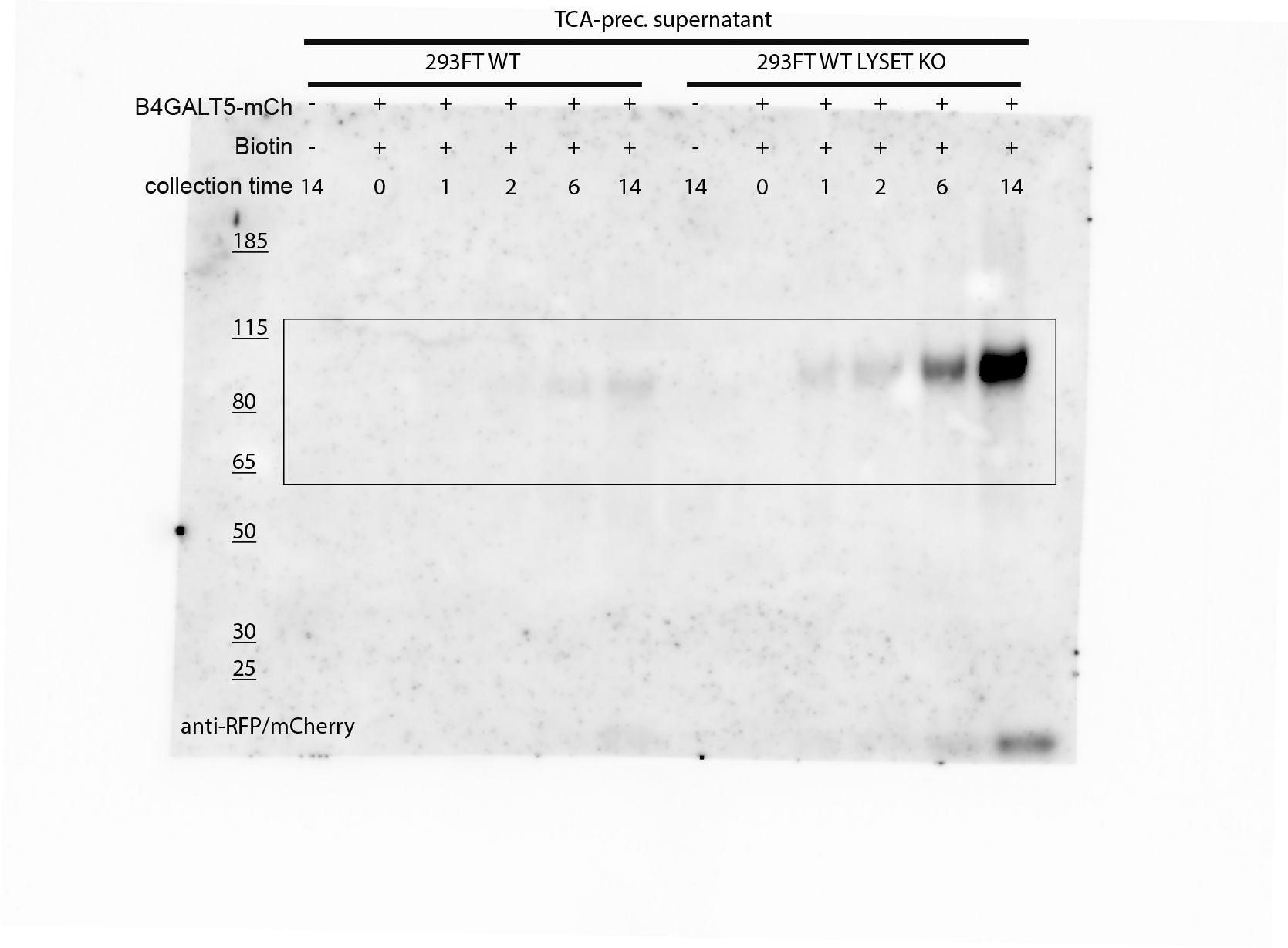

Supplement: Supplementary file 5 — Source data Fig. 2 [file 44318_2024_305_MOESM5_ESM.zip › Figure 2/2G/source data RFP supernatant time series 20240906_134251-42_Ch_Chemi.tif]

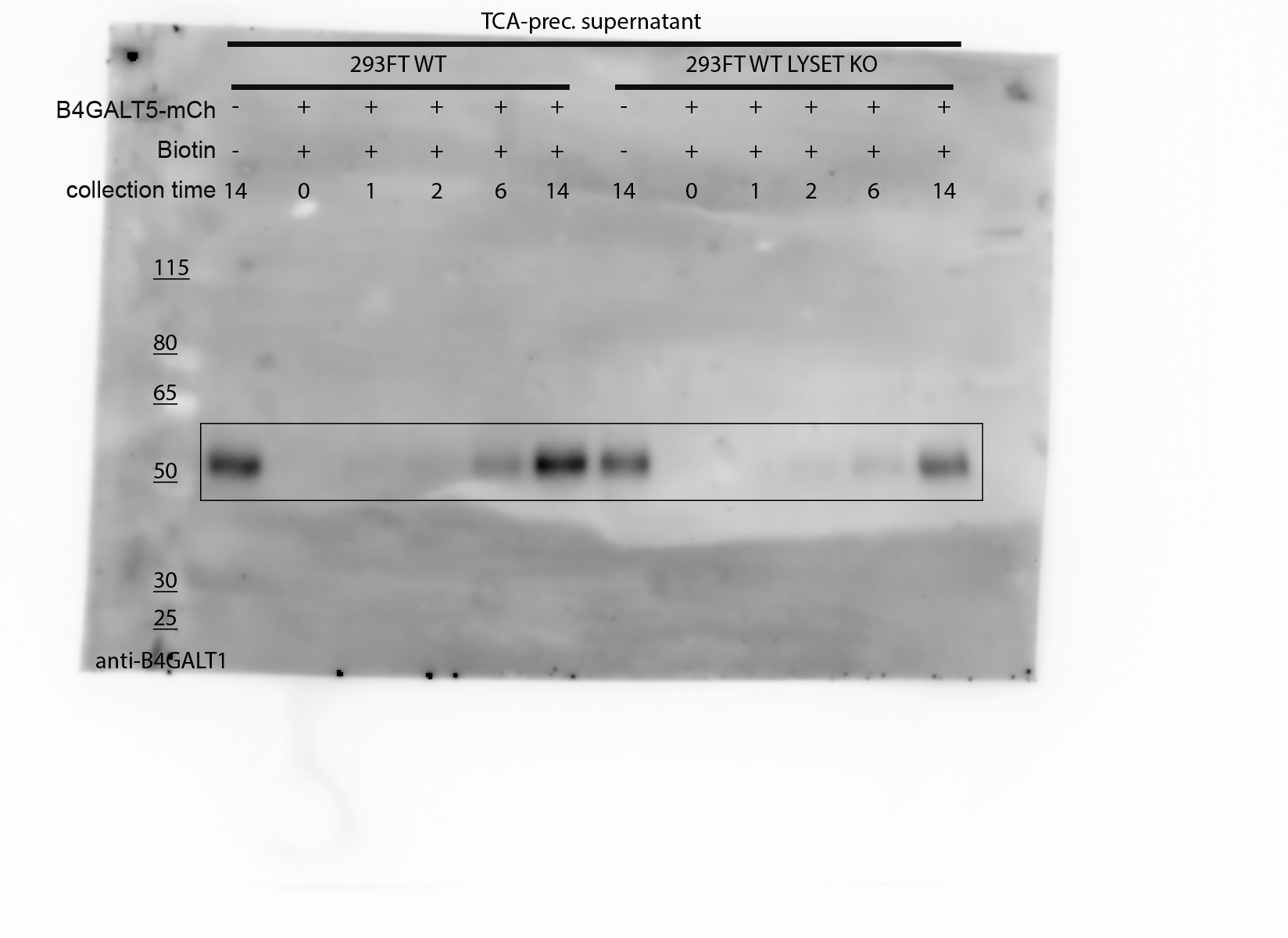

Supplement: Supplementary file 5 — Source data Fig. 2 [file 44318_2024_305_MOESM5_ESM.zip › Figure 2/2G/source data B4GALT1 supernatant time series 20240912_115106-22_Ch_Chemi.tif]

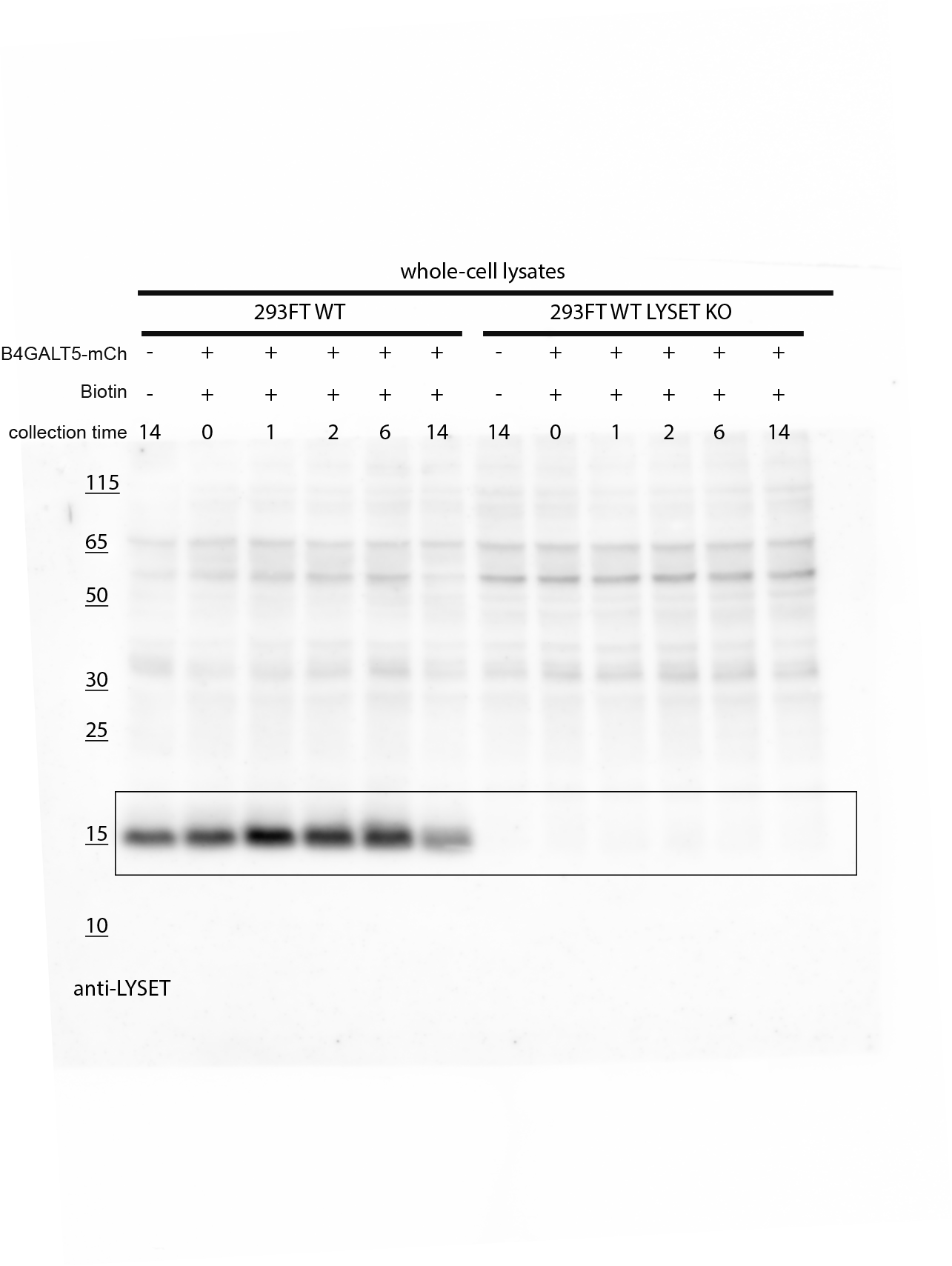

Supplement: Supplementary file 5 — Source data Fig. 2 [file 44318_2024_305_MOESM5_ESM.zip › Figure 2/2G/source data LYSET time series 20240906_105949-15_Ch_Chemi.tif]

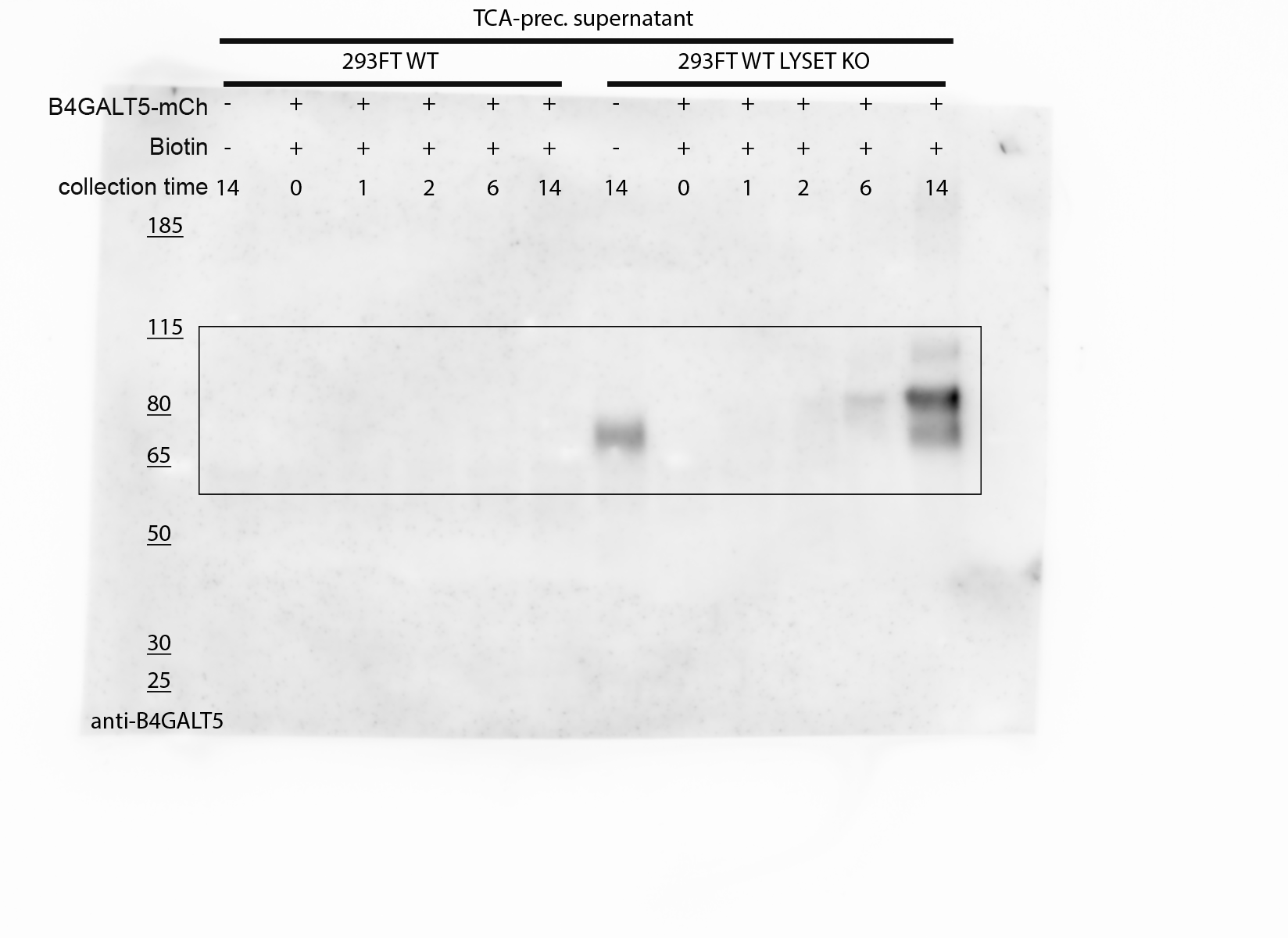

Supplement: Supplementary file 5 — Source data Fig. 2 [file 44318_2024_305_MOESM5_ESM.zip › Figure 2/2G/source data B4GALT5 supernatant time series 20240908_123112-14_Ch_Chemi.tif]

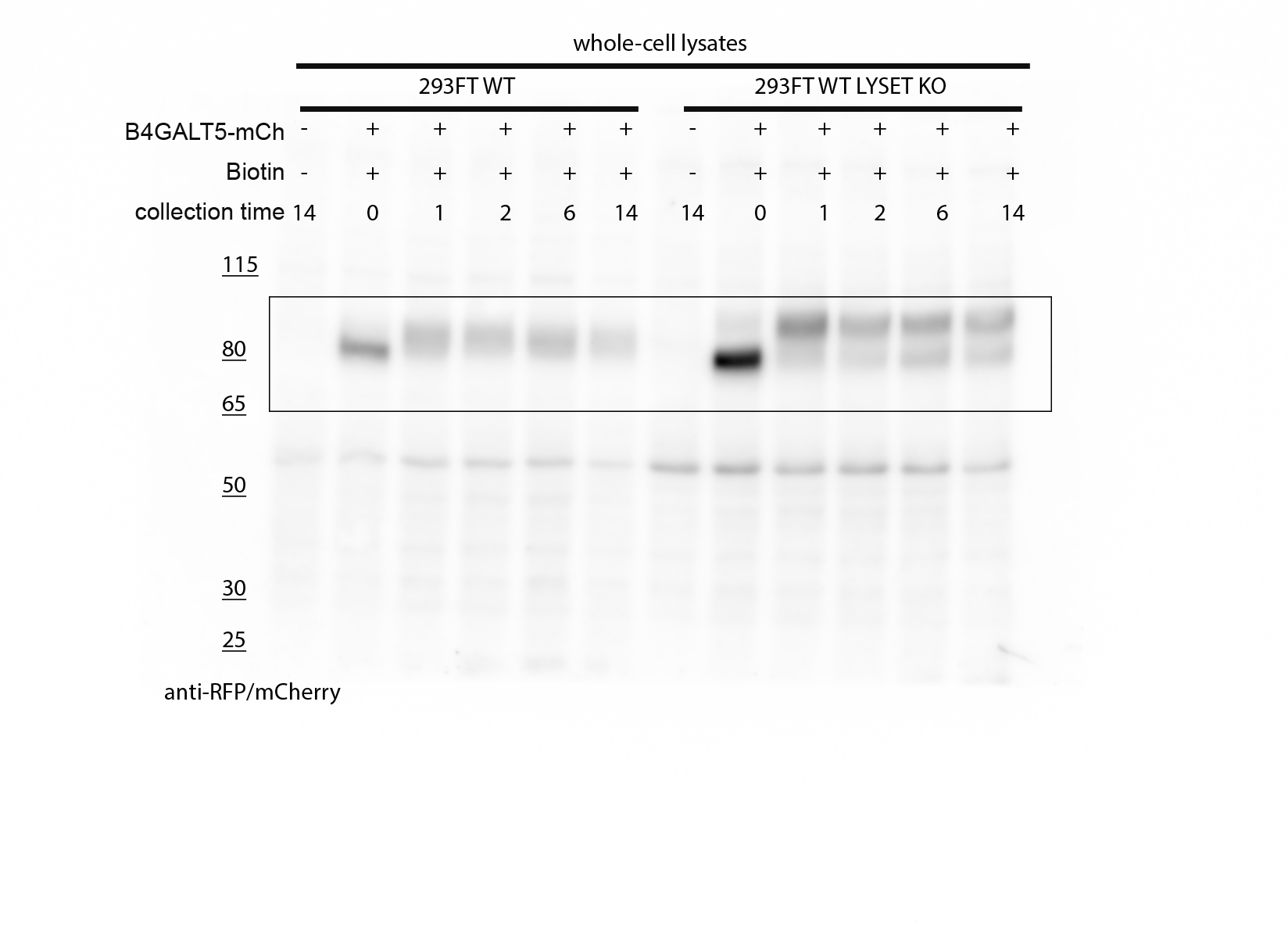

Supplement: Supplementary file 5 — Source data Fig. 2 [file 44318_2024_305_MOESM5_ESM.zip › Figure 2/2G/source data RFP mCherry cell lysates time series 20240830 20240830_114743-07_Ch_Chemi.tif]

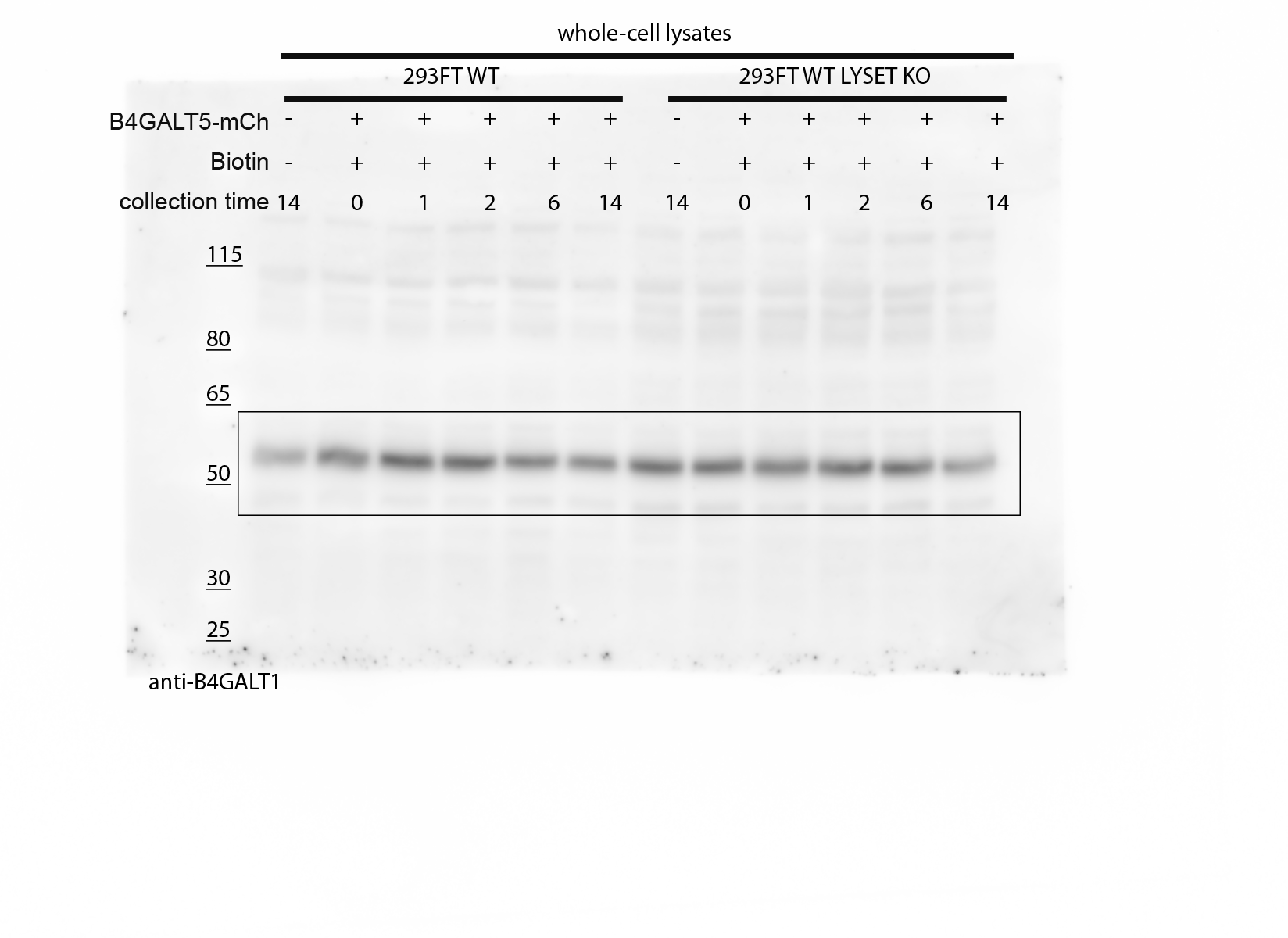

Supplement: Supplementary file 5 — Source data Fig. 2 [file 44318_2024_305_MOESM5_ESM.zip › Figure 2/2G/source data B4GALT1 cell lysates time series 20240902_132305-06_Ch_Chemi.tif]

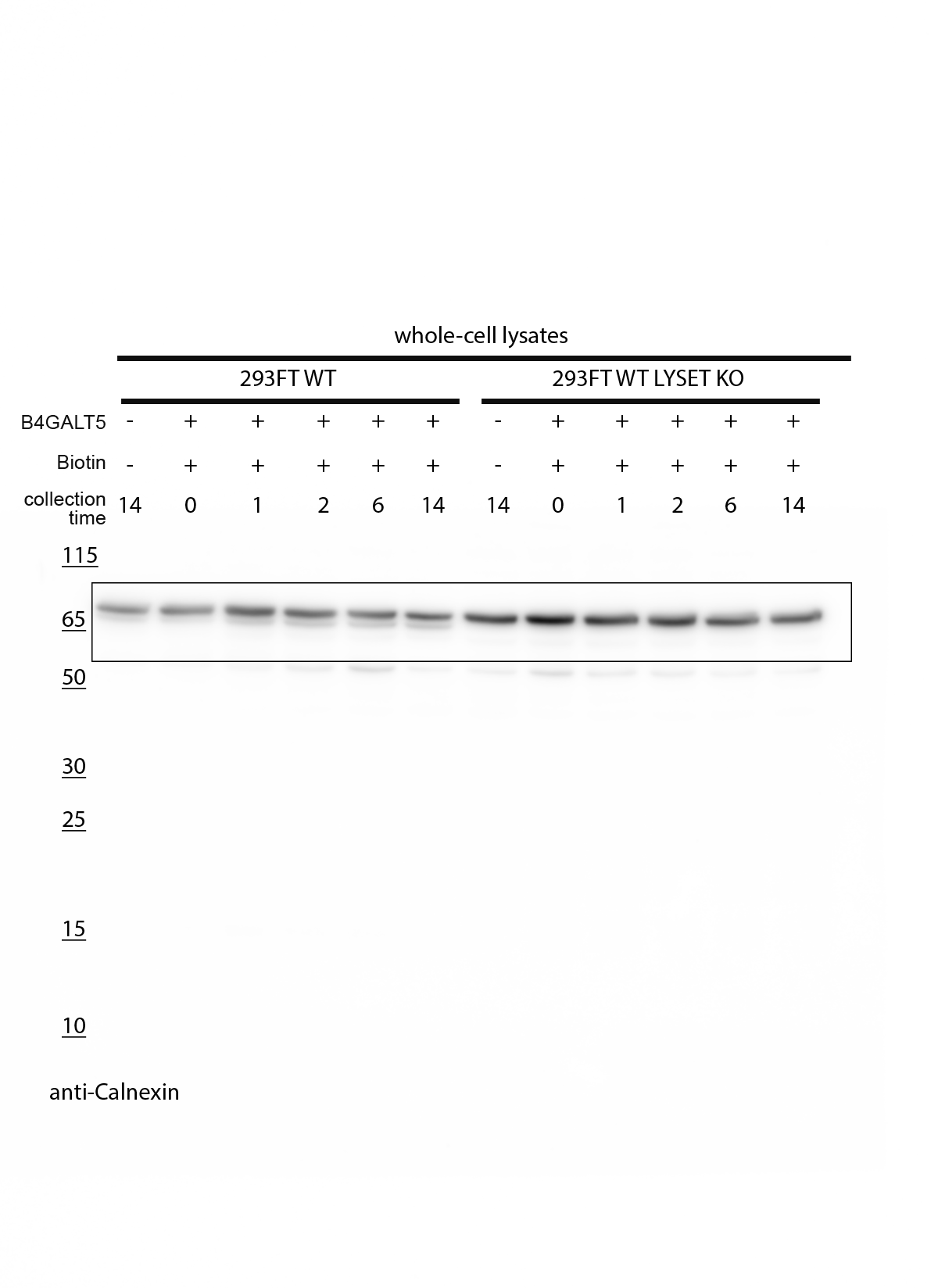

Supplement: Supplementary file 5 — Source data Fig. 2 [file 44318_2024_305_MOESM5_ESM.zip › Figure 2/2G/source data Calnexin time series 20240912_110843-02_Ch_Chemi.tif]

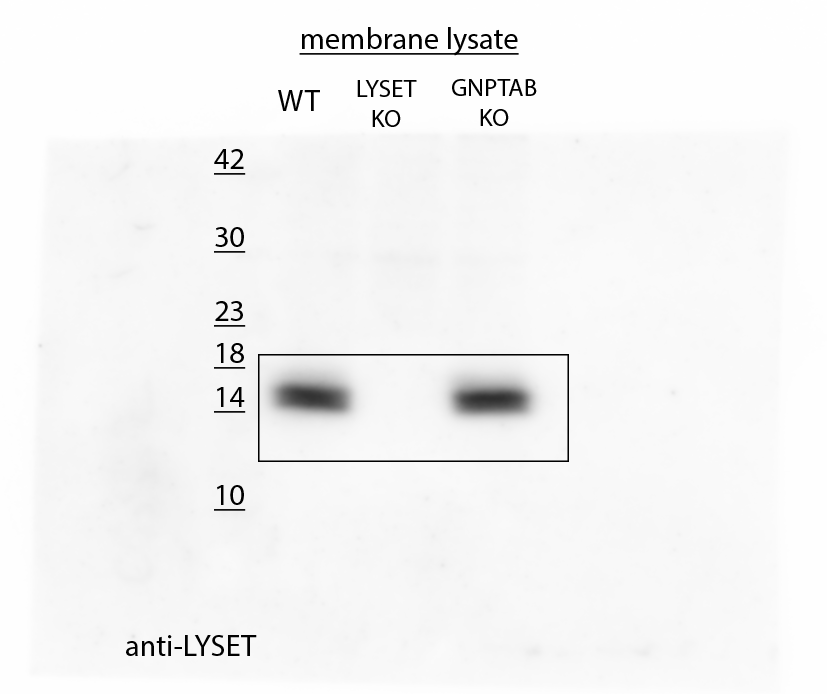

Supplement: Supplementary file 5 — Source data Fig. 2 [file 44318_2024_305_MOESM5_ESM.zip › Figure 2/2A/source data LYSET time series 20230629_151744-10_Ch_Chemi.tif]

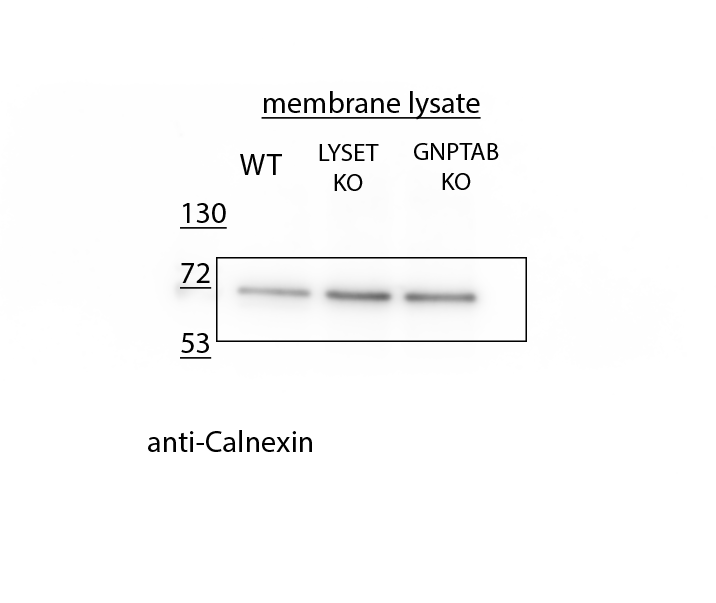

Supplement: Supplementary file 5 — Source data Fig. 2 [file 44318_2024_305_MOESM5_ESM.zip › Figure 2/2A/source data Calnexin for LYSET time series 20230629_152809-01_Ch_Chemi.tif]

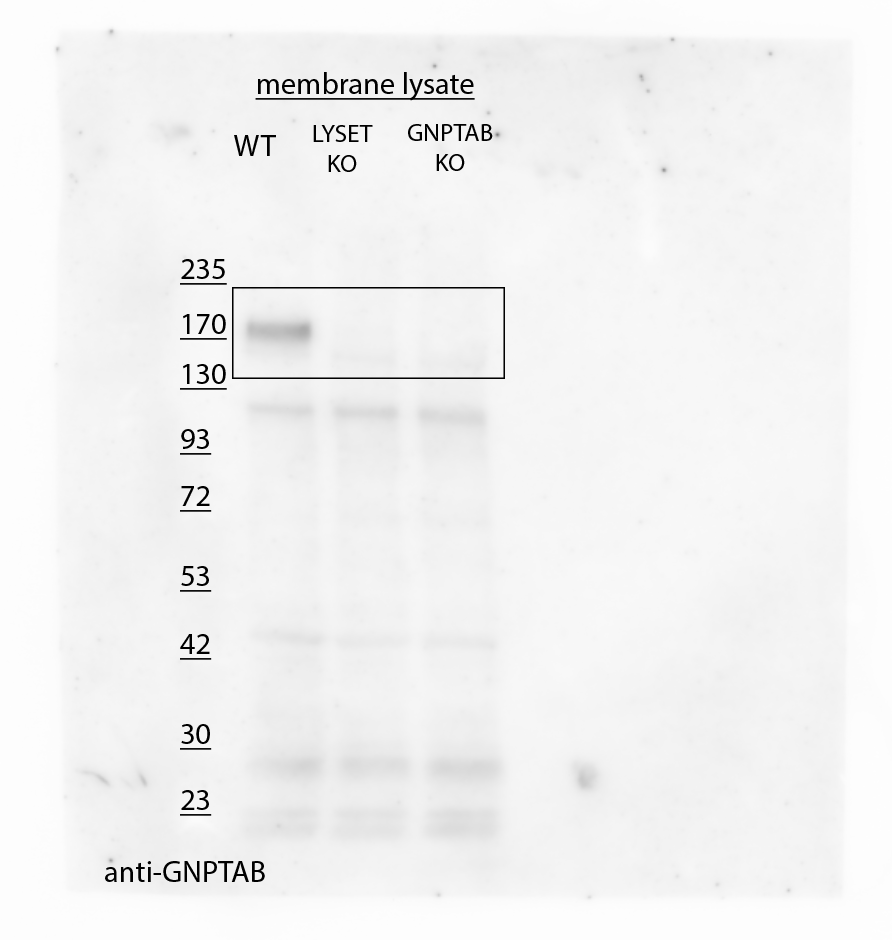

Supplement: Supplementary file 5 — Source data Fig. 2 [file 44318_2024_305_MOESM5_ESM.zip › Figure 2/2A/source data GNPTAB time series 20230807_153730-20_Ch_Chemi.tif]

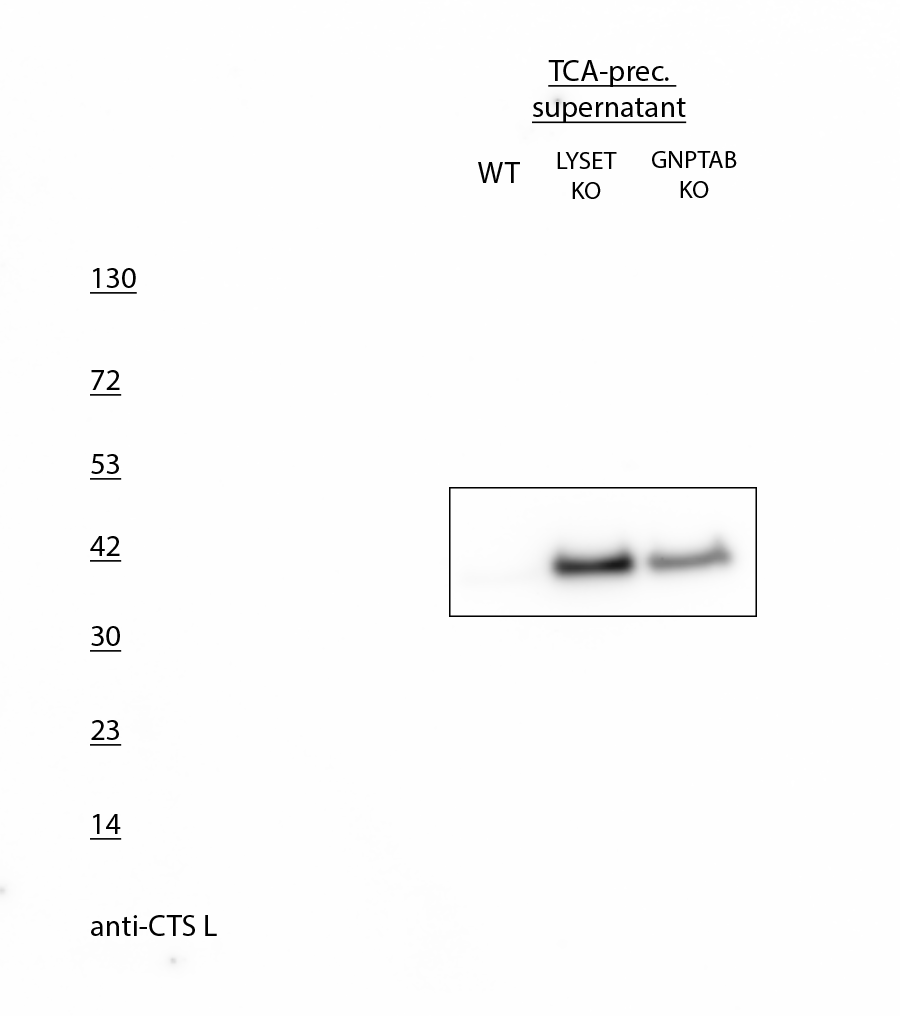

Supplement: Supplementary file 5 — Source data Fig. 2 [file 44318_2024_305_MOESM5_ESM.zip › Figure 2/2A/souroce data CTS L time series 20230705_145043-18_Ch_Chemi.tif]

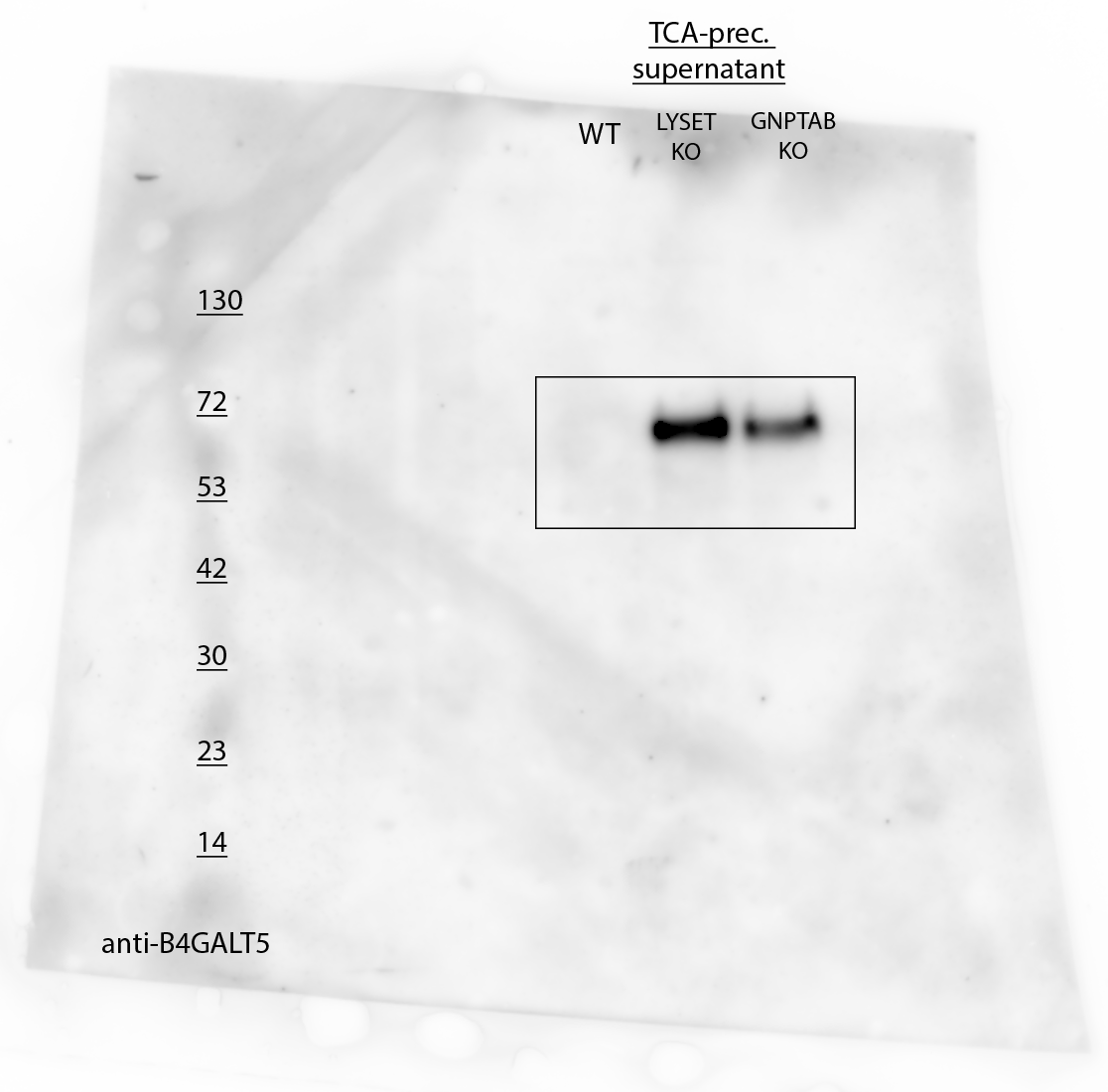

Supplement: Supplementary file 5 — Source data Fig. 2 [file 44318_2024_305_MOESM5_ESM.zip › Figure 2/2A/source data B4GALT5 time series 20230629_145105-06_Ch_Chemi.tif]

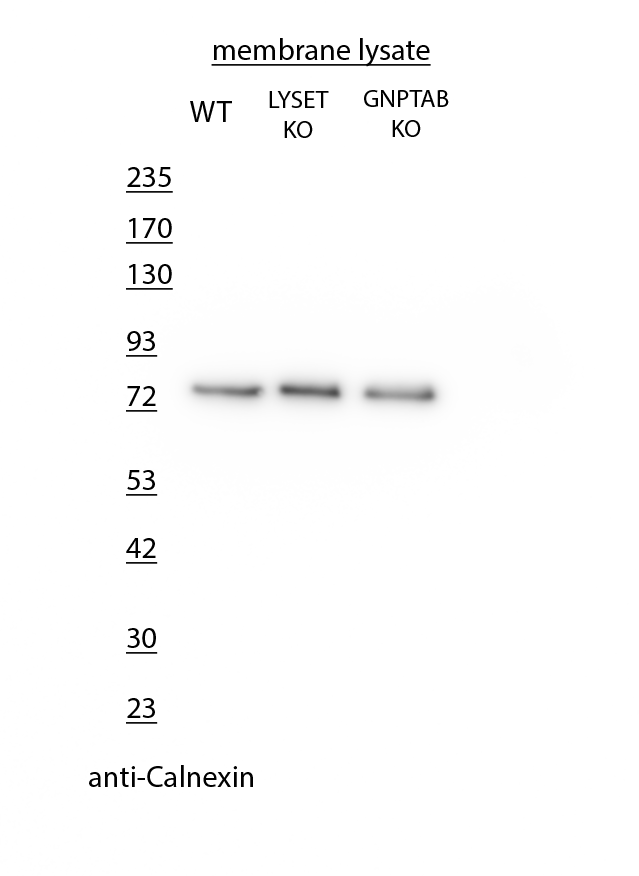

Supplement: Supplementary file 5 — Source data Fig. 2 [file 44318_2024_305_MOESM5_ESM.zip › Figure 2/2A/source data Calnexin for GNPTAB 20240229_143600-03_Ch_Chemi.tif]

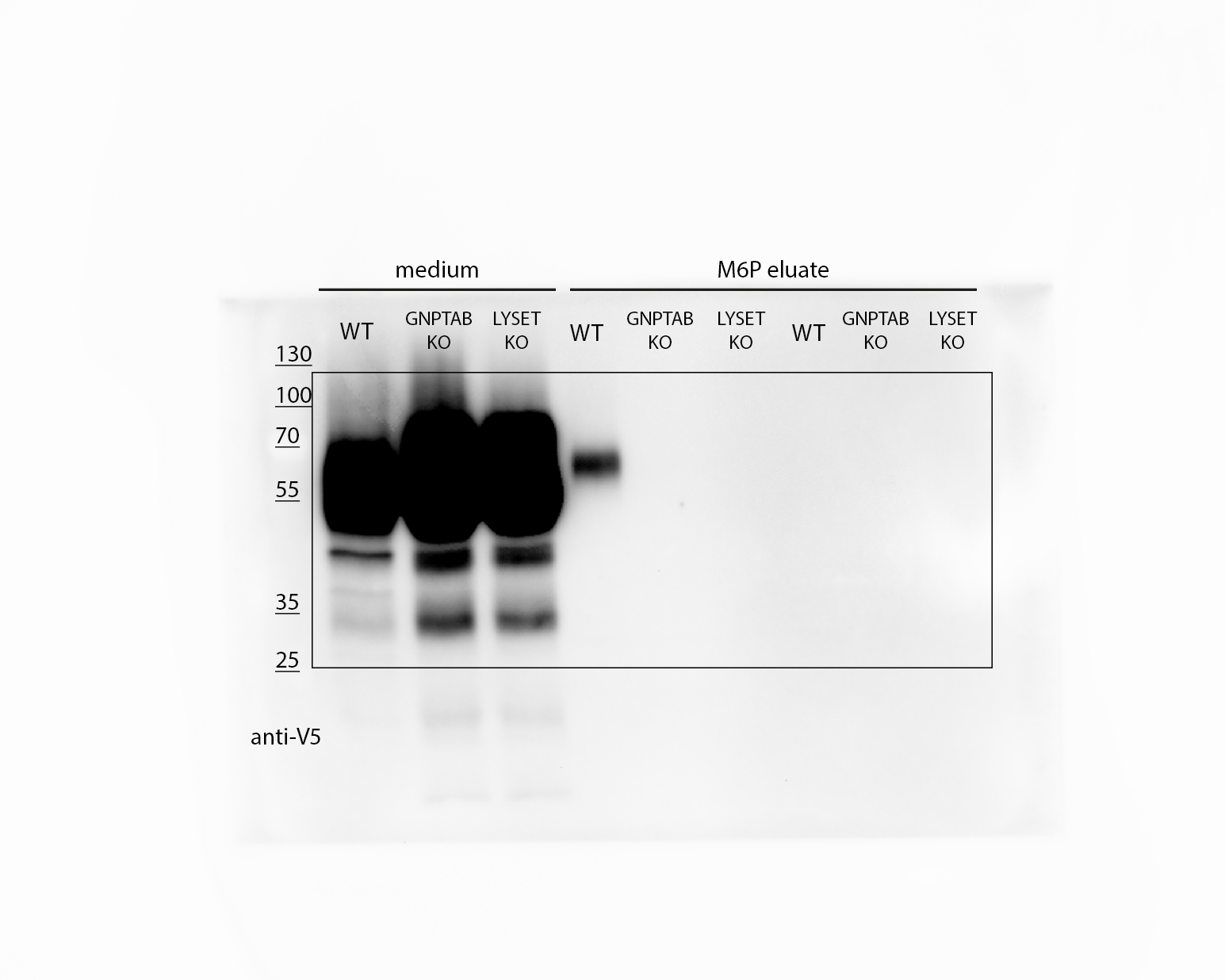

Supplement: Supplementary file 5 — Source data Fig. 2 [file 44318_2024_305_MOESM5_ESM.zip › Figure 2/2F/source data V5 (M6P eluate).tif]

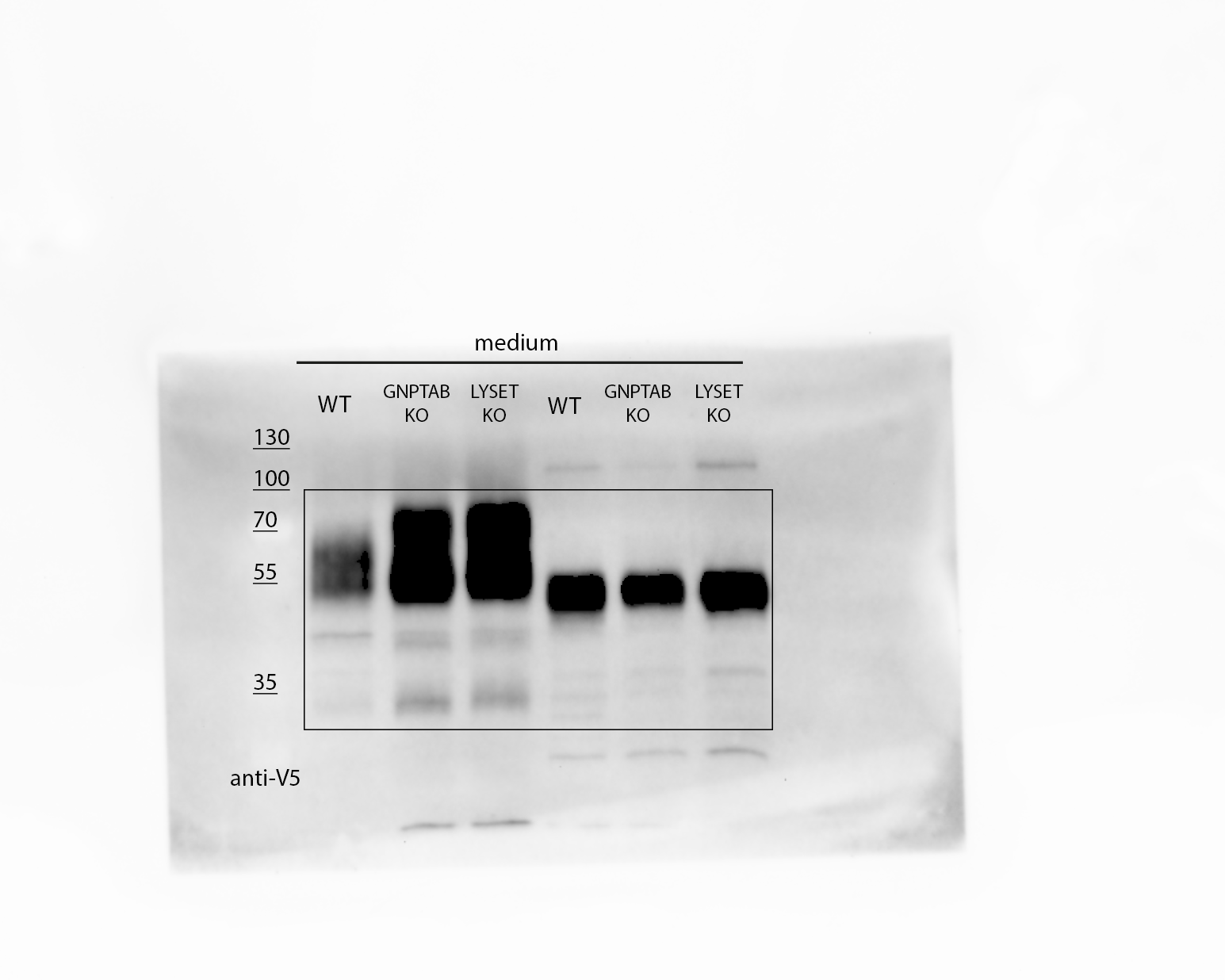

Supplement: Supplementary file 5 — Source data Fig. 2 [file 44318_2024_305_MOESM5_ESM.zip › Figure 2/2F/source data V5 (medium).tif]

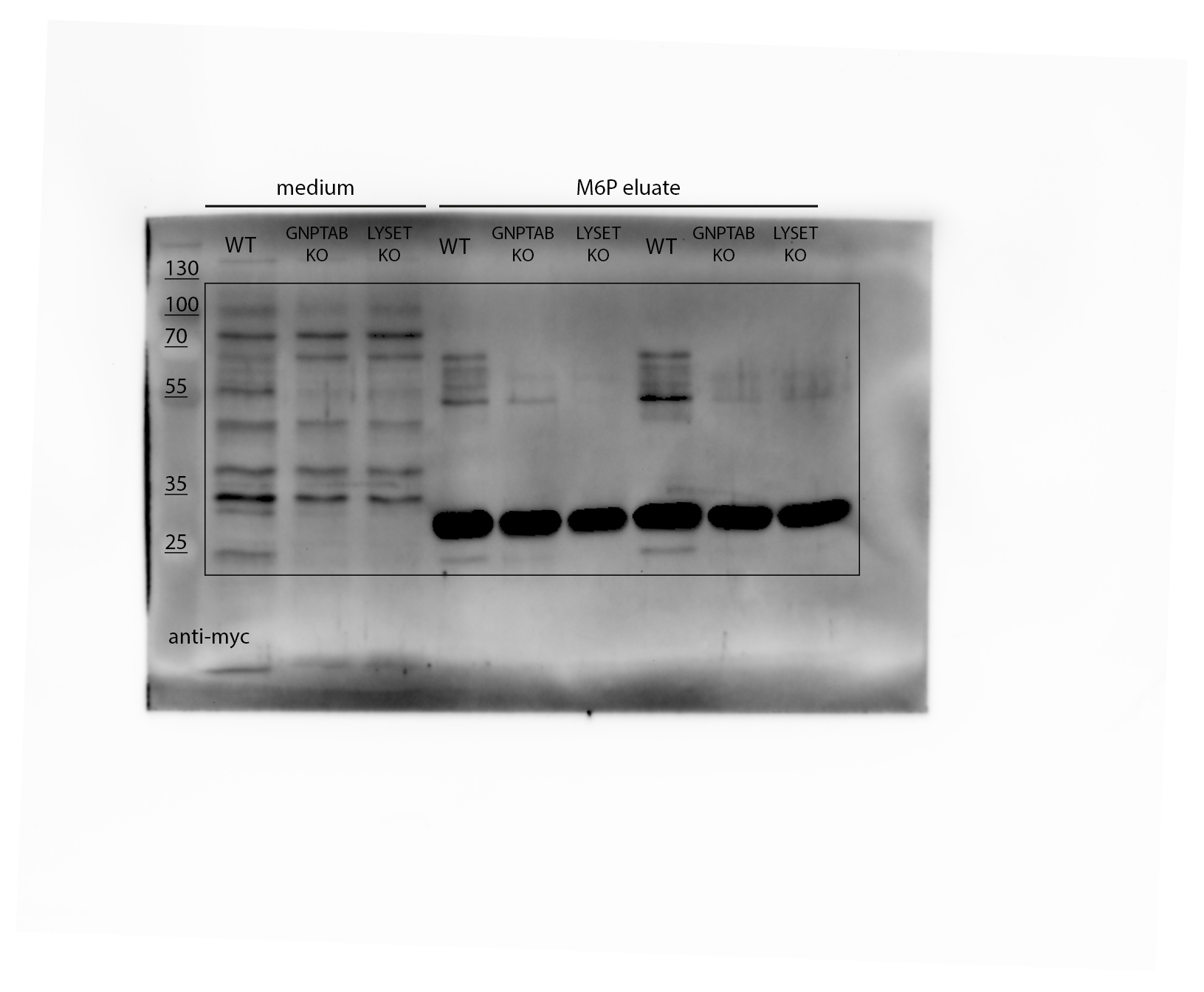

Supplement: Supplementary file 5 — Source data Fig. 2 [file 44318_2024_305_MOESM5_ESM.zip › Figure 2/2F/source data myc.tif]

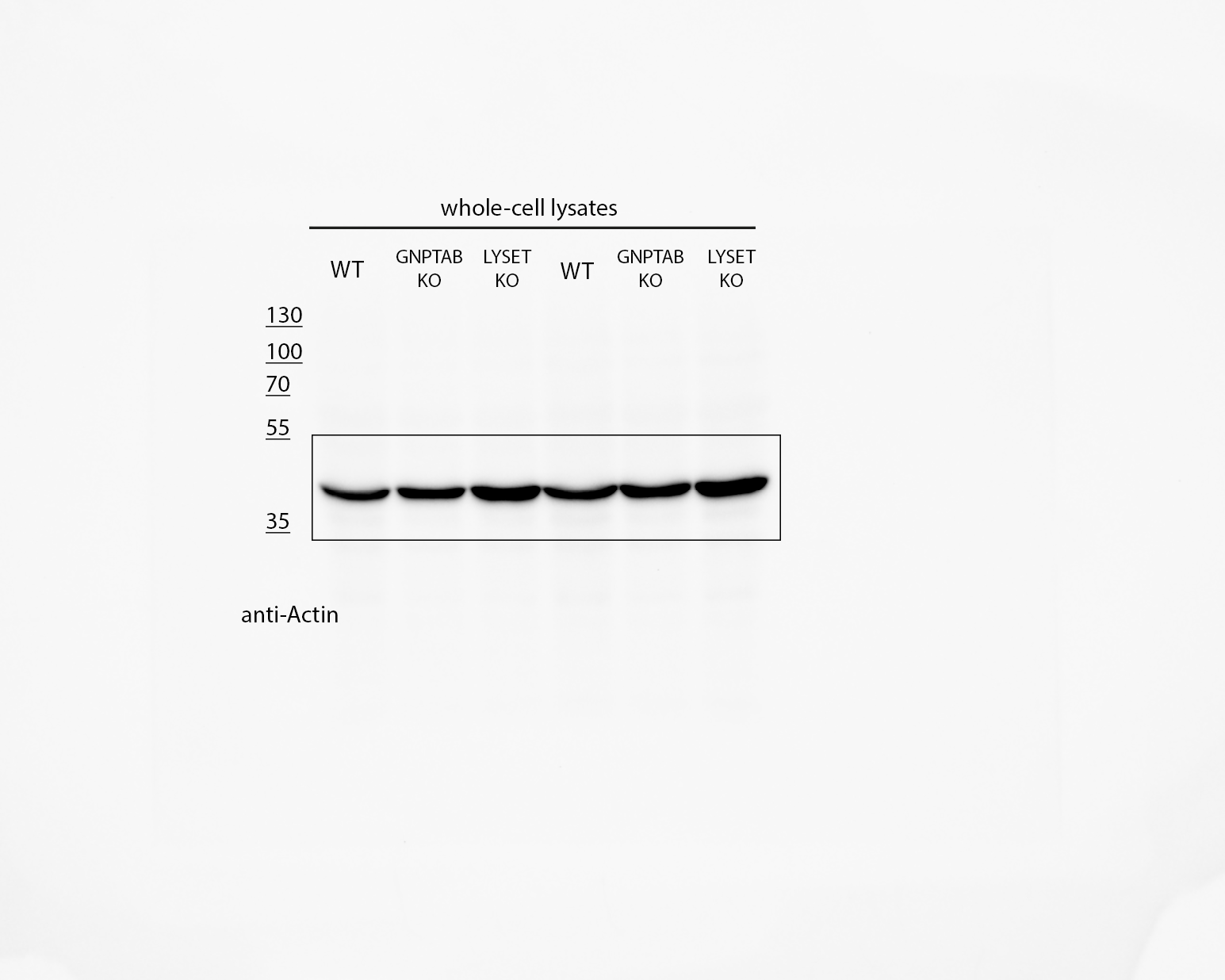

Supplement: Supplementary file 5 — Source data Fig. 2 [file 44318_2024_305_MOESM5_ESM.zip › Figure 2/2F/source data actin.tif]

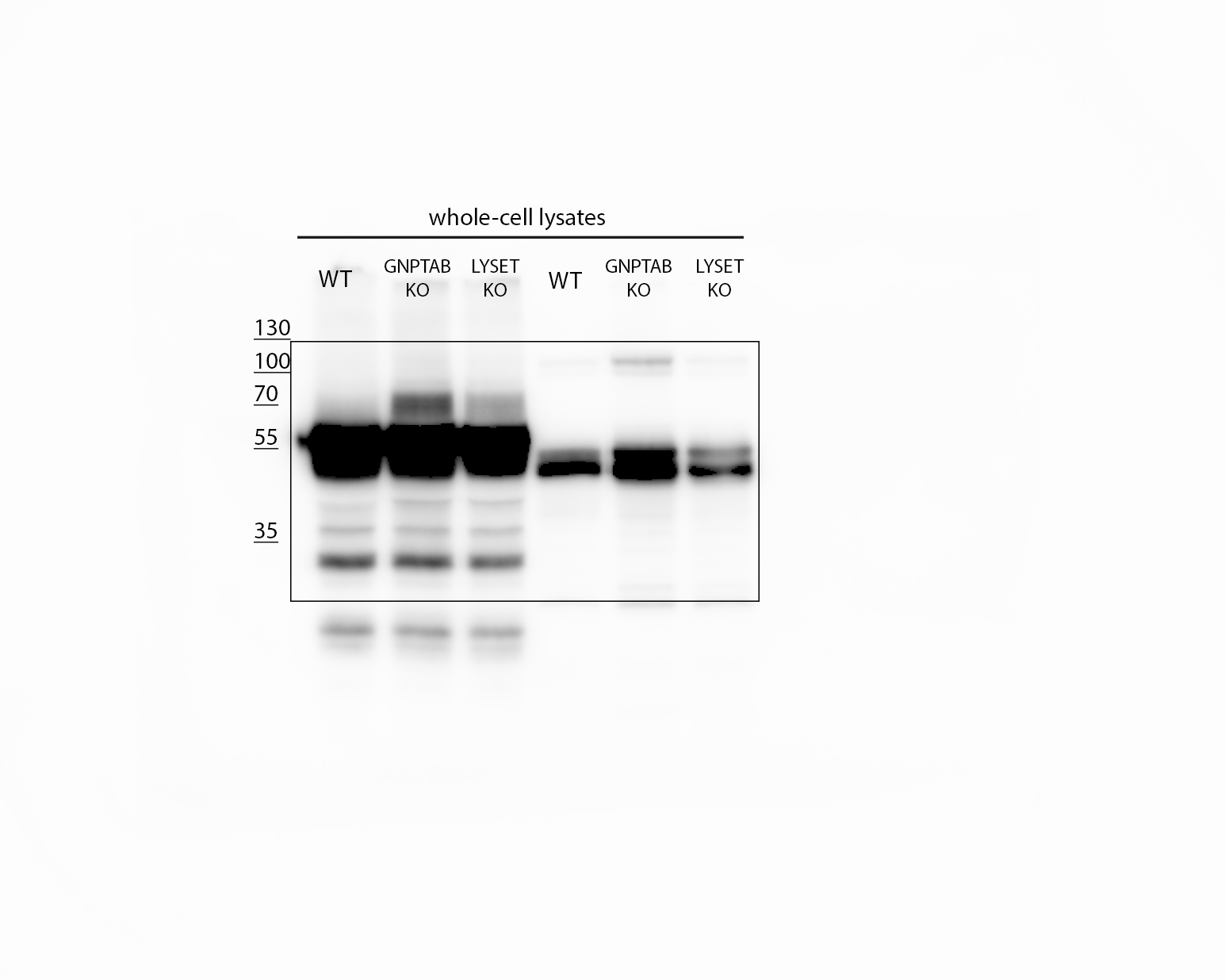

Supplement: Supplementary file 5 — Source data Fig. 2 [file 44318_2024_305_MOESM5_ESM.zip › Figure 2/2F/source data V5 (cell lysates).tif]

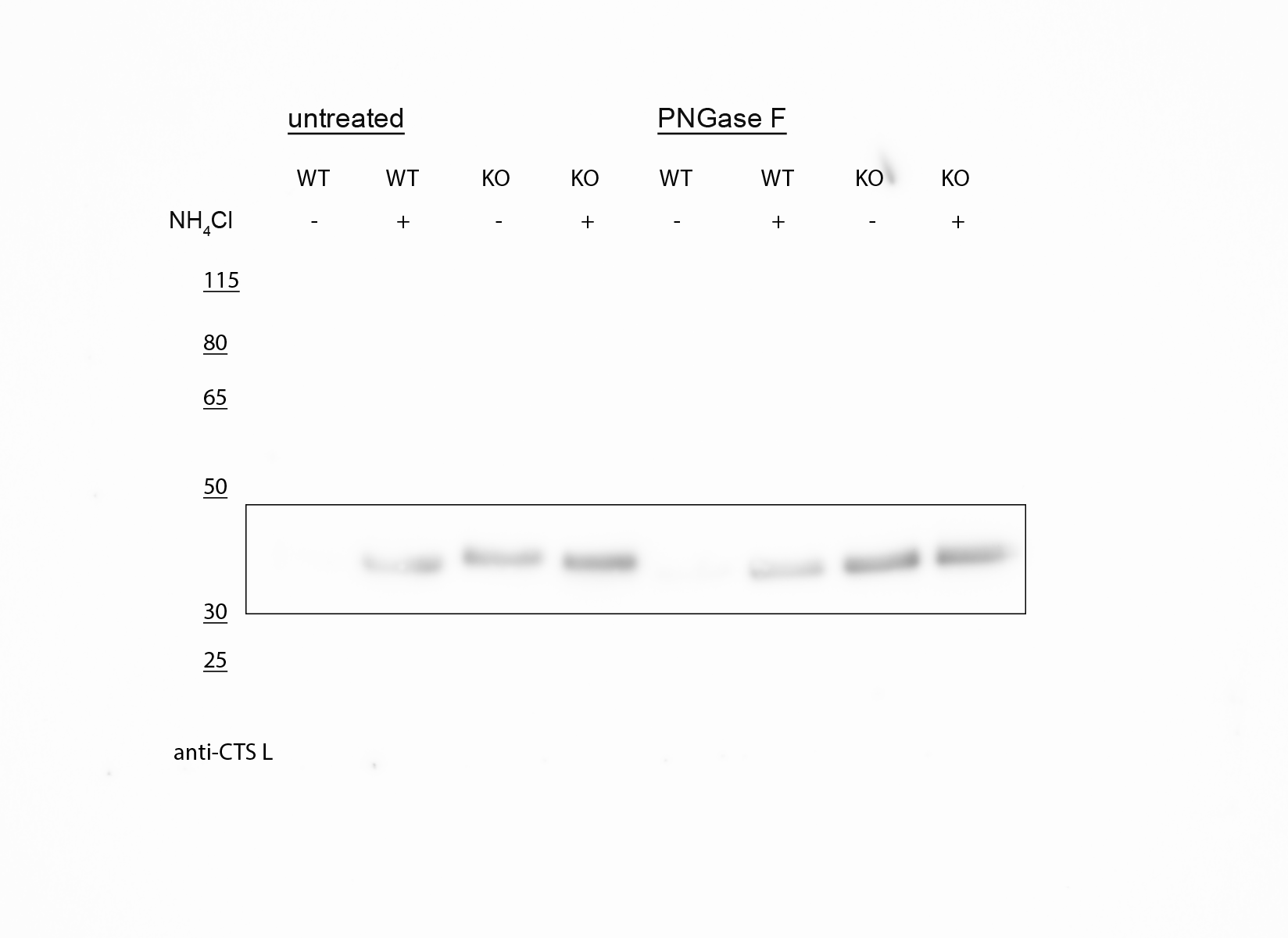

Supplement: Supplementary file 5 — Source data Fig. 2 [file 44318_2024_305_MOESM5_ESM.zip › Figure 2/2C/source data CTSL 20240110_172217-17_Ch_Chemi.tif]

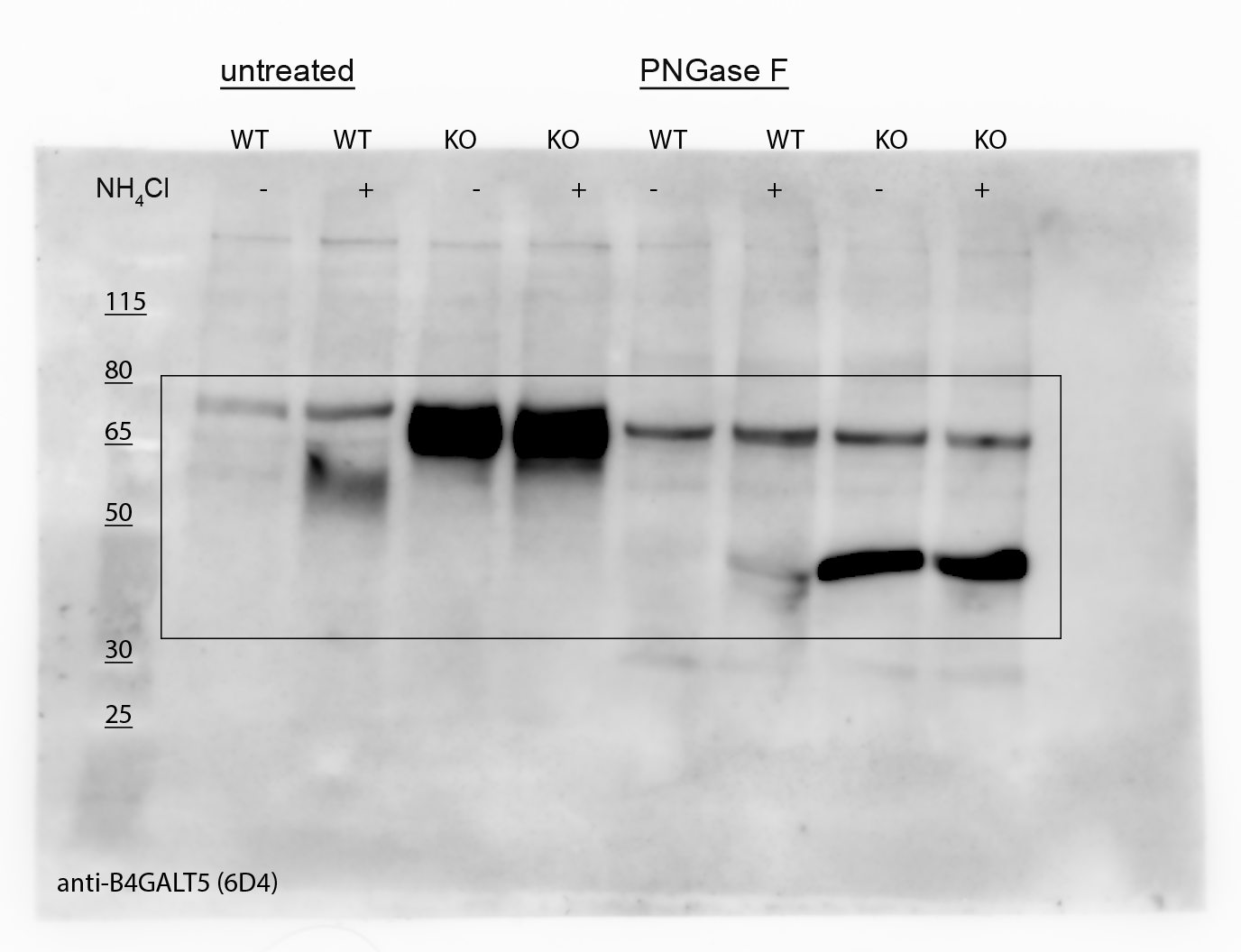

Supplement: Supplementary file 5 — Source data Fig. 2 [file 44318_2024_305_MOESM5_ESM.zip › Figure 2/2C/source data B4GALT5 6D4 (LYSET KO) 20240110_152919-14_Ch_Chemi.tif]

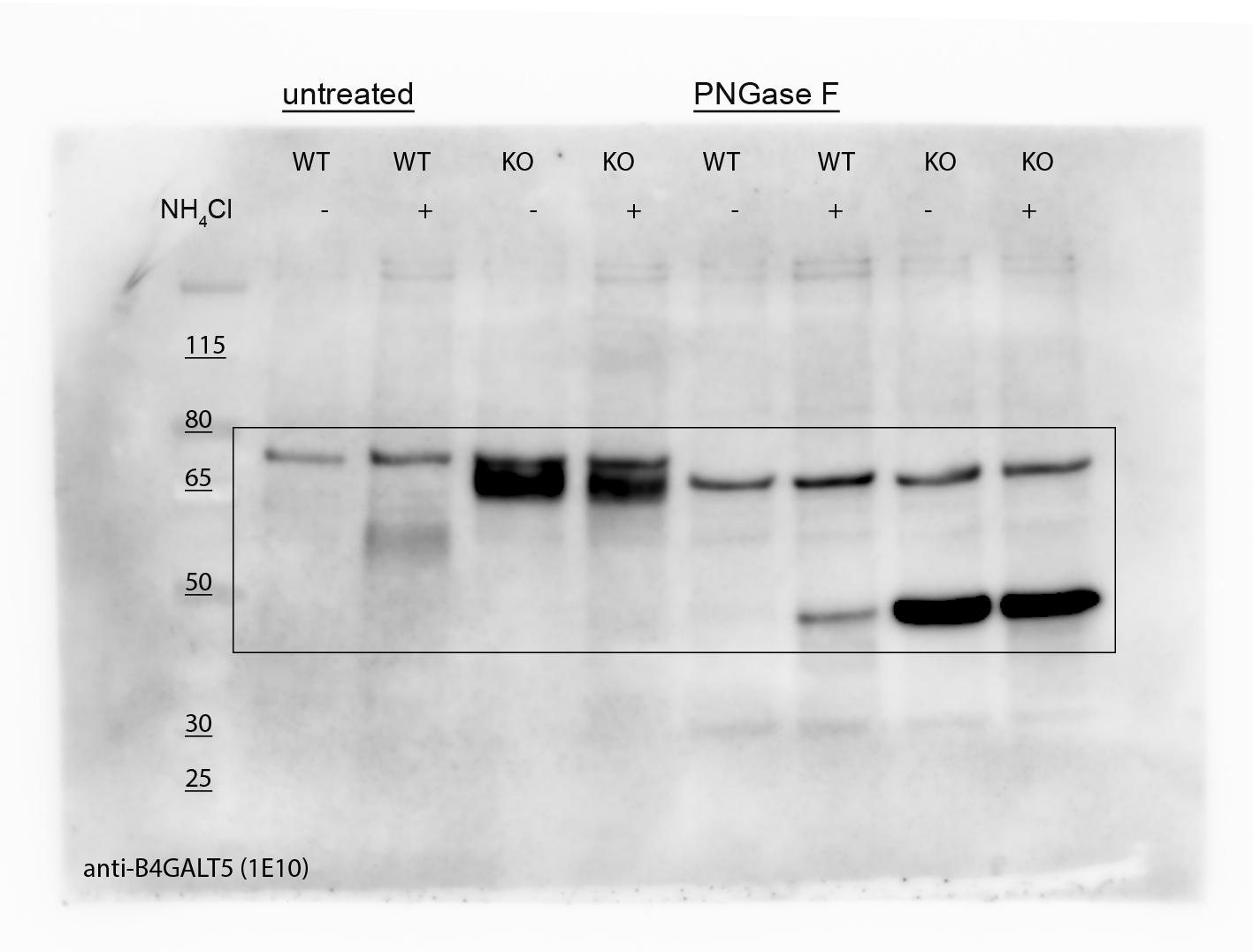

Supplement: Supplementary file 5 — Source data Fig. 2 [file 44318_2024_305_MOESM5_ESM.zip › Figure 2/2C/source data 09.1 B4GALT5 IE10 atto time series 20231221_152857-24_Ch_Chemi.tif]

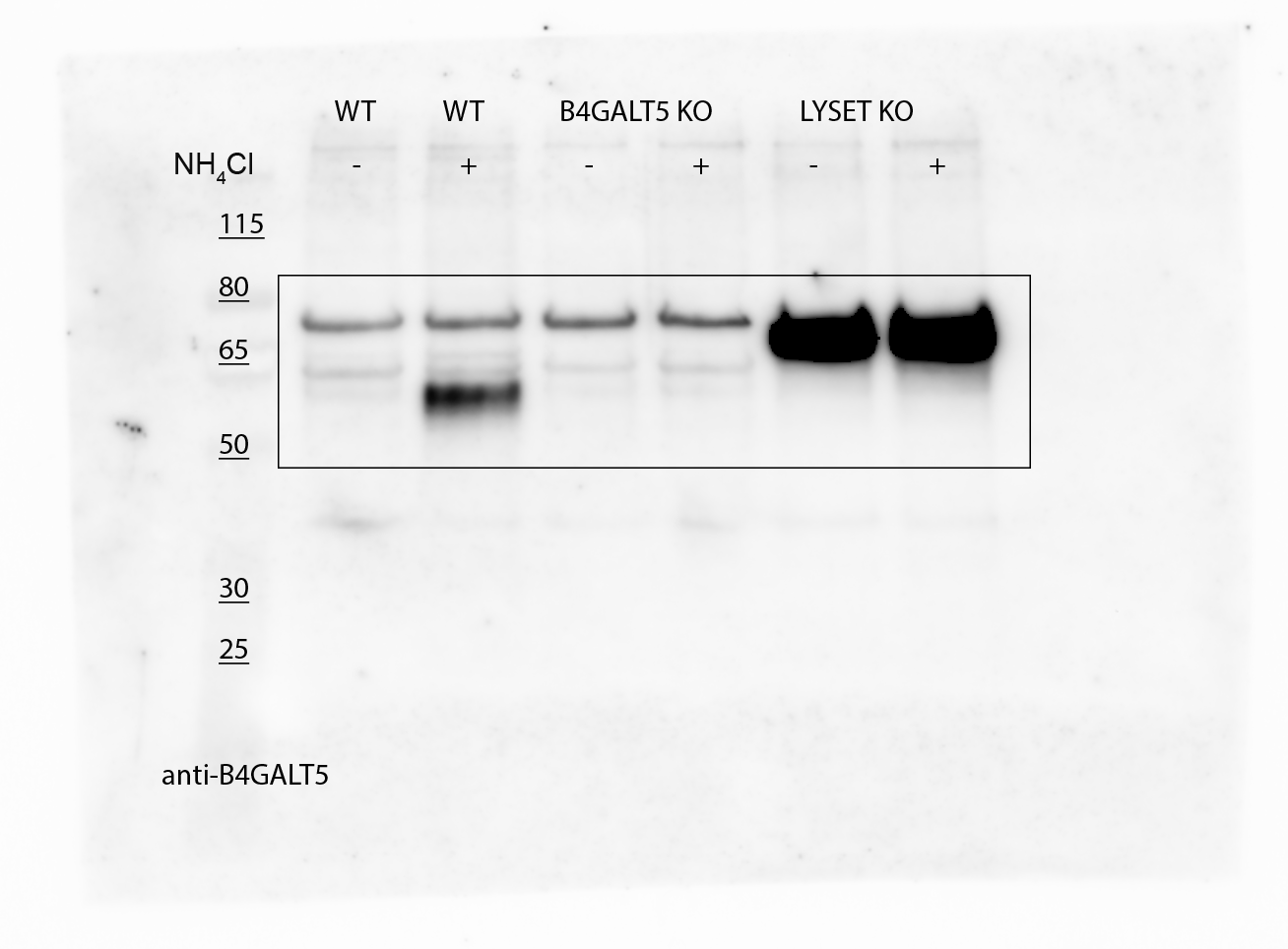

Supplement: Supplementary file 5 — Source data Fig. 2 [file 44318_2024_305_MOESM5_ESM.zip › Figure 2/2D/source data B4GALT5 long exp. time series 20240201_143925-20_Ch_Chemi.tif]

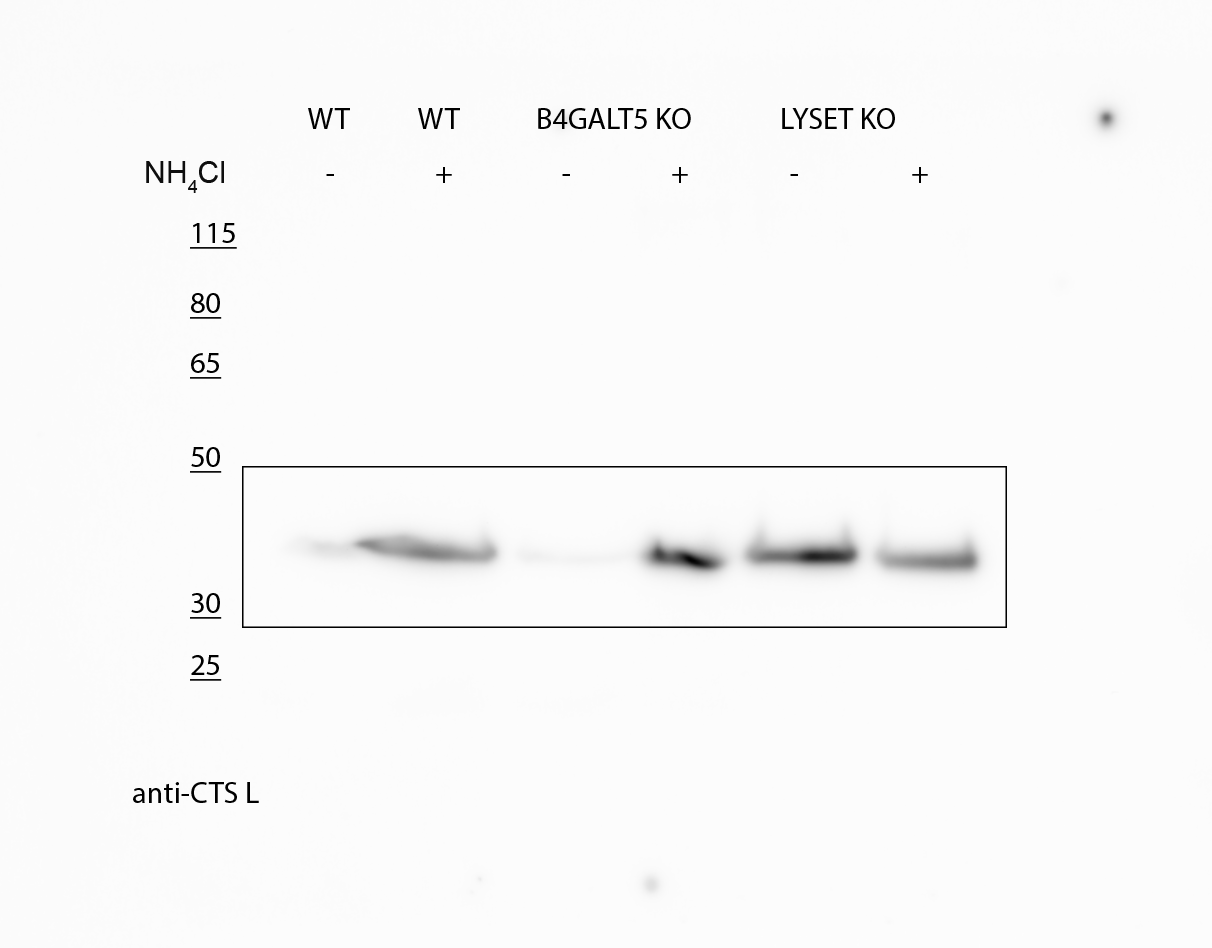

Supplement: Supplementary file 5 — Source data Fig. 2 [file 44318_2024_305_MOESM5_ESM.zip › Figure 2/2D/source data CTSL weak time series 20240202_132458-21_Ch_Chemi.tif]

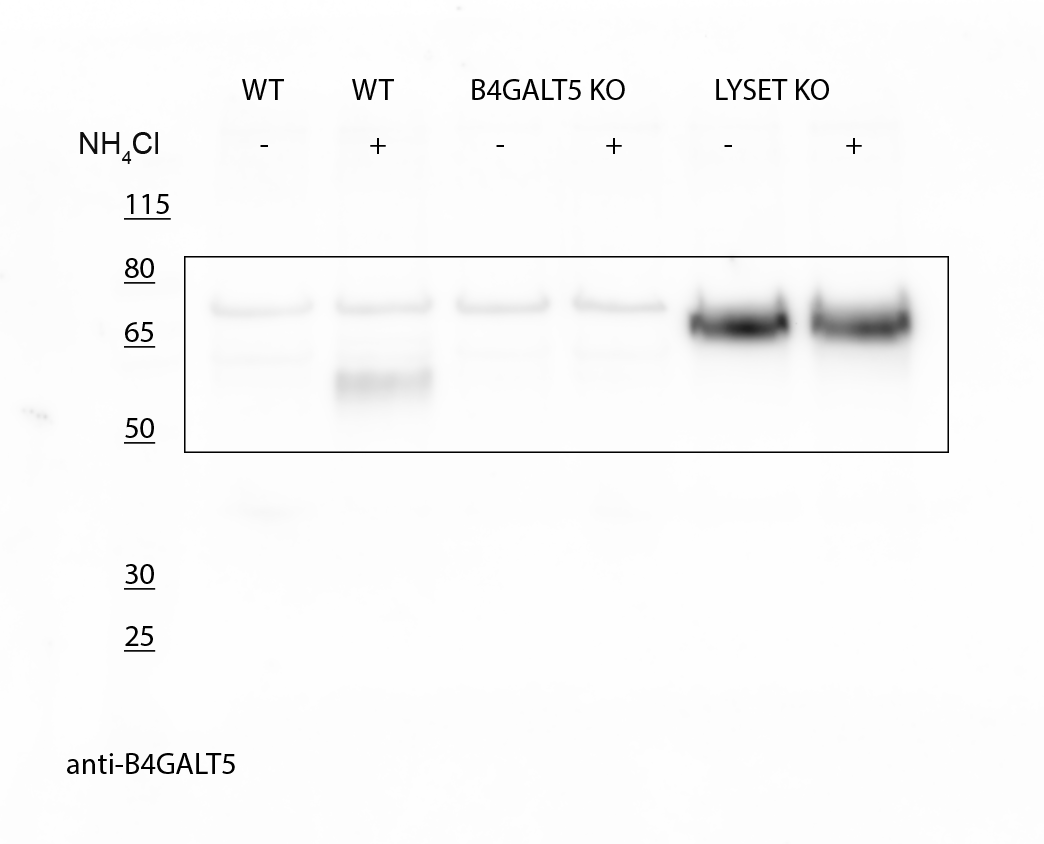

Supplement: Supplementary file 5 — Source data Fig. 2 [file 44318_2024_305_MOESM5_ESM.zip › Figure 2/2D/source data B4GALT5 6D4 short exp. time series 20240201_143925-03_Ch_Chemi.tif]

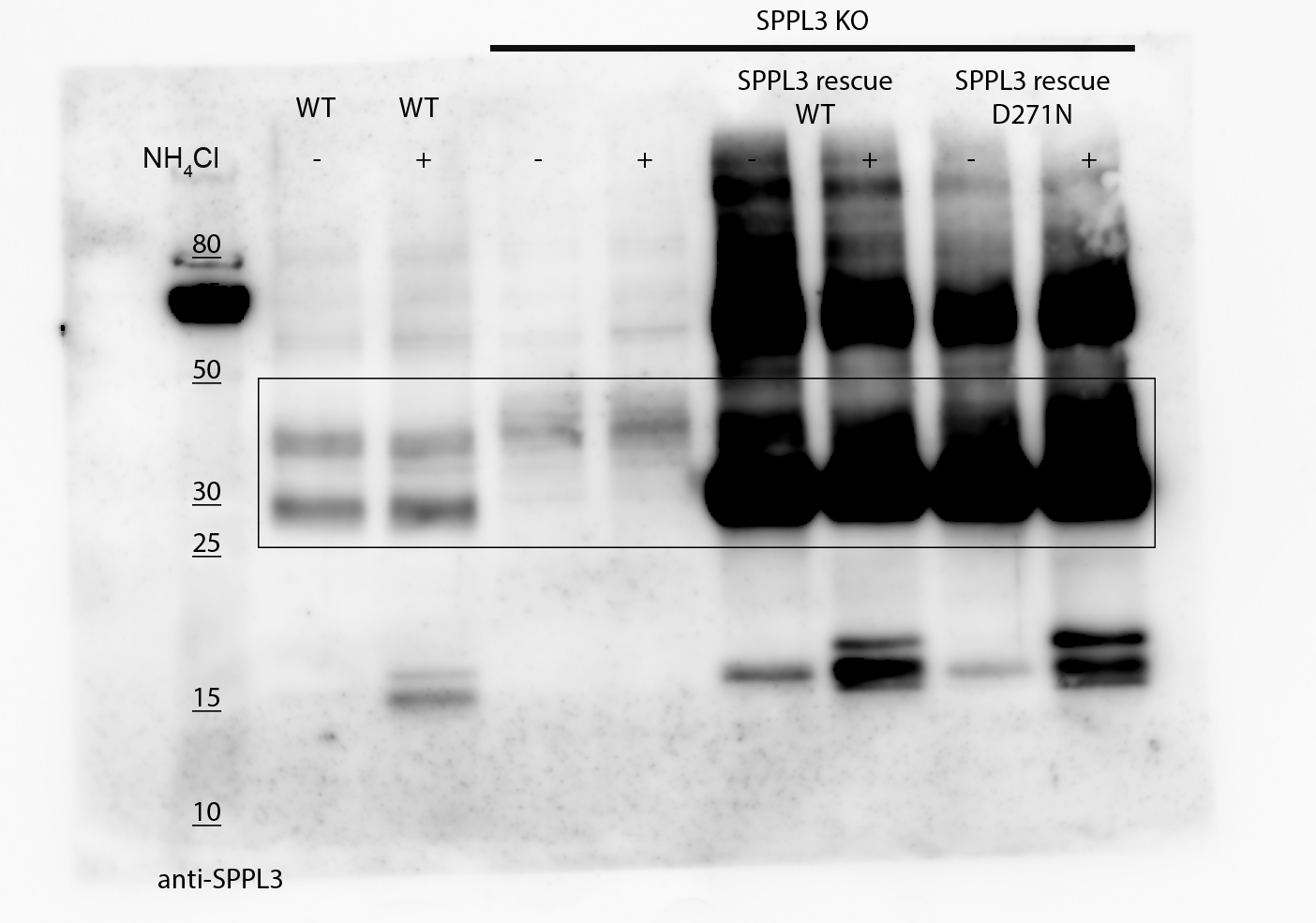

Supplement: Supplementary file 5 — Source data Fig. 2 [file 44318_2024_305_MOESM5_ESM.zip › Figure 2/2E/source data SPPL3 long exp. time series 20240223_123357-40_Ch_Chemi.tif]

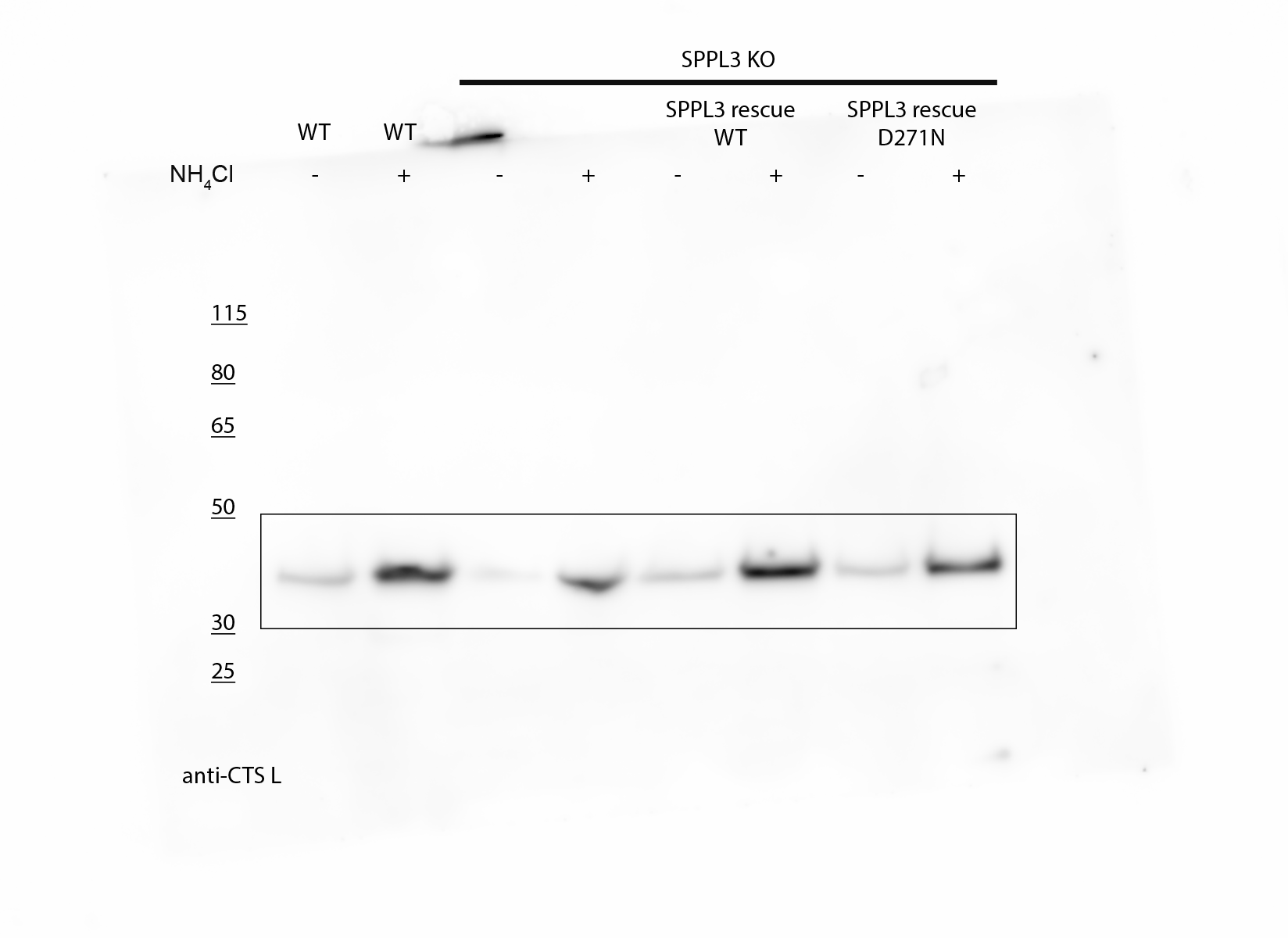

Supplement: Supplementary file 5 — Source data Fig. 2 [file 44318_2024_305_MOESM5_ESM.zip › Figure 2/2E/source data 12.1 CTSL strong time series 20240208_161009-07_Ch_Chemi.tif]

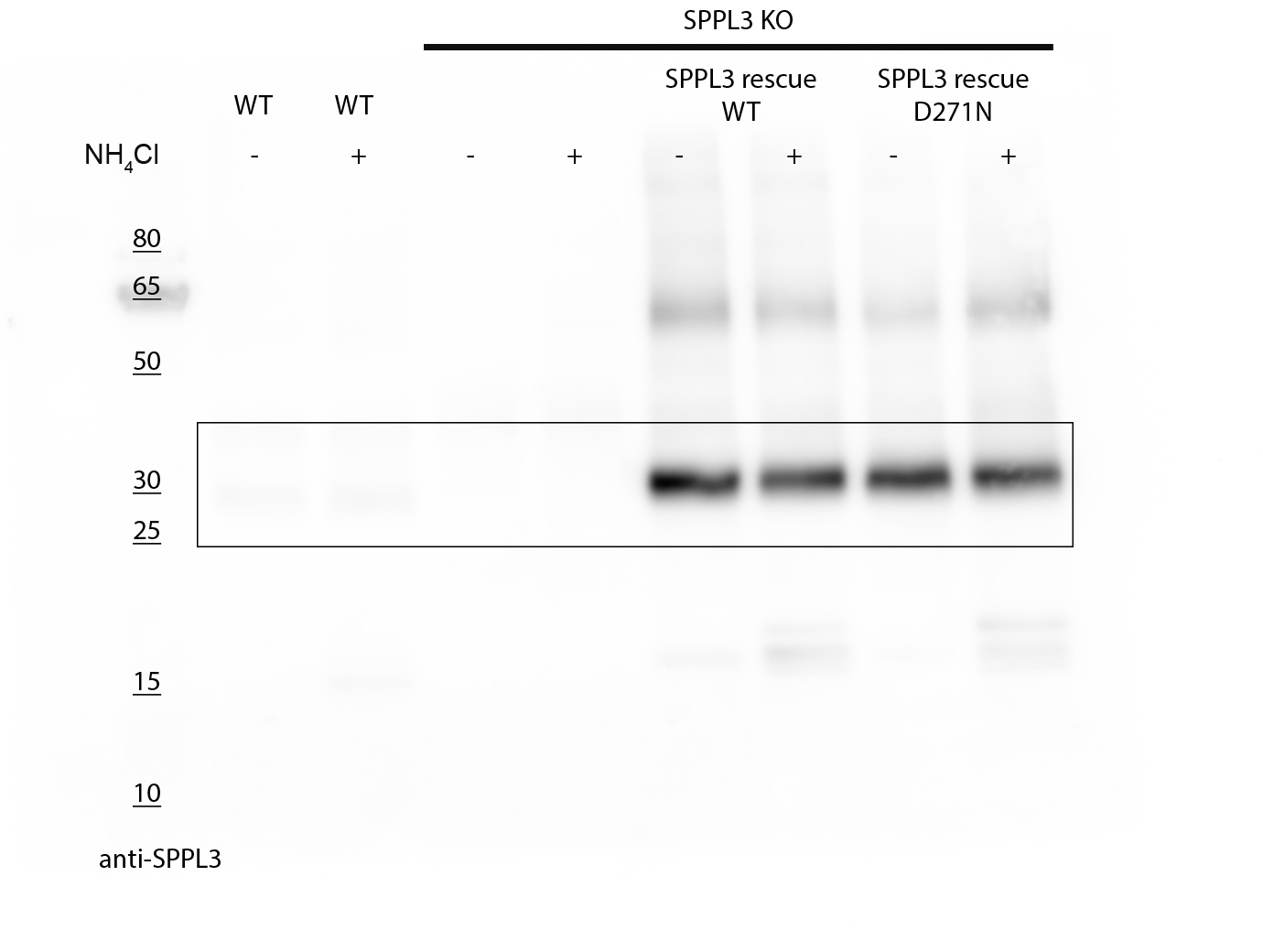

Supplement: Supplementary file 5 — Source data Fig. 2 [file 44318_2024_305_MOESM5_ESM.zip › Figure 2/2E/source data SPPL3 short exp. 20240223_123357-02_Ch_Chemi.tif]

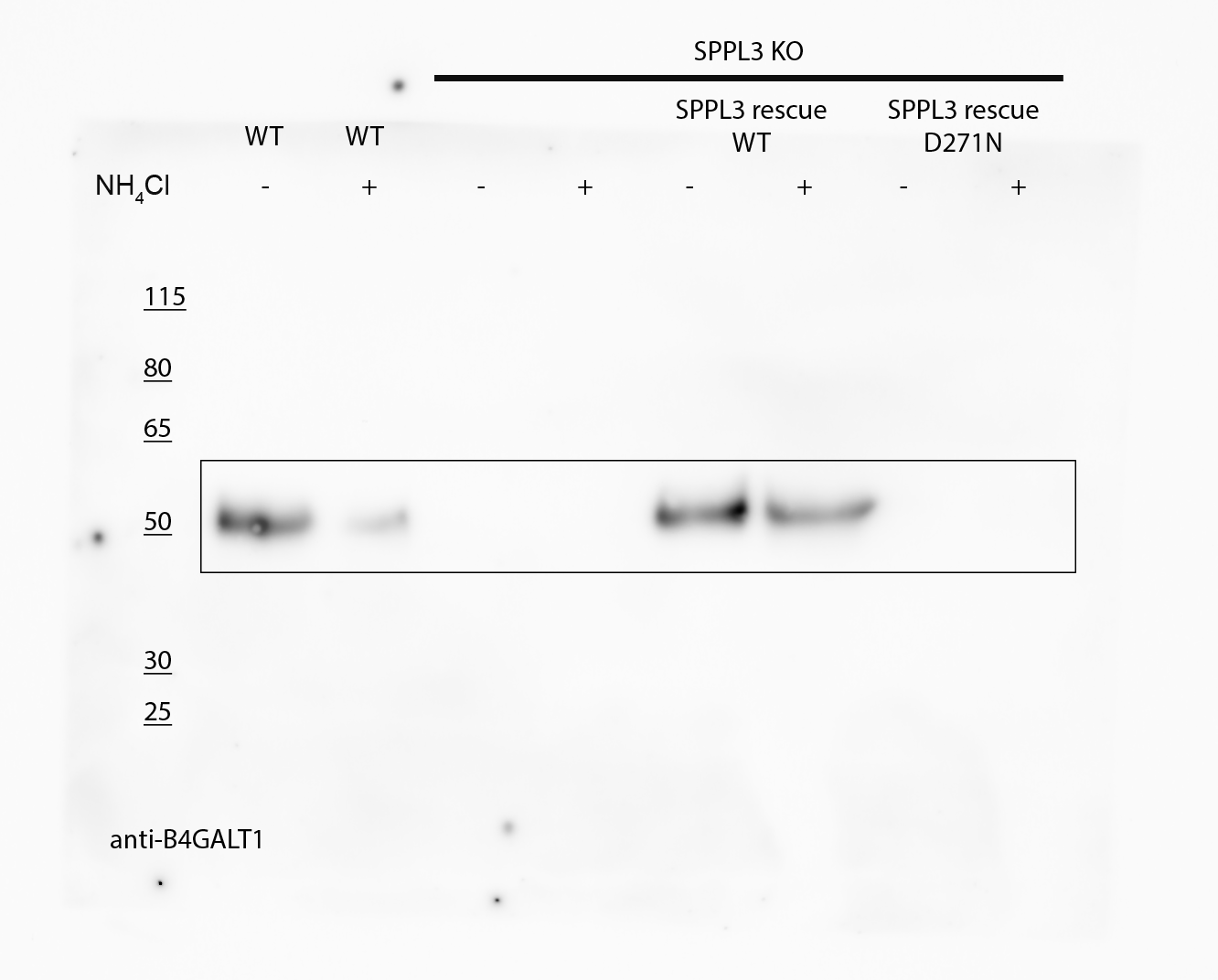

Supplement: Supplementary file 5 — Source data Fig. 2 [file 44318_2024_305_MOESM5_ESM.zip › Figure 2/2E/source data 12.2 B4GALT1 strong time series 20240209_122704-29_Ch_Chemi.tif]

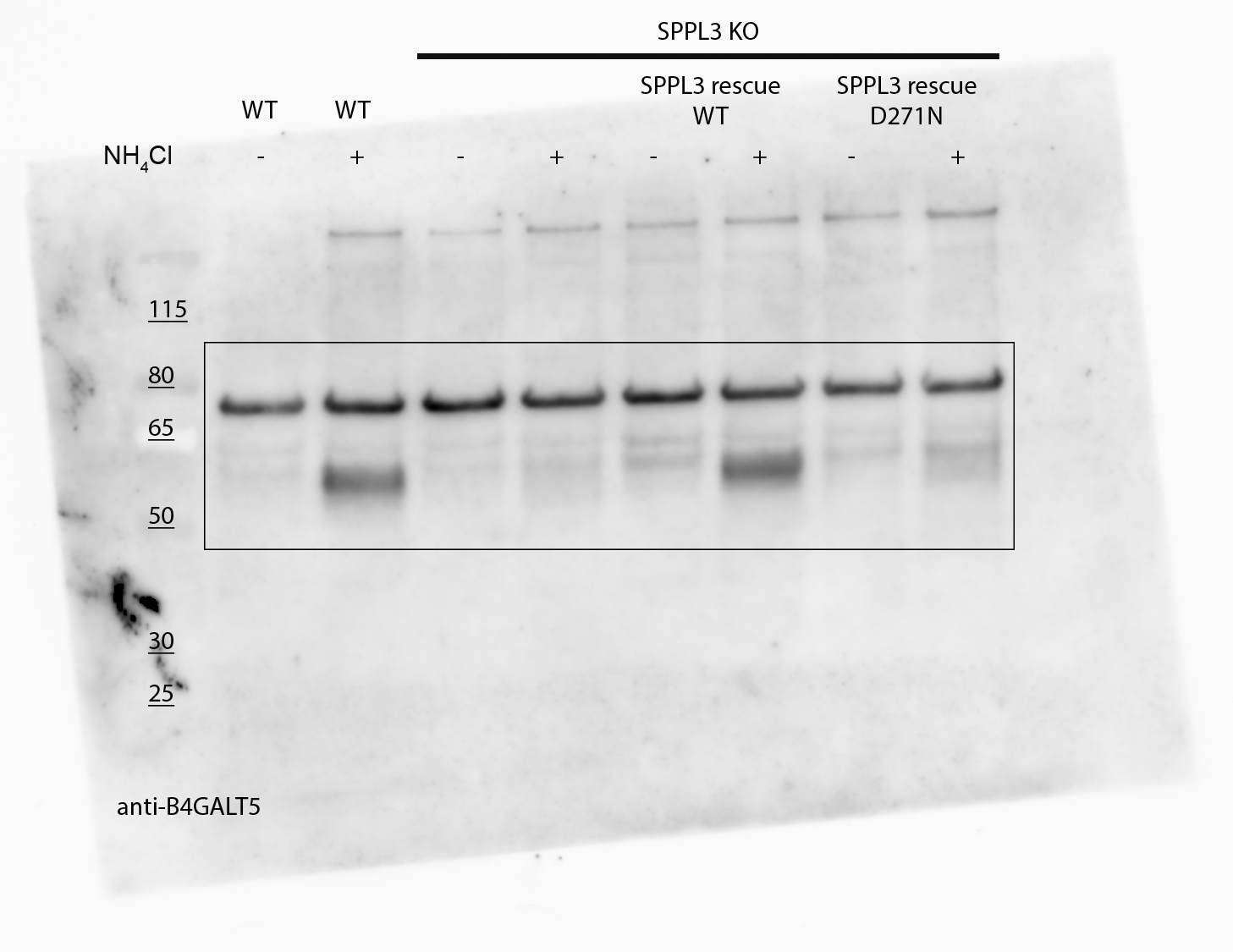

Supplement: Supplementary file 5 — Source data Fig. 2 [file 44318_2024_305_MOESM5_ESM.zip › Figure 2/2E/source data B4GALT5 IE10 atto time series 20240206_151100-30_Ch_Chemi.tif]

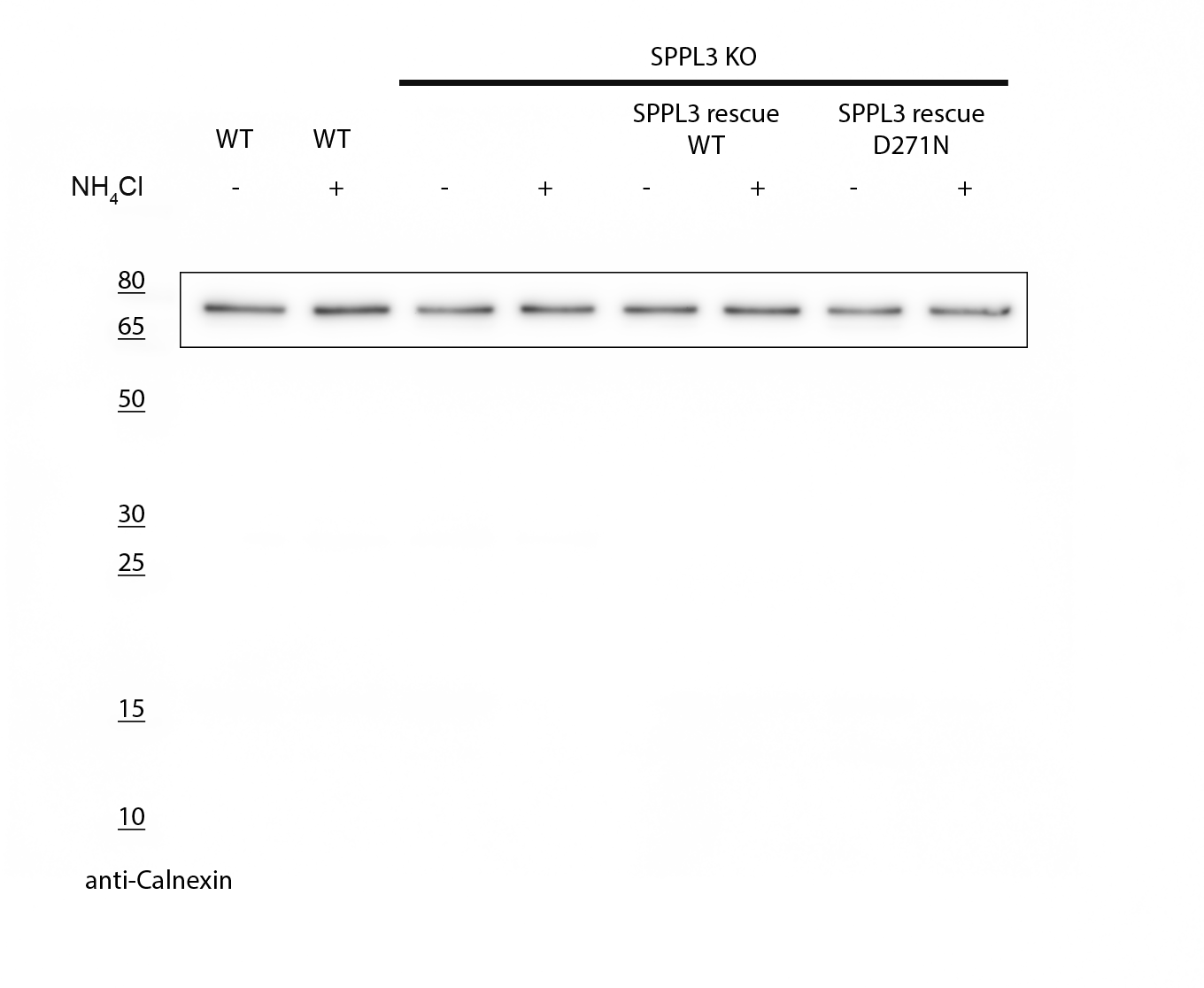

Supplement: Supplementary file 5 — Source data Fig. 2 [file 44318_2024_305_MOESM5_ESM.zip › Figure 2/2E/source data Calnexin time series 20240227_153027-03_Ch_Chemi.tif]

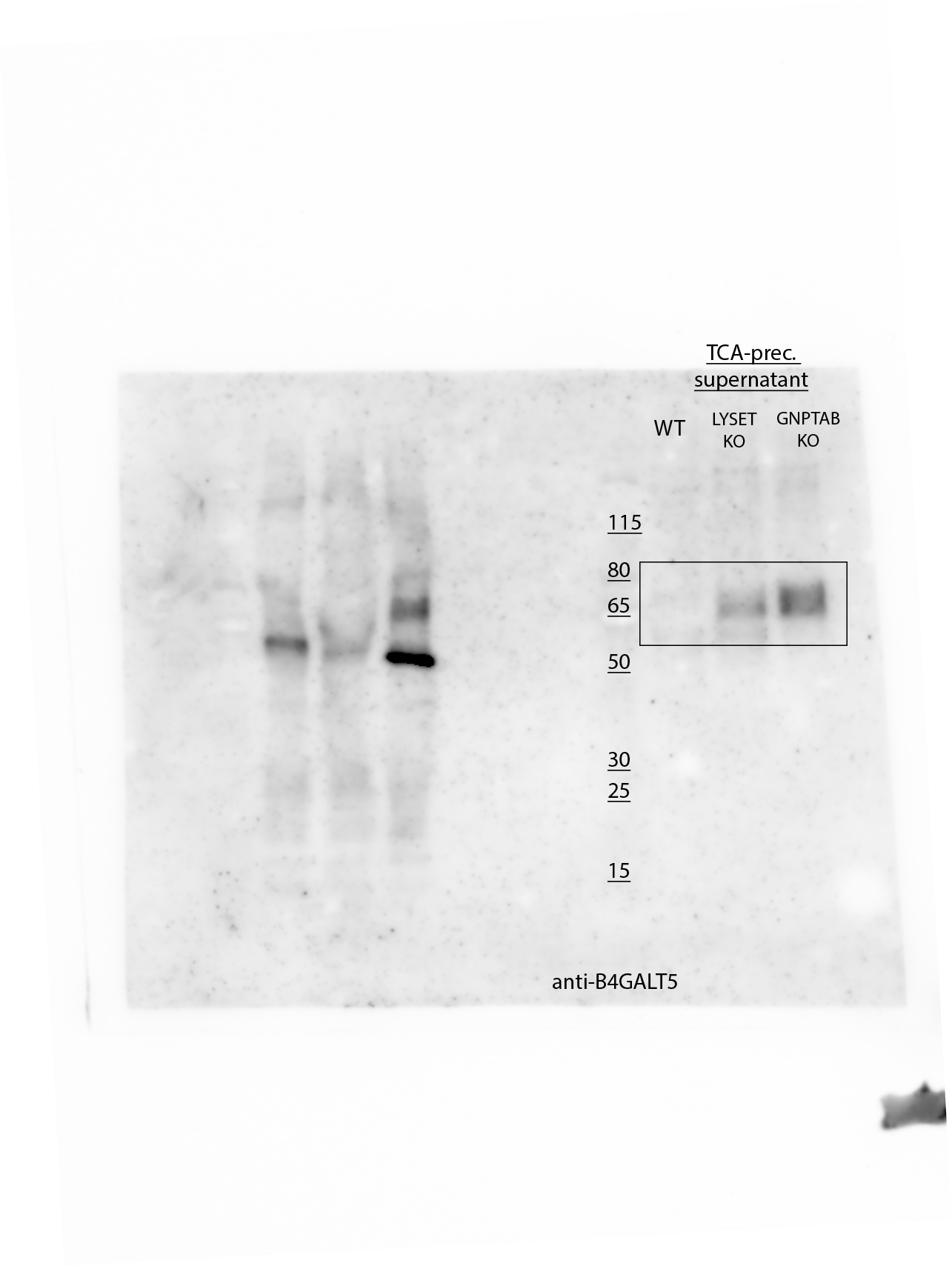

Supplement: Supplementary file 5 — Source data Fig. 2 [file 44318_2024_305_MOESM5_ESM.zip › Figure 2/2B/source data B4GALT5 6D4 serie 20231004_142650-25_Ch_Chemi.tif]

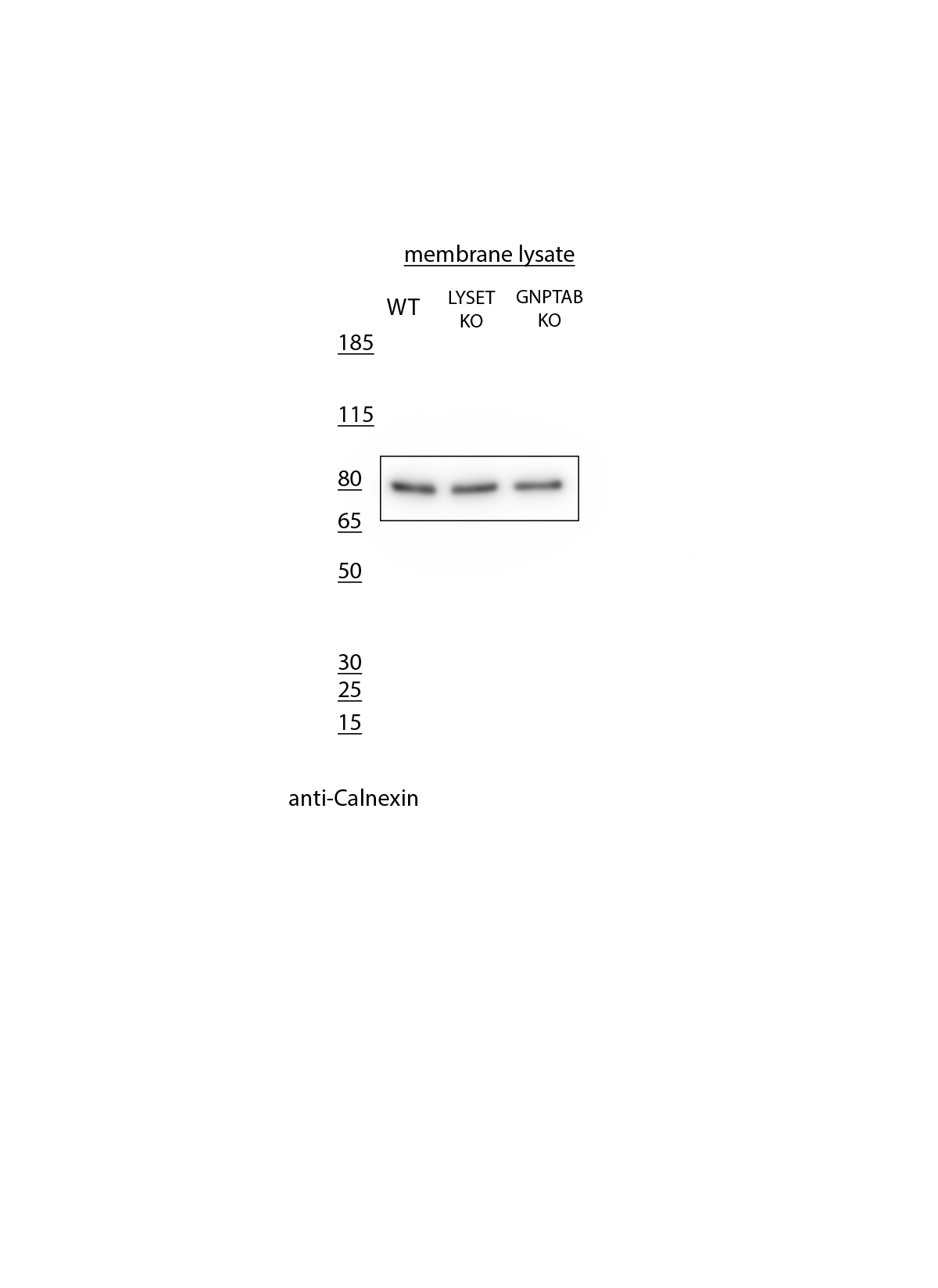

Supplement: Supplementary file 5 — Source data Fig. 2 [file 44318_2024_305_MOESM5_ESM.zip › Figure 2/2B/source data Calnexin for GNPTAB 20240929_171824-02_Ch_Chemi.tif]

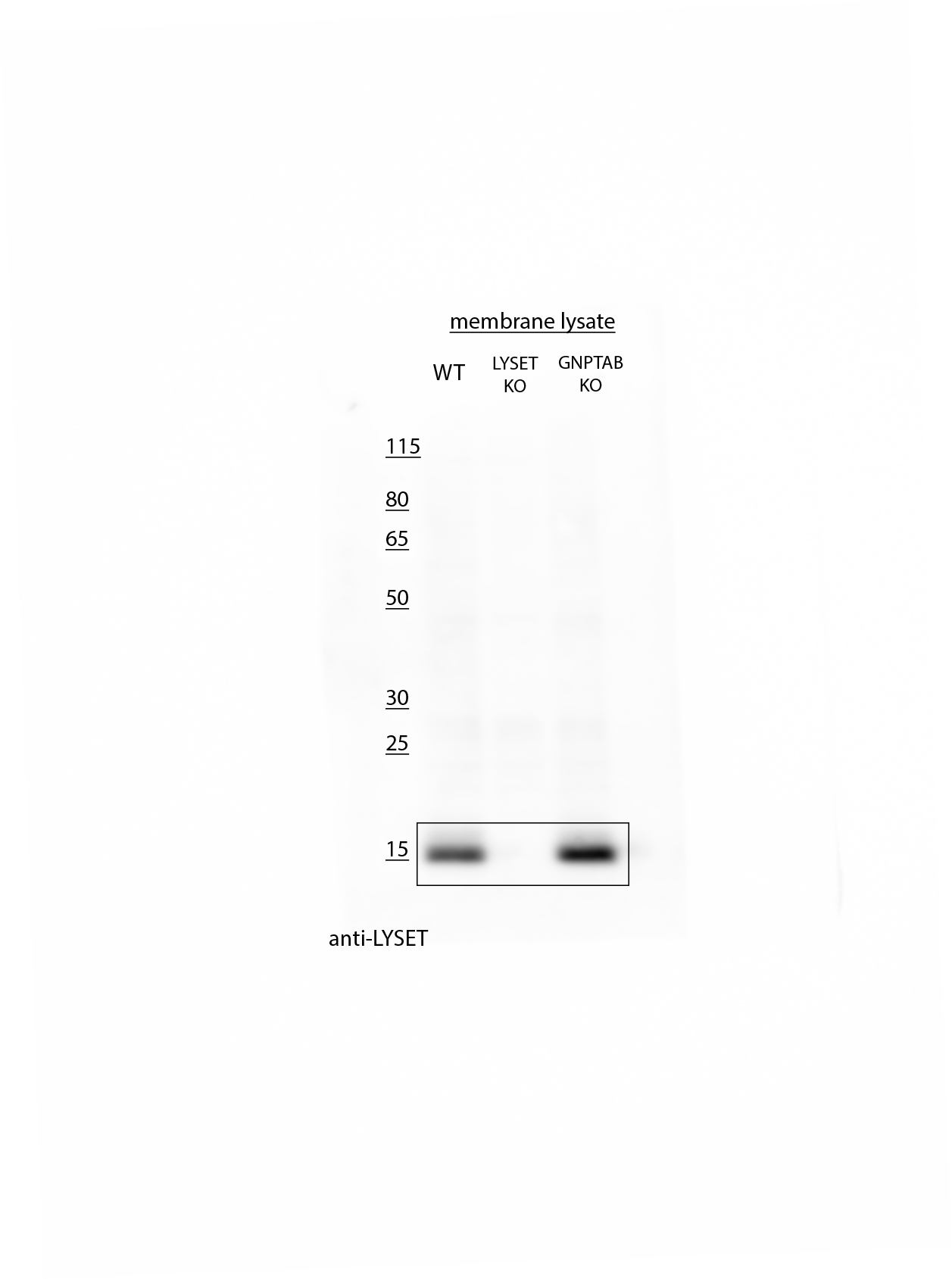

Supplement: Supplementary file 5 — Source data Fig. 2 [file 44318_2024_305_MOESM5_ESM.zip › Figure 2/2B/source data LYSET serie 20231004_132031-05_Ch_Chemi.tif]

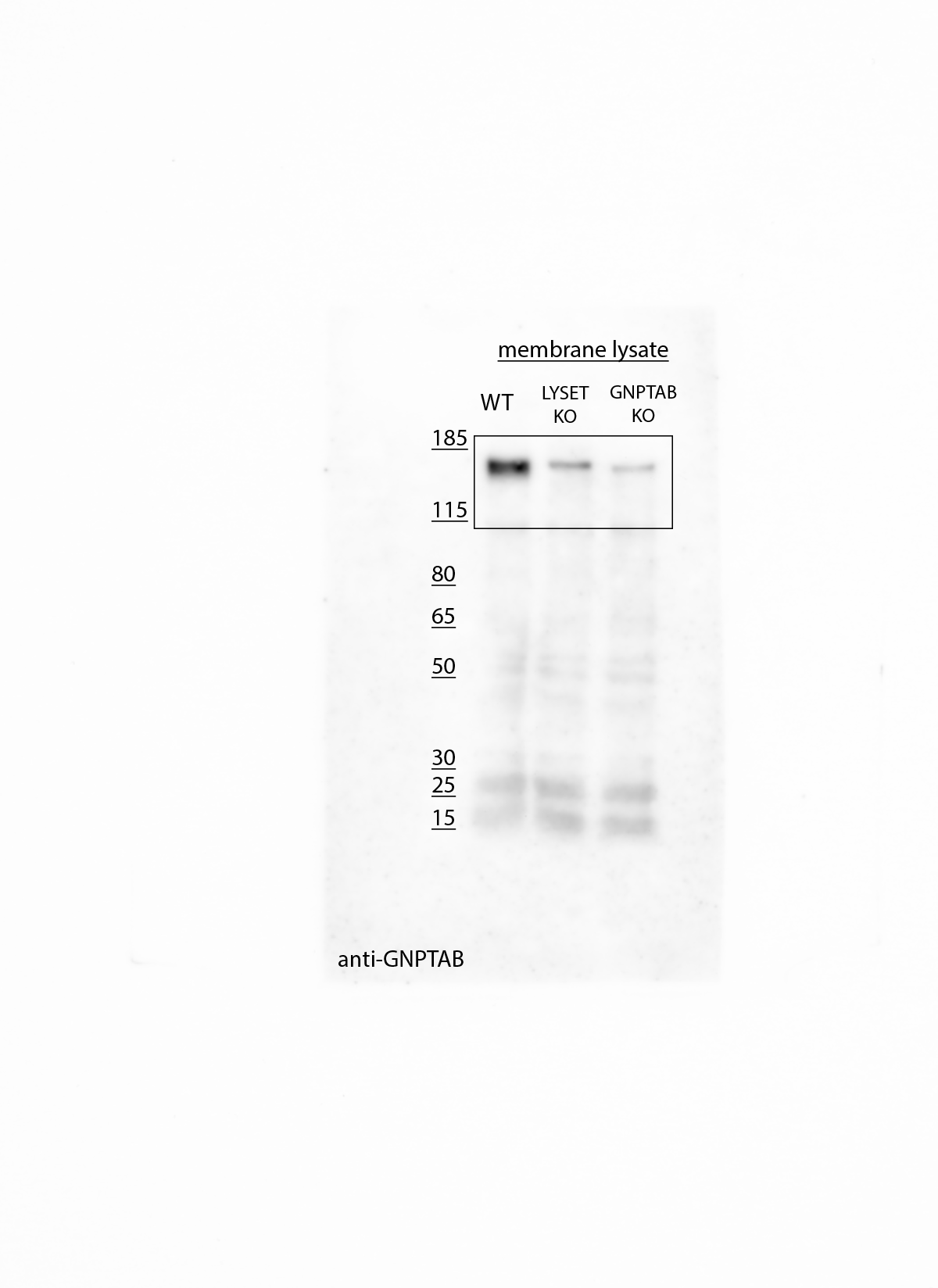

Supplement: Supplementary file 5 — Source data Fig. 2 [file 44318_2024_305_MOESM5_ESM.zip › Figure 2/2B/source data GNPTAB serie 20231004_133434-23_Ch_Chemi.tif]

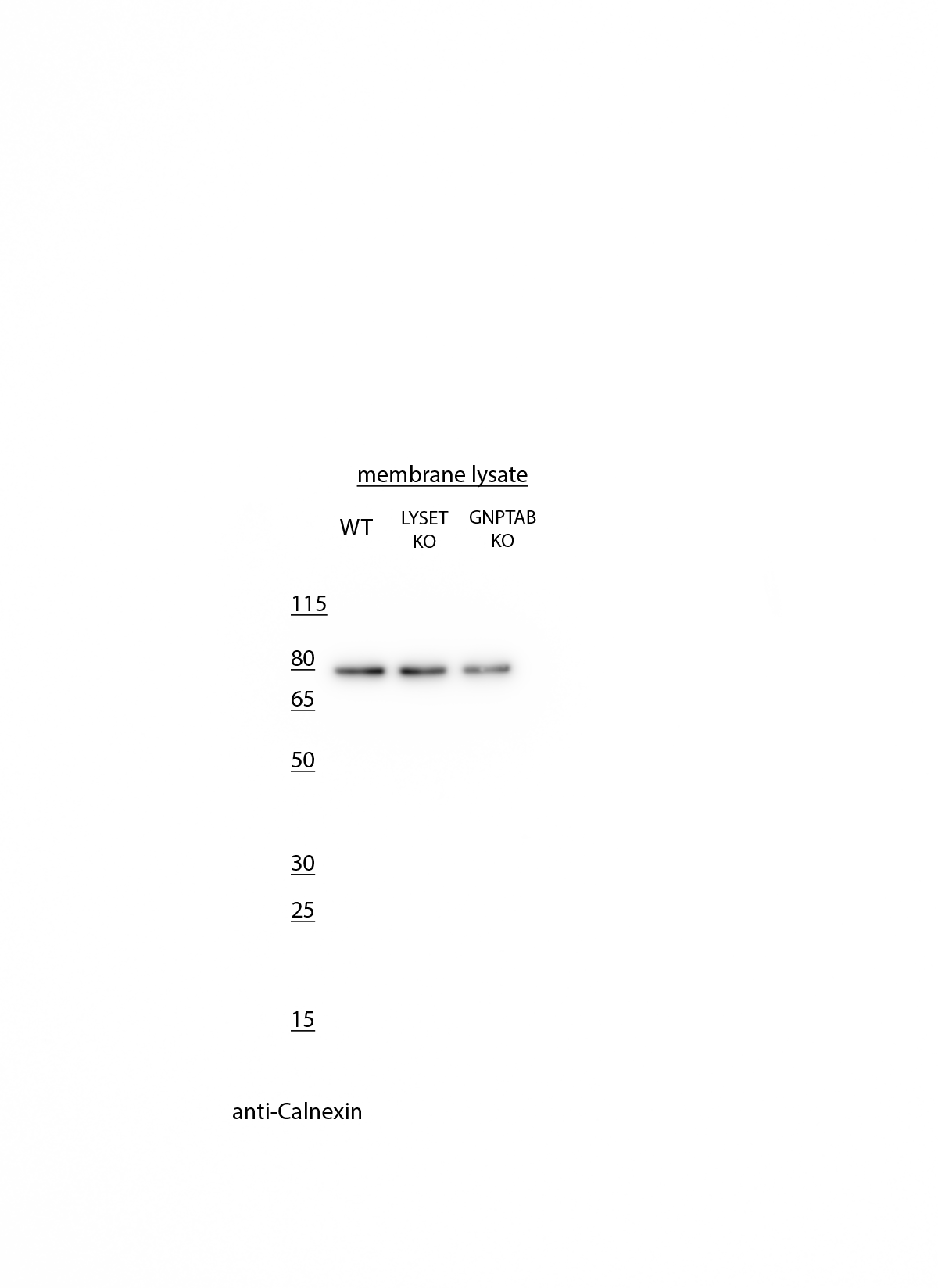

Supplement: Supplementary file 5 — Source data Fig. 2 [file 44318_2024_305_MOESM5_ESM.zip › Figure 2/2B/source data Calnexin for LYSET 20240929_172307-03_Ch_Chemi.tif]

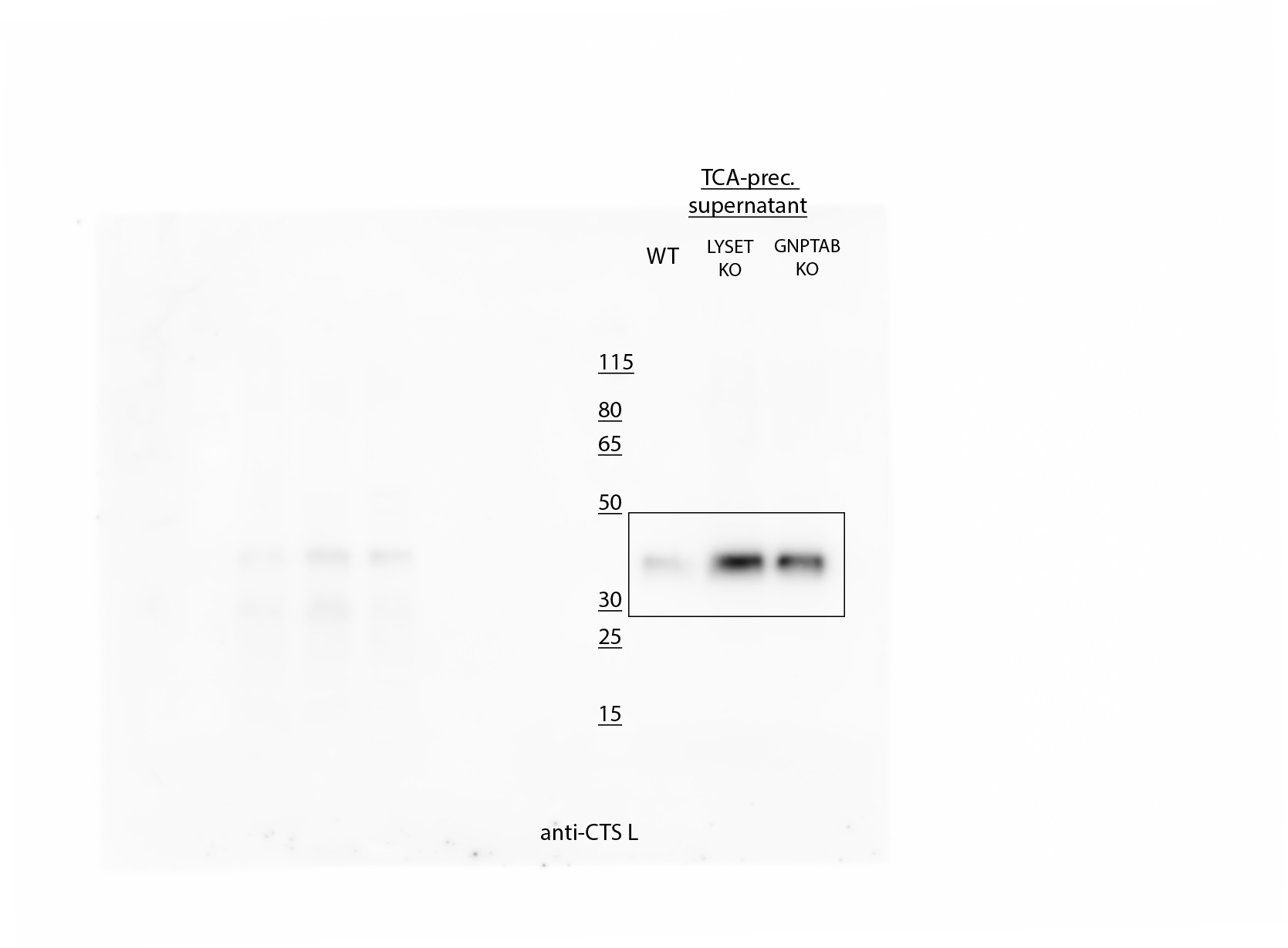

Supplement: Supplementary file 5 — Source data Fig. 2 [file 44318_2024_305_MOESM5_ESM.zip › Figure 2/2B/source data 231123_170.3 CTSL time series 20231123_125335-04_Ch_Chemi.tif]

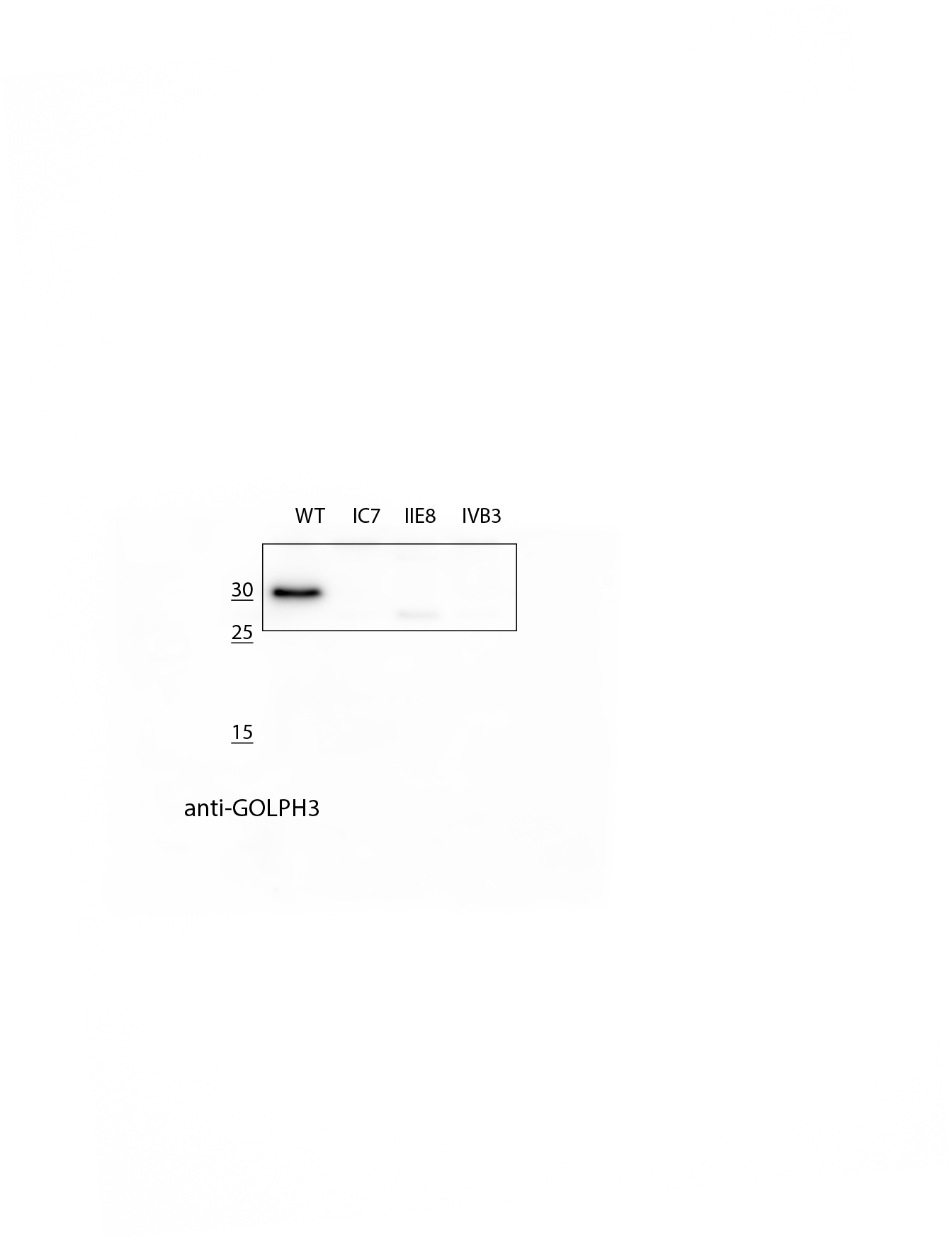

Supplement: Supplementary file 6 — Source data Fig. 3 [file 44318_2024_305_MOESM6_ESM.zip › Figure 3/3E/source data GOLPH3 20240113_122321-02_Ch_Chemi.tif]

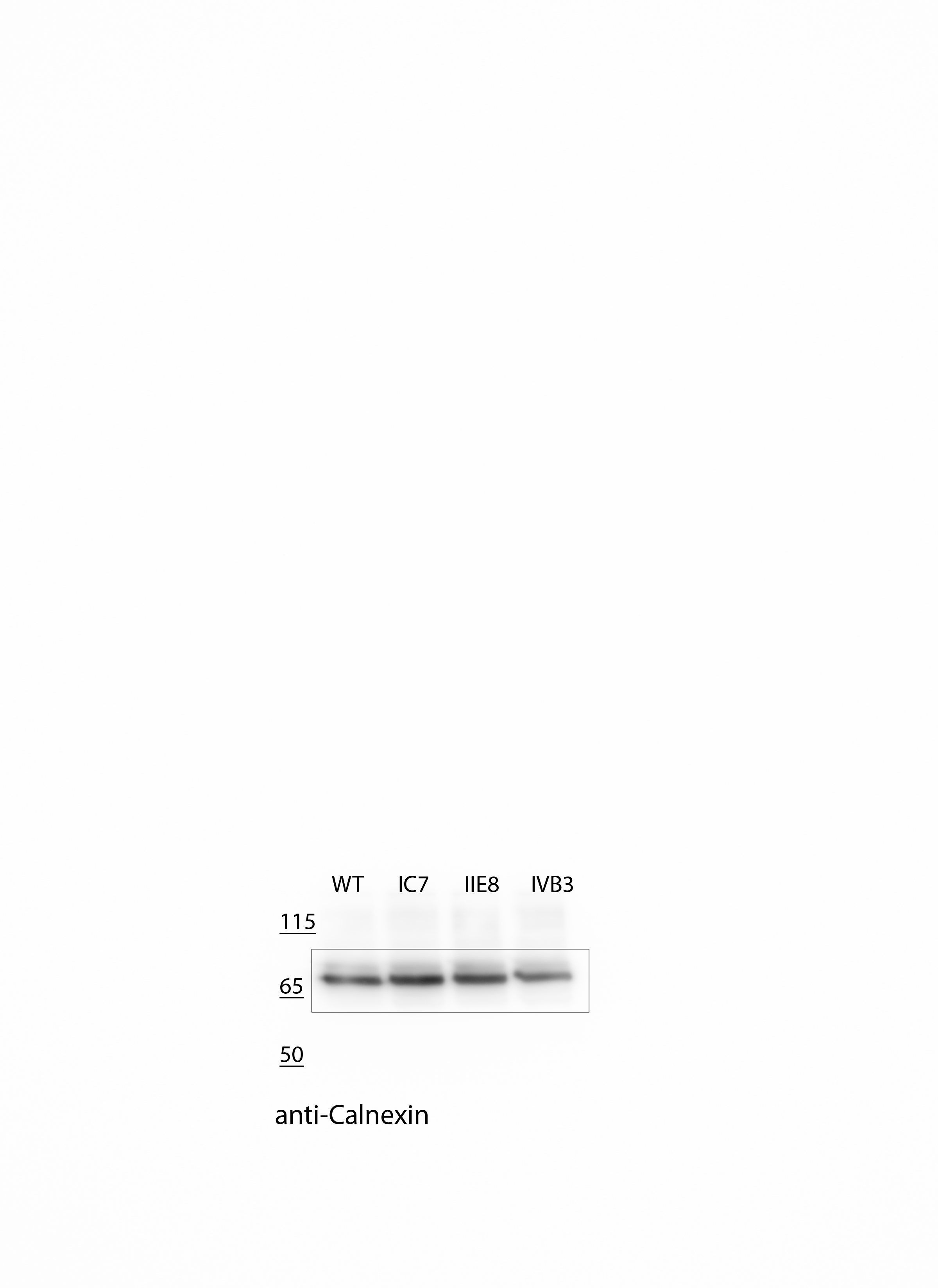

Supplement: Supplementary file 6 — Source data Fig. 3 [file 44318_2024_305_MOESM6_ESM.zip › Figure 3/3E/source data Calnexin for GOLPH3 20240113_123906-06_Ch_Chemi.tif]

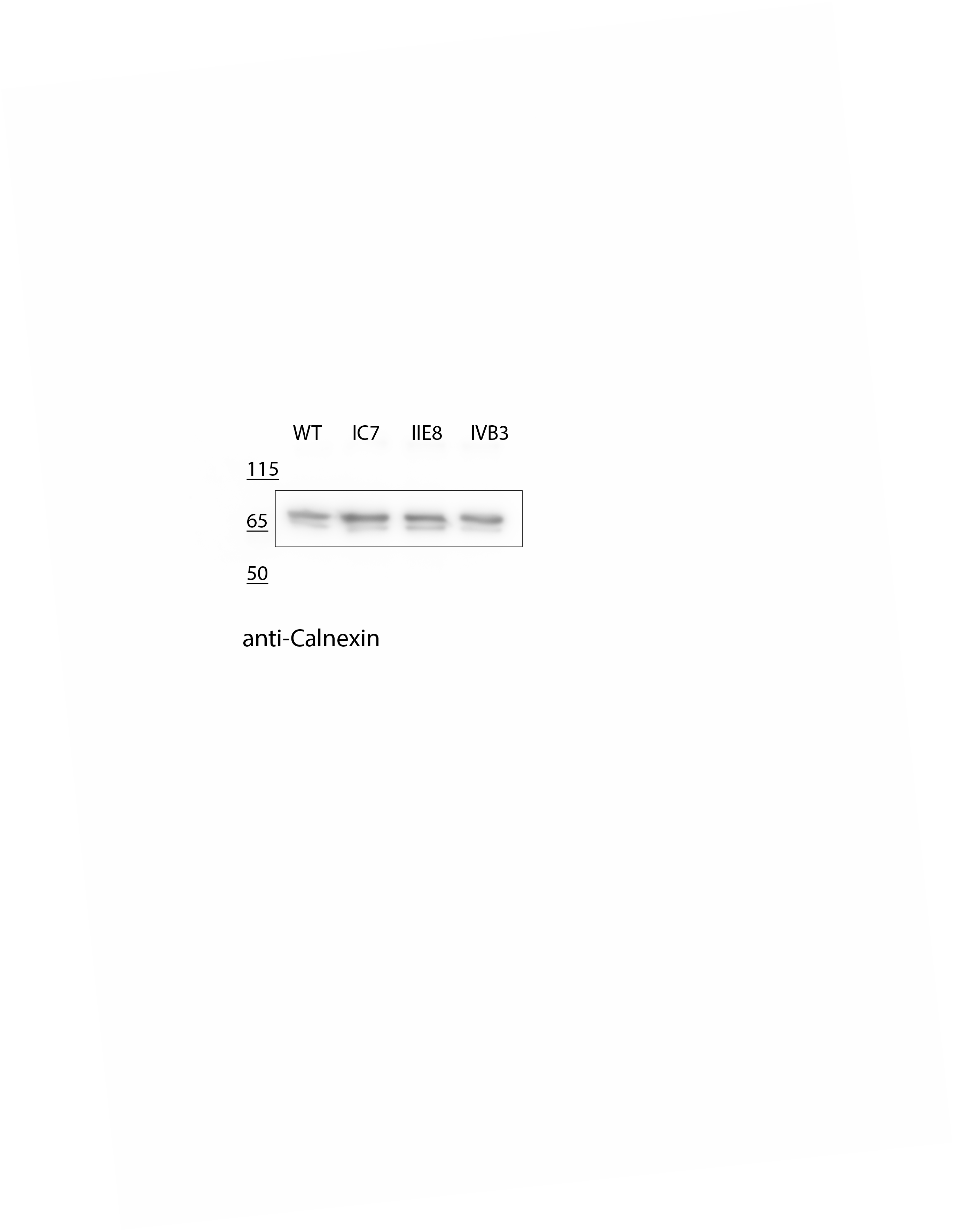

Supplement: Supplementary file 6 — Source data Fig. 3 [file 44318_2024_305_MOESM6_ESM.zip › Figure 3/3E/source data Calnexin for LYSET 20230920_150930_Ch_Chemi.tif]

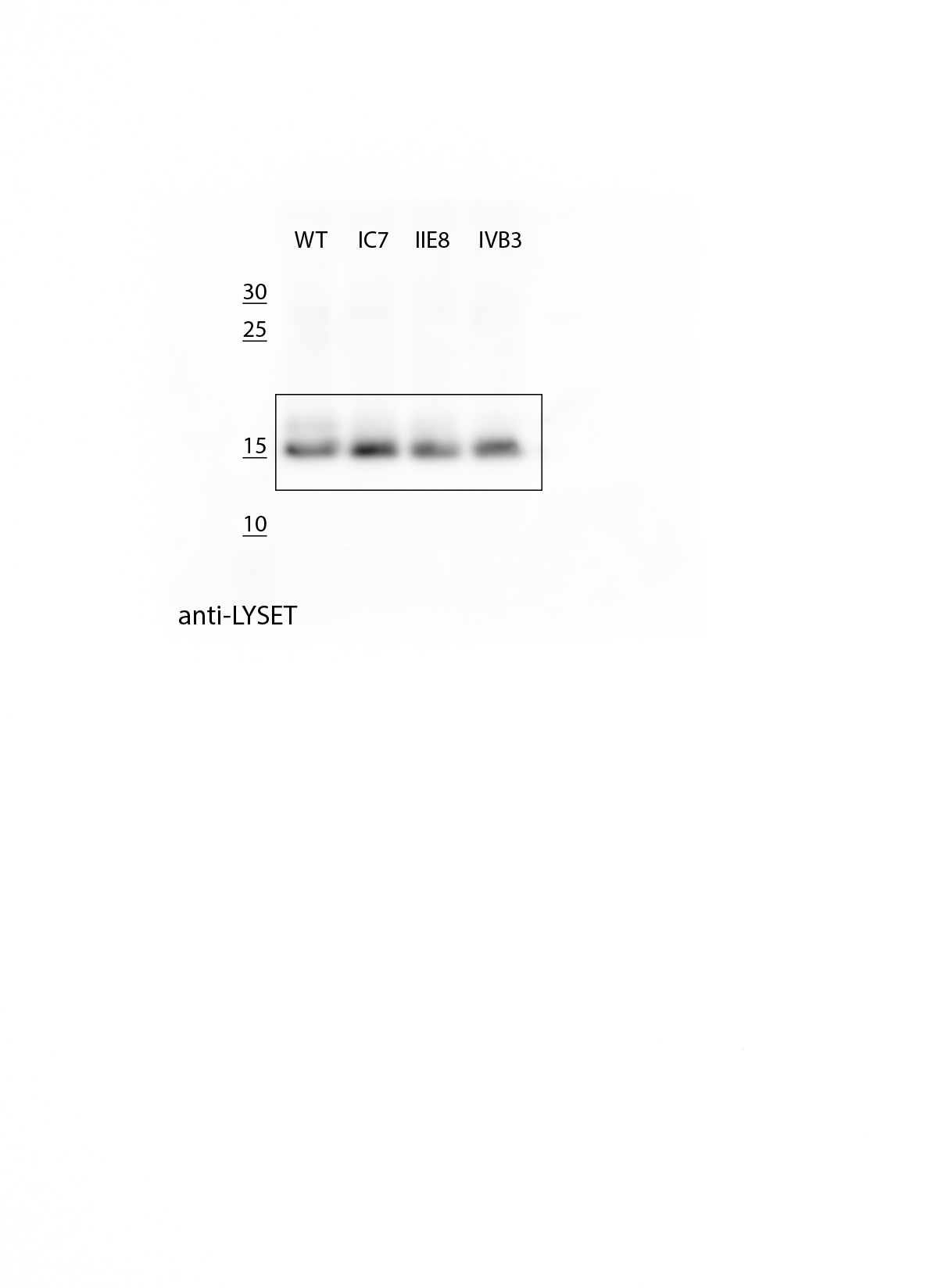

Supplement: Supplementary file 6 — Source data Fig. 3 [file 44318_2024_305_MOESM6_ESM.zip › Figure 3/3E/source data LYSET time series 20230920_151911-04_Ch_Chemi.tif]

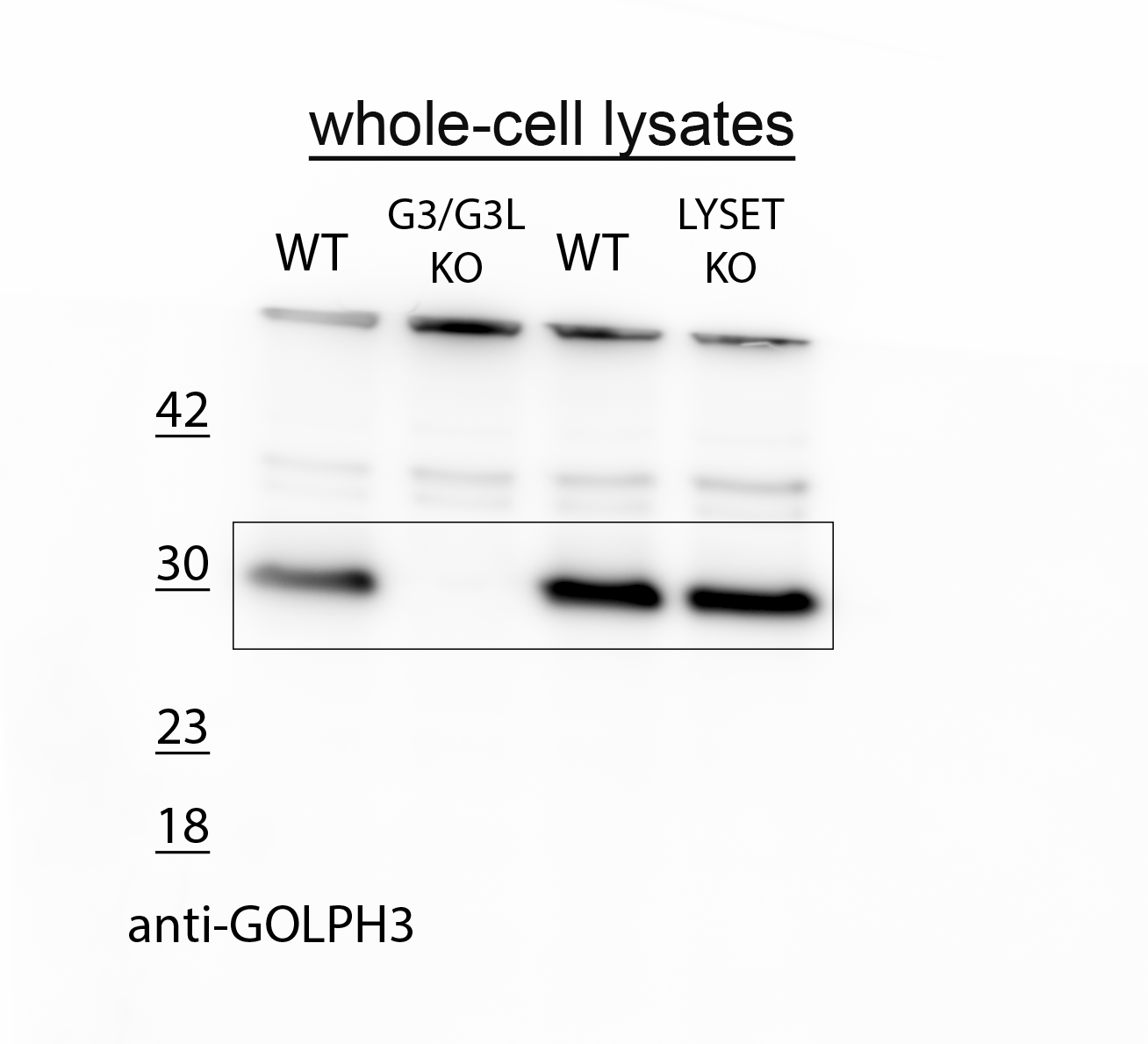

Supplement: Supplementary file 6 — Source data Fig. 3 [file 44318_2024_305_MOESM6_ESM.zip › Figure 3/3B/source data GOLPH3 time series 1x1 20230531_125050-01_Ch_Chemi.tif]

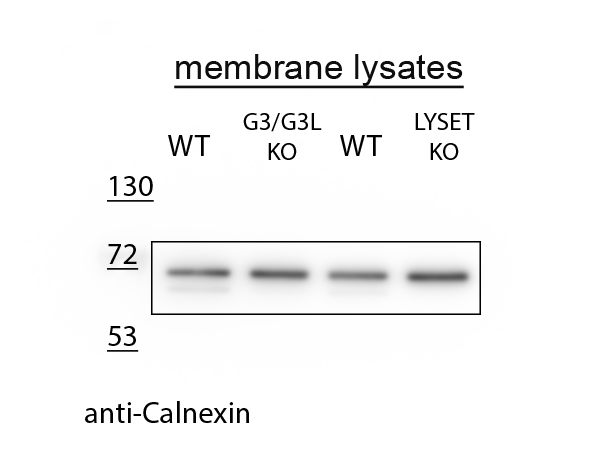

Supplement: Supplementary file 6 — Source data Fig. 3 [file 44318_2024_305_MOESM6_ESM.zip › Figure 3/3B/source data Calnexin for LYSET time series 20230531_125659-04_Ch_Chemi.tif]

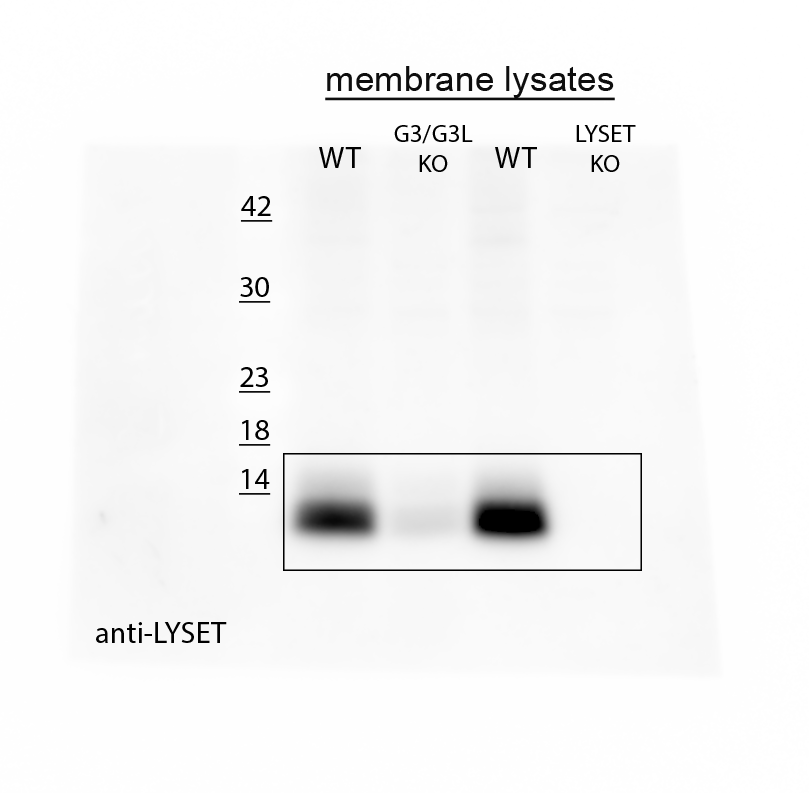

Supplement: Supplementary file 6 — Source data Fig. 3 [file 44318_2024_305_MOESM6_ESM.zip › Figure 3/3B/source data LYSET short exp. time series 20230531_124027-04_Ch_Chemi.tif]

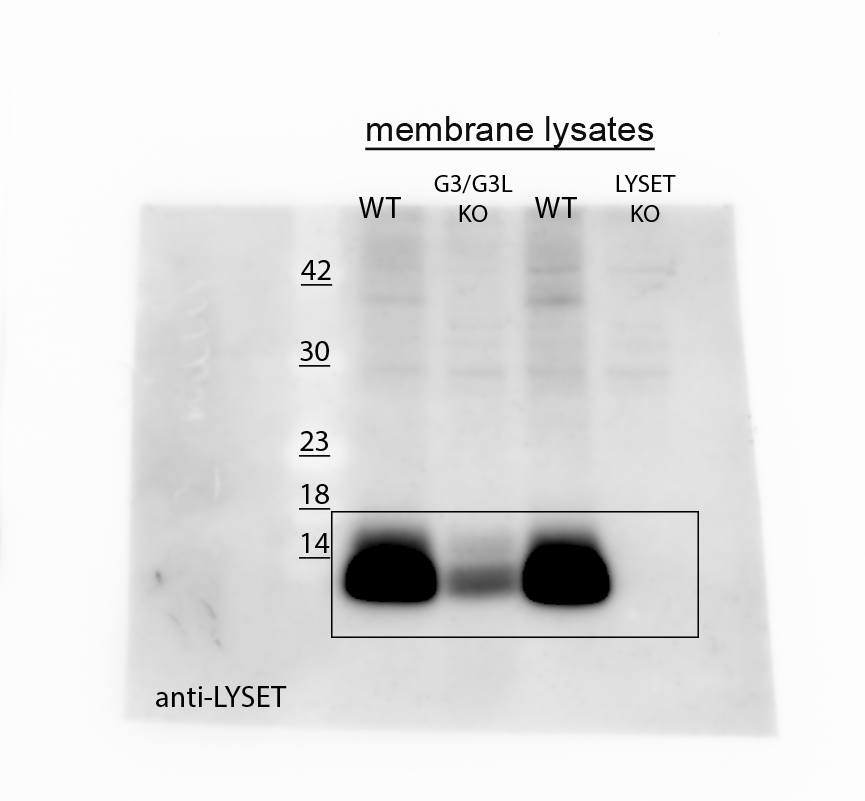

Supplement: Supplementary file 6 — Source data Fig. 3 [file 44318_2024_305_MOESM6_ESM.zip › Figure 3/3B/source data LYSET long exp. time series 20230531_124027-20_Ch_Chemi.tif]

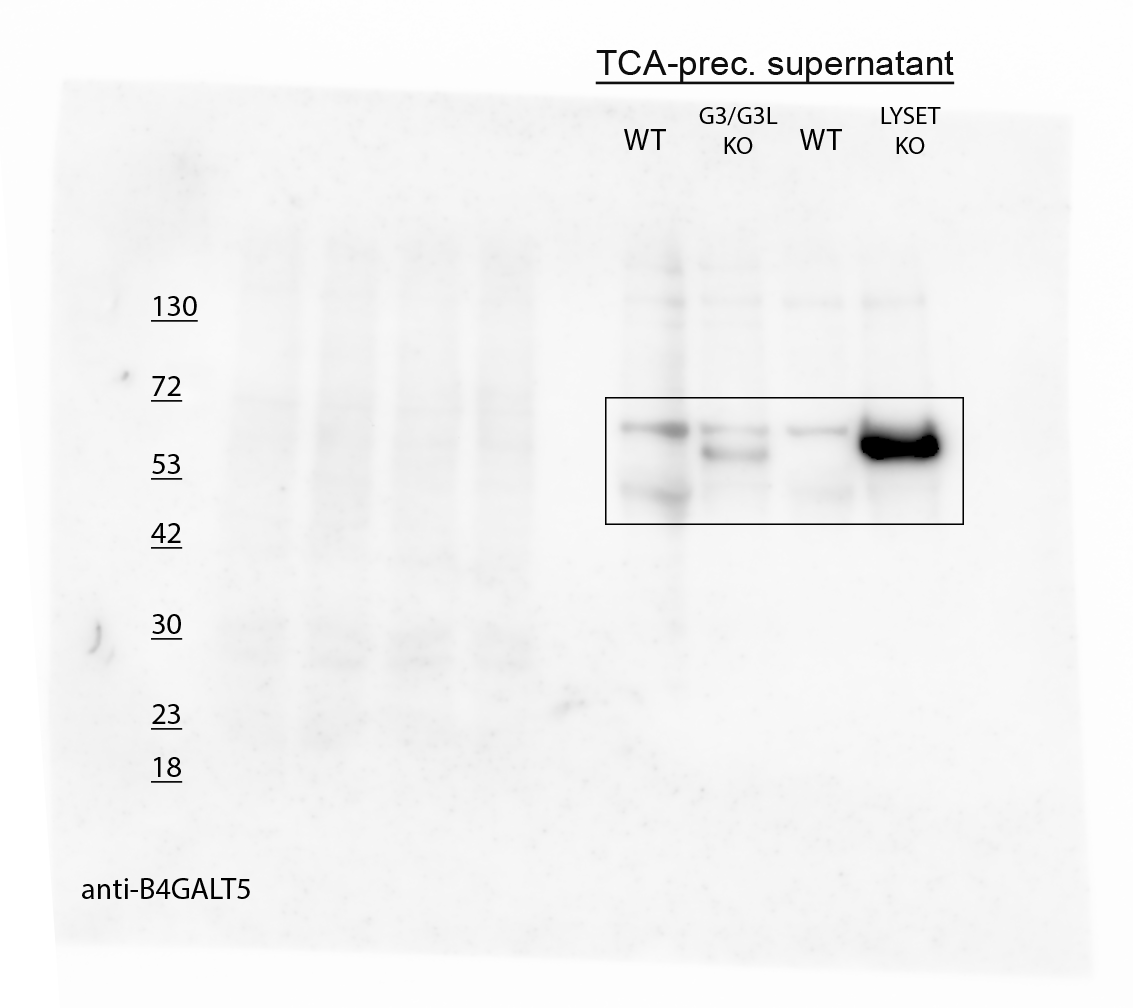

Supplement: Supplementary file 6 — Source data Fig. 3 [file 44318_2024_305_MOESM6_ESM.zip › Figure 3/3B/source data B4GALT5 time series 20230530_132032-15_Ch_Chemi.tif]

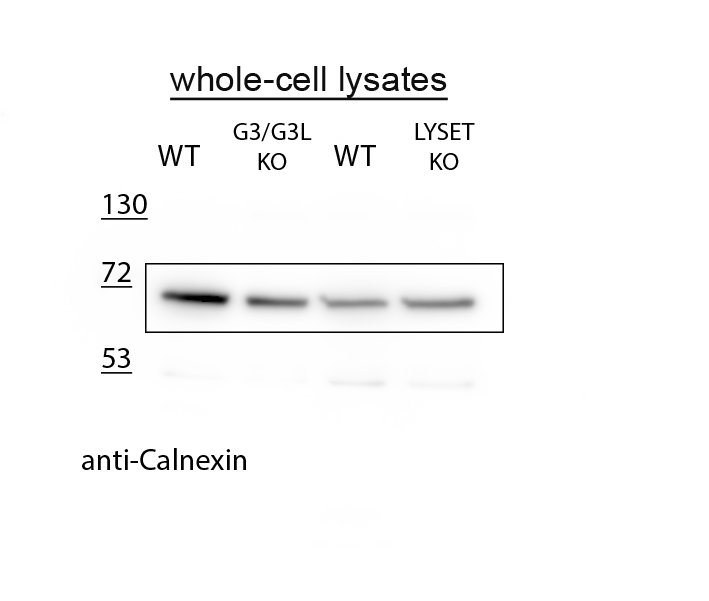

Supplement: Supplementary file 6 — Source data Fig. 3 [file 44318_2024_305_MOESM6_ESM.zip › Figure 3/3B/source data Calnexin for GOLPH3, GOLPH3(L) time series 20230531_130237-03_Ch_Chemi.tif]

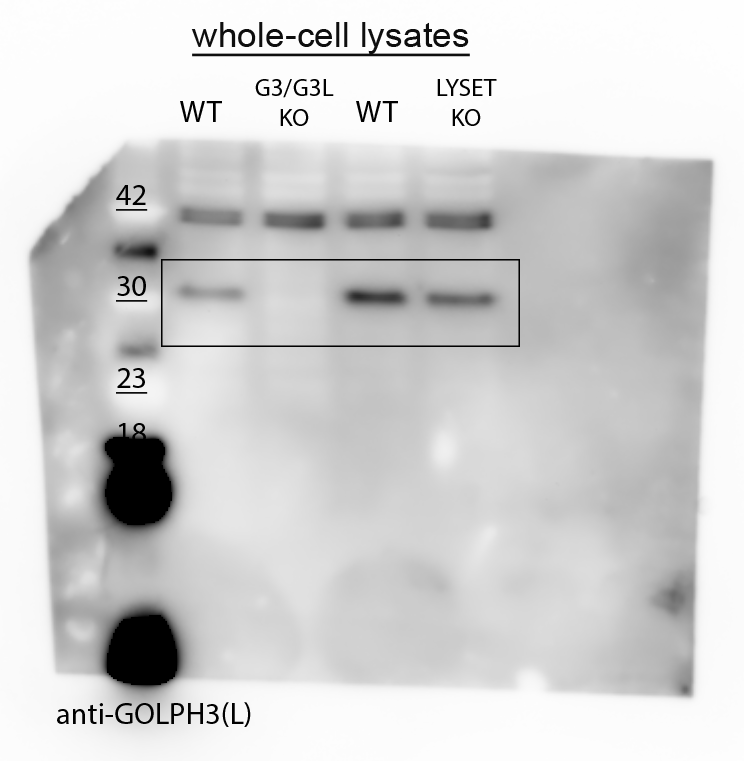

Supplement: Supplementary file 6 — Source data Fig. 3 [file 44318_2024_305_MOESM6_ESM.zip › Figure 3/3B/source data GOLPH3(L) 20231202_161951-10_Ch_Chemi.tif]

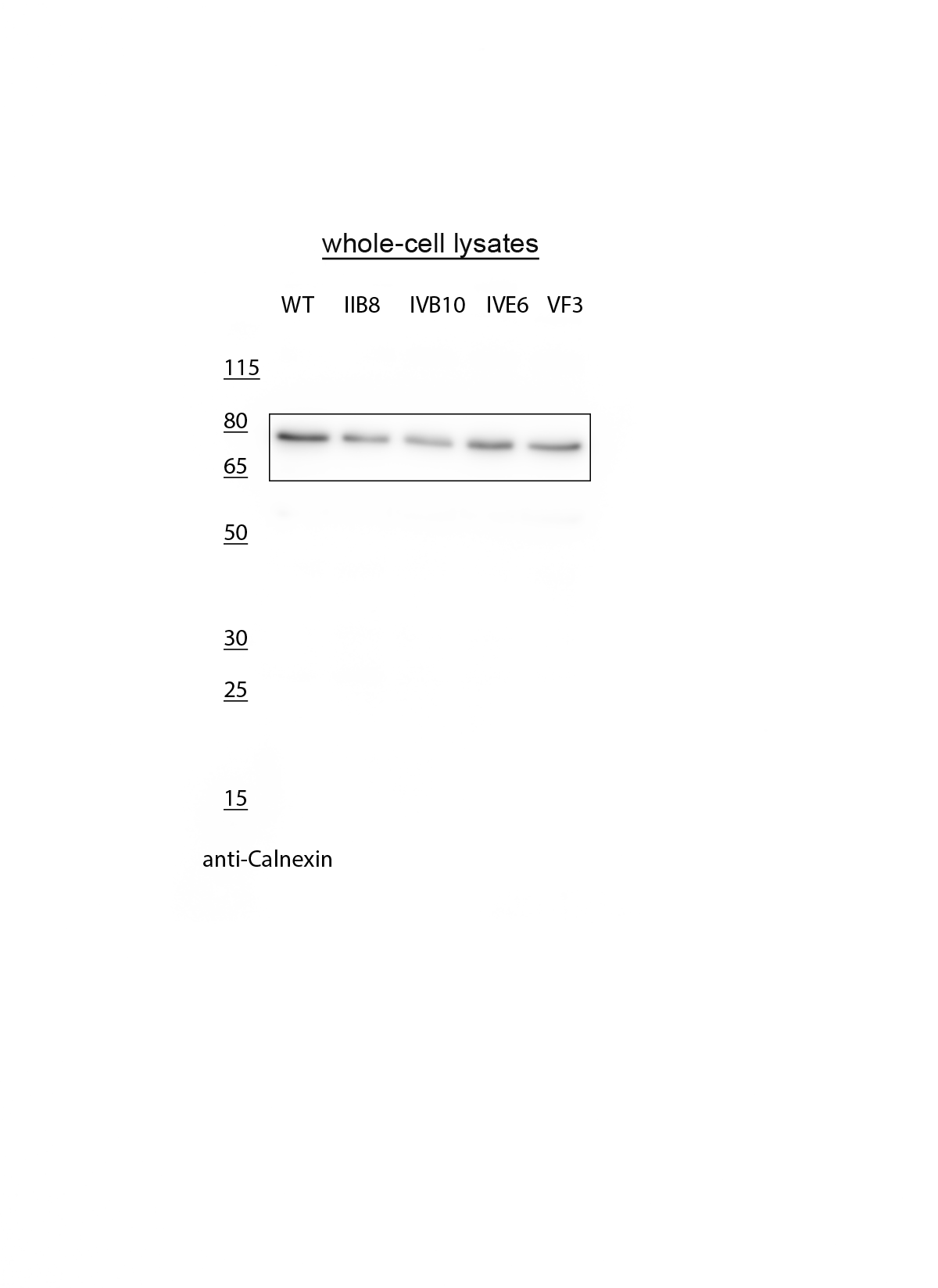

Supplement: Supplementary file 6 — Source data Fig. 3 [file 44318_2024_305_MOESM6_ESM.zip › Figure 3/3C/source data Calnexin for GRASP55 time series 20231009_173845-02_Ch_Chemi.tif]

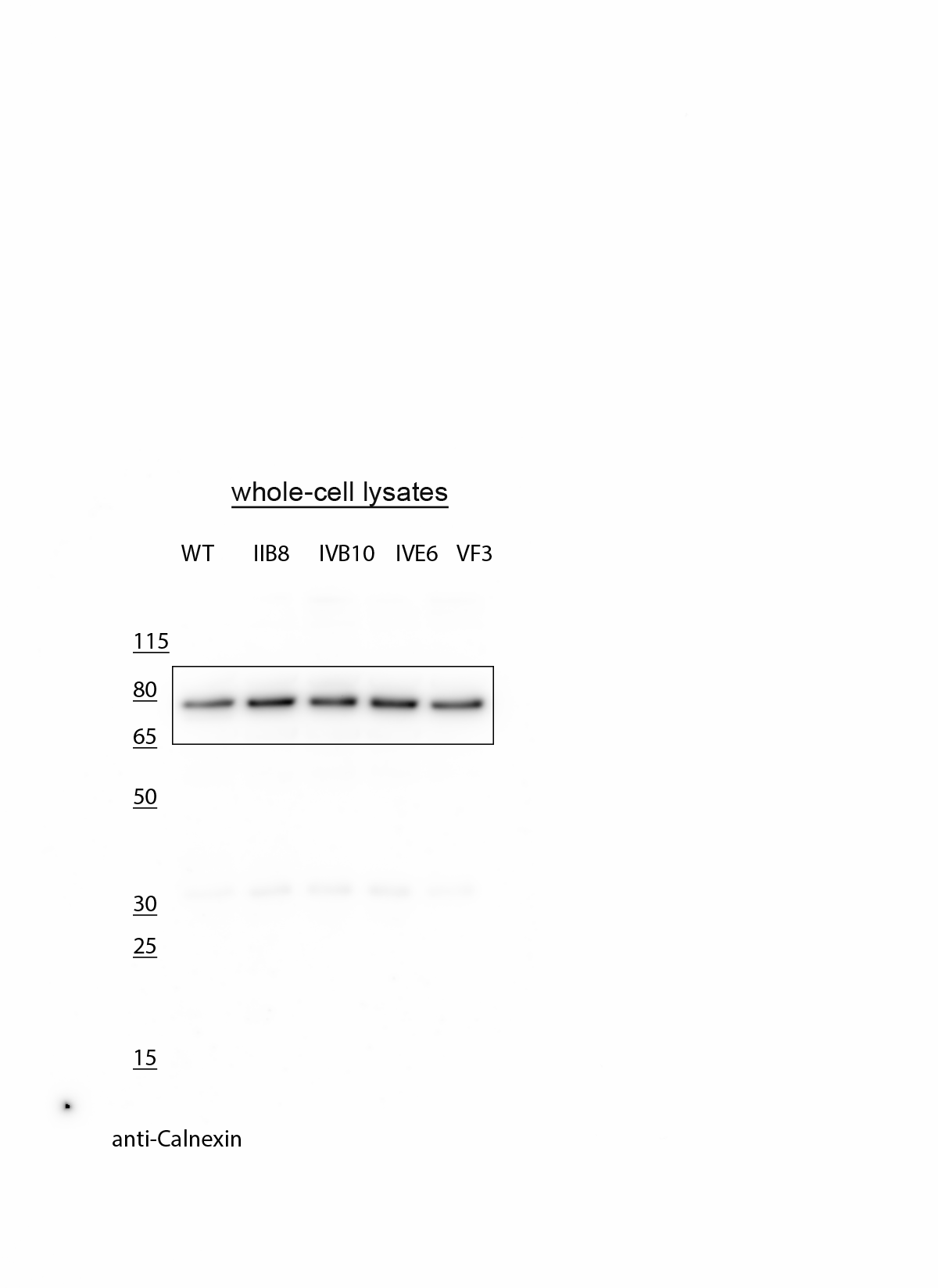

Supplement: Supplementary file 6 — Source data Fig. 3 [file 44318_2024_305_MOESM6_ESM.zip › Figure 3/3C/source data Calnexin for COG3, GOLGA3 20240323_172528-05_Ch_Chemi.tif]

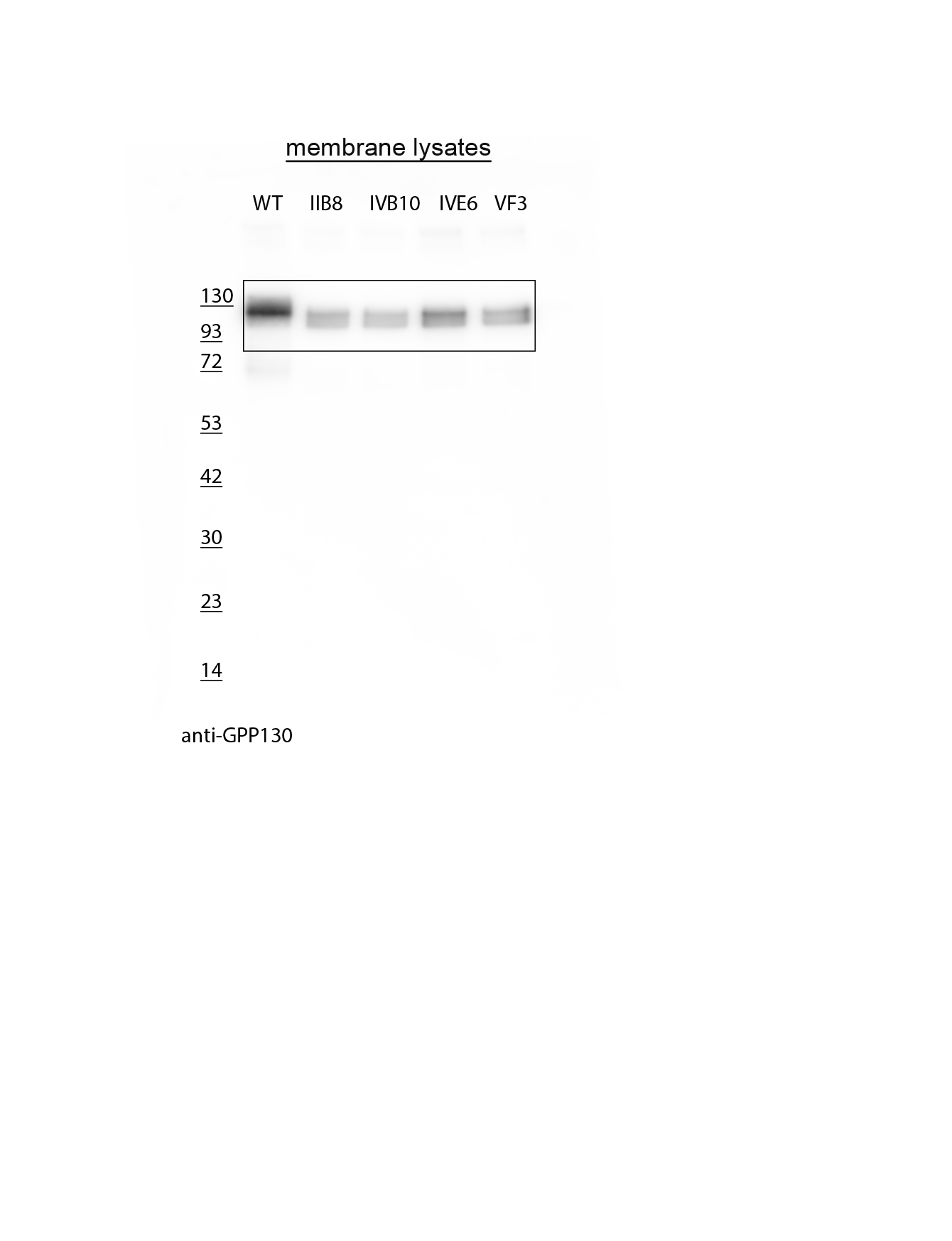

Supplement: Supplementary file 6 — Source data Fig. 3 [file 44318_2024_305_MOESM6_ESM.zip › Figure 3/3C/source data GPP130 weak ECL time series 20230818_135119-01_Ch_Chemi.tif]

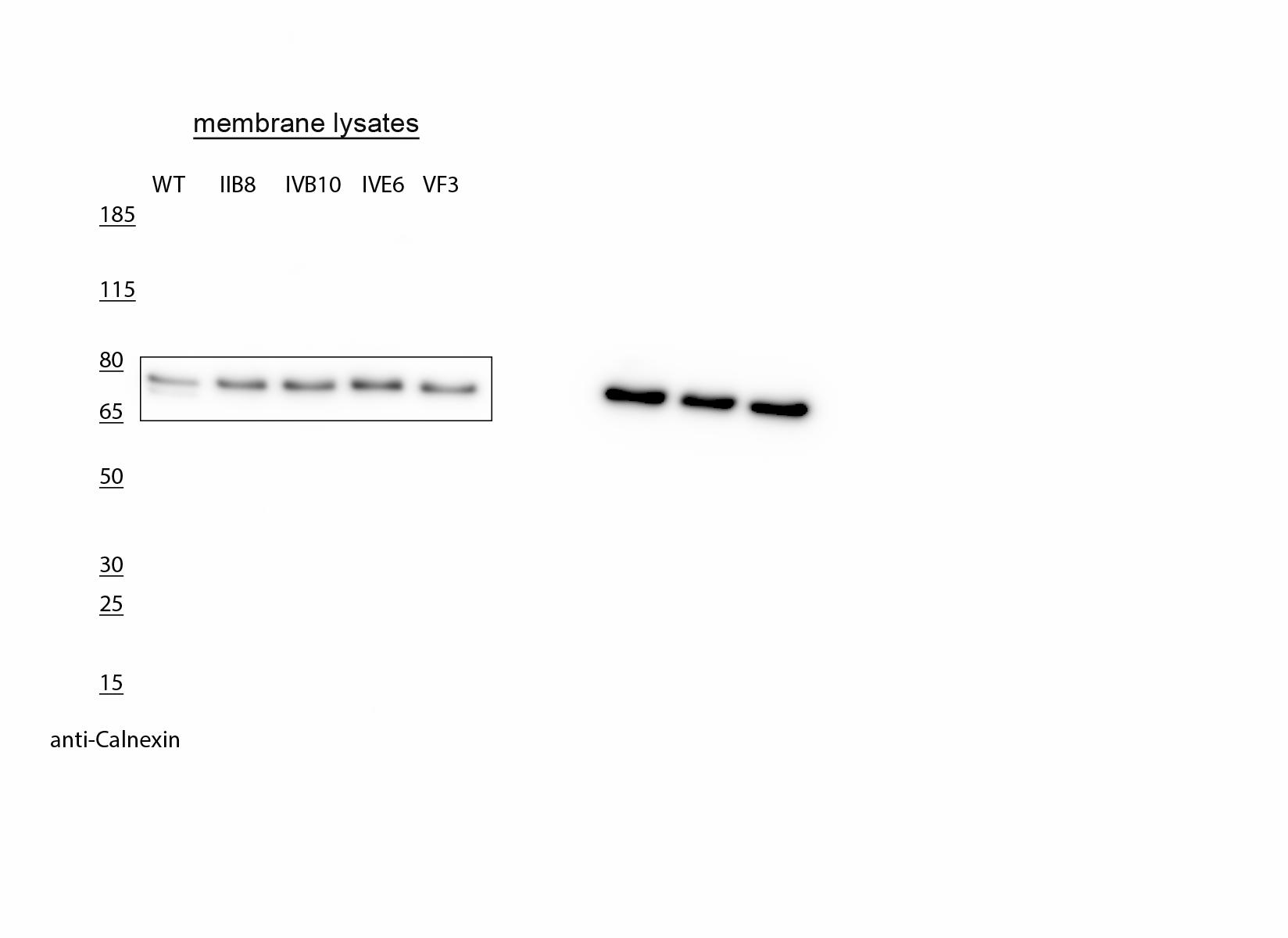

Supplement: Supplementary file 6 — Source data Fig. 3 [file 44318_2024_305_MOESM6_ESM.zip › Figure 3/3C/source data Calnexin for GNPTAB 20240322_161957-06_Ch_Chemi.tif]

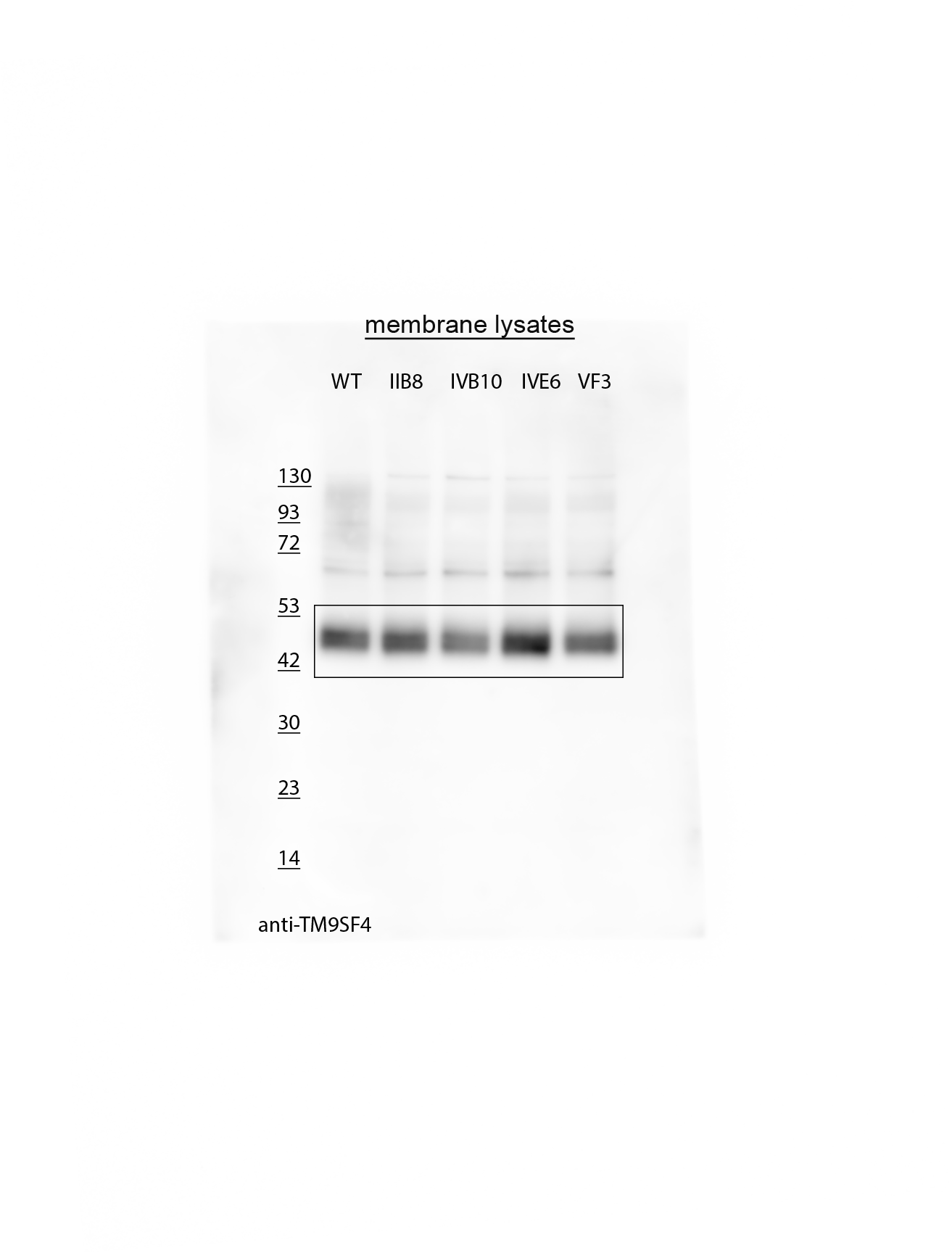

Supplement: Supplementary file 6 — Source data Fig. 3 [file 44318_2024_305_MOESM6_ESM.zip › Figure 3/3C/source data TM9SF4 time series 20230901_121102-04_Ch_Chemi.tif]

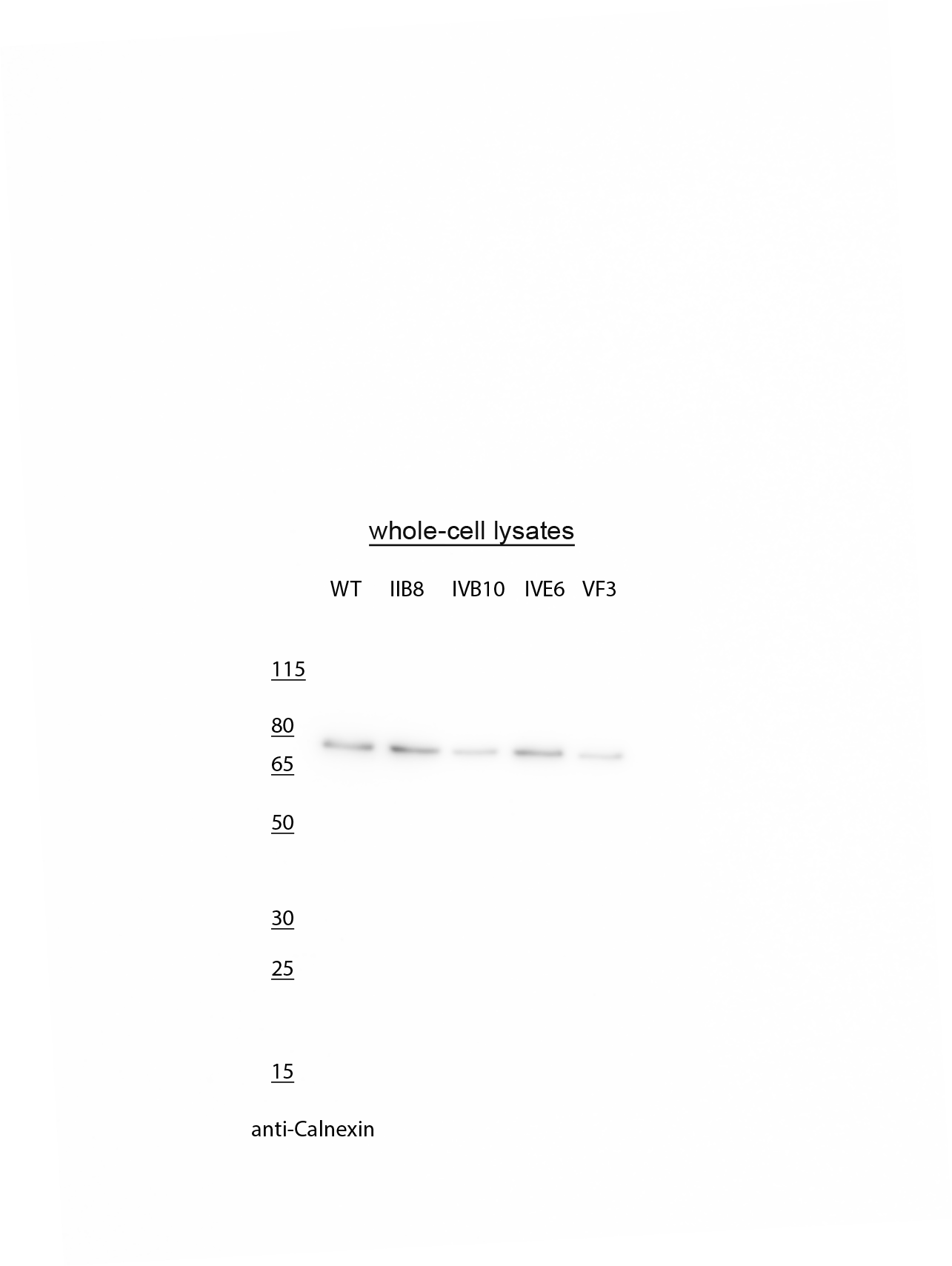

Supplement: Supplementary file 6 — Source data Fig. 3 [file 44318_2024_305_MOESM6_ESM.zip › Figure 3/3C/source data Calnexin for GOLPH3, GM130 20240323_141703-11_Ch_Chemi.tif]

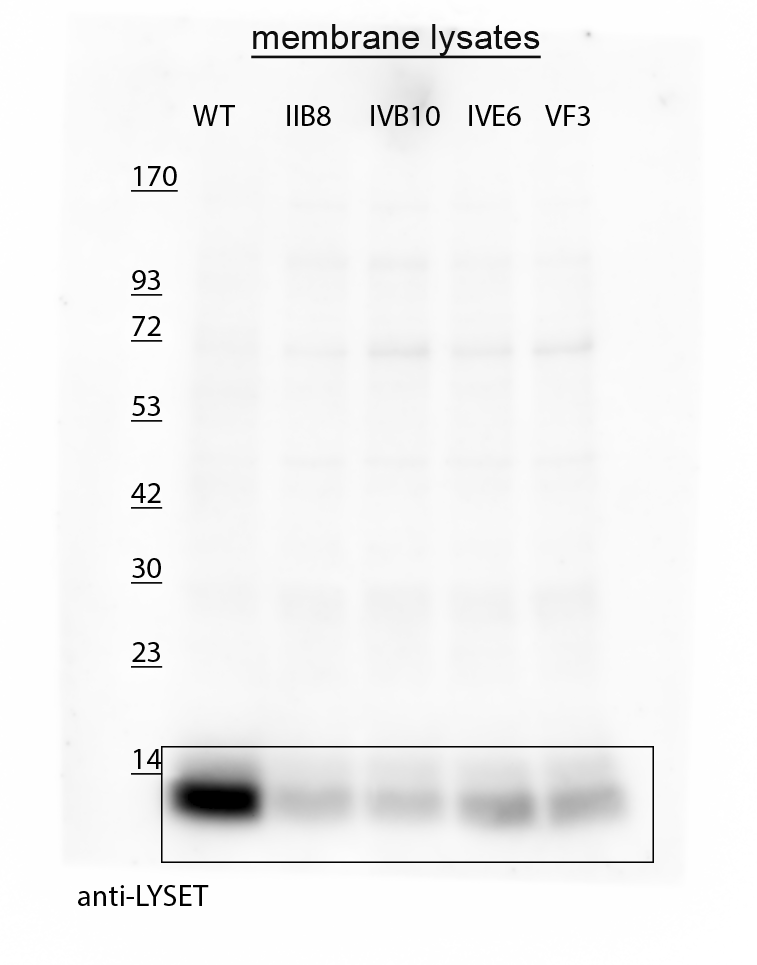

Supplement: Supplementary file 6 — Source data Fig. 3 [file 44318_2024_305_MOESM6_ESM.zip › Figure 3/3C/source data LYSET time series 20230818_131401-04_Ch_Chemi.tif]

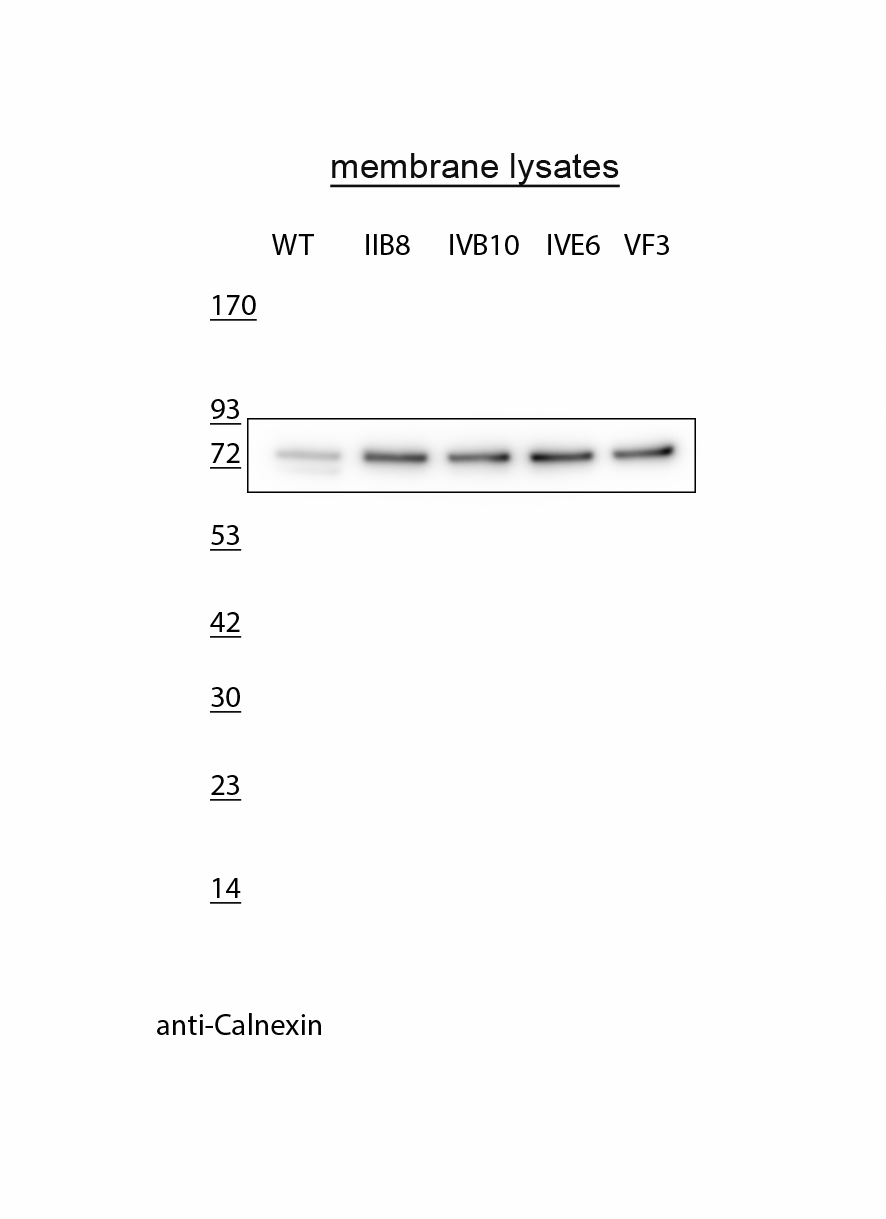

Supplement: Supplementary file 6 — Source data Fig. 3 [file 44318_2024_305_MOESM6_ESM.zip › Figure 3/3C/source data Calnexin for LYSET, GS27 20240322_162603-06_Ch_Chemi.tif]

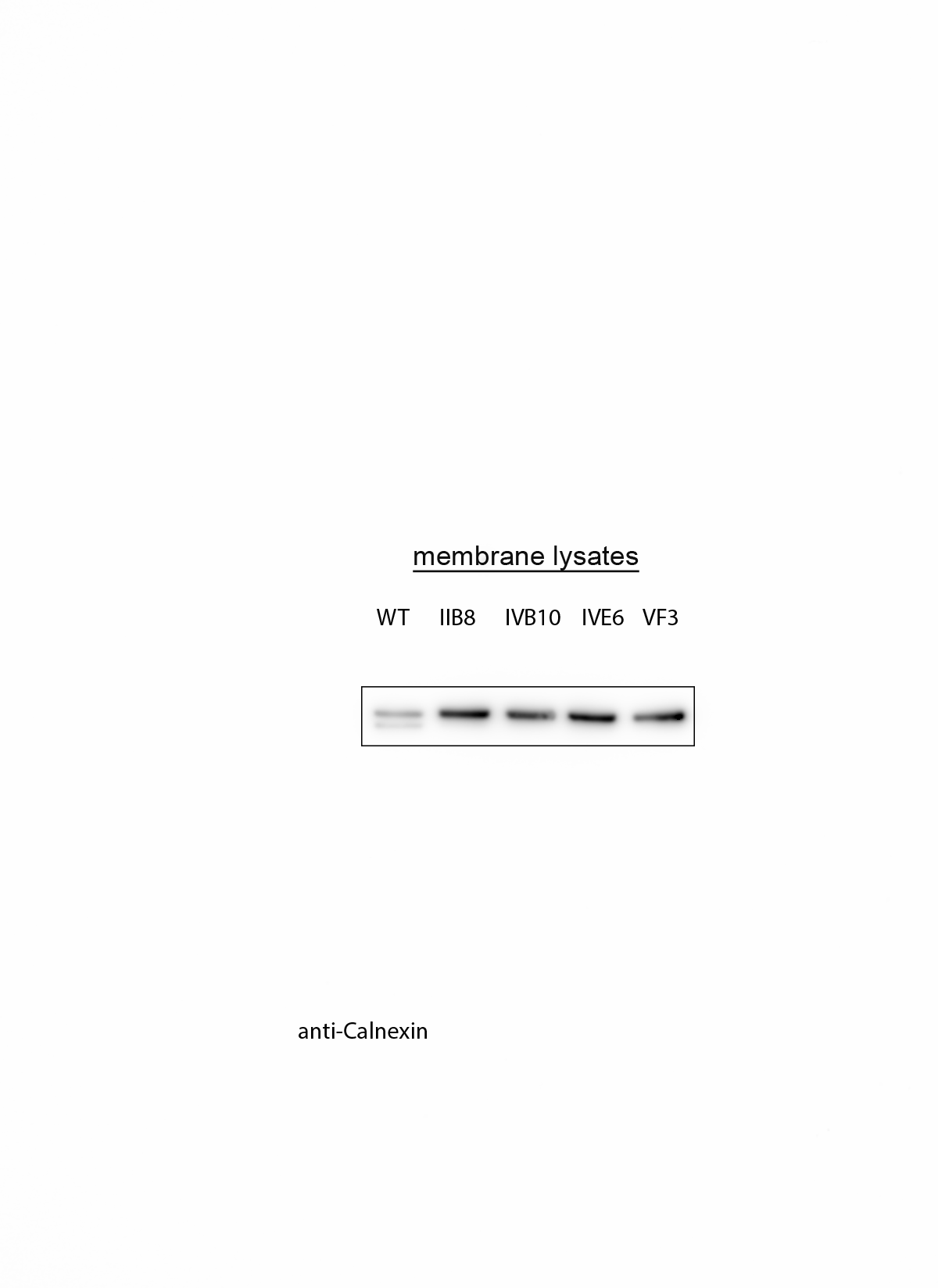

Supplement: Supplementary file 6 — Source data Fig. 3 [file 44318_2024_305_MOESM6_ESM.zip › Figure 3/3C/source data Calnexin for TM9SF2 20240320_124358-08_Ch_Chemi.tif]

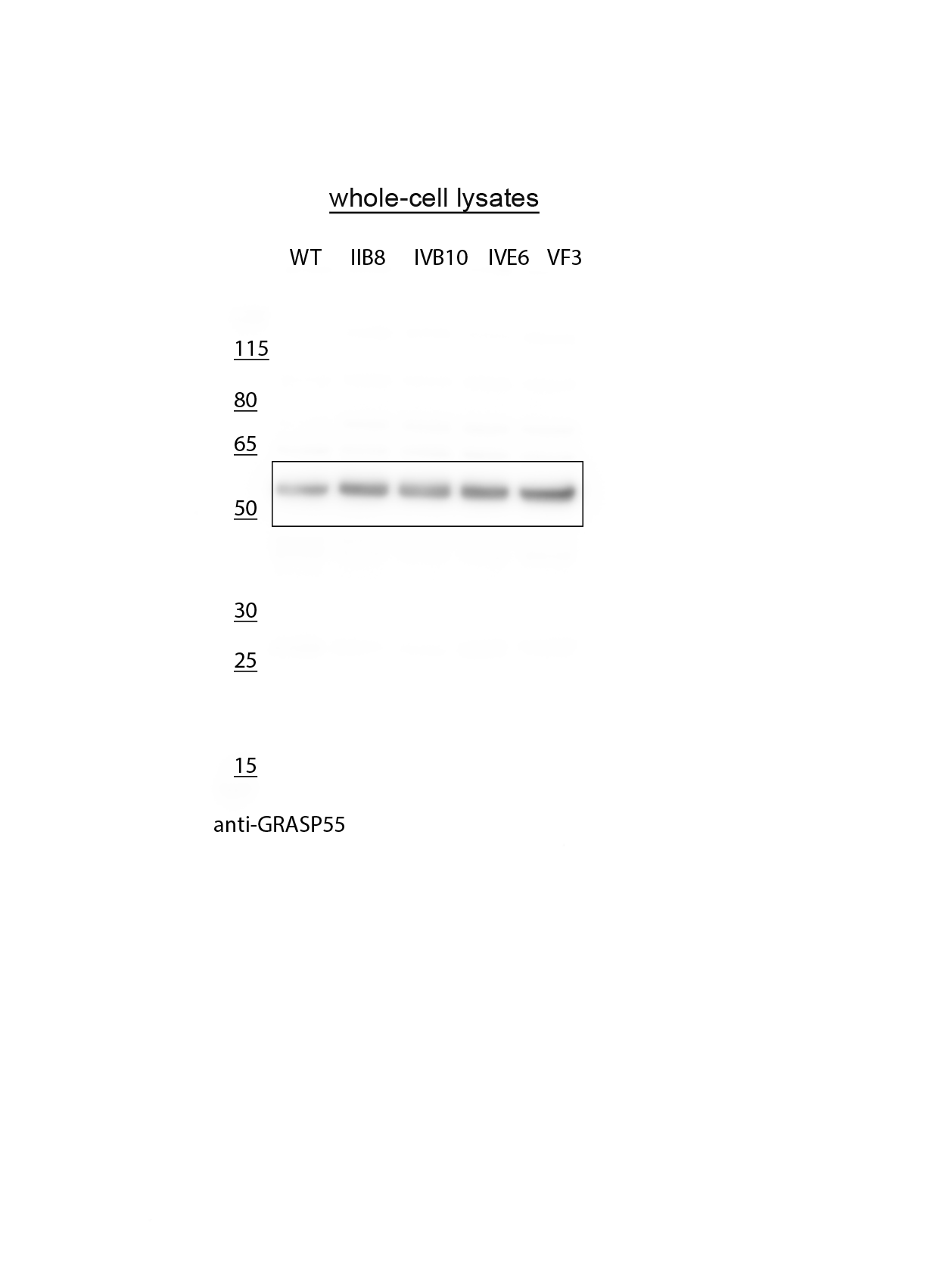

Supplement: Supplementary file 6 — Source data Fig. 3 [file 44318_2024_305_MOESM6_ESM.zip › Figure 3/3C/source data GRASP55 time series 20231005_171720-01_Ch_Chemi.tif]

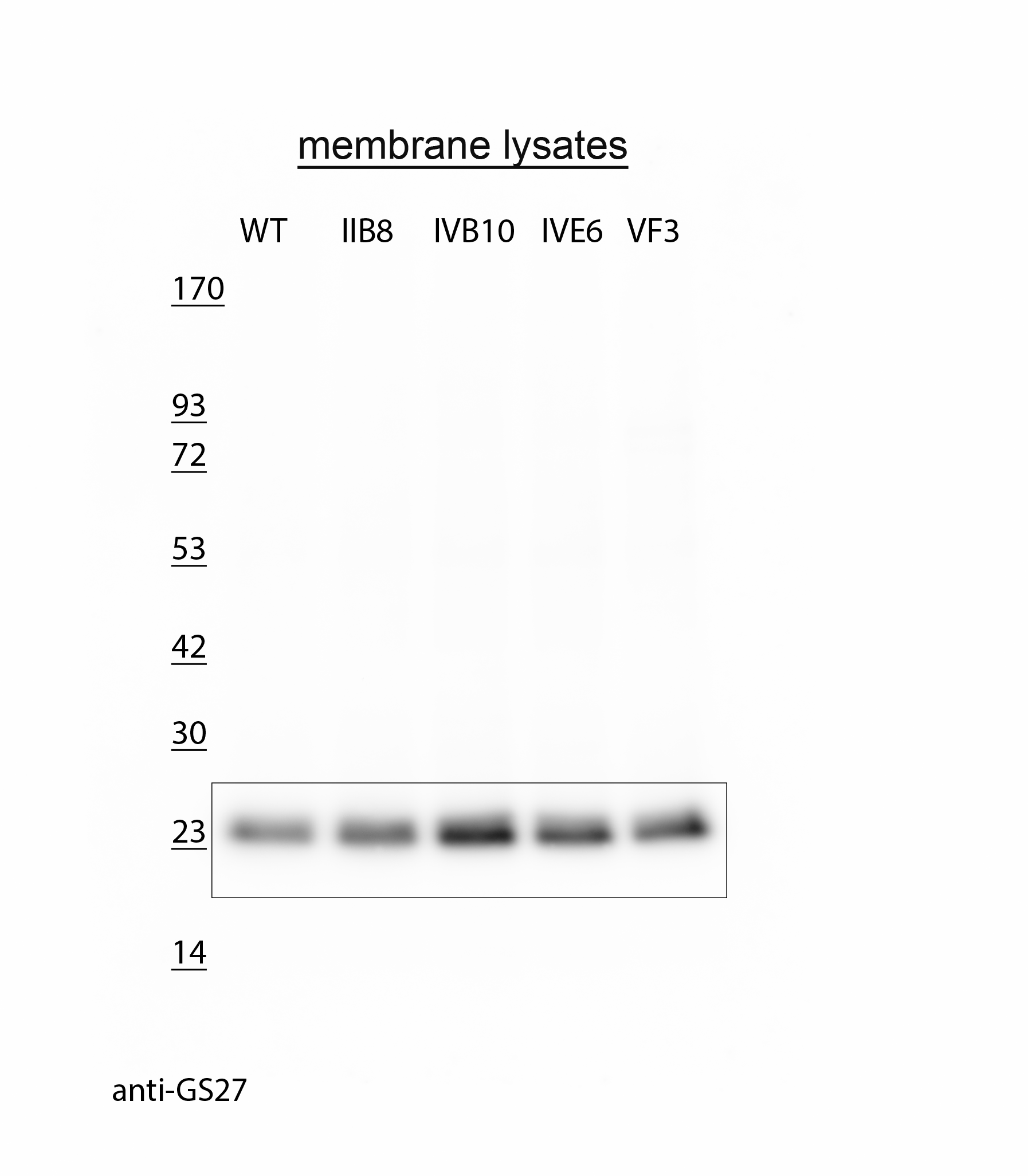

Supplement: Supplementary file 6 — Source data Fig. 3 [file 44318_2024_305_MOESM6_ESM.zip › Figure 3/3C/sourced data GS27 time series 20230901_114928-02_Ch_Chemi.tif]

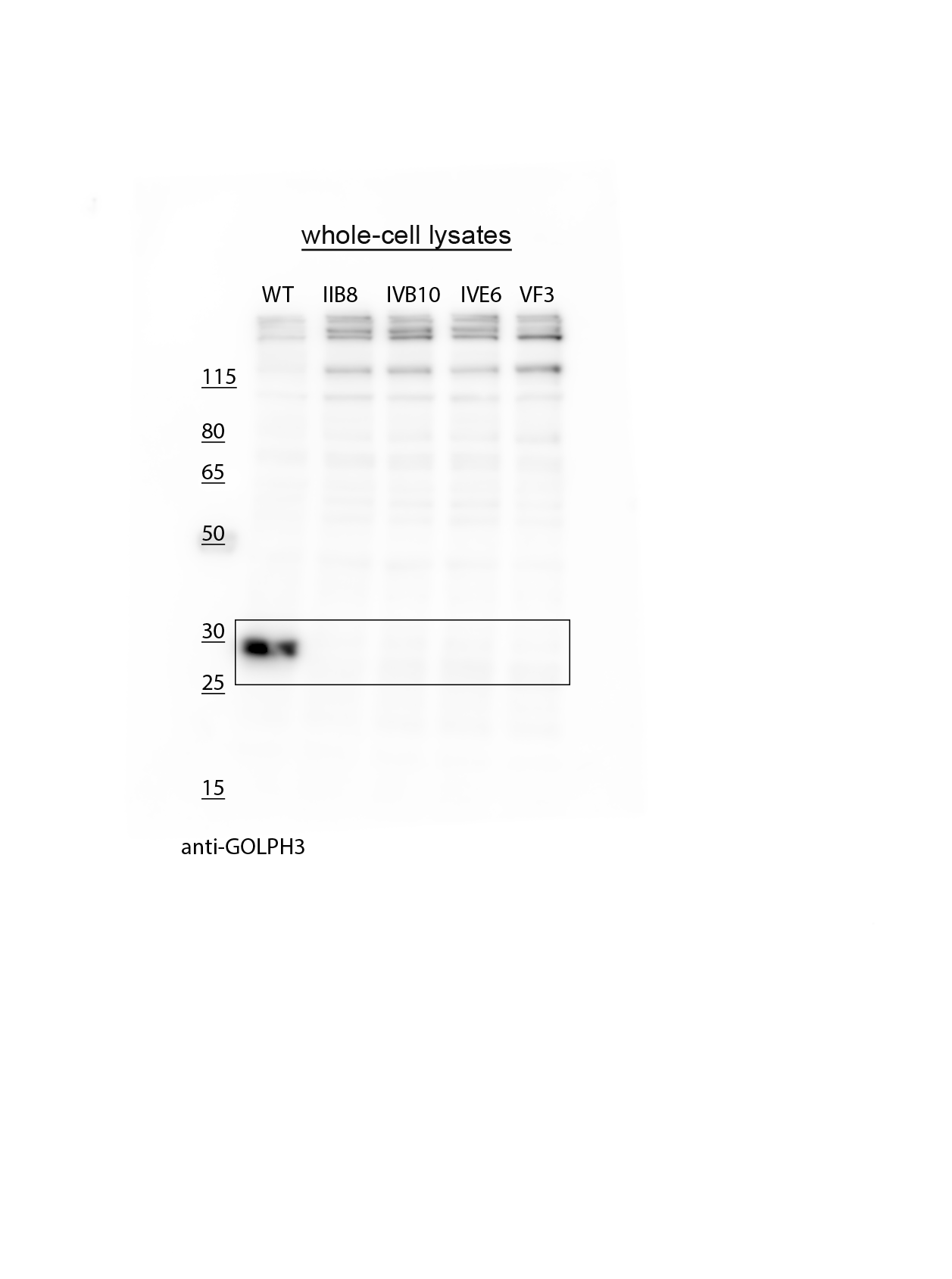

Supplement: Supplementary file 6 — Source data Fig. 3 [file 44318_2024_305_MOESM6_ESM.zip › Figure 3/3C/source data G3 (PTG) time series 20231006_144650-01_Ch_Chemi.tif]

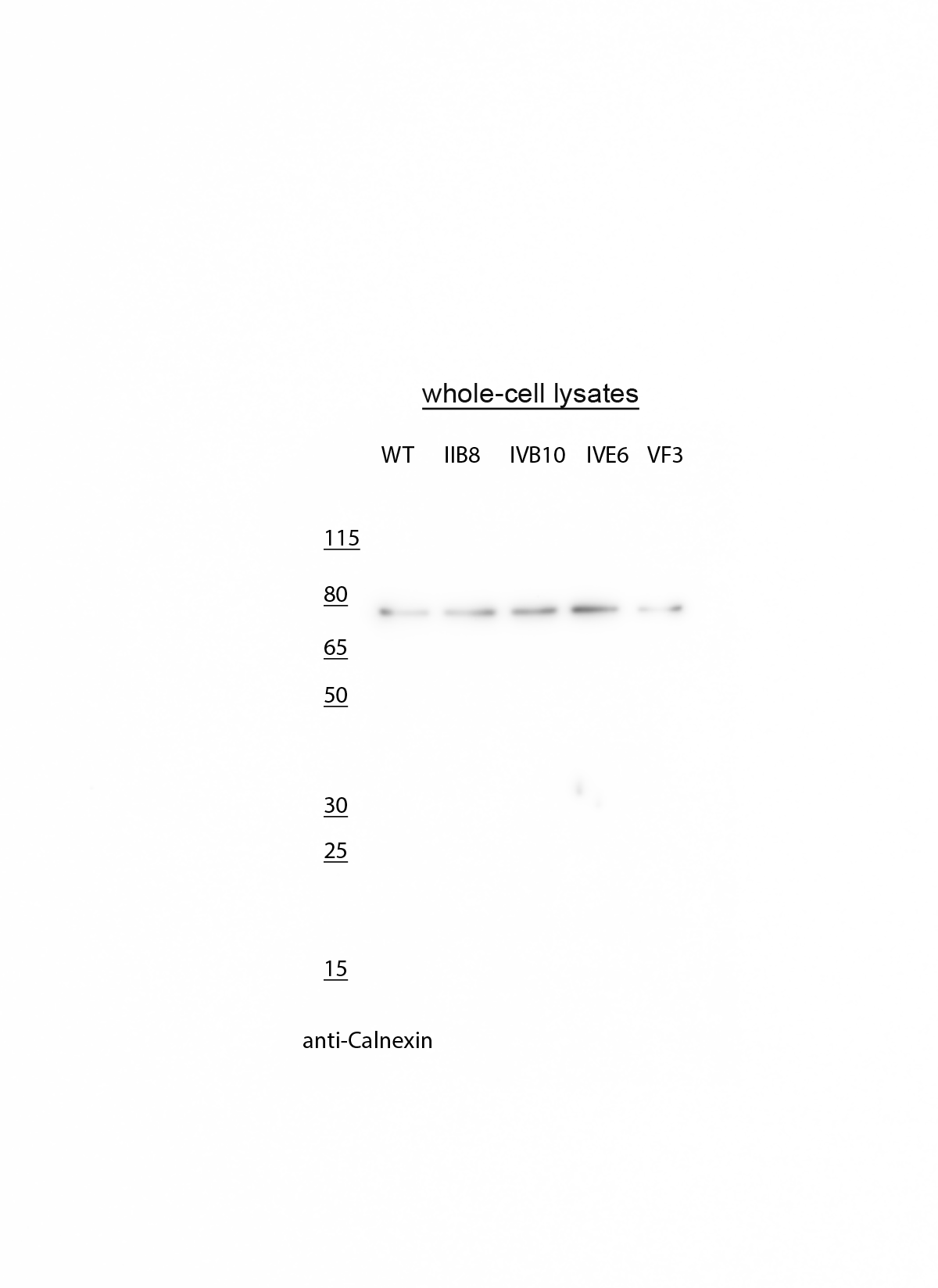

Supplement: Supplementary file 6 — Source data Fig. 3 [file 44318_2024_305_MOESM6_ESM.zip › Figure 3/3C/source data Calnexin for GRASP65 20240323_140545-08_Ch_Chemi.tif]

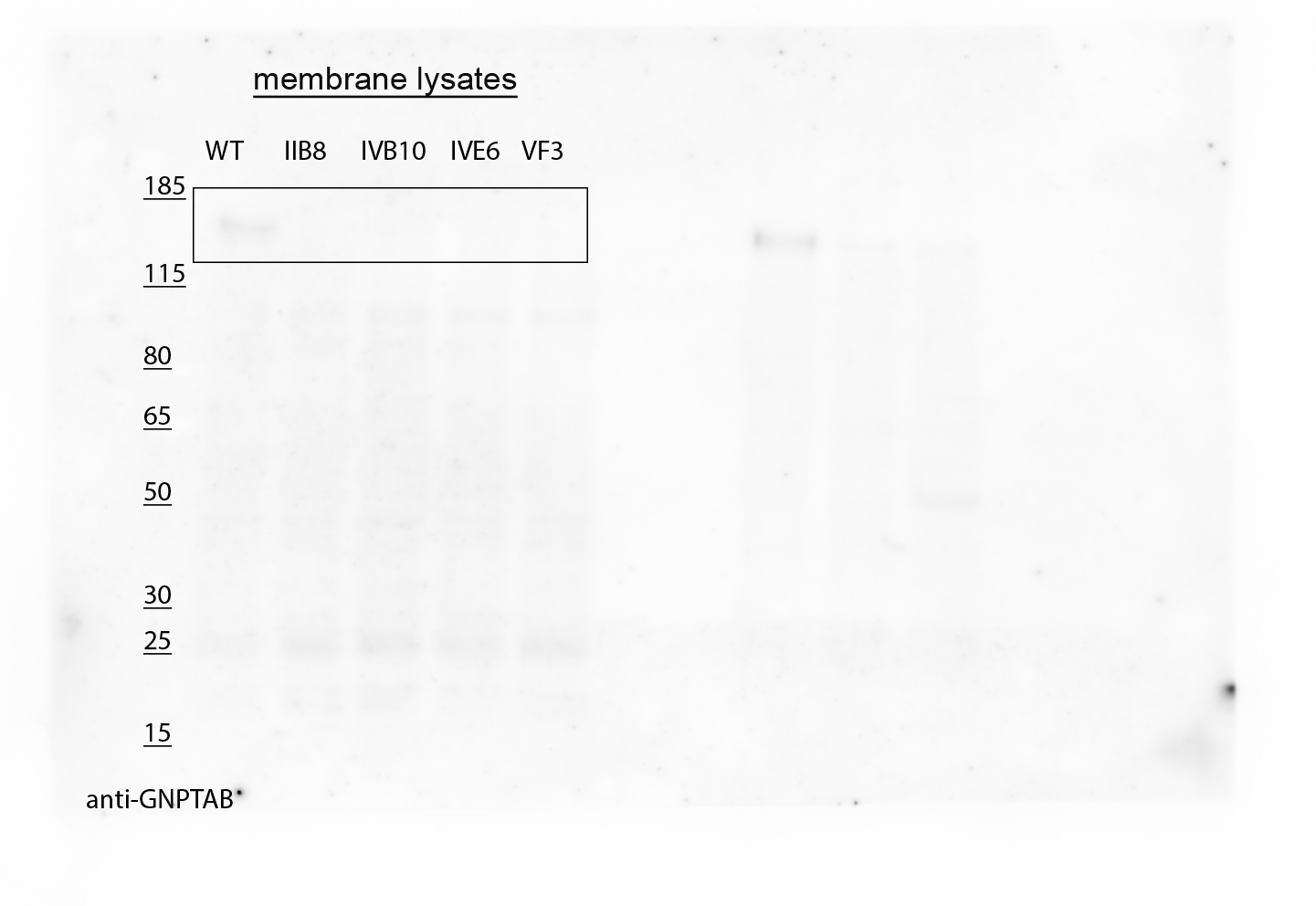

Supplement: Supplementary file 6 — Source data Fig. 3 [file 44318_2024_305_MOESM6_ESM.zip › Figure 3/3C/source data GNPTAB time series 20230919_161806-20_Ch_Chemi.tif]

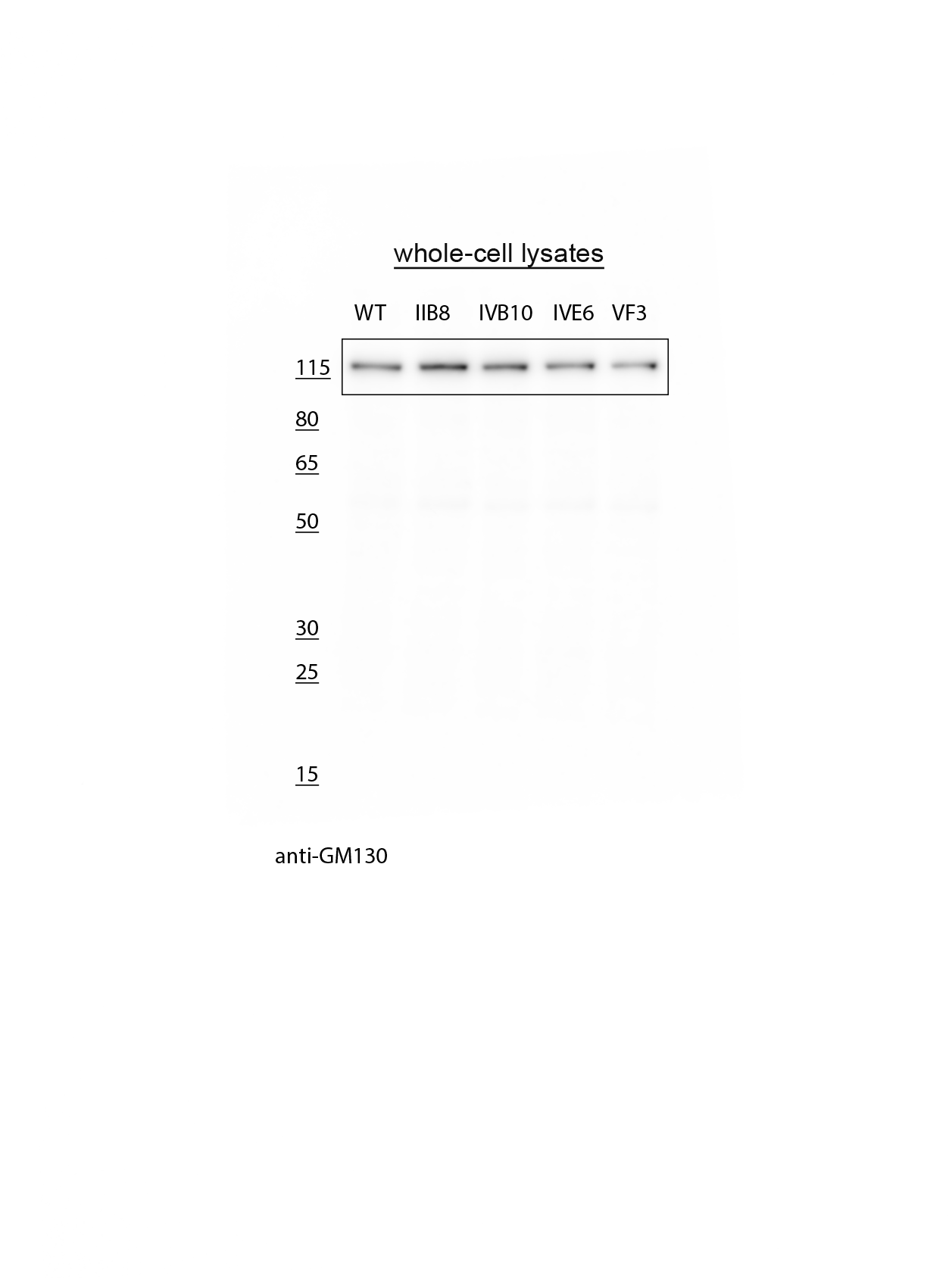

Supplement: Supplementary file 6 — Source data Fig. 3 [file 44318_2024_305_MOESM6_ESM.zip › Figure 3/3C/source data GM130 2.0 time series 20231011_170224-02_Ch_Chemi.tif]

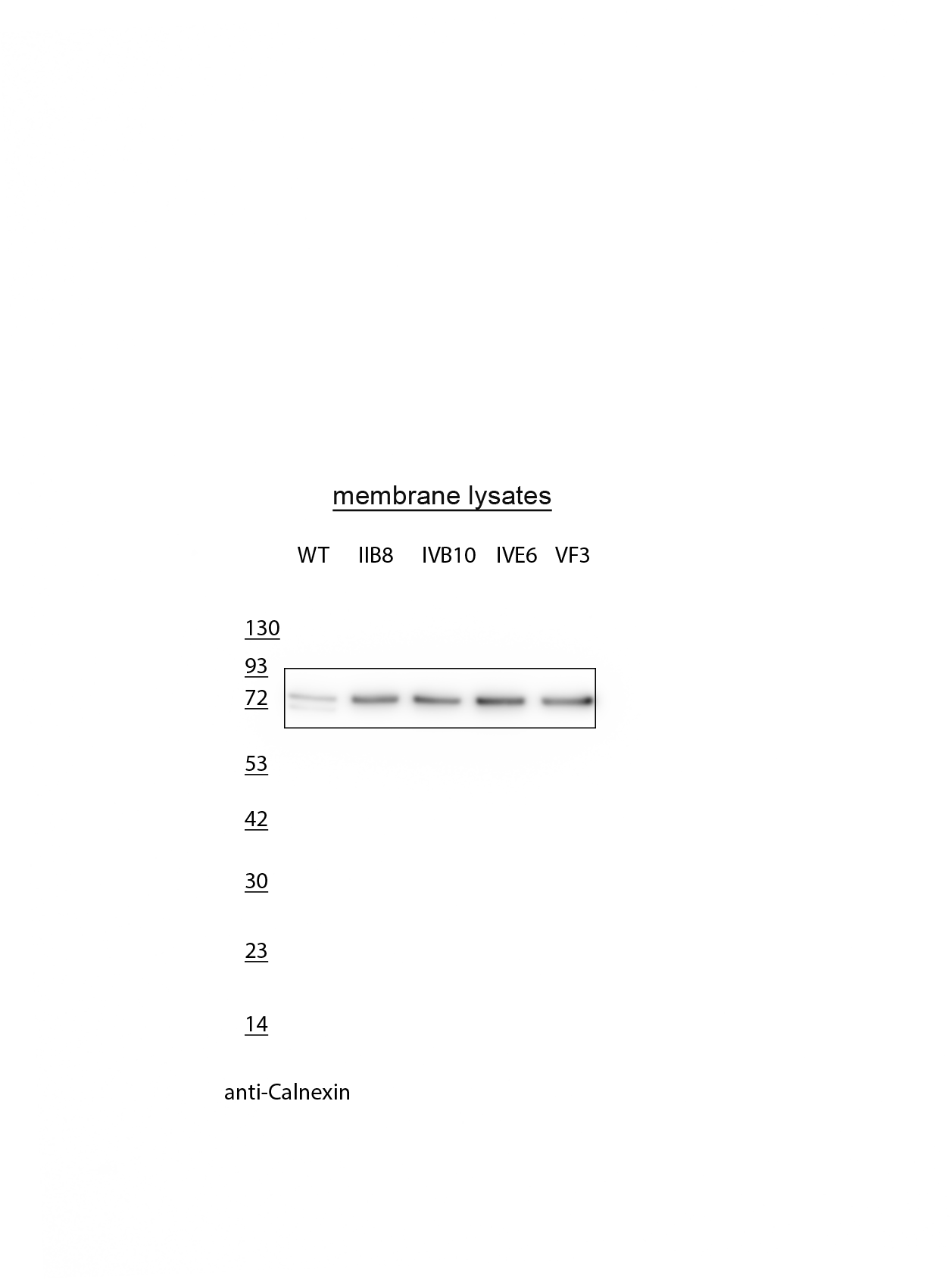

Supplement: Supplementary file 6 — Source data Fig. 3 [file 44318_2024_305_MOESM6_ESM.zip › Figure 3/3C/source data Calnexin for GPP130, TM9SF4 20240322_163943-03_Ch_Chemi.tif]

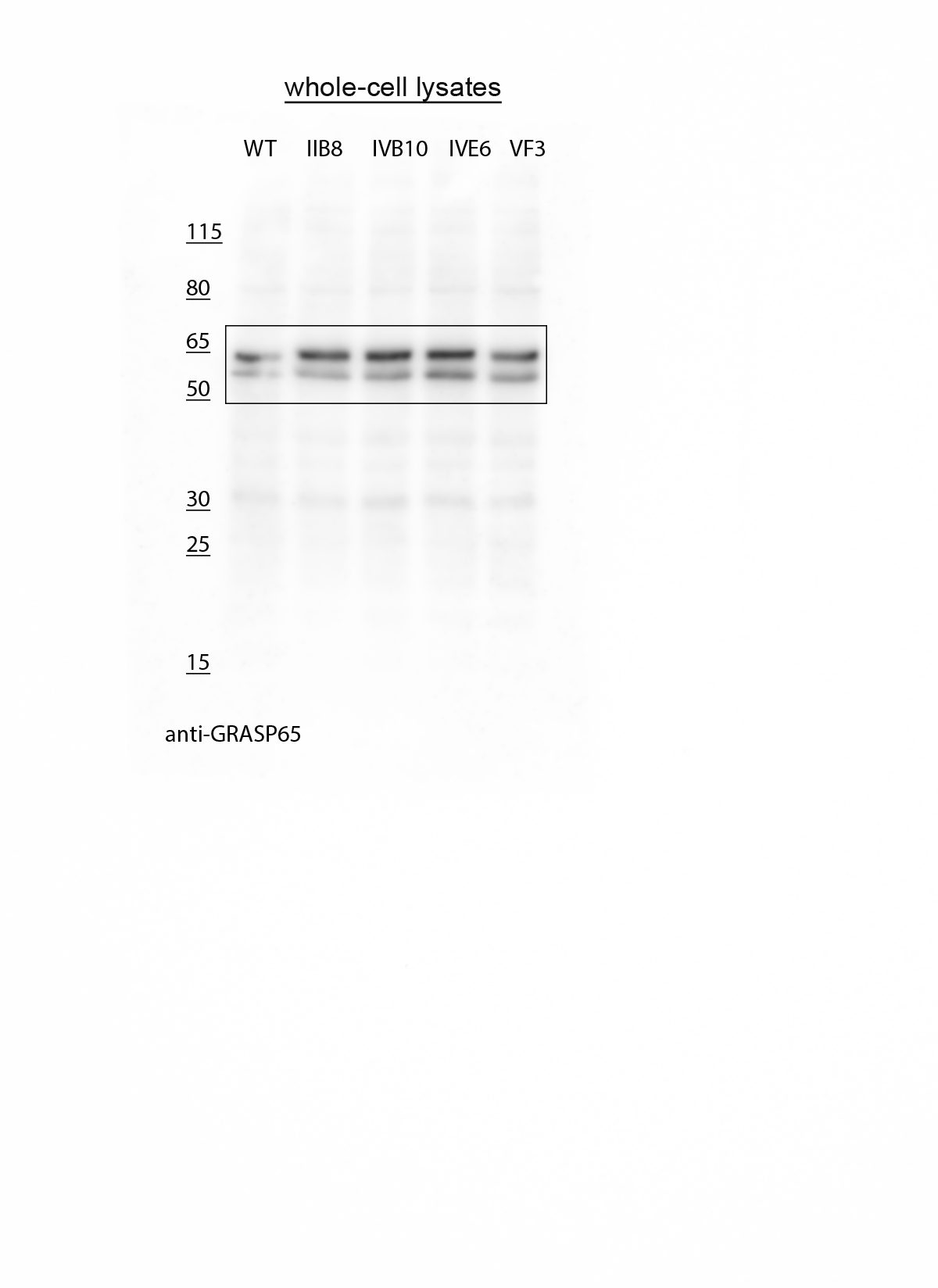

Supplement: Supplementary file 6 — Source data Fig. 3 [file 44318_2024_305_MOESM6_ESM.zip › Figure 3/3C/source data GRASP65 time series 20231005_173718-06_Ch_Chemi.tif]

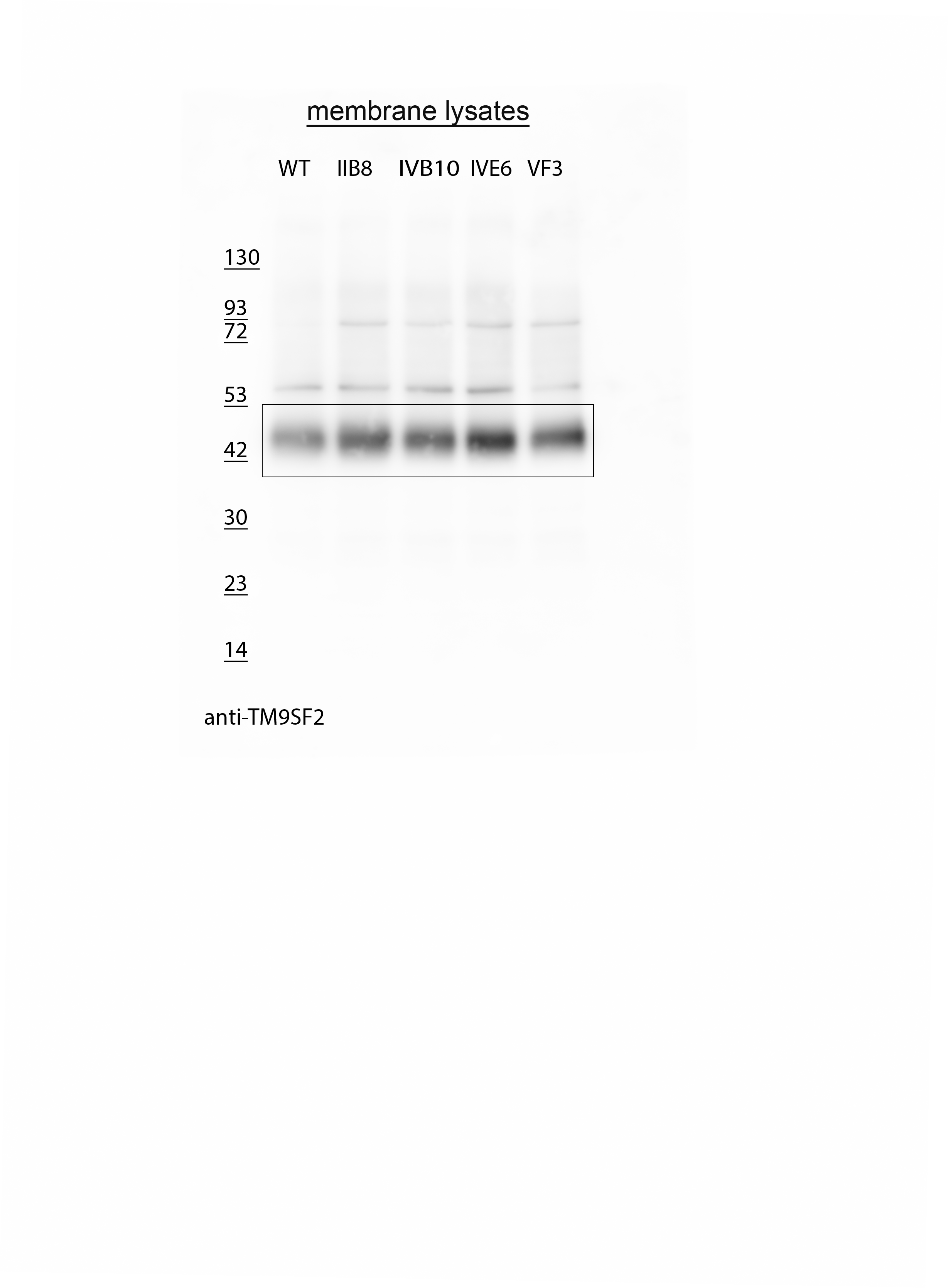

Supplement: Supplementary file 6 — Source data Fig. 3 [file 44318_2024_305_MOESM6_ESM.zip › Figure 3/3C/source data TM9SF2 time series 20230818_132251-01_Ch_Chemi.tif]

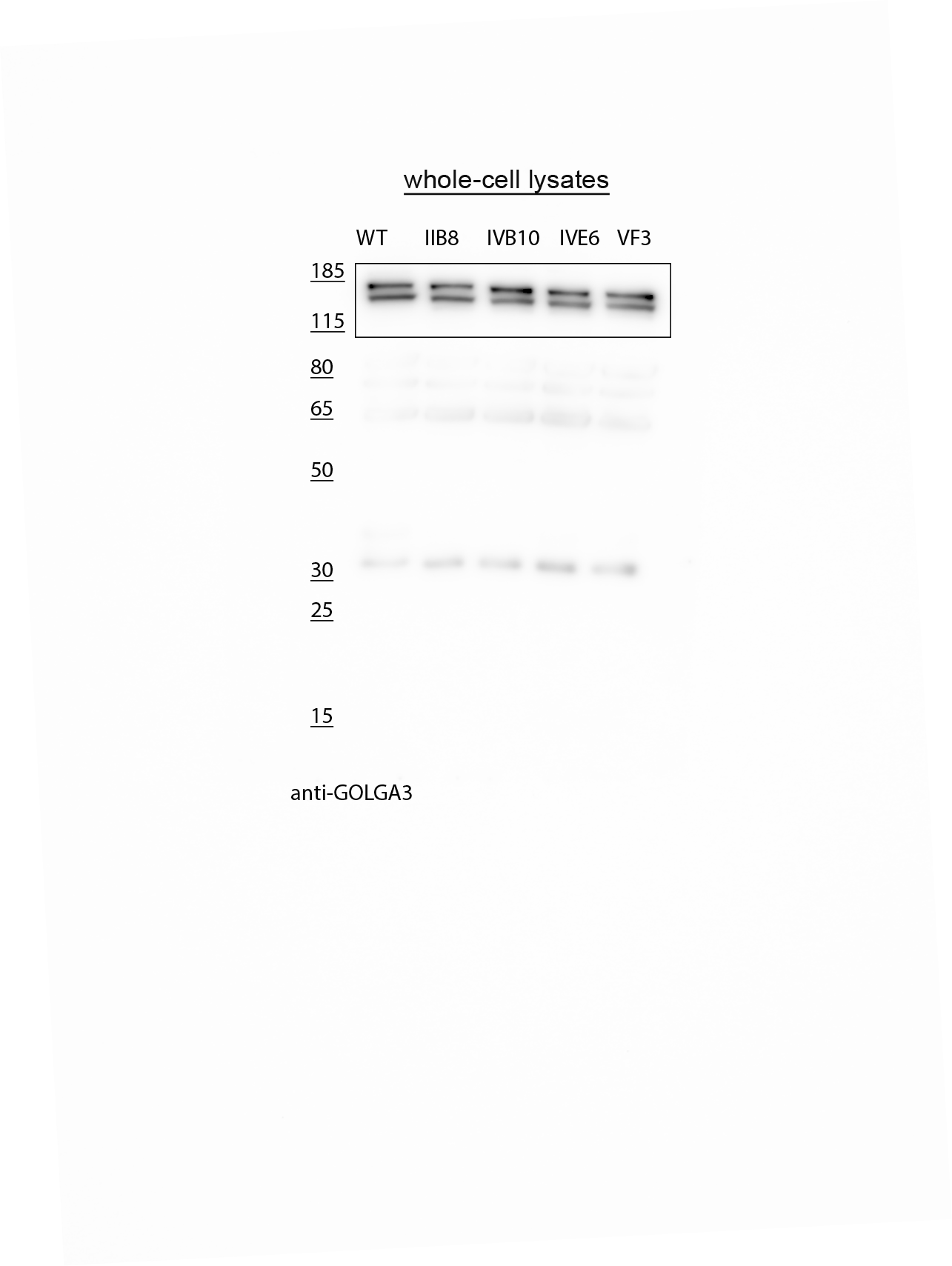

Supplement: Supplementary file 6 — Source data Fig. 3 [file 44318_2024_305_MOESM6_ESM.zip › Figure 3/3C/source data GOLGA3 time series 20231009_174315-14_Ch_Chemi.tif]

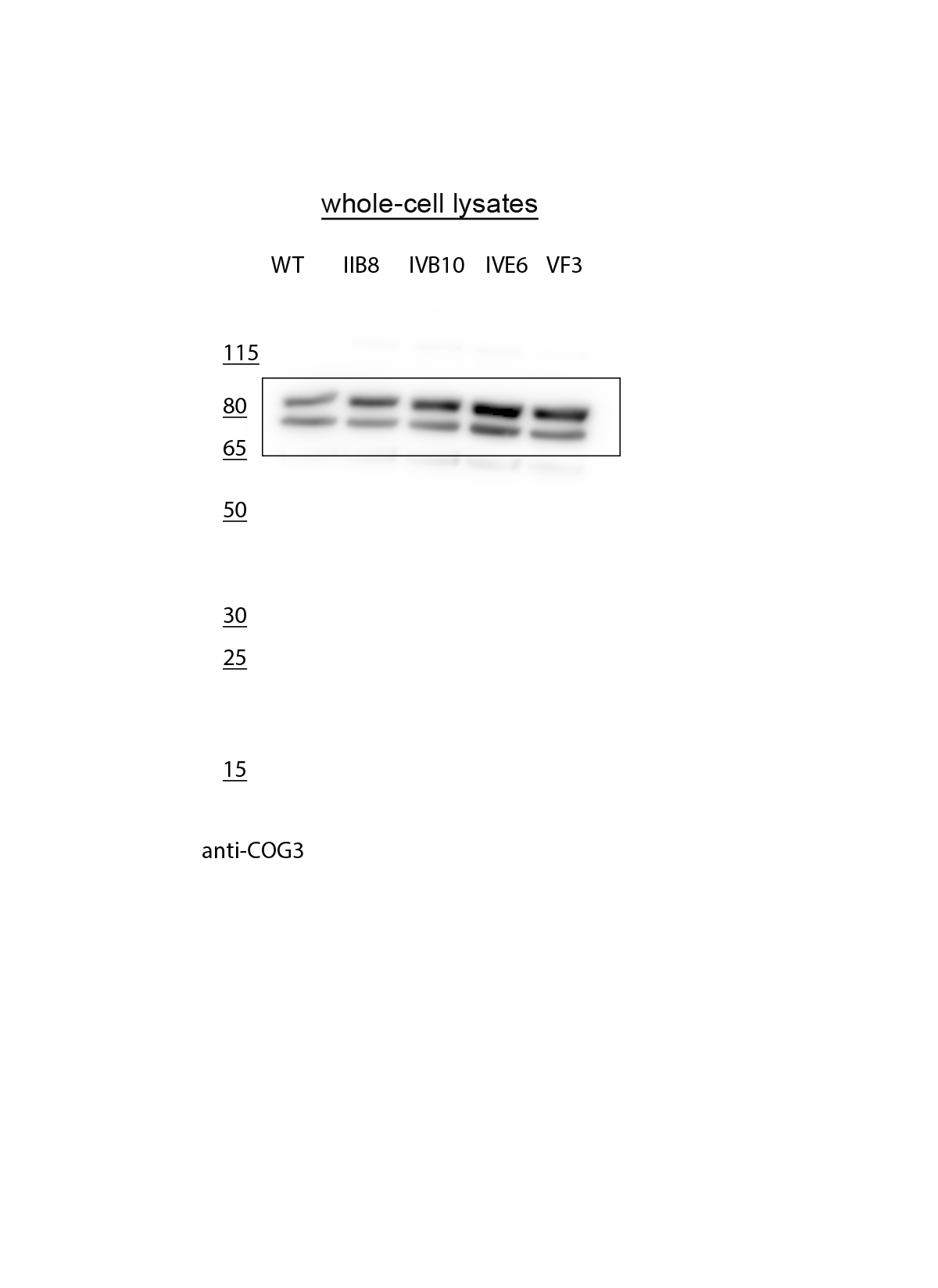

Supplement: Supplementary file 6 — Source data Fig. 3 [file 44318_2024_305_MOESM6_ESM.zip › Figure 3/3C/source data COG3 time series 20231005_170943-01_Ch_Chemi.tif]
