# Supplementary figures and images for: GOLPH3 and GOLPH3L maintain Golgi localization of LYSET and a functional mannose 6-phosphate transport pathway (part 2 of 4)
Source: EMBO J. 2024 Nov 25;43(24):6264–90. doi: 10.1038/s44318-024-00305-z (PMC11649813; doi:10.1038/s44318-024-00305-z)

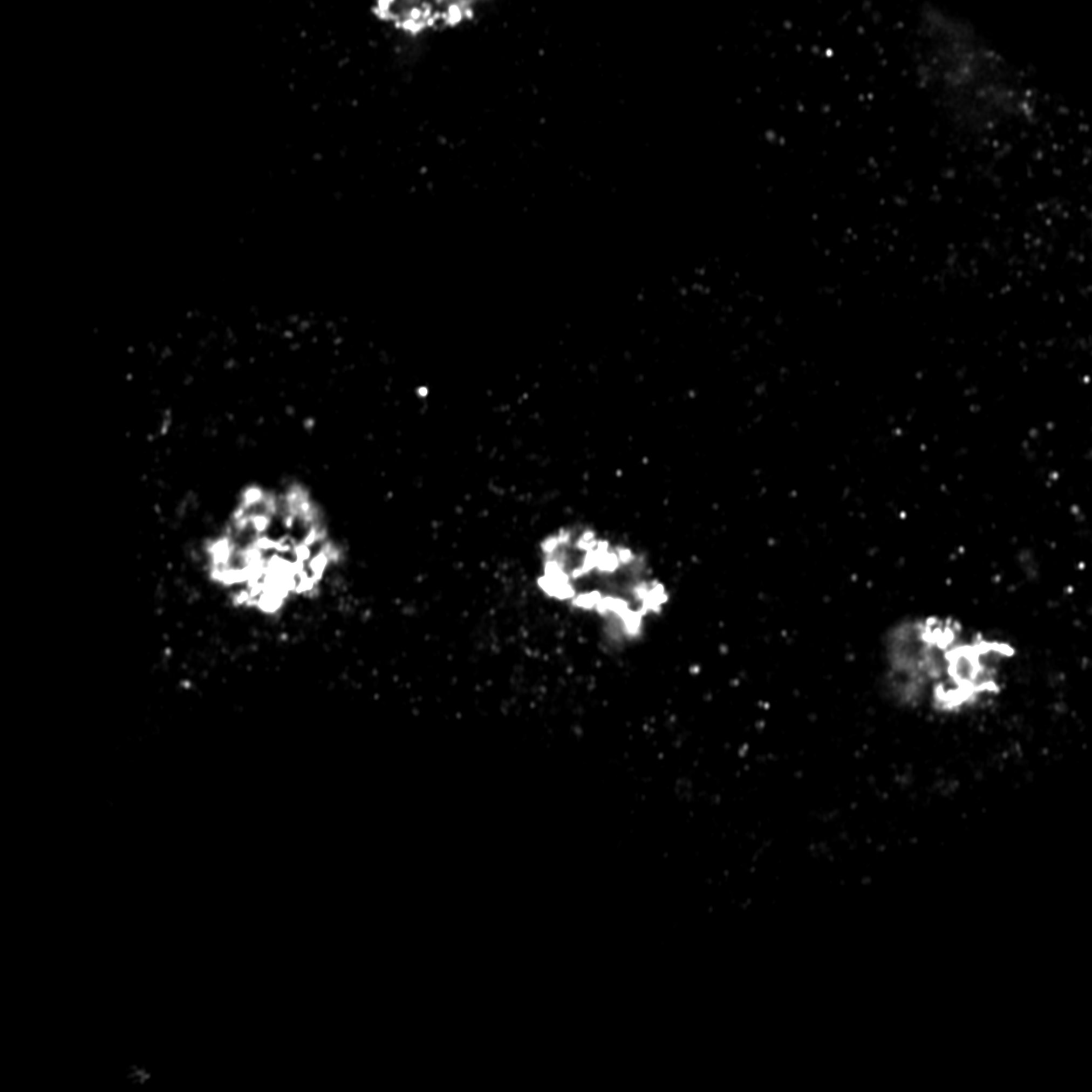

Supplement: Supplementary file 6 — Source data Fig. 3 [file 44318_2024_305_MOESM6_ESM.zip › Figure 3/3H/WT_GM_PT_ctrl_2_(PT594_C=0)_Airyscan Processing.tif]

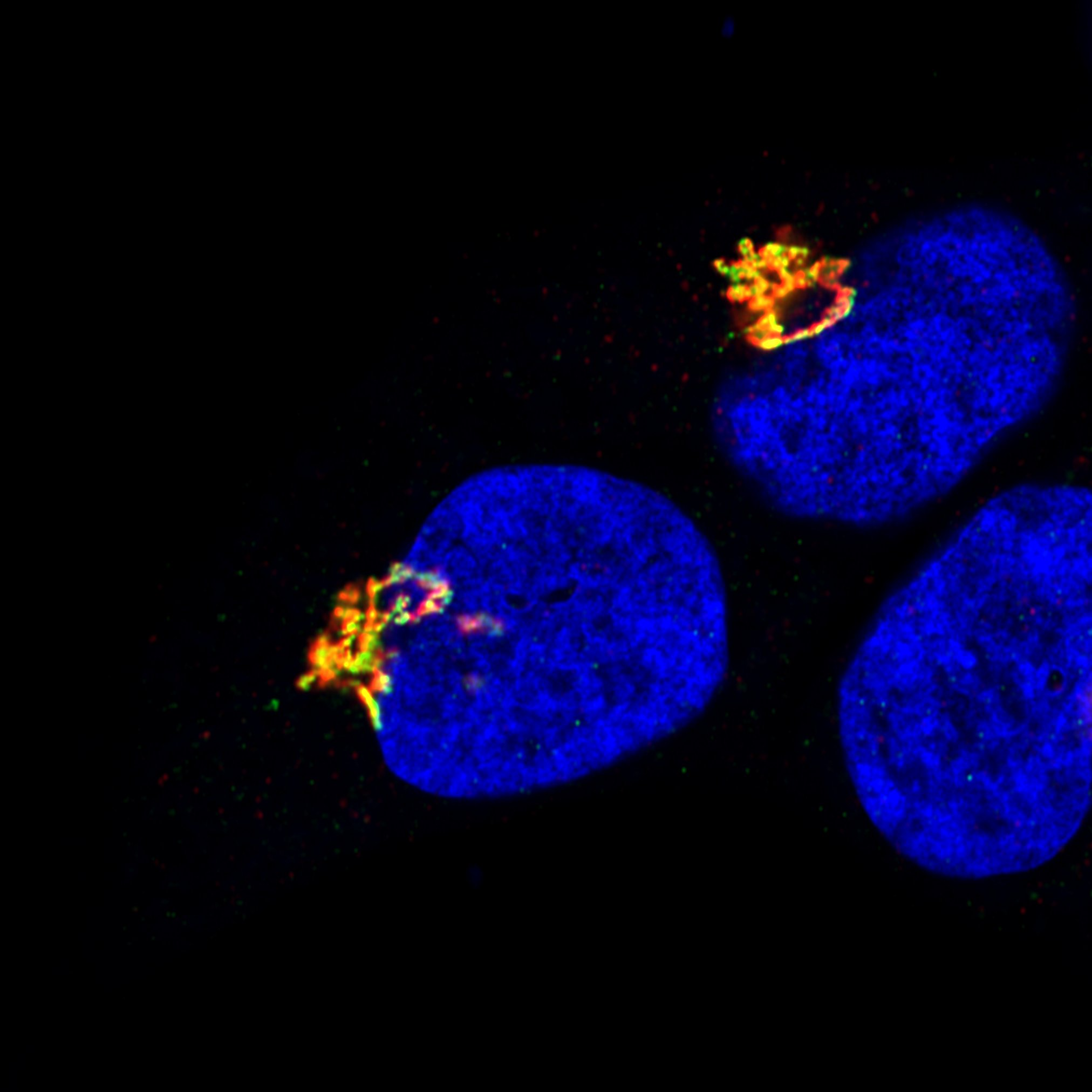

Supplement: Supplementary file 6 — Source data Fig. 3 [file 44318_2024_305_MOESM6_ESM.zip › Figure 3/3H/WT_GM_PT_ctrl_3_(merge)_Airyscan Processing.tif]

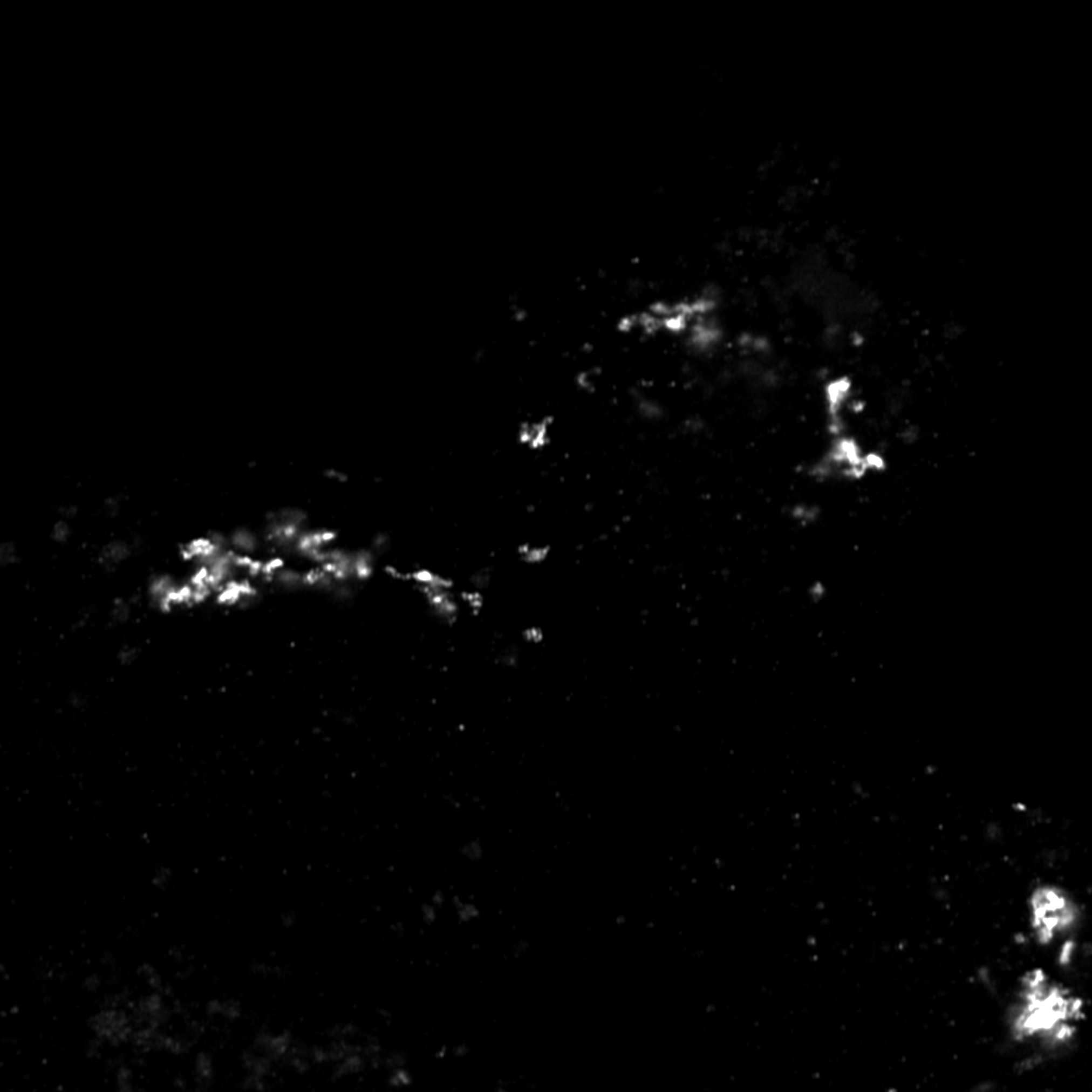

Supplement: Supplementary file 6 — Source data Fig. 3 [file 44318_2024_305_MOESM6_ESM.zip › Figure 3/3H/GOLPH_KO_GM_PT_ctrl_2_(GM130_488_C=1)_Airyscan Processing.tif]

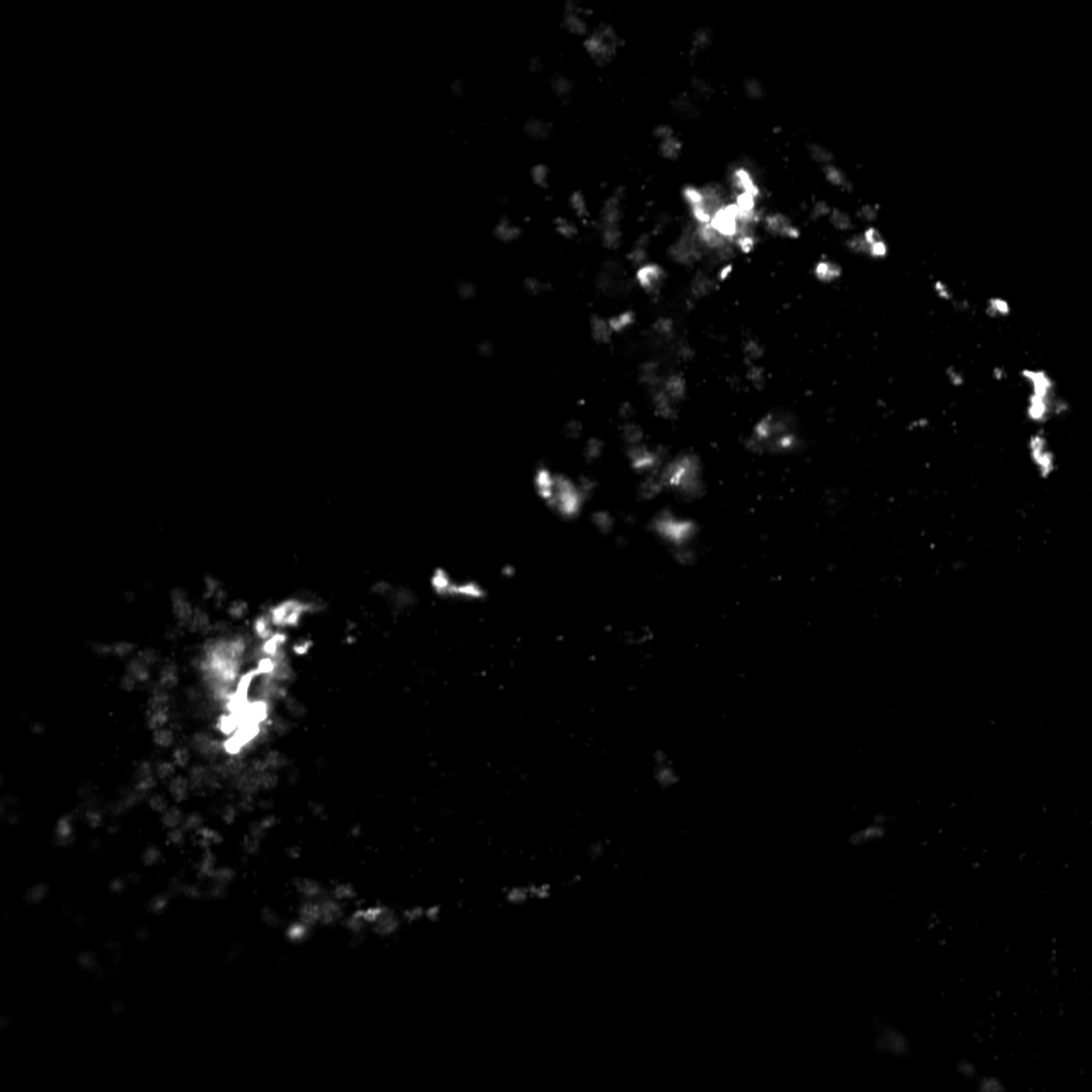

Supplement: Supplementary file 6 — Source data Fig. 3 [file 44318_2024_305_MOESM6_ESM.zip › Figure 3/3H/GOLPH_KO_GM_PT_ctrl_1_(GM130_488_C=1)_Airyscan Processing.tif]

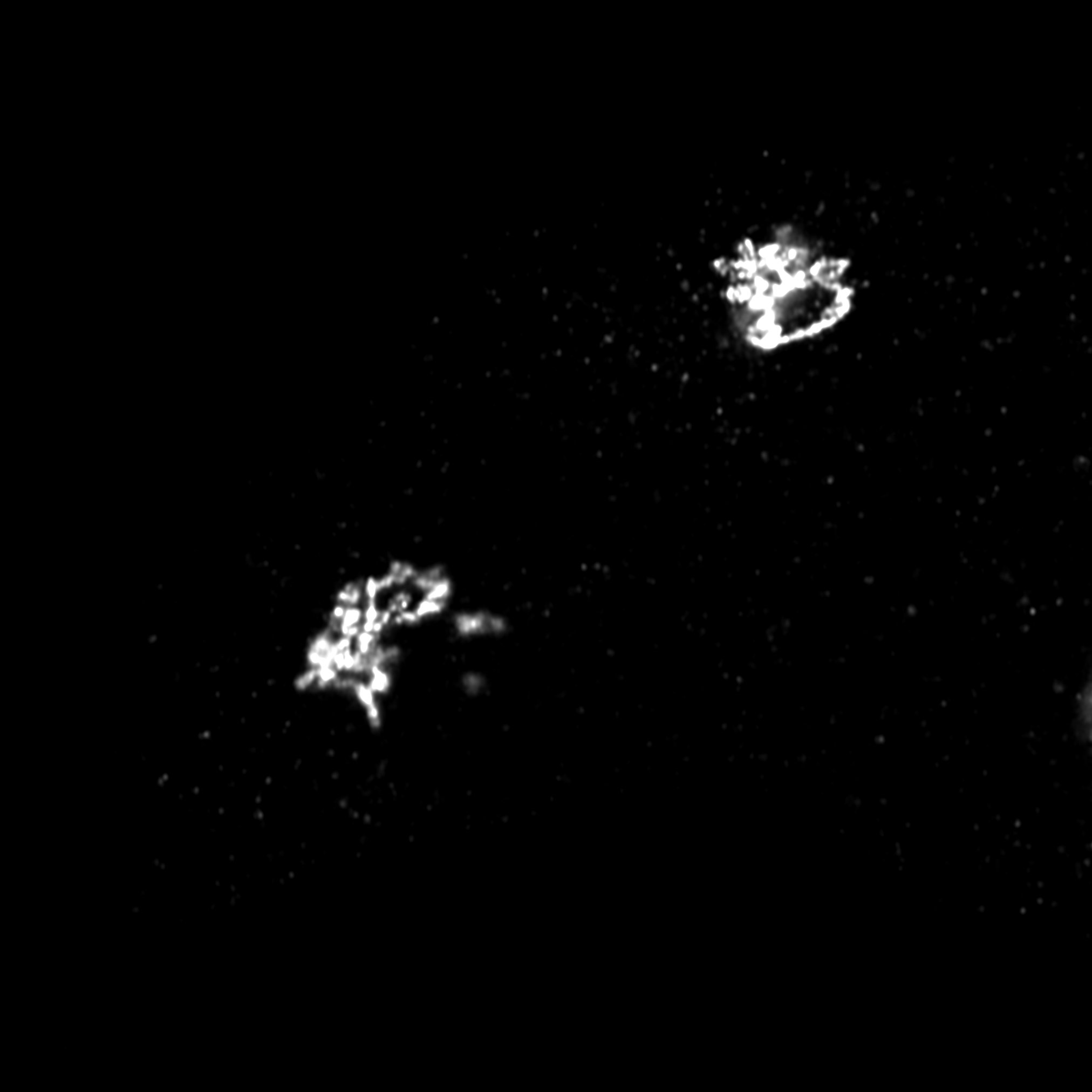

Supplement: Supplementary file 6 — Source data Fig. 3 [file 44318_2024_305_MOESM6_ESM.zip › Figure 3/3H/WT_GM_PT_ctrl_3_(PT594_C=0)_Airyscan Processing.tif]

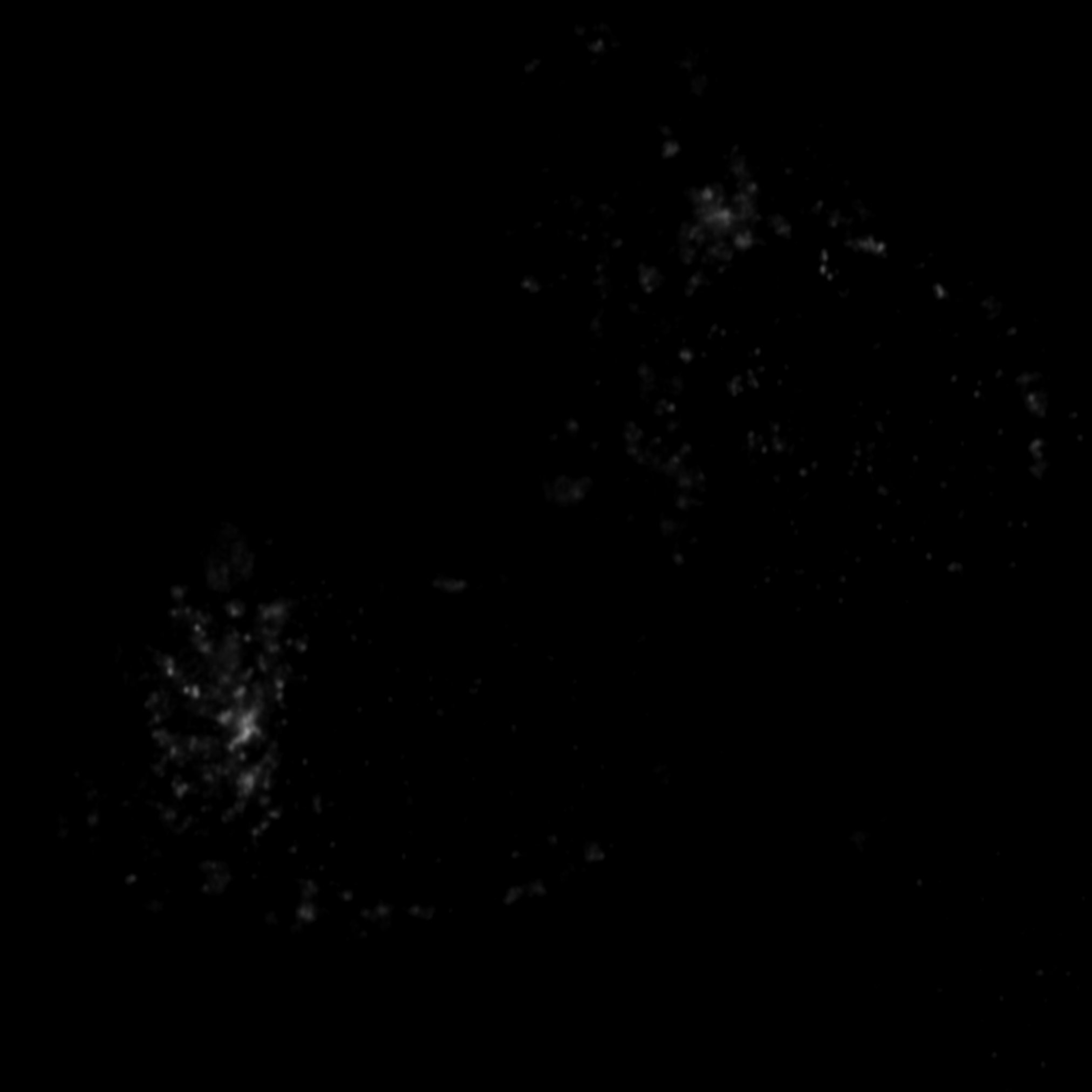

Supplement: Supplementary file 6 — Source data Fig. 3 [file 44318_2024_305_MOESM6_ESM.zip › Figure 3/3H/GOLPH_KO_GM_PT_ctrl_1_(PT594_C=0)_Airyscan Processing.tif]

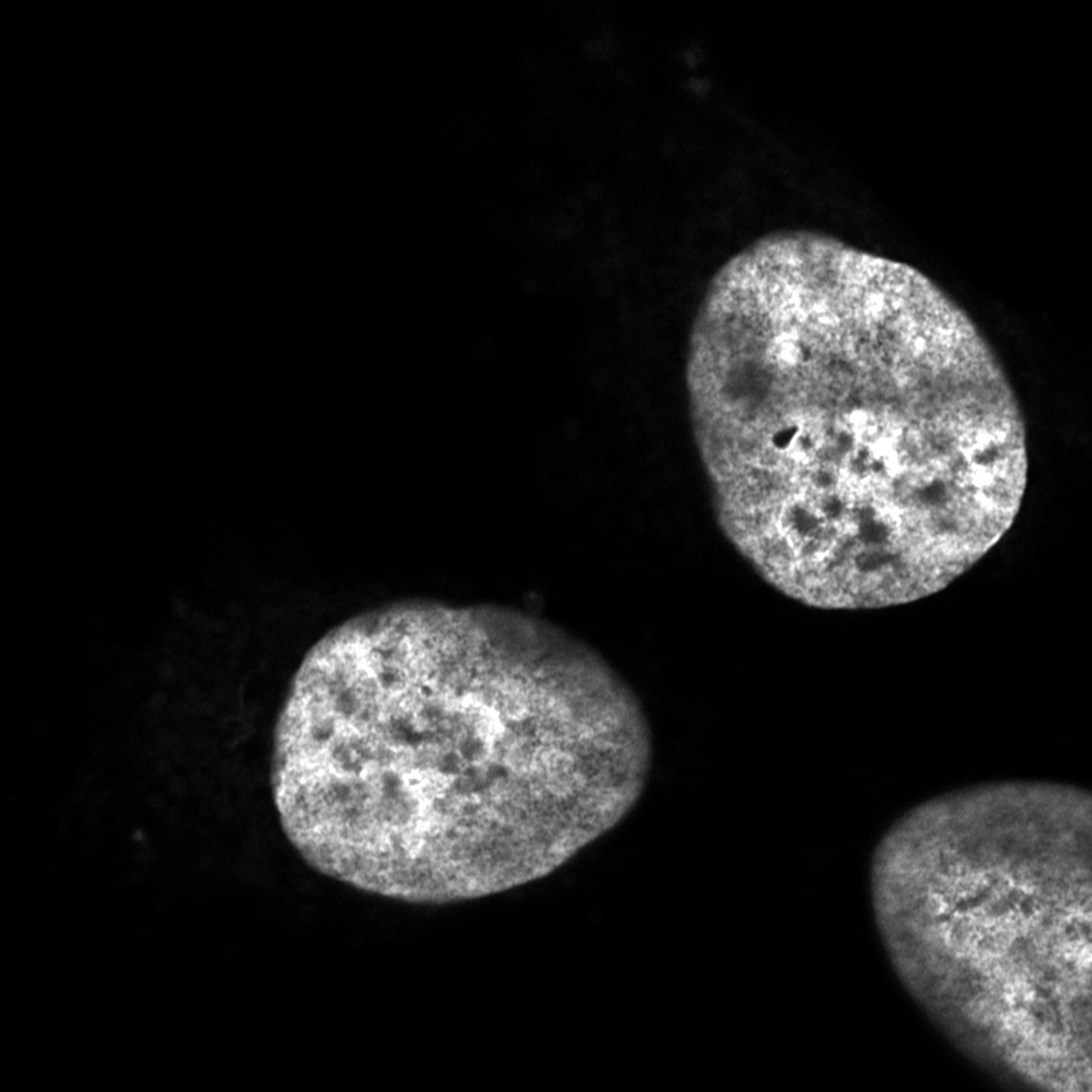

Supplement: Supplementary file 6 — Source data Fig. 3 [file 44318_2024_305_MOESM6_ESM.zip › Figure 3/3H/GOLPH_KO_GM_PT_ctrl_1_(Hoechst_C=2)_Airyscan Processing.tif]

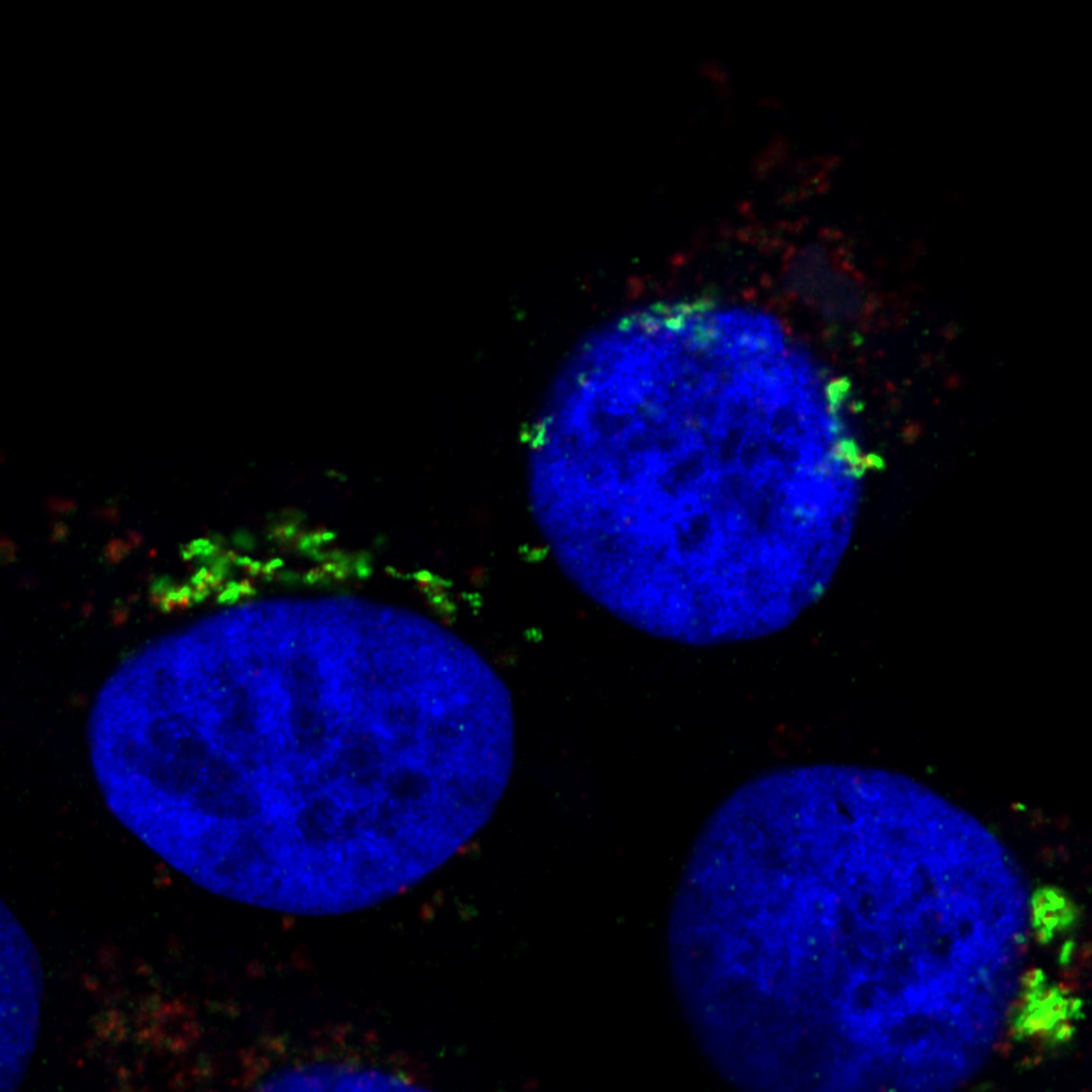

Supplement: Supplementary file 6 — Source data Fig. 3 [file 44318_2024_305_MOESM6_ESM.zip › Figure 3/3H/GOLPH_KO_GM_PT_ctrl_2_(merge)_Airyscan Processing.tif]

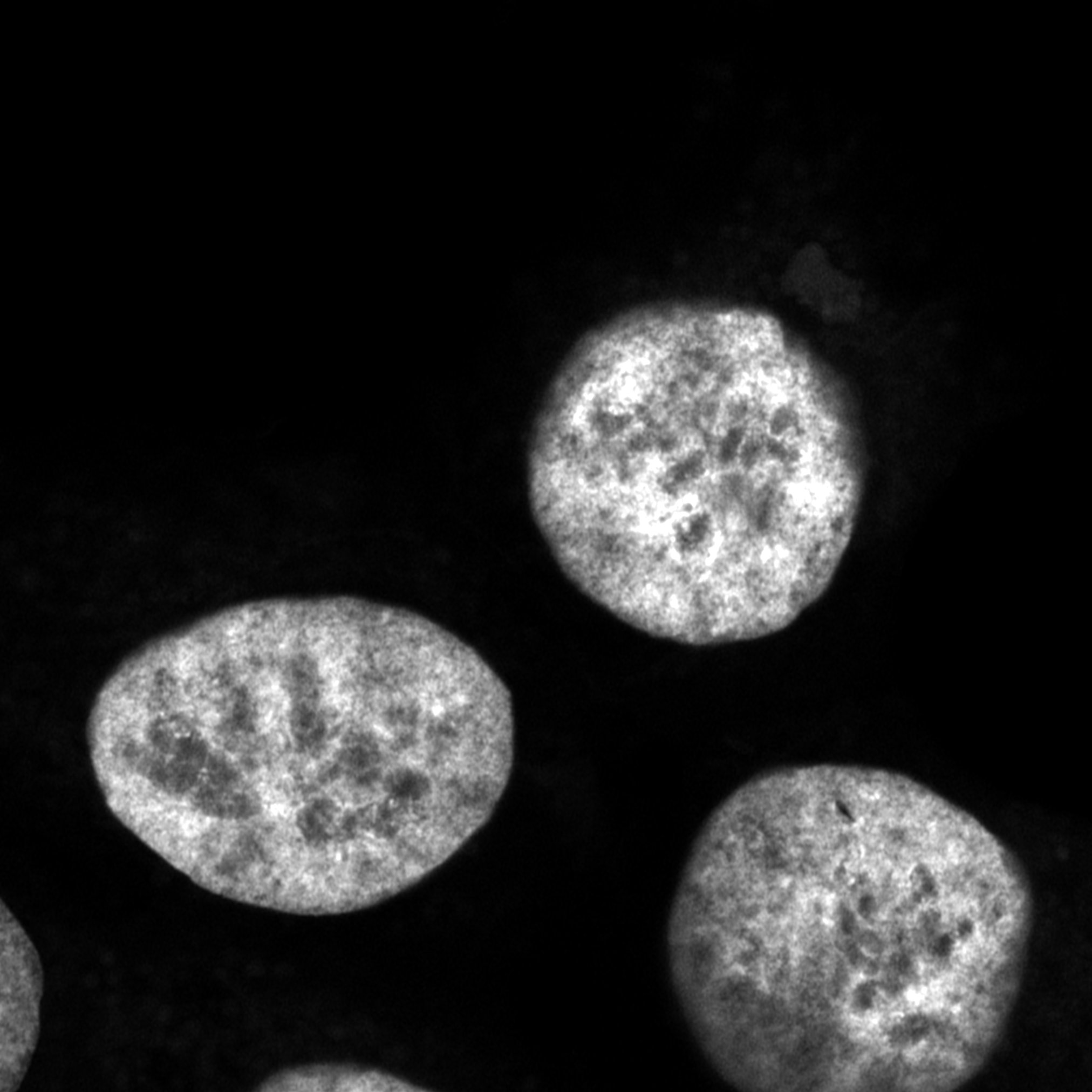

Supplement: Supplementary file 6 — Source data Fig. 3 [file 44318_2024_305_MOESM6_ESM.zip › Figure 3/3H/GOLPH_KO_GM_PT_ctrl_2_(Hoechst_C=2)_Airyscan Processing.tif]

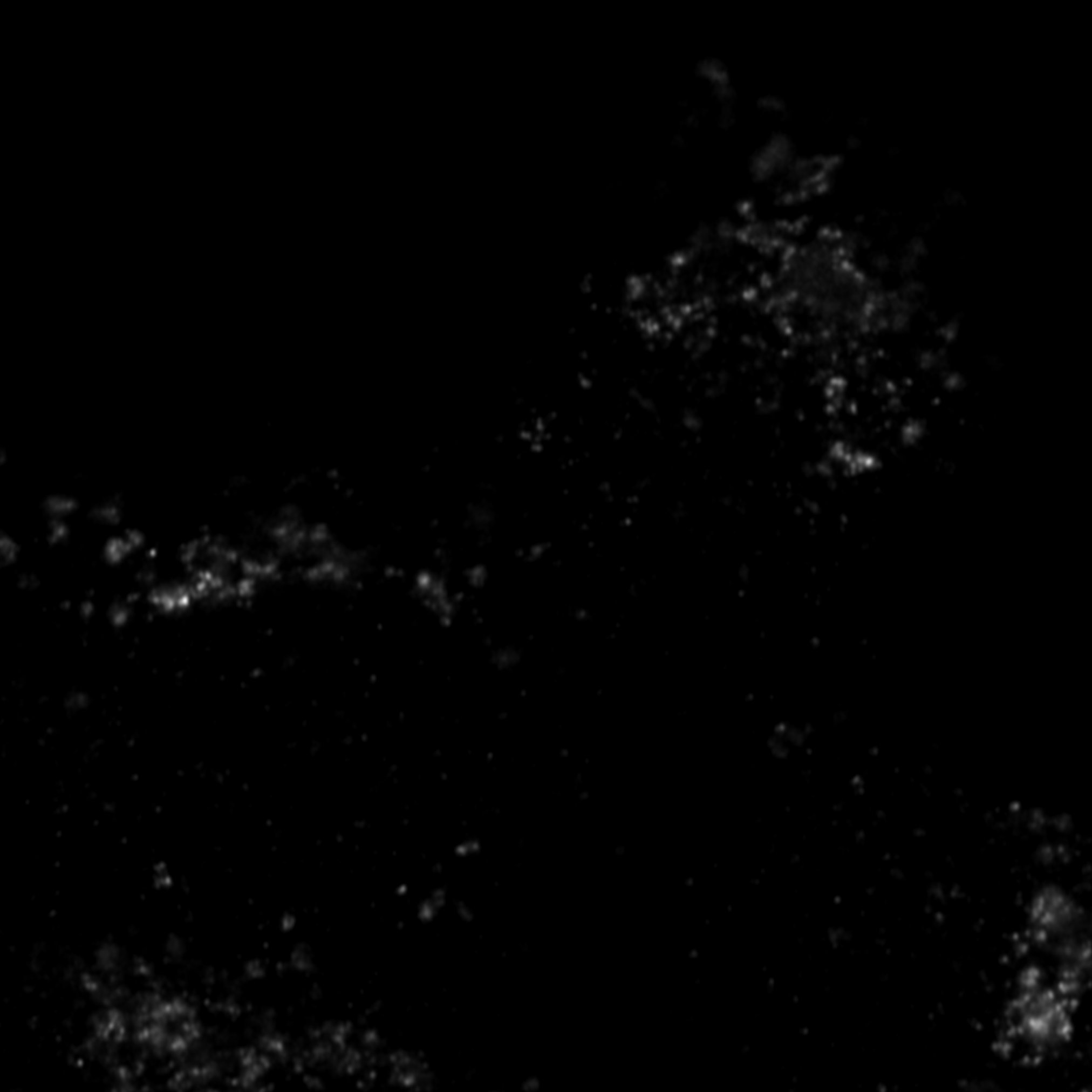

Supplement: Supplementary file 6 — Source data Fig. 3 [file 44318_2024_305_MOESM6_ESM.zip › Figure 3/3H/GOLPH_KO_GM_PT_ctrl_2_(PT594_C=0)_Airyscan Processing.tif]

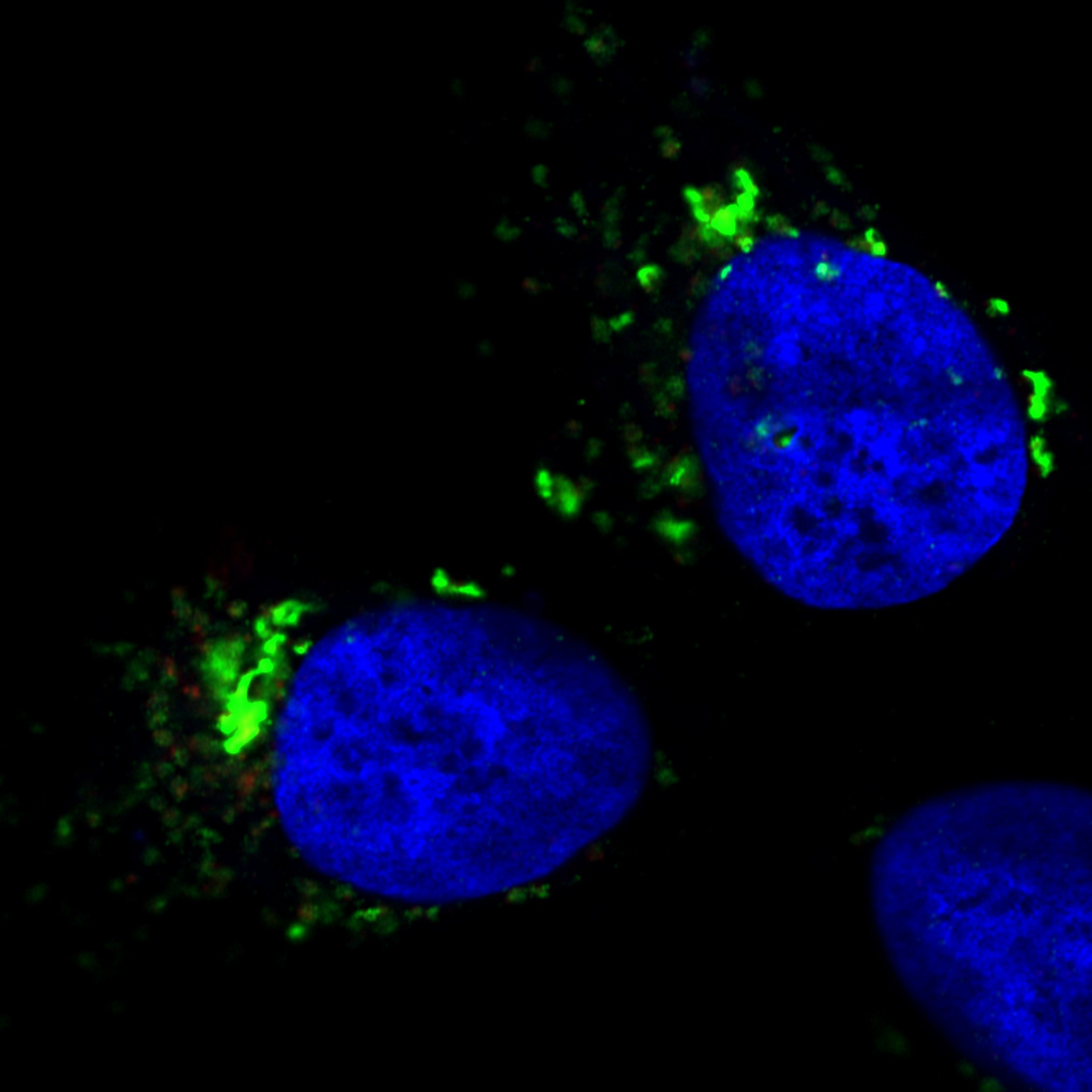

Supplement: Supplementary file 6 — Source data Fig. 3 [file 44318_2024_305_MOESM6_ESM.zip › Figure 3/3H/GOLPH_KO_GM_PT_ctrl_1_(merge)_Airyscan Processing.tif]

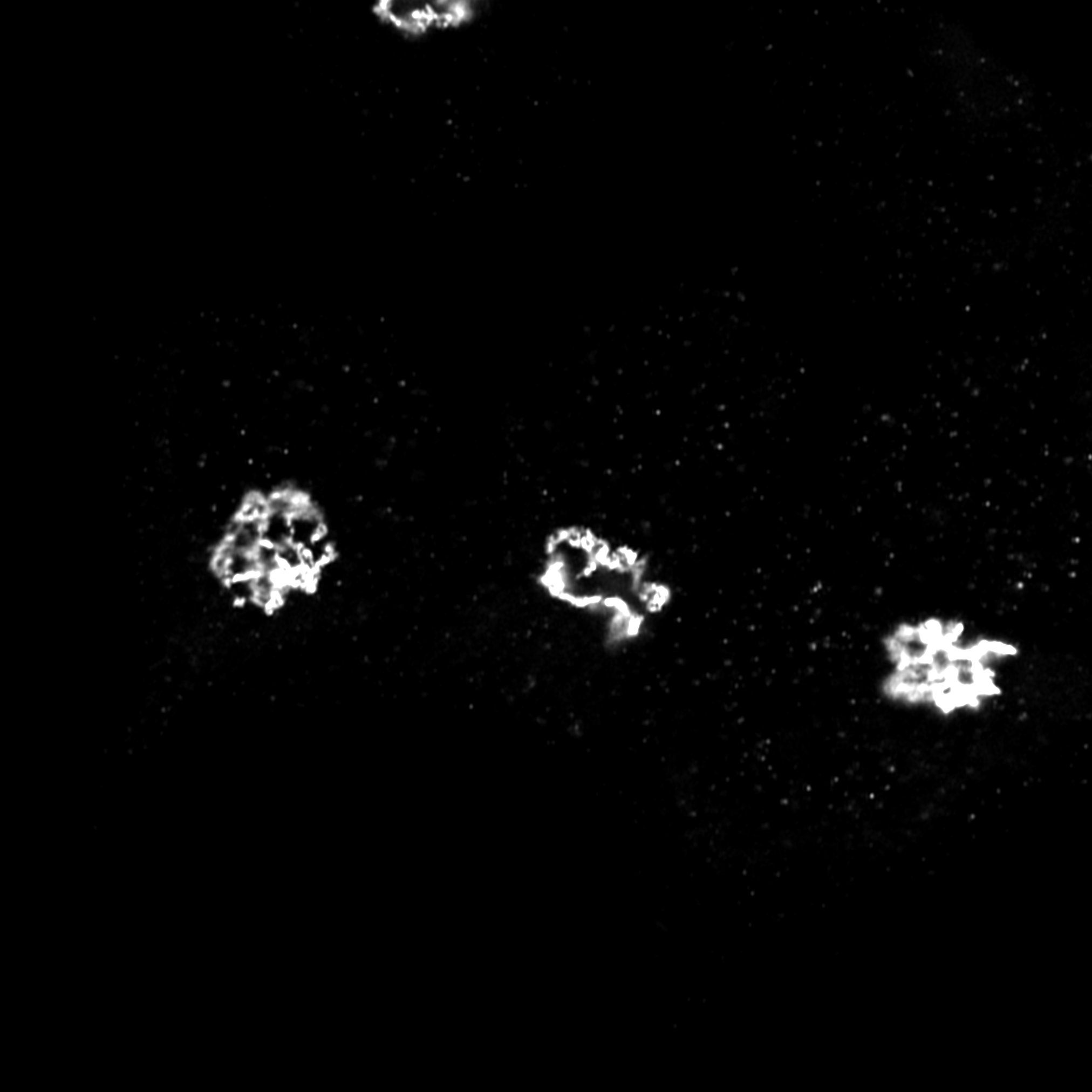

Supplement: Supplementary file 6 — Source data Fig. 3 [file 44318_2024_305_MOESM6_ESM.zip › Figure 3/3H/WT_GM_PT_ctrl_2_(GM130_488_C=1)_Airyscan Processing.tif]

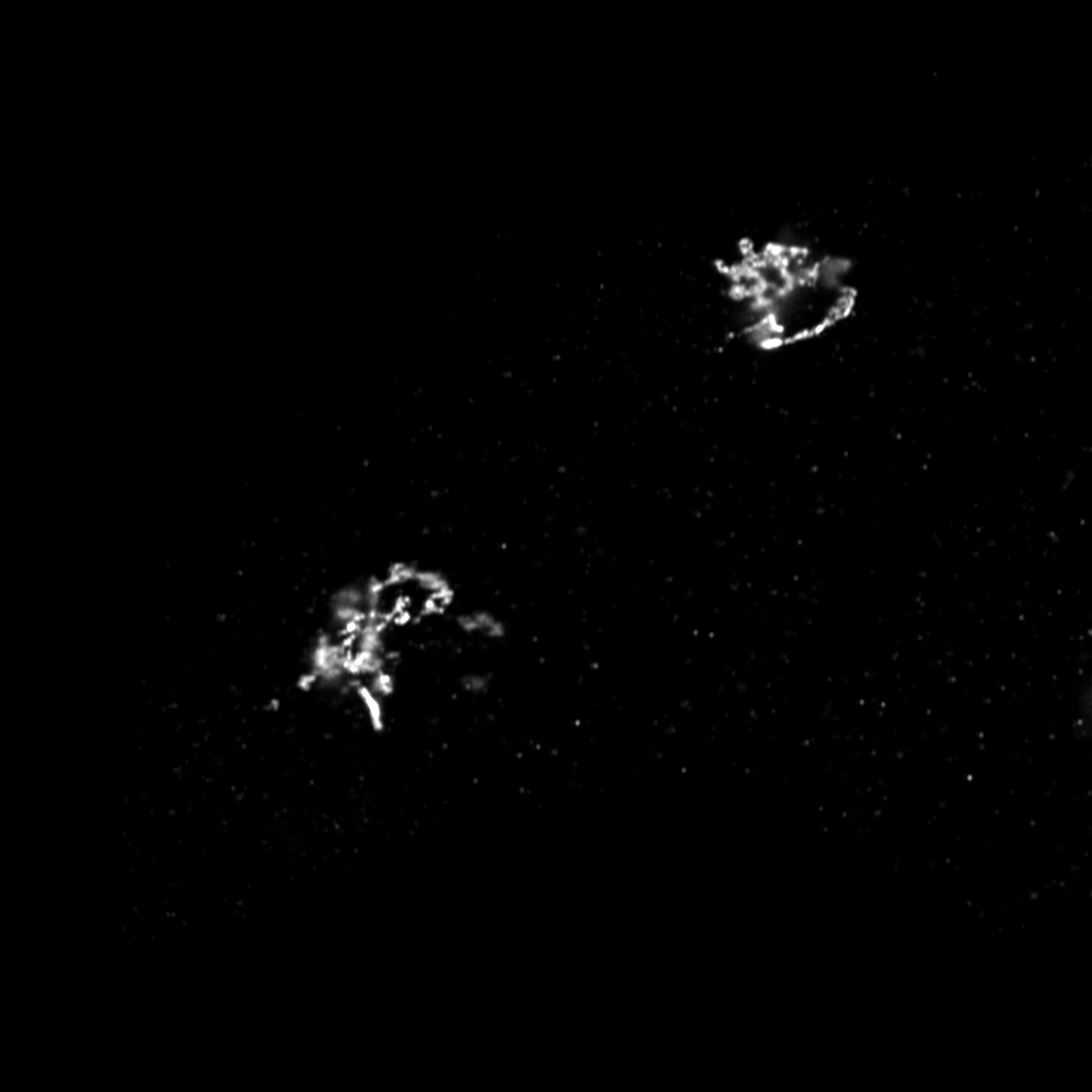

Supplement: Supplementary file 6 — Source data Fig. 3 [file 44318_2024_305_MOESM6_ESM.zip › Figure 3/3H/WT_GM_PT_ctrl_3_(GM130_488_C=1)_Airyscan Processing.tif]

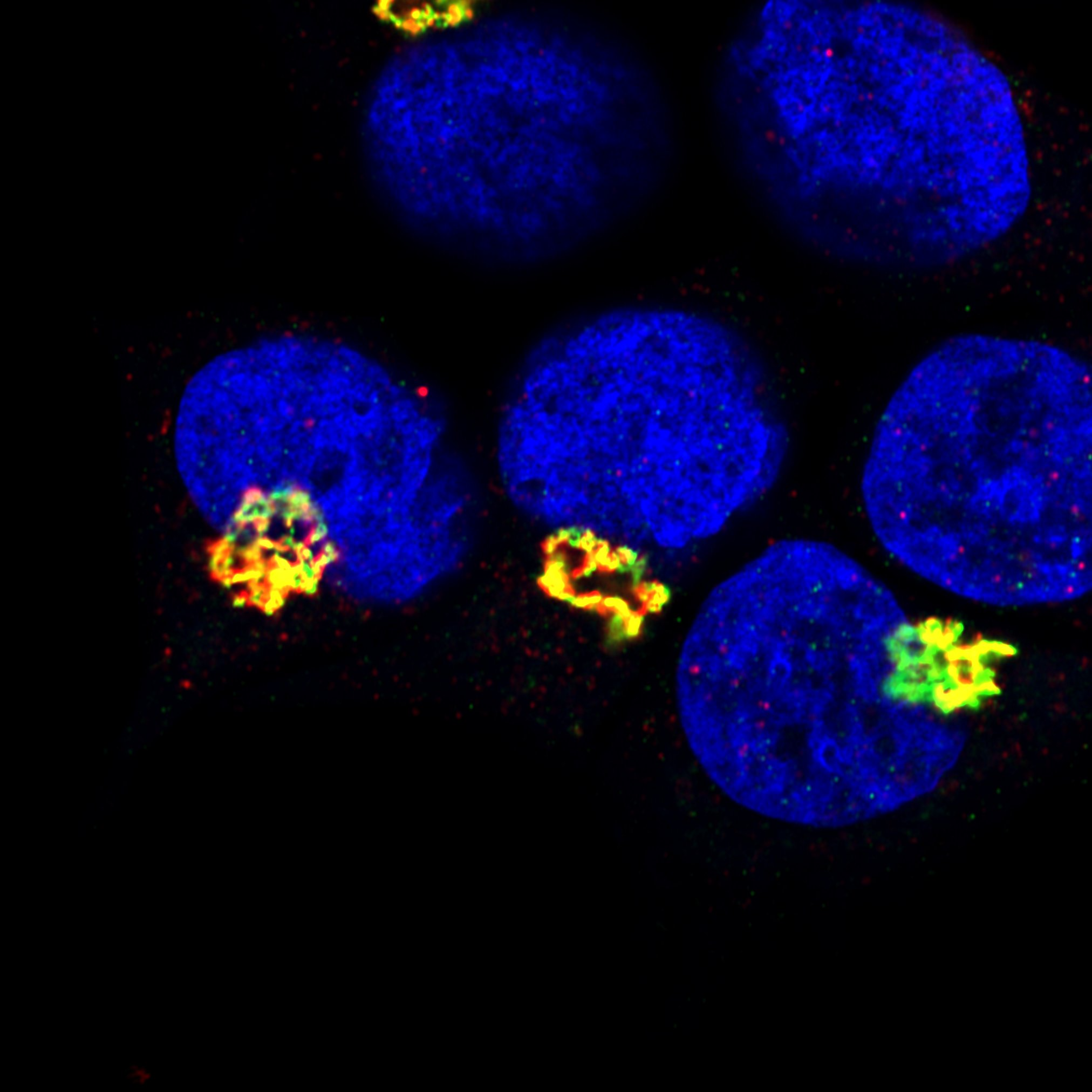

Supplement: Supplementary file 6 — Source data Fig. 3 [file 44318_2024_305_MOESM6_ESM.zip › Figure 3/3H/WT_GM_PT_ctrl_2_(merge)_Airyscan Processing.tif]

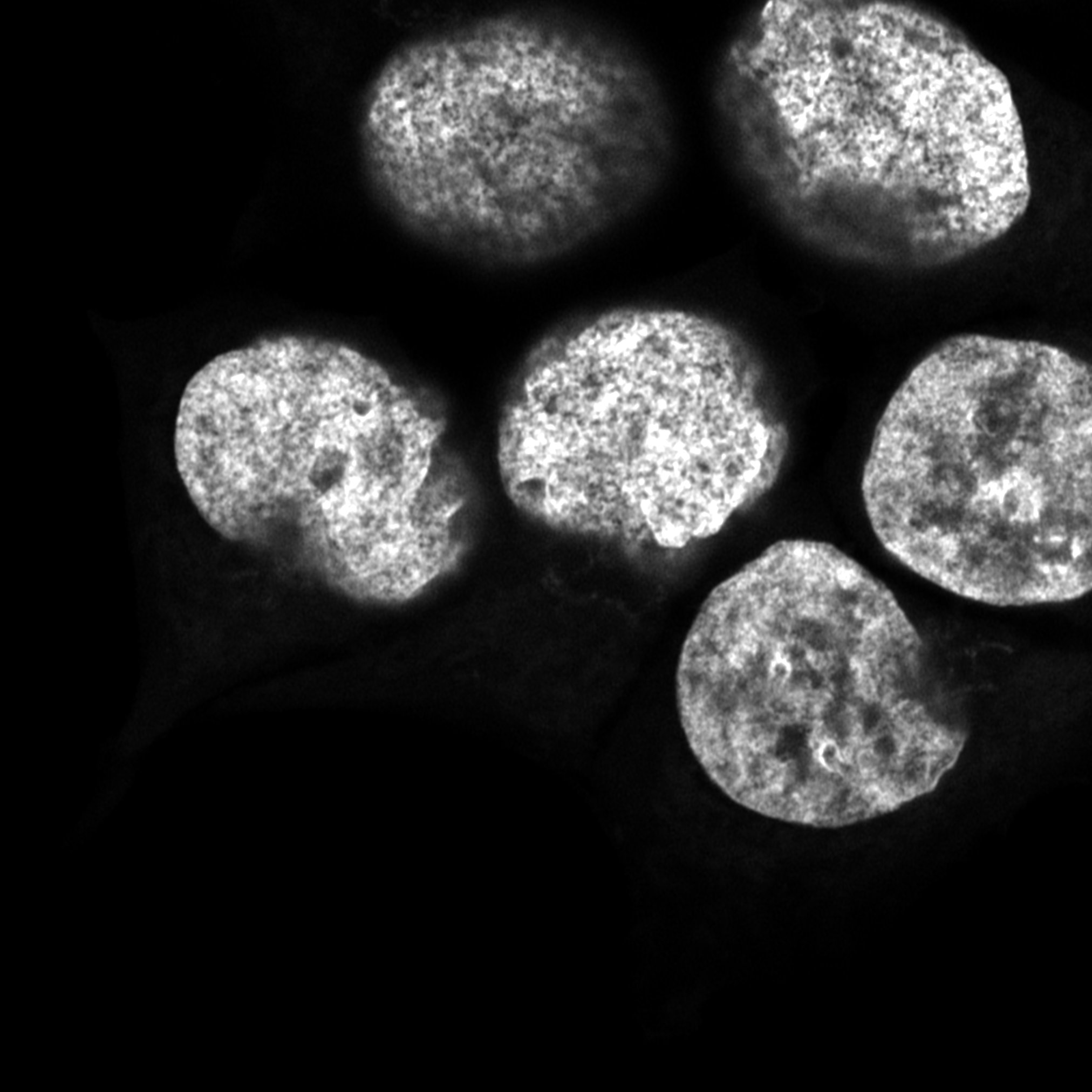

Supplement: Supplementary file 6 — Source data Fig. 3 [file 44318_2024_305_MOESM6_ESM.zip › Figure 3/3H/WT_GM_PT_ctrl_2_(Hoechst_C=2)_Airyscan Processing.tif]

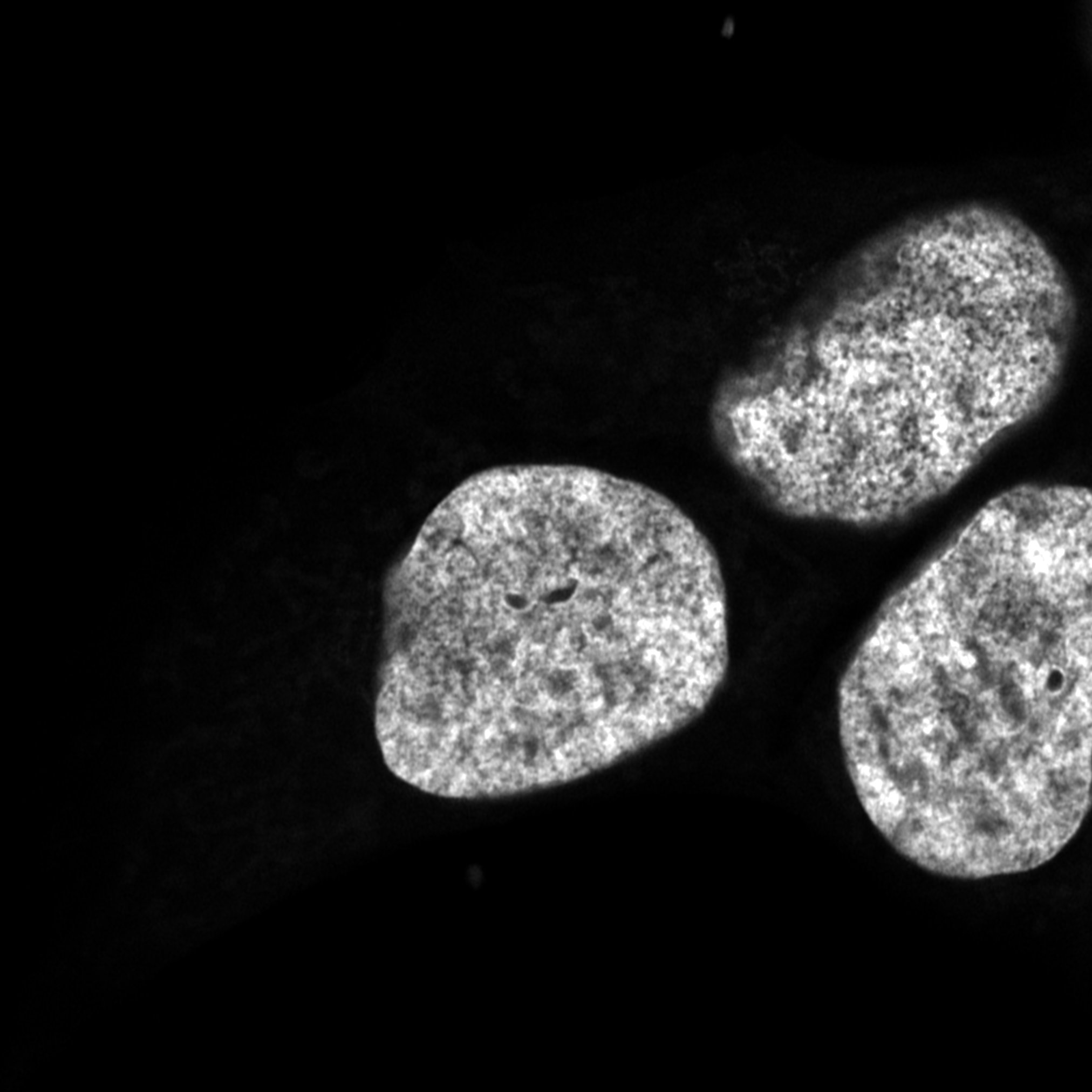

Supplement: Supplementary file 6 — Source data Fig. 3 [file 44318_2024_305_MOESM6_ESM.zip › Figure 3/3H/WT_GM_PT_ctrl_3_(Hoechst_C=2)_Airyscan Processing.tif]

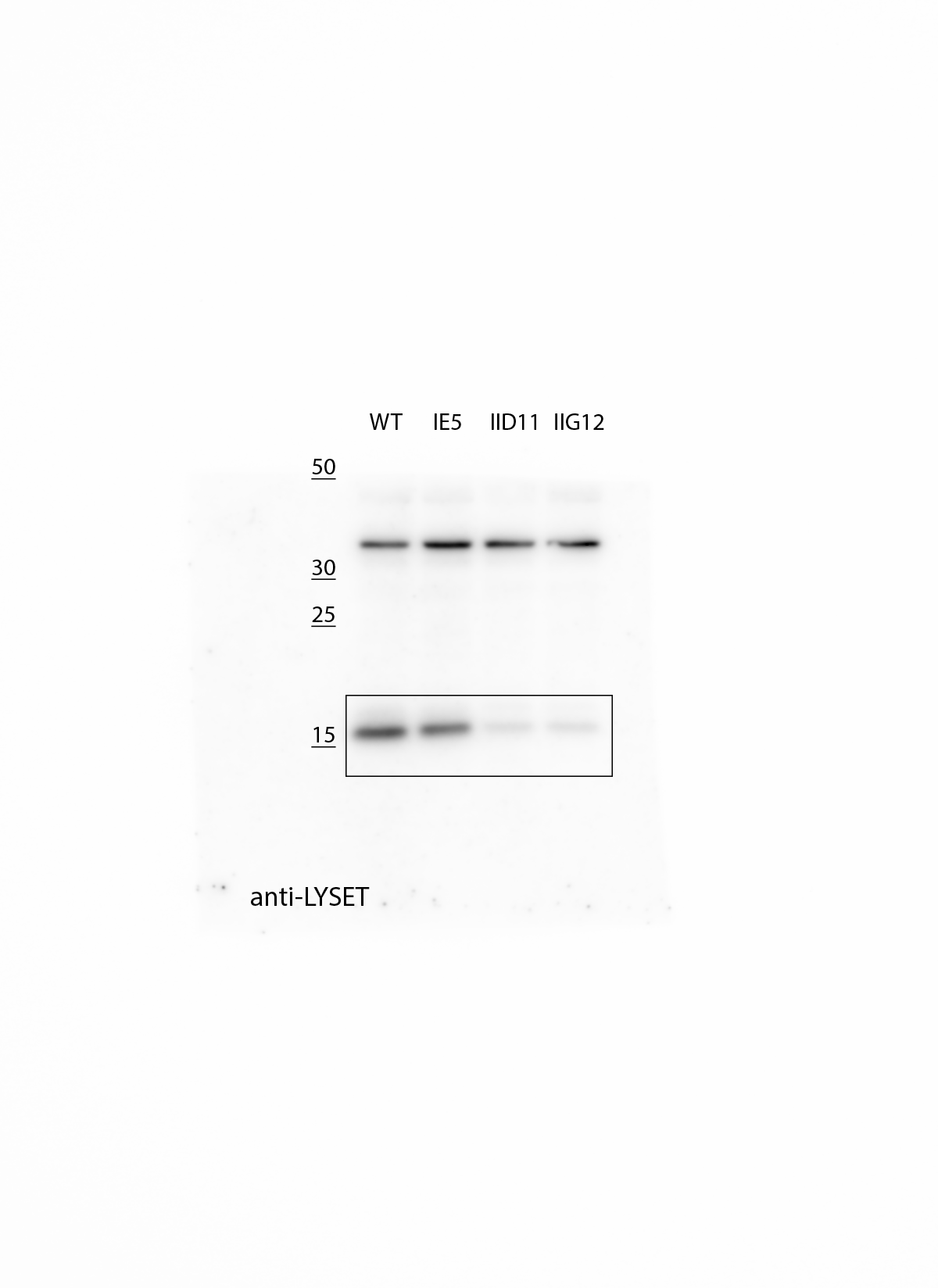

Supplement: Supplementary file 6 — Source data Fig. 3 [file 44318_2024_305_MOESM6_ESM.zip › Figure 3/3F/source data LYSET.tif]

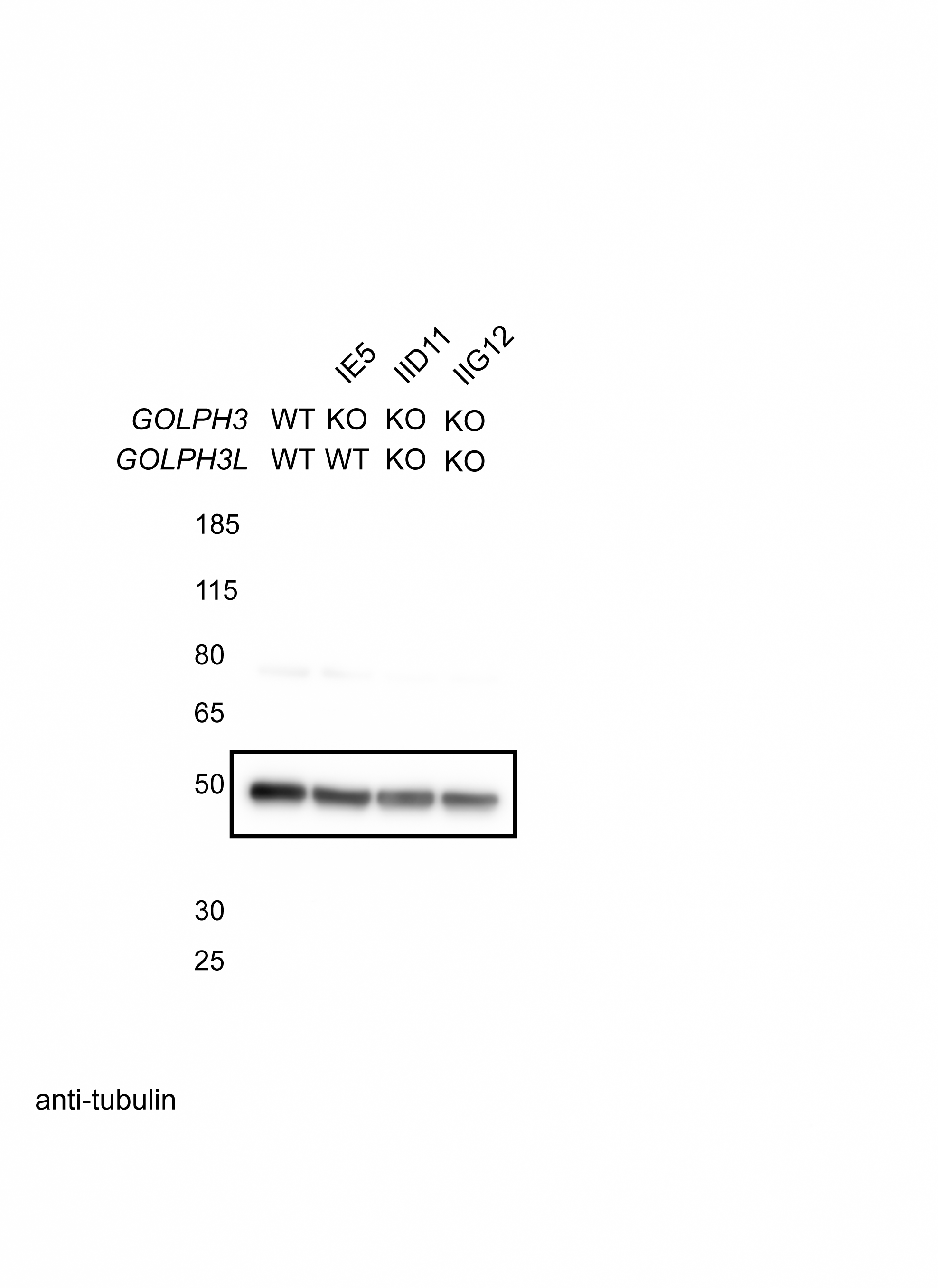

Supplement: Supplementary file 6 — Source data Fig. 3 [file 44318_2024_305_MOESM6_ESM.zip › Figure 3/3F/Tubulin for MAN1B1-01.tif]

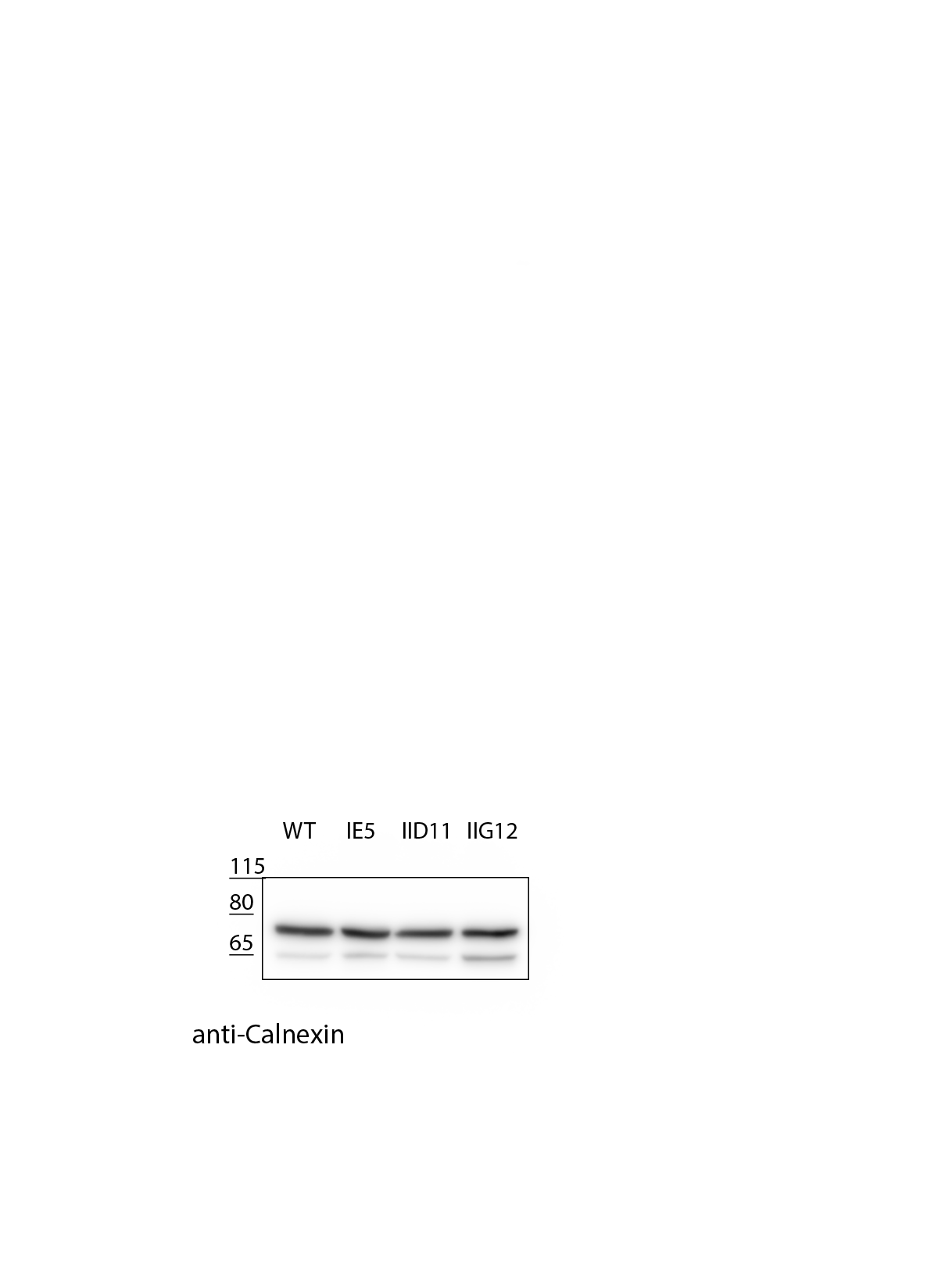

Supplement: Supplementary file 6 — Source data Fig. 3 [file 44318_2024_305_MOESM6_ESM.zip › Figure 3/3F/source data Calnexin for LYSET.tif]

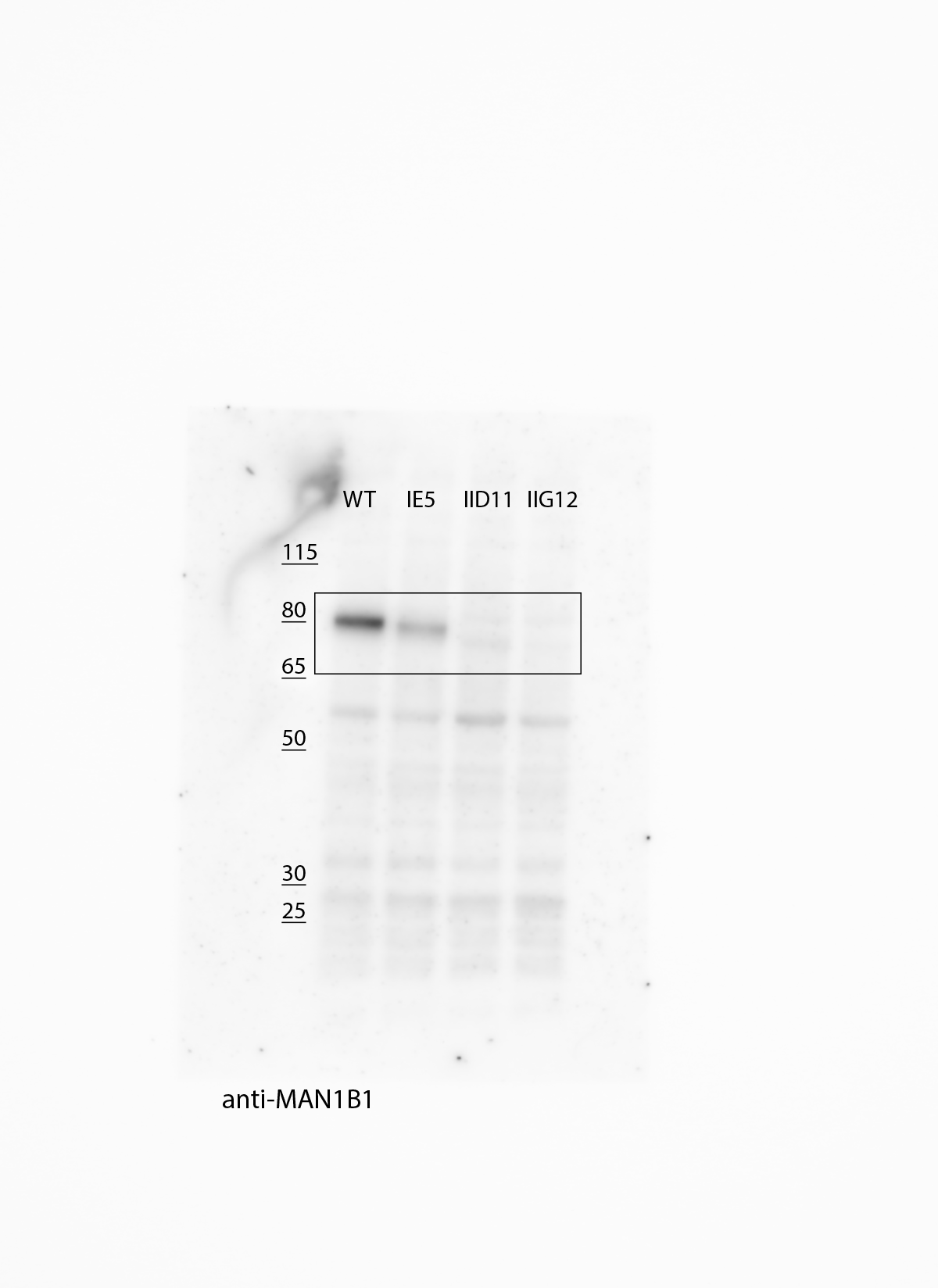

Supplement: Supplementary file 6 — Source data Fig. 3 [file 44318_2024_305_MOESM6_ESM.zip › Figure 3/3F/source data MAN1B1.tif]

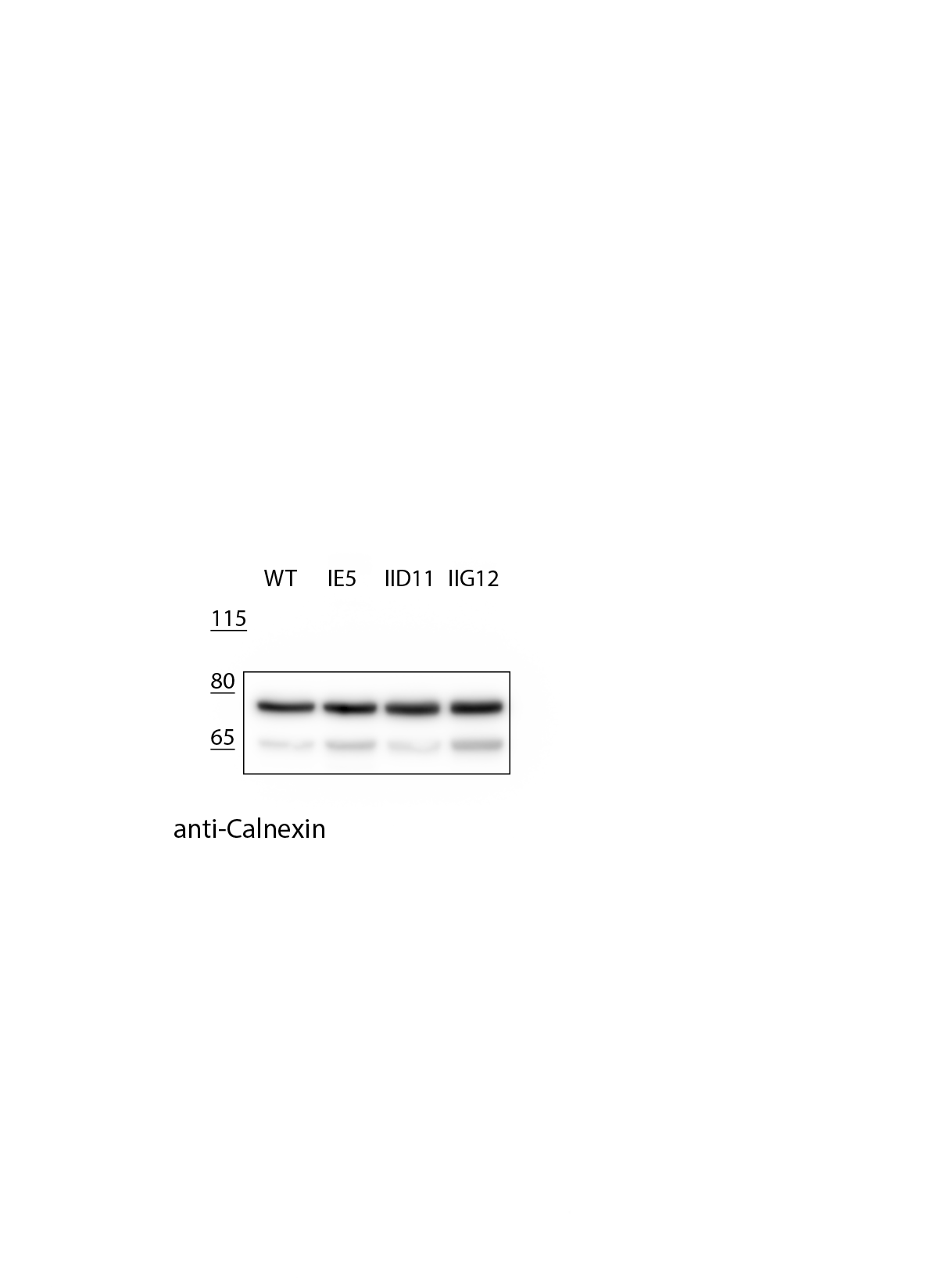

Supplement: Supplementary file 6 — Source data Fig. 3 [file 44318_2024_305_MOESM6_ESM.zip › Figure 3/3F/source data Calnexin for GOLPH3.tif]

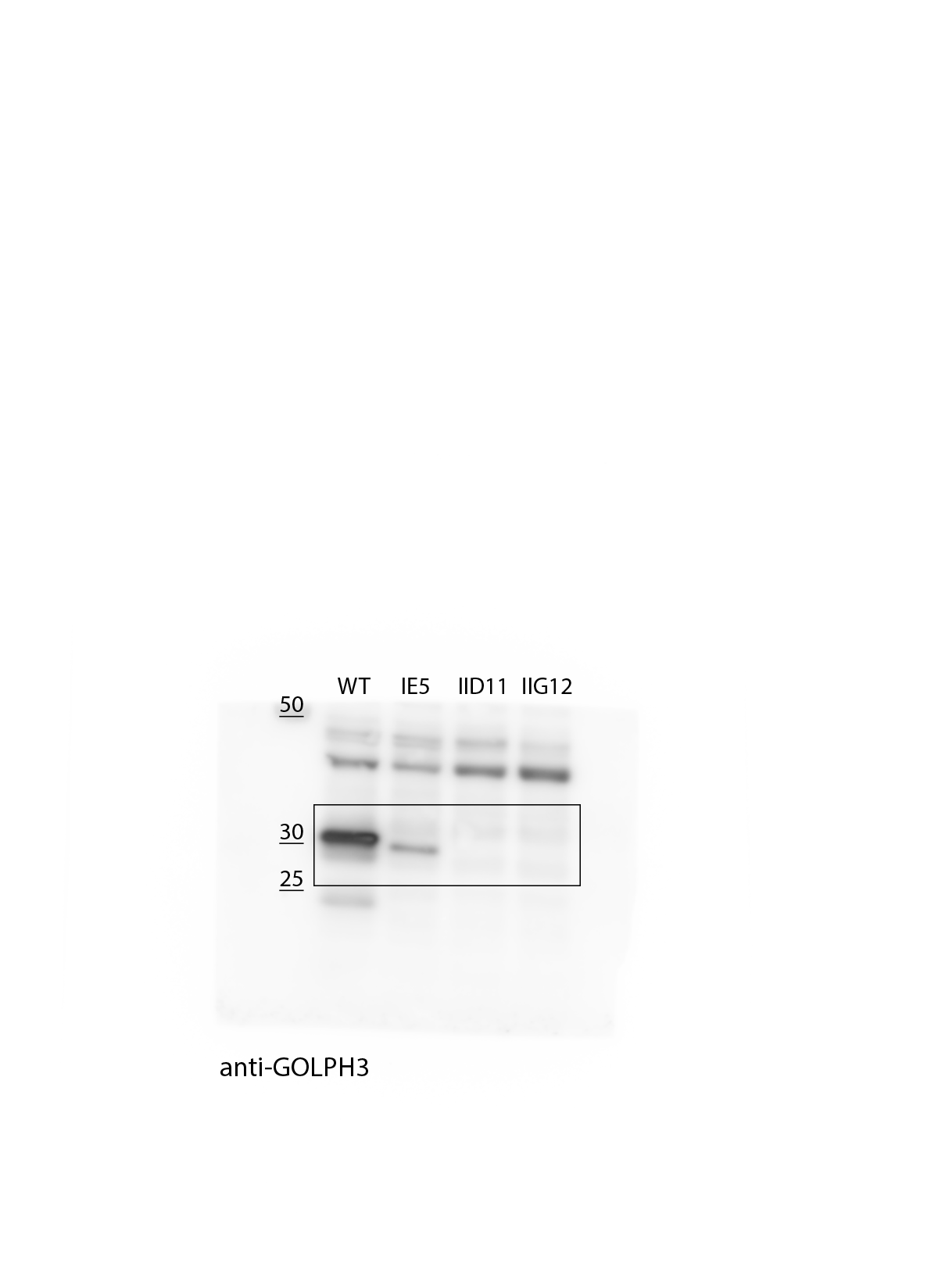

Supplement: Supplementary file 6 — Source data Fig. 3 [file 44318_2024_305_MOESM6_ESM.zip › Figure 3/3F/source data GOLPH3.tif]

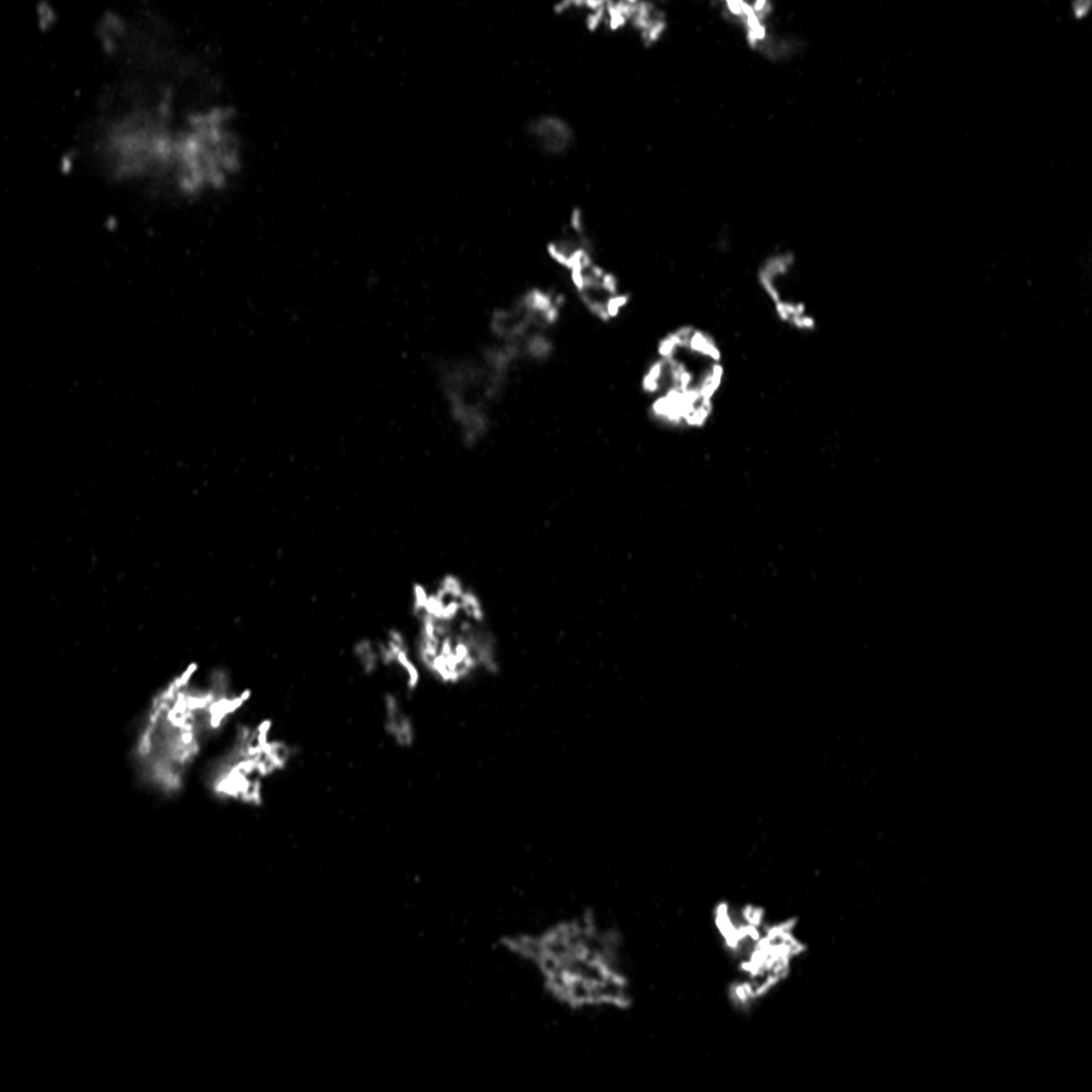

Supplement: Supplementary file 6 — Source data Fig. 3 [file 44318_2024_305_MOESM6_ESM.zip › Figure 3/3G/WT_ctrl_LYSET_GM_3_(GM130_594_C=0))_Airyscan Processing.tiff]

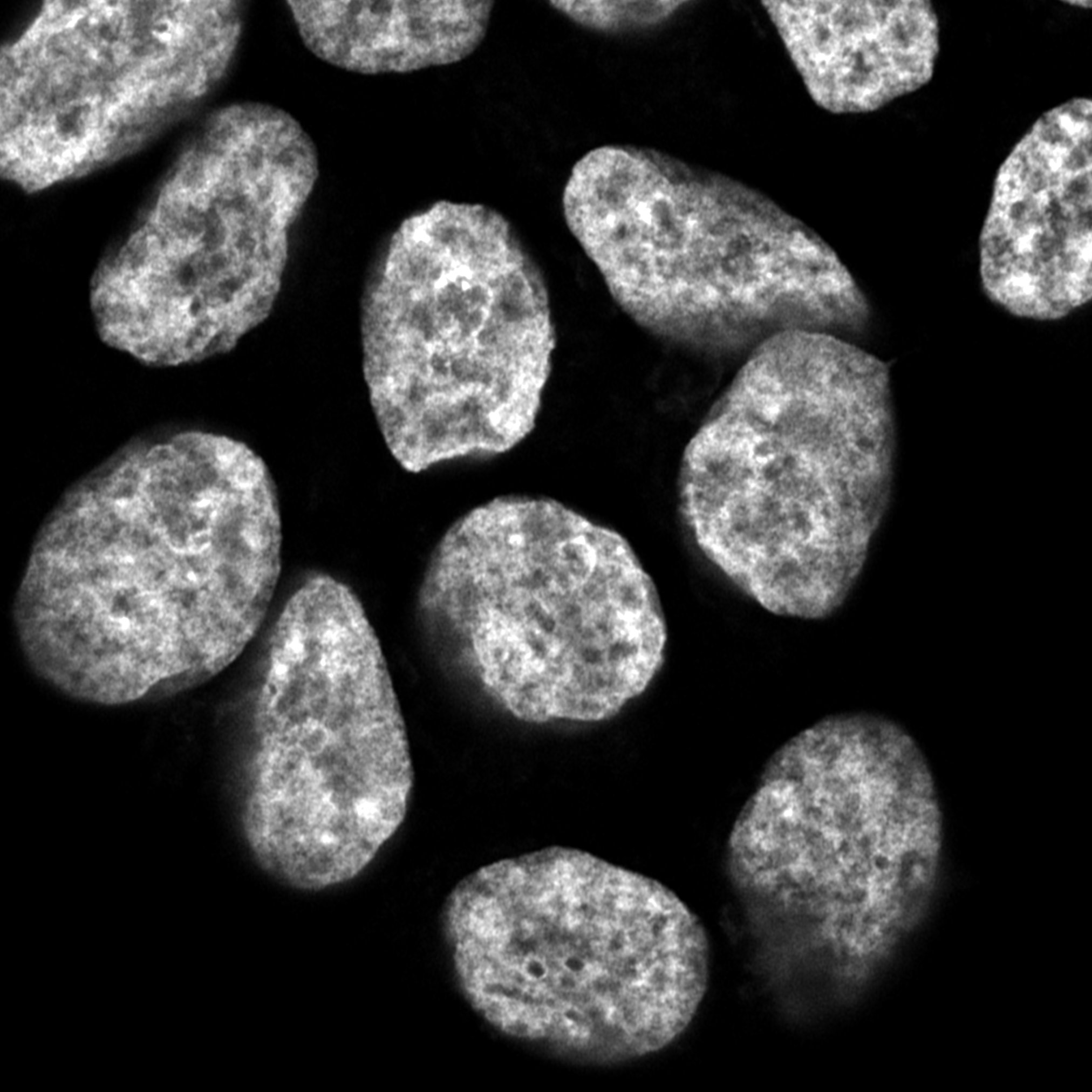

Supplement: Supplementary file 6 — Source data Fig. 3 [file 44318_2024_305_MOESM6_ESM.zip › Figure 3/3G/WT_ctrl_LYSET_GM_3_(Hoechst_C=2)_Airyscan Processing.tiff]

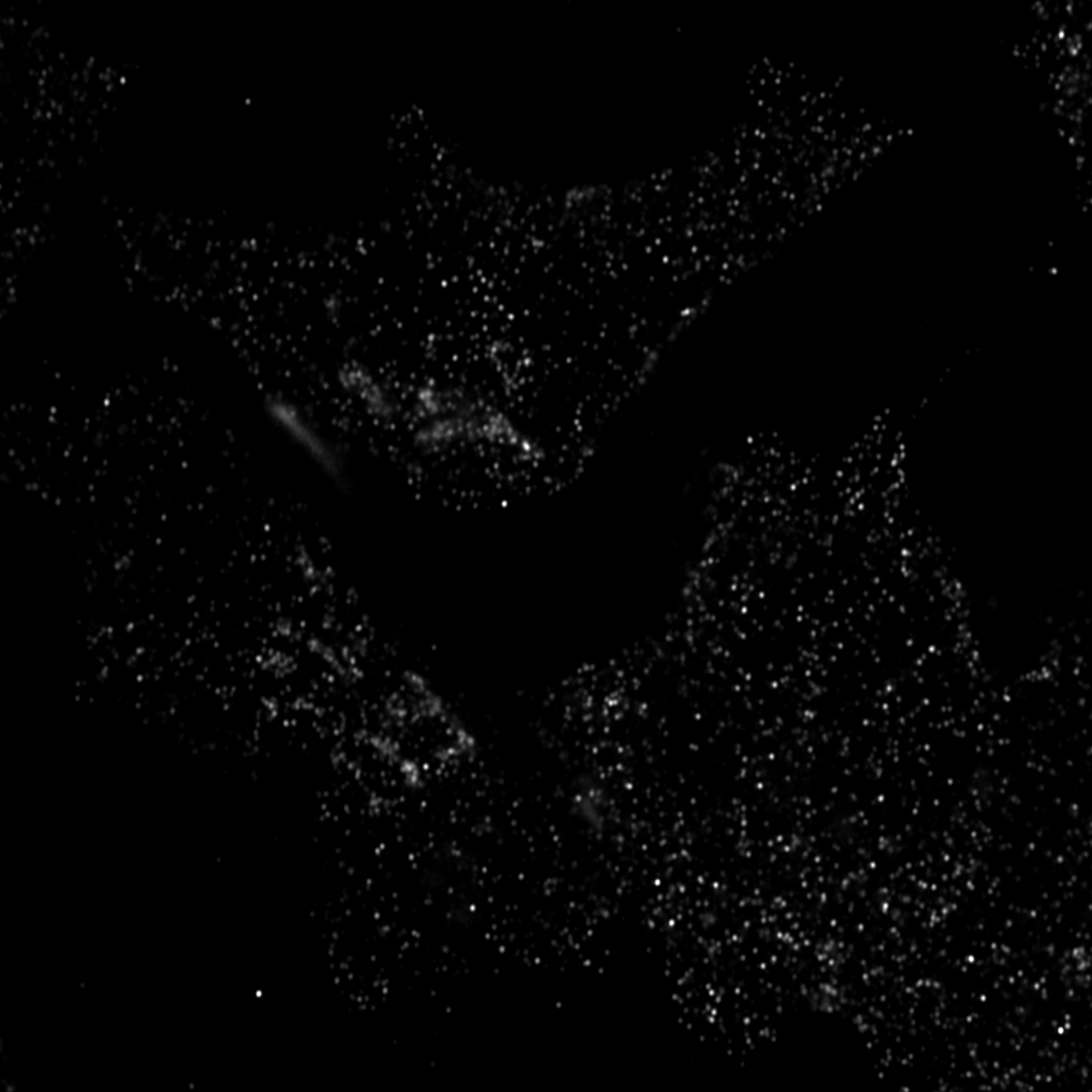

Supplement: Supplementary file 6 — Source data Fig. 3 [file 44318_2024_305_MOESM6_ESM.zip › Figure 3/3G/GOLPH_KO_ctrl_LYSET_GM_3_(LYSET488_C=1))_Airyscan Processing.tiff]

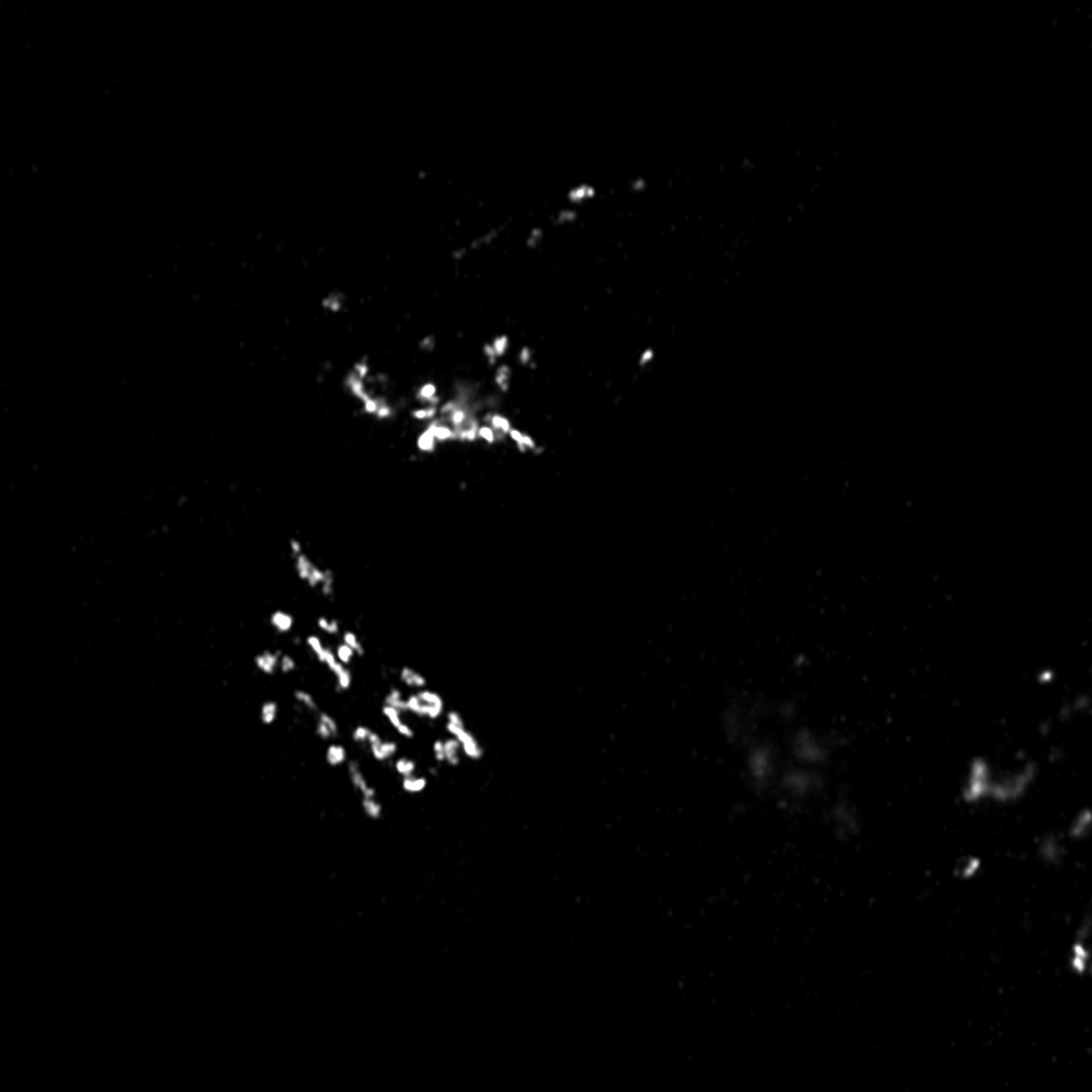

Supplement: Supplementary file 6 — Source data Fig. 3 [file 44318_2024_305_MOESM6_ESM.zip › Figure 3/3G/GOLPH_KO_ctrl_LYSET_GM_3_(GM130_594_C=0))_Airyscan Processing.tiff]

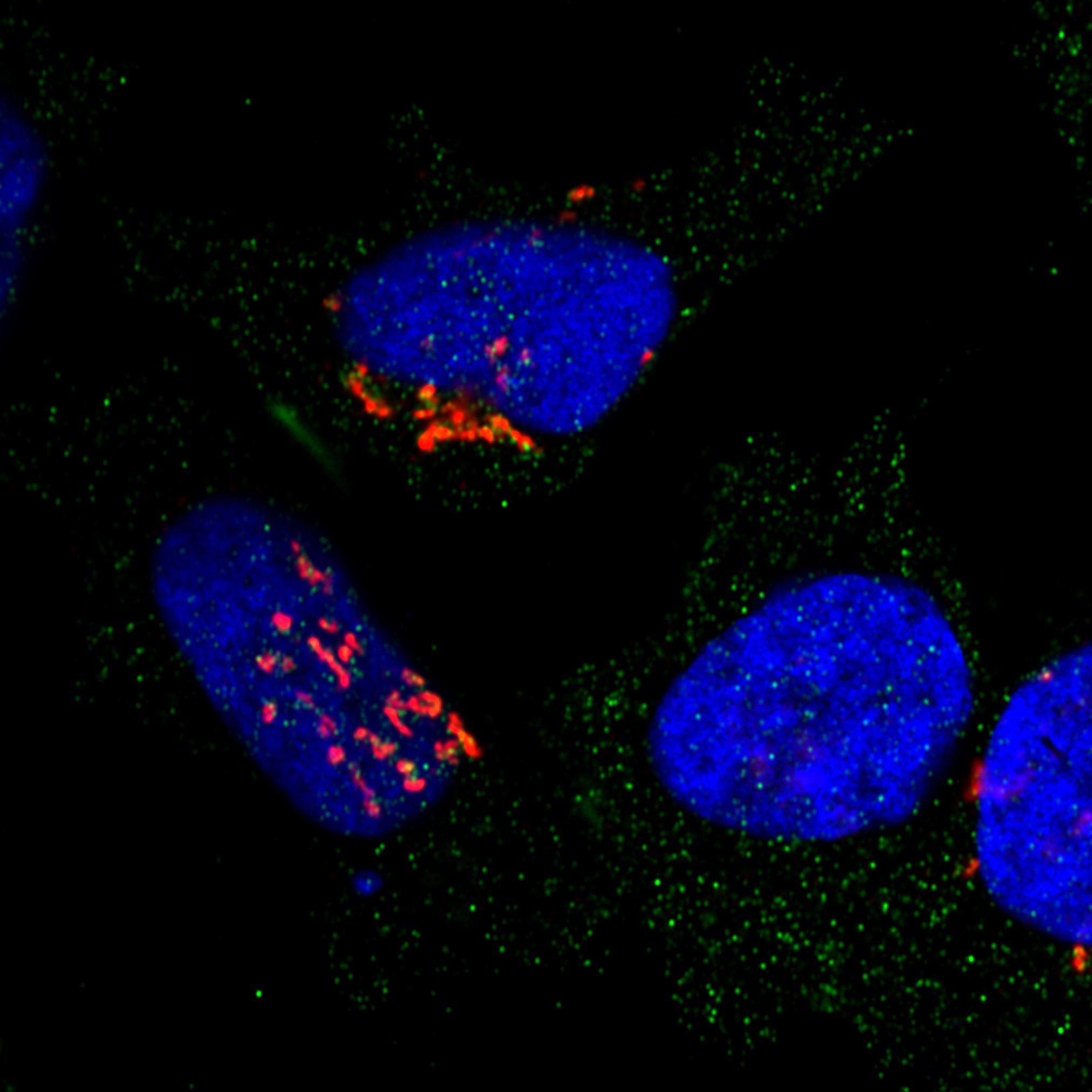

Supplement: Supplementary file 6 — Source data Fig. 3 [file 44318_2024_305_MOESM6_ESM.zip › Figure 3/3G/GOLPH_KO_ctrl_LYSET_GM_3_(merge)_Airyscan Processing.tiff]

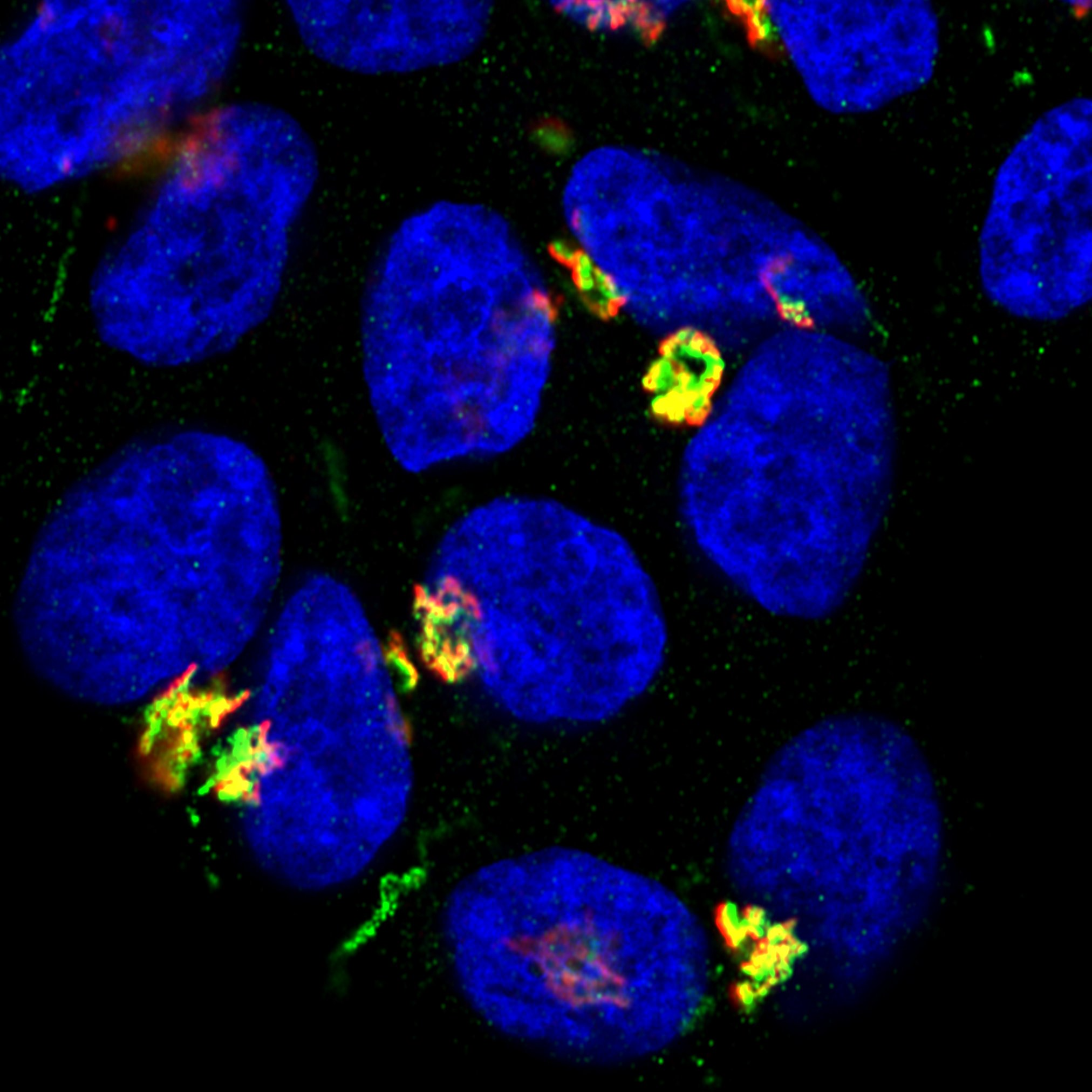

Supplement: Supplementary file 6 — Source data Fig. 3 [file 44318_2024_305_MOESM6_ESM.zip › Figure 3/3G/WT_ctrl_LYSET_GM_3_(merge)_Airyscan Processing.tiff]

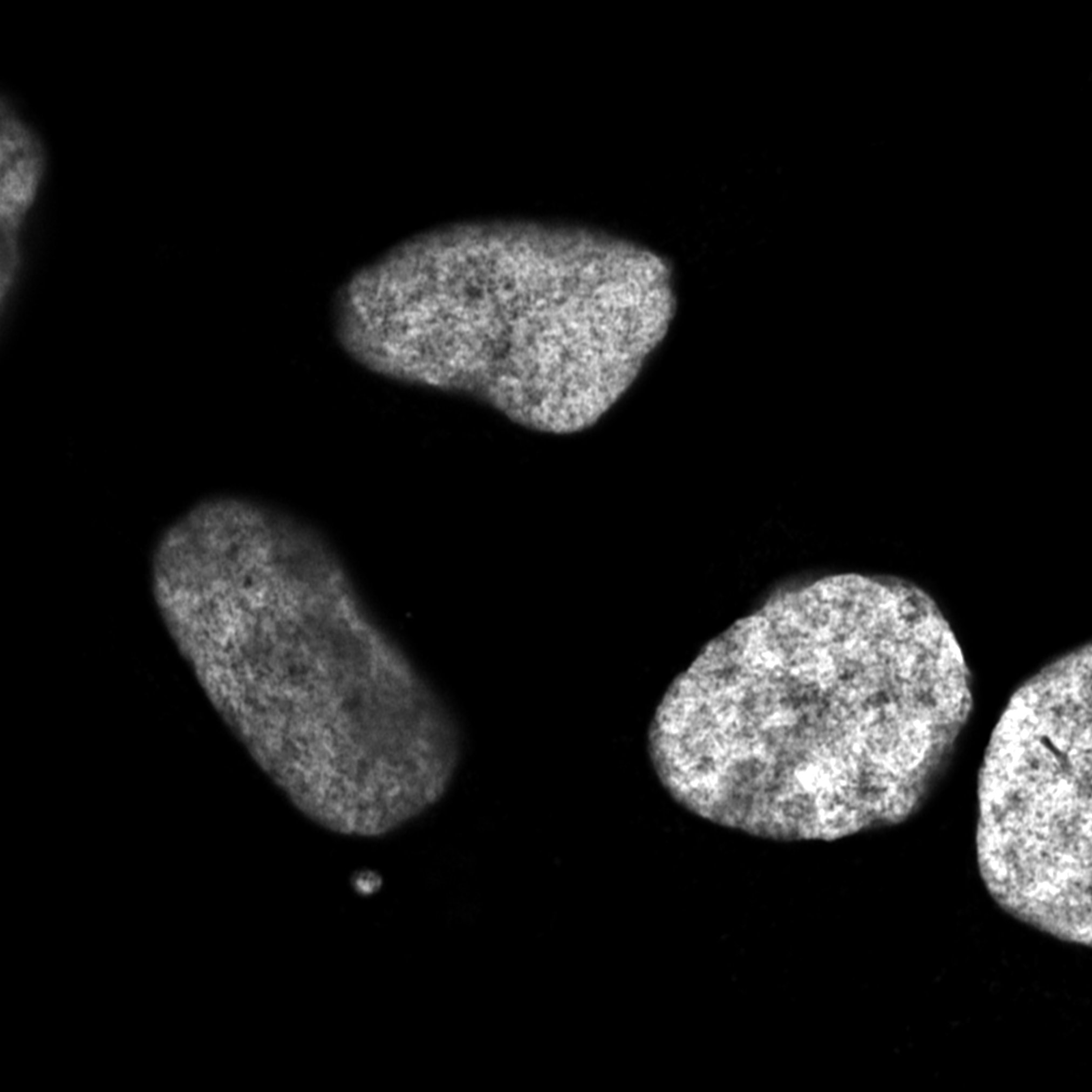

Supplement: Supplementary file 6 — Source data Fig. 3 [file 44318_2024_305_MOESM6_ESM.zip › Figure 3/3G/GOLPH_KO_ctrl_LYSET_GM_3_(Hoechst_C=2)_Airyscan Processing.tiff]

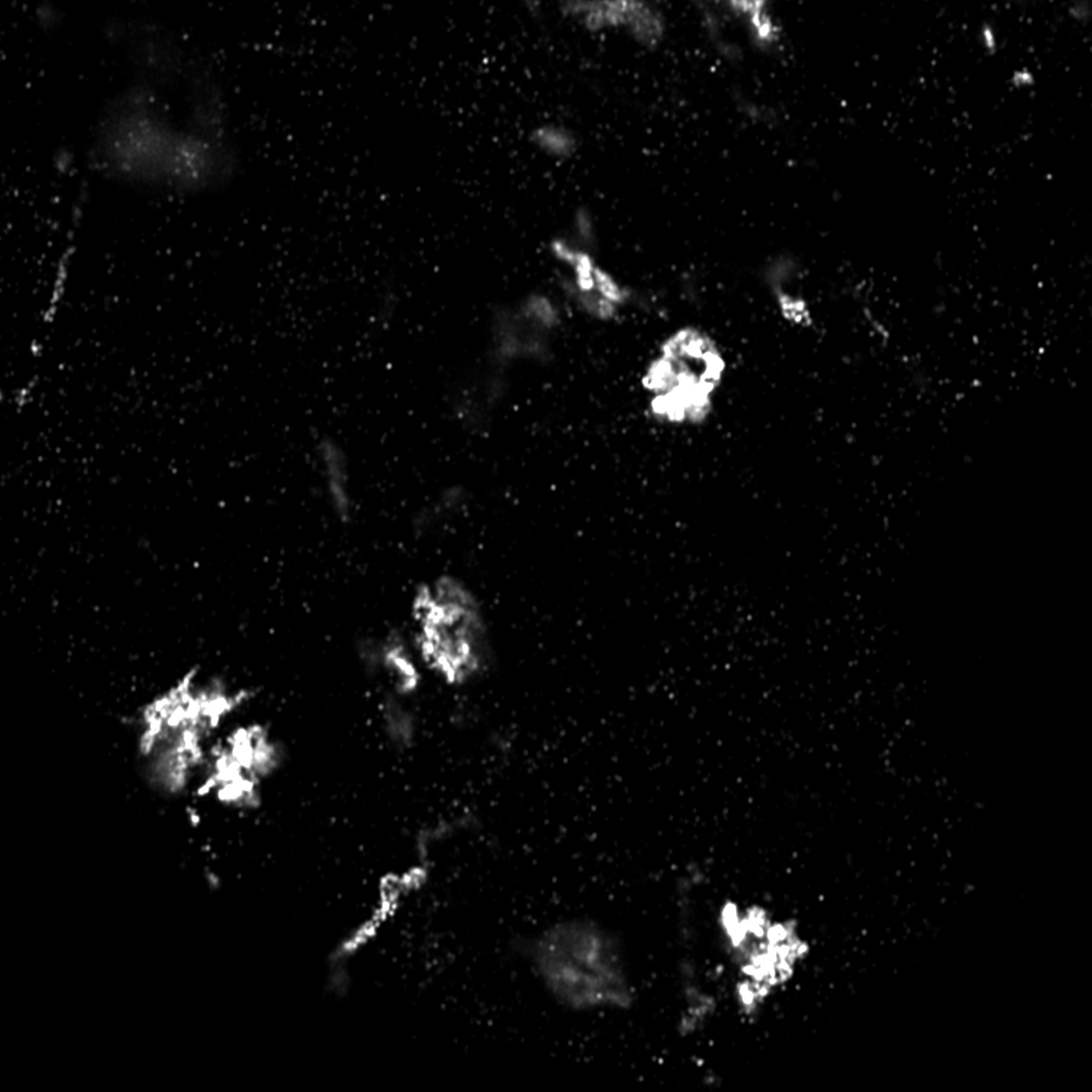

Supplement: Supplementary file 6 — Source data Fig. 3 [file 44318_2024_305_MOESM6_ESM.zip › Figure 3/3G/WT_ctrl_LYSET_GM_3_(LYSET488_C=1))_Airyscan Processing.tiff]

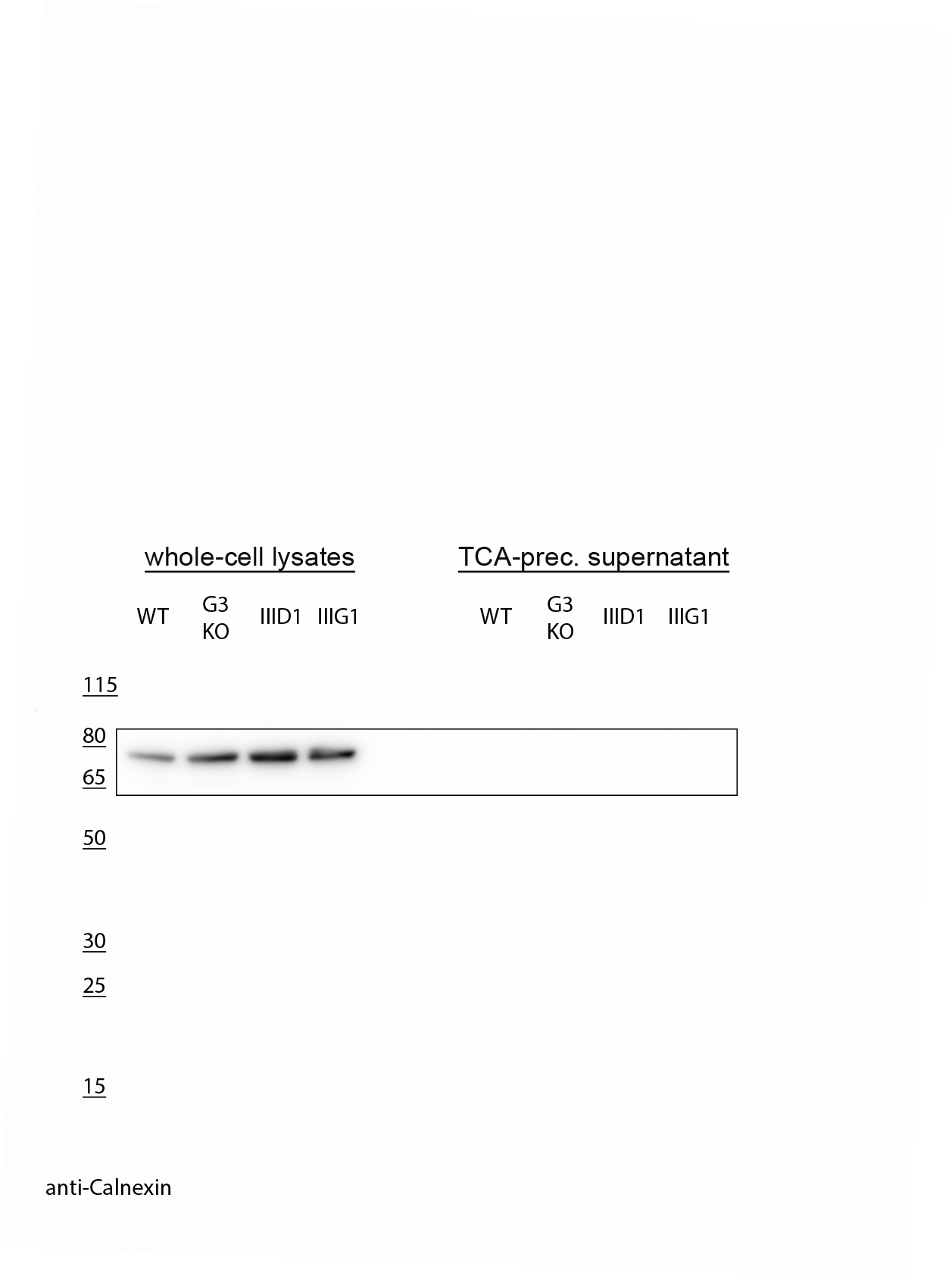

Supplement: Supplementary file 7 — Source data Fig. 4 [file 44318_2024_305_MOESM7_ESM.zip › Figure 4/4E/source data Calnexin for CTSC.tif]

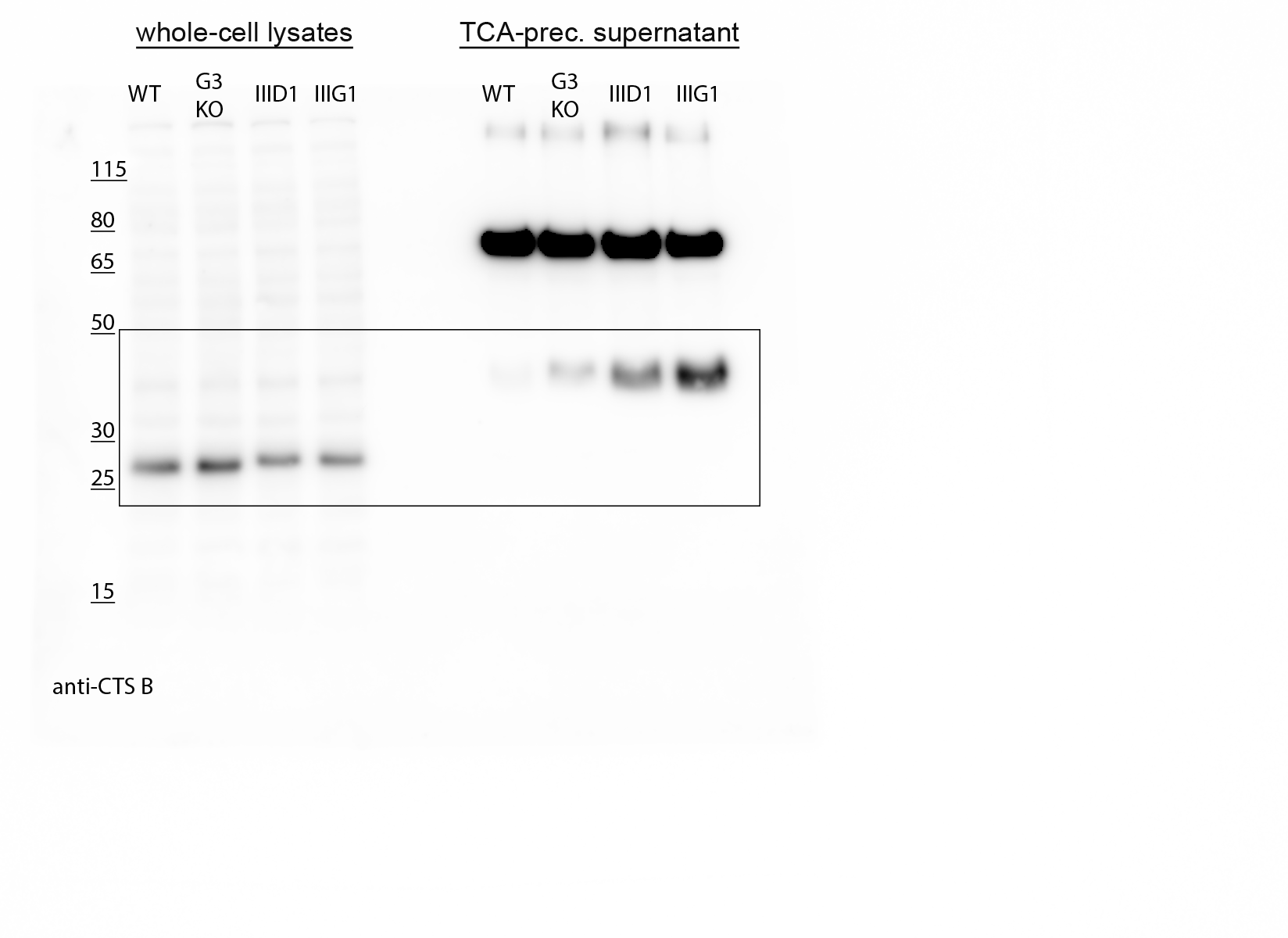

Supplement: Supplementary file 7 — Source data Fig. 4 [file 44318_2024_305_MOESM7_ESM.zip › Figure 4/4E/source data CTSB.tif]

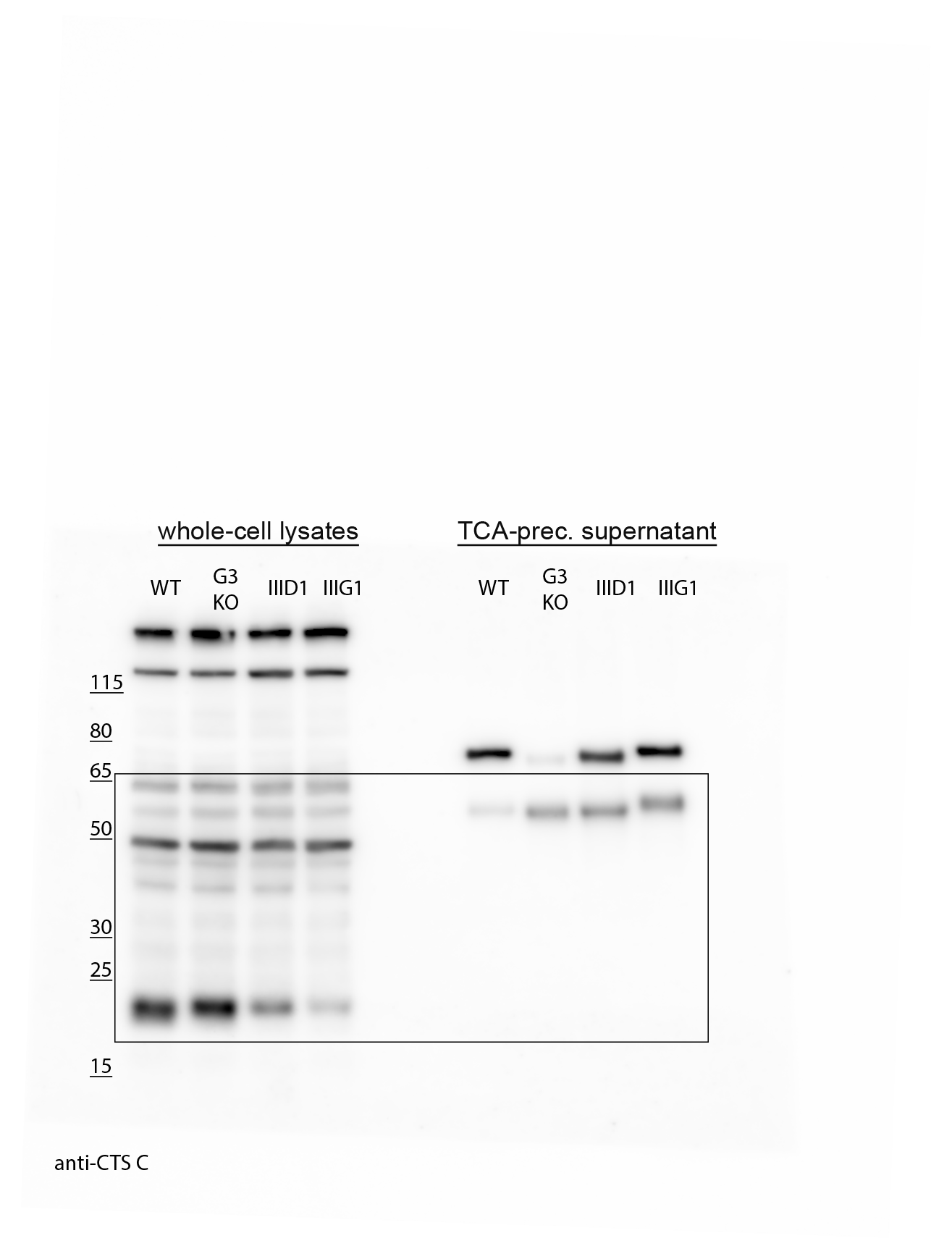

Supplement: Supplementary file 7 — Source data Fig. 4 [file 44318_2024_305_MOESM7_ESM.zip › Figure 4/4E/source data CTSC.tif]

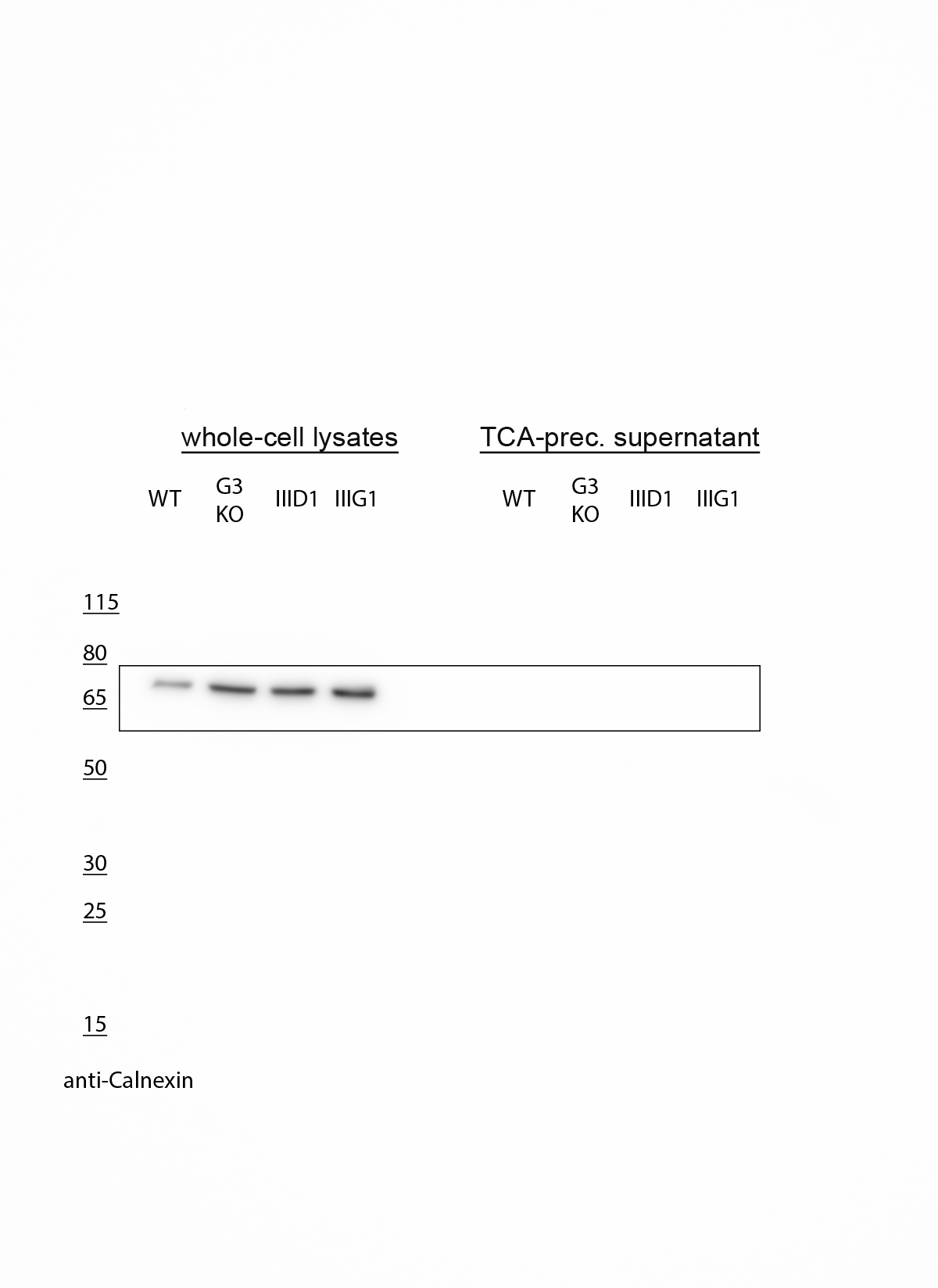

Supplement: Supplementary file 7 — Source data Fig. 4 [file 44318_2024_305_MOESM7_ESM.zip › Figure 4/4E/source data Calnexin for HEX B, GLA.tif]

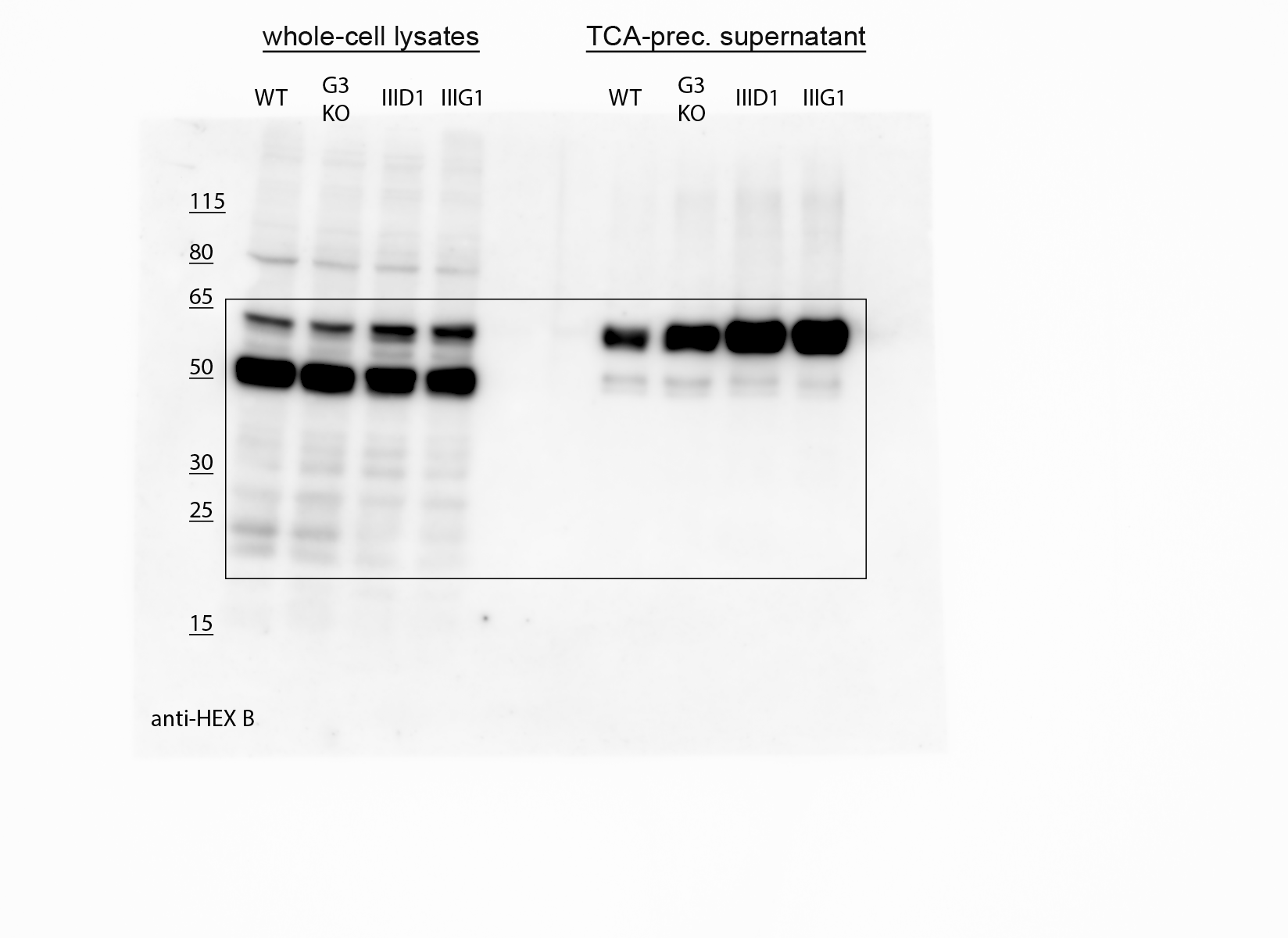

Supplement: Supplementary file 7 — Source data Fig. 4 [file 44318_2024_305_MOESM7_ESM.zip › Figure 4/4E/source data Hex B.tif]

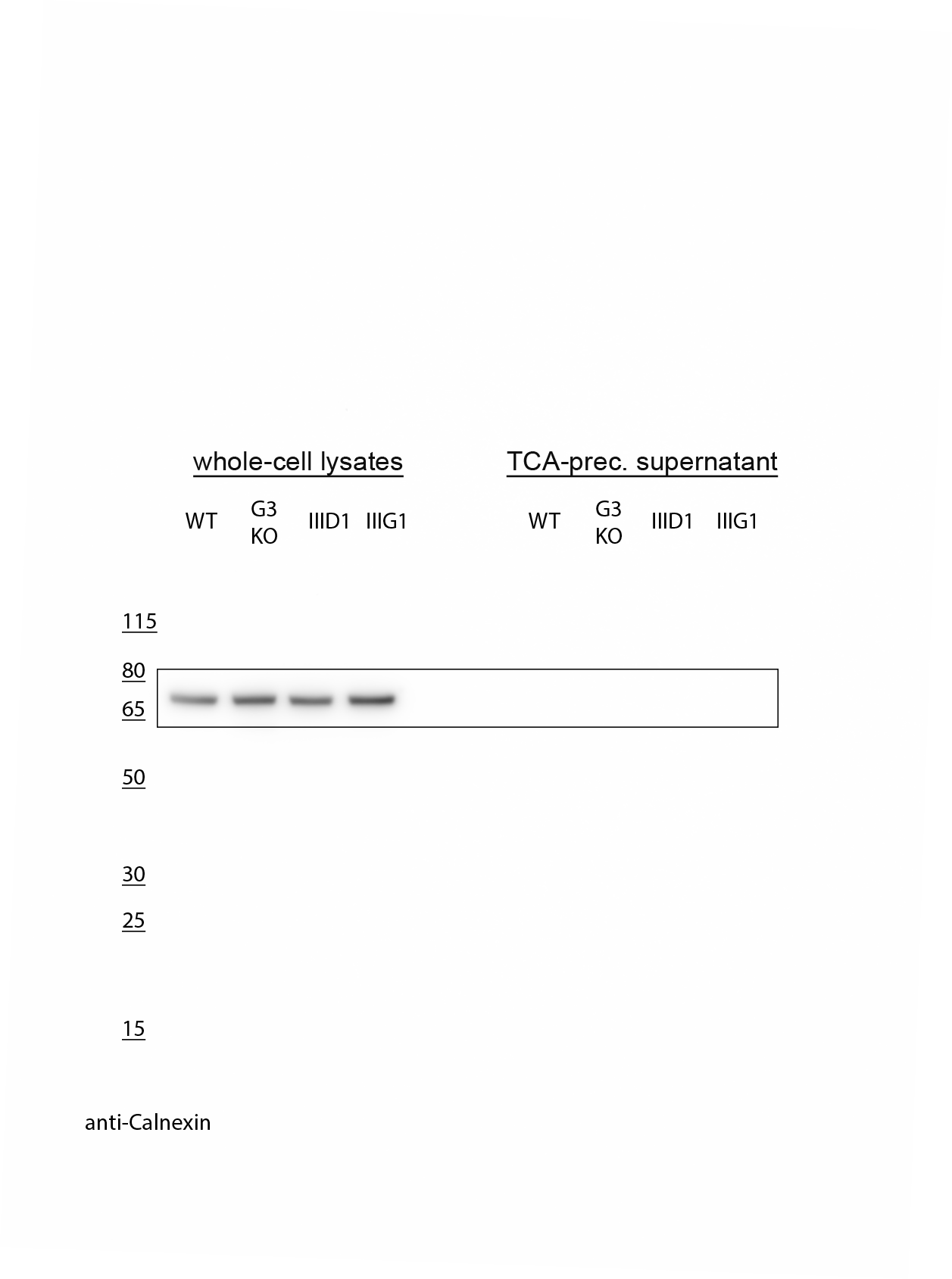

Supplement: Supplementary file 7 — Source data Fig. 4 [file 44318_2024_305_MOESM7_ESM.zip › Figure 4/4E/source data Calnexin for CTS L, CTS B.tif]

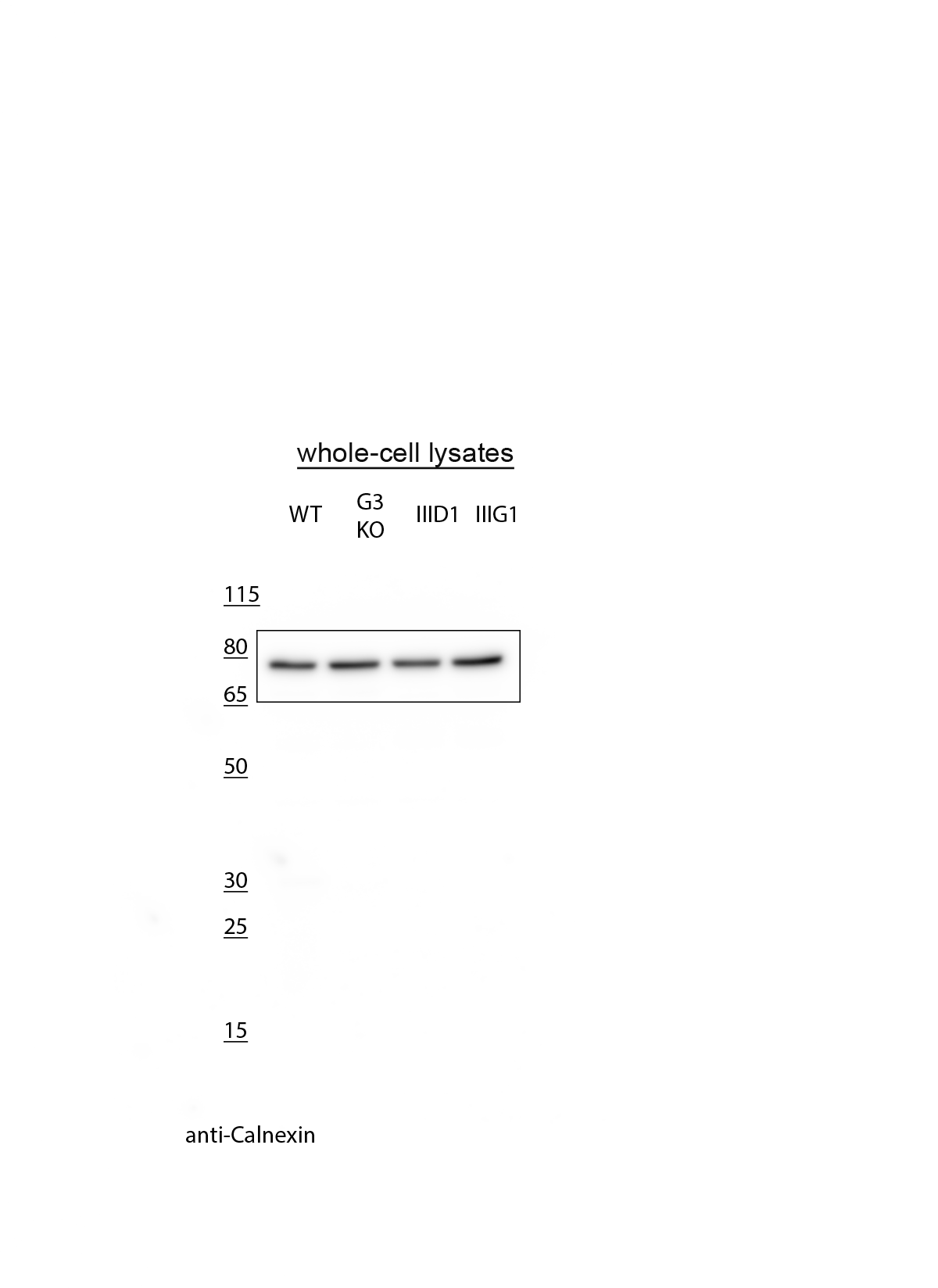

Supplement: Supplementary file 7 — Source data Fig. 4 [file 44318_2024_305_MOESM7_ESM.zip › Figure 4/4E/source data Calnexin for GOLPH3.tif]

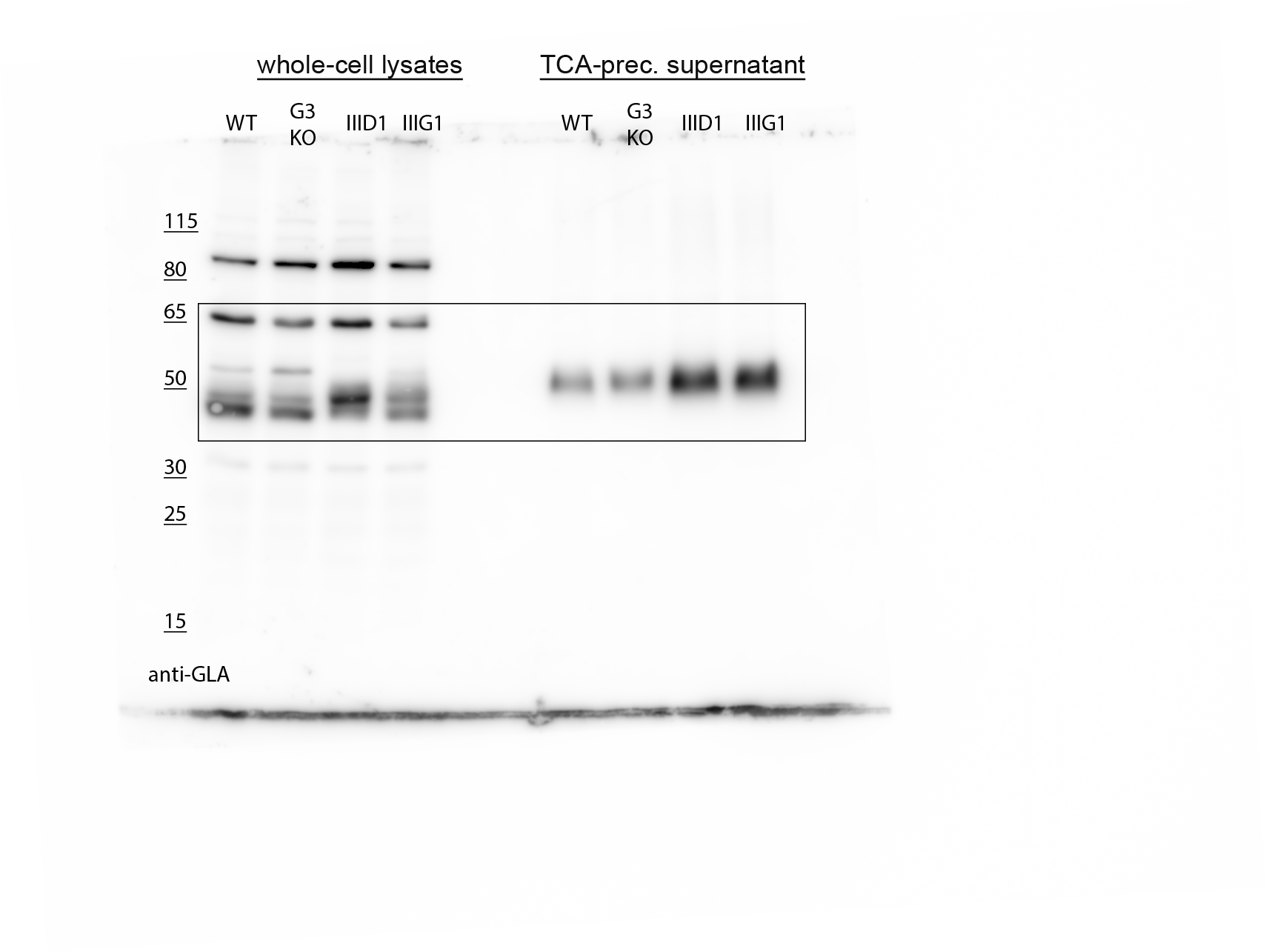

Supplement: Supplementary file 7 — Source data Fig. 4 [file 44318_2024_305_MOESM7_ESM.zip › Figure 4/4E/source data GLA.tif]

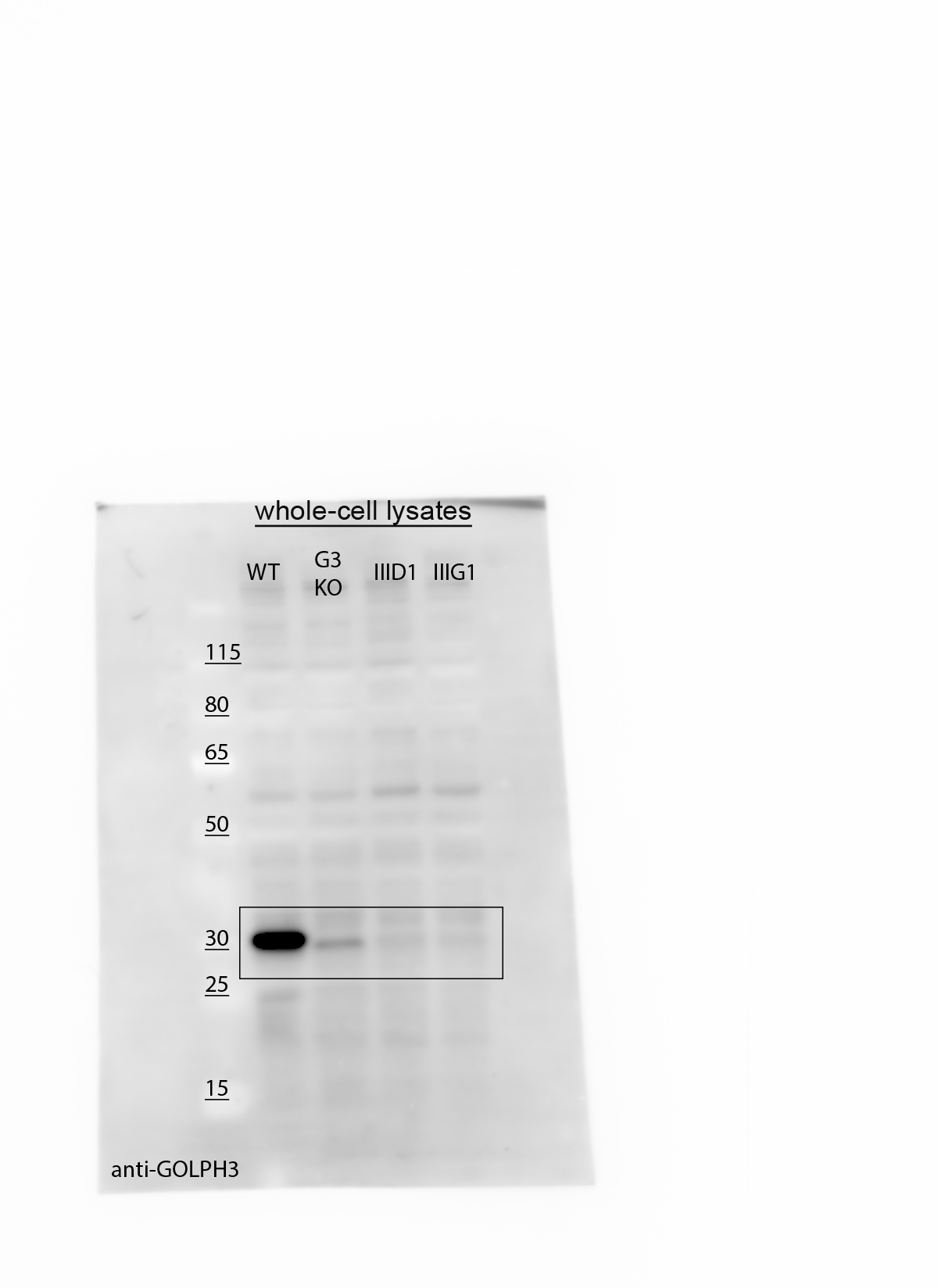

Supplement: Supplementary file 7 — Source data Fig. 4 [file 44318_2024_305_MOESM7_ESM.zip › Figure 4/4E/source data GOLPH3.tif]

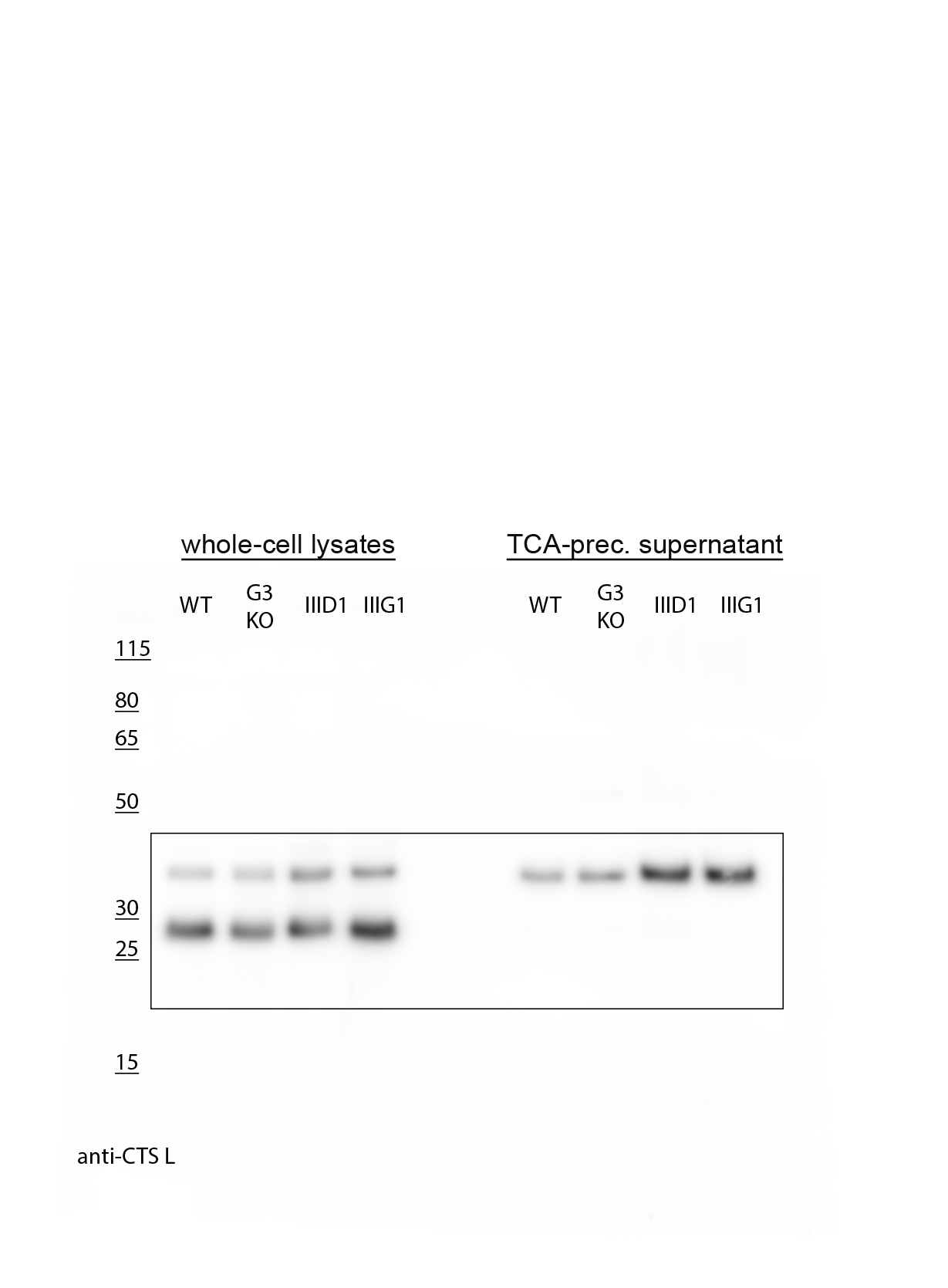

Supplement: Supplementary file 7 — Source data Fig. 4 [file 44318_2024_305_MOESM7_ESM.zip › Figure 4/4E/source data CTSL.tif]

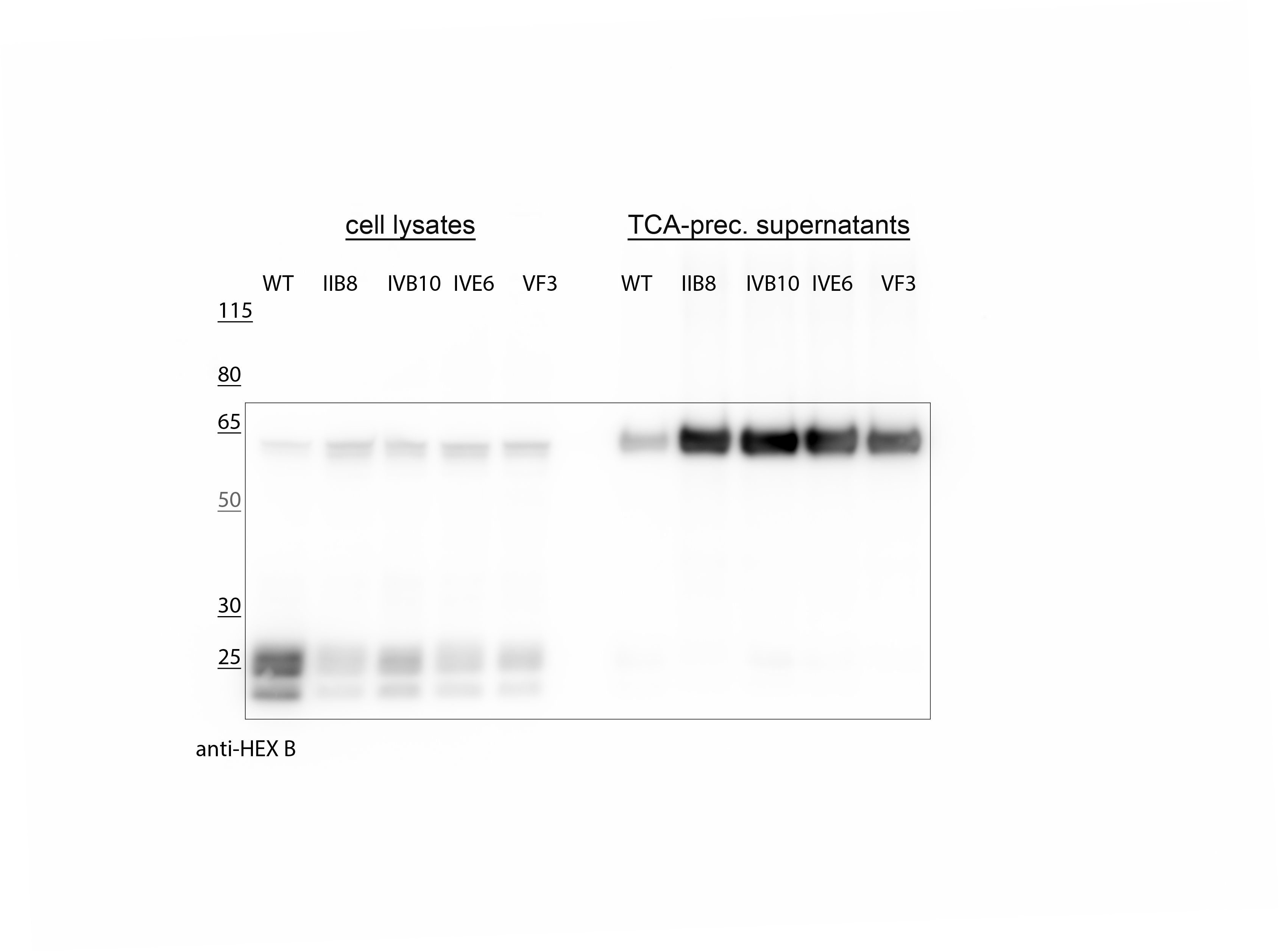

Supplement: Supplementary file 7 — Source data Fig. 4 [file 44318_2024_305_MOESM7_ESM.zip › Figure 4/4B/source data HEX B 20240828_145017-01_Ch_Chemi.tif]

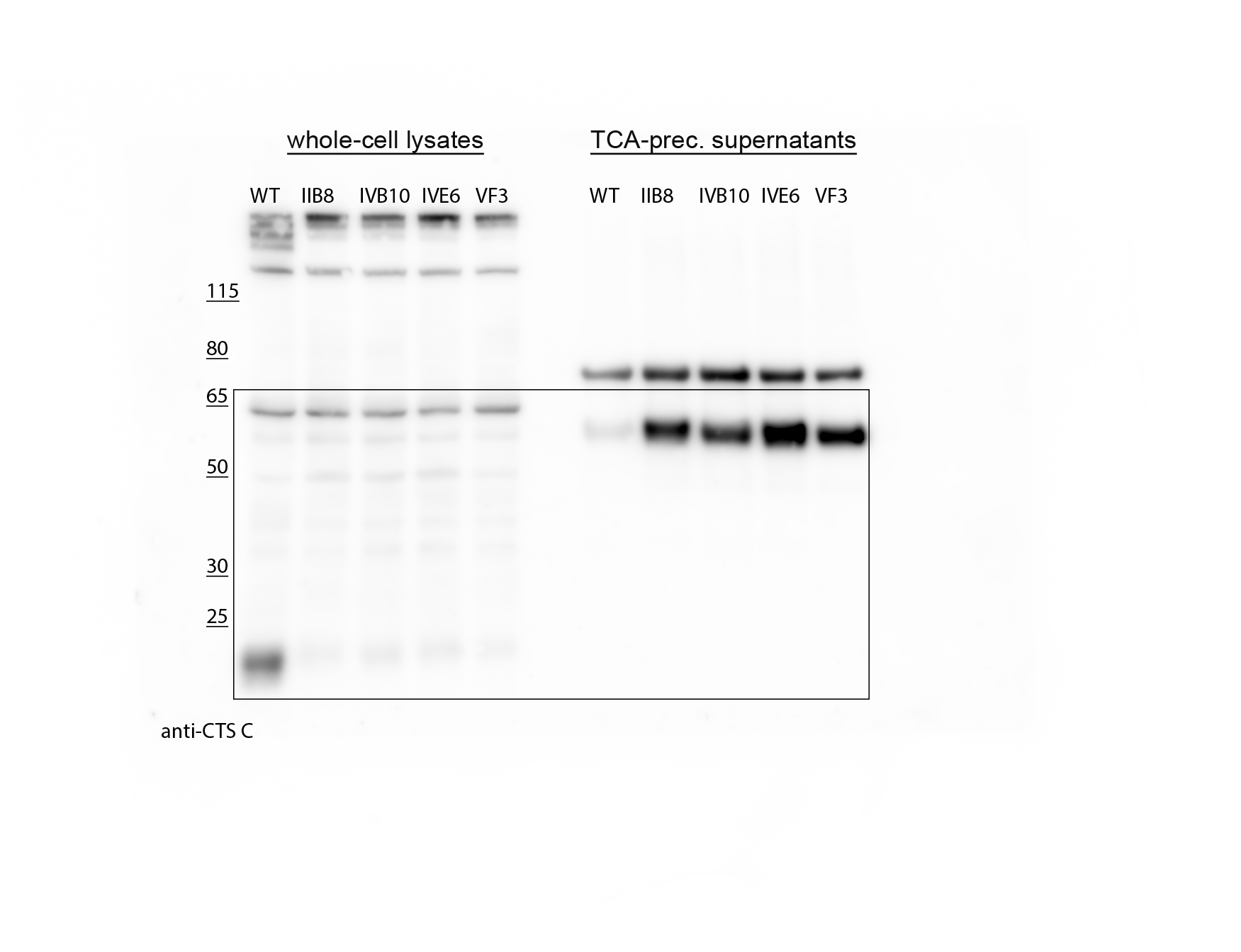

Supplement: Supplementary file 7 — Source data Fig. 4 [file 44318_2024_305_MOESM7_ESM.zip › Figure 4/4B/source data CTSC 20240904_140544-03_Ch_Chemi.tif]

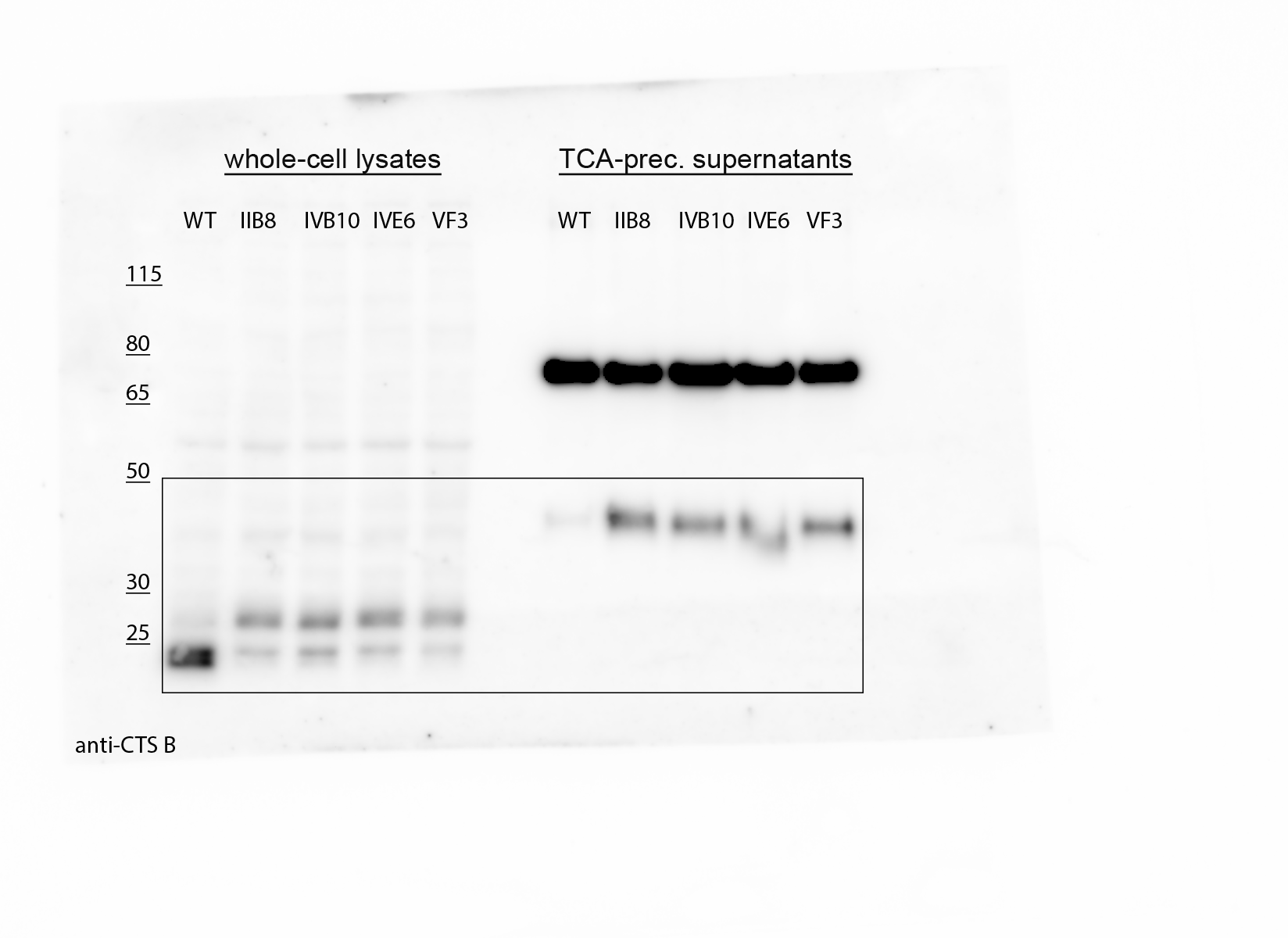

Supplement: Supplementary file 7 — Source data Fig. 4 [file 44318_2024_305_MOESM7_ESM.zip › Figure 4/4B/source data CTSB 20240904_135754-12_Ch_Chemi.tif]

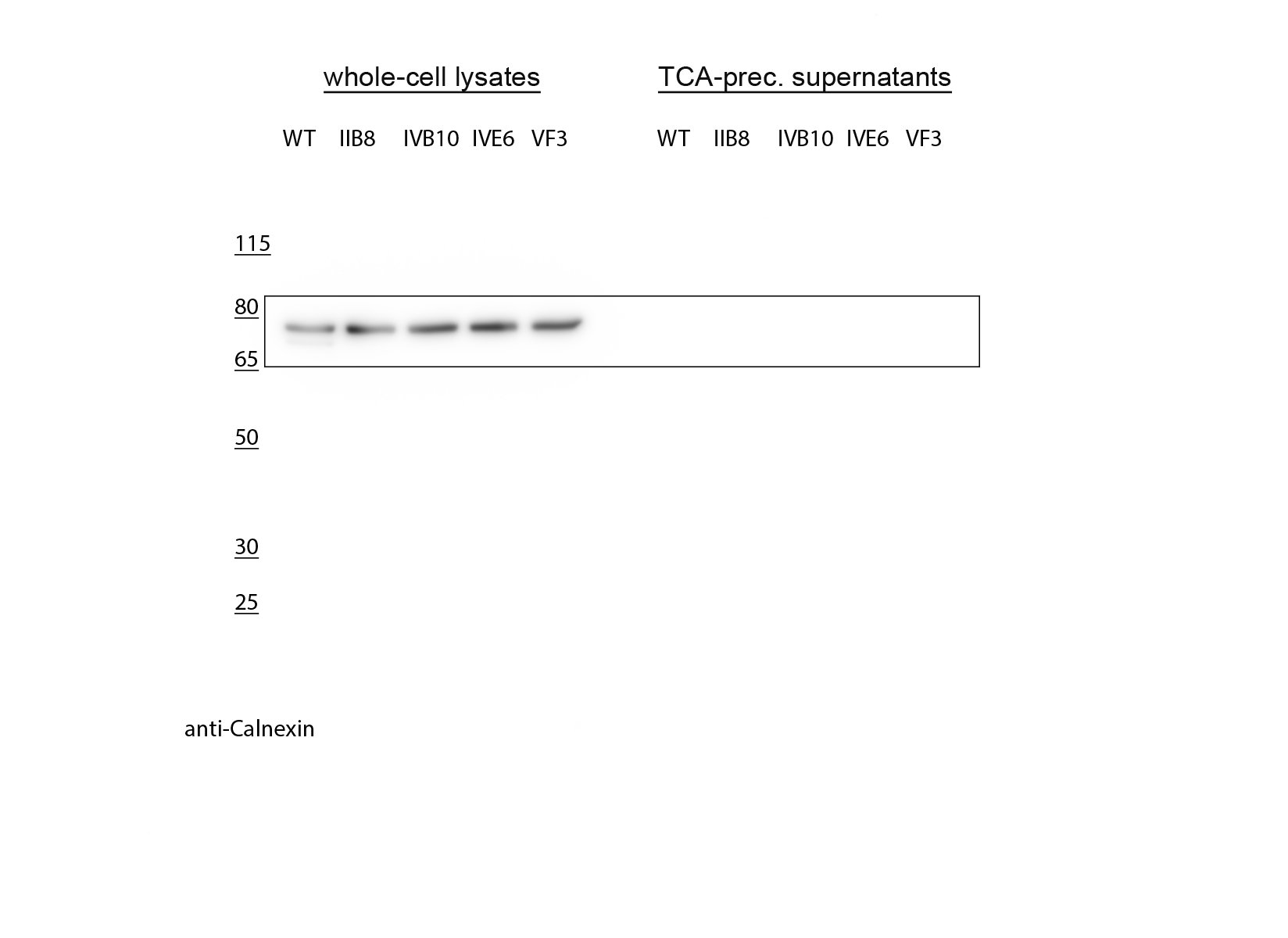

Supplement: Supplementary file 7 — Source data Fig. 4 [file 44318_2024_305_MOESM7_ESM.zip › Figure 4/4B/source data Calnexin for CTS C, GOLPH3 20240906_140342-02_Ch_Chemi.tif]

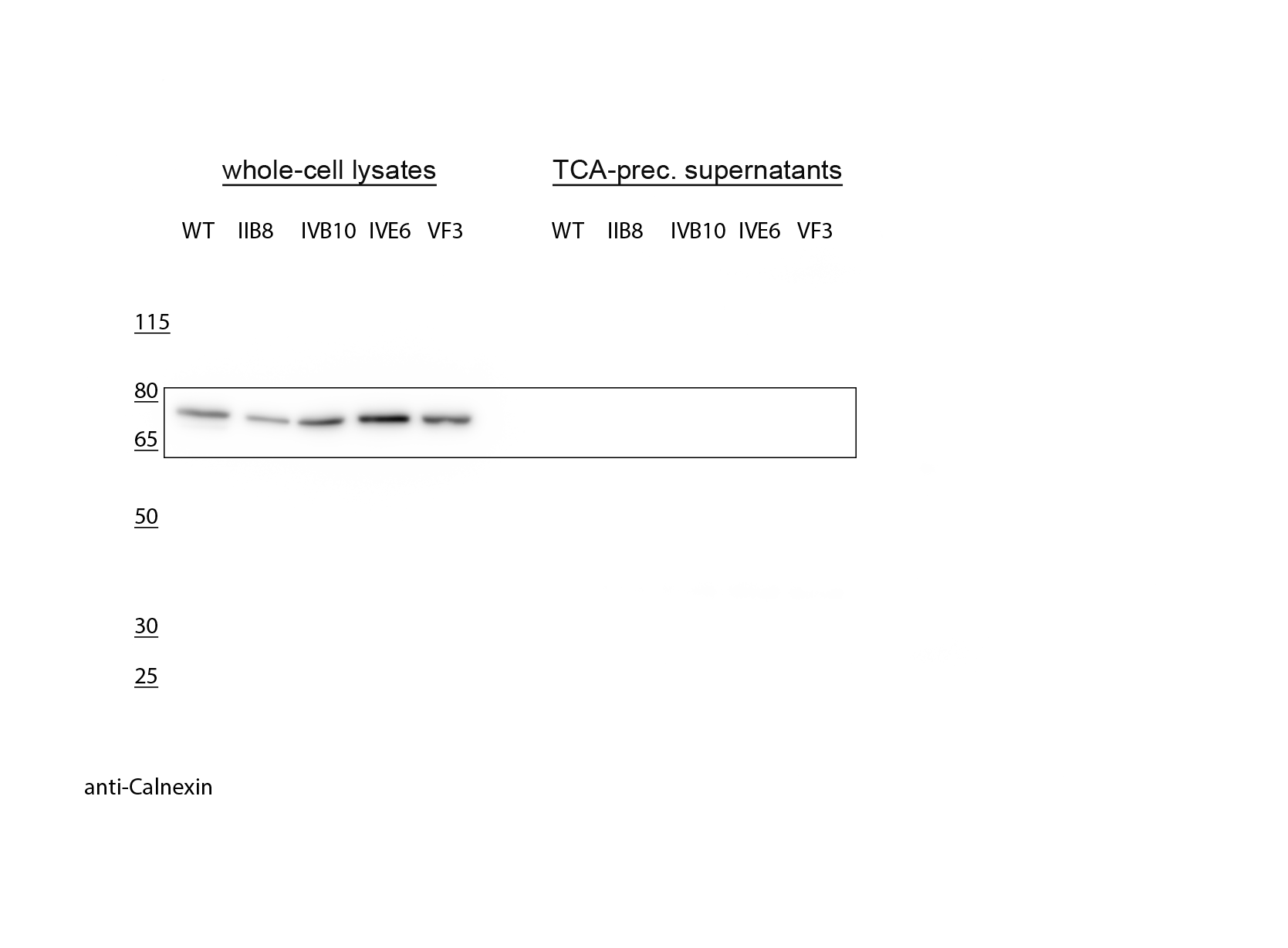

Supplement: Supplementary file 7 — Source data Fig. 4 [file 44318_2024_305_MOESM7_ESM.zip › Figure 4/4B/source data Calnexin for HEX B, CTS B, CTS L 20240906_141010-02_Ch_Chemi.tif]

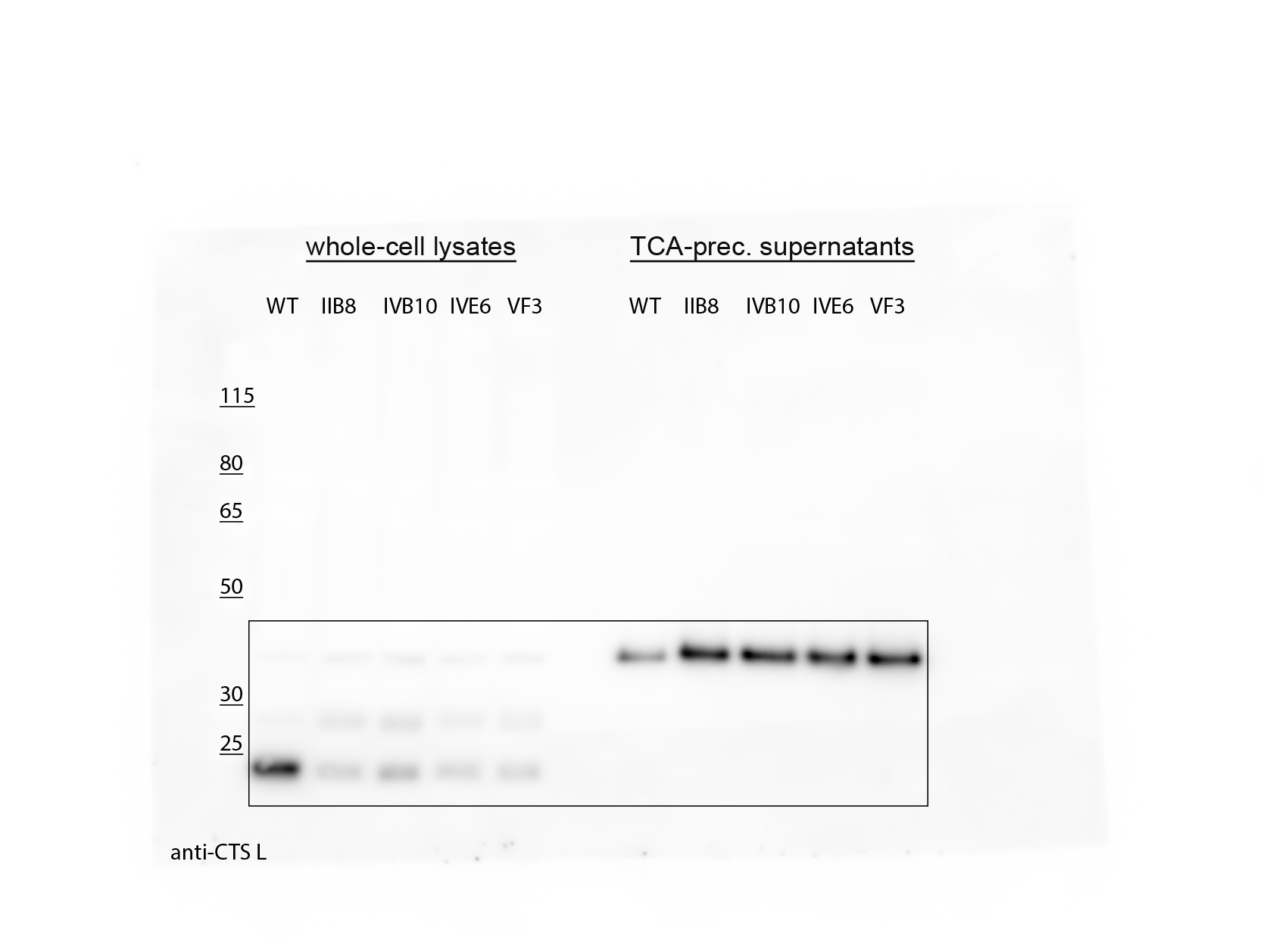

Supplement: Supplementary file 7 — Source data Fig. 4 [file 44318_2024_305_MOESM7_ESM.zip › Figure 4/4B/source data CTSL 20240905_142309-02_Ch_Chemi.tif]

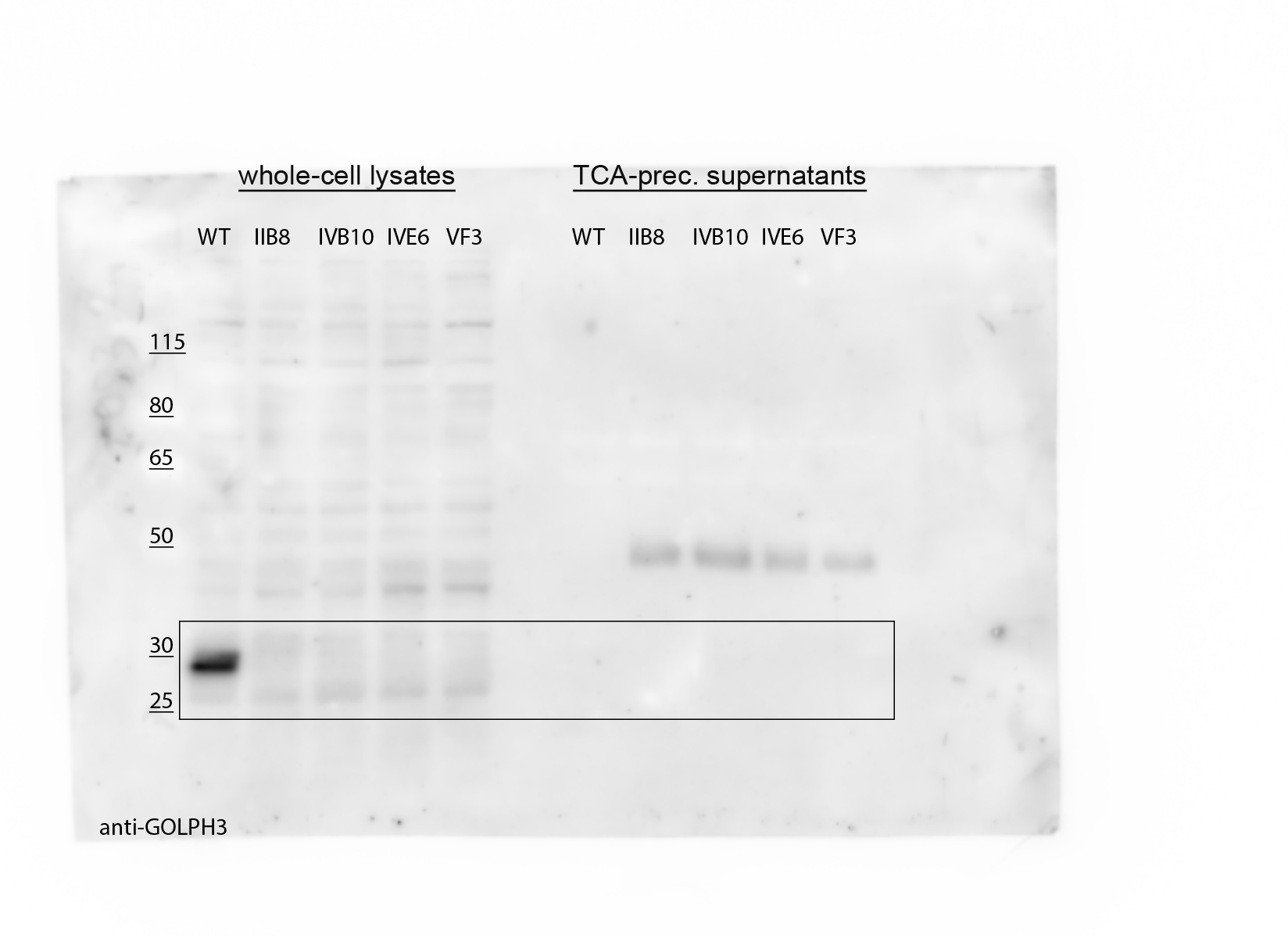

Supplement: Supplementary file 7 — Source data Fig. 4 [file 44318_2024_305_MOESM7_ESM.zip › Figure 4/4B/source data GOLPH3 20240905_141726-03_Ch_Chemi.tif]

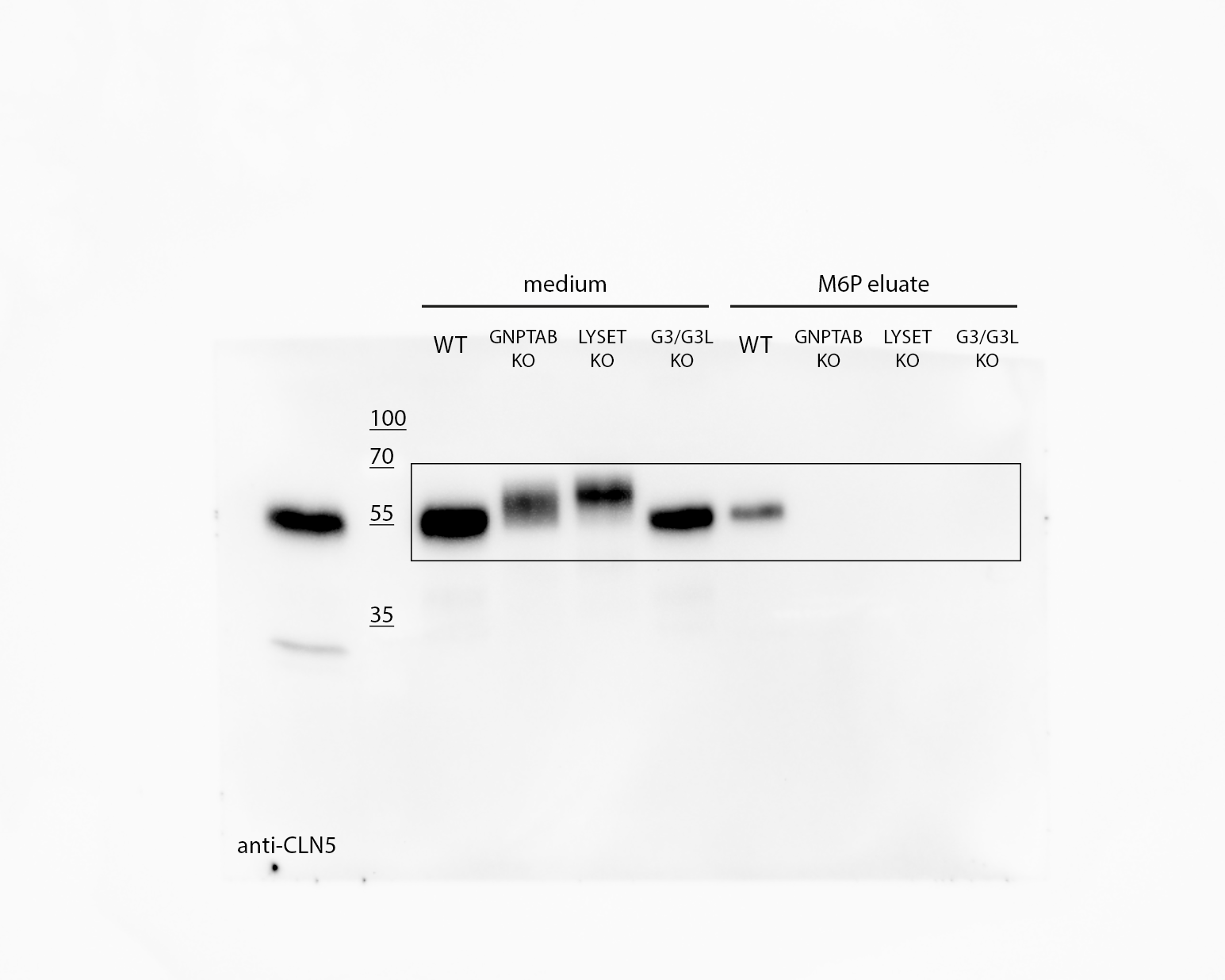

Supplement: Supplementary file 7 — Source data Fig. 4 [file 44318_2024_305_MOESM7_ESM.zip › Figure 4/4A/source data CLN5 (HEK).tif]

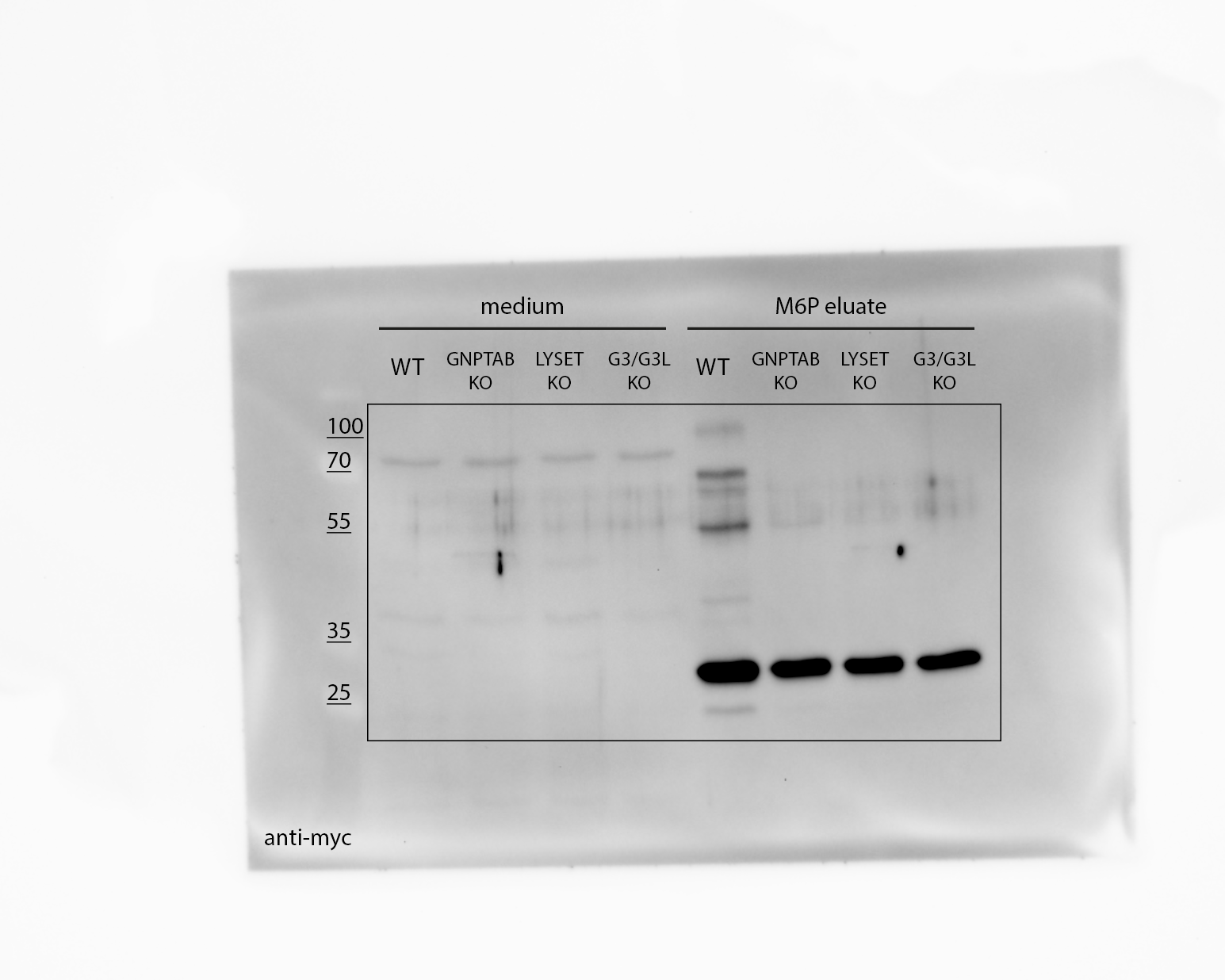

Supplement: Supplementary file 7 — Source data Fig. 4 [file 44318_2024_305_MOESM7_ESM.zip › Figure 4/4A/source data myc (HAP1).tif]

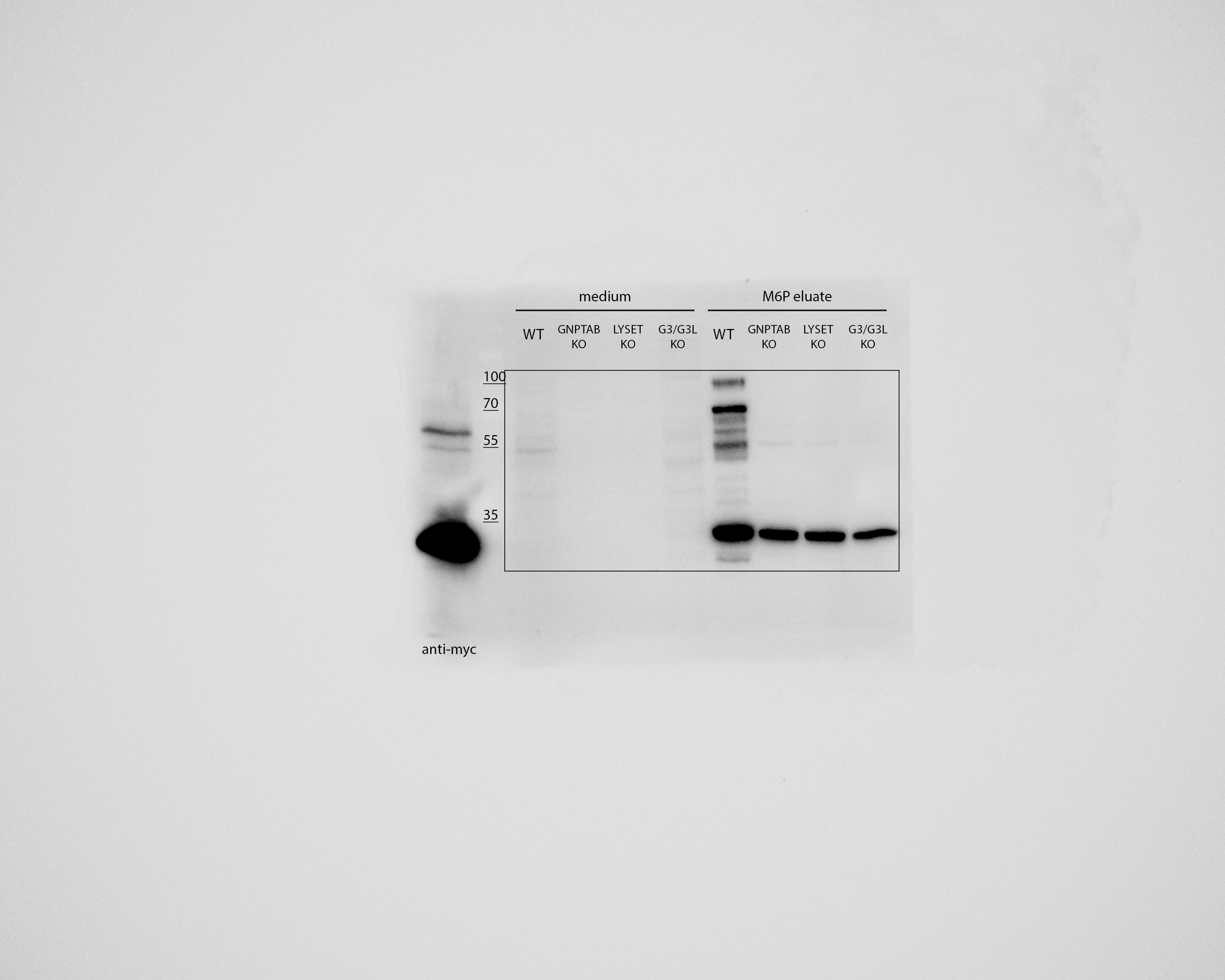

Supplement: Supplementary file 7 — Source data Fig. 4 [file 44318_2024_305_MOESM7_ESM.zip › Figure 4/4A/source data myc (HEK).tif]

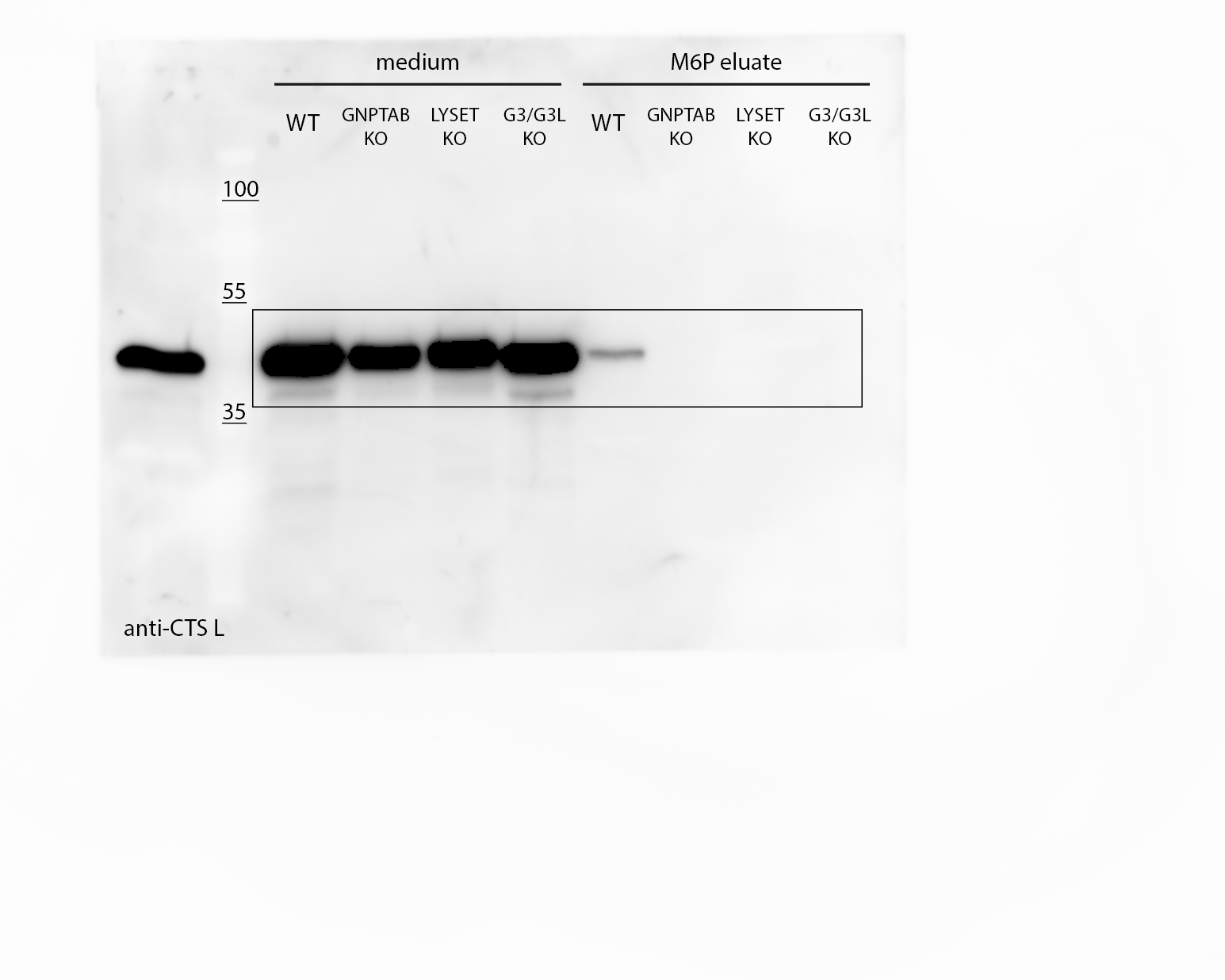

Supplement: Supplementary file 7 — Source data Fig. 4 [file 44318_2024_305_MOESM7_ESM.zip › Figure 4/4A/source data CTS L.tif]

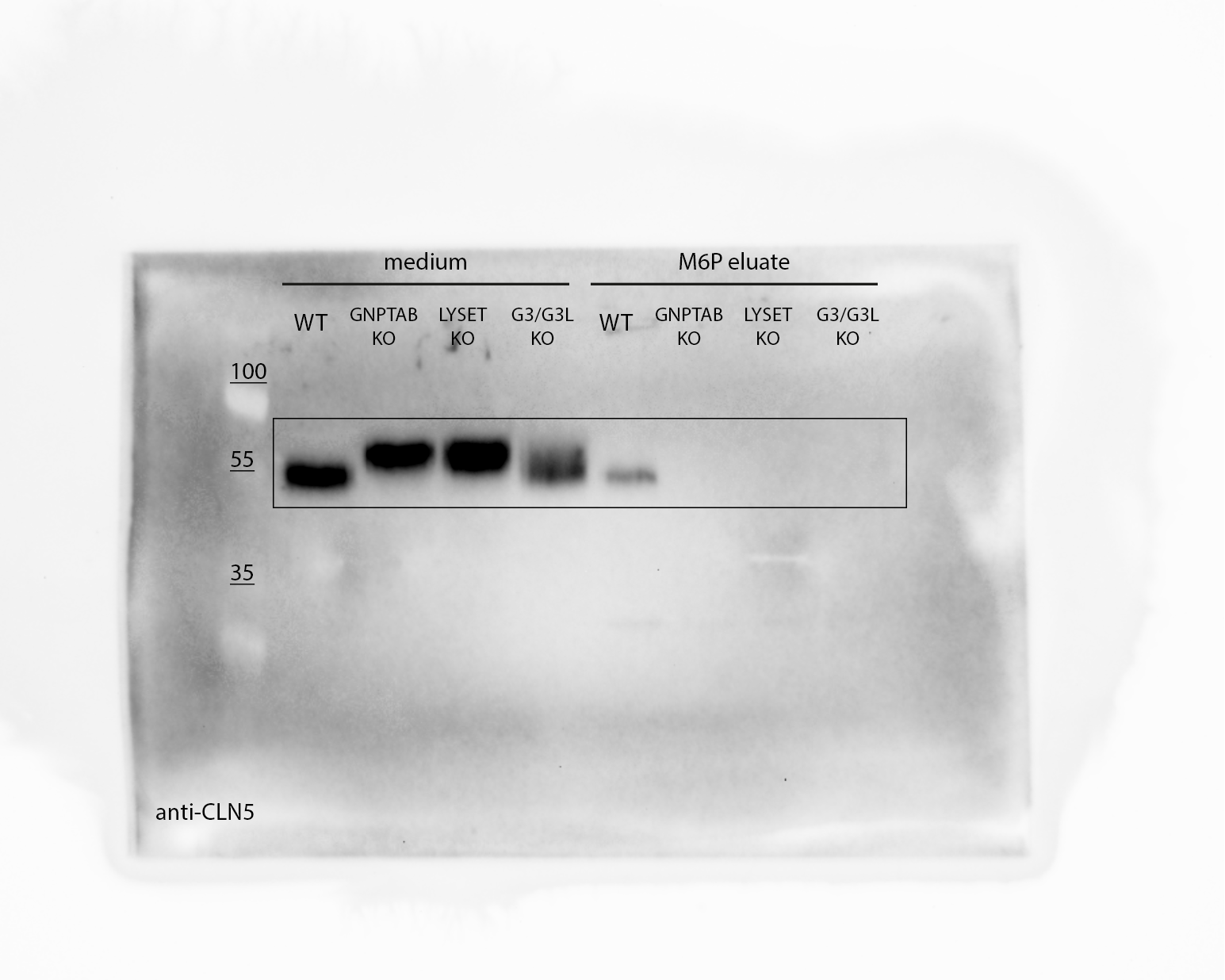

Supplement: Supplementary file 7 — Source data Fig. 4 [file 44318_2024_305_MOESM7_ESM.zip › Figure 4/4A/source data CLN5 (HAP).tif]

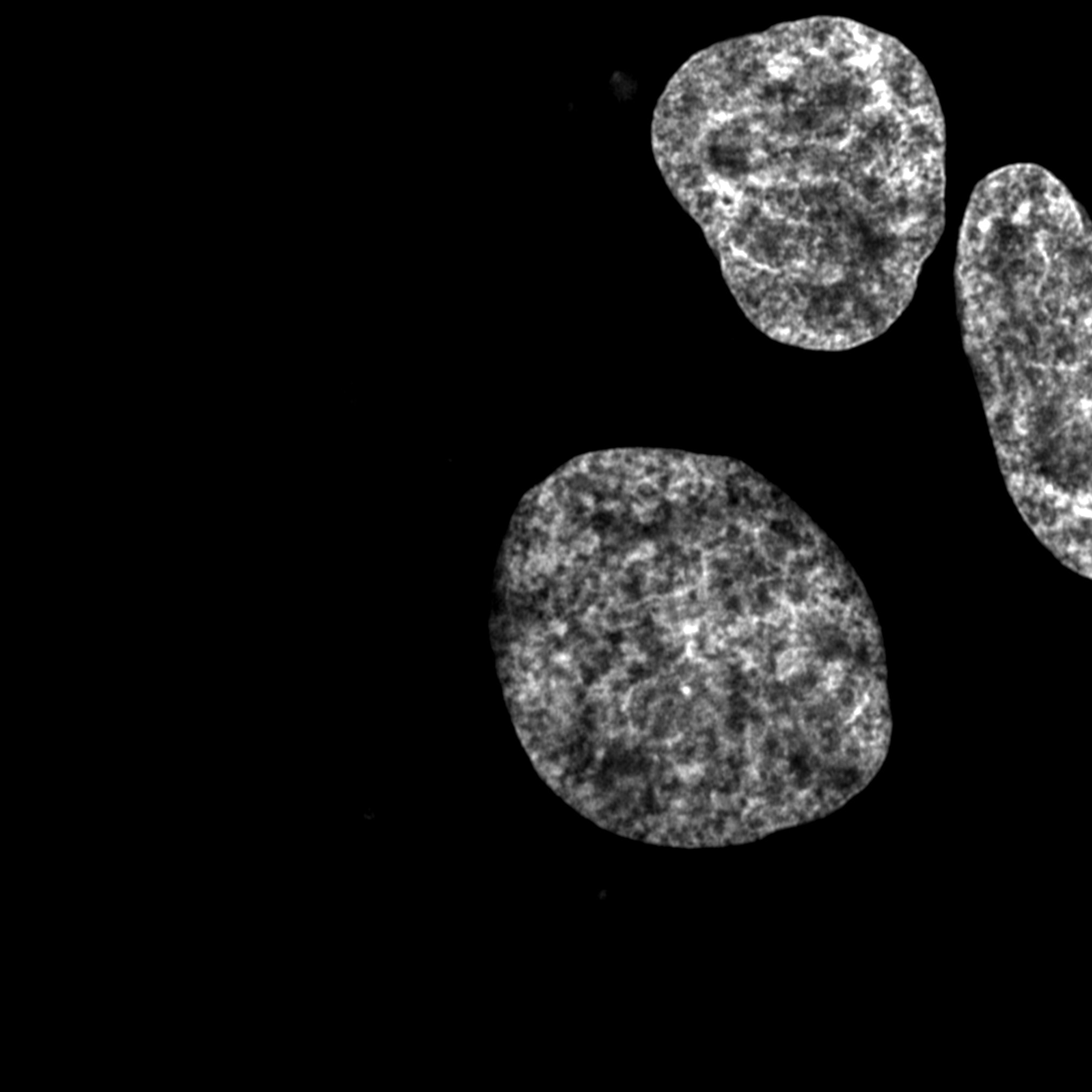

Supplement: Supplementary file 8 — Source data Fig. 5 [file 44318_2024_305_MOESM8_ESM.zip › Figure 5/5G/GOLPH KO_PI_LYSET_LAMP_6_(Hoechst_C=2)Airyscan Processing.tiff]

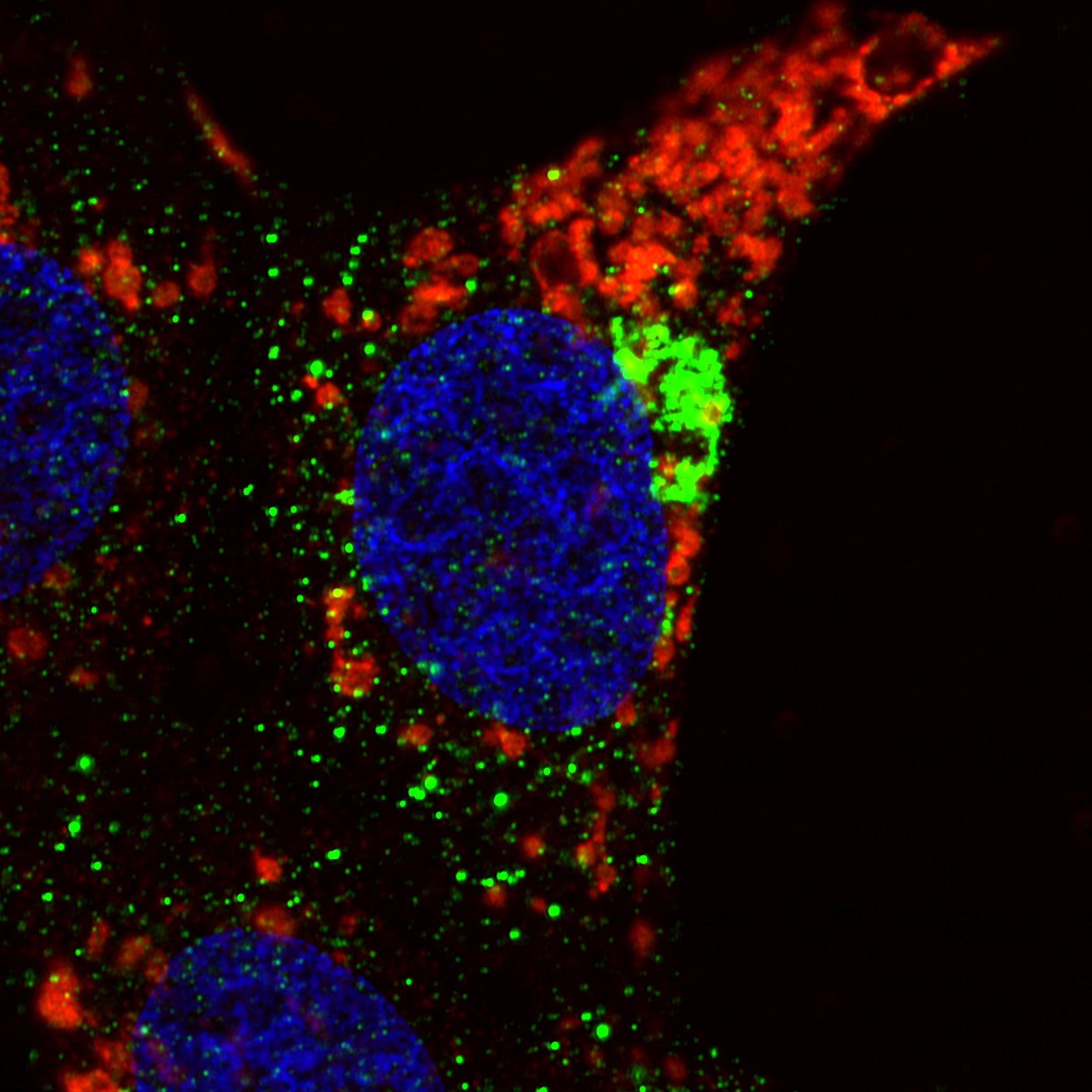

Supplement: Supplementary file 8 — Source data Fig. 5 [file 44318_2024_305_MOESM8_ESM.zip › Figure 5/5G/WT_PI_LYSET_LAMP_3_(merge)_Airyscan Processing.tiff]

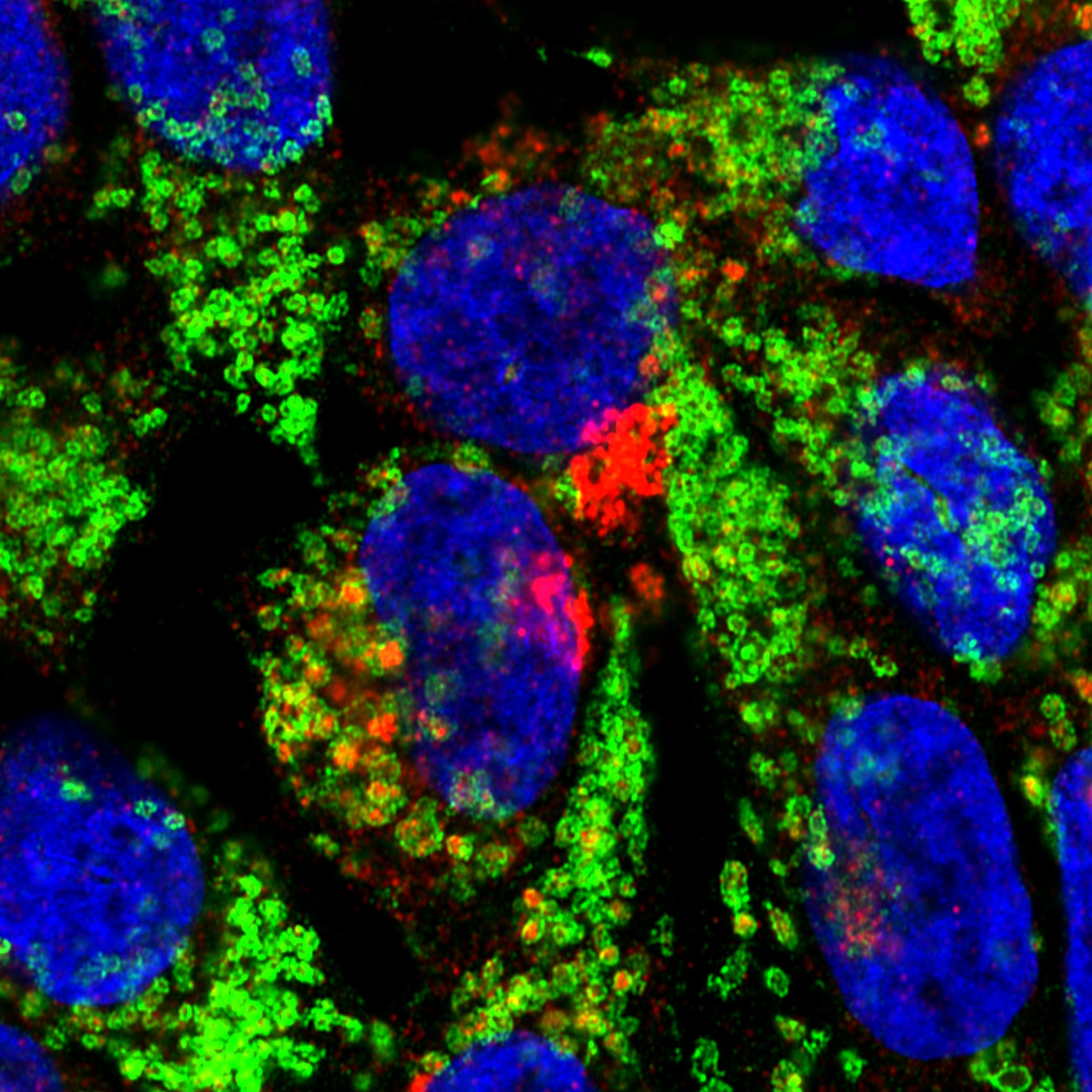

Supplement: Supplementary file 8 — Source data Fig. 5 [file 44318_2024_305_MOESM8_ESM.zip › Figure 5/5G/WT_PI_LAMP_PT_1_(merge)_Airyscan Processing.tif]

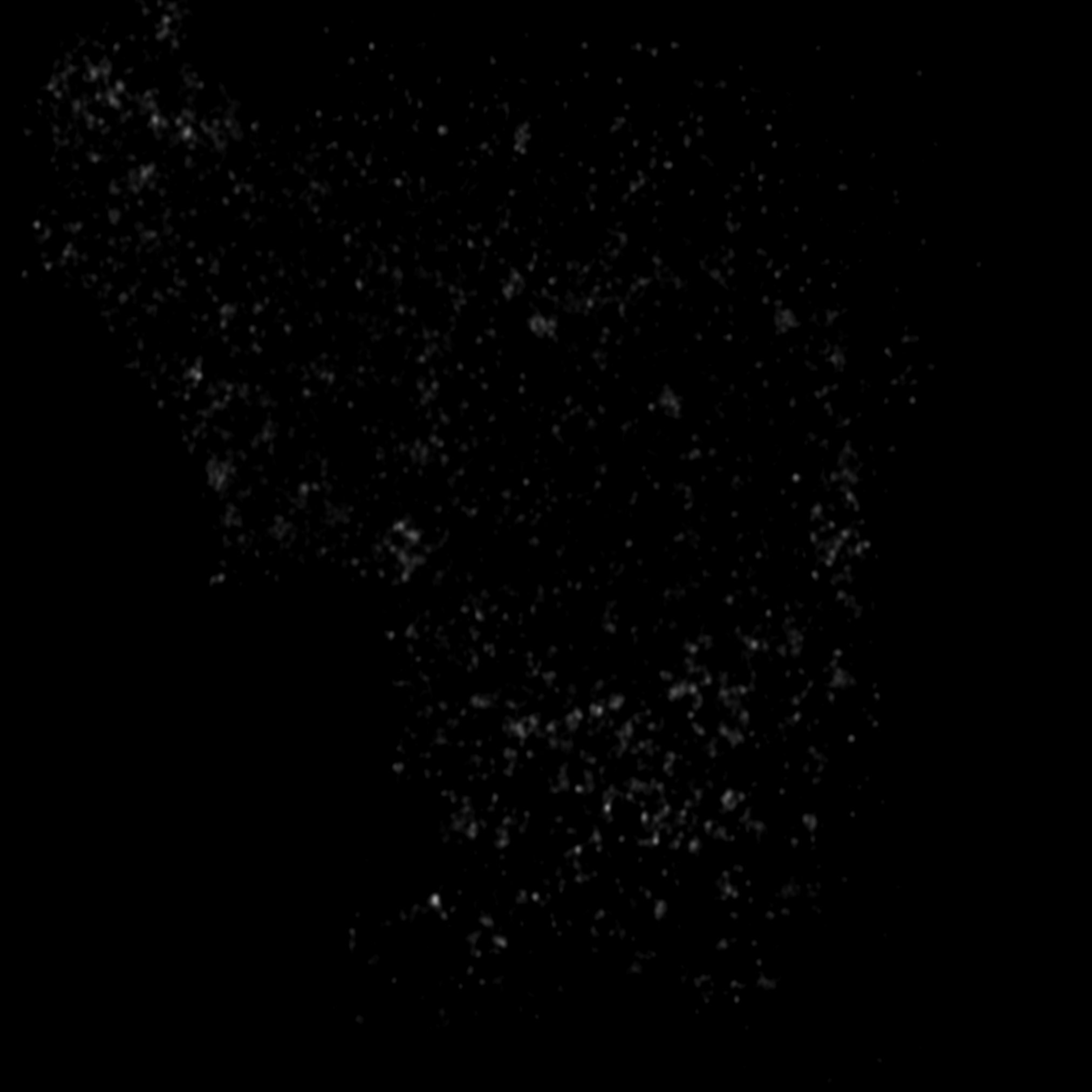

Supplement: Supplementary file 8 — Source data Fig. 5 [file 44318_2024_305_MOESM8_ESM.zip › Figure 5/5G/GOLPH_KO_ctrl_LAMP_PT_2_(PT594_C=0)_Airyscan Processing.tif]

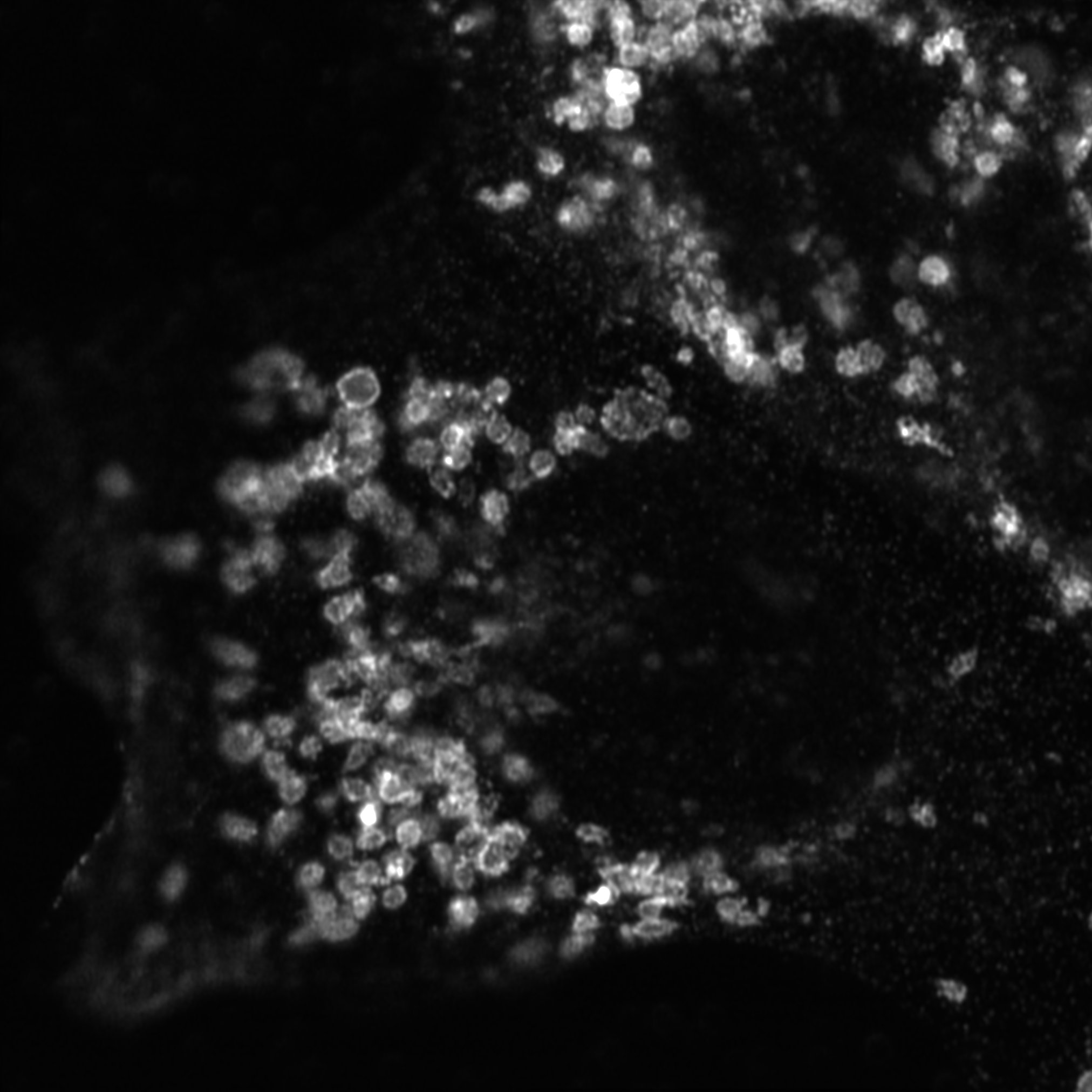

Supplement: Supplementary file 8 — Source data Fig. 5 [file 44318_2024_305_MOESM8_ESM.zip › Figure 5/5G/GOLPH KO_PI_LYSET_LAMP_6_(LAMP594_C=0)_Airyscan Processing.tiff]

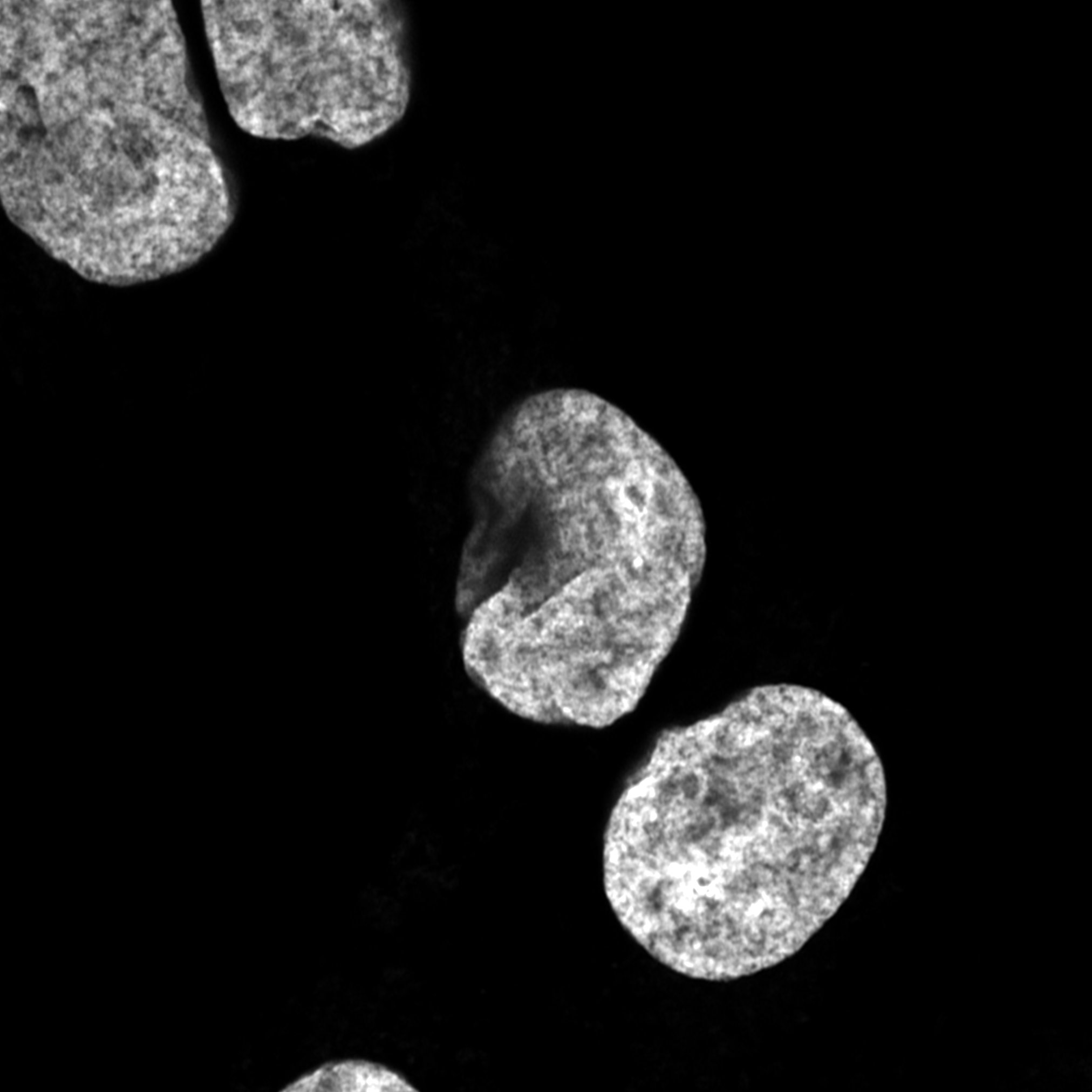

Supplement: Supplementary file 8 — Source data Fig. 5 [file 44318_2024_305_MOESM8_ESM.zip › Figure 5/5G/WT_ctrl_LAMP_PT_2_(Hoechst_C=2)_Airyscan Processing.tif]

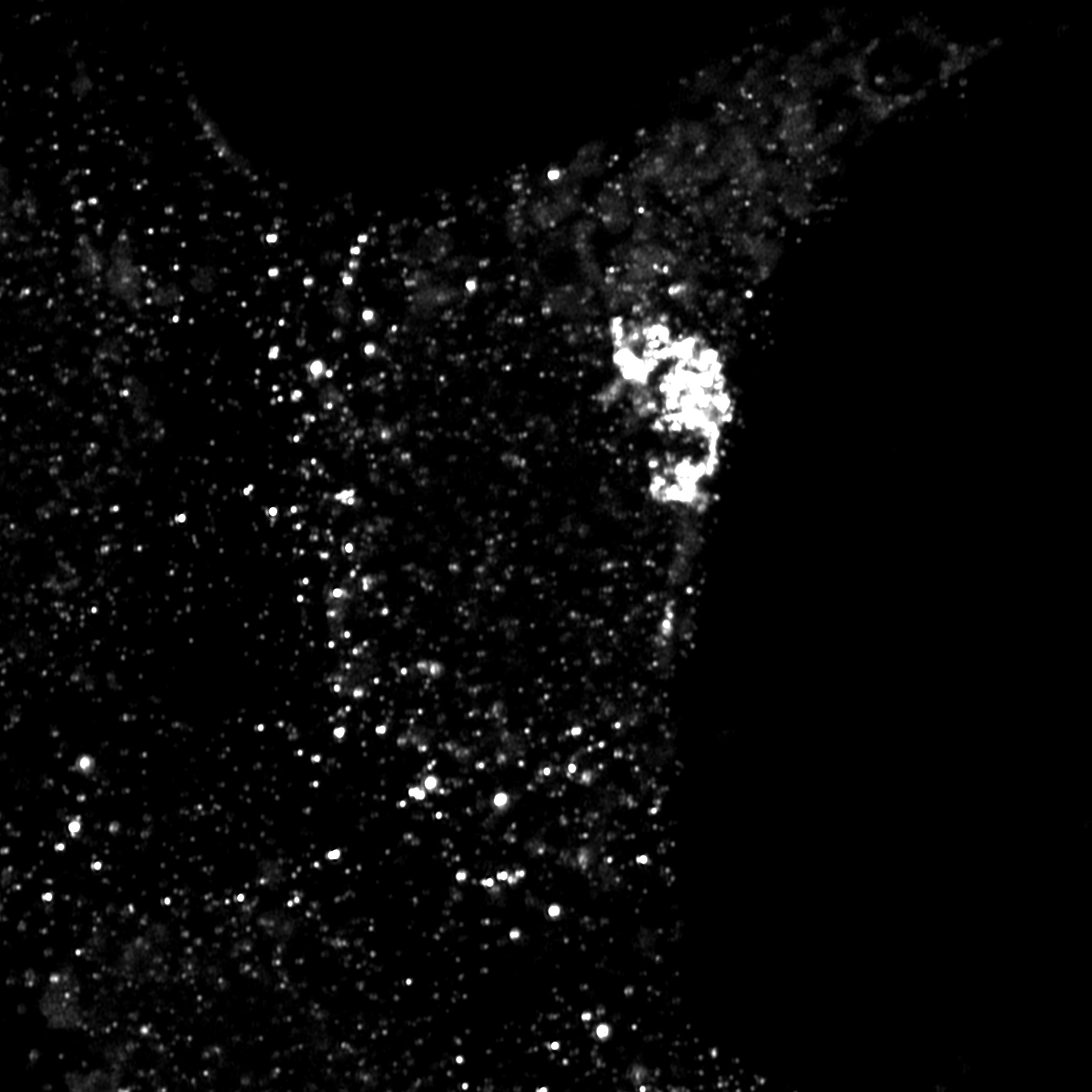

Supplement: Supplementary file 8 — Source data Fig. 5 [file 44318_2024_305_MOESM8_ESM.zip › Figure 5/5G/WT_PI_LYSET_LAMP_3_(LYSET_C=1)_Airyscan Processing.tiff]

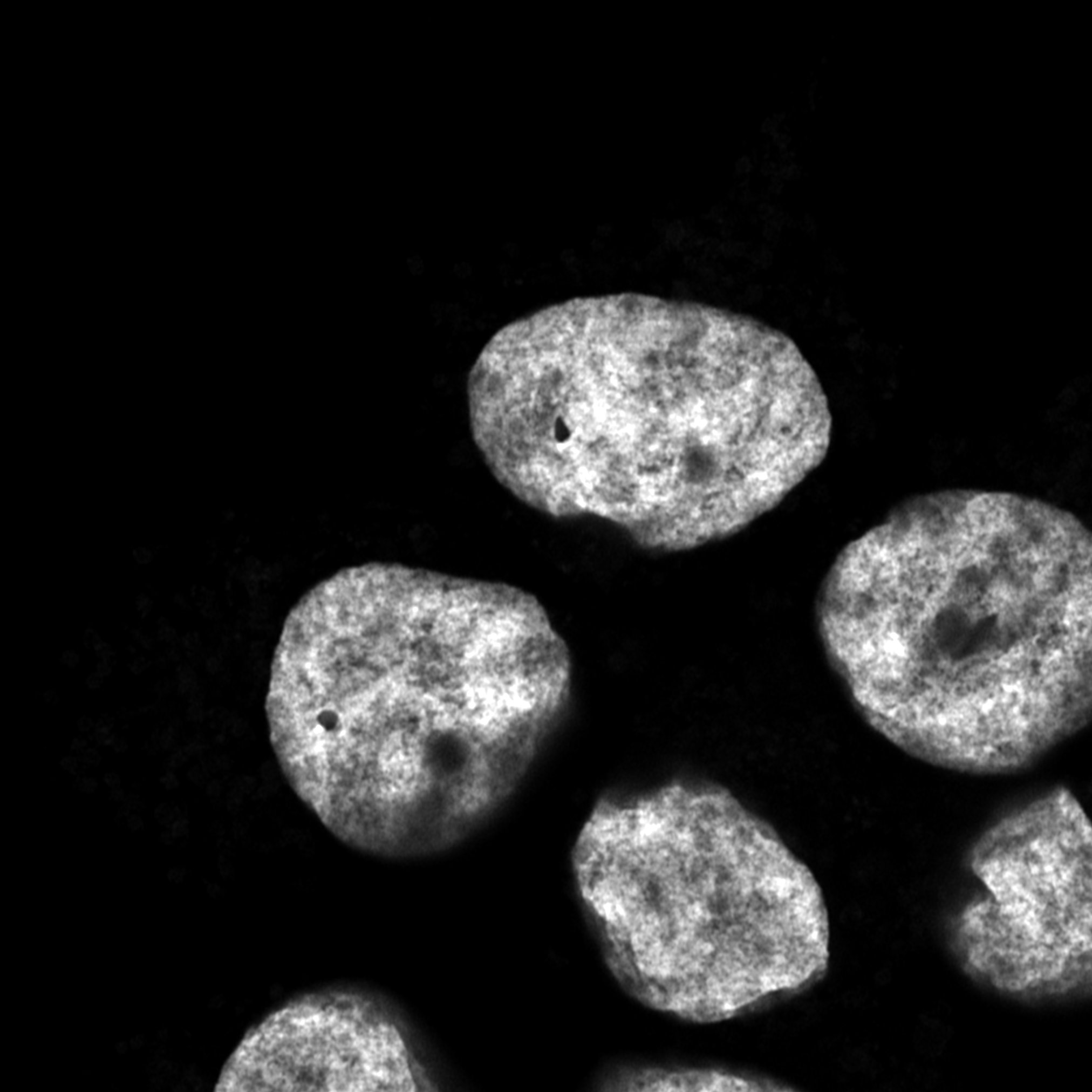

Supplement: Supplementary file 8 — Source data Fig. 5 [file 44318_2024_305_MOESM8_ESM.zip › Figure 5/5G/GOLPH_KO_PI_LAMP_PT_3_(Hoechst_C=2)_Airyscan Processing.tif]

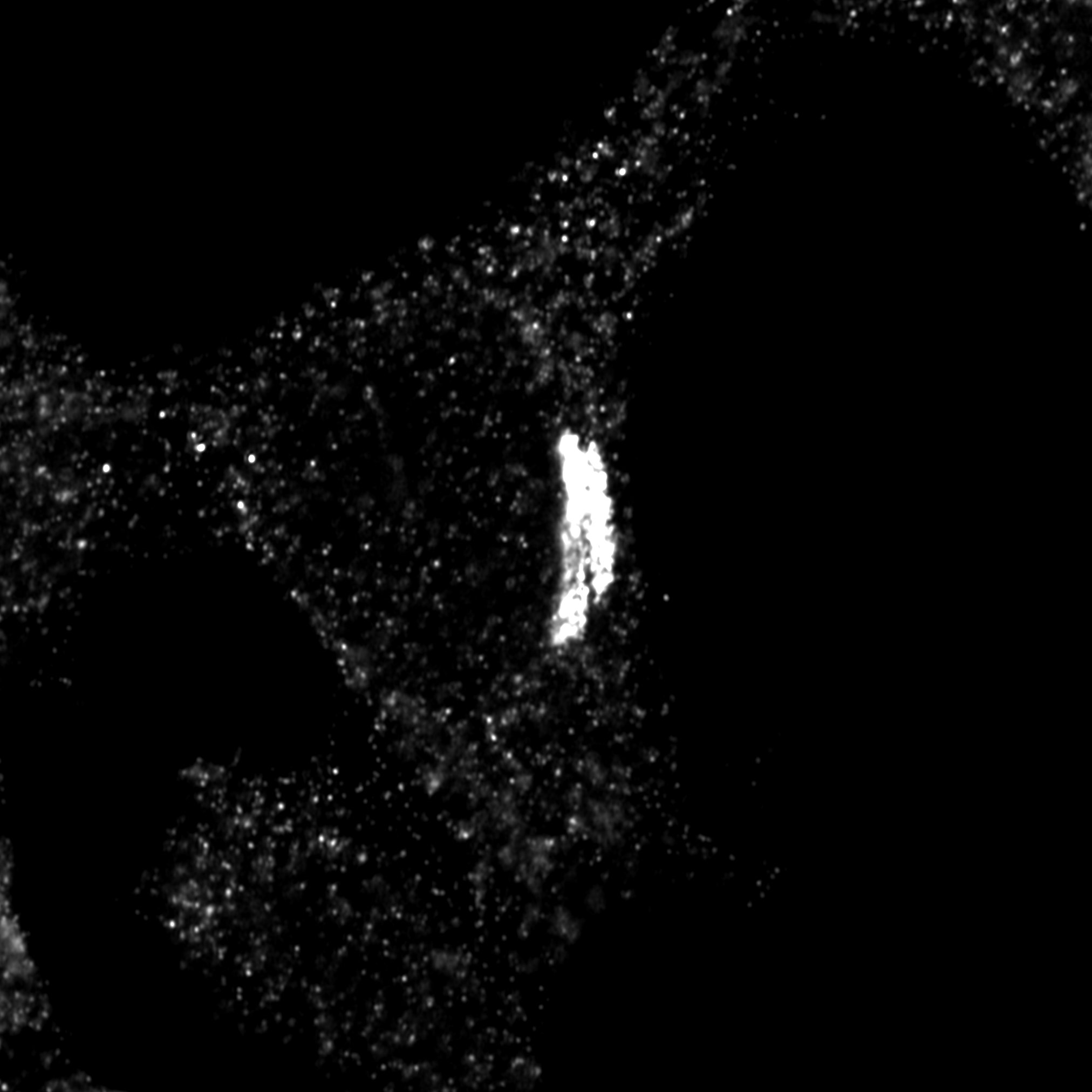

Supplement: Supplementary file 8 — Source data Fig. 5 [file 44318_2024_305_MOESM8_ESM.zip › Figure 5/5G/WT_ctrl_LYSET_LAMP_2_(LYSET488_C=1)_Airyscan Processing.tiff]

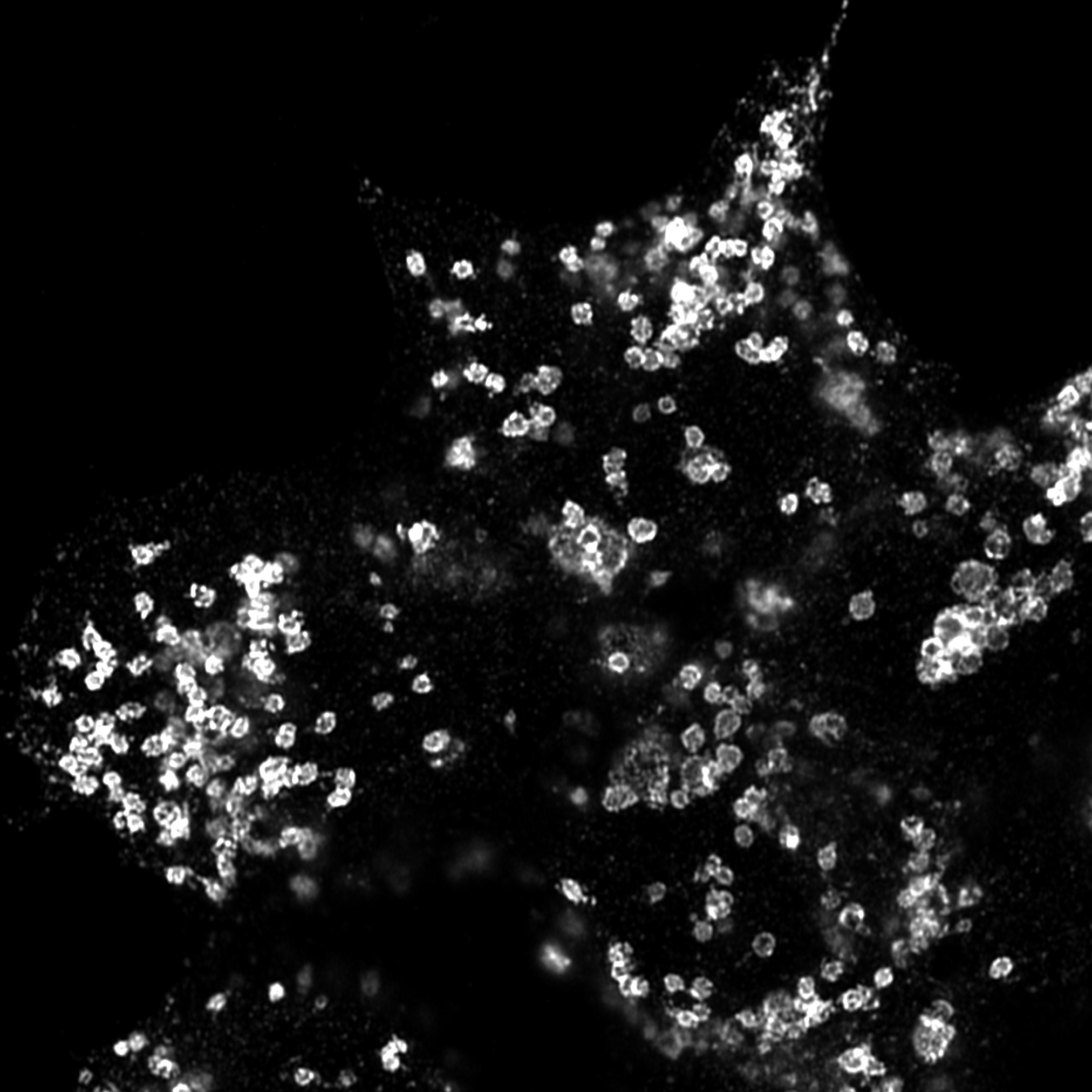

Supplement: Supplementary file 8 — Source data Fig. 5 [file 44318_2024_305_MOESM8_ESM.zip › Figure 5/5G/GOLPH_KO_PI_LAMP_PT_3_(LAMP2_488_C=1)_Airyscan Processing.tif]

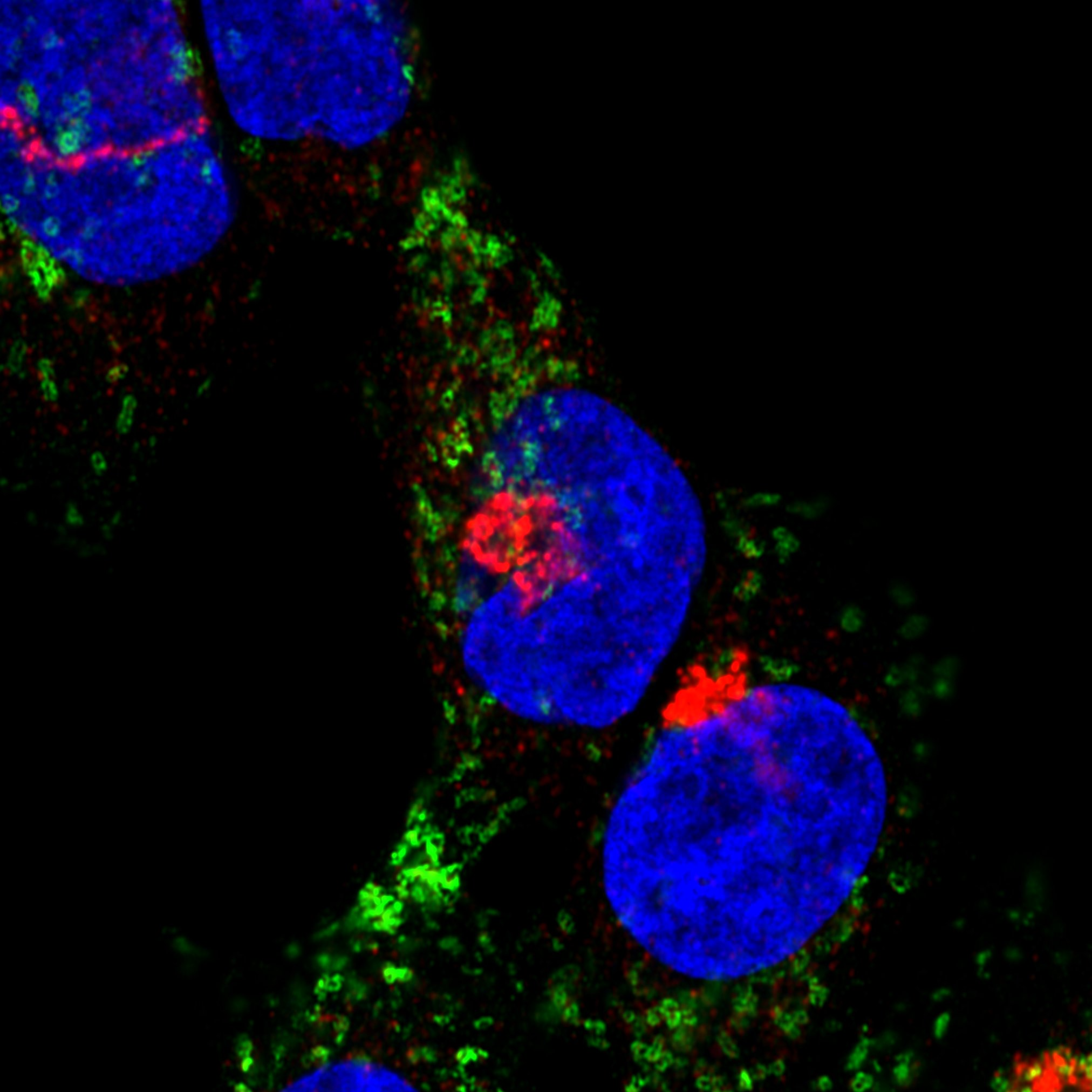

Supplement: Supplementary file 8 — Source data Fig. 5 [file 44318_2024_305_MOESM8_ESM.zip › Figure 5/5G/WT_ctrl_LAMP_PT_2_(merge)_Airyscan Processing.tif]

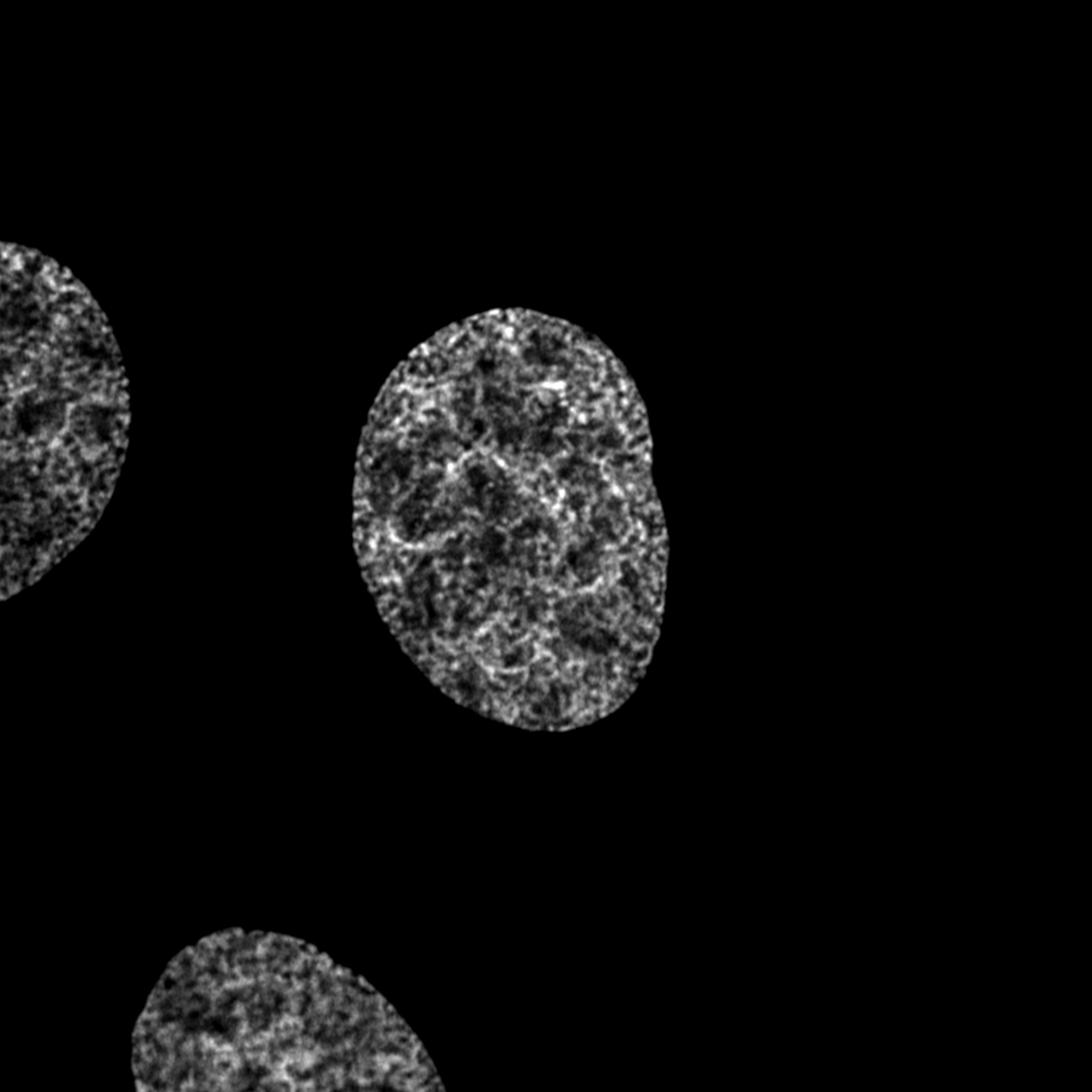

Supplement: Supplementary file 8 — Source data Fig. 5 [file 44318_2024_305_MOESM8_ESM.zip › Figure 5/5G/WT_PI_LYSET_LAMP_3_(Hoechst_C=2)_Airyscan Processing.tiff]

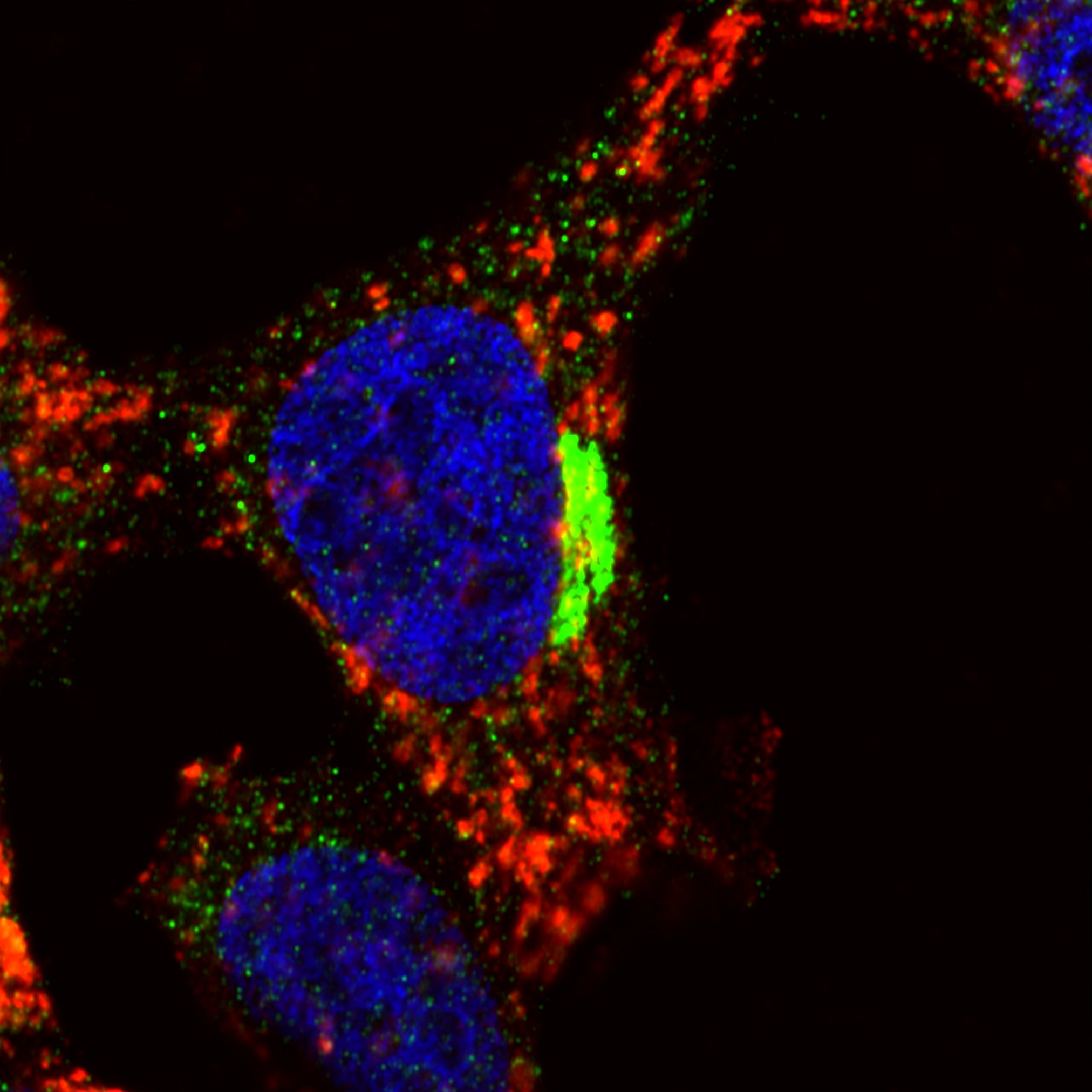

Supplement: Supplementary file 8 — Source data Fig. 5 [file 44318_2024_305_MOESM8_ESM.zip › Figure 5/5G/WT_ctrl_LYSET_LAMP_2_(merge)_Airyscan Processing.tiff]

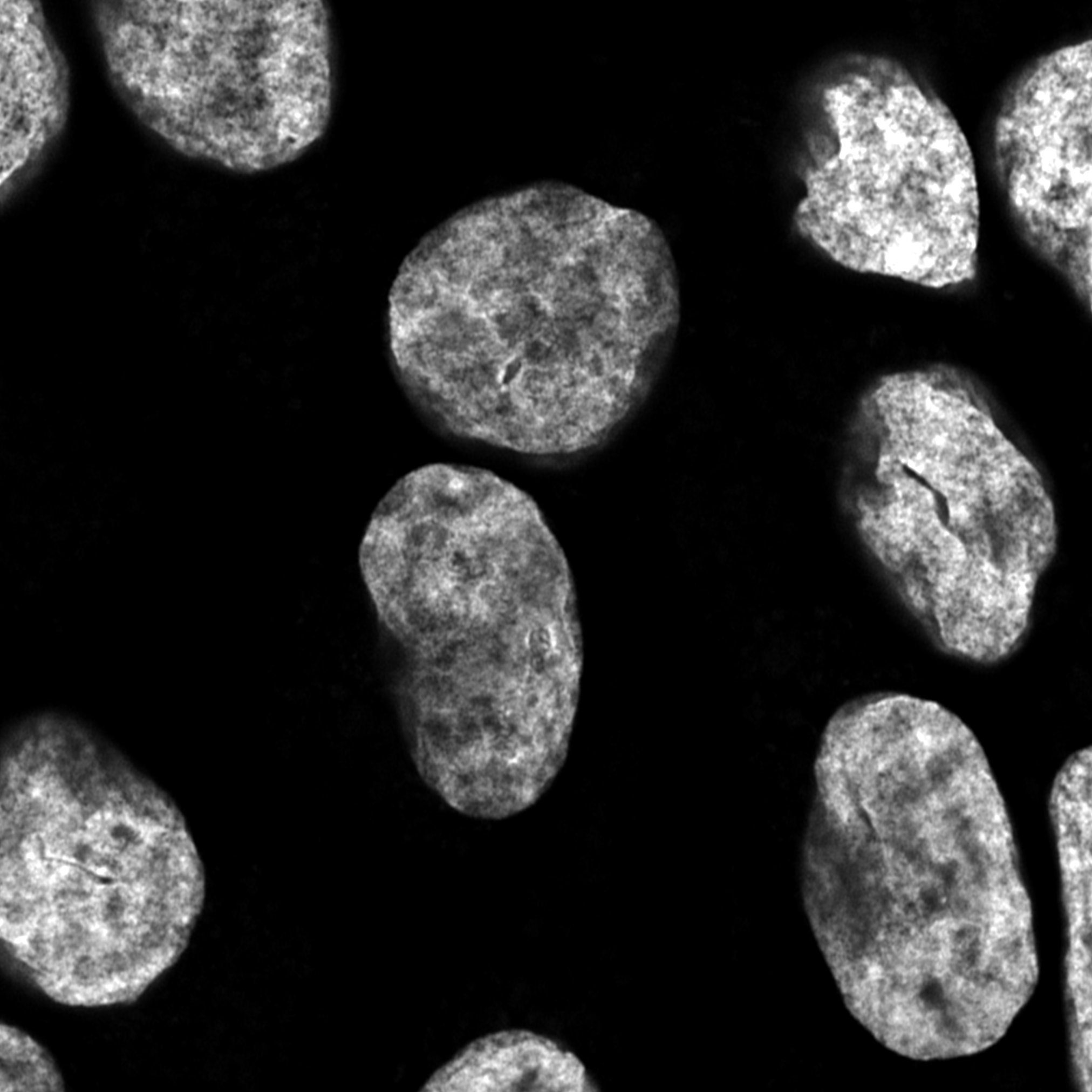

Supplement: Supplementary file 8 — Source data Fig. 5 [file 44318_2024_305_MOESM8_ESM.zip › Figure 5/5G/WT_PI_LAMP_PT_1_(Hoechst_C=2)_Airyscan Processing.tif]

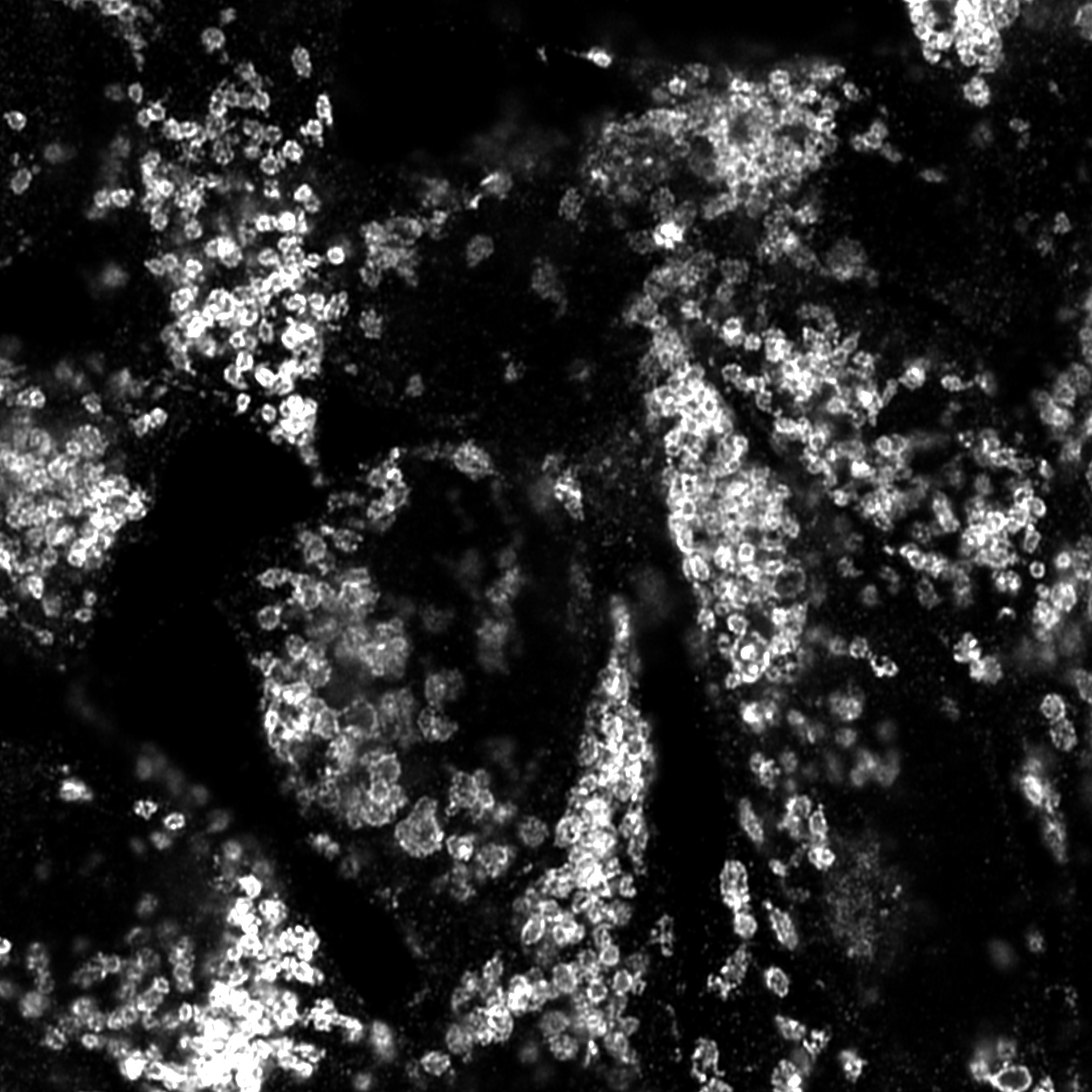

Supplement: Supplementary file 8 — Source data Fig. 5 [file 44318_2024_305_MOESM8_ESM.zip › Figure 5/5G/WT_PI_LAMP_PT_1_(LAMP2488_C=1)_Airyscan Processing.tif]

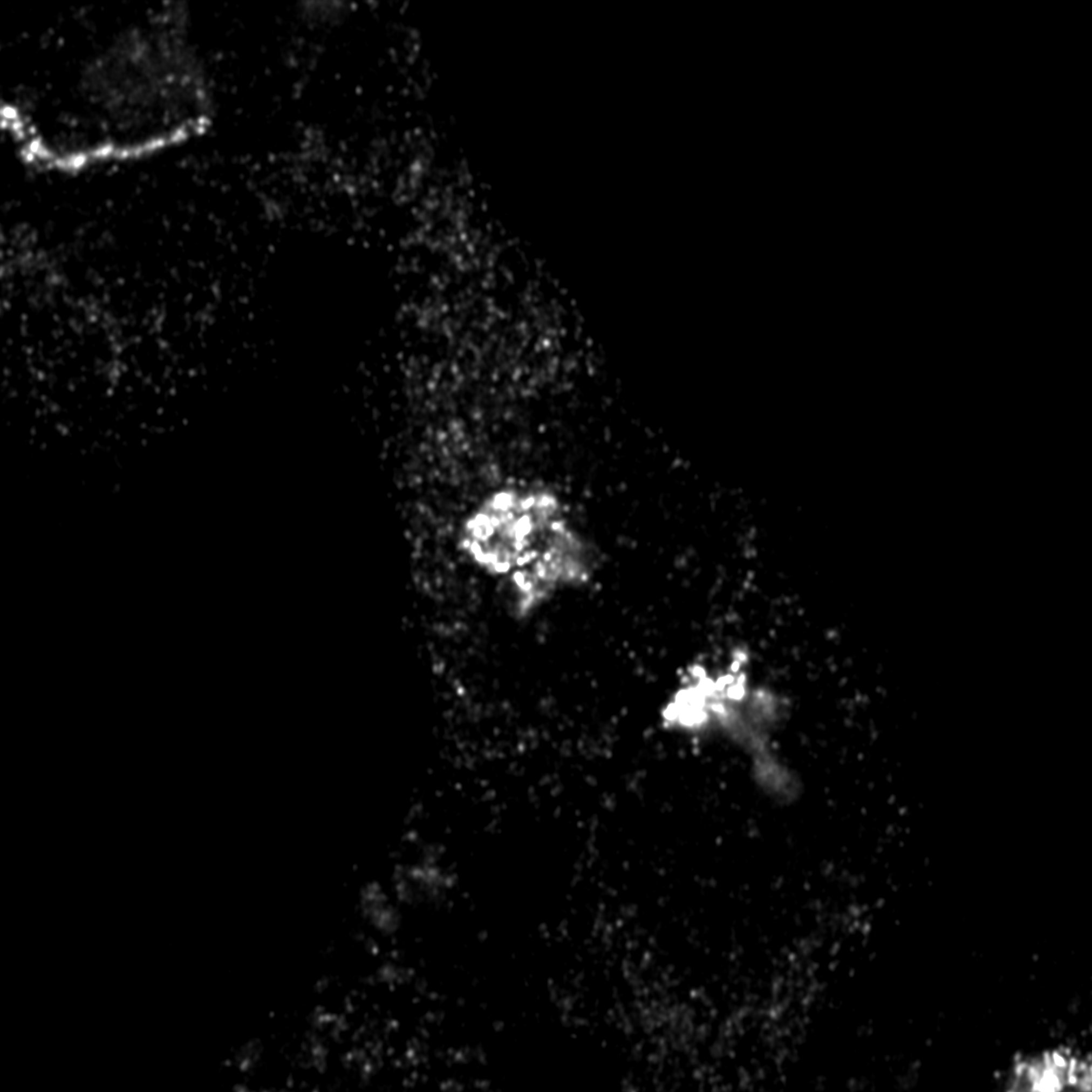

Supplement: Supplementary file 8 — Source data Fig. 5 [file 44318_2024_305_MOESM8_ESM.zip › Figure 5/5G/WT_ctrl_LAMP_PT_2_(PT594_C=0)_Airyscan Processing.tif]

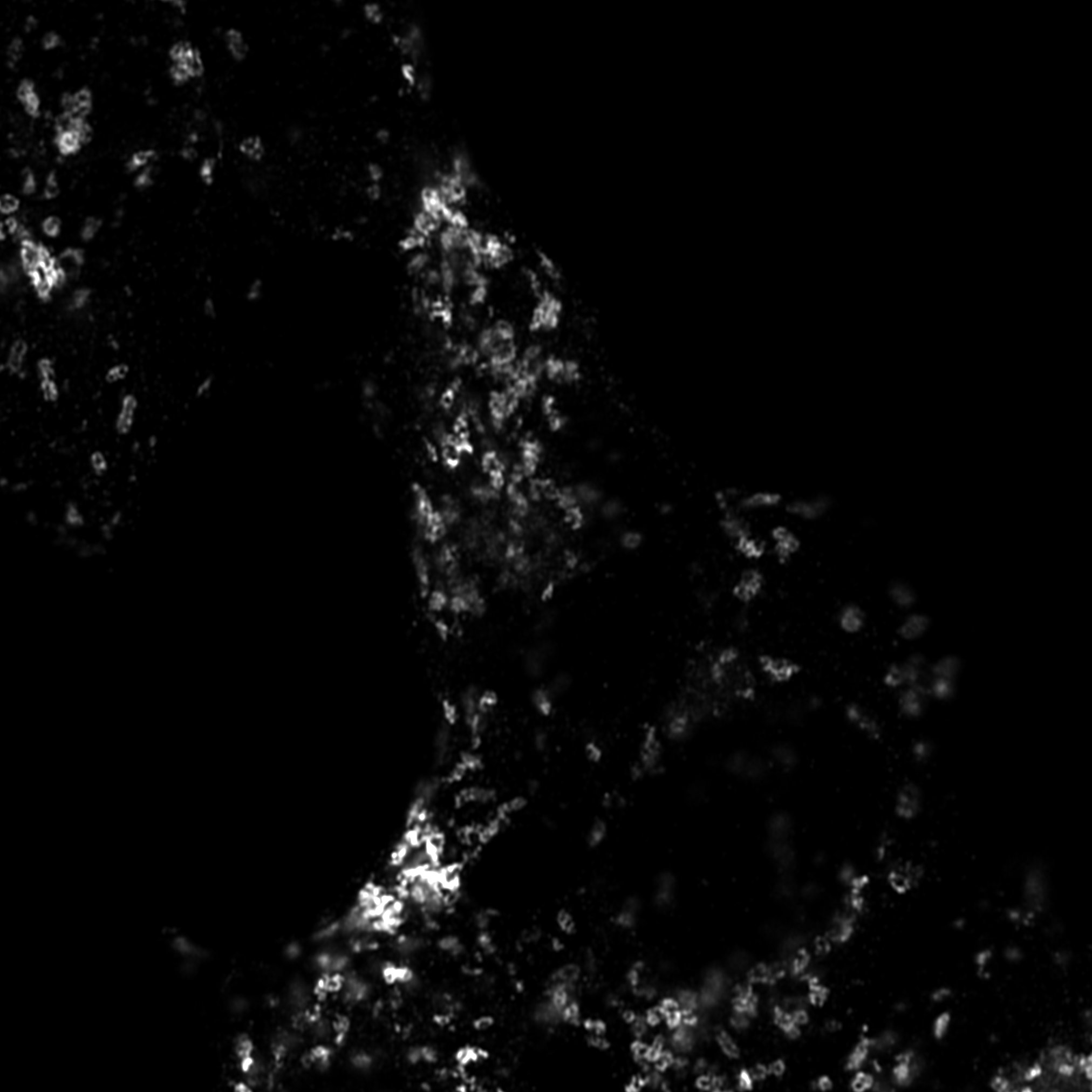

Supplement: Supplementary file 8 — Source data Fig. 5 [file 44318_2024_305_MOESM8_ESM.zip › Figure 5/5G/WT_ctrl_LAMP_PT_2_(LAMP2488_C=1)_Airyscan Processing.tif]

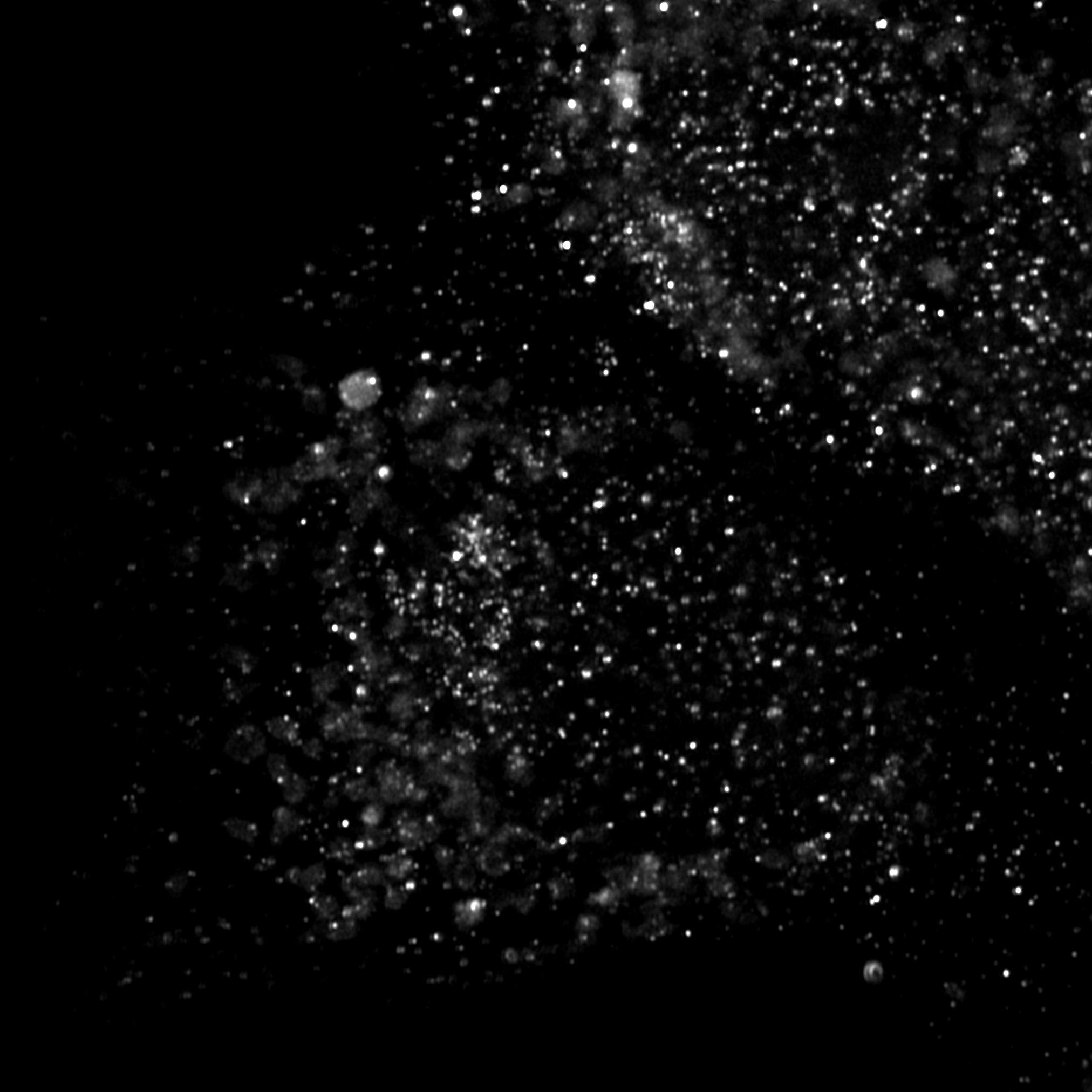

Supplement: Supplementary file 8 — Source data Fig. 5 [file 44318_2024_305_MOESM8_ESM.zip › Figure 5/5G/GOLPH KO_PI_LYSET_LAMP_6_(LYSET488_C=1)Airyscan Processing.tiff]

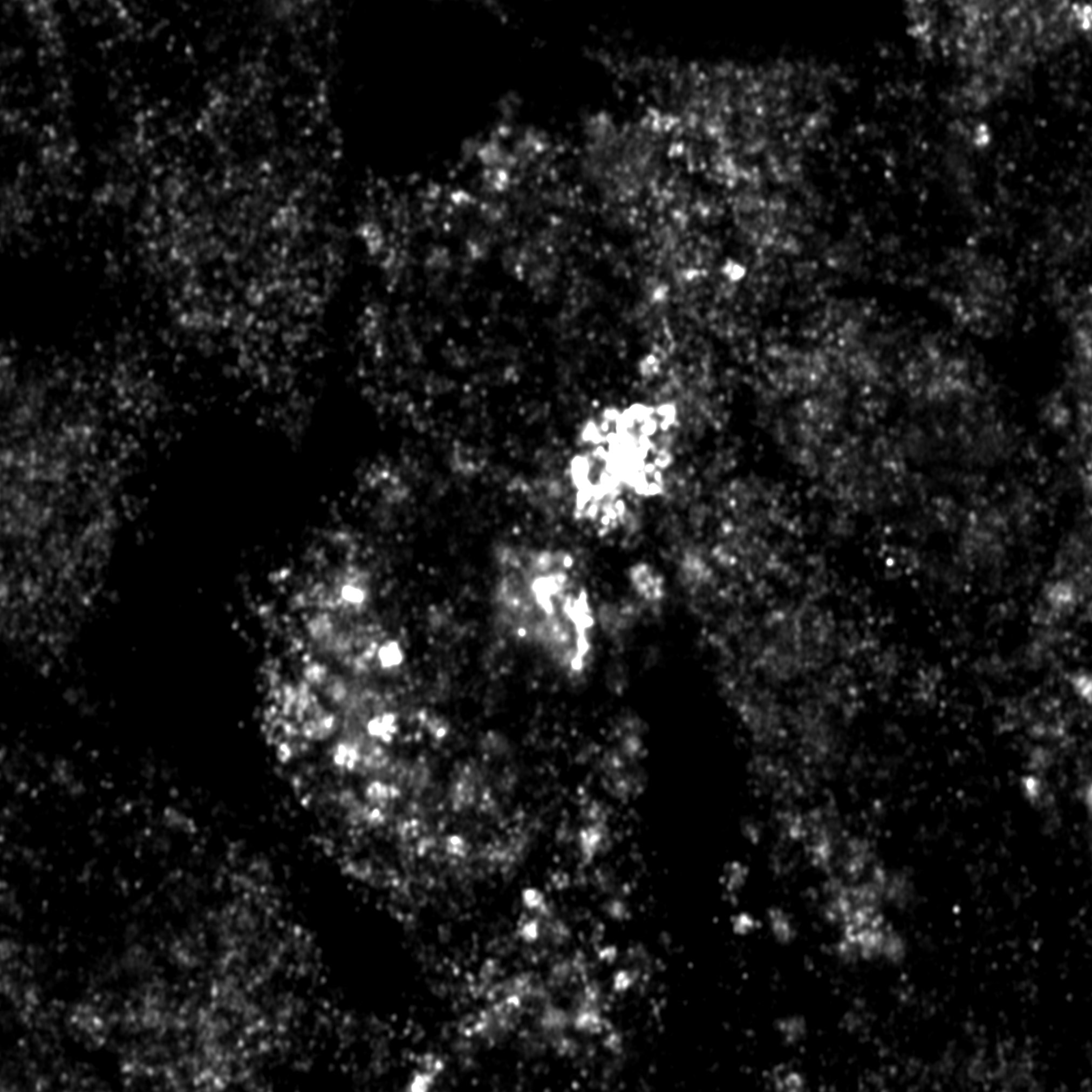

Supplement: Supplementary file 8 — Source data Fig. 5 [file 44318_2024_305_MOESM8_ESM.zip › Figure 5/5G/WT_PI_LAMP_PT_1_(PT594_C=0)_Airyscan Processing.tif]

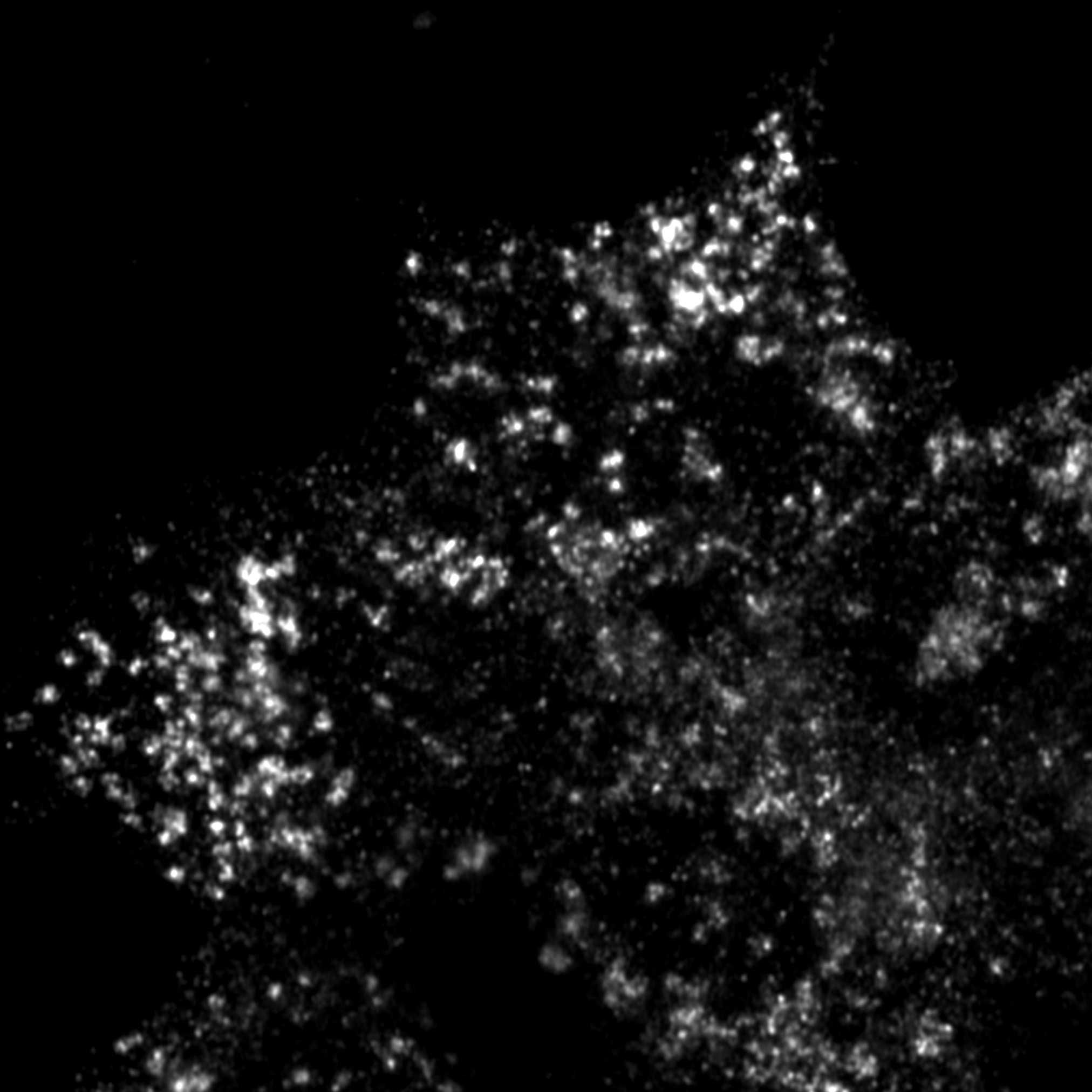

Supplement: Supplementary file 8 — Source data Fig. 5 [file 44318_2024_305_MOESM8_ESM.zip › Figure 5/5G/GOLPH_KO_PI_LAMP_PT_3_(PT594_C=0)_Airyscan Processing.tif]

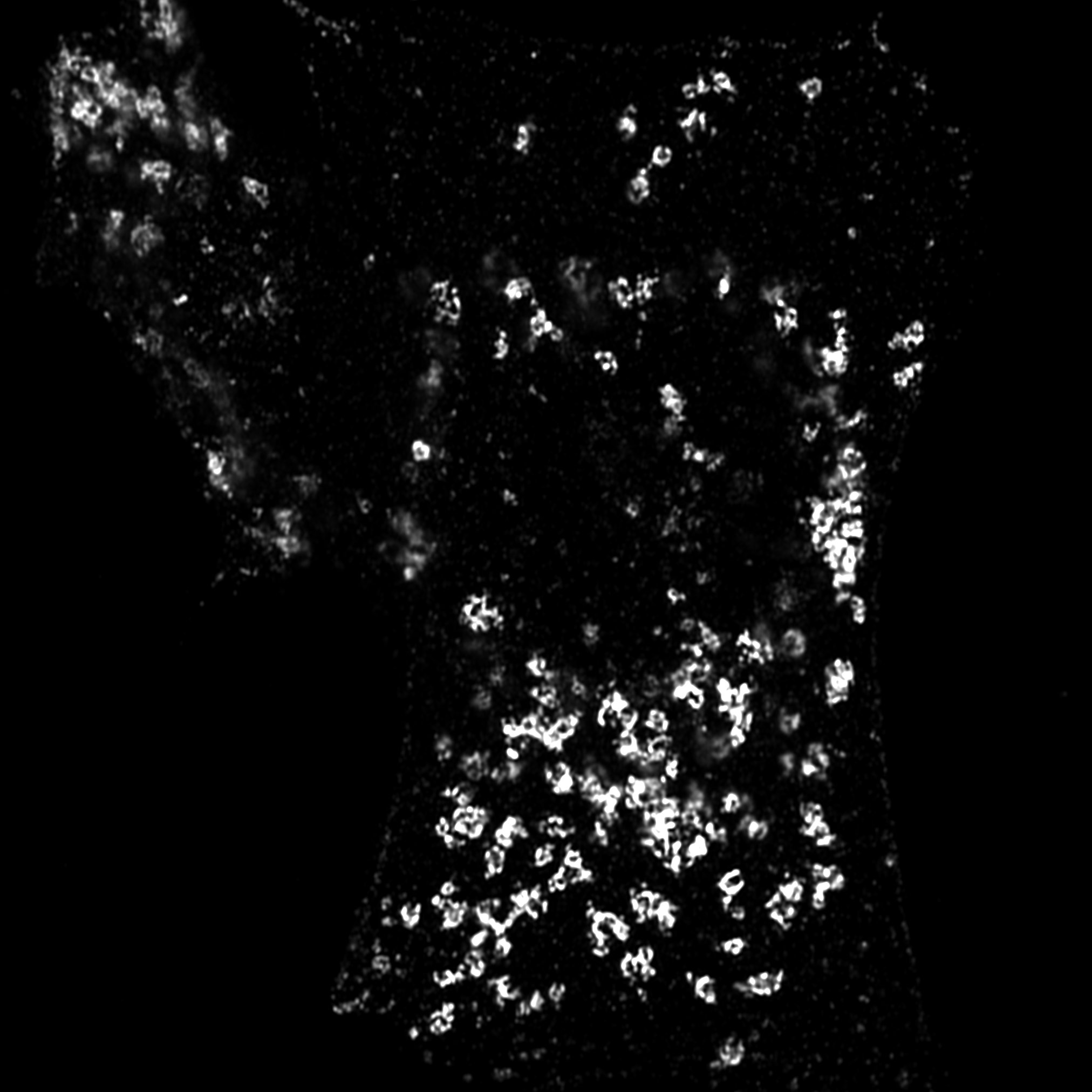

Supplement: Supplementary file 8 — Source data Fig. 5 [file 44318_2024_305_MOESM8_ESM.zip › Figure 5/5G/GOLPH_KO_ctrl_LAMP_PT_2_(LAMP2_488_C=1)_Airyscan Processing.tif]

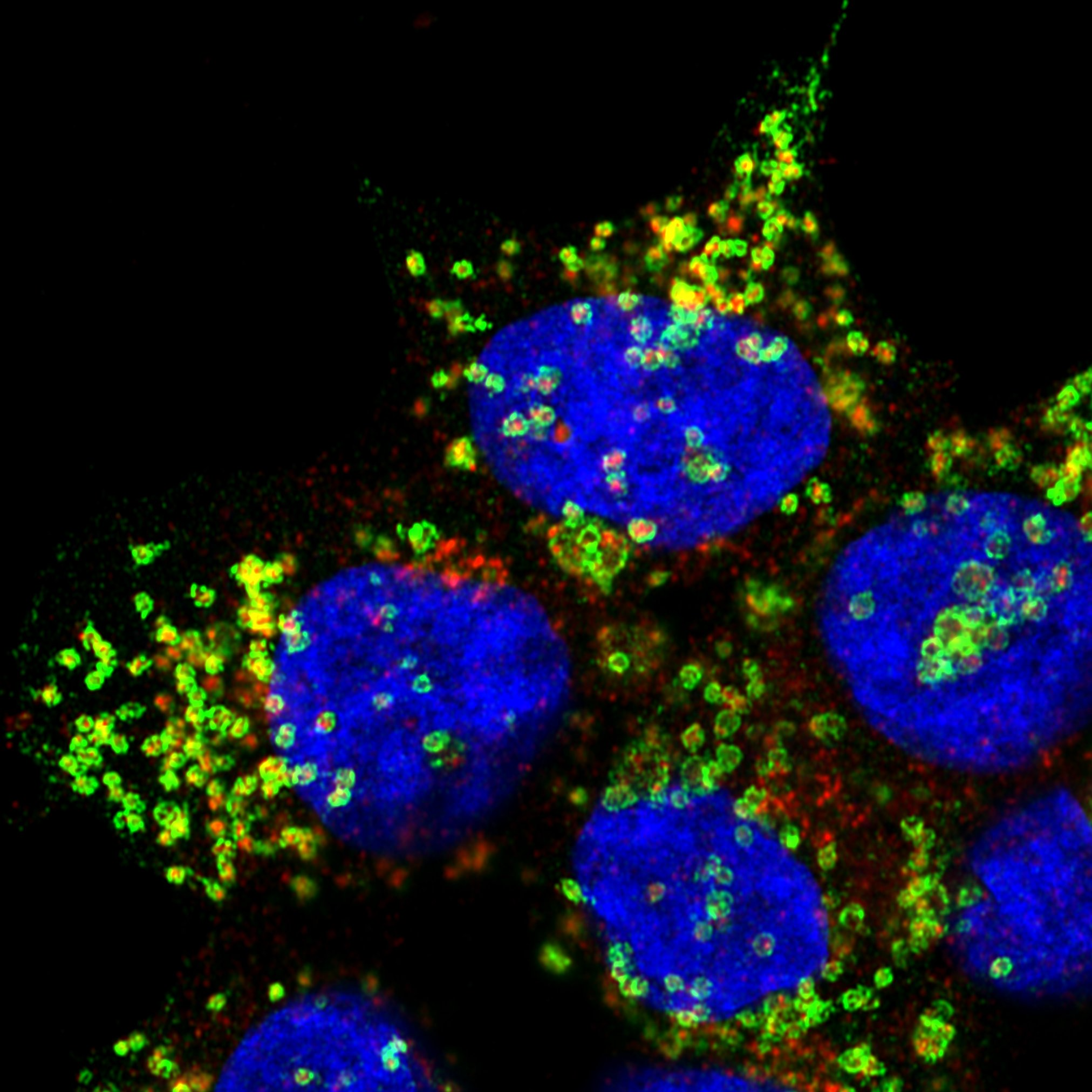

Supplement: Supplementary file 8 — Source data Fig. 5 [file 44318_2024_305_MOESM8_ESM.zip › Figure 5/5G/GOLPH_KO_PI_LAMP_PT_3_(merge)_Airyscan Processing.tif]

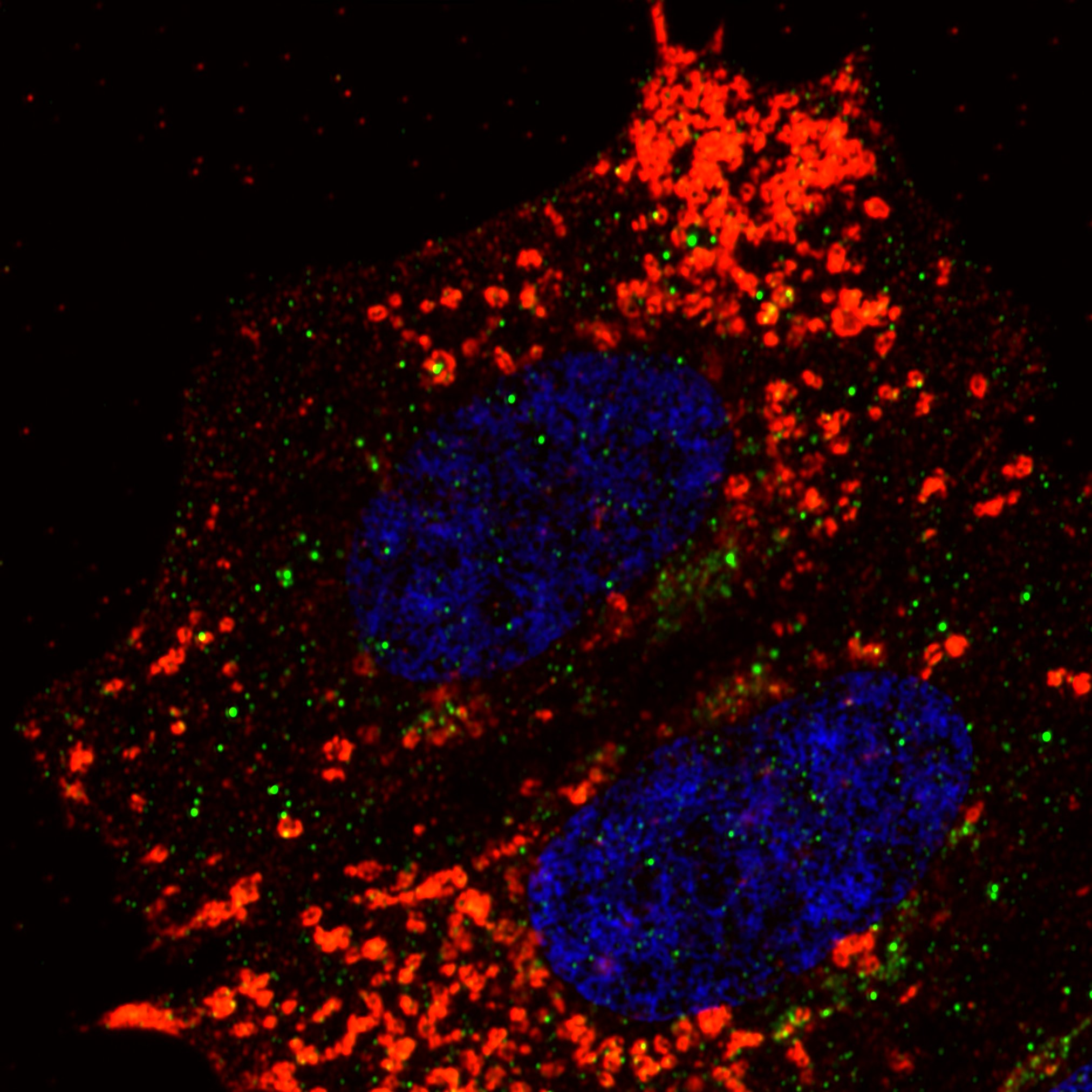

Supplement: Supplementary file 8 — Source data Fig. 5 [file 44318_2024_305_MOESM8_ESM.zip › Figure 5/5G/GOLPH KO_ctrl_LYSET_LAMP_2_(merge)_Airyscan Processing.tiff]

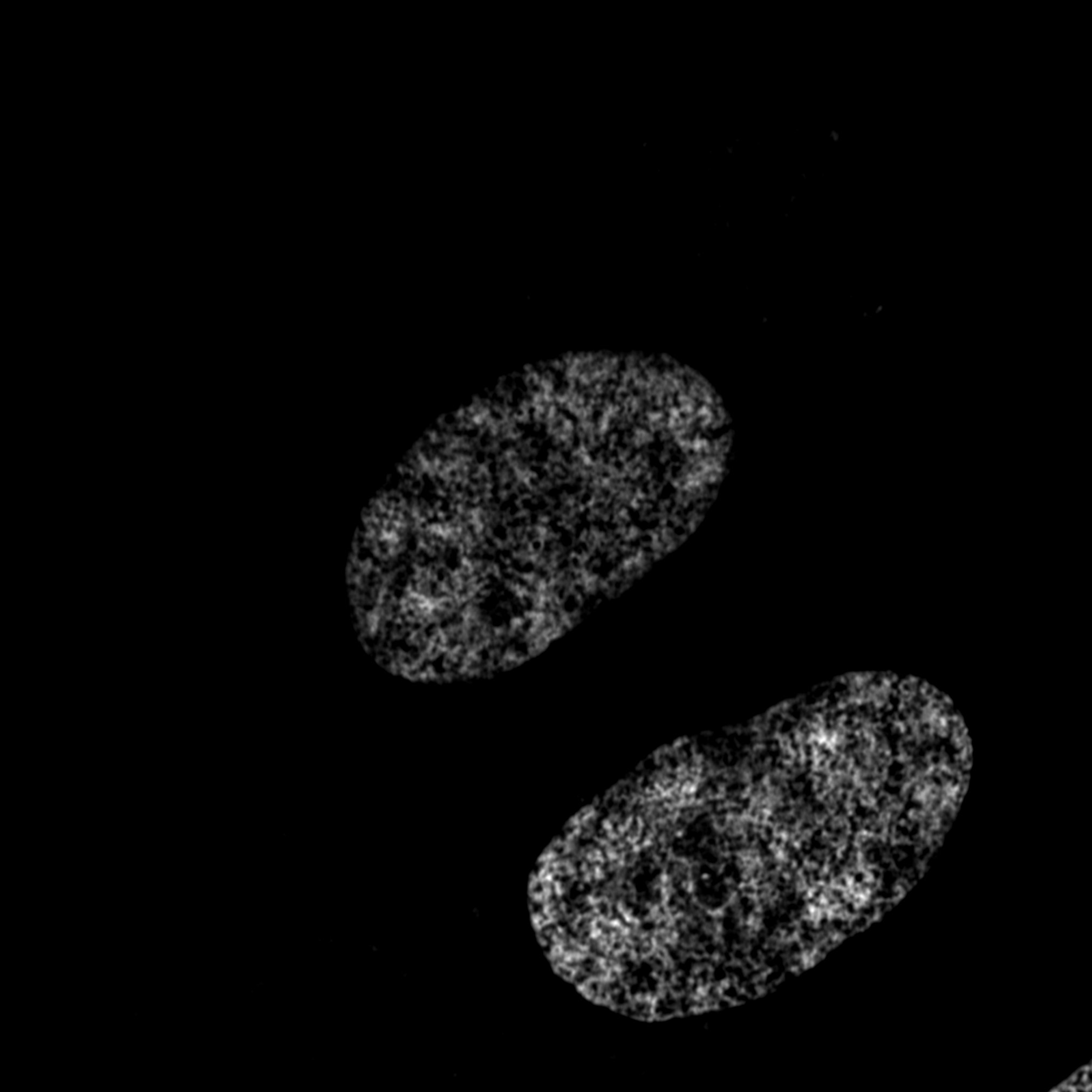

Supplement: Supplementary file 8 — Source data Fig. 5 [file 44318_2024_305_MOESM8_ESM.zip › Figure 5/5G/GOLPH KO_ctrl_LYSET_LAMP_2_(Hoechst_C=2)=Airyscan Processing.tiff]

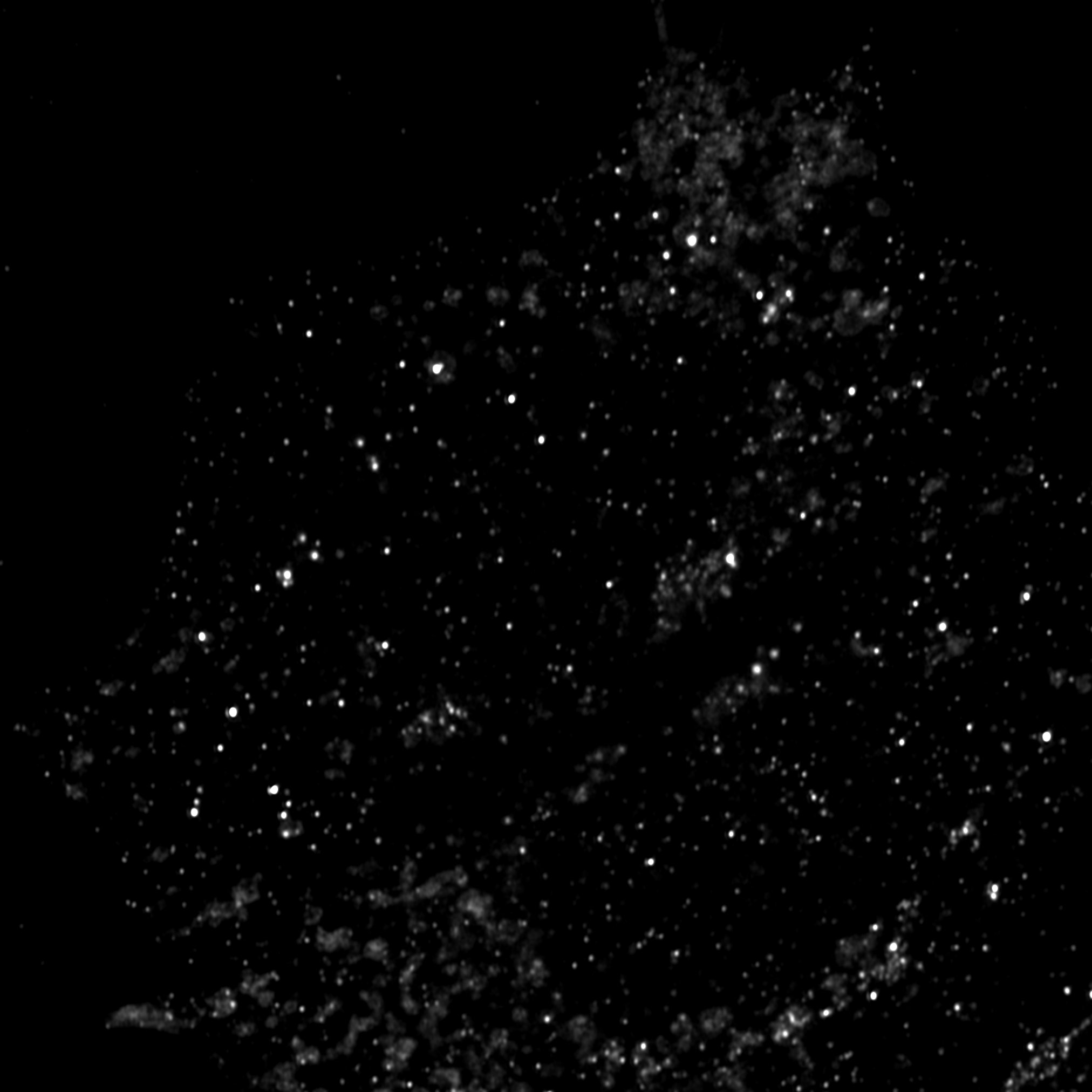

Supplement: Supplementary file 8 — Source data Fig. 5 [file 44318_2024_305_MOESM8_ESM.zip › Figure 5/5G/GOLPH KO_ctrl_LYSET_LAMP_2_(LYSET488_C=1)_Airyscan Processing.tiff]

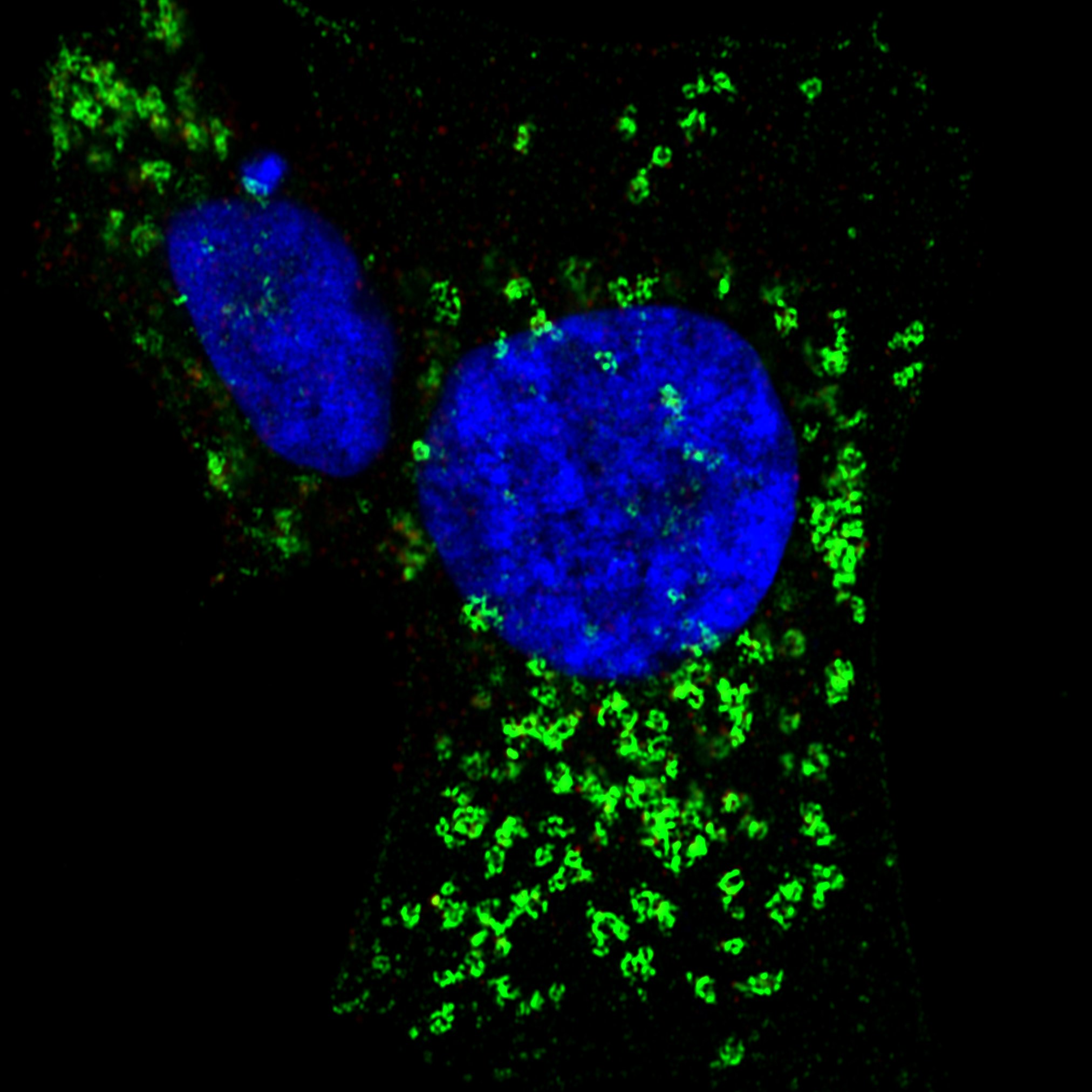

Supplement: Supplementary file 8 — Source data Fig. 5 [file 44318_2024_305_MOESM8_ESM.zip › Figure 5/5G/GOLPH_KO_ctrl_LAMP_PT_2_(merge)_Airyscan Processing.tif]

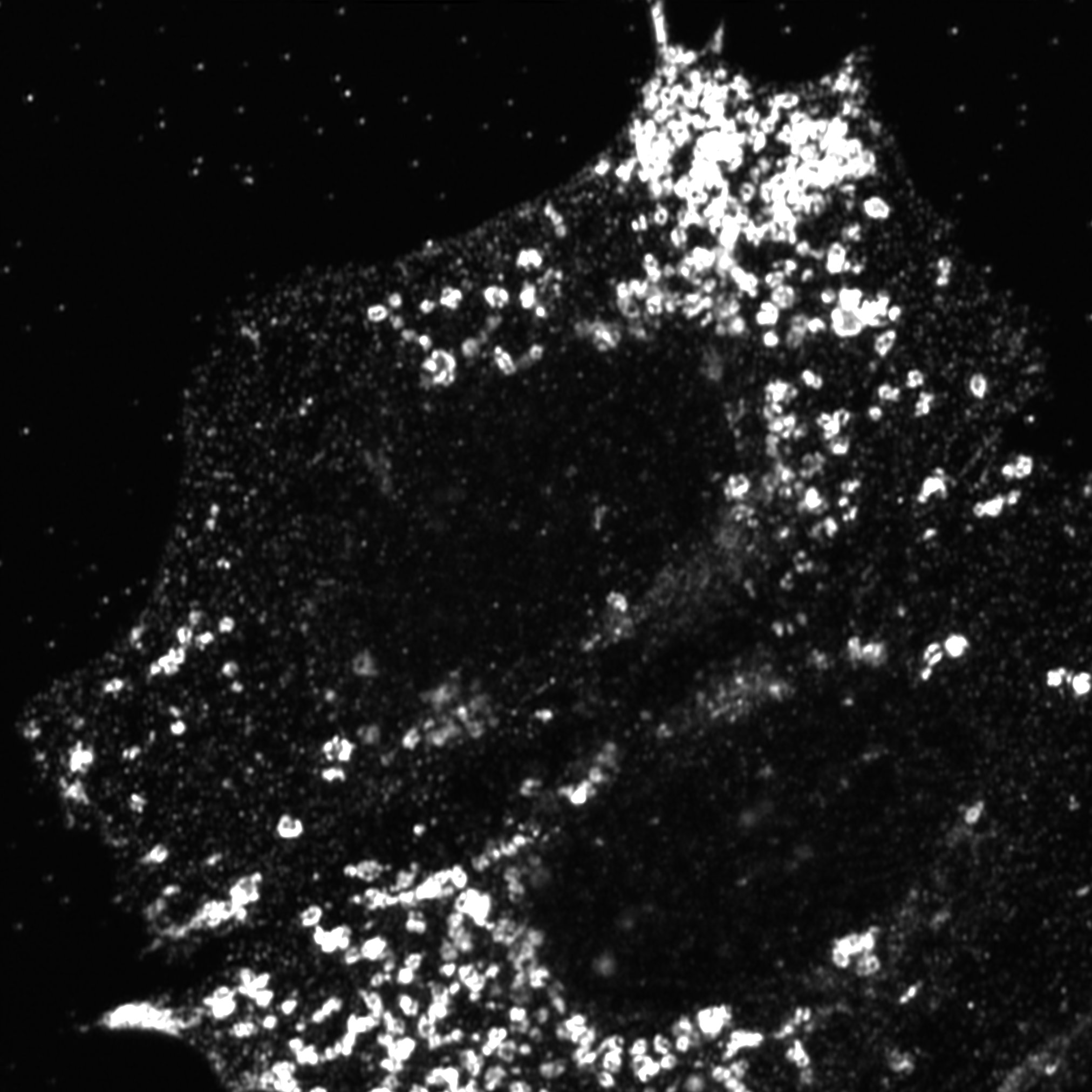

Supplement: Supplementary file 8 — Source data Fig. 5 [file 44318_2024_305_MOESM8_ESM.zip › Figure 5/5G/GOLPH KO_ctrl_LYSET_LAMP_2_(LAMP594_C=0)_Airyscan Processing.tiff]

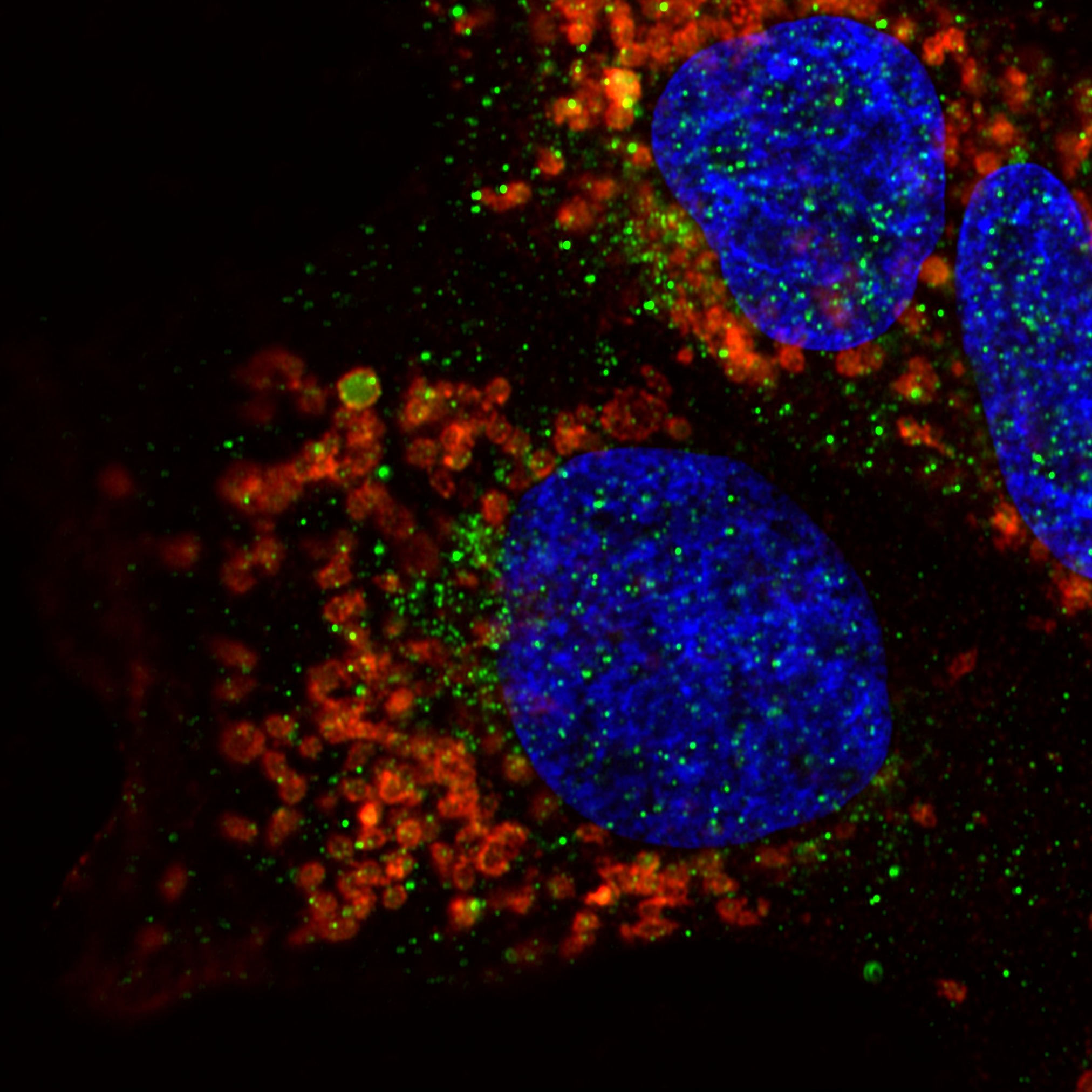

Supplement: Supplementary file 8 — Source data Fig. 5 [file 44318_2024_305_MOESM8_ESM.zip › Figure 5/5G/GOLPH KO_PI_LYSET_LAMP_6_(merge)Airyscan Processing.tiff]

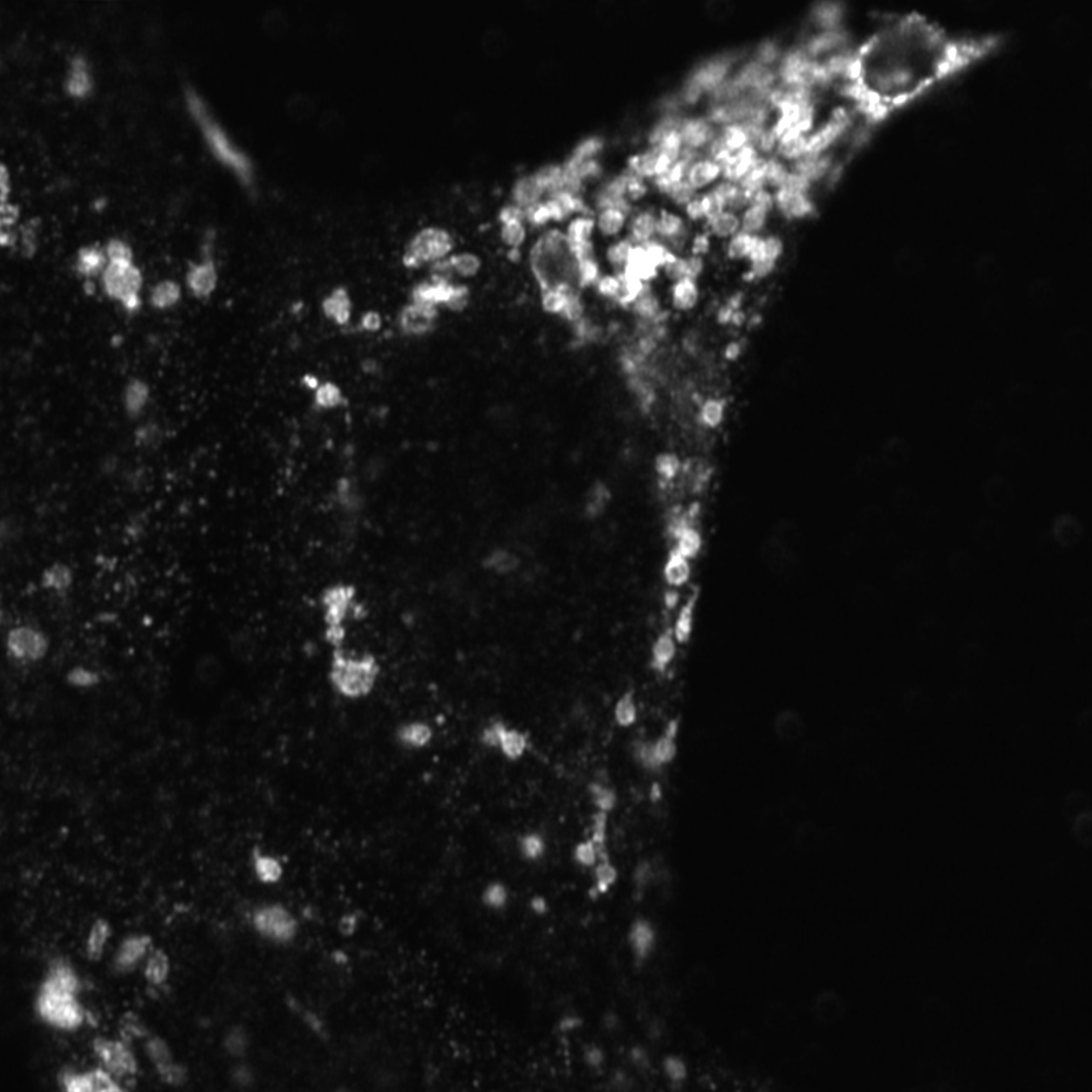

Supplement: Supplementary file 8 — Source data Fig. 5 [file 44318_2024_305_MOESM8_ESM.zip › Figure 5/5G/WT_PI_LYSET_LAMP_3_(LAMP594_C=0)Airyscan Processing.tiff]

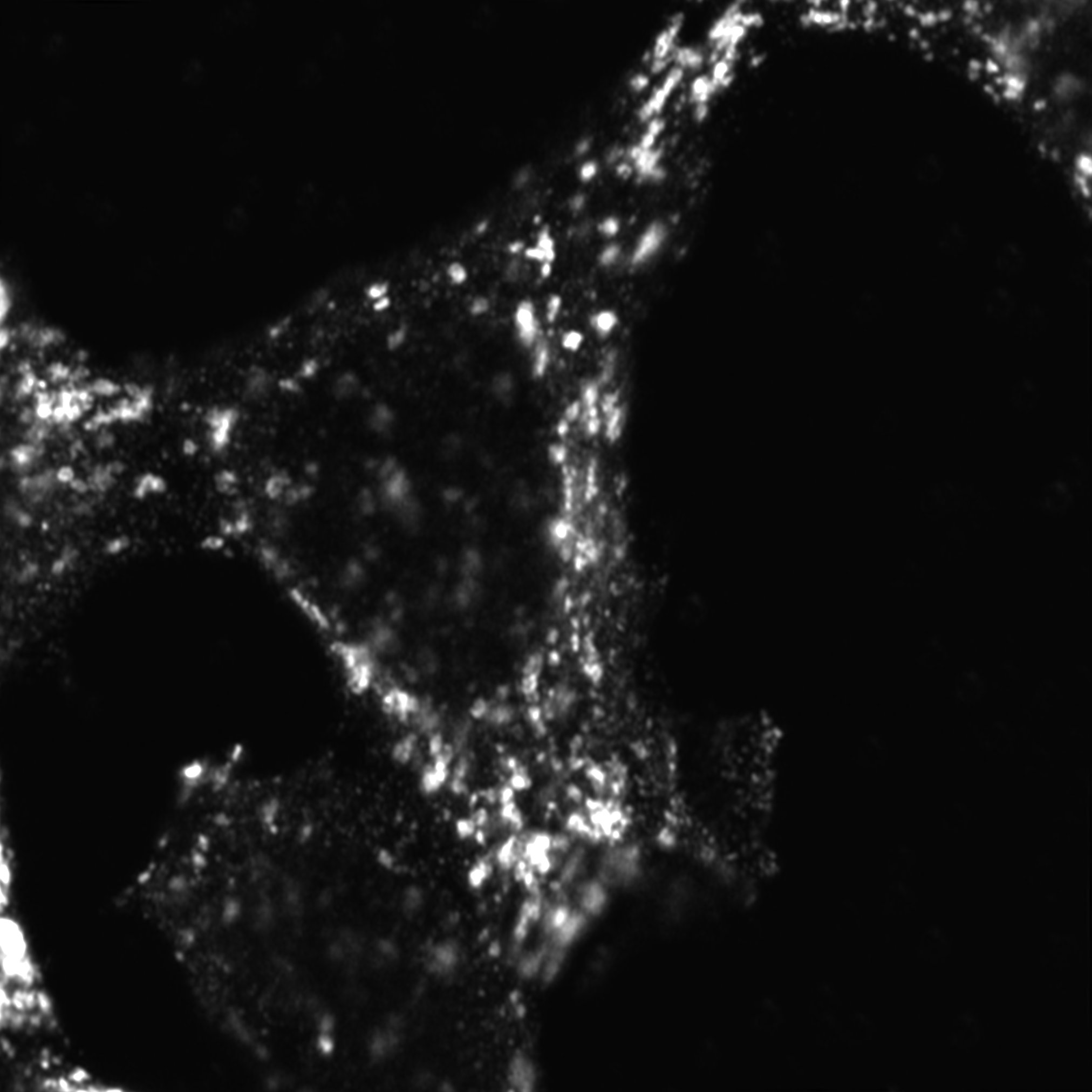

Supplement: Supplementary file 8 — Source data Fig. 5 [file 44318_2024_305_MOESM8_ESM.zip › Figure 5/5G/WT_ctrl_LYSET_LAMP_2_(LAMP594_C=0)_Airyscan Processing.tiff]

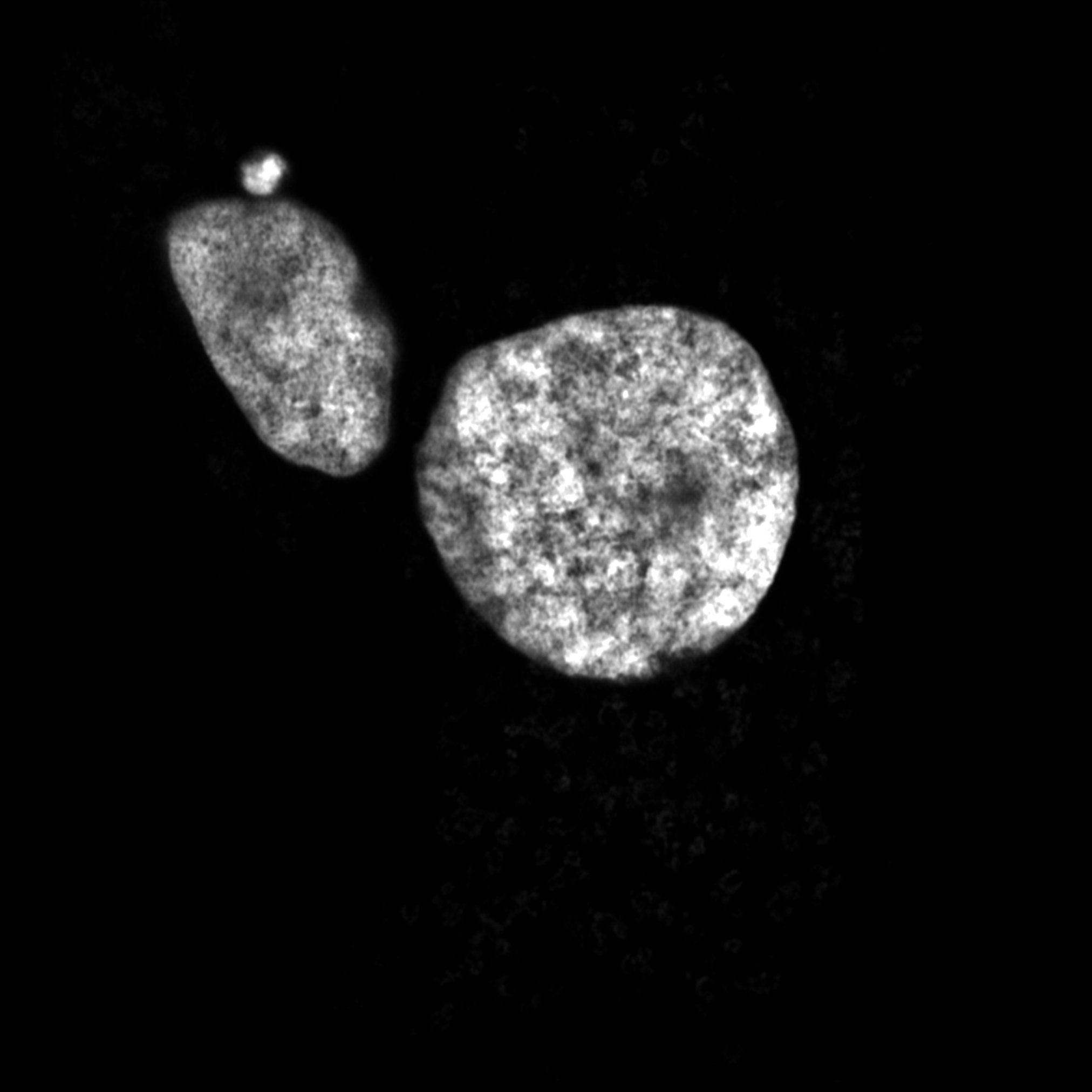

Supplement: Supplementary file 8 — Source data Fig. 5 [file 44318_2024_305_MOESM8_ESM.zip › Figure 5/5G/GOLPH_KO_ctrl_LAMP_PT_2_(Hoechst_C=2)_Airyscan Processing.tif]

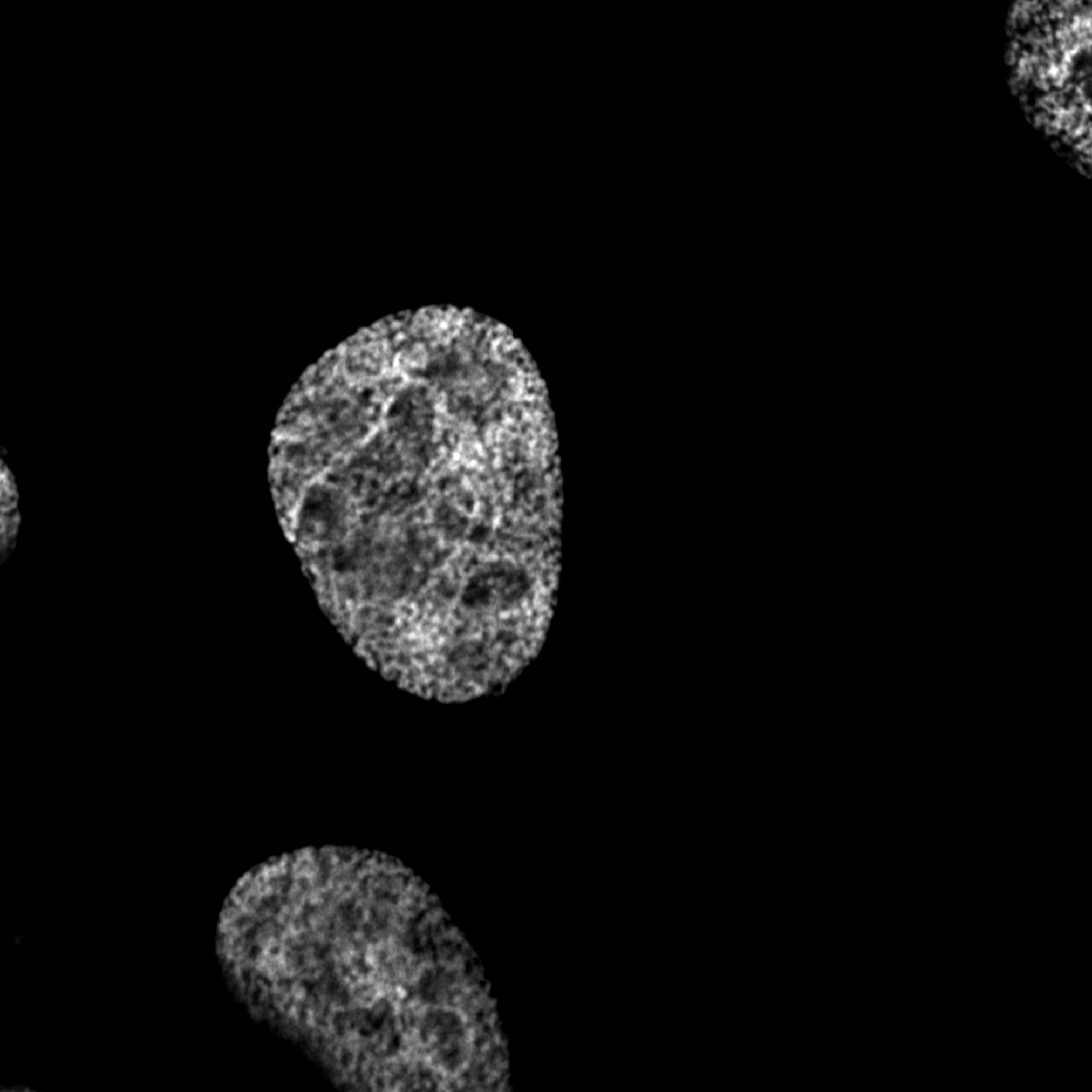

Supplement: Supplementary file 8 — Source data Fig. 5 [file 44318_2024_305_MOESM8_ESM.zip › Figure 5/5G/WT_ctrl_LYSET_LAMP_2_(Hoechst_C=2)Airyscan Processing.tiff]

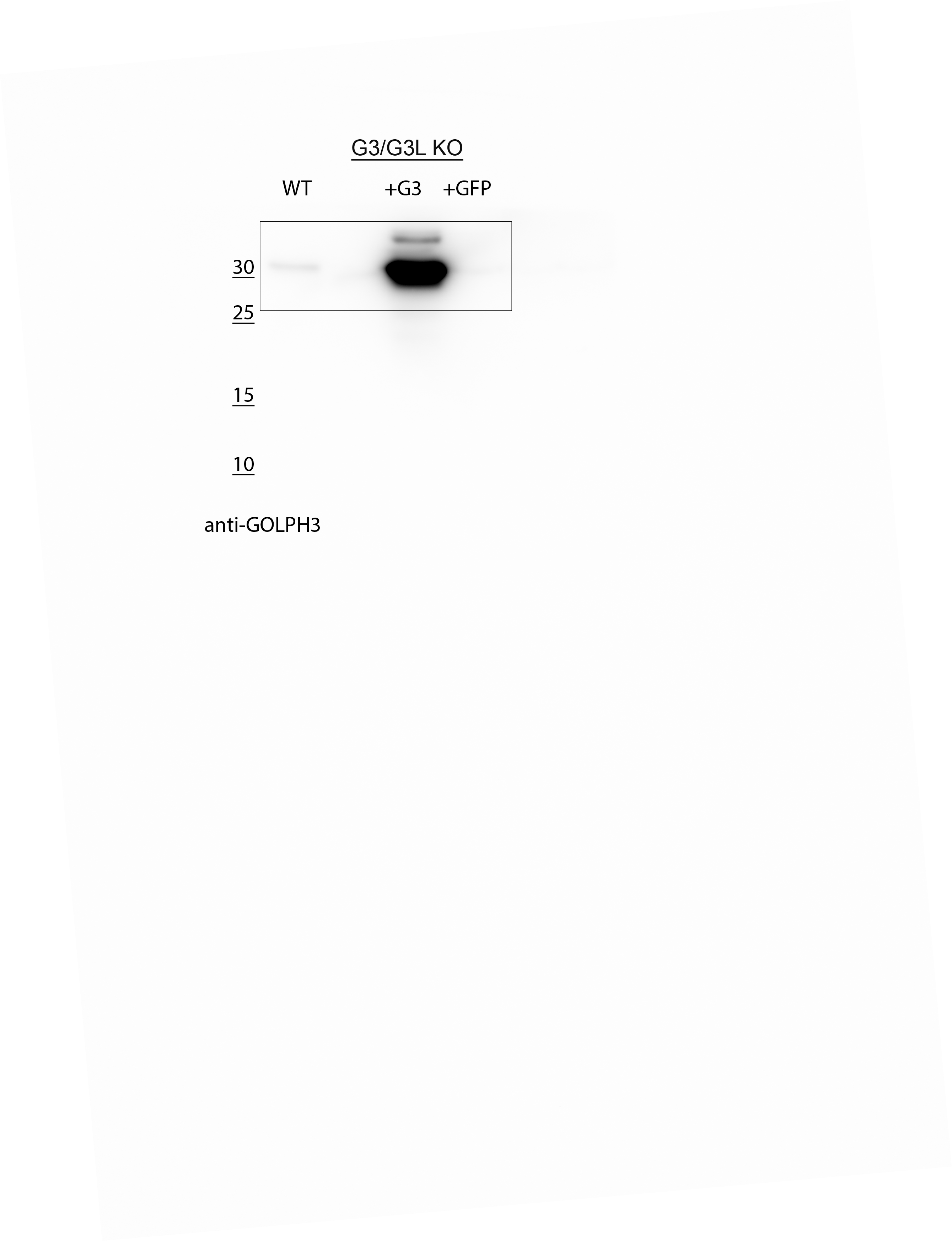

Supplement: Supplementary file 8 — Source data Fig. 5 [file 44318_2024_305_MOESM8_ESM.zip › Figure 5/5A/source data GOLPH3 time series 20231004_151010-09_Ch_Chemi.tif]

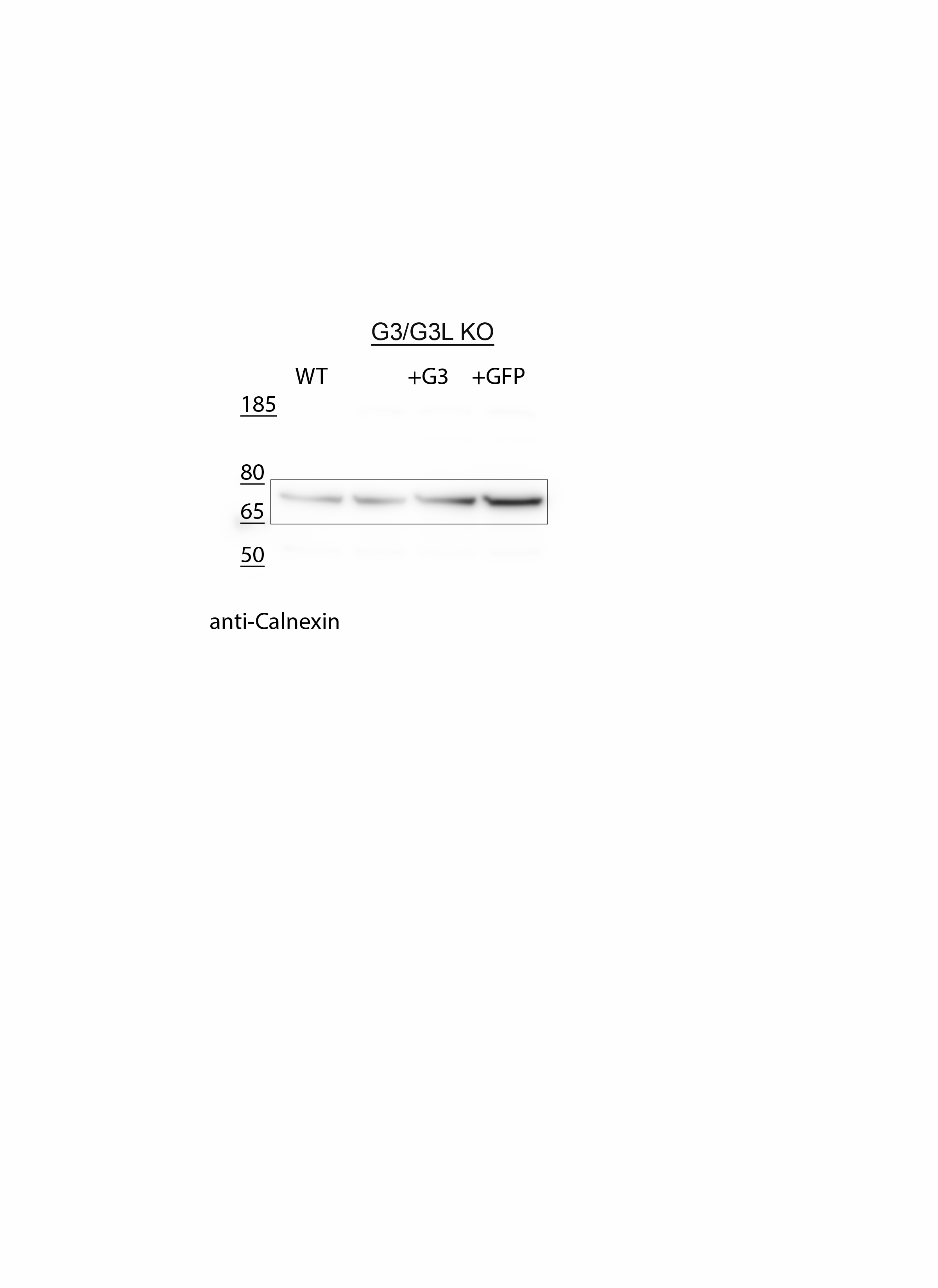

Supplement: Supplementary file 8 — Source data Fig. 5 [file 44318_2024_305_MOESM8_ESM.zip › Figure 5/5A/source data Calnexin for LYSET, GOLPH3 time series 20230928_160531-02_Ch_Chemi.tif]

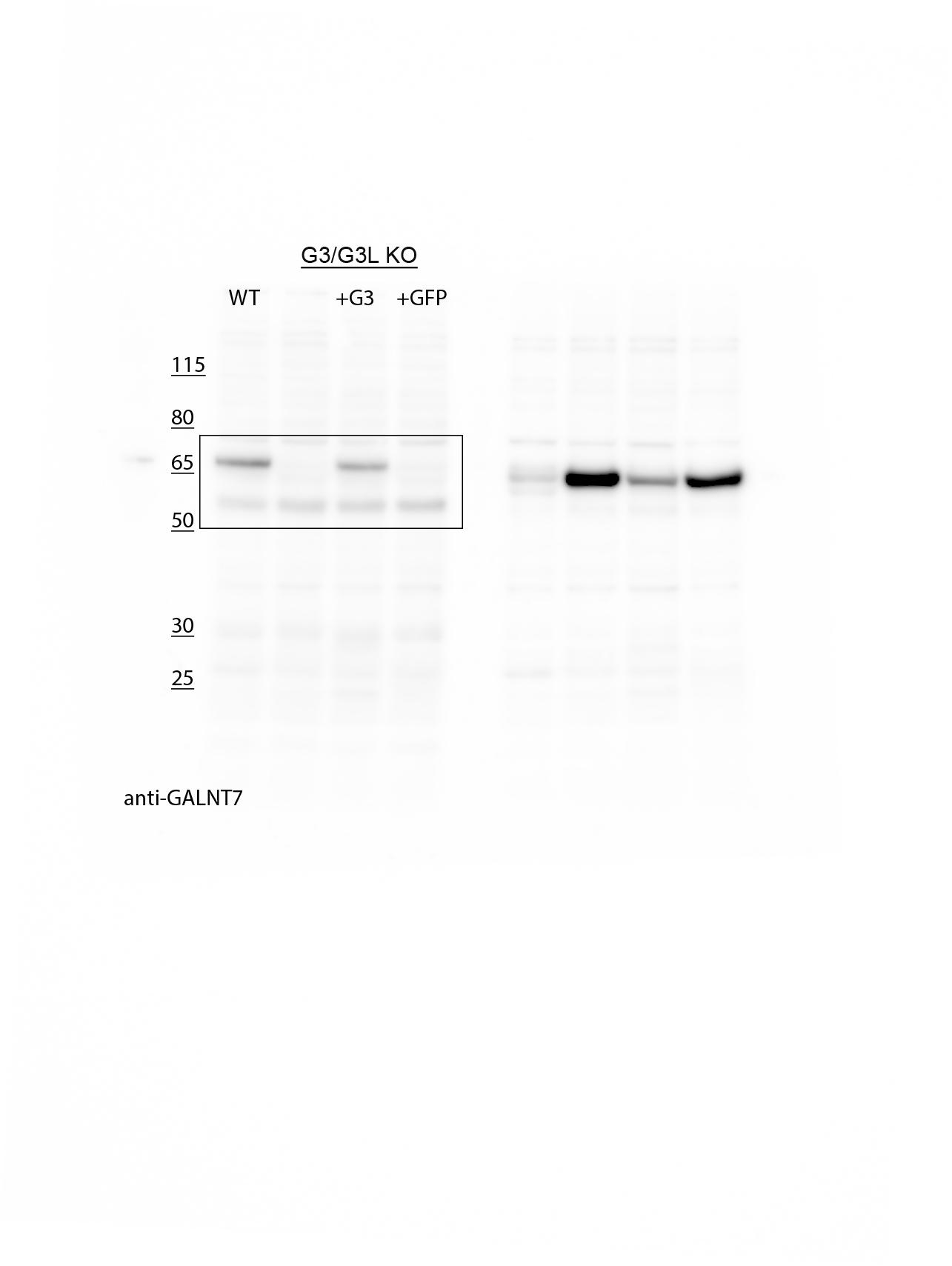

Supplement: Supplementary file 8 — Source data Fig. 5 [file 44318_2024_305_MOESM8_ESM.zip › Figure 5/5A/source data GALNT7 time series 20231004_145316-05_Ch_Chemi.tif]

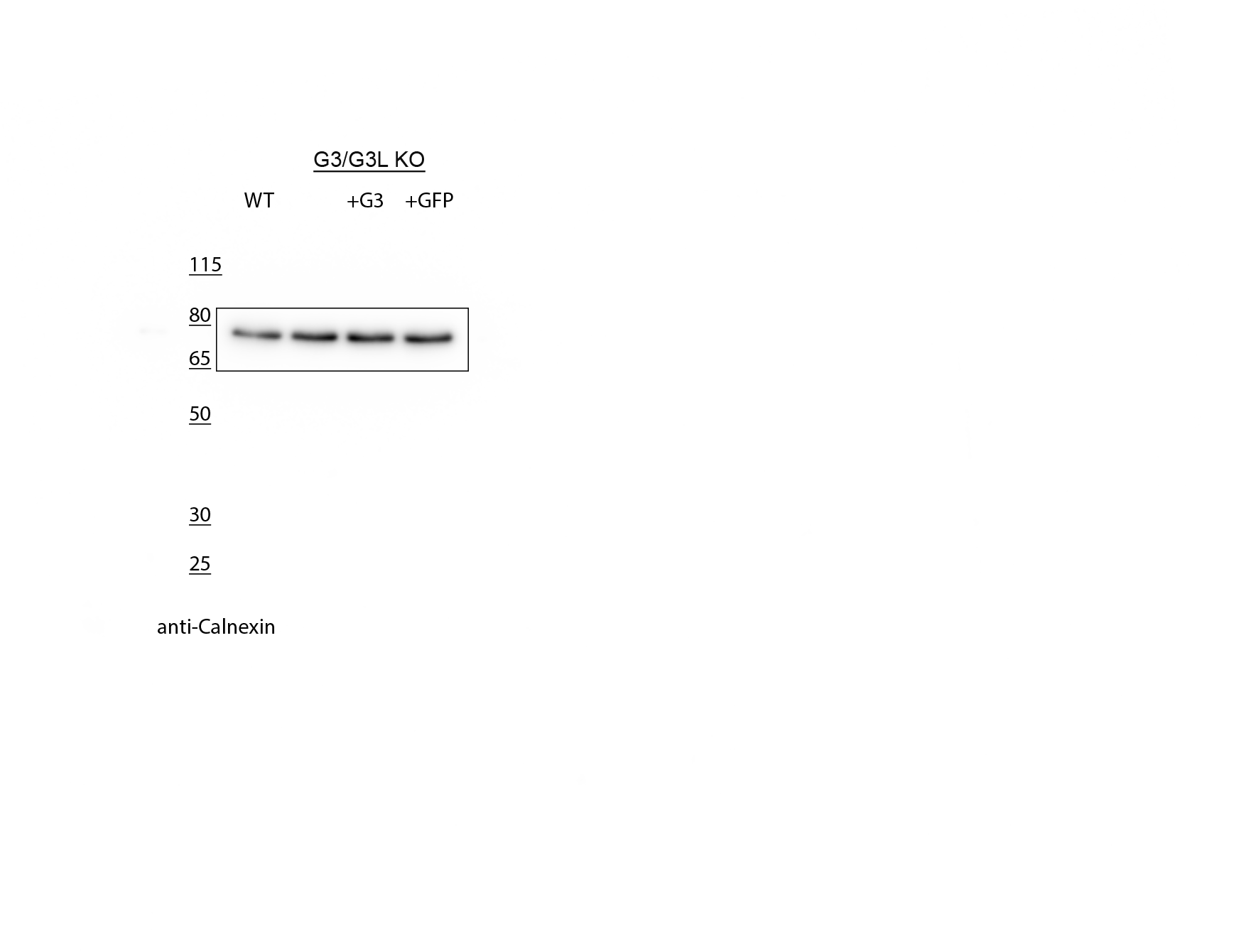

Supplement: Supplementary file 8 — Source data Fig. 5 [file 44318_2024_305_MOESM8_ESM.zip › Figure 5/5A/source data Calnexin for GALNT7 20240327_164557-02_Ch_Chemi.tif]

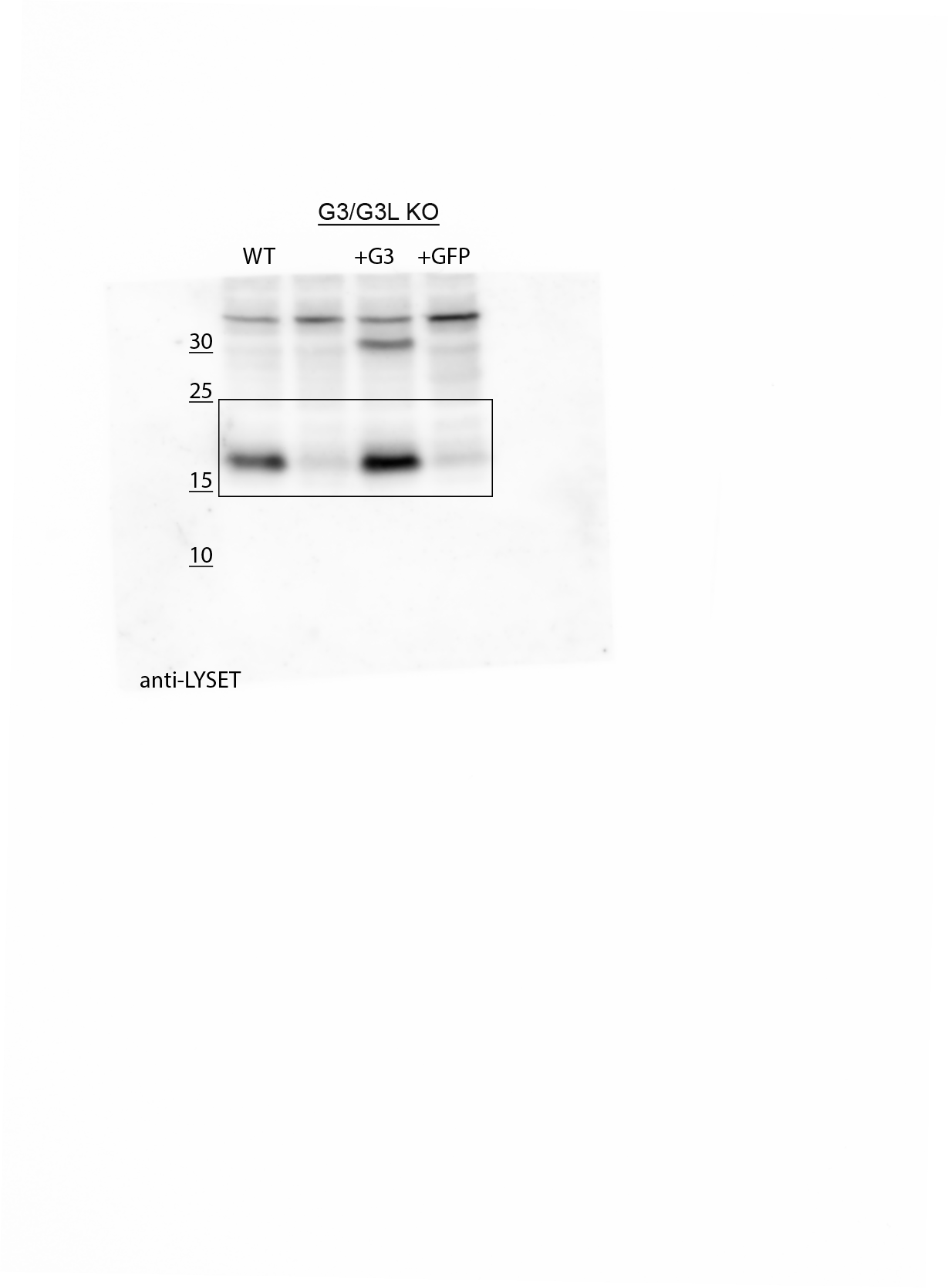

Supplement: Supplementary file 8 — Source data Fig. 5 [file 44318_2024_305_MOESM8_ESM.zip › Figure 5/5A/source data LYSET 20230928_152116-12_Ch_Chemi.tif]

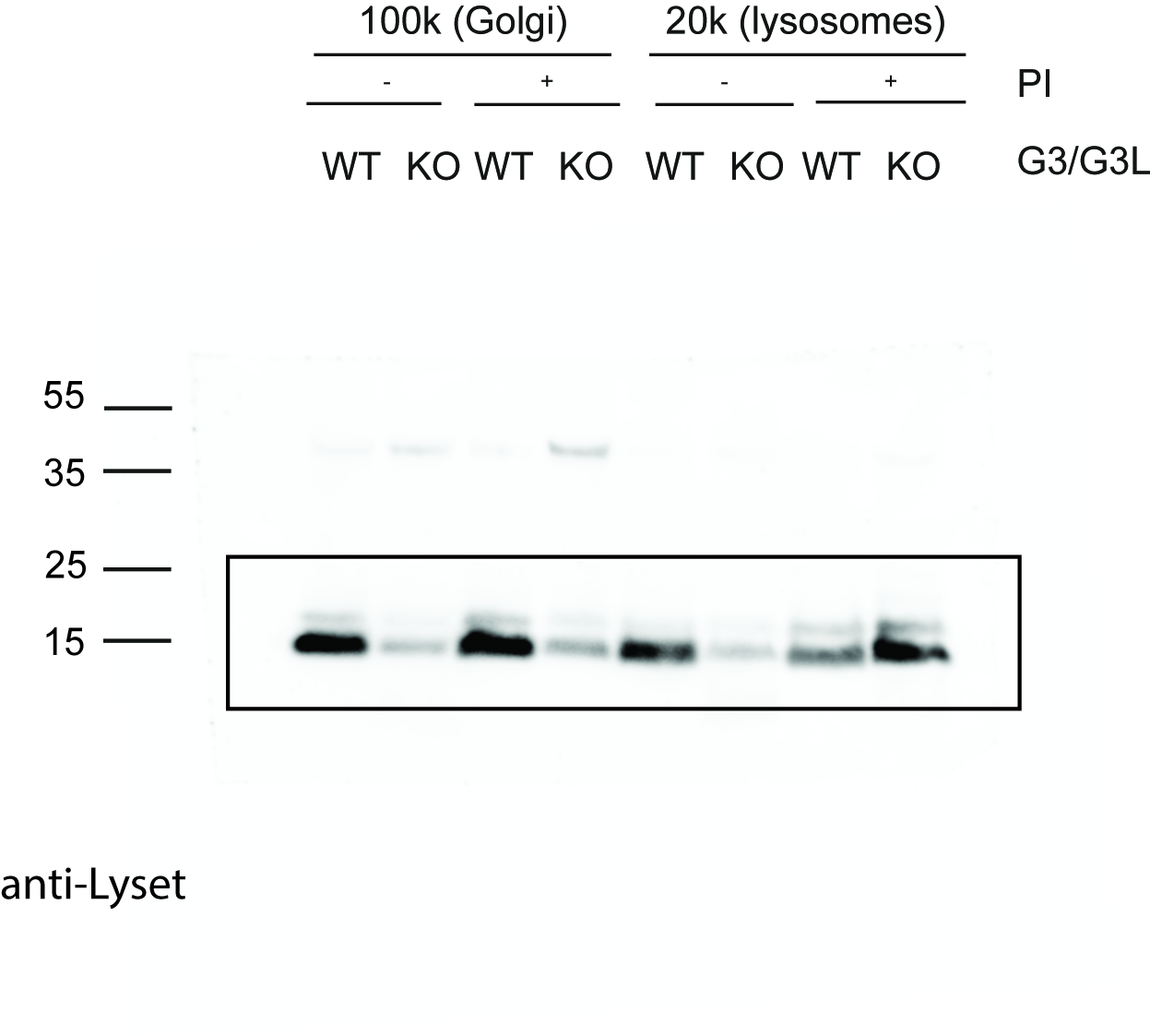

Supplement: Supplementary file 8 — Source data Fig. 5 [file 44318_2024_305_MOESM8_ESM.zip › Figure 5/5F/Lyset.tif]

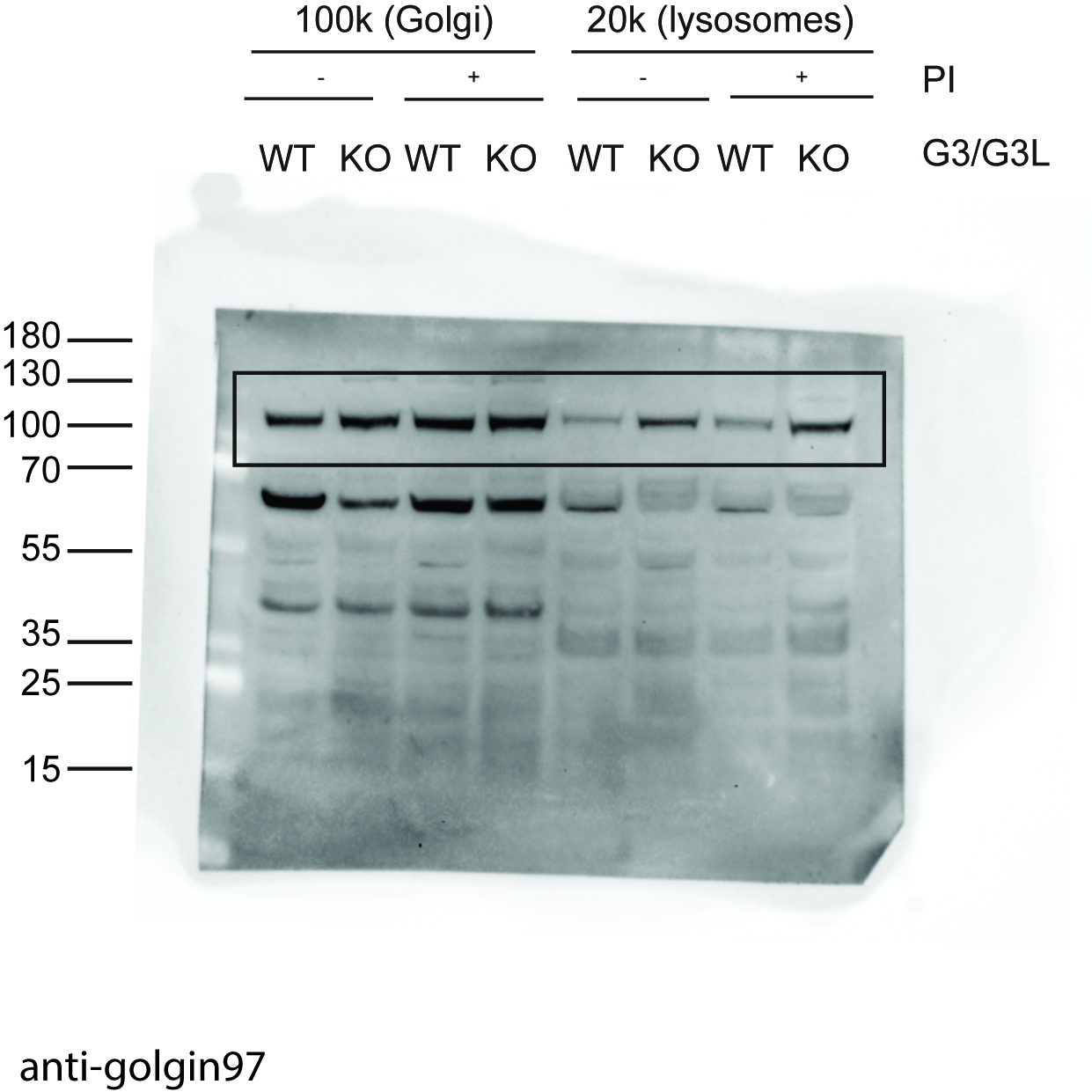

Supplement: Supplementary file 8 — Source data Fig. 5 [file 44318_2024_305_MOESM8_ESM.zip › Figure 5/5F/golgin97.tif]

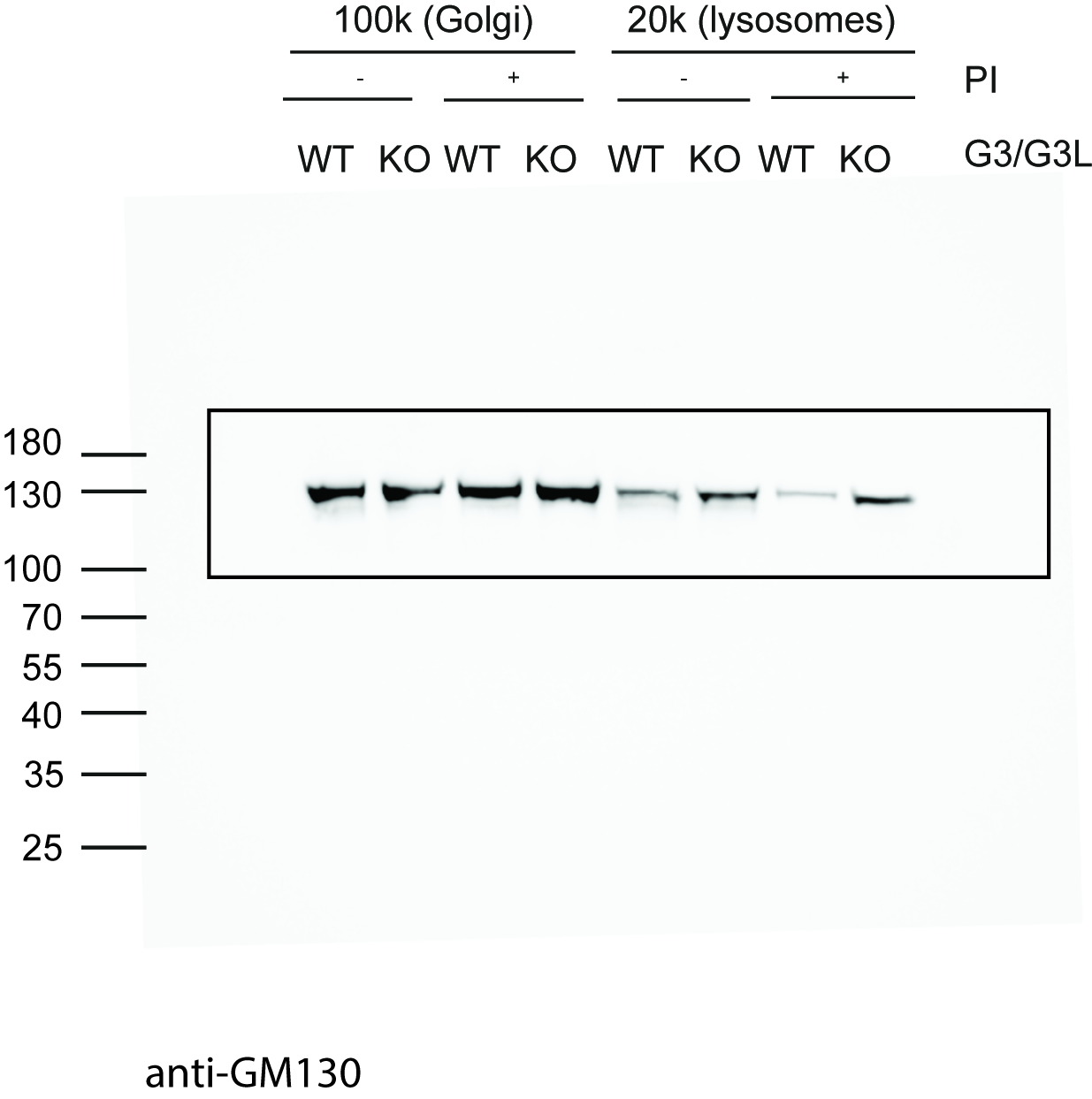

Supplement: Supplementary file 8 — Source data Fig. 5 [file 44318_2024_305_MOESM8_ESM.zip › Figure 5/5F/GM130.tif]

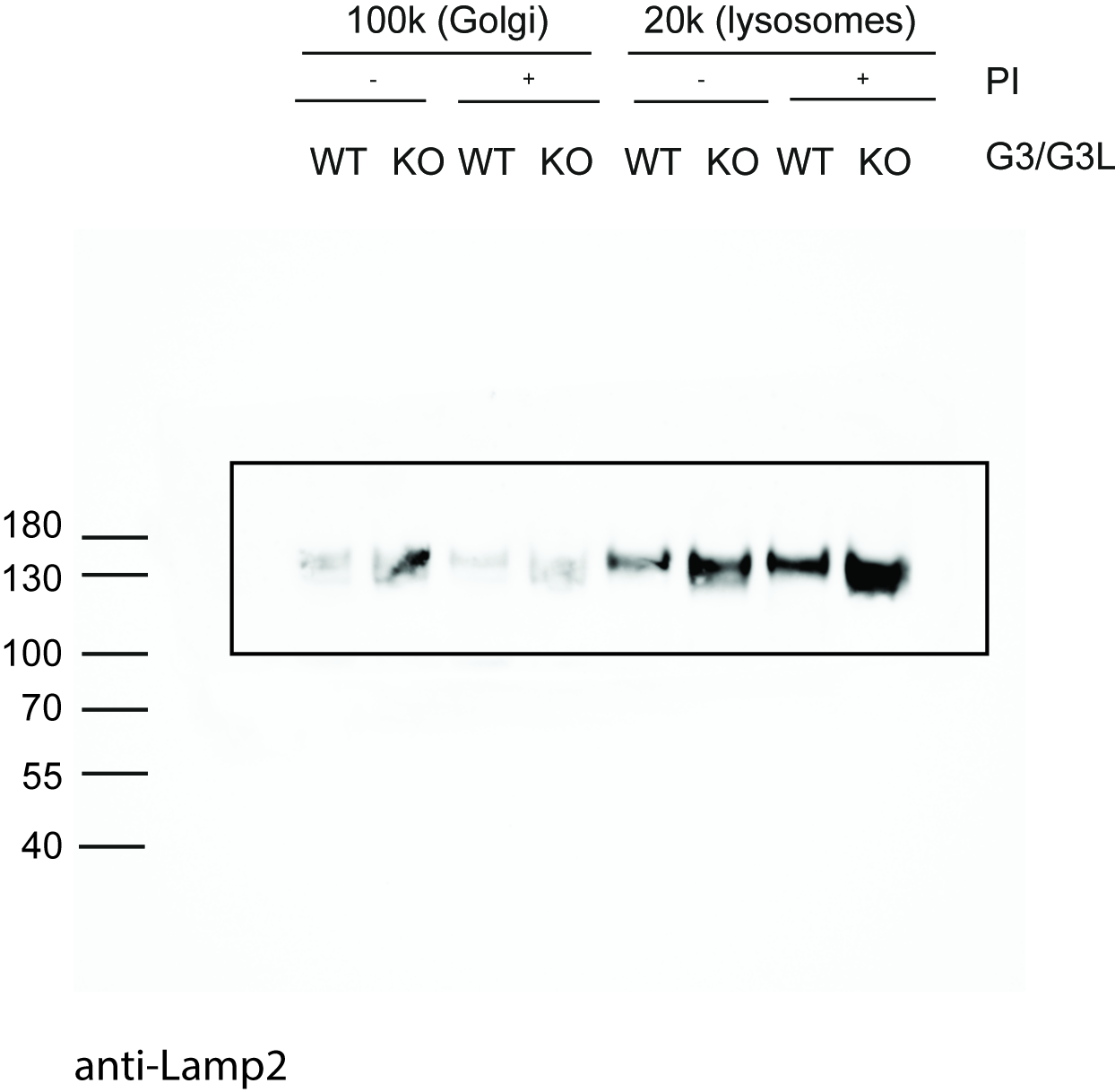

Supplement: Supplementary file 8 — Source data Fig. 5 [file 44318_2024_305_MOESM8_ESM.zip › Figure 5/5F/Lamp2.tif]

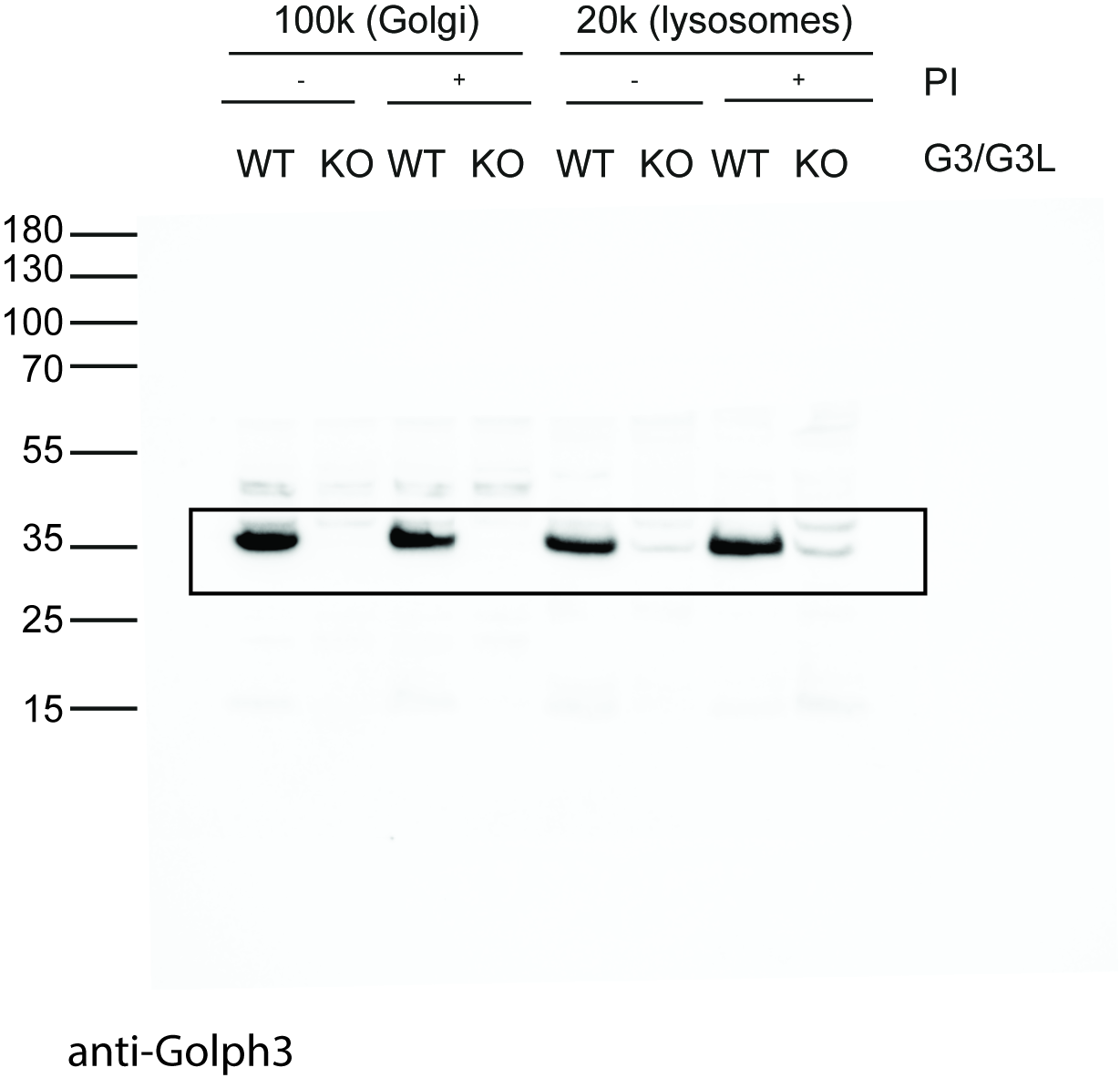

Supplement: Supplementary file 8 — Source data Fig. 5 [file 44318_2024_305_MOESM8_ESM.zip › Figure 5/5F/Golph3.tif]

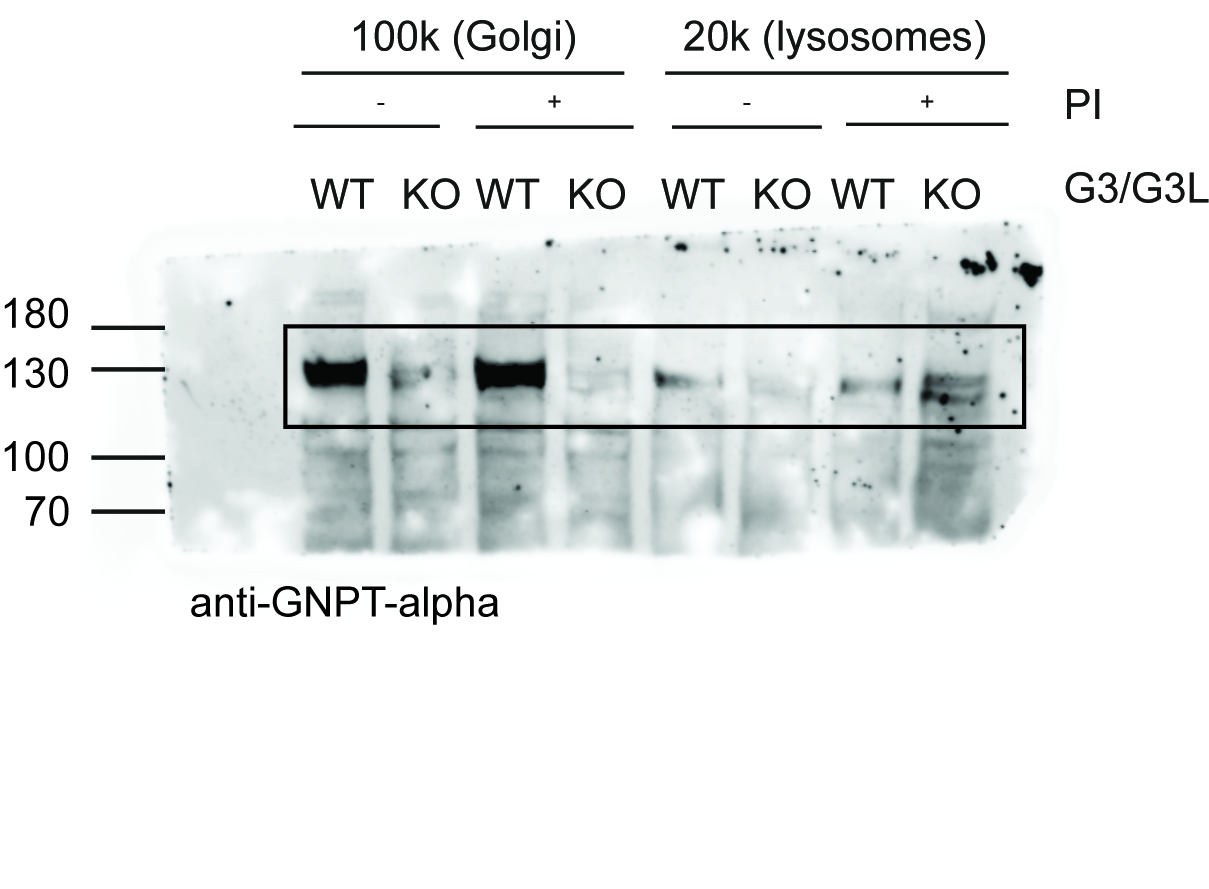

Supplement: Supplementary file 8 — Source data Fig. 5 [file 44318_2024_305_MOESM8_ESM.zip › Figure 5/5F/GNPTalpha.tif]

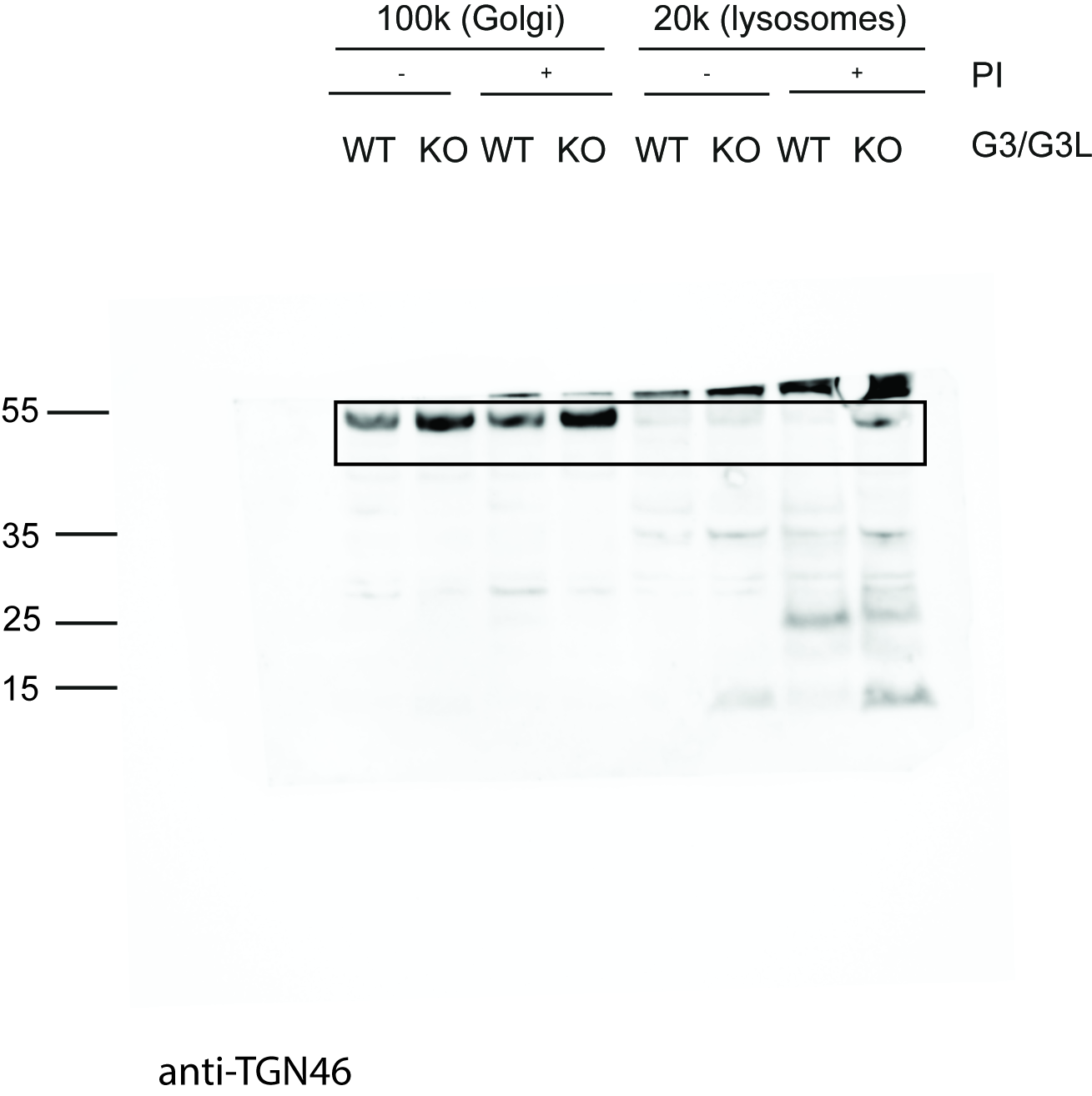

Supplement: Supplementary file 8 — Source data Fig. 5 [file 44318_2024_305_MOESM8_ESM.zip › Figure 5/5F/TGN46.tif]

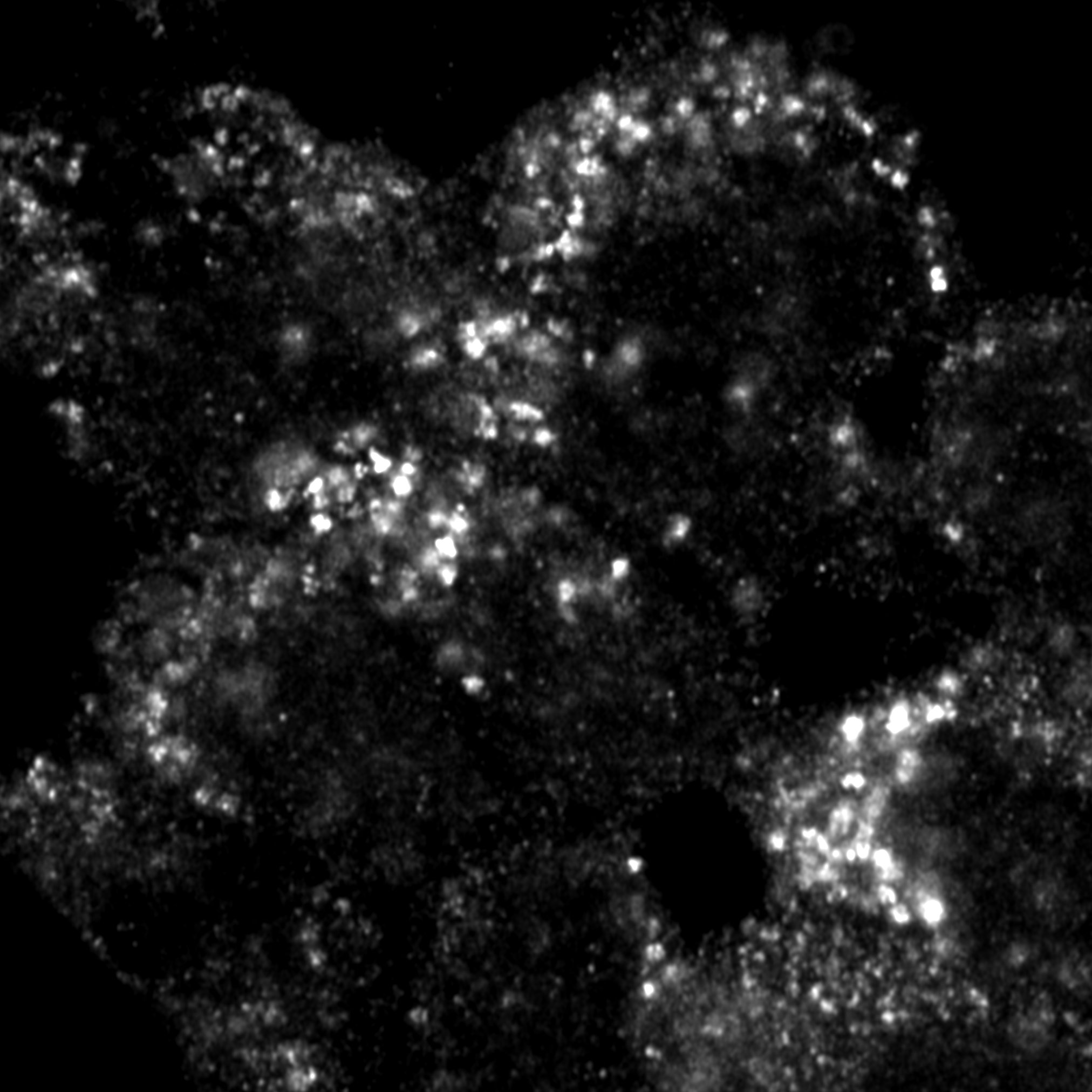

Supplement: Supplementary file 8 — Source data Fig. 5 [file 44318_2024_305_MOESM8_ESM.zip › Figure 5/5H/GOLPH_KO_PI_GOLPH_PT_1_(PT594_C=0)Airyscan Processing.tif]

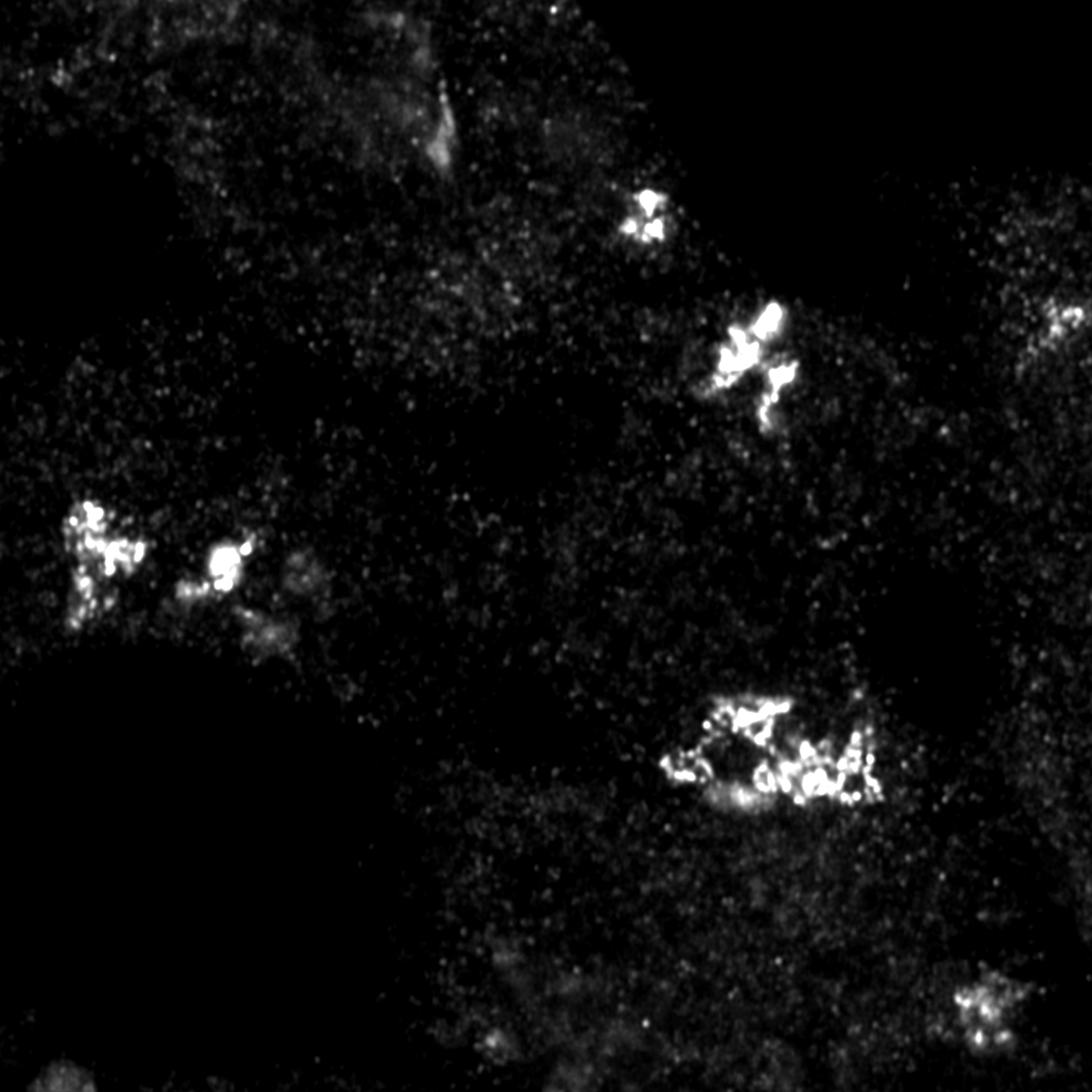

Supplement: Supplementary file 8 — Source data Fig. 5 [file 44318_2024_305_MOESM8_ESM.zip › Figure 5/5H/WT_ctrl_GOLPH_PT_1_(PT594_C=0)Airyscan Processing.tif]

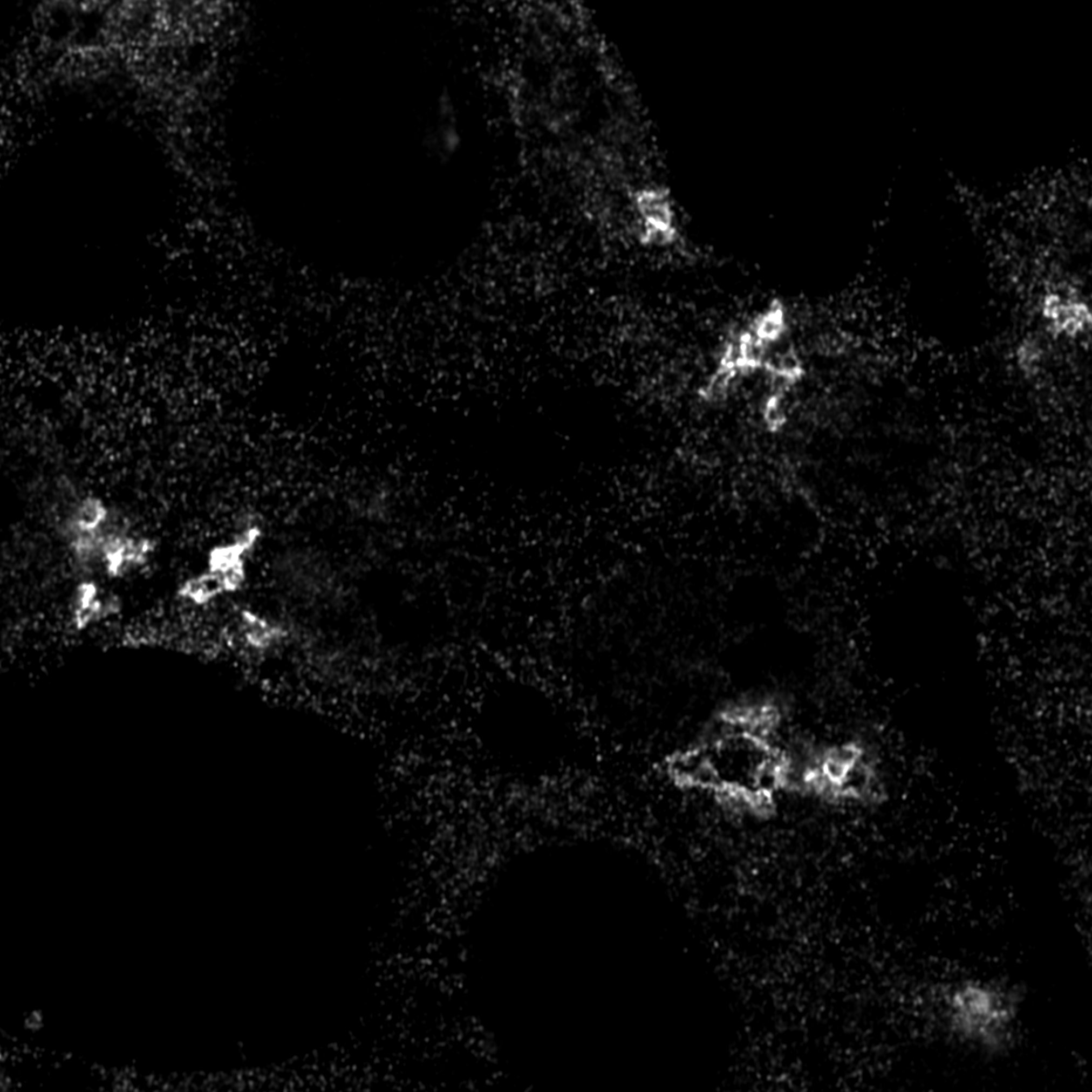

Supplement: Supplementary file 8 — Source data Fig. 5 [file 44318_2024_305_MOESM8_ESM.zip › Figure 5/5H/WT_ctrl_GOLPH_PT_1_(GOLPH488_C=1)Airyscan Processing.tif]

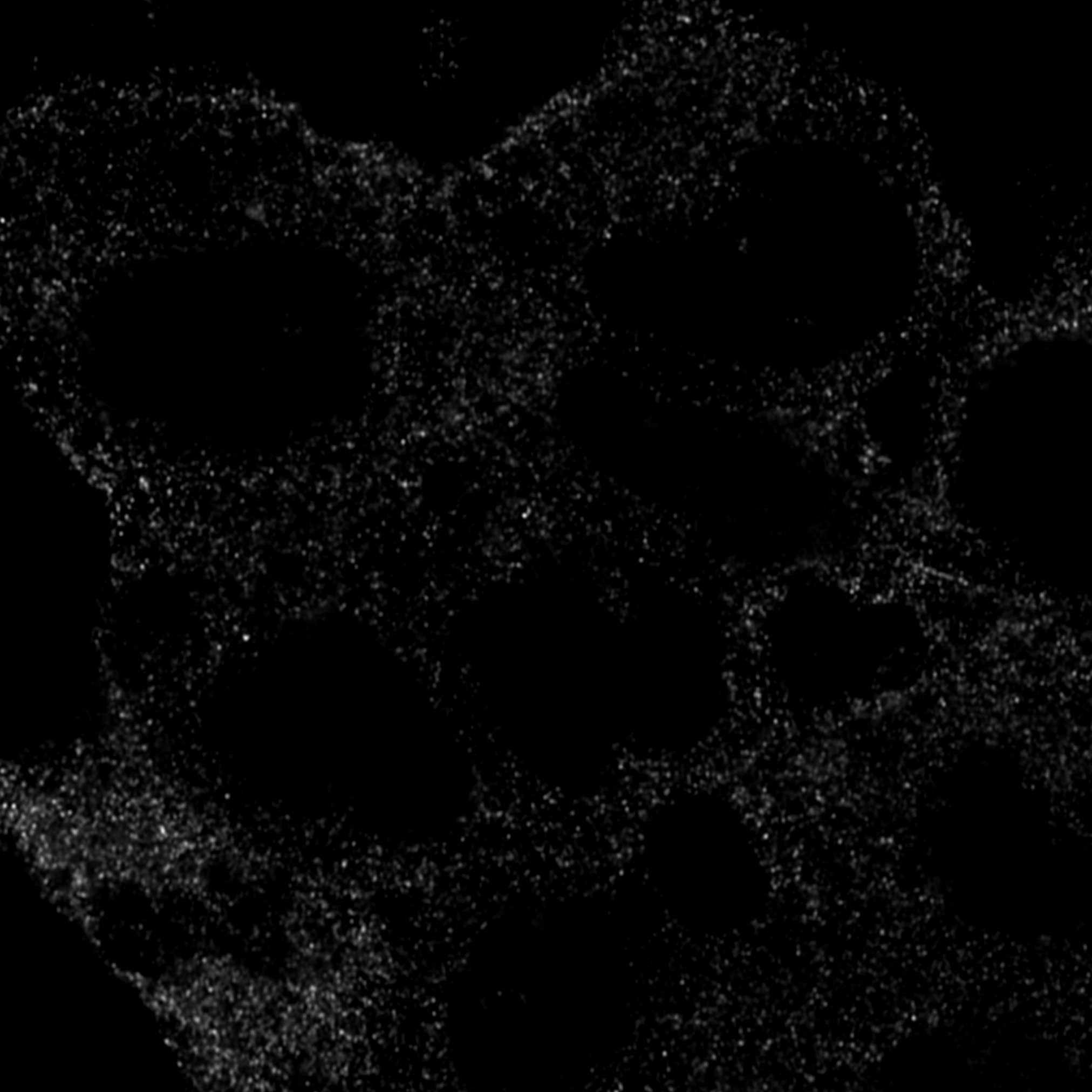

Supplement: Supplementary file 8 — Source data Fig. 5 [file 44318_2024_305_MOESM8_ESM.zip › Figure 5/5H/GOLPH_KO_PI_GOLPH_PT_1_(GOLPH488_C=1)Airyscan Processing.tif]
