# Supplementary figures and images for: GOLPH3 and GOLPH3L maintain Golgi localization of LYSET and a functional mannose 6-phosphate transport pathway (part 3 of 4)
Source: EMBO J. 2024 Nov 25;43(24):6264–90. doi: 10.1038/s44318-024-00305-z (PMC11649813; doi:10.1038/s44318-024-00305-z)

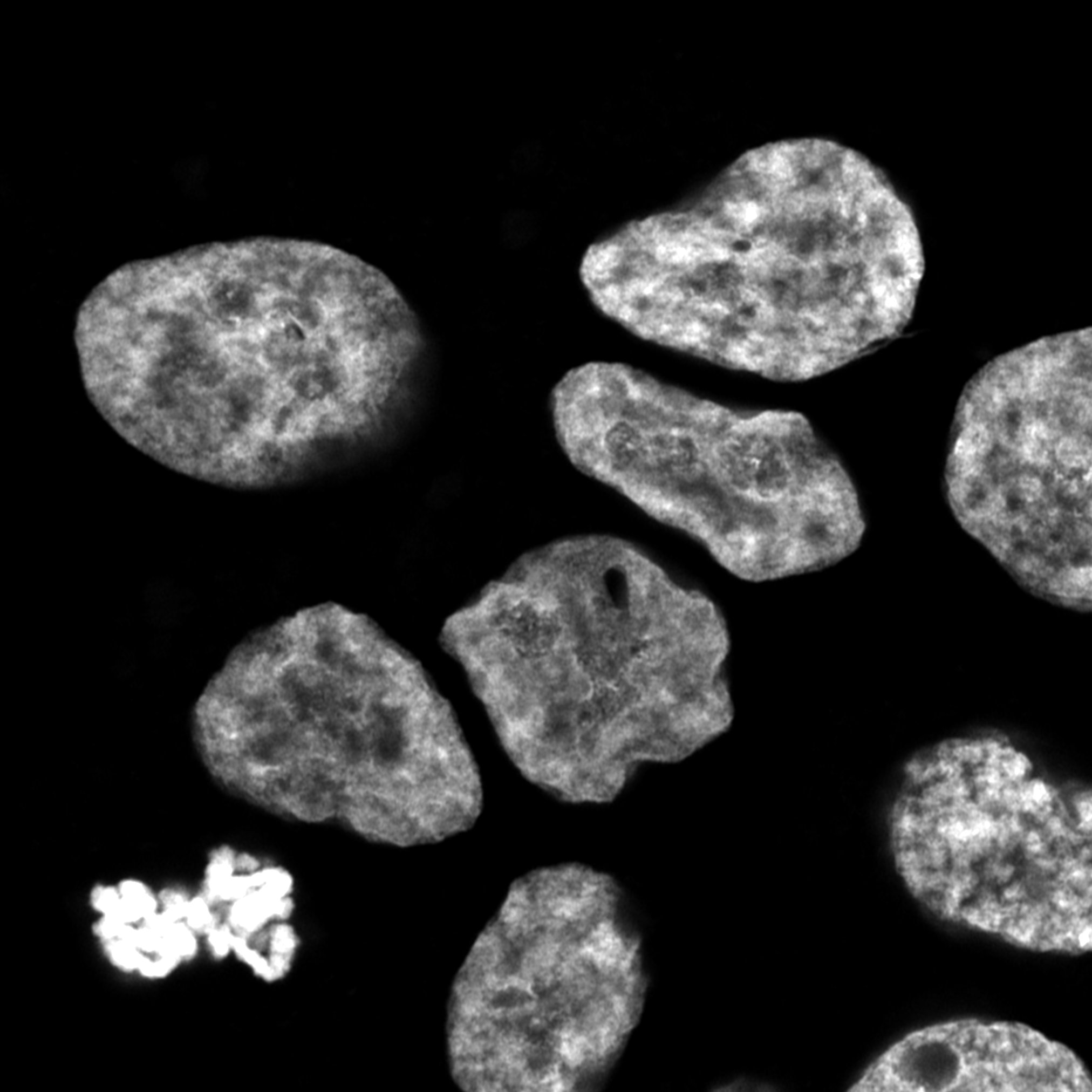

Supplement: Supplementary file 8 — Source data Fig. 5 [file 44318_2024_305_MOESM8_ESM.zip › Figure 5/5H/GOLPH_KO_PI_GOLPH_PT_1_(Hoechst_C=2)Airyscan Processing.tif]

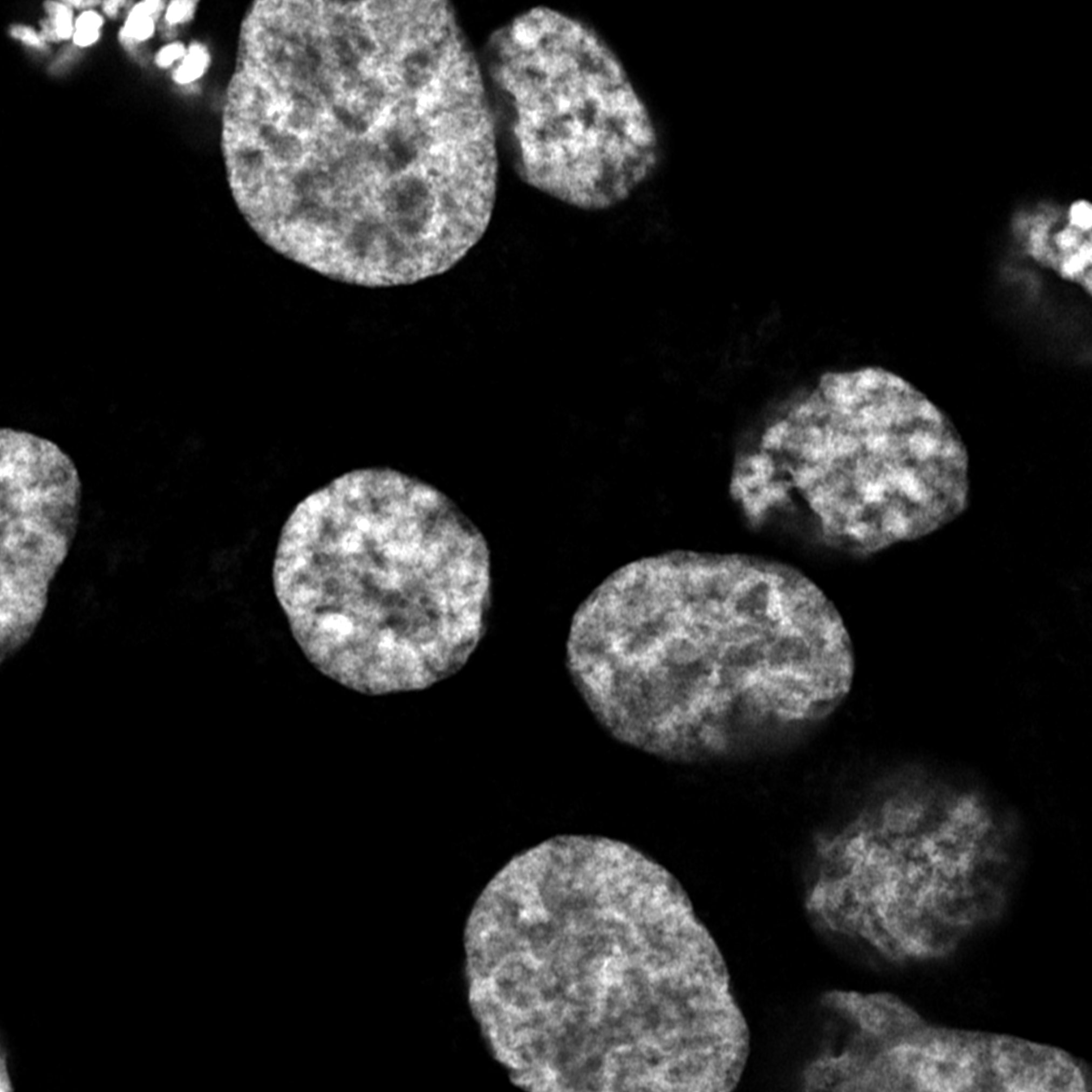

Supplement: Supplementary file 8 — Source data Fig. 5 [file 44318_2024_305_MOESM8_ESM.zip › Figure 5/5H/WT_ctrl_GOLPH_PT_1_(Hoechst_C=2)Airyscan Processing.tif]

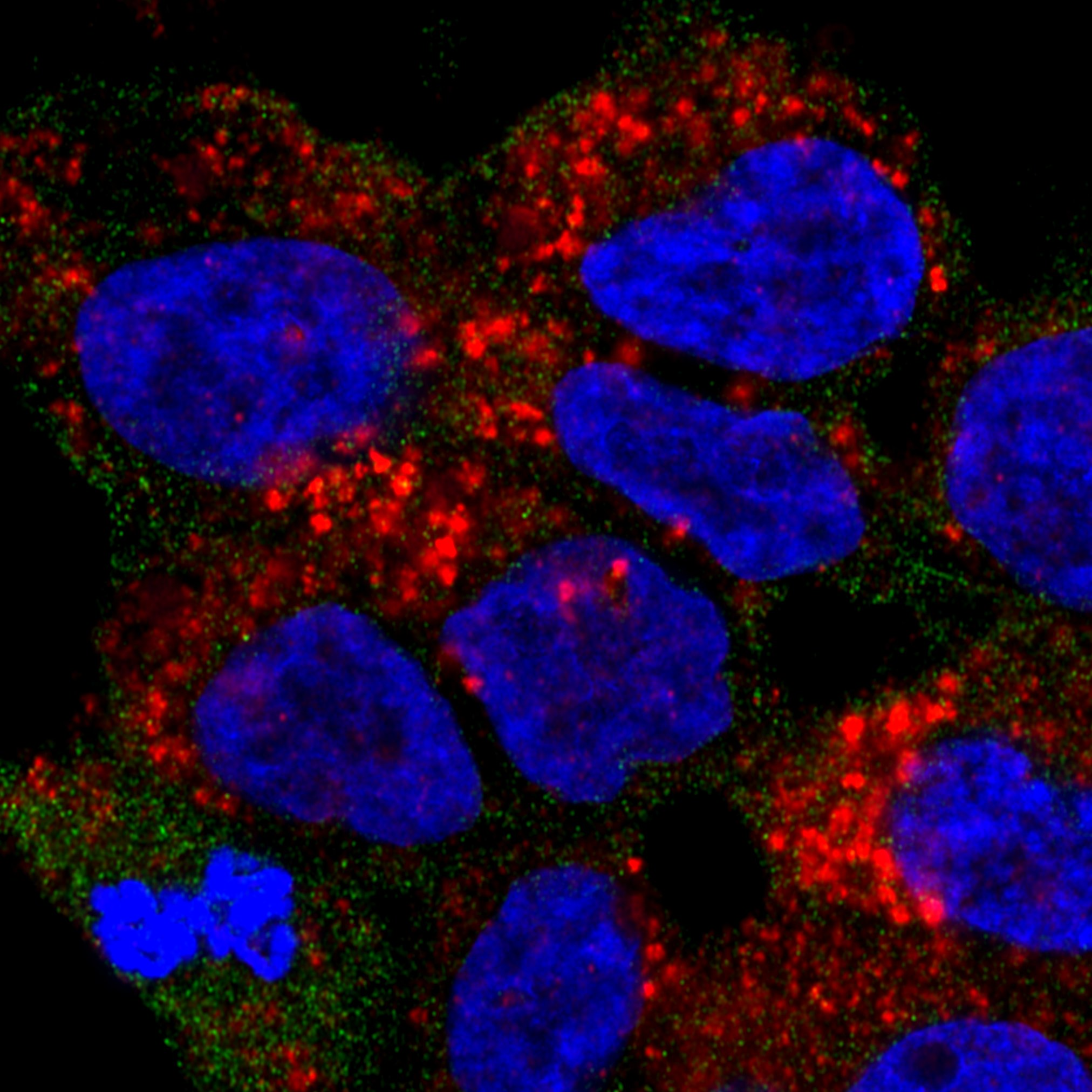

Supplement: Supplementary file 8 — Source data Fig. 5 [file 44318_2024_305_MOESM8_ESM.zip › Figure 5/5H/GOLPH_KO_PI_GOLPH_PT_1_(merge)Airyscan Processing.tif]

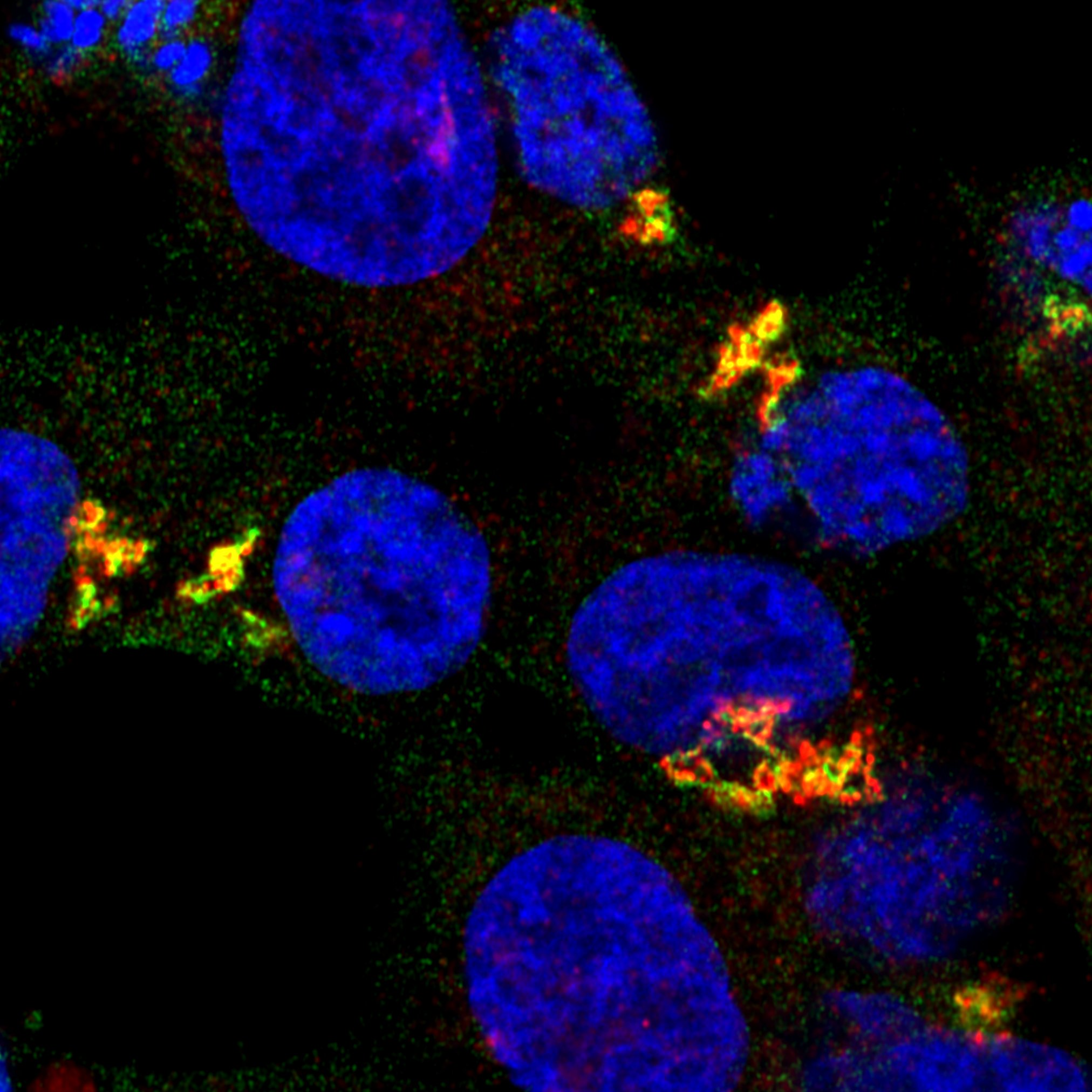

Supplement: Supplementary file 8 — Source data Fig. 5 [file 44318_2024_305_MOESM8_ESM.zip › Figure 5/5H/WT_ctrl_GOLPH_PT_1_(merge)Airyscan Processing.tif]

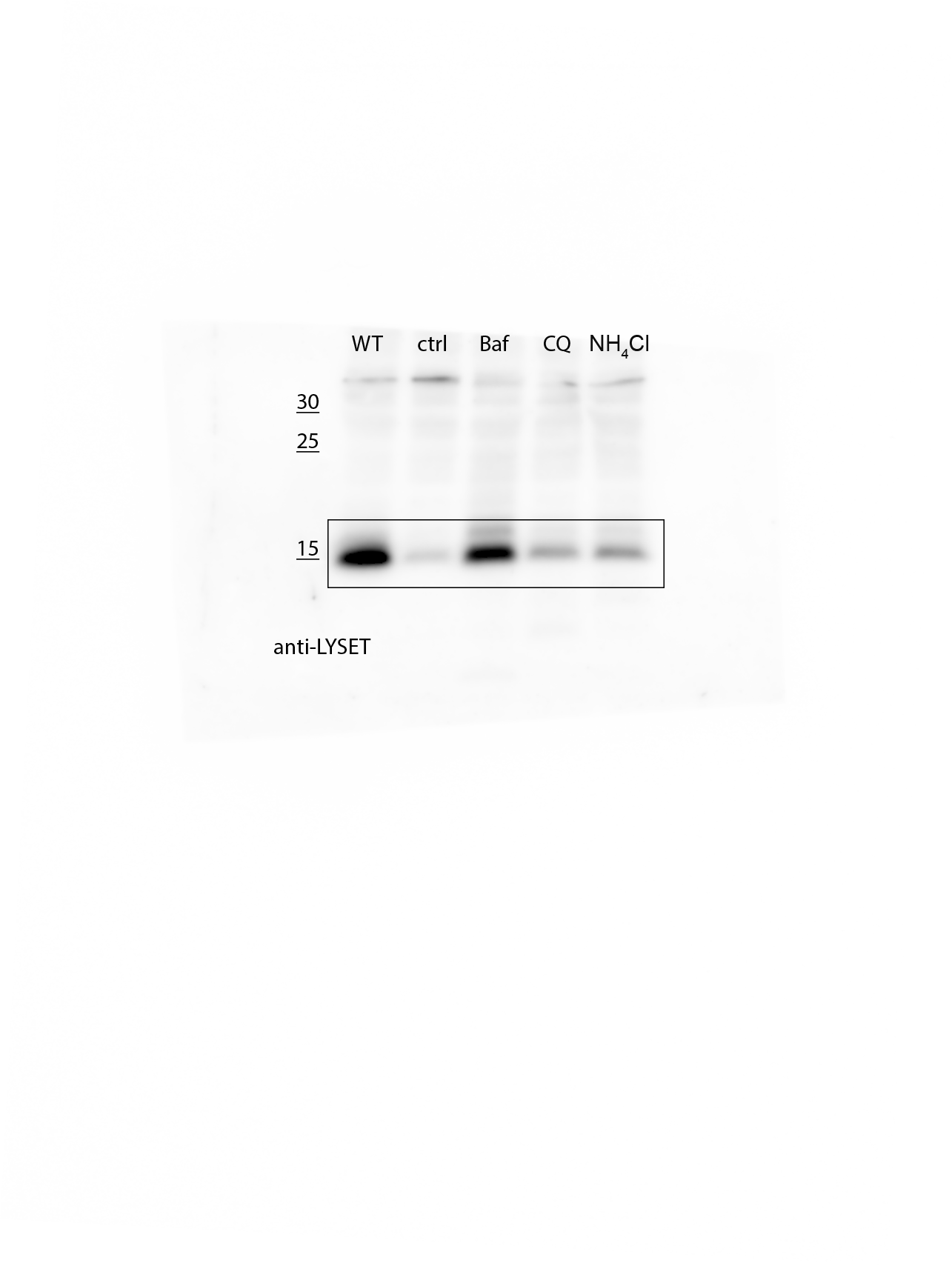

Supplement: Supplementary file 8 — Source data Fig. 5 [file 44318_2024_305_MOESM8_ESM.zip › Figure 5/5D/source data LYSET.tif]

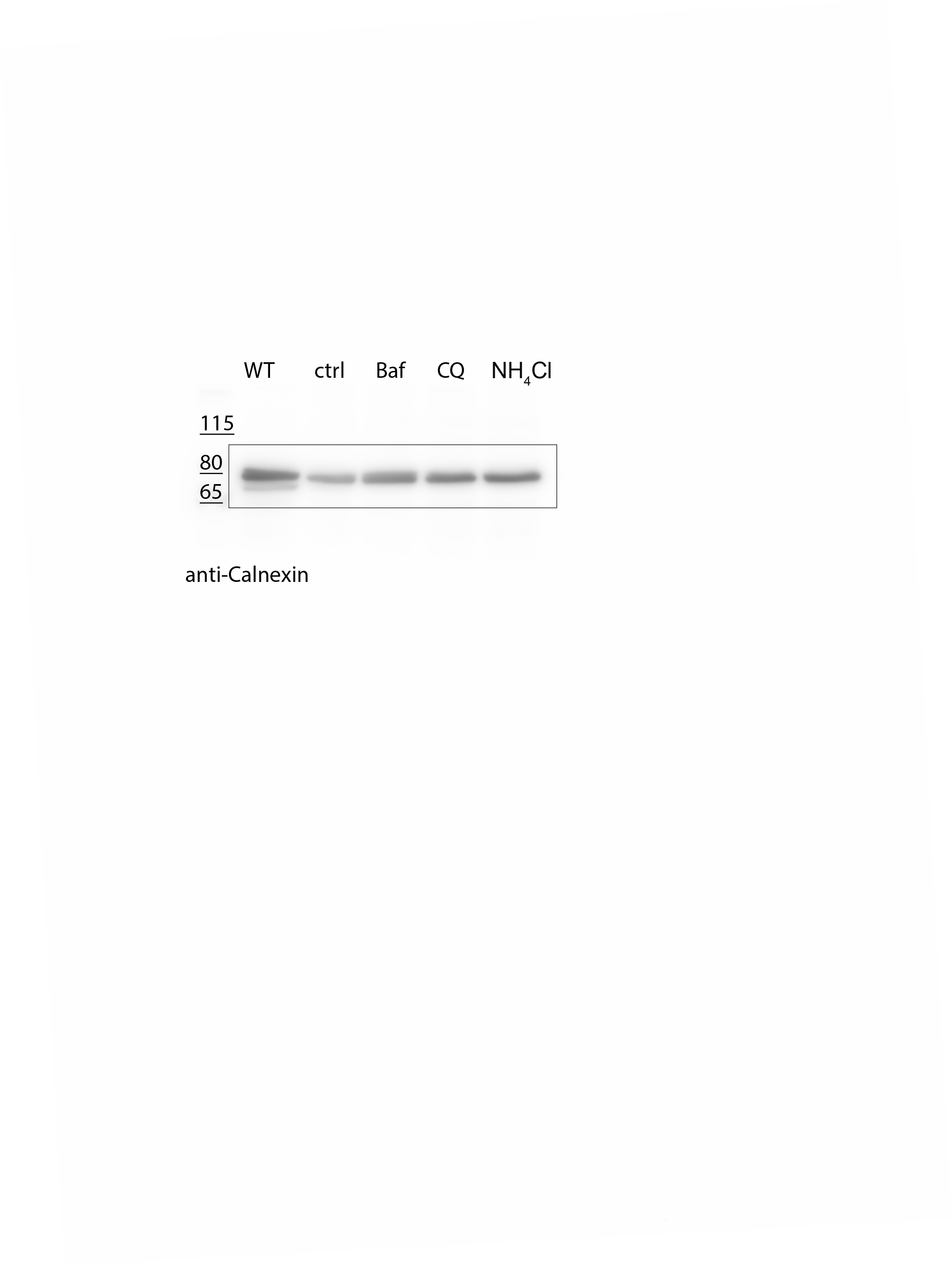

Supplement: Supplementary file 8 — Source data Fig. 5 [file 44318_2024_305_MOESM8_ESM.zip › Figure 5/5D/source data Calnexin.tif]

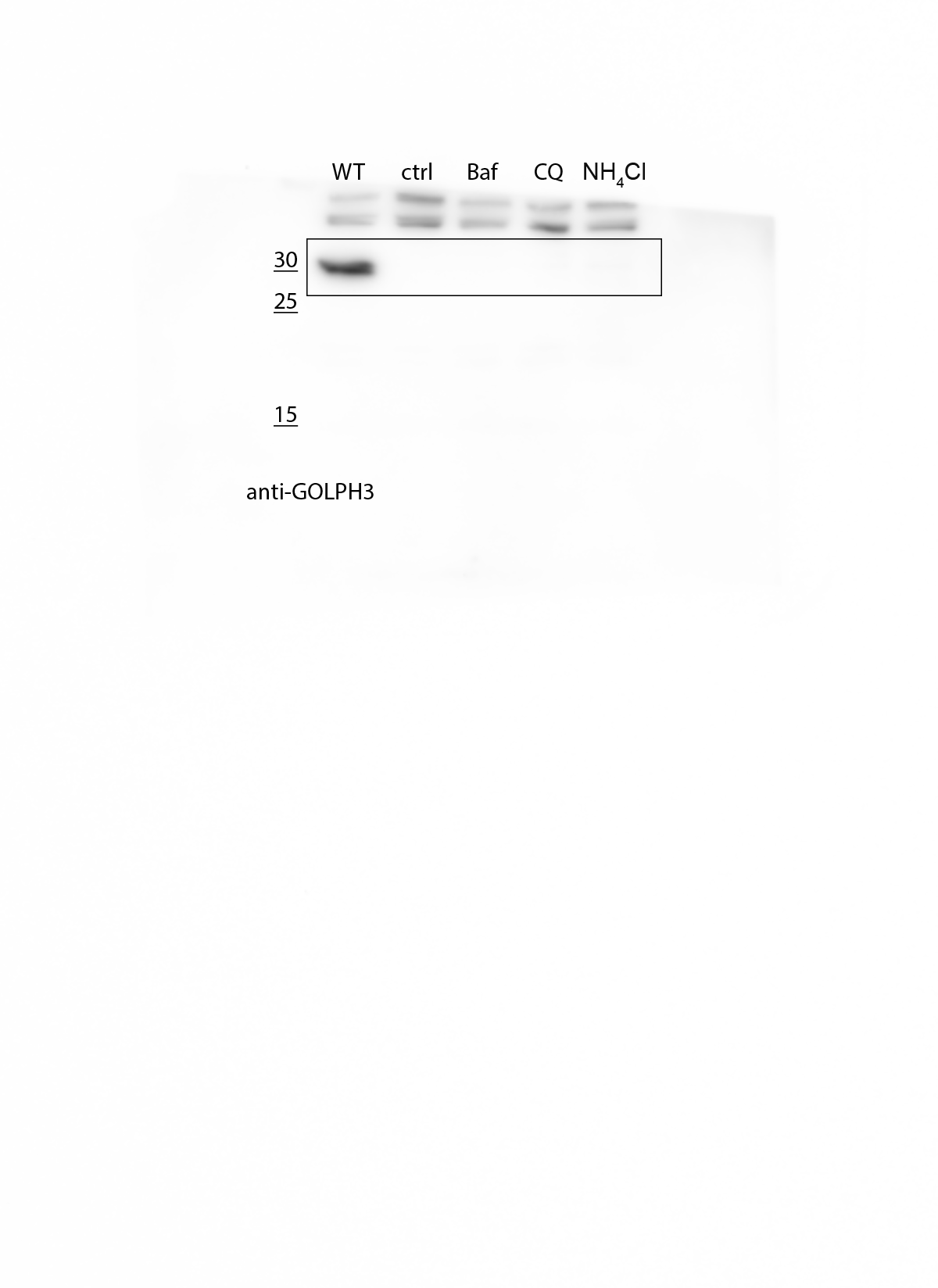

Supplement: Supplementary file 8 — Source data Fig. 5 [file 44318_2024_305_MOESM8_ESM.zip › Figure 5/5D/source data GOLPH3.tif]

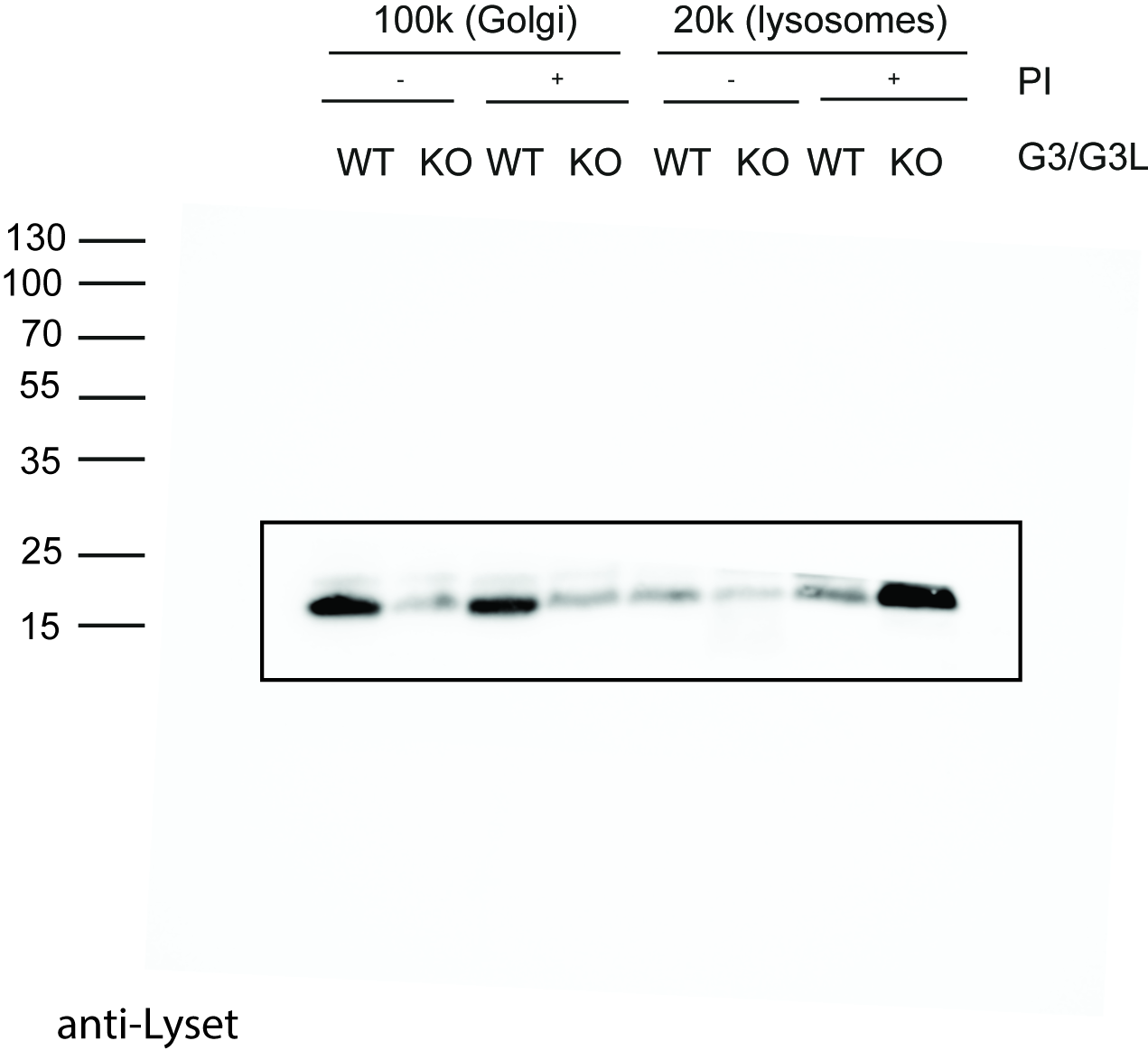

Supplement: Supplementary file 8 — Source data Fig. 5 [file 44318_2024_305_MOESM8_ESM.zip › Figure 5/5E/Lyset.tif]

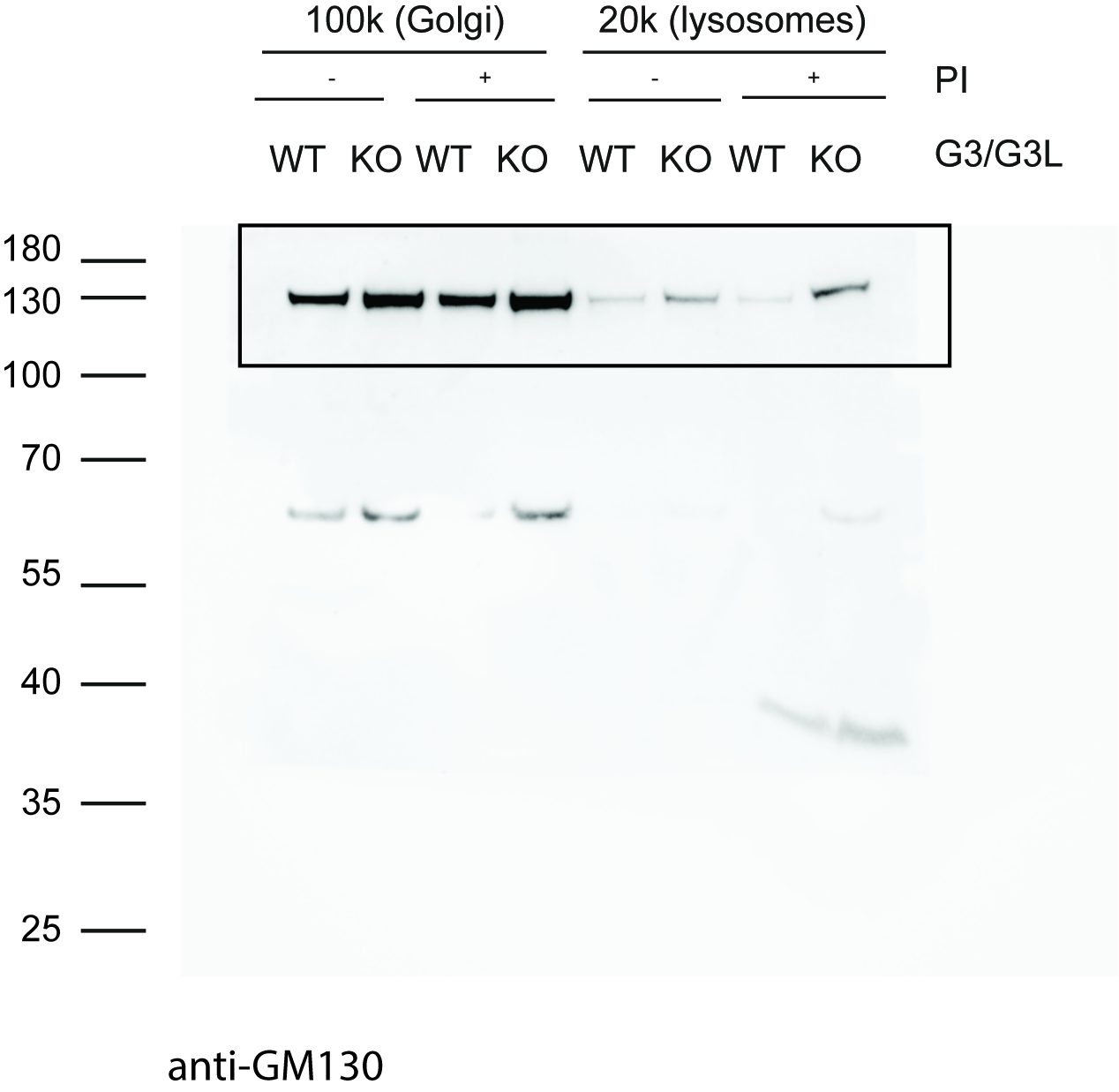

Supplement: Supplementary file 8 — Source data Fig. 5 [file 44318_2024_305_MOESM8_ESM.zip › Figure 5/5E/GM130.tif]

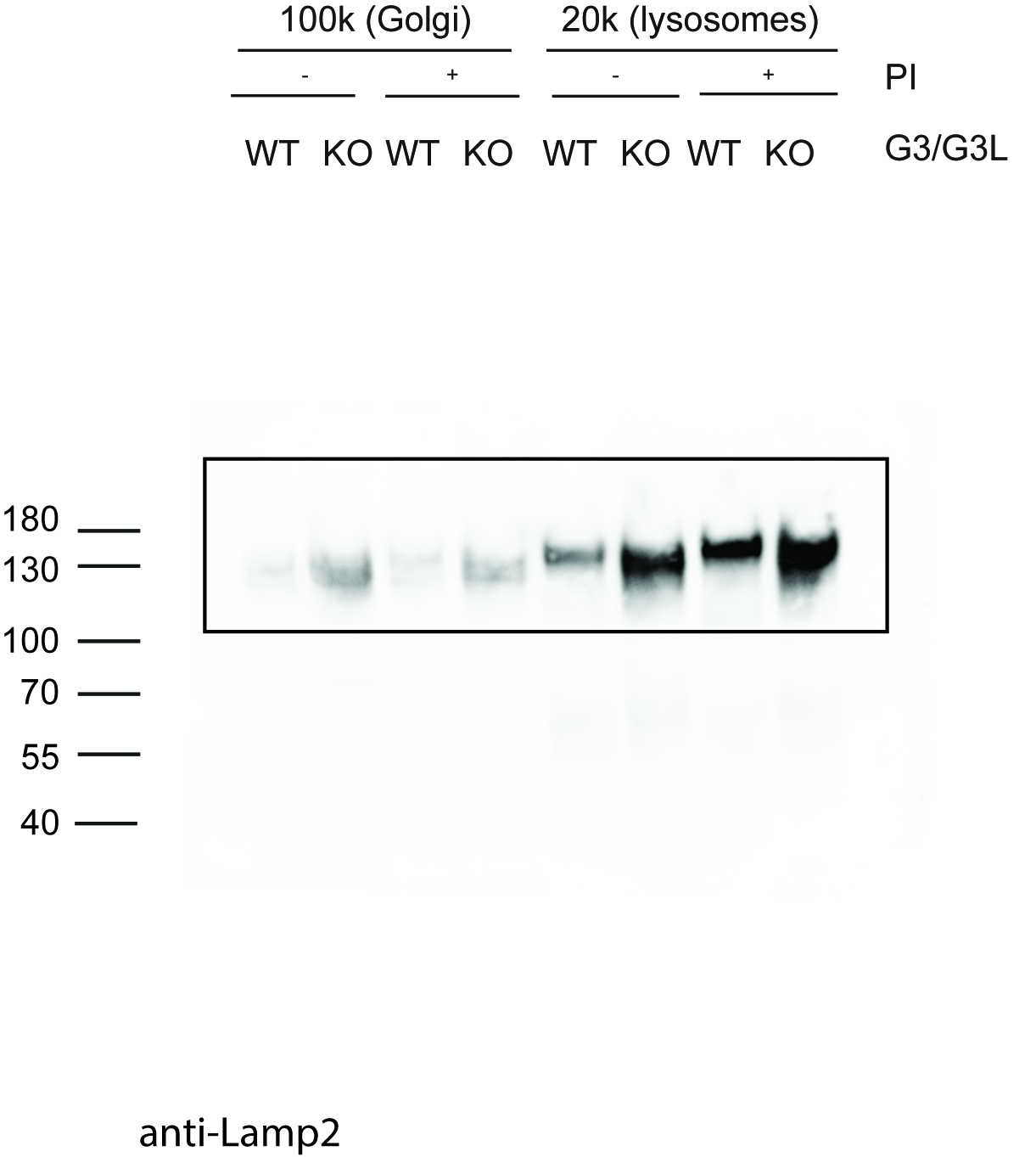

Supplement: Supplementary file 8 — Source data Fig. 5 [file 44318_2024_305_MOESM8_ESM.zip › Figure 5/5E/Lamp2.tif]

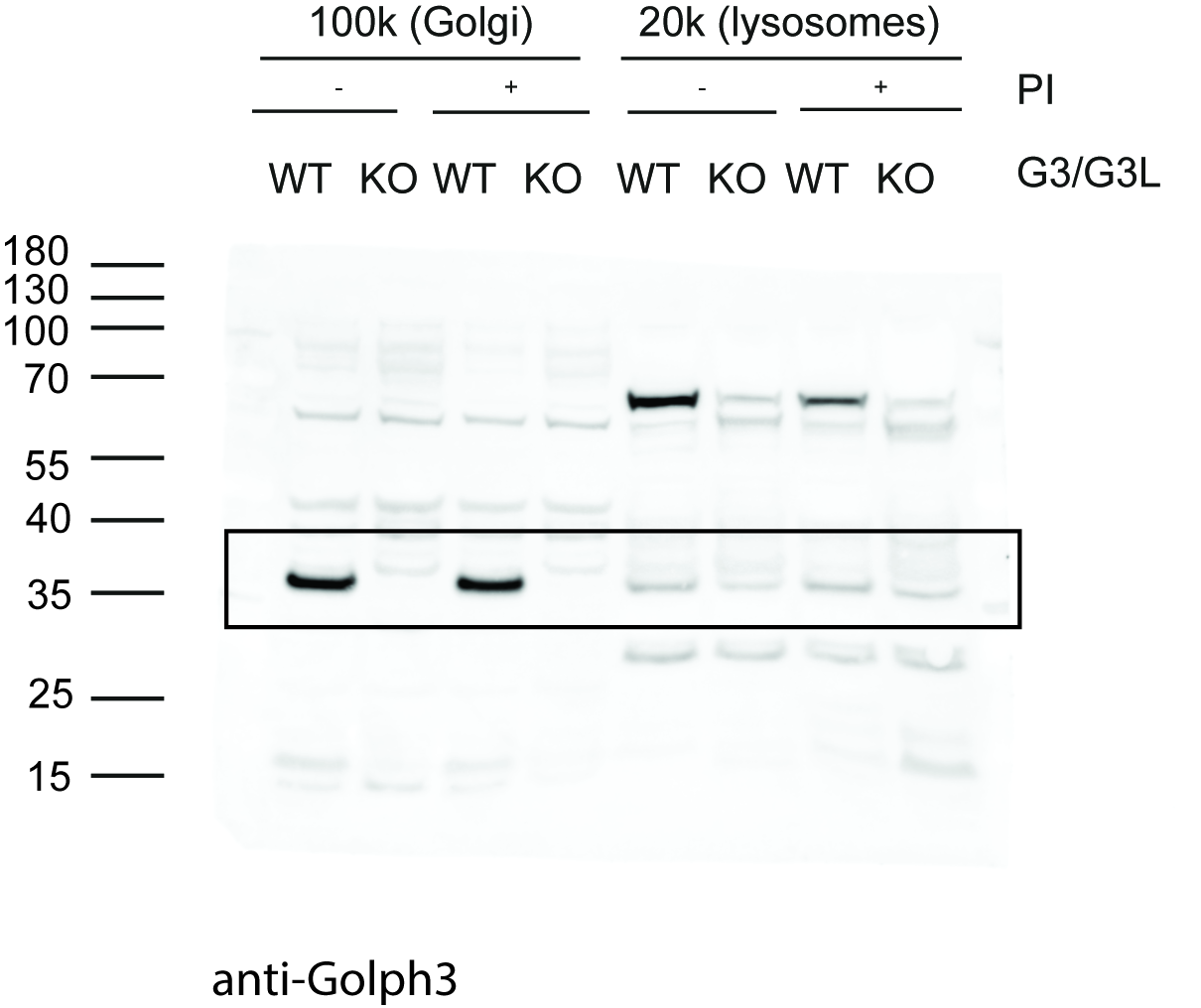

Supplement: Supplementary file 8 — Source data Fig. 5 [file 44318_2024_305_MOESM8_ESM.zip › Figure 5/5E/Golph3.tif]

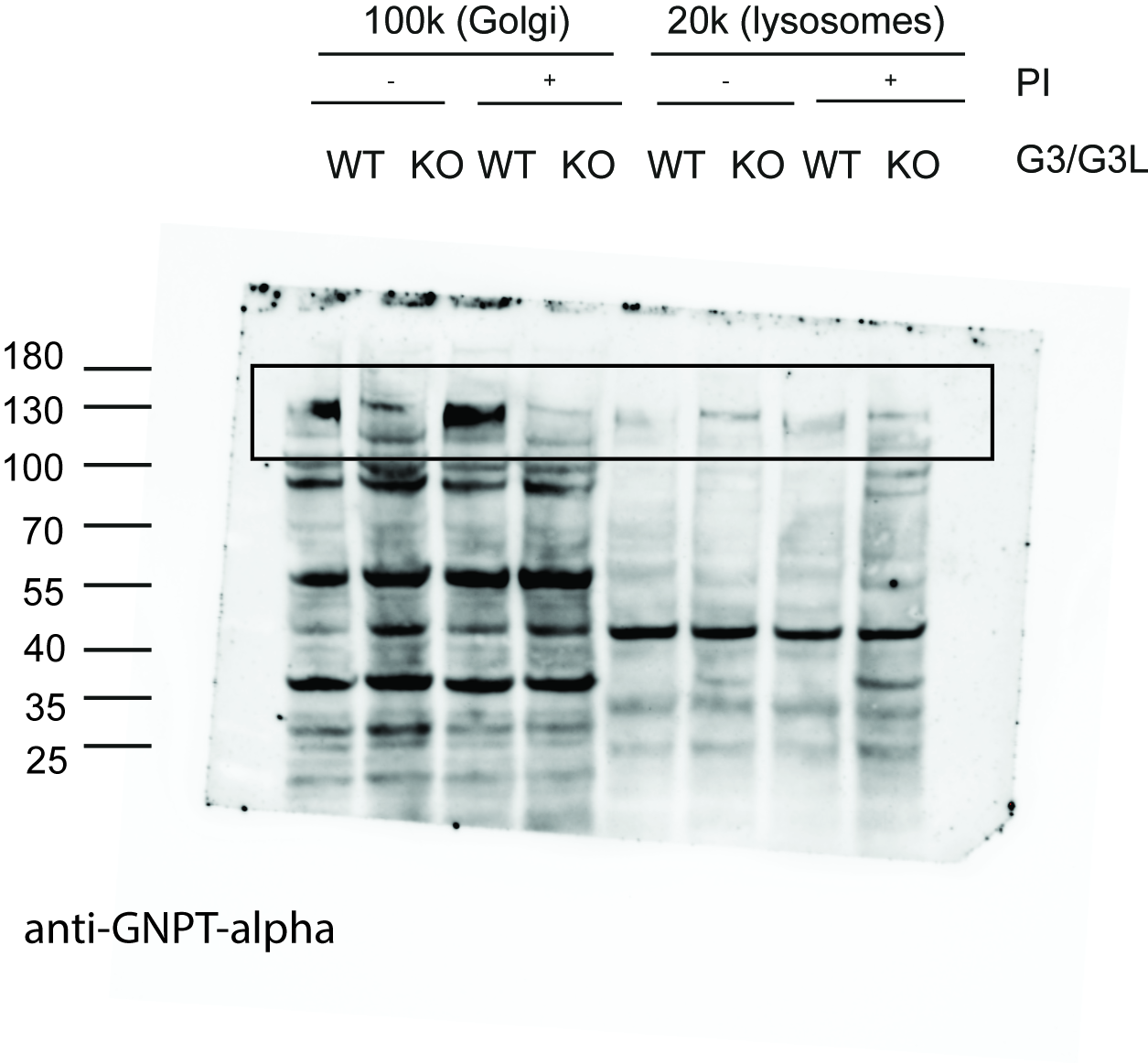

Supplement: Supplementary file 8 — Source data Fig. 5 [file 44318_2024_305_MOESM8_ESM.zip › Figure 5/5E/GNPTalpha.tif]

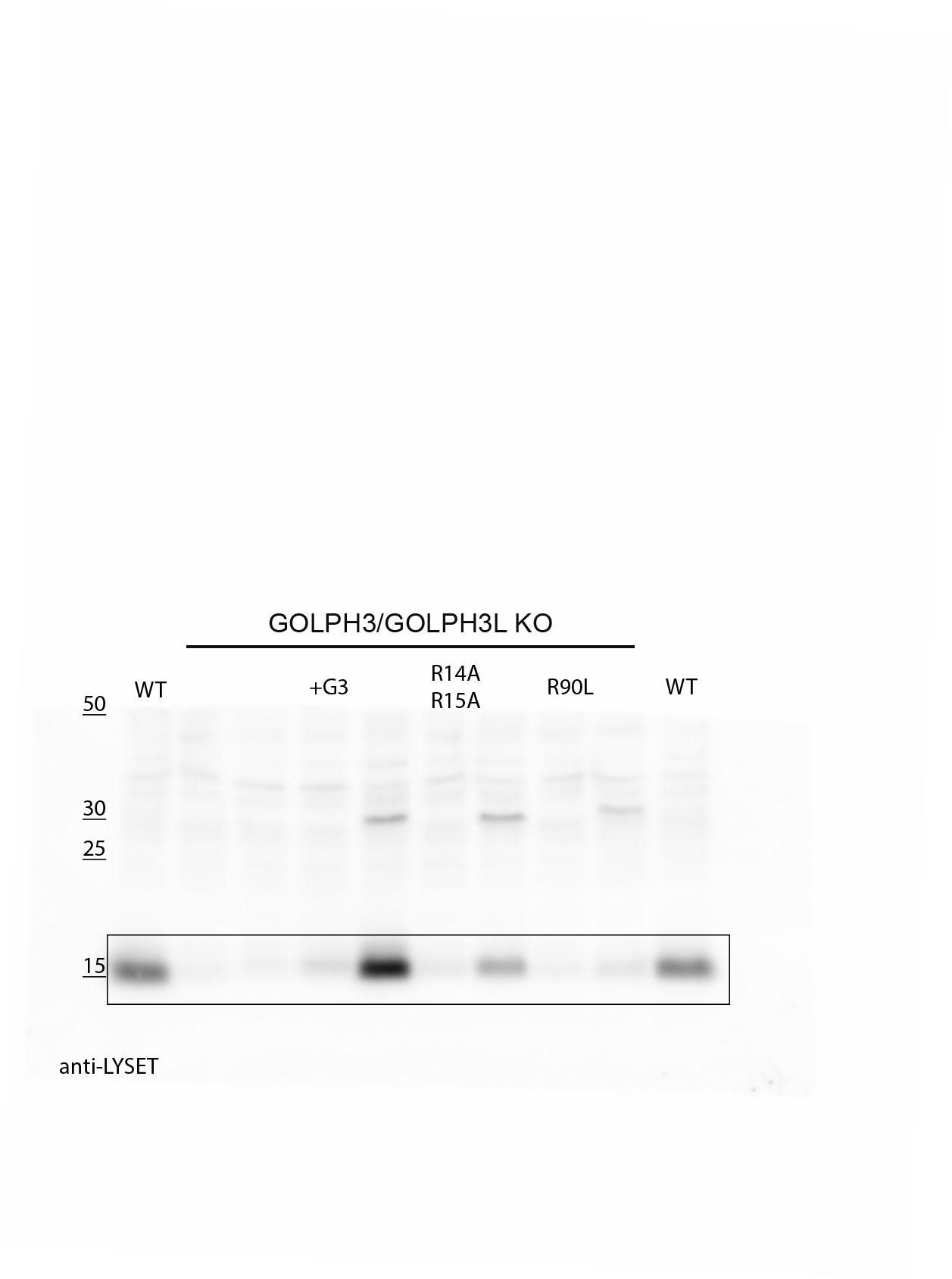

Supplement: Supplementary file 8 — Source data Fig. 5 [file 44318_2024_305_MOESM8_ESM.zip › Figure 5/5B/source data LYSET.tif]

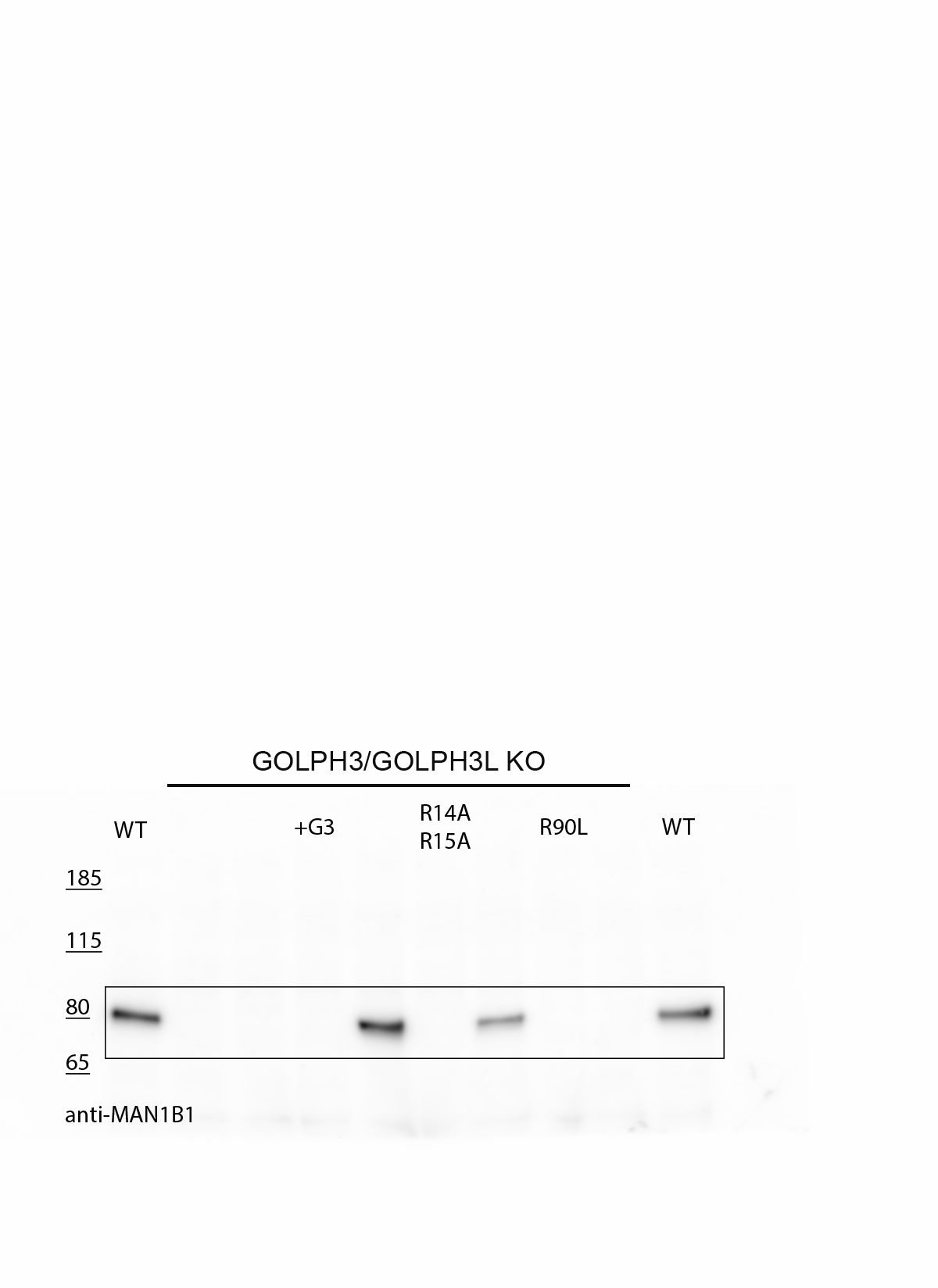

Supplement: Supplementary file 8 — Source data Fig. 5 [file 44318_2024_305_MOESM8_ESM.zip › Figure 5/5B/source data MAN1B1.tif]

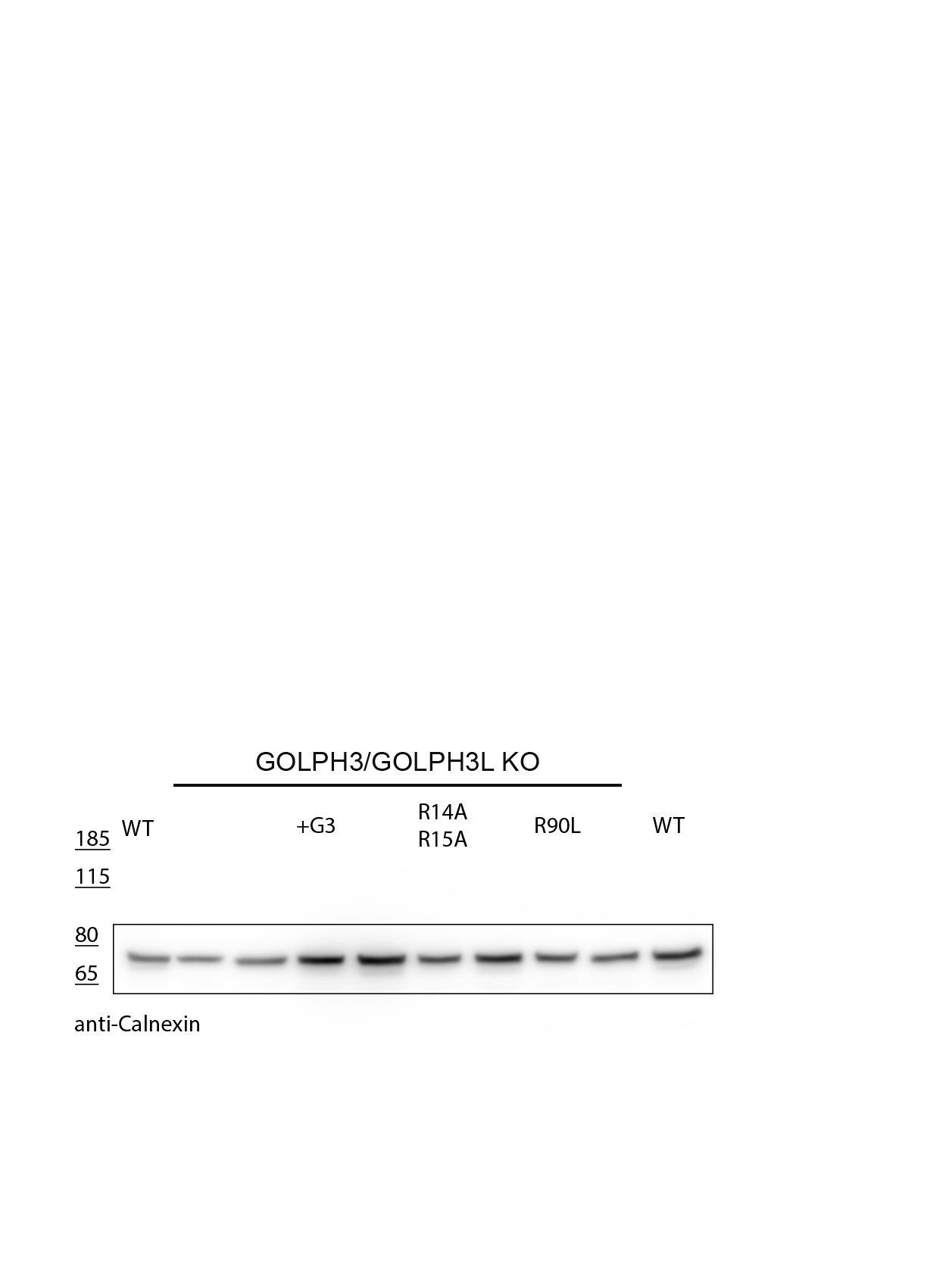

Supplement: Supplementary file 8 — Source data Fig. 5 [file 44318_2024_305_MOESM8_ESM.zip › Figure 5/5B/source data Calnexin for LYSET, HEX B.tif]

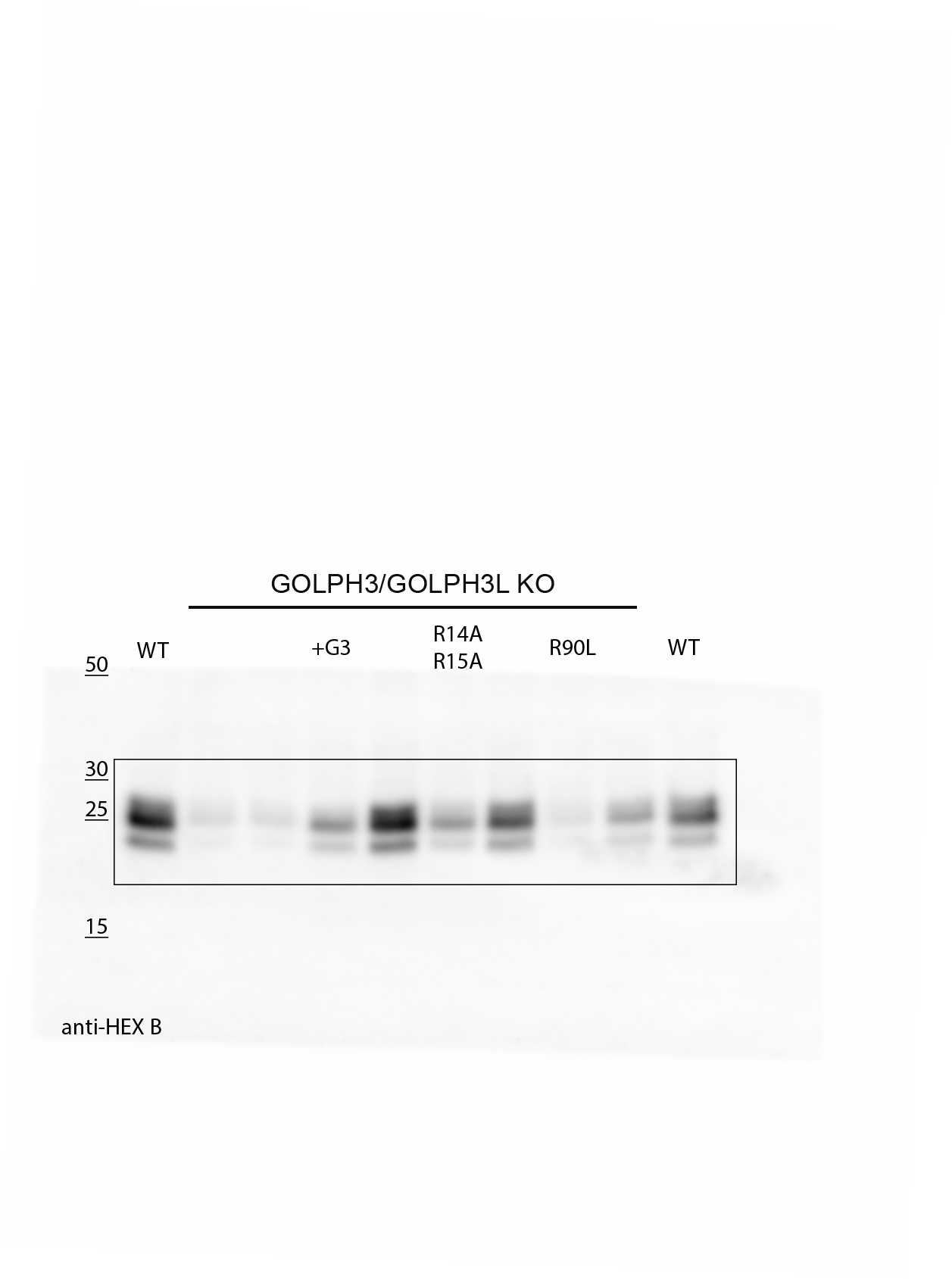

Supplement: Supplementary file 8 — Source data Fig. 5 [file 44318_2024_305_MOESM8_ESM.zip › Figure 5/5B/source data HEX B.tif]

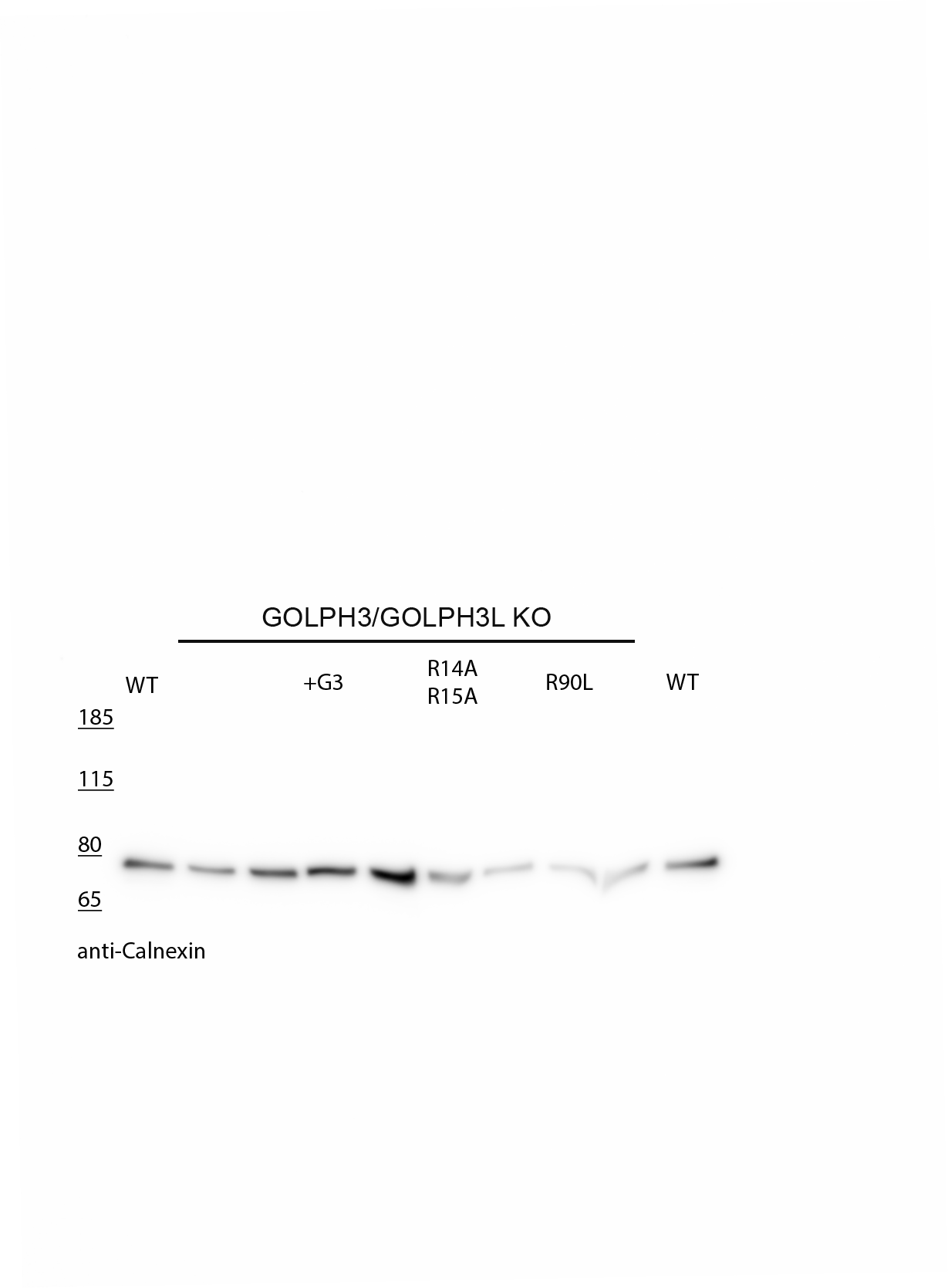

Supplement: Supplementary file 8 — Source data Fig. 5 [file 44318_2024_305_MOESM8_ESM.zip › Figure 5/5B/Calnexin for MAN1B1.tif]

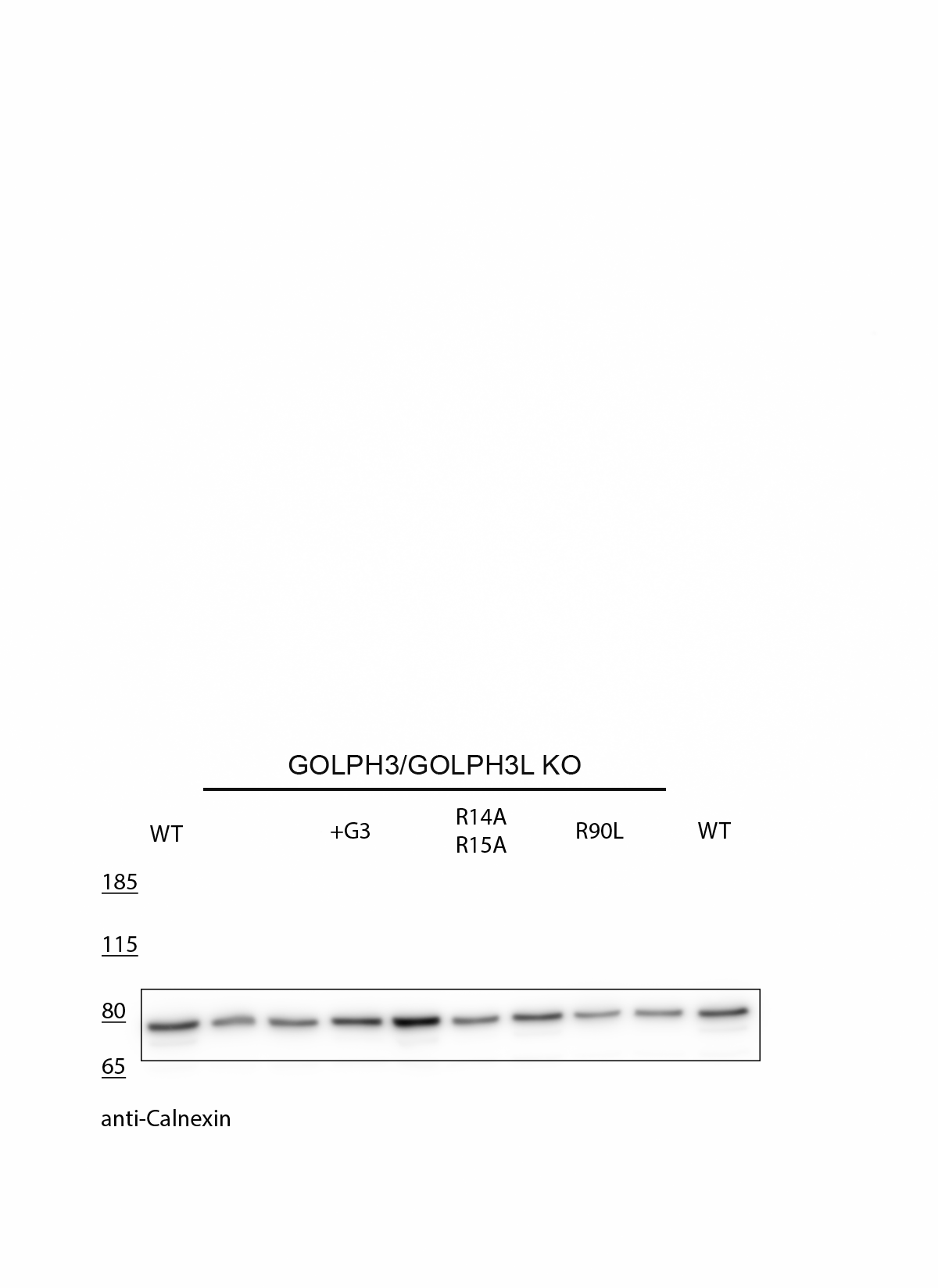

Supplement: Supplementary file 8 — Source data Fig. 5 [file 44318_2024_305_MOESM8_ESM.zip › Figure 5/5B/source data Calnexin for GOLPH3.tif]

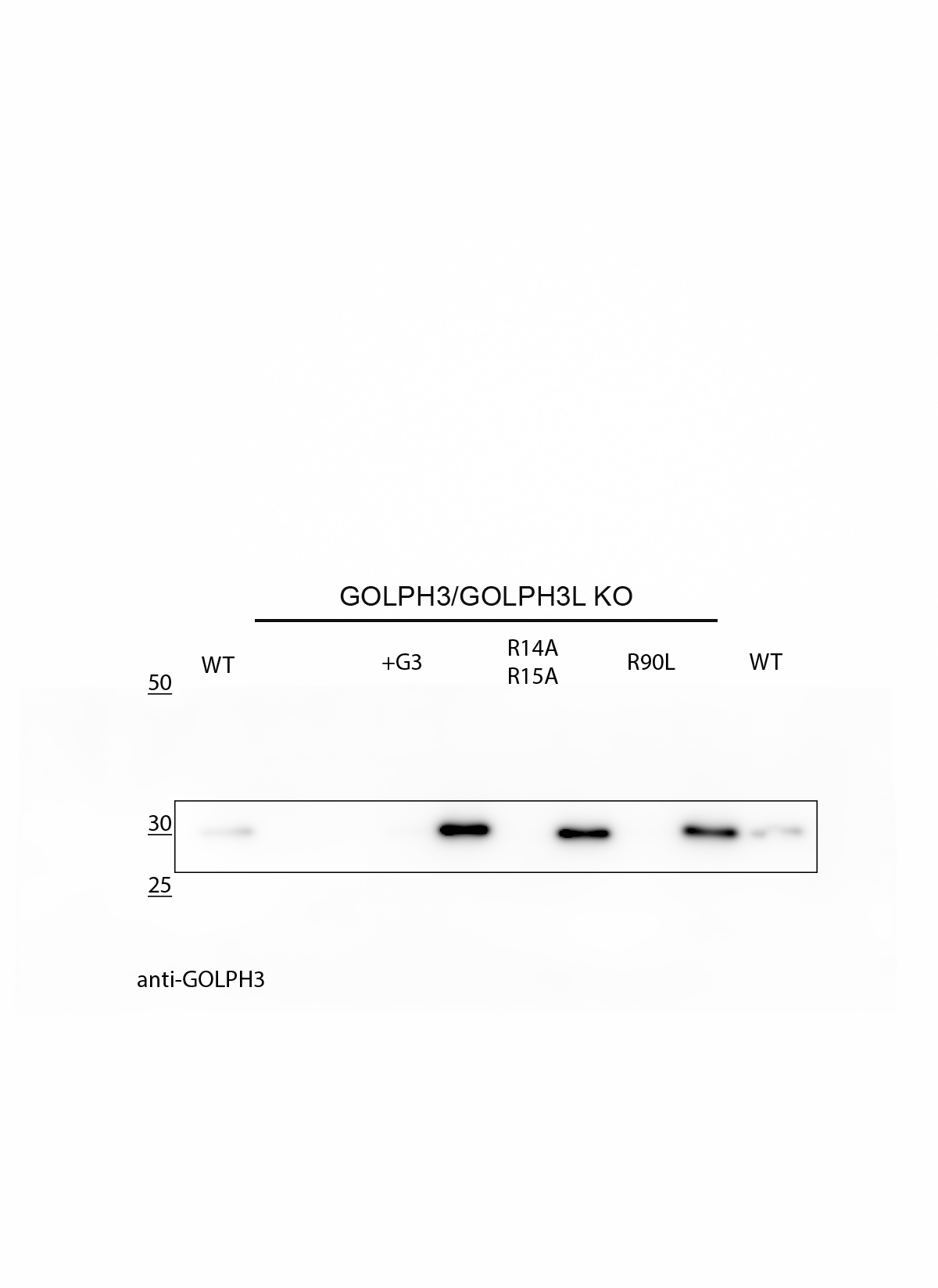

Supplement: Supplementary file 8 — Source data Fig. 5 [file 44318_2024_305_MOESM8_ESM.zip › Figure 5/5B/source data GOLPH3 short exp..tif]

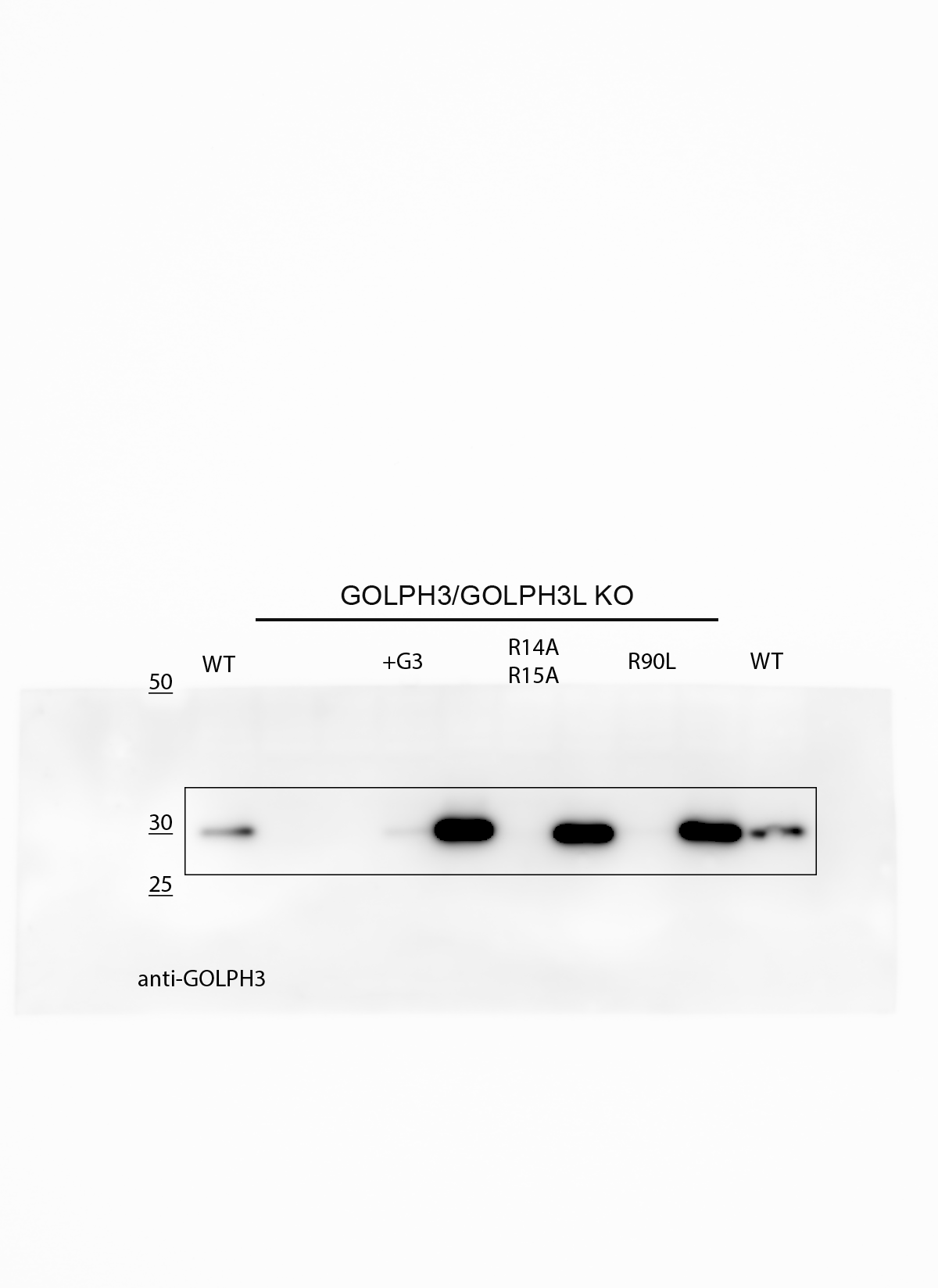

Supplement: Supplementary file 8 — Source data Fig. 5 [file 44318_2024_305_MOESM8_ESM.zip › Figure 5/5B/source data GOLPH3 long exp..tif]

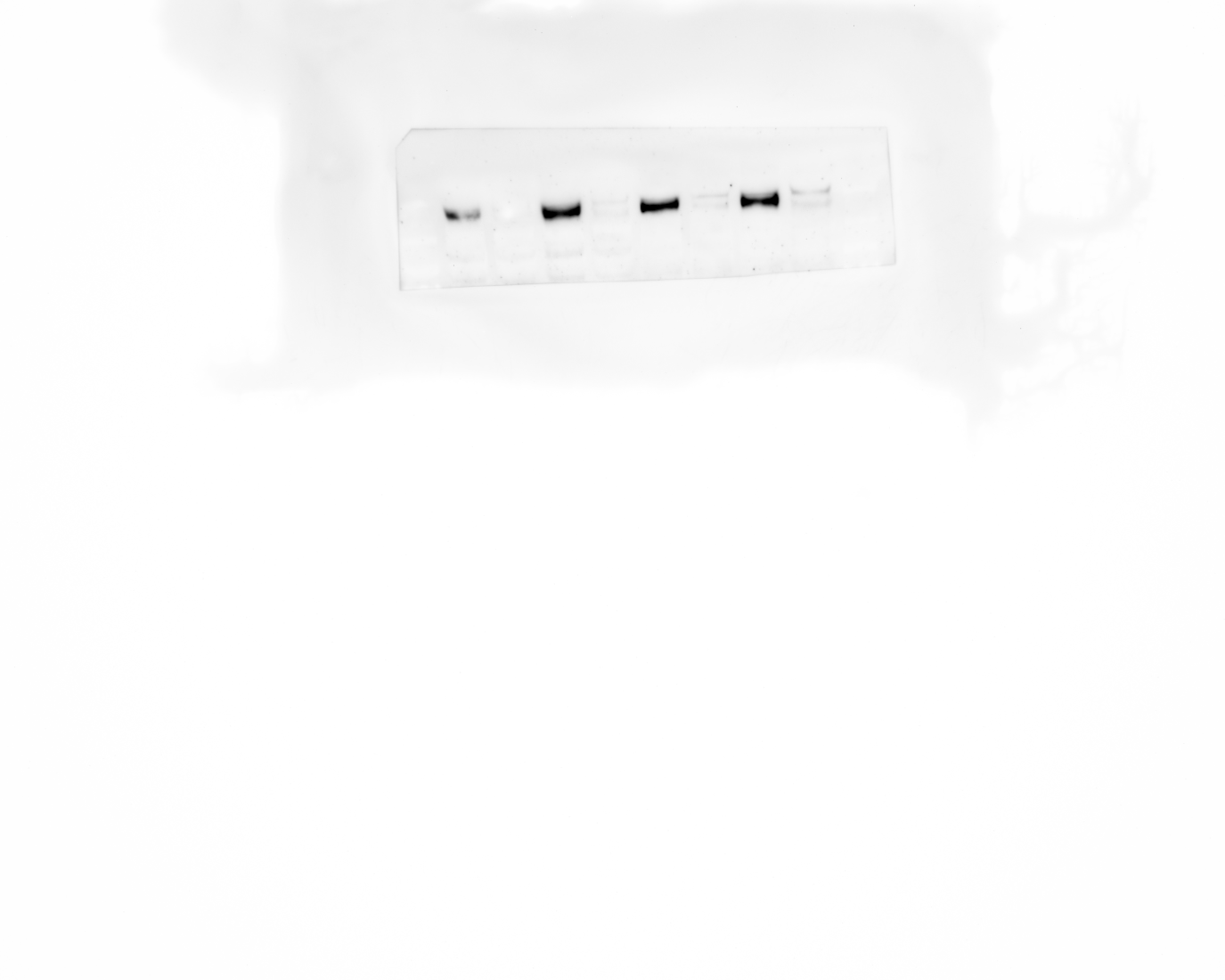

Supplement: Supplementary file 8 — Source data Fig. 5 [file 44318_2024_305_MOESM8_ESM.zip › Figure 5/Quantification 5E and 5F/5F/3) zilei2024-08-26rg95(wt g3ko wt+pi g3ko+pi(golgi)wt g3ko wt+pi g3ko+pi(lyso))hap1_1.tif]

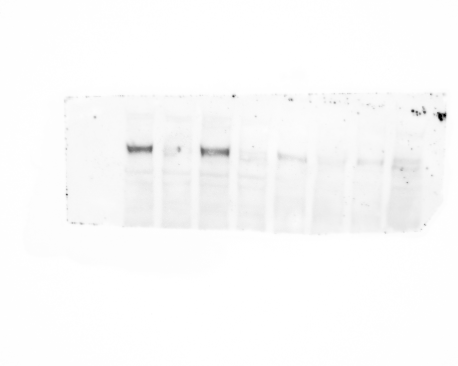

Supplement: Supplementary file 8 — Source data Fig. 5 [file 44318_2024_305_MOESM8_ESM.zip › Figure 5/Quantification 5E and 5F/5F/Sabrina 2024-02-14 16h28m01s_HAP.tif]

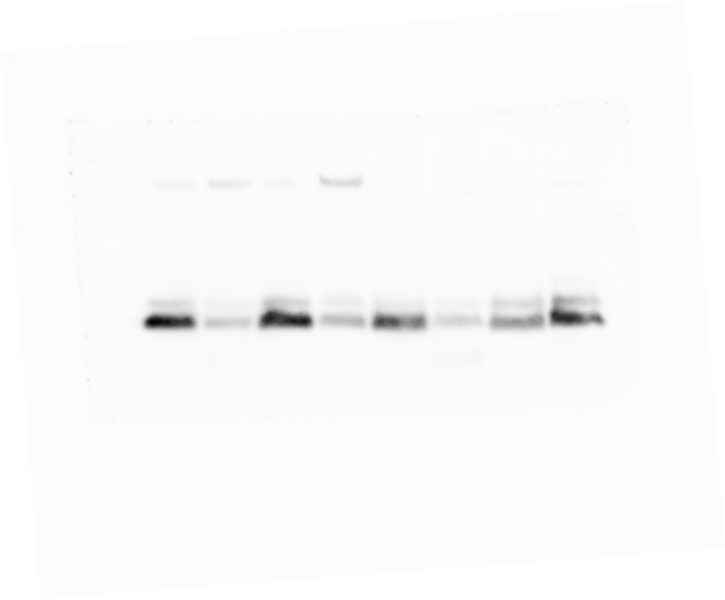

Supplement: Supplementary file 8 — Source data Fig. 5 [file 44318_2024_305_MOESM8_ESM.zip › Figure 5/Quantification 5E and 5F/5F/Sabrina 2024-02-14 13h44m46s.tif]

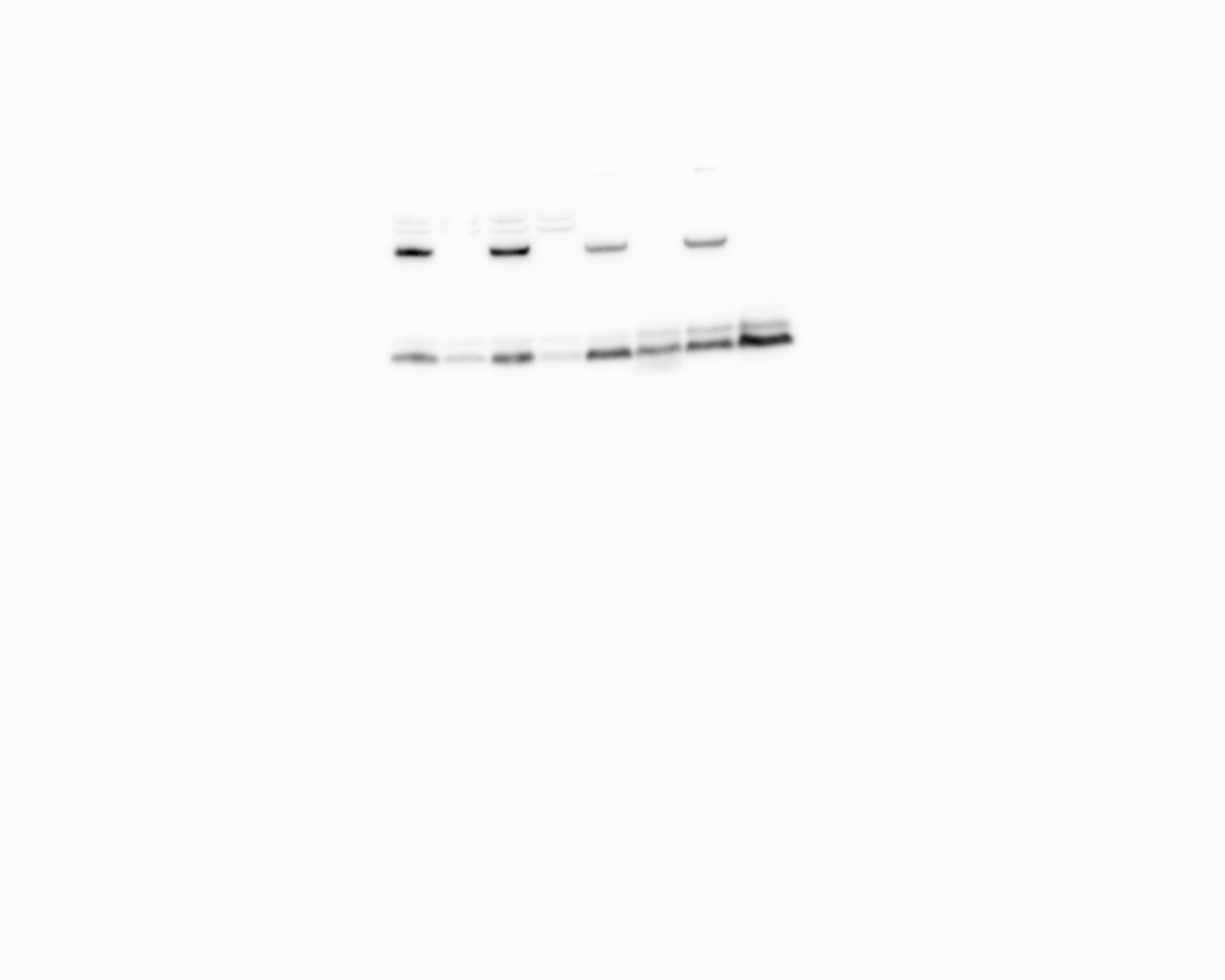

Supplement: Supplementary file 8 — Source data Fig. 5 [file 44318_2024_305_MOESM8_ESM.zip › Figure 5/Quantification 5E and 5F/5F/4) zilei2024-08-27lyset(wt g3ko wt+pi g3ko+pi(golgi)wt g3ko wt+pi g3ko+pi(lyso))hap1_1.tif]

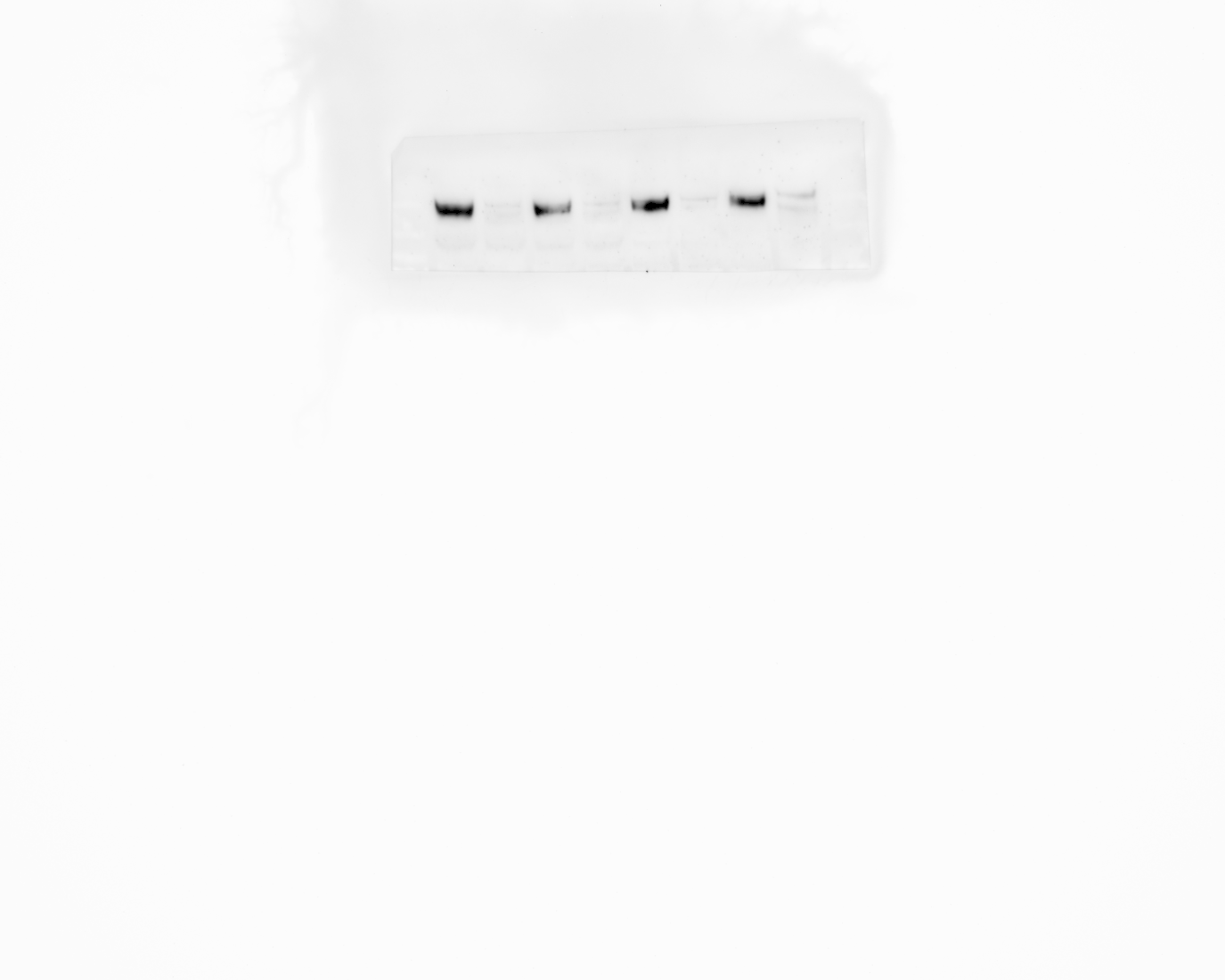

Supplement: Supplementary file 8 — Source data Fig. 5 [file 44318_2024_305_MOESM8_ESM.zip › Figure 5/Quantification 5E and 5F/5F/1) zilei2024-08-22rg95(wt g3ko wt+pi g3ko+pi(golgi)wt g3ko wt+pi g3ko+pi(lyso))hap1_1.tif]

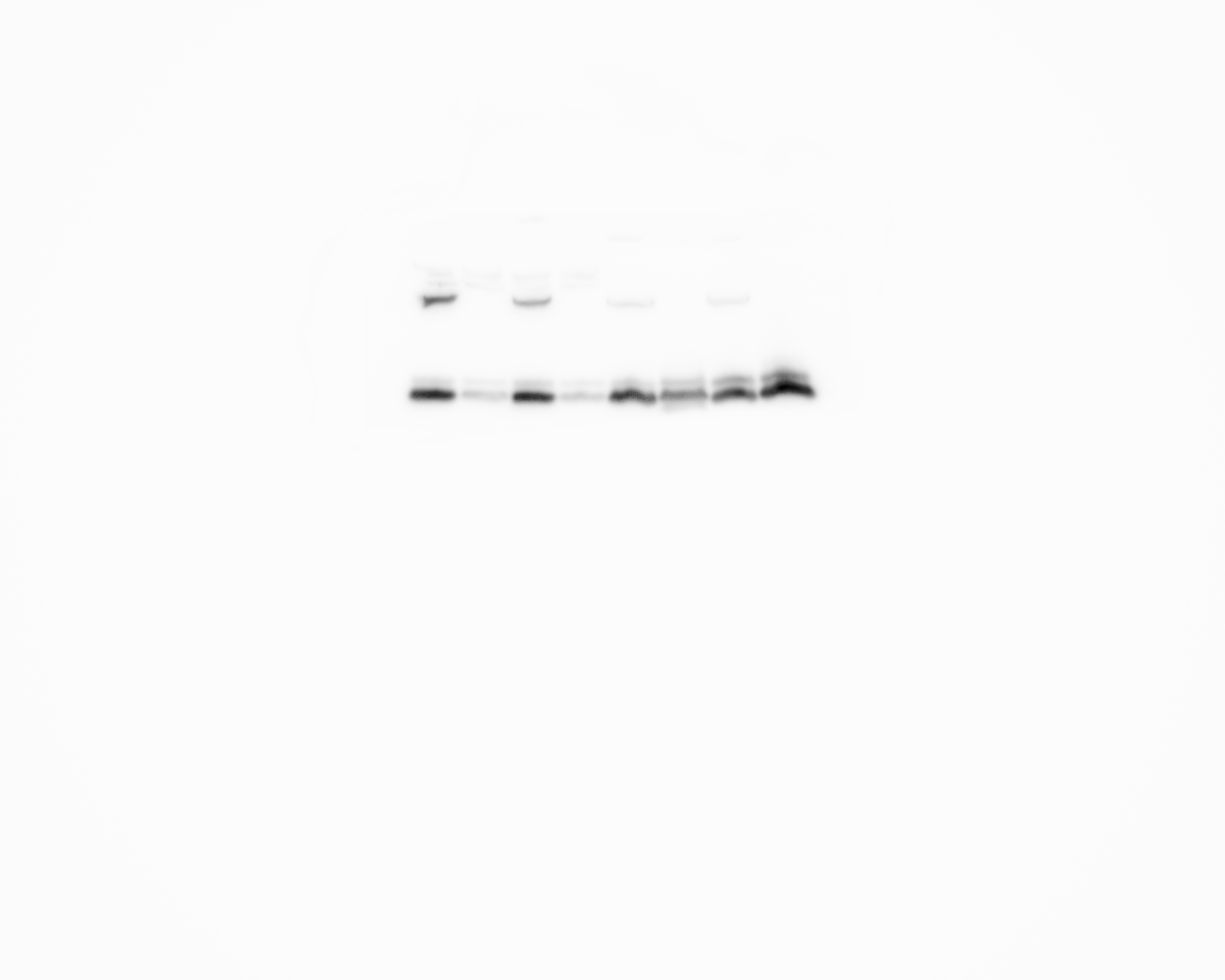

Supplement: Supplementary file 8 — Source data Fig. 5 [file 44318_2024_305_MOESM8_ESM.zip › Figure 5/Quantification 5E and 5F/5F/2) zilei2024-08-23lyset(wt g3ko wt+pi g3ko+pi(golgi)wt g3ko wt+pi g3ko+pi(lyso))hap1_1.tif]

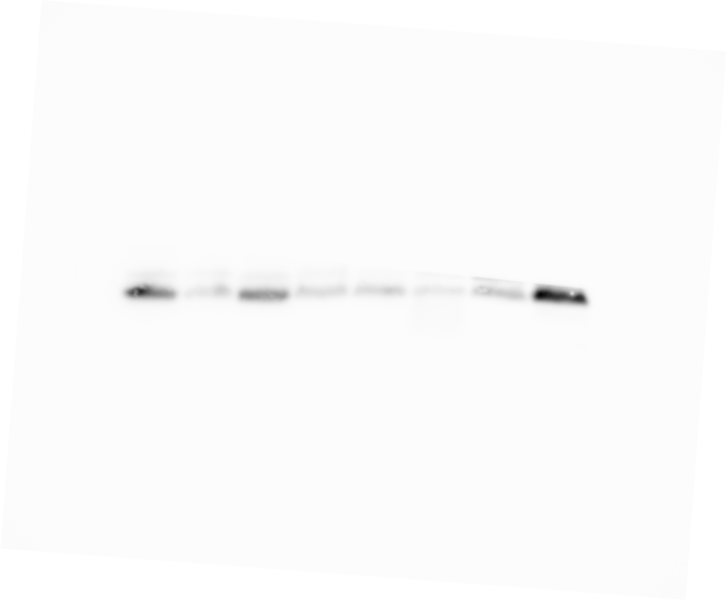

Supplement: Supplementary file 8 — Source data Fig. 5 [file 44318_2024_305_MOESM8_ESM.zip › Figure 5/Quantification 5E and 5F/5E/Sabrina 2024-02-22 15h34m50s.tif]

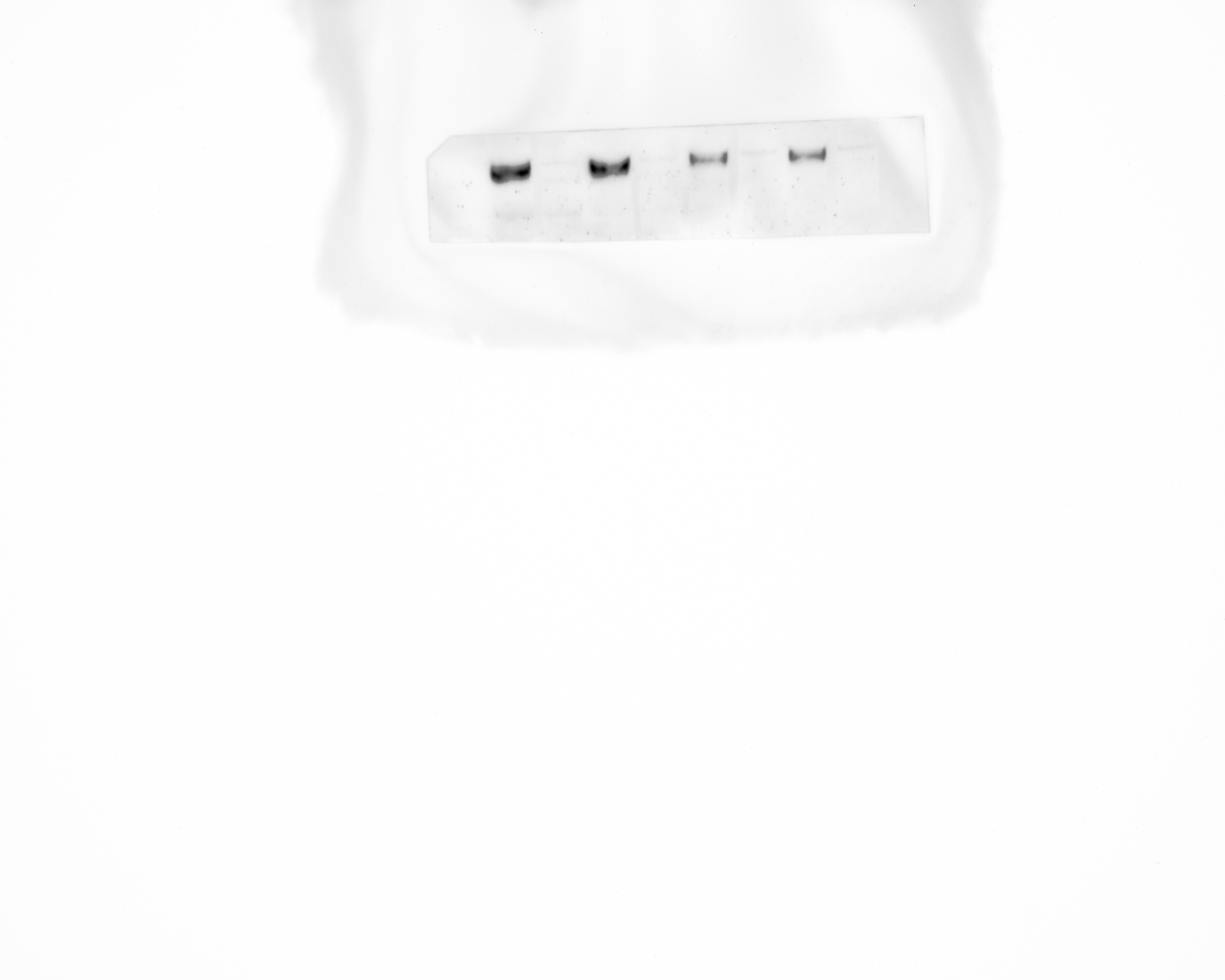

Supplement: Supplementary file 8 — Source data Fig. 5 [file 44318_2024_305_MOESM8_ESM.zip › Figure 5/Quantification 5E and 5F/5E/zilei2024-08-26rg95(wt g3ko wt+pi g3ko+pi(golgi)wt g3ko wt+pi g3ko+pi(lyso))hek_1[1].tif]

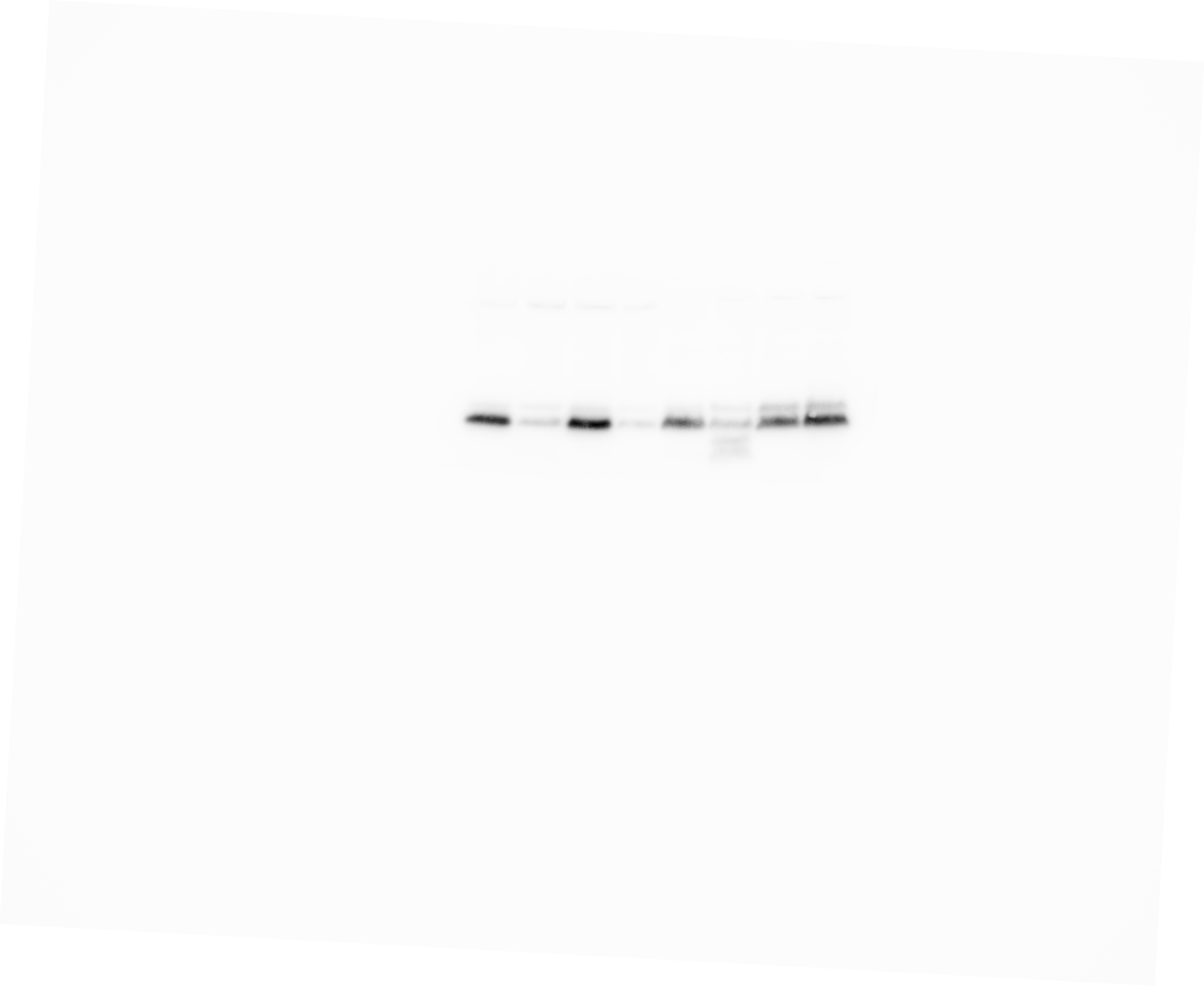

Supplement: Supplementary file 8 — Source data Fig. 5 [file 44318_2024_305_MOESM8_ESM.zip › Figure 5/Quantification 5E and 5F/5E/zilei2024-09-27Lyset(wt g3ko wt+pi g3ko+pi(golgi)wt g3ko wt+pi g3ko+pi(lyso))hek_1.tif]

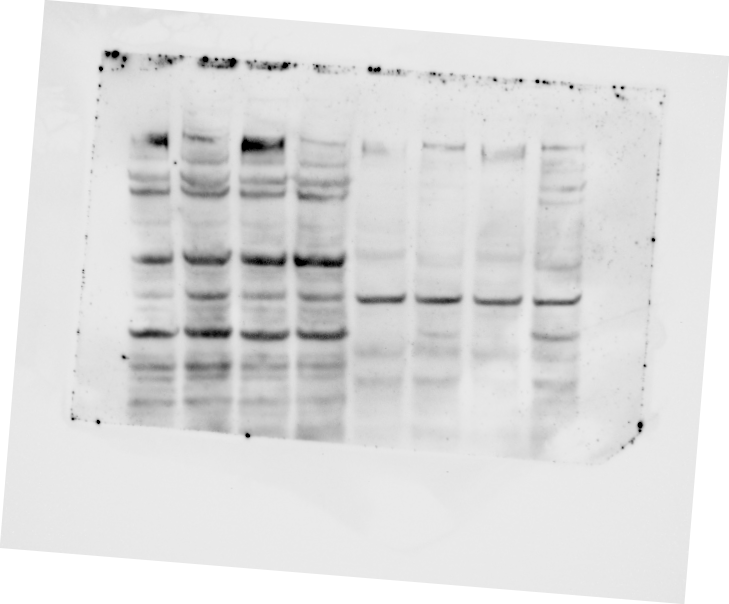

Supplement: Supplementary file 8 — Source data Fig. 5 [file 44318_2024_305_MOESM8_ESM.zip › Figure 5/Quantification 5E and 5F/5E/Sabrina 2024-02-22 15h39m10s.tif]

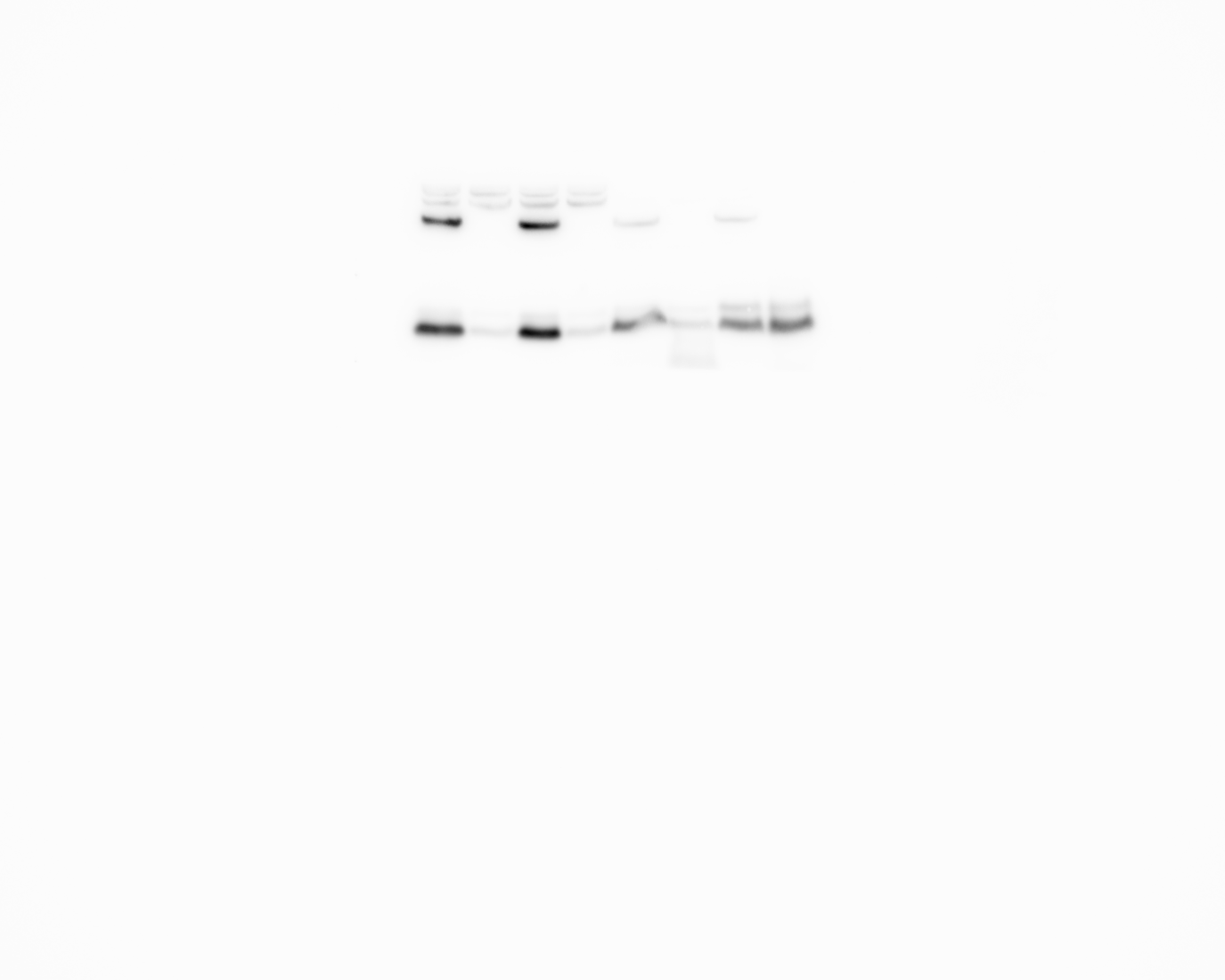

Supplement: Supplementary file 8 — Source data Fig. 5 [file 44318_2024_305_MOESM8_ESM.zip › Figure 5/Quantification 5E and 5F/5E/zilei2024-08-27lyset(wt g3ko wt+pi g3ko+pi(golgi)wt g3ko wt+pi g3ko+pi(lyso))hek_1.tif]

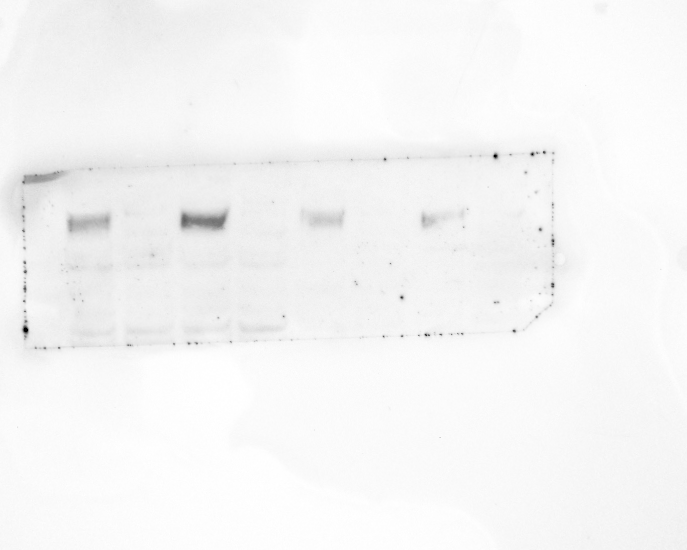

Supplement: Supplementary file 8 — Source data Fig. 5 [file 44318_2024_305_MOESM8_ESM.zip › Figure 5/Quantification 5E and 5F/5E/Sabrina 2024-09-28 15h50m20s.tif]

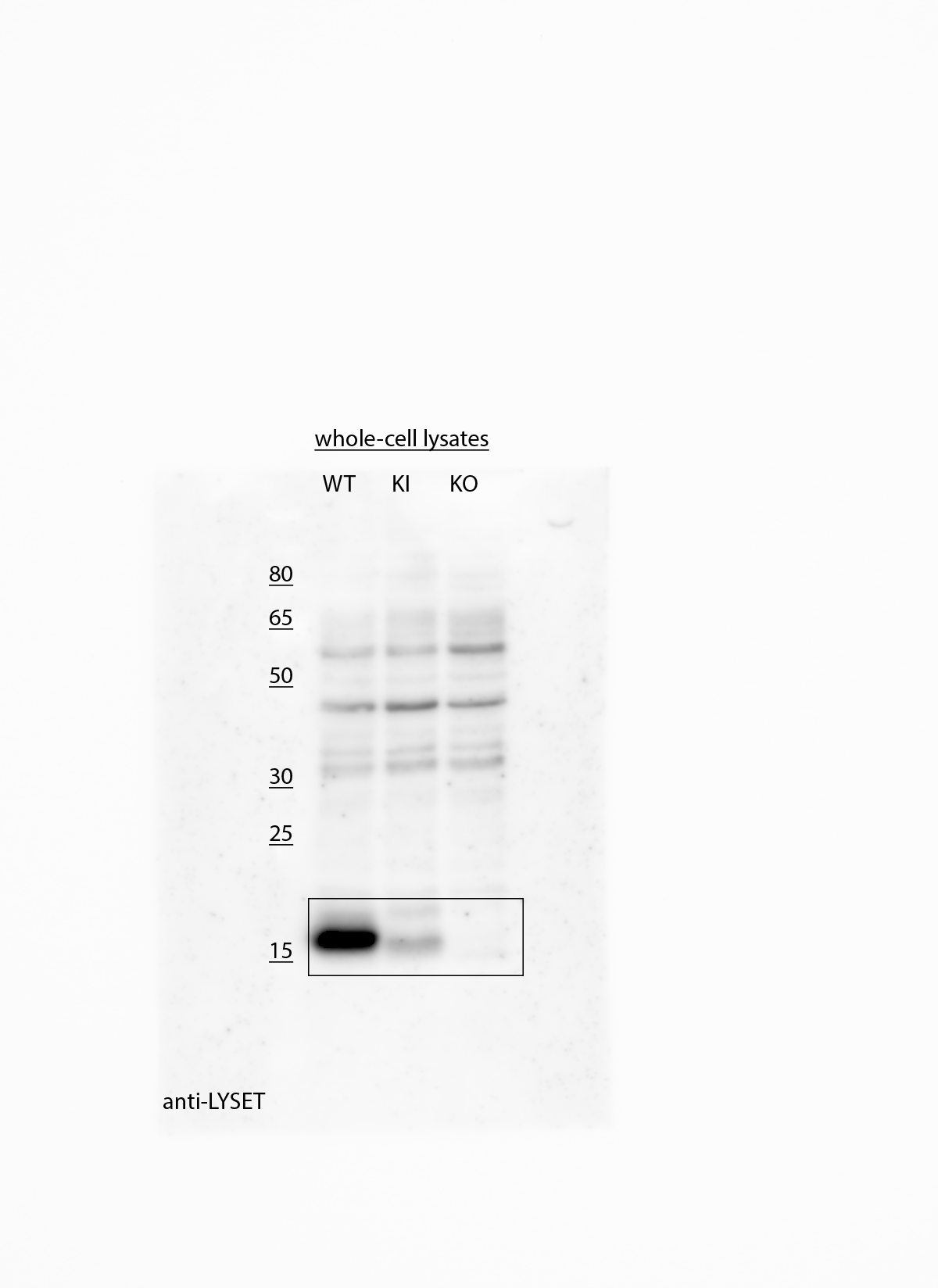

Supplement: Supplementary file 9 — Source data Fig. 6 [file 44318_2024_305_MOESM9_ESM.zip › Figure 6/6F/source data LYSET long exp..tif]

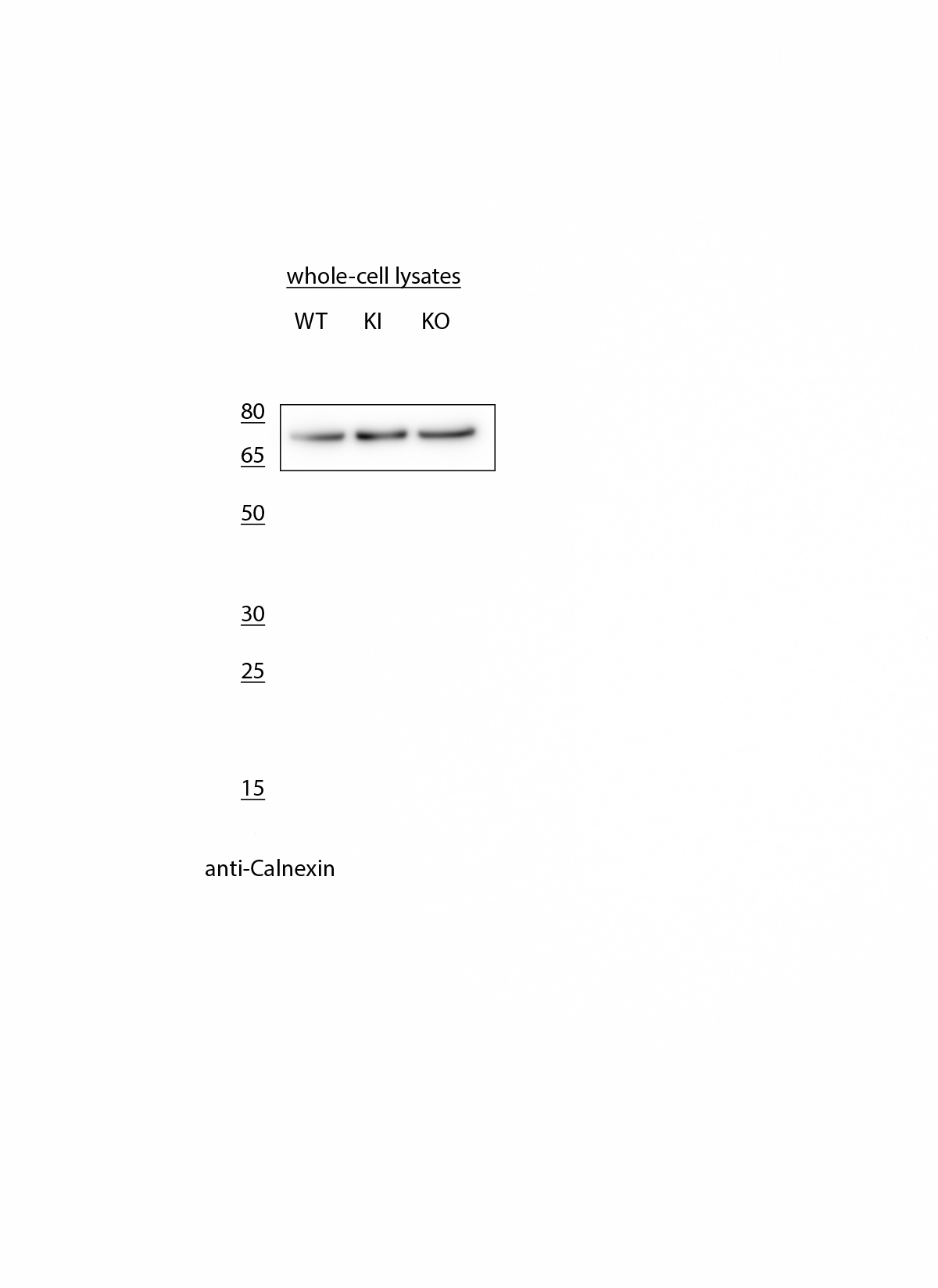

Supplement: Supplementary file 9 — Source data Fig. 6 [file 44318_2024_305_MOESM9_ESM.zip › Figure 6/6F/source data Calnexin for LYSET.tif]

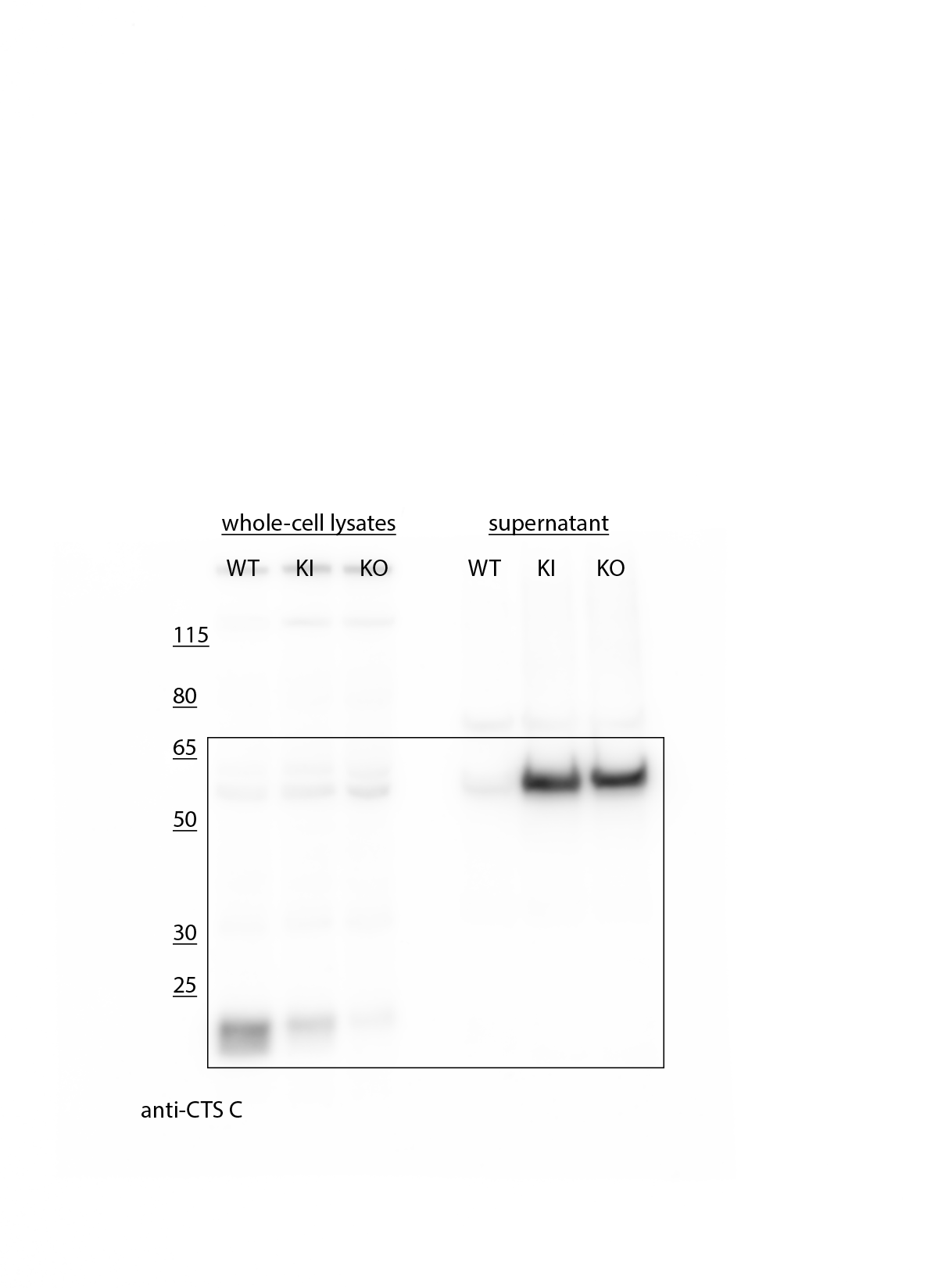

Supplement: Supplementary file 9 — Source data Fig. 6 [file 44318_2024_305_MOESM9_ESM.zip › Figure 6/6F/source data CTS C.tif]

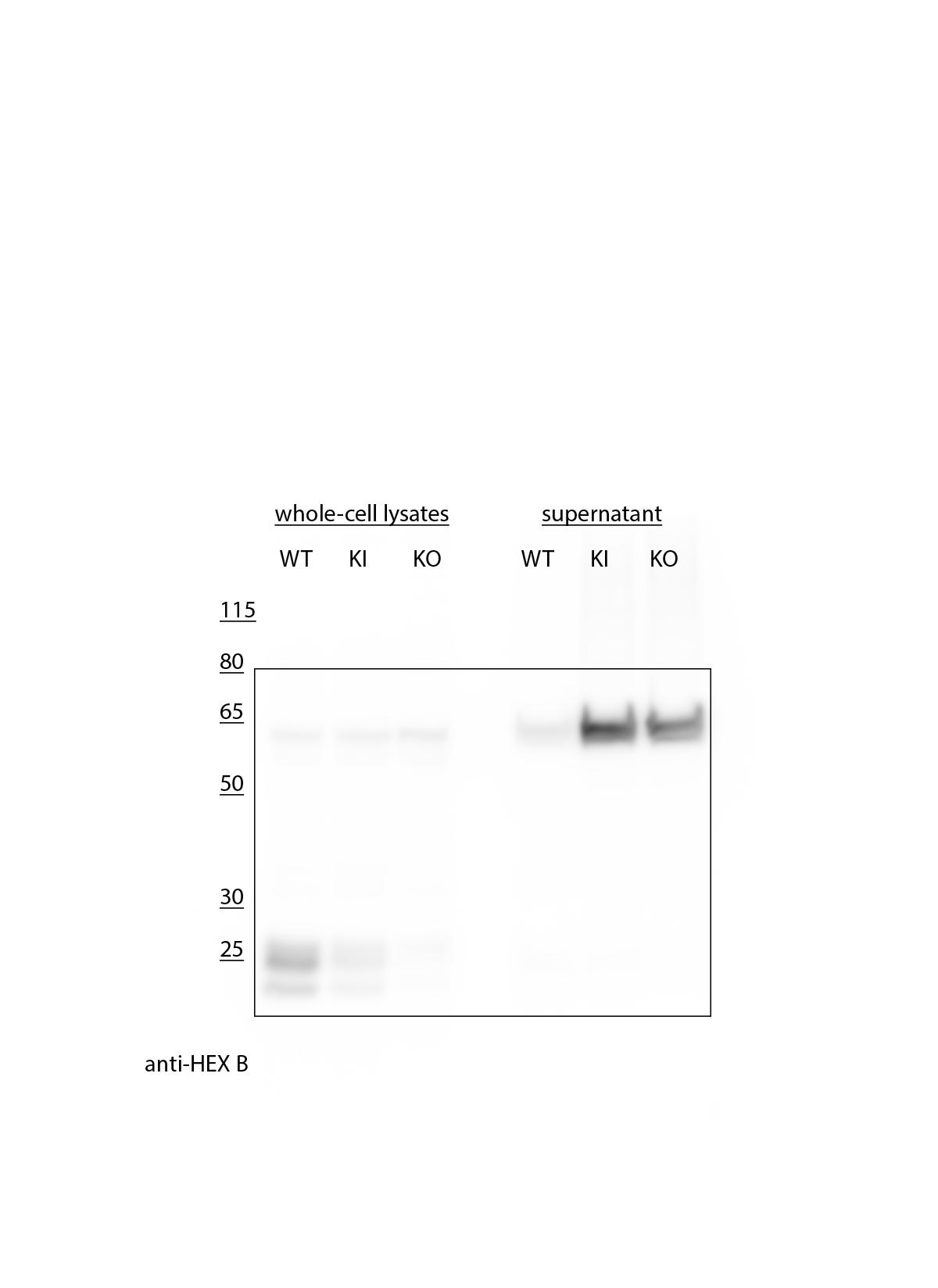

Supplement: Supplementary file 9 — Source data Fig. 6 [file 44318_2024_305_MOESM9_ESM.zip › Figure 6/6F/source data HEX B.tif]

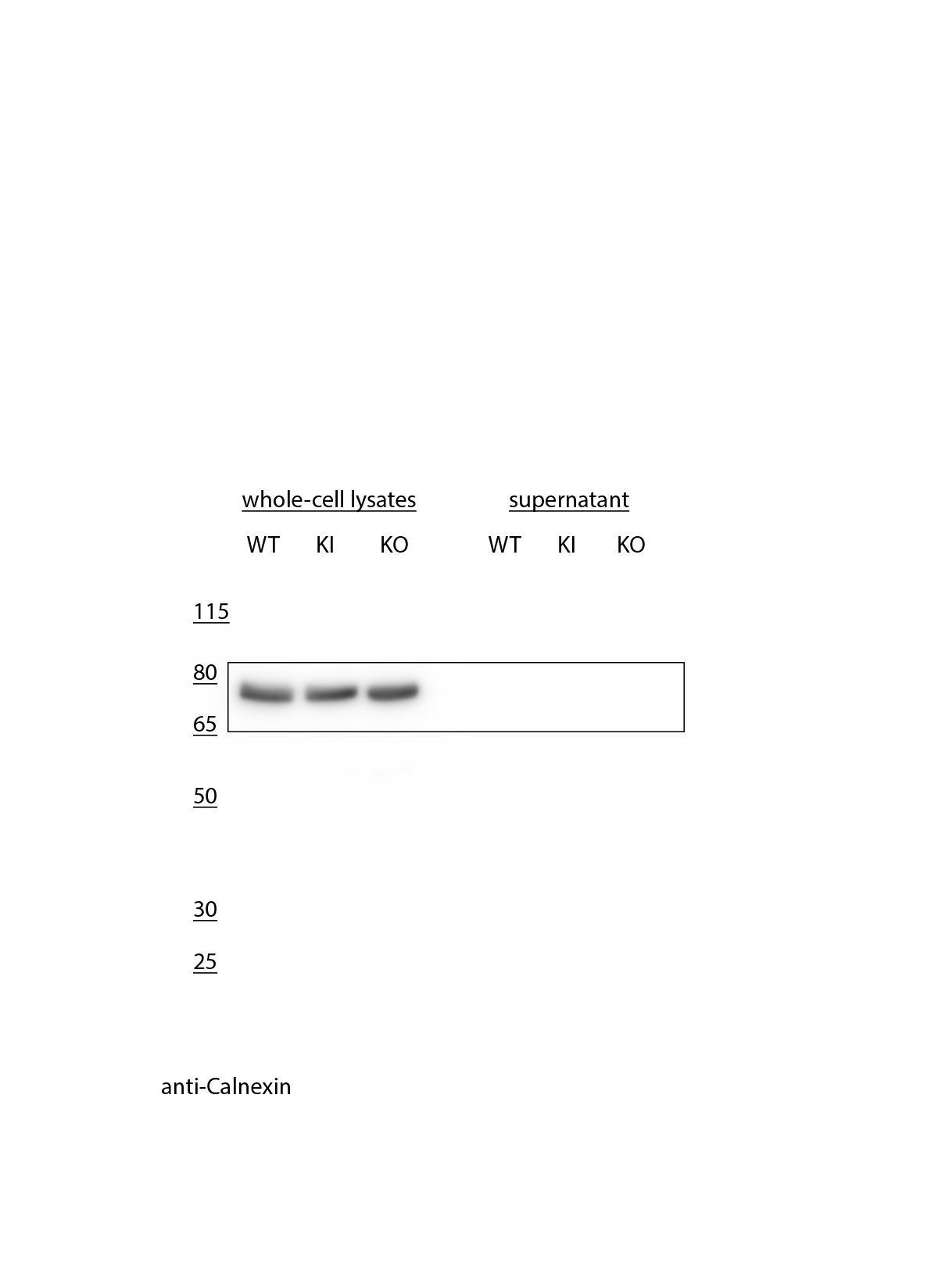

Supplement: Supplementary file 9 — Source data Fig. 6 [file 44318_2024_305_MOESM9_ESM.zip › Figure 6/6F/source data calnexin for CTS C.tif]

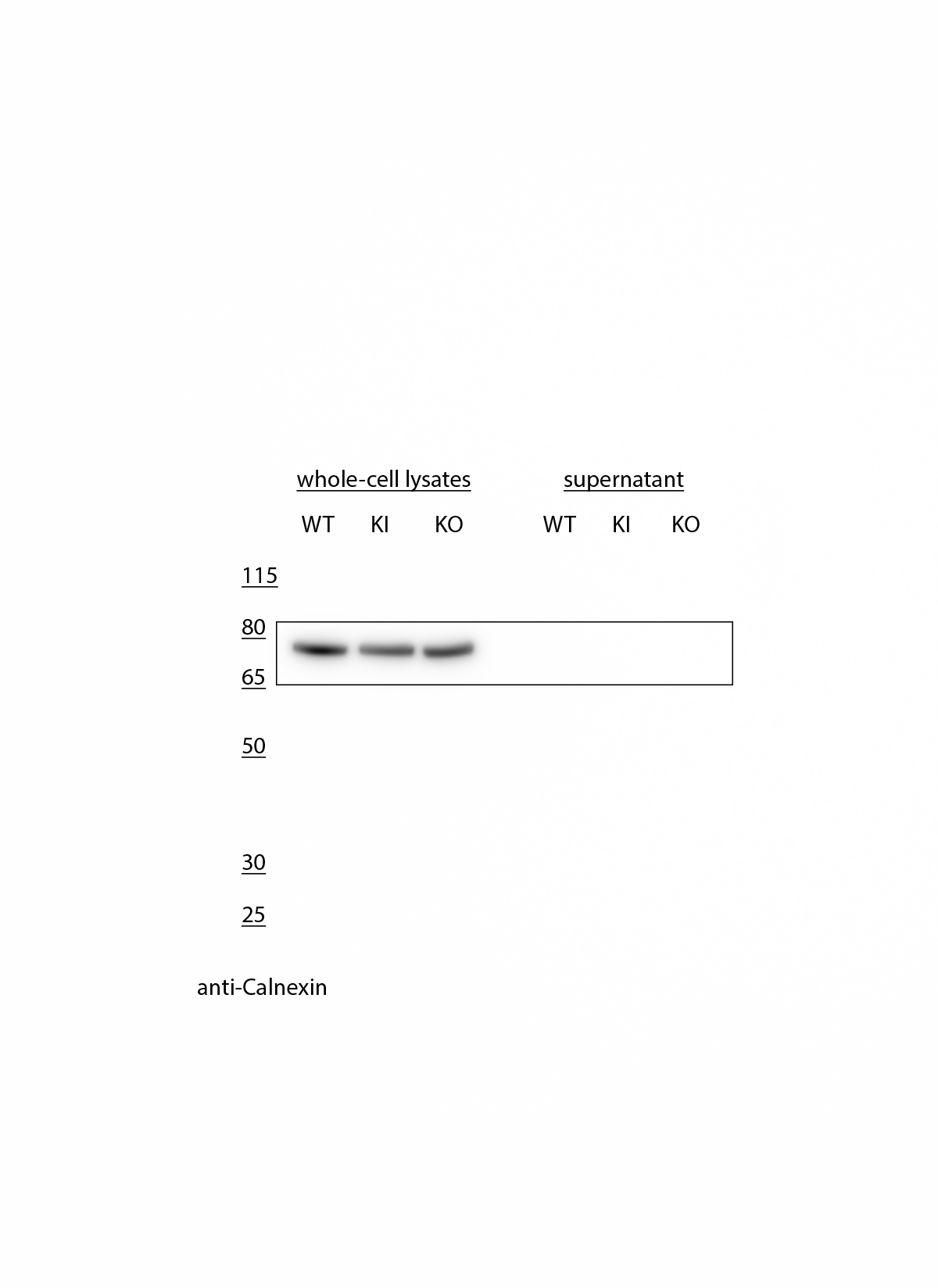

Supplement: Supplementary file 9 — Source data Fig. 6 [file 44318_2024_305_MOESM9_ESM.zip › Figure 6/6F/source data Calnexin for HEX B.tif]

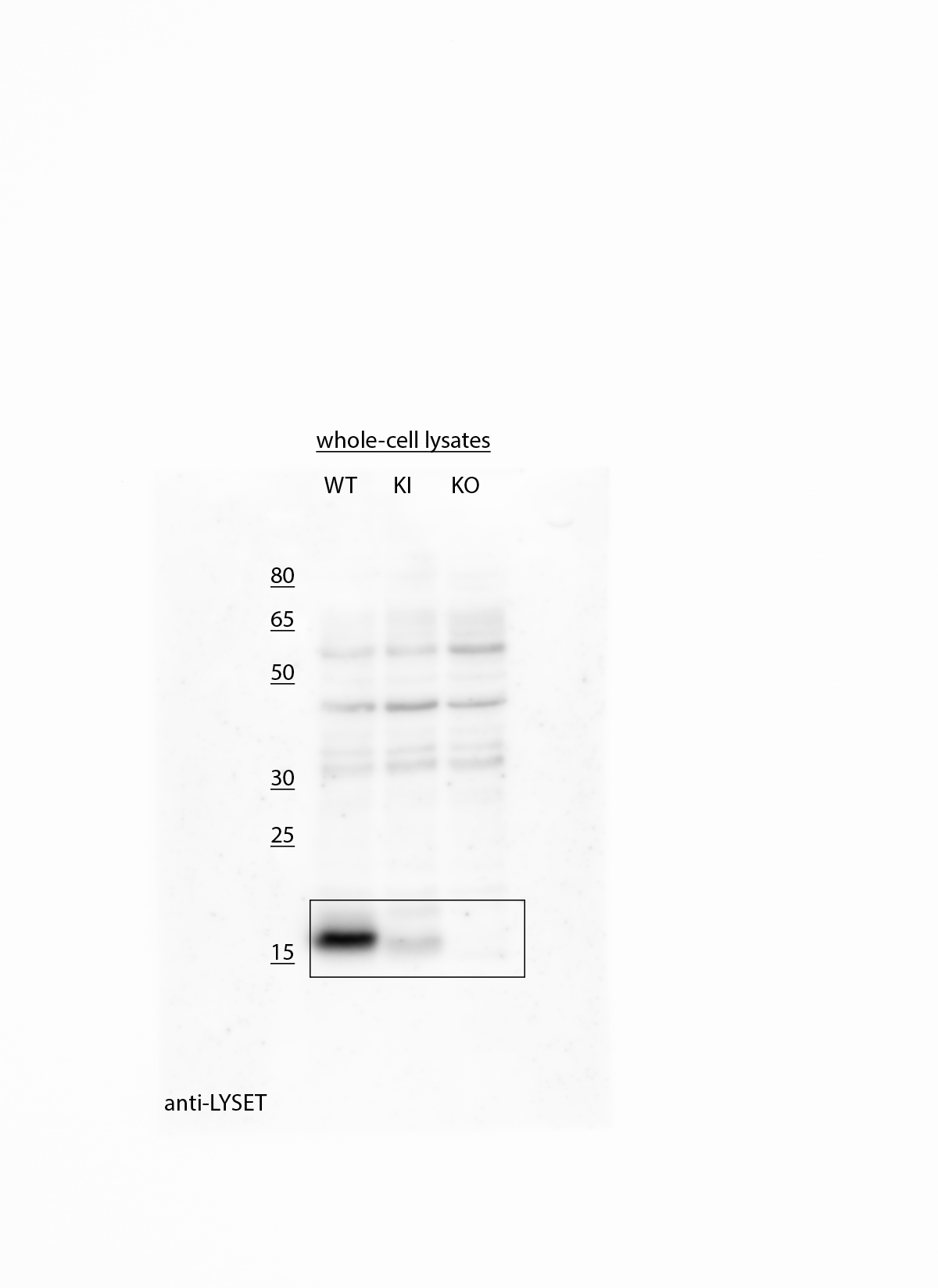

Supplement: Supplementary file 9 — Source data Fig. 6 [file 44318_2024_305_MOESM9_ESM.zip › Figure 6/6F/source data LYSET short exp..tif]

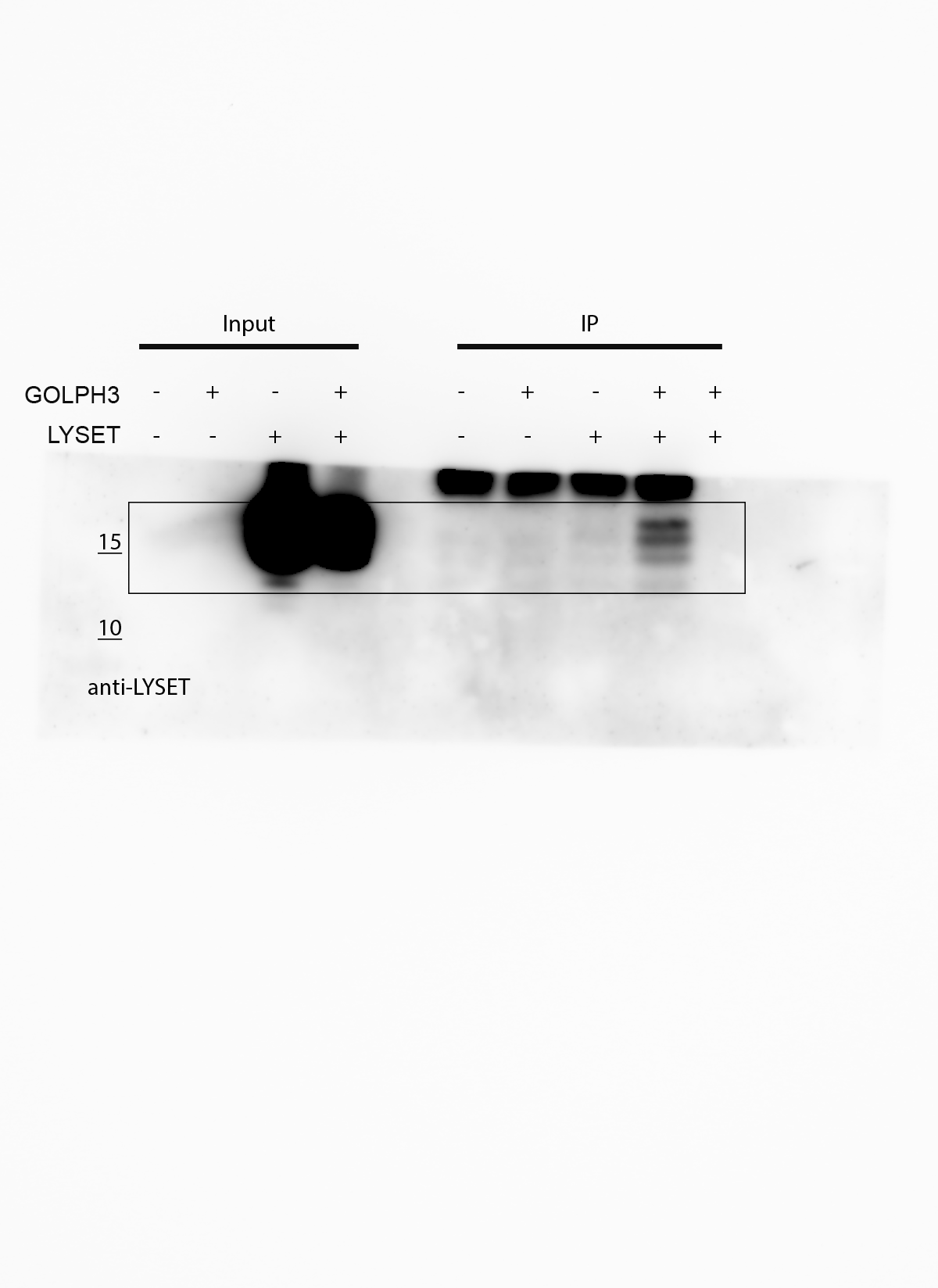

Supplement: Supplementary file 9 — Source data Fig. 6 [file 44318_2024_305_MOESM9_ESM.zip › Figure 6/6A/source data LYSET long exp..tif]

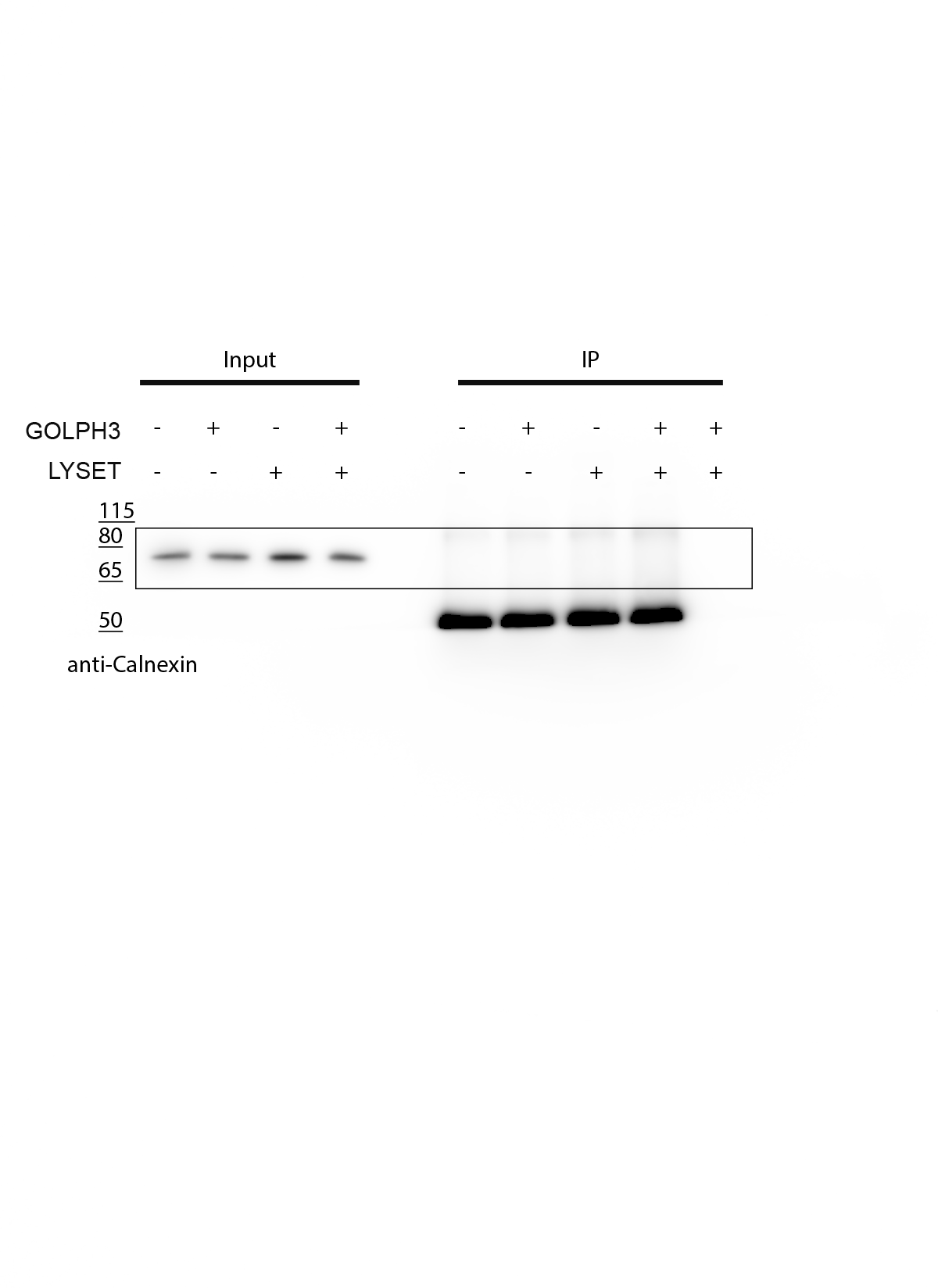

Supplement: Supplementary file 9 — Source data Fig. 6 [file 44318_2024_305_MOESM9_ESM.zip › Figure 6/6A/source data Calnexin.tif]

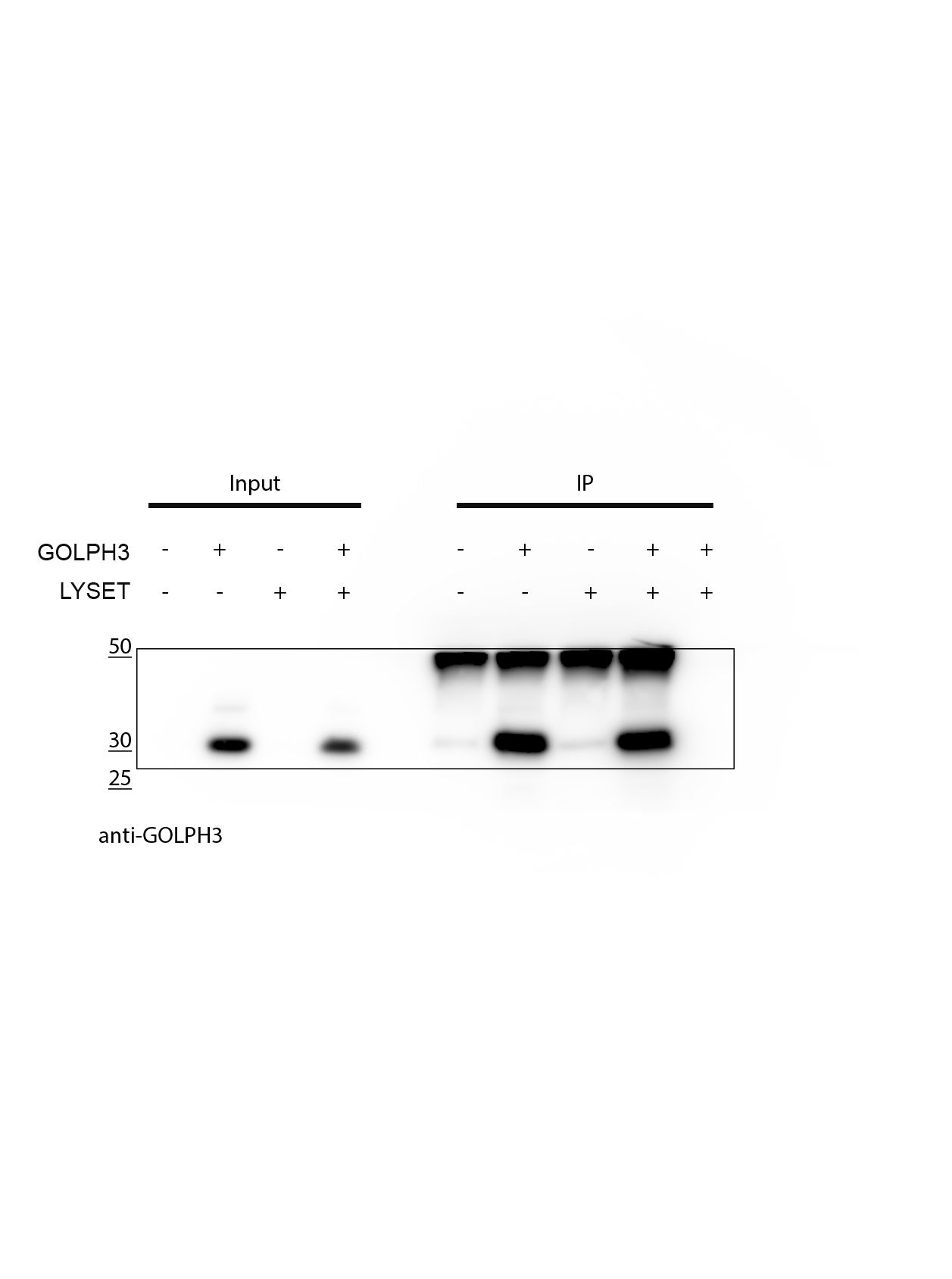

Supplement: Supplementary file 9 — Source data Fig. 6 [file 44318_2024_305_MOESM9_ESM.zip › Figure 6/6A/source data GOLPH3.tif]

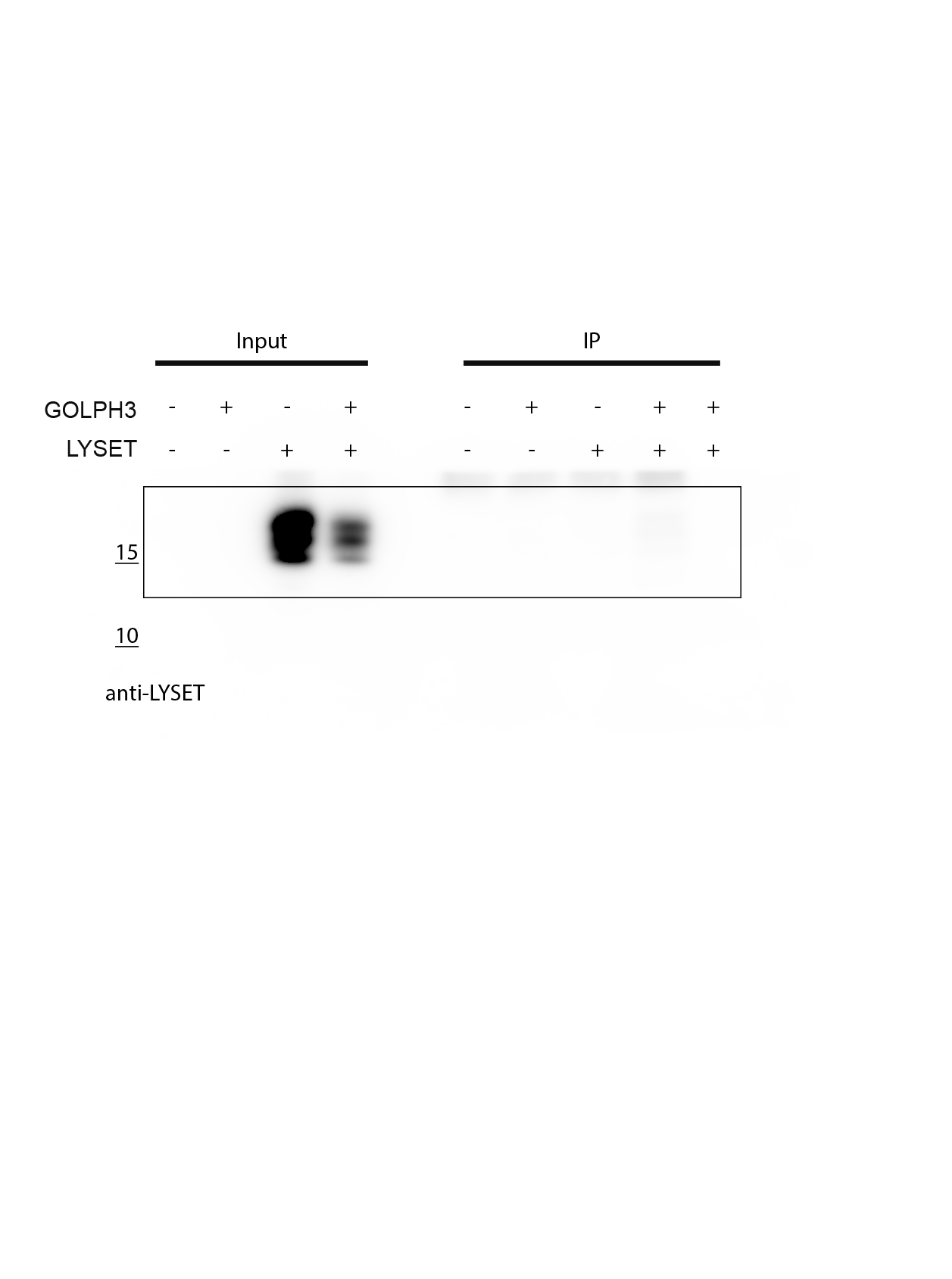

Supplement: Supplementary file 9 — Source data Fig. 6 [file 44318_2024_305_MOESM9_ESM.zip › Figure 6/6A/source data LYSET short exp..tif]

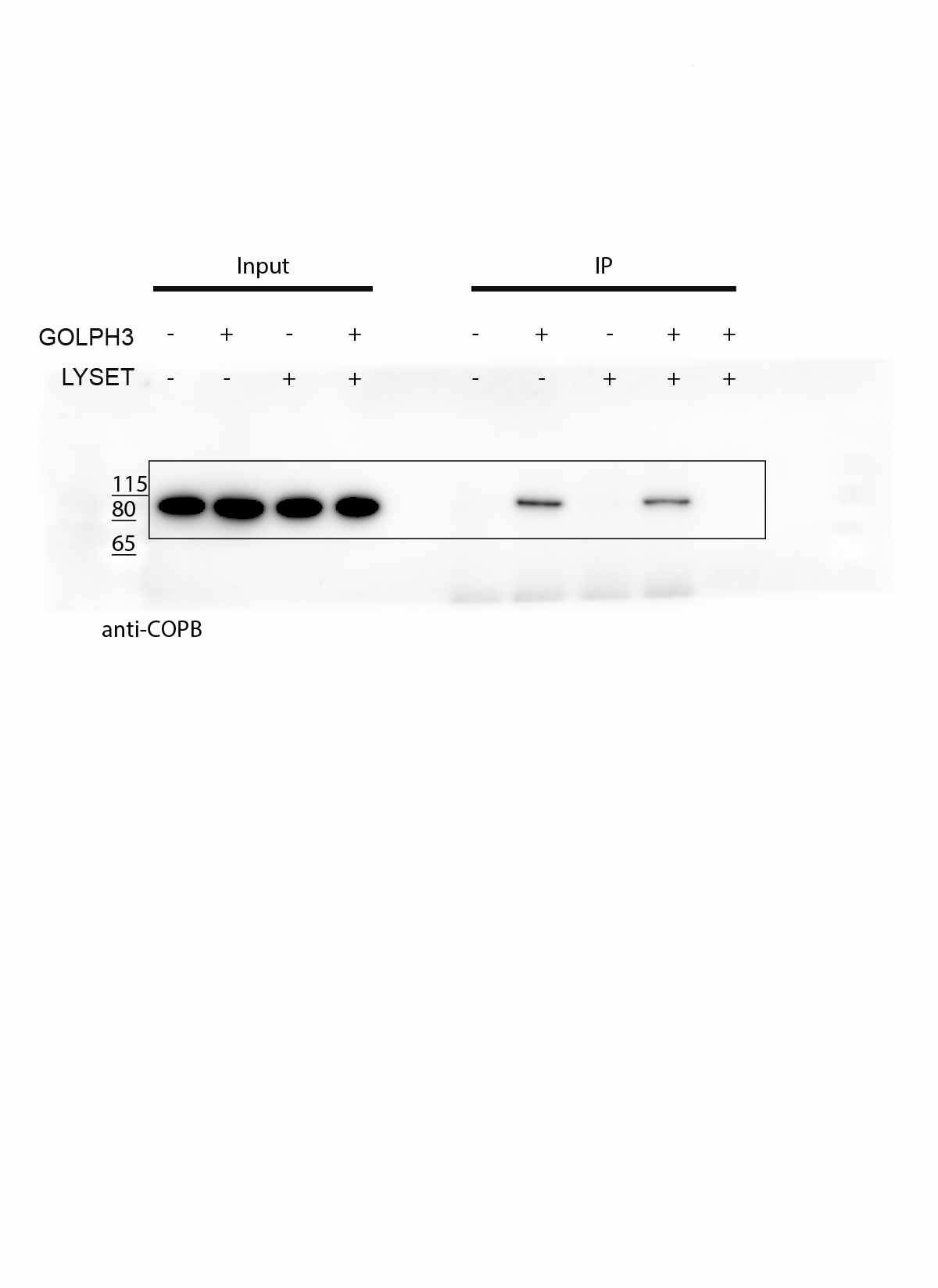

Supplement: Supplementary file 9 — Source data Fig. 6 [file 44318_2024_305_MOESM9_ESM.zip › Figure 6/6A/source data COPB.tif]

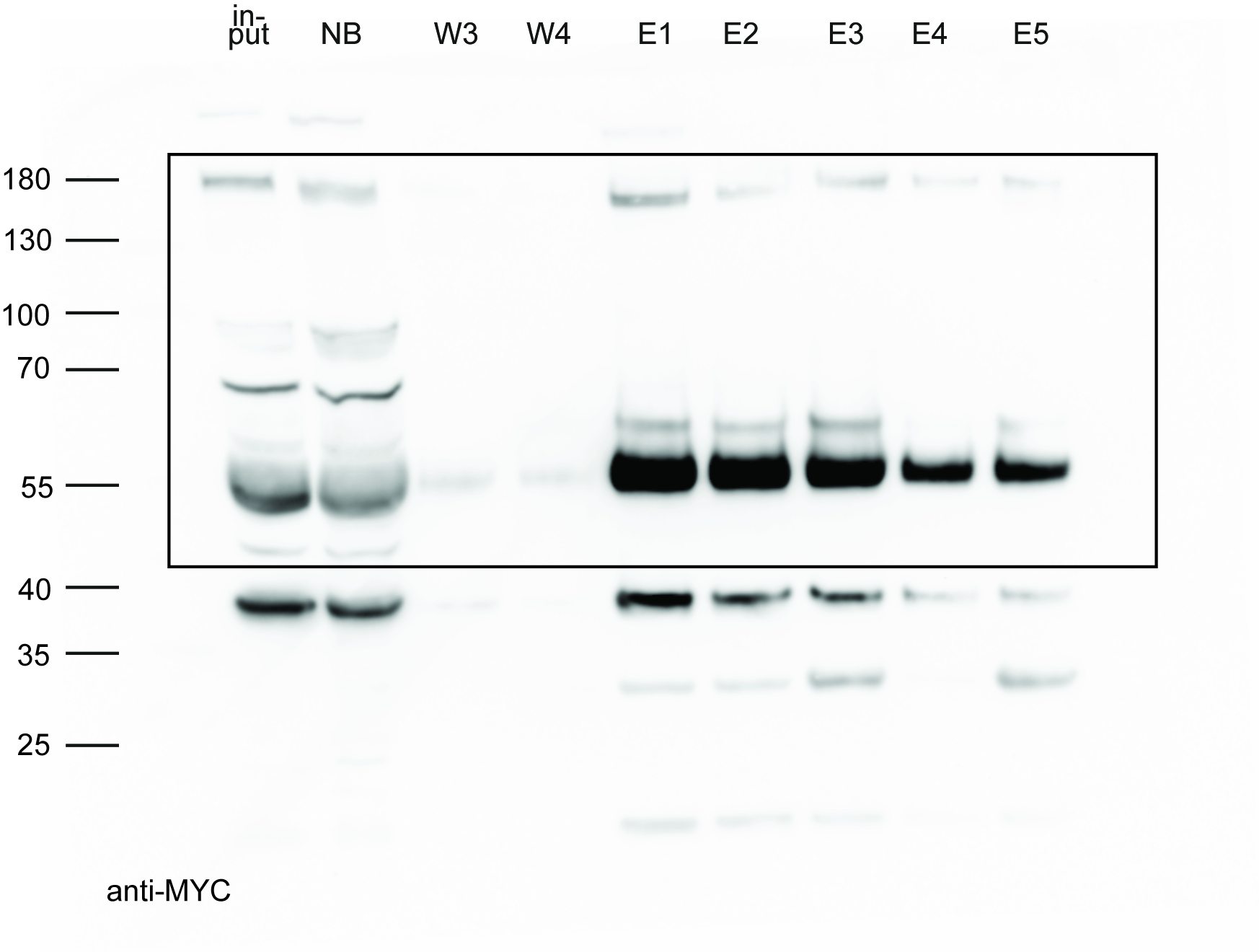

Supplement: Supplementary file 9 — Source data Fig. 6 [file 44318_2024_305_MOESM9_ESM.zip › Figure 6/6B/myc.tif]

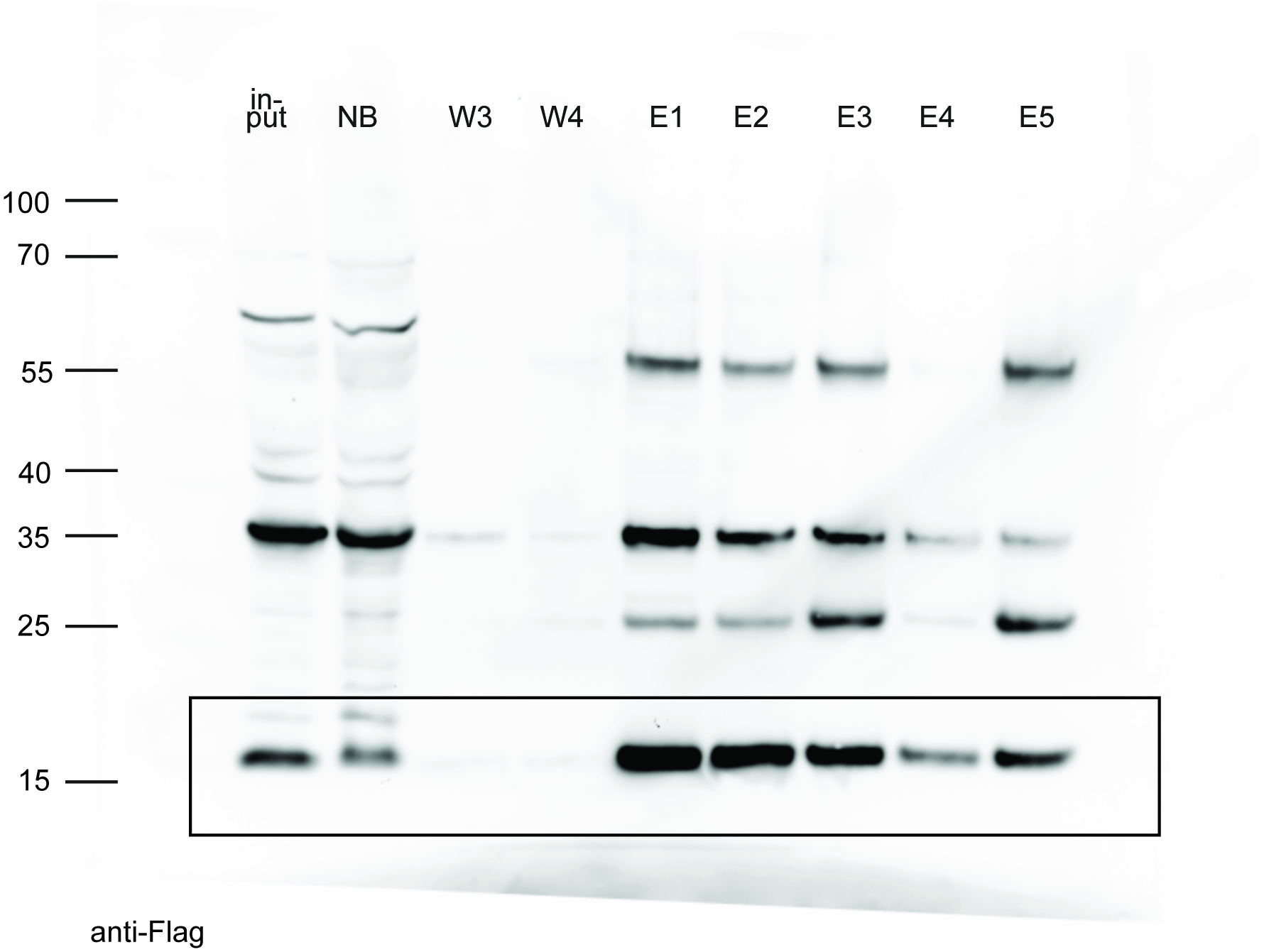

Supplement: Supplementary file 9 — Source data Fig. 6 [file 44318_2024_305_MOESM9_ESM.zip › Figure 6/6B/Flag.tif]

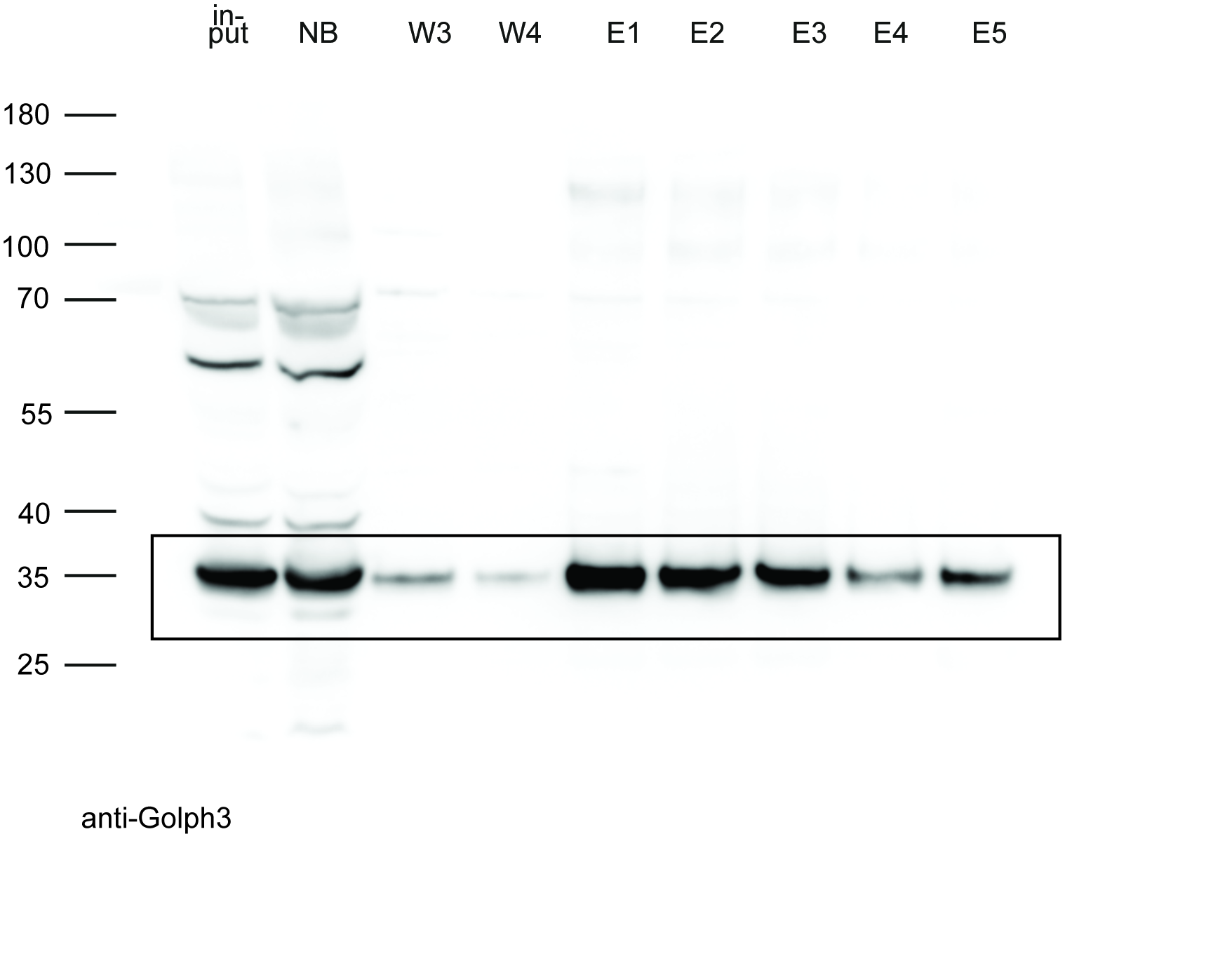

Supplement: Supplementary file 9 — Source data Fig. 6 [file 44318_2024_305_MOESM9_ESM.zip › Figure 6/6B/Golph3.tif]

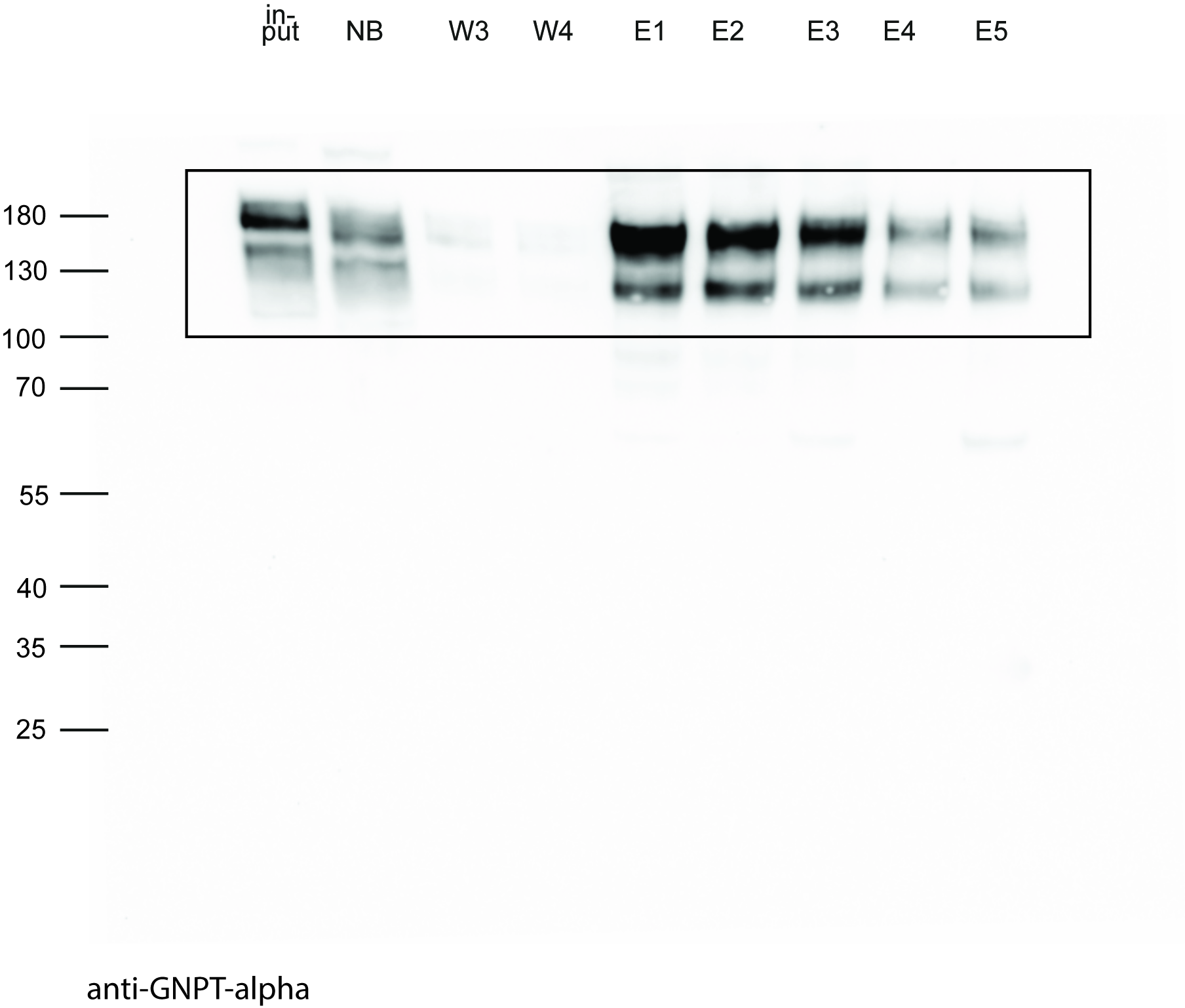

Supplement: Supplementary file 9 — Source data Fig. 6 [file 44318_2024_305_MOESM9_ESM.zip › Figure 6/6B/GNPT-alpha.tif]

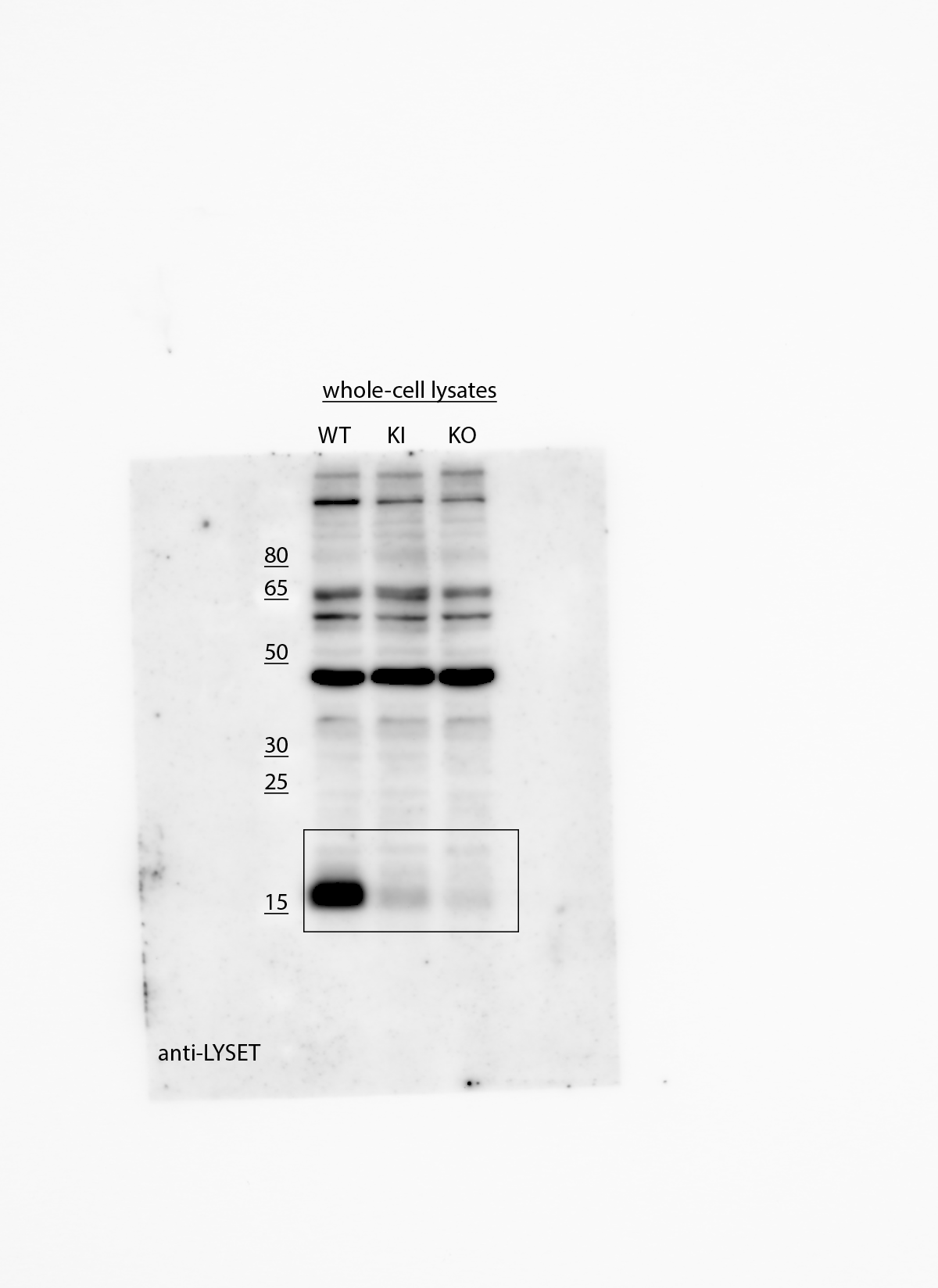

Supplement: Supplementary file 9 — Source data Fig. 6 [file 44318_2024_305_MOESM9_ESM.zip › Figure 6/6E/source data LYSET long exp..tif]

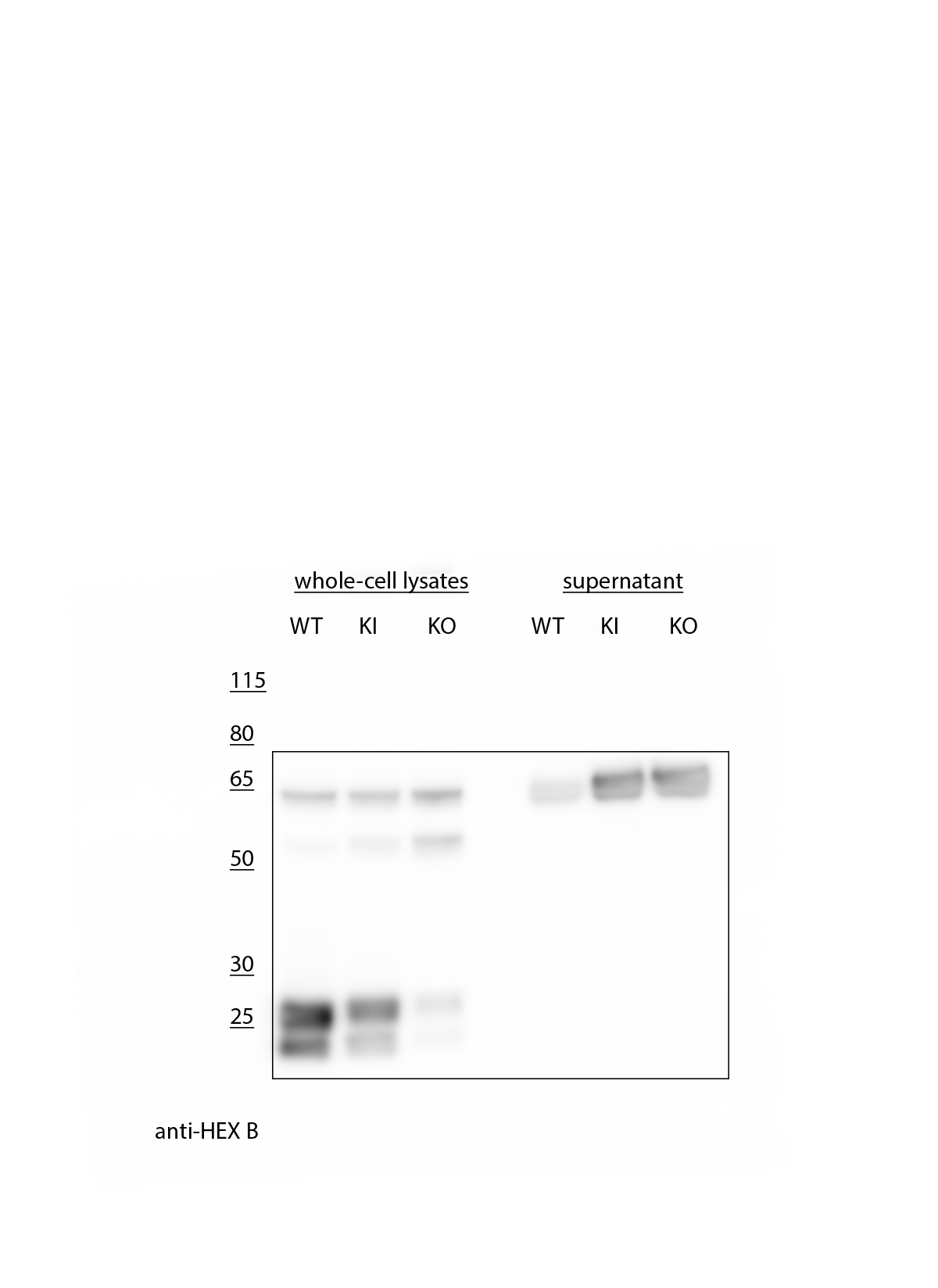

Supplement: Supplementary file 9 — Source data Fig. 6 [file 44318_2024_305_MOESM9_ESM.zip › Figure 6/6E/source data HEX B.tif]

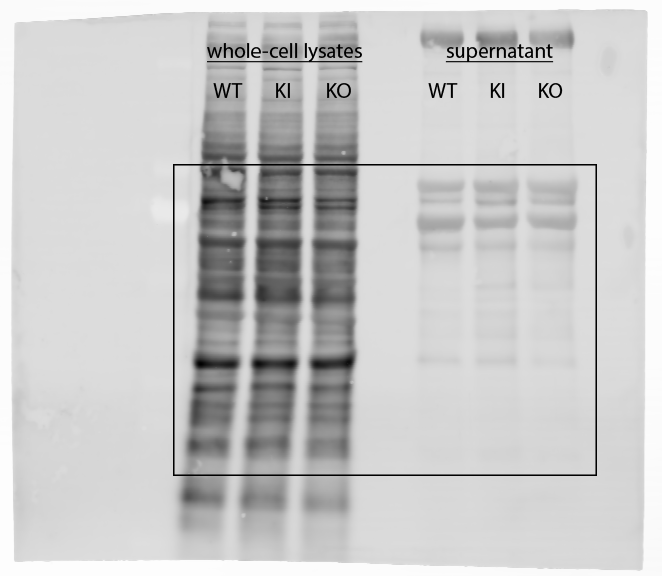

Supplement: Supplementary file 9 — Source data Fig. 6 [file 44318_2024_305_MOESM9_ESM.zip › Figure 6/6E/source data no stain for Hex B, CTS L.tif]

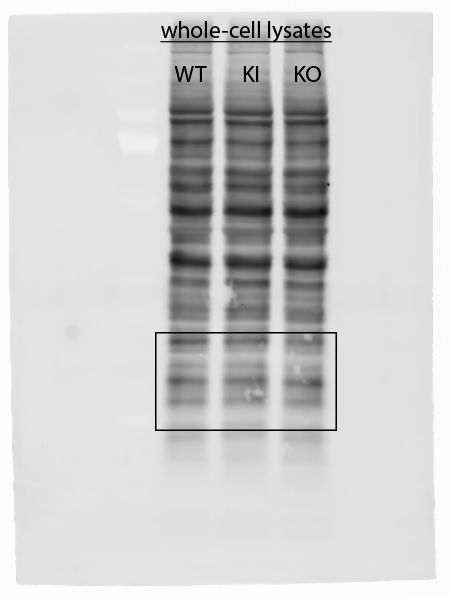

Supplement: Supplementary file 9 — Source data Fig. 6 [file 44318_2024_305_MOESM9_ESM.zip › Figure 6/6E/source data no stain for LYSET.tif]

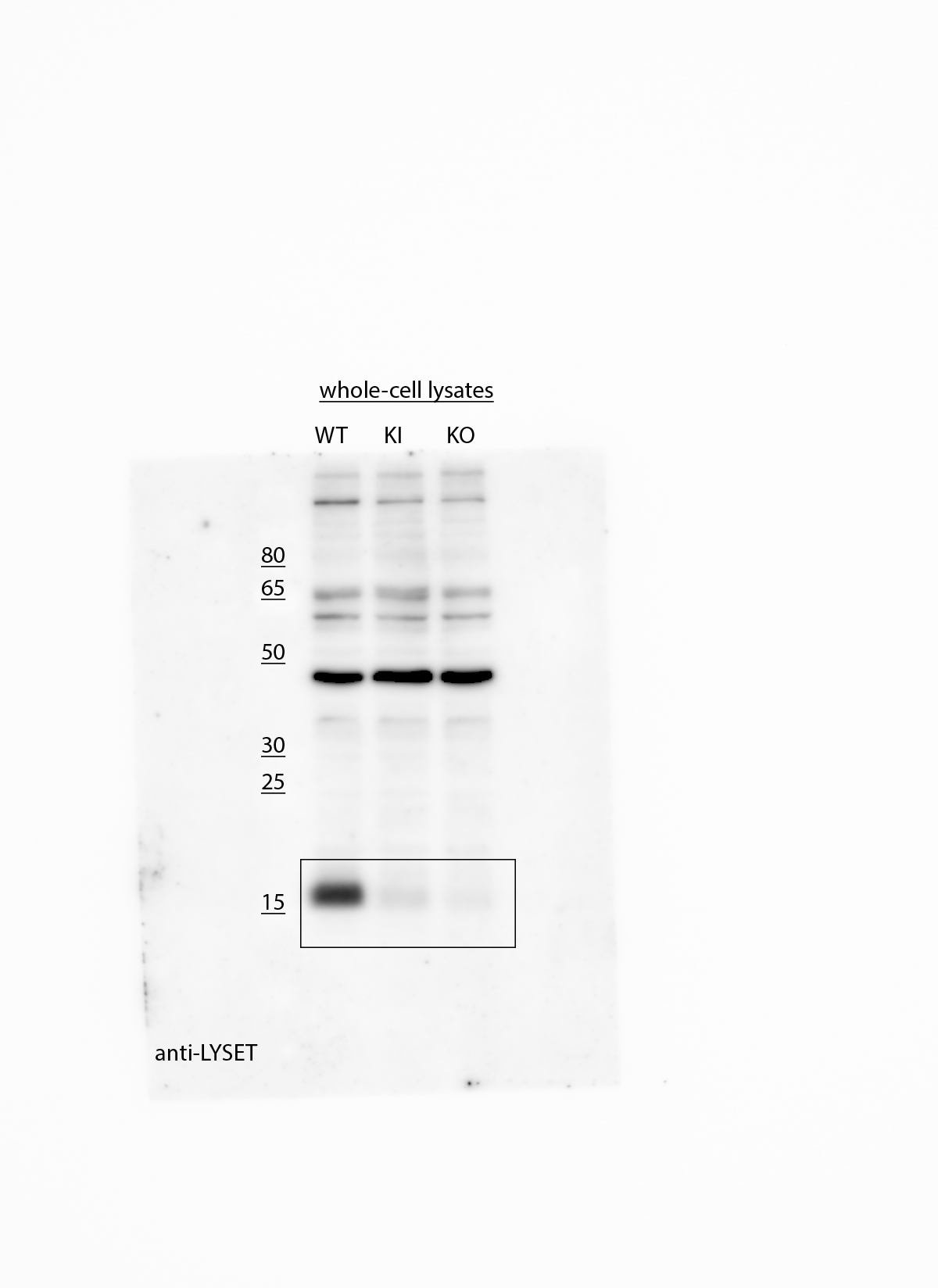

Supplement: Supplementary file 9 — Source data Fig. 6 [file 44318_2024_305_MOESM9_ESM.zip › Figure 6/6E/source data LYSET short exp..tif]

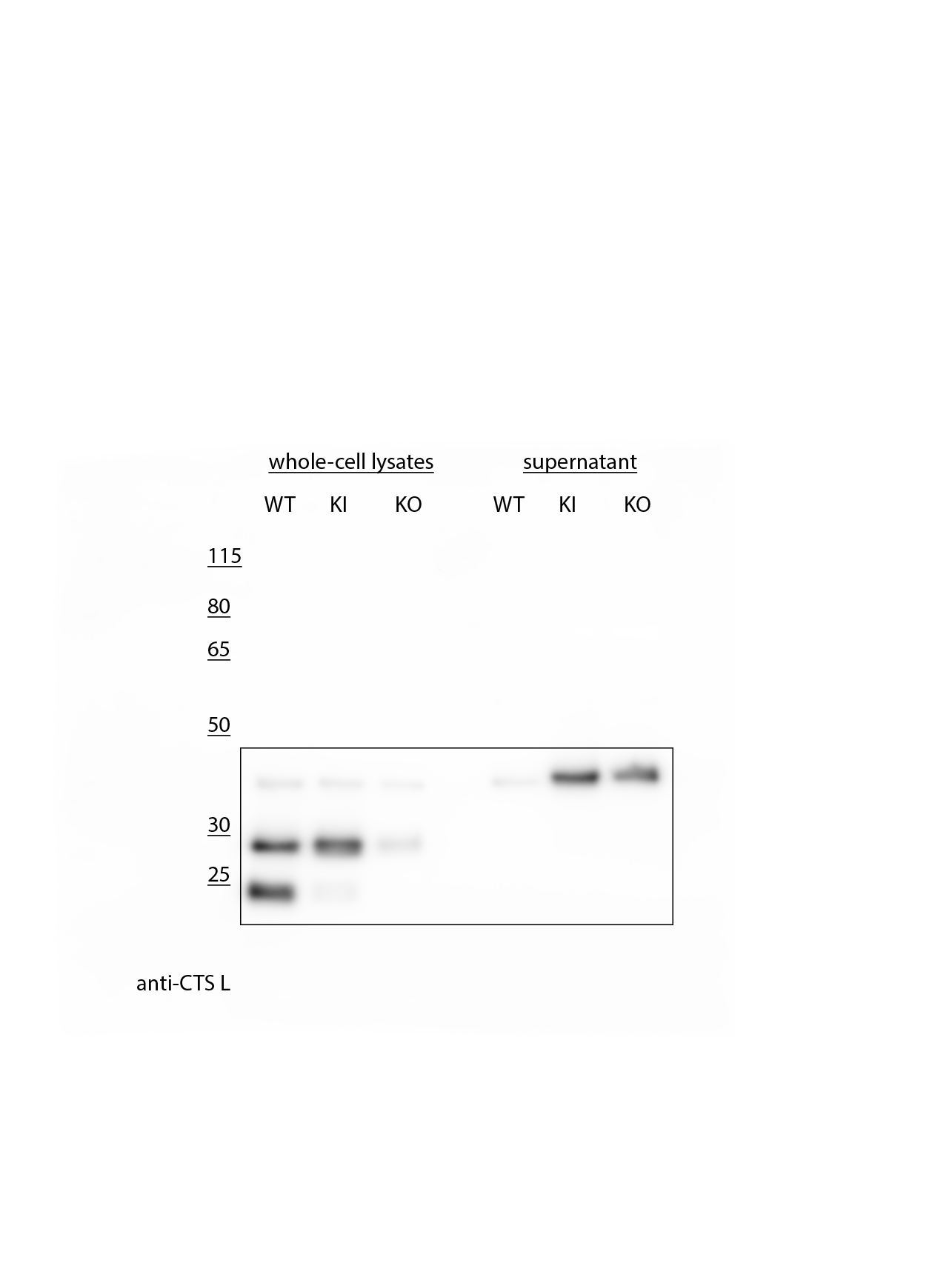

Supplement: Supplementary file 9 — Source data Fig. 6 [file 44318_2024_305_MOESM9_ESM.zip › Figure 6/6E/source data CTSL.tif]

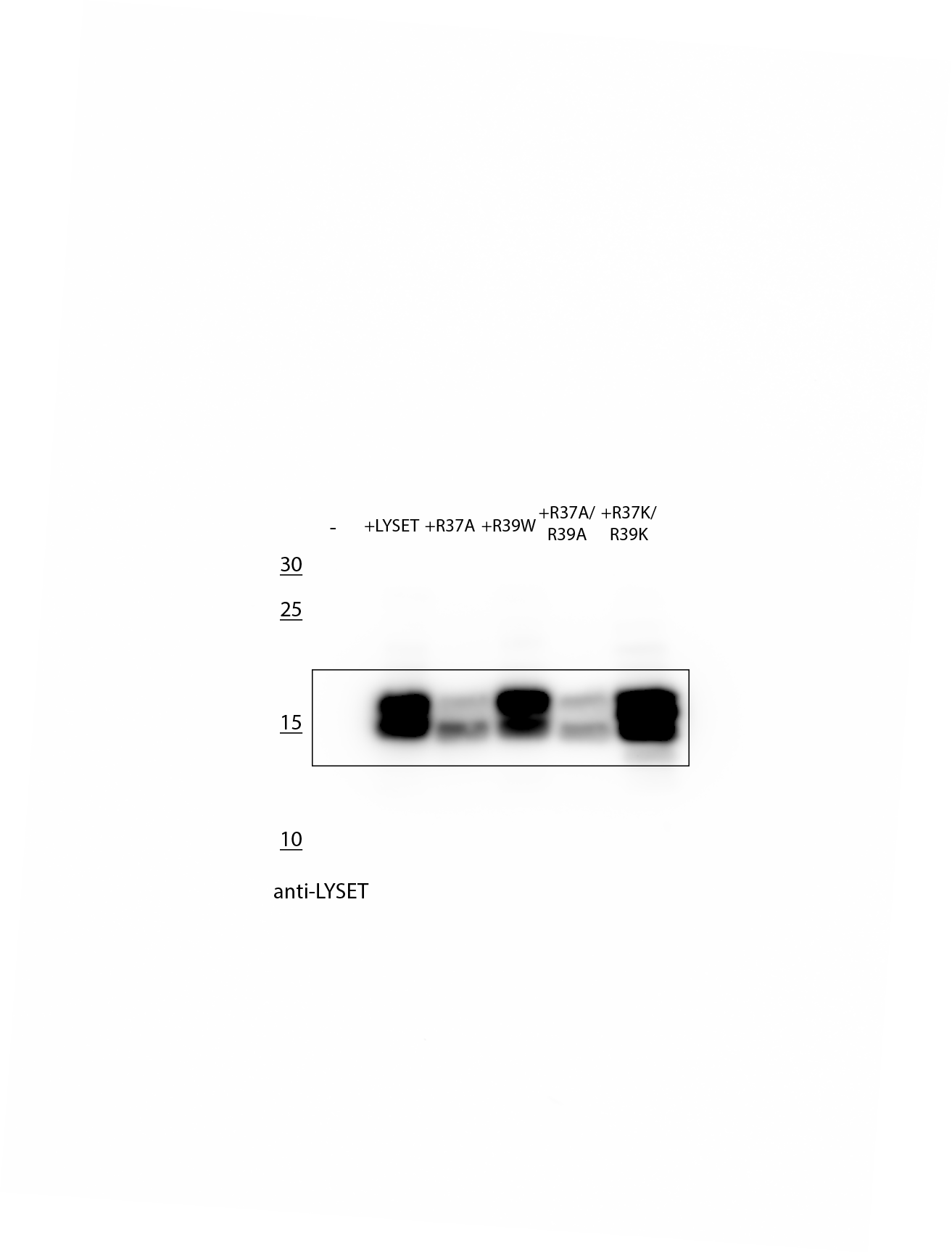

Supplement: Supplementary file 9 — Source data Fig. 6 [file 44318_2024_305_MOESM9_ESM.zip › Figure 6/6C/source data LYSET long exp..tif]

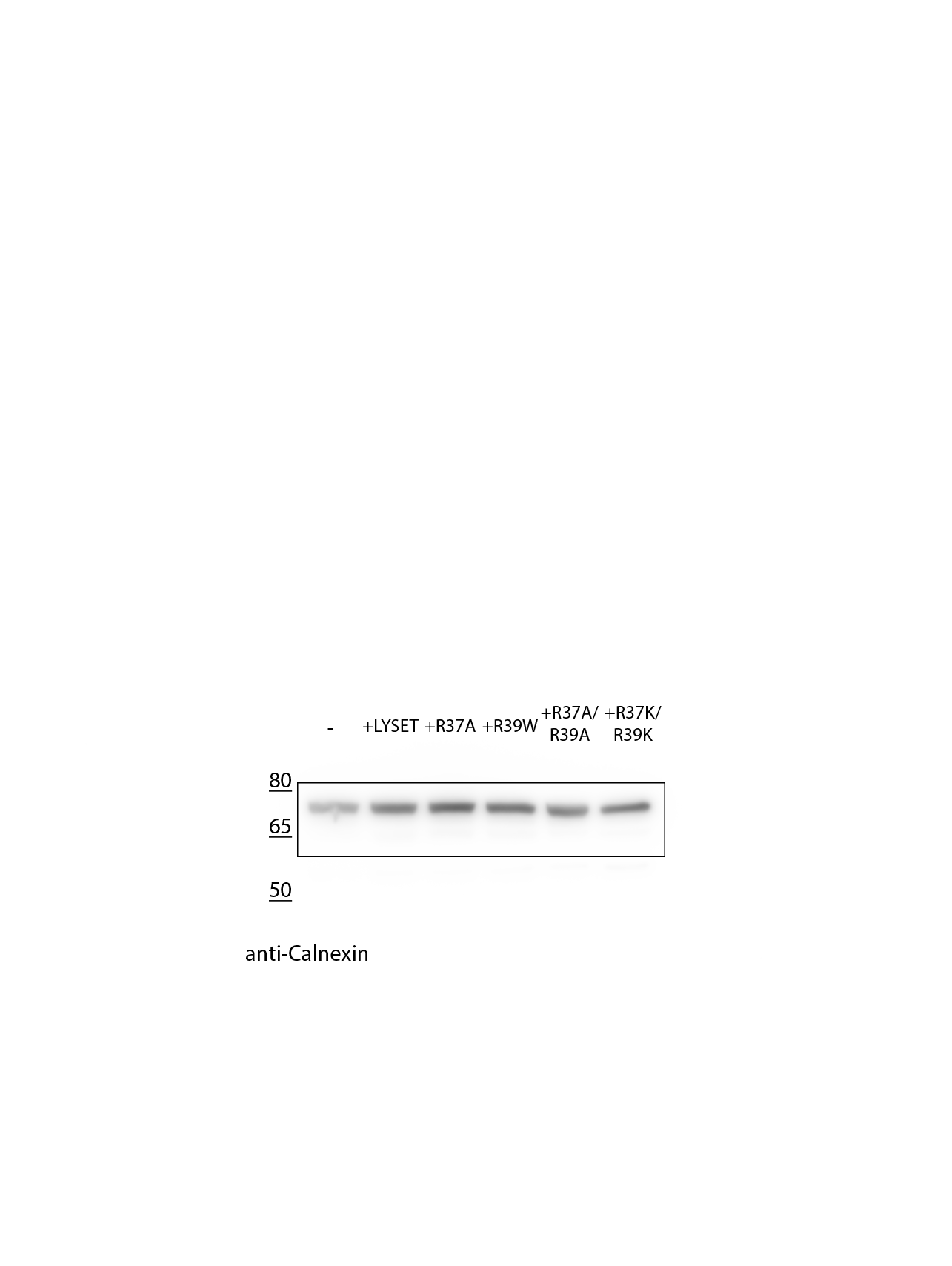

Supplement: Supplementary file 9 — Source data Fig. 6 [file 44318_2024_305_MOESM9_ESM.zip › Figure 6/6C/source data Calnexin.tif]

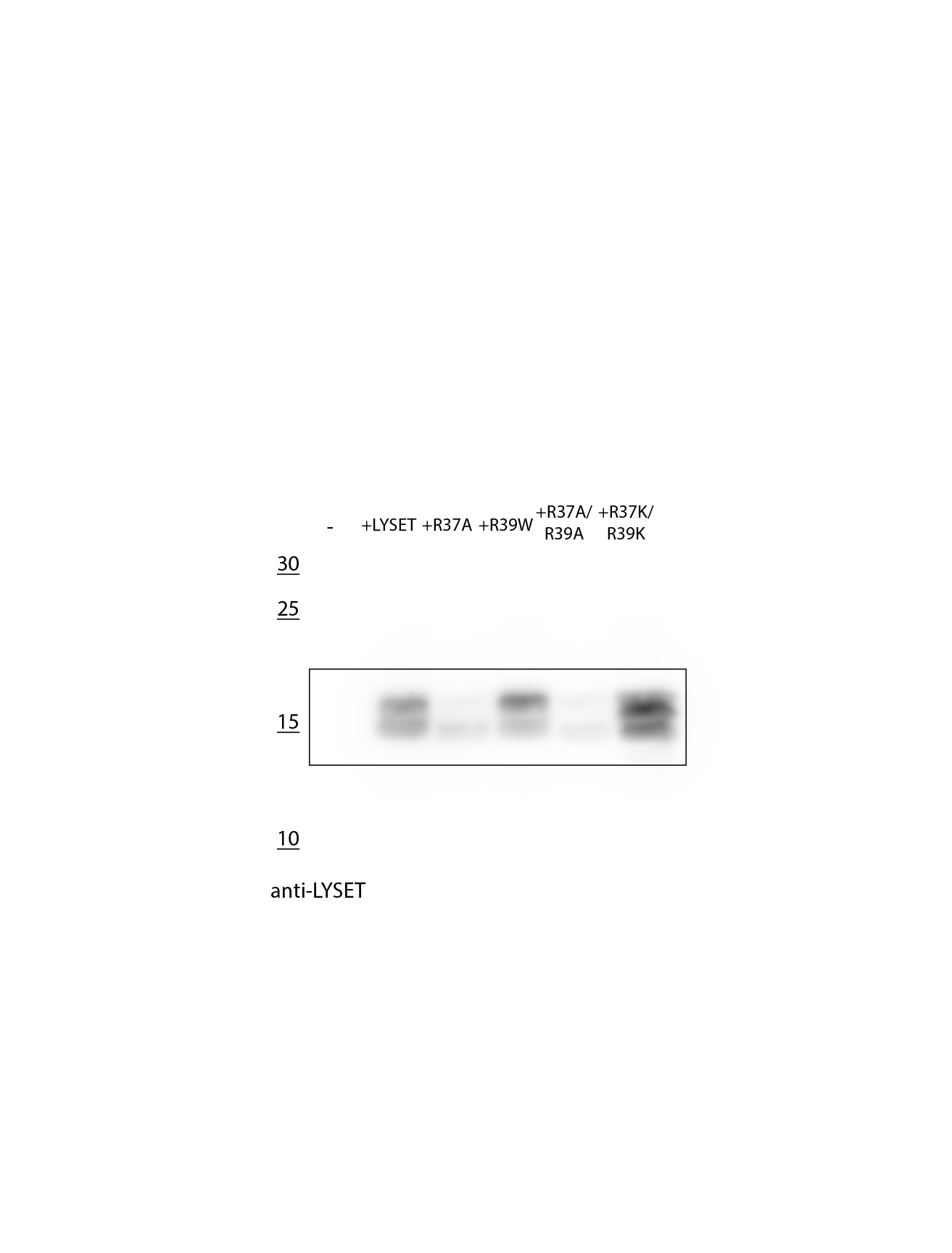

Supplement: Supplementary file 9 — Source data Fig. 6 [file 44318_2024_305_MOESM9_ESM.zip › Figure 6/6C/source data LYSET short exp..tif]

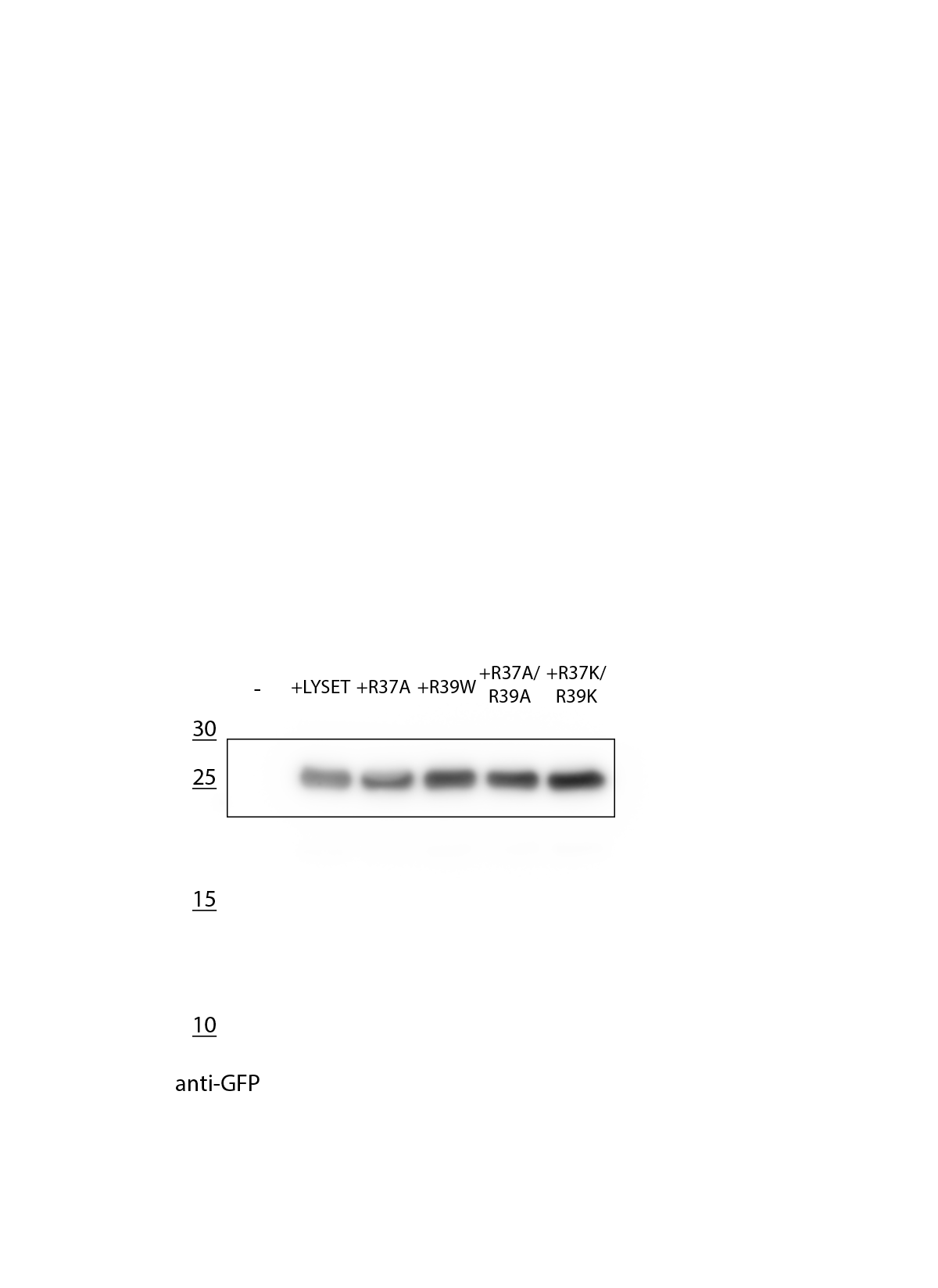

Supplement: Supplementary file 9 — Source data Fig. 6 [file 44318_2024_305_MOESM9_ESM.zip › Figure 6/6C/source data GFP.tif]

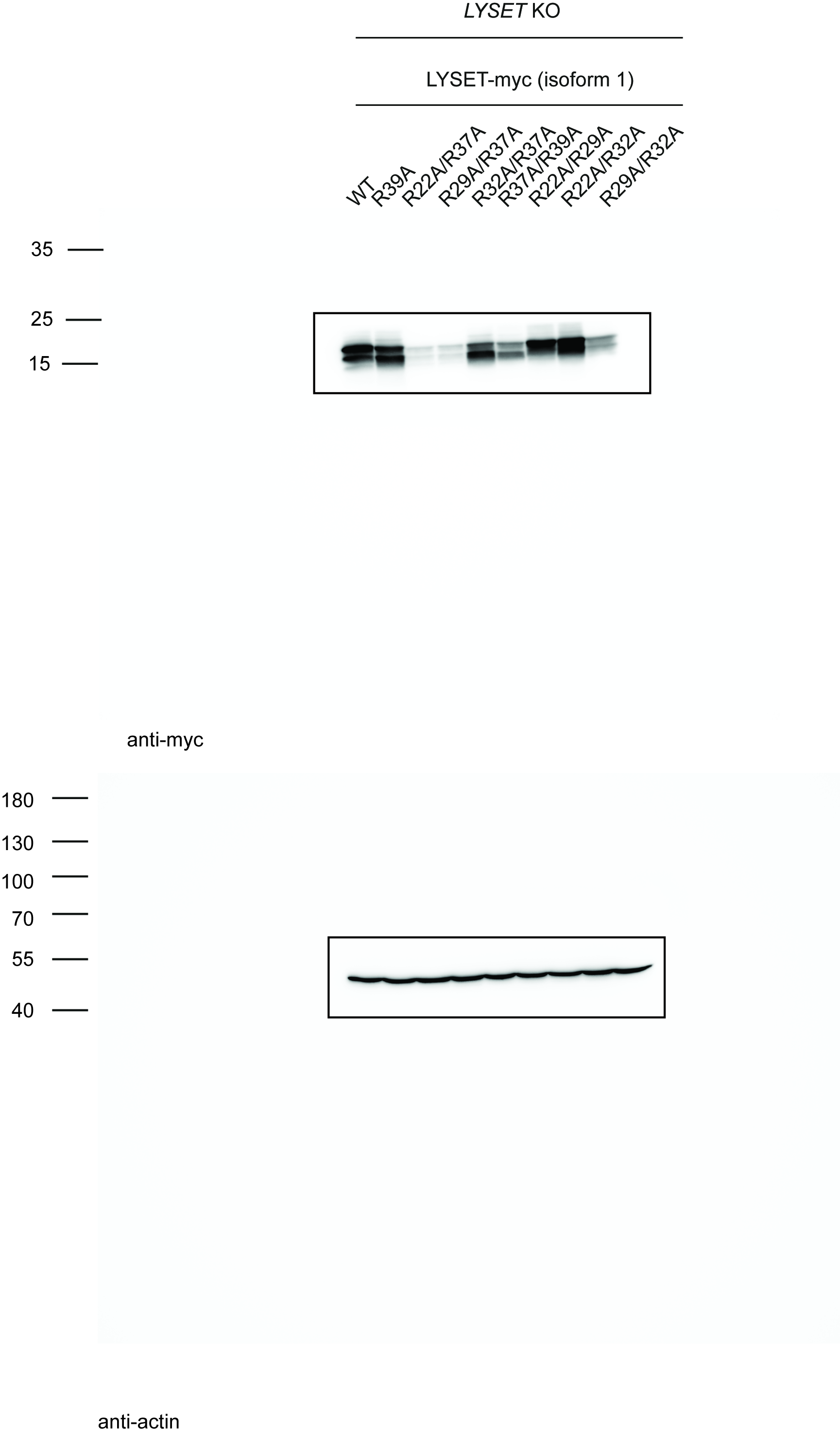

Supplement: Supplementary file 10 — Source data for Expanded View [file 44318_2024_305_MOESM10_ESM.zip › Figure EV1/EV1G/2) myc-actin.tif]

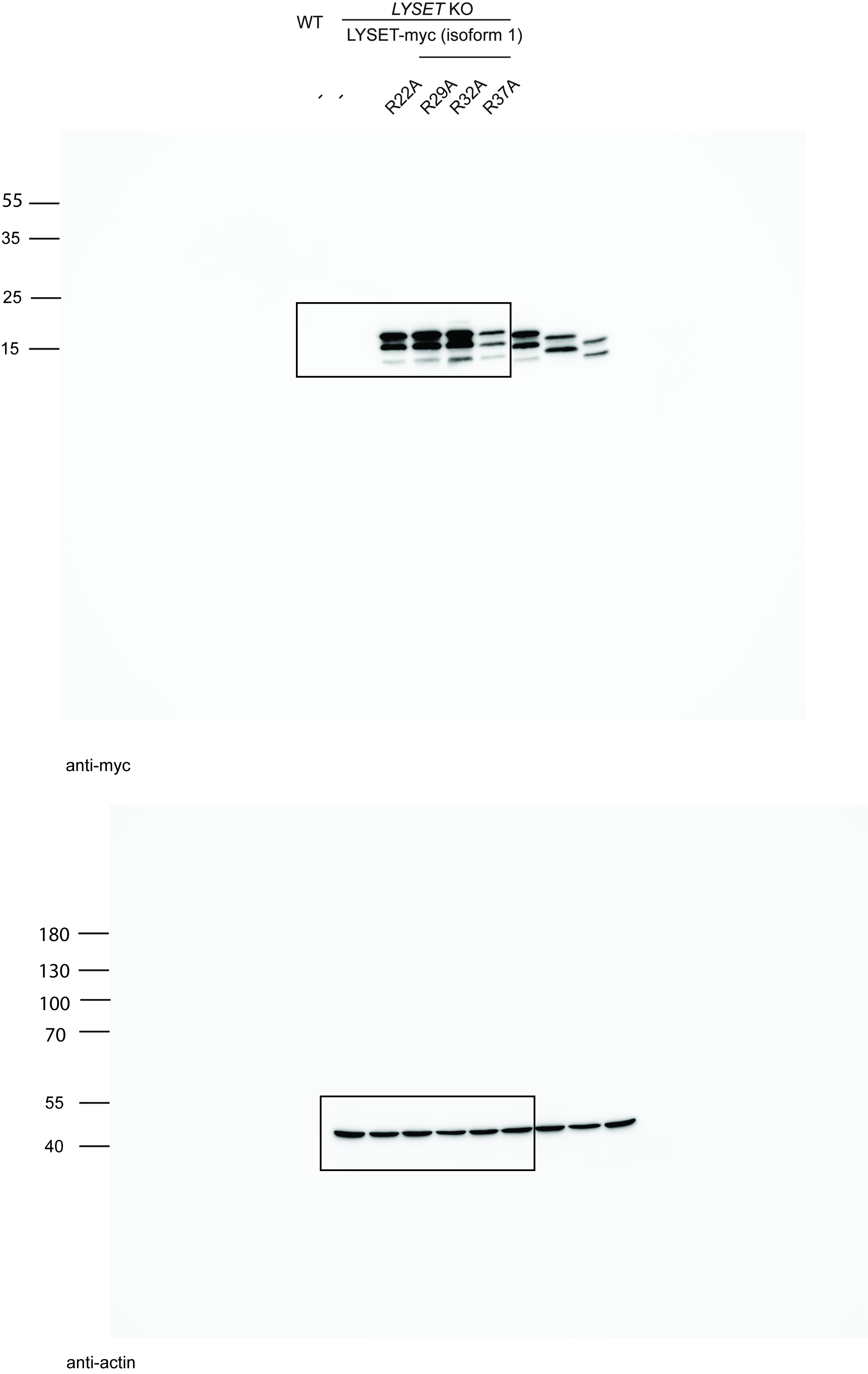

Supplement: Supplementary file 10 — Source data for Expanded View [file 44318_2024_305_MOESM10_ESM.zip › Figure EV1/EV1G/1) myc-actin.tif]

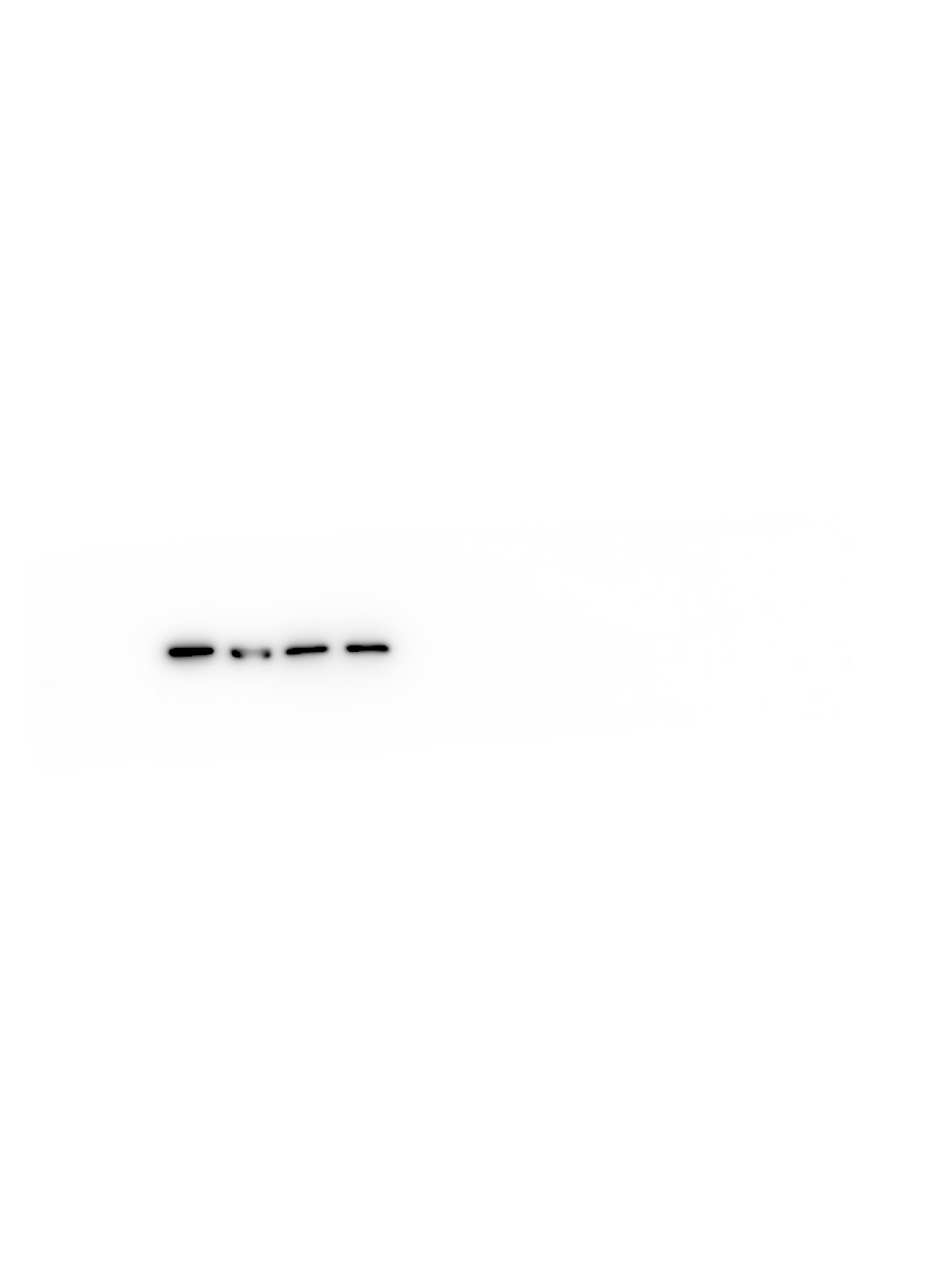

Supplement: Supplementary file 10 — Source data for Expanded View [file 44318_2024_305_MOESM10_ESM.zip › Figure EV1/EV1F/COPB shorter exposure 16bit original 20240313_162823-01_Ch_Chemi.tif]

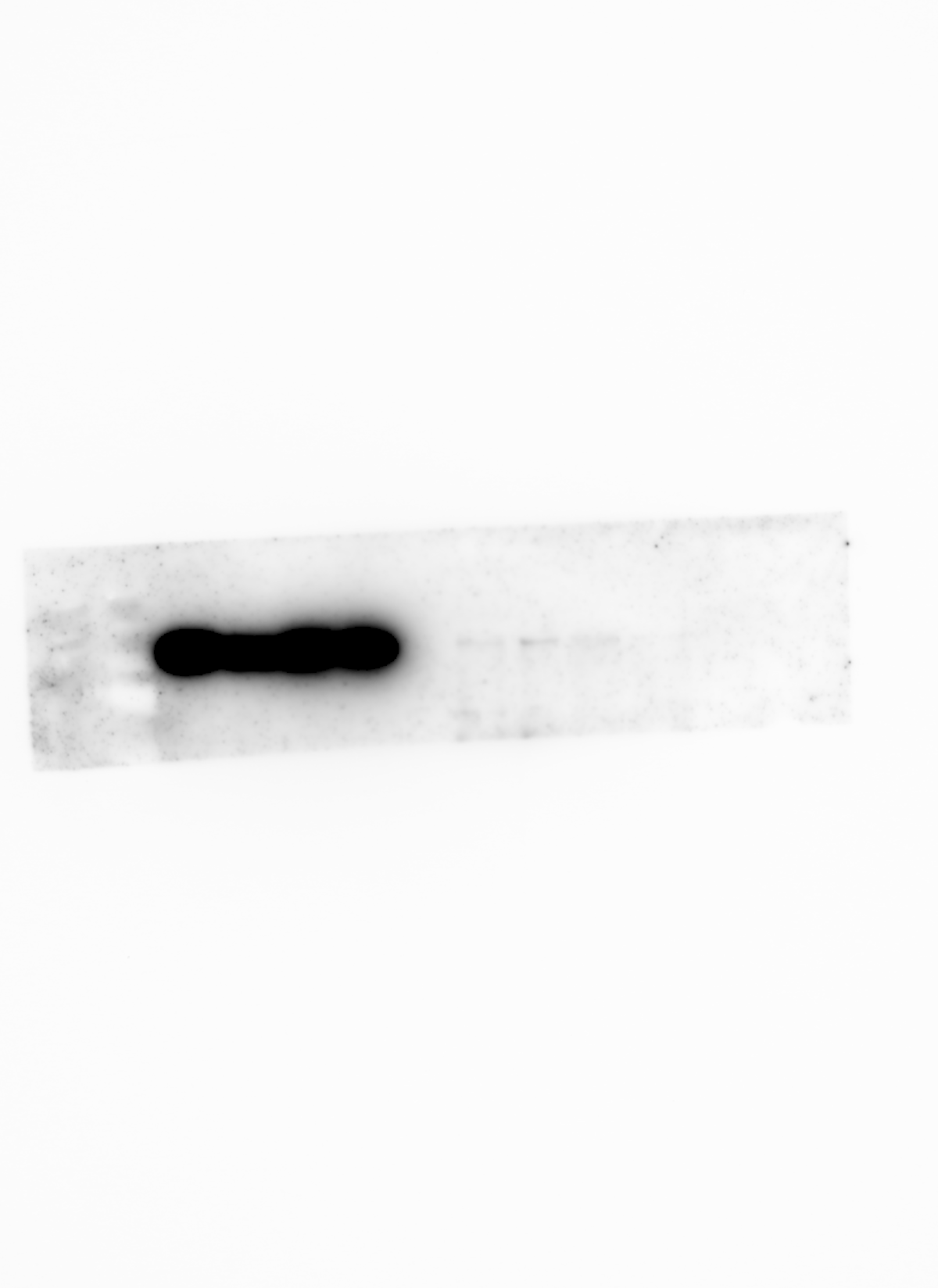

Supplement: Supplementary file 10 — Source data for Expanded View [file 44318_2024_305_MOESM10_ESM.zip › Figure EV1/EV1F/COPB 16bit original 20240313_162823-25_Ch_Chemi.tif]

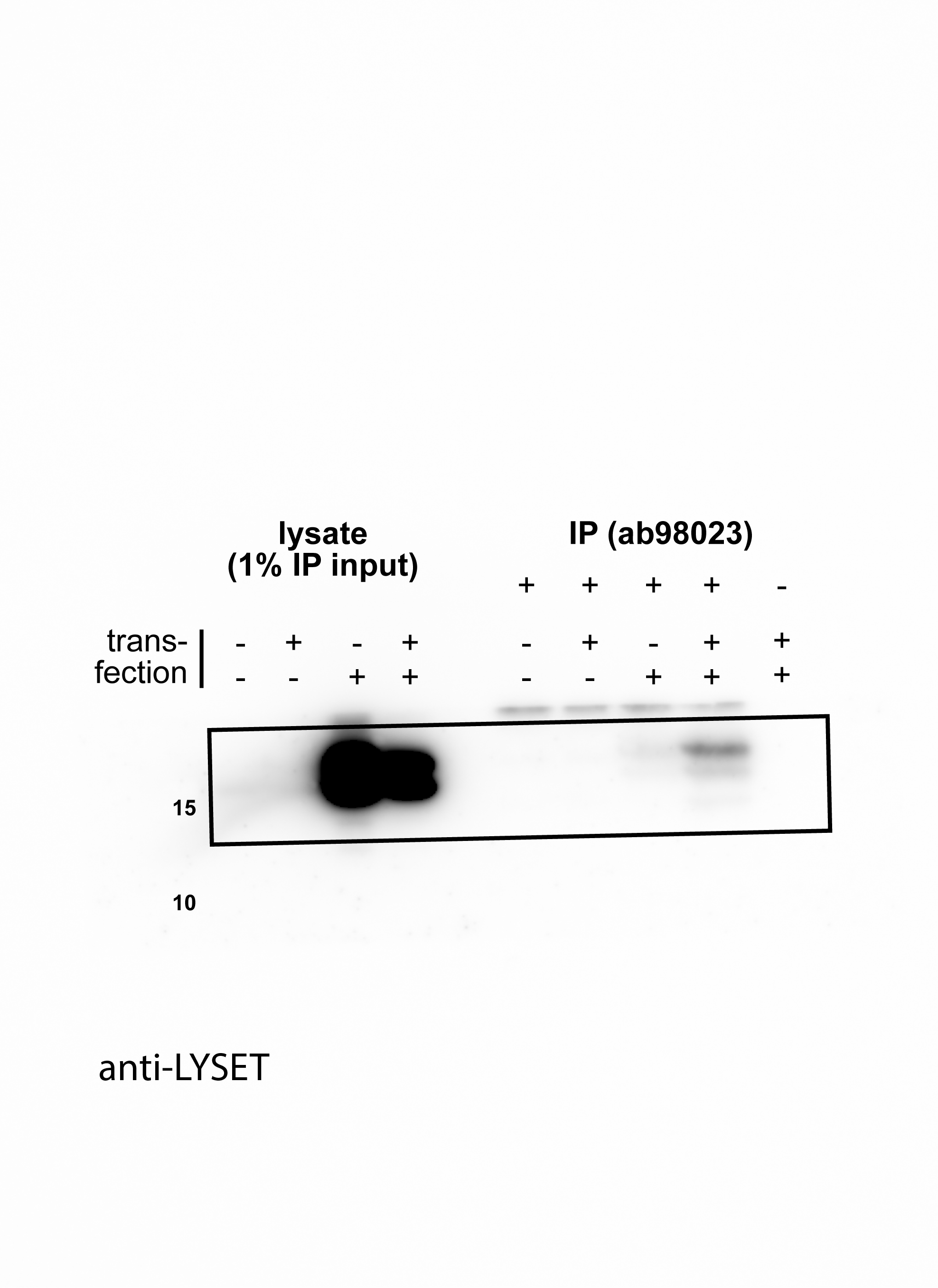

Supplement: Supplementary file 10 — Source data for Expanded View [file 44318_2024_305_MOESM10_ESM.zip › Figure EV1/EV1F/LYSET 8bit annotated 20240313_161855-12_Ch_Chemi-01.tif]

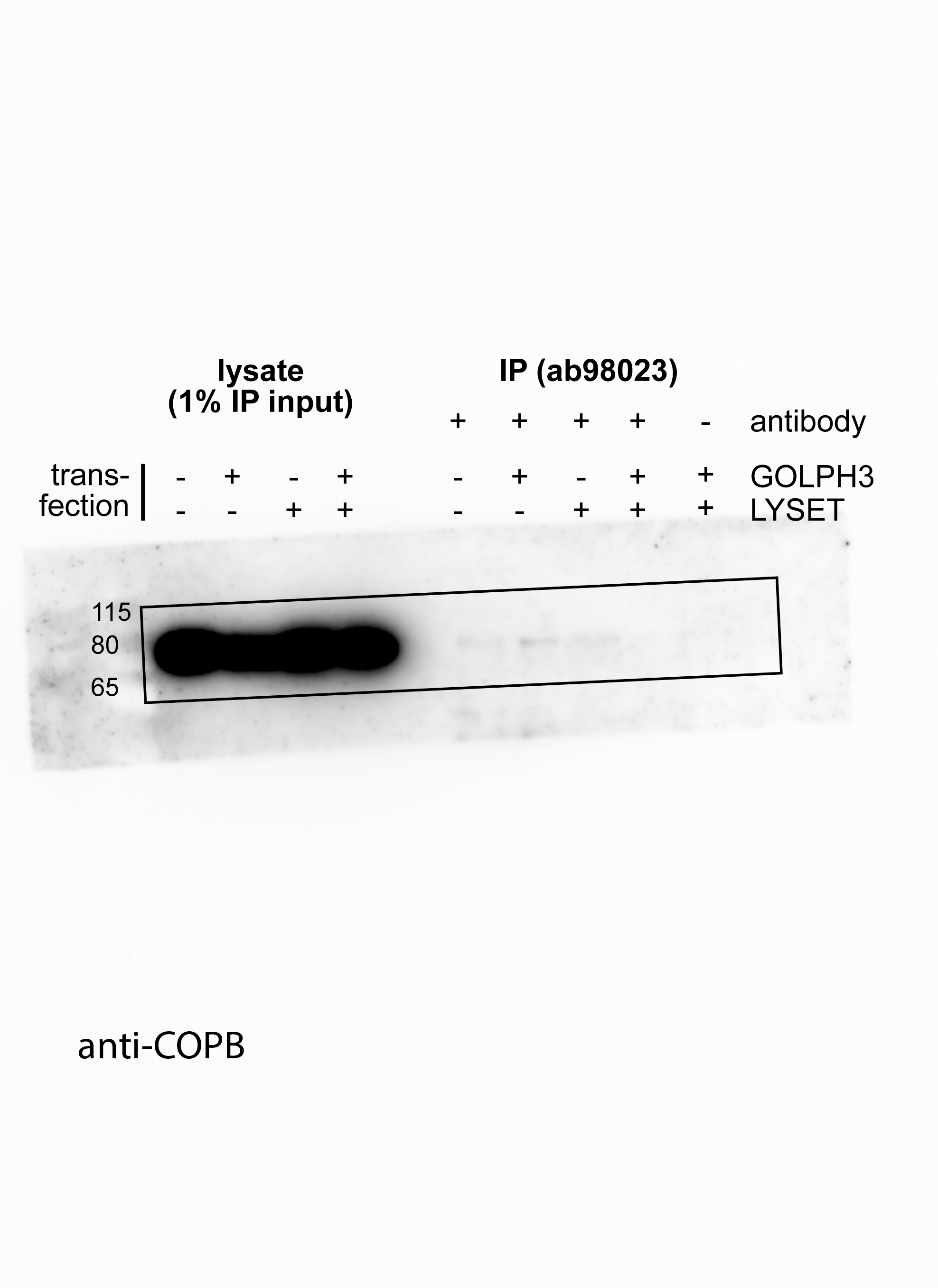

Supplement: Supplementary file 10 — Source data for Expanded View [file 44318_2024_305_MOESM10_ESM.zip › Figure EV1/EV1F/COPB 8bit annotated 20240313_162823-25_Ch_Chemi-01.tif]

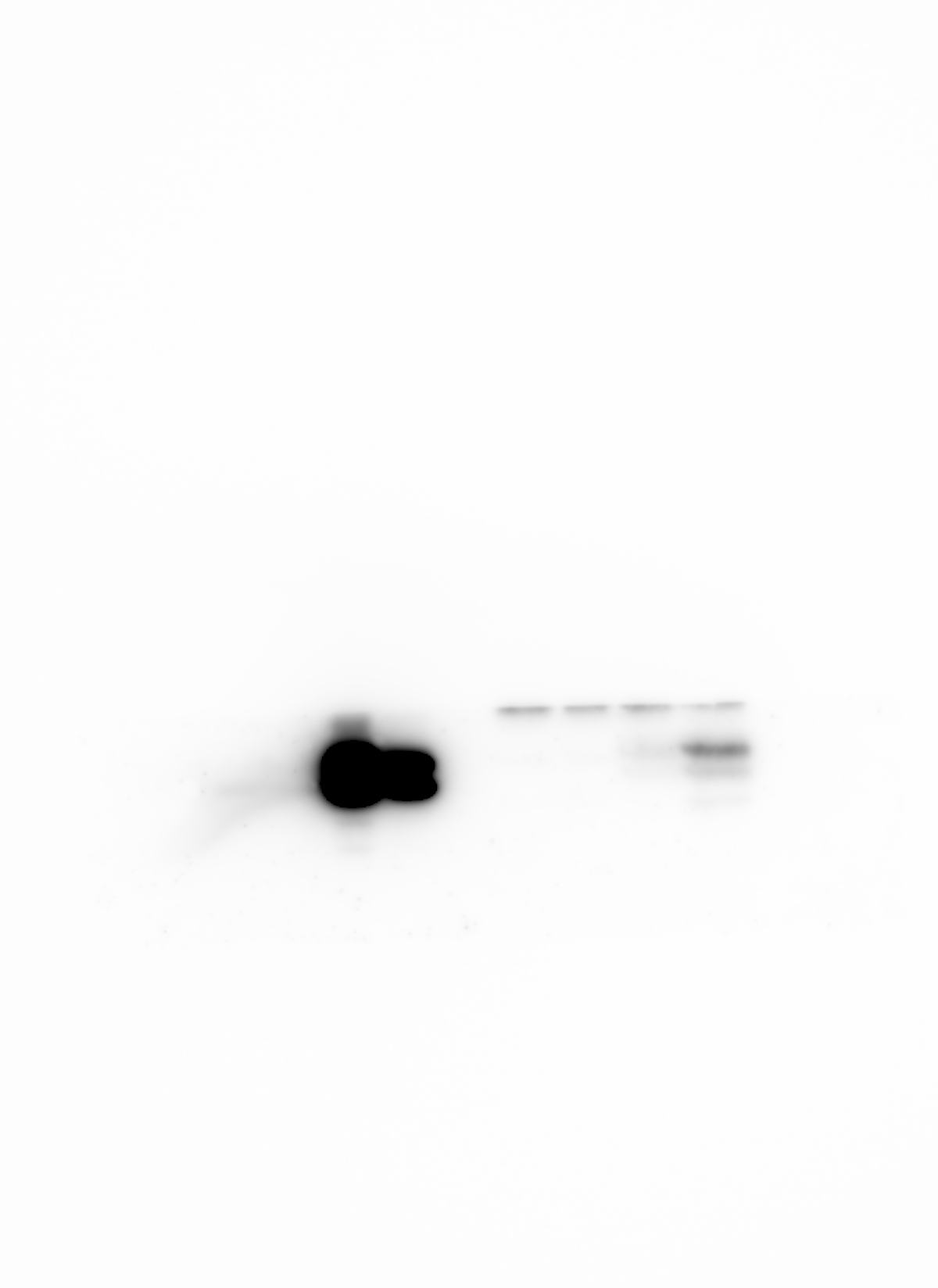

Supplement: Supplementary file 10 — Source data for Expanded View [file 44318_2024_305_MOESM10_ESM.zip › Figure EV1/EV1F/LYSET 16bit original 20240313_161855-12_Ch_Chemi.tif]

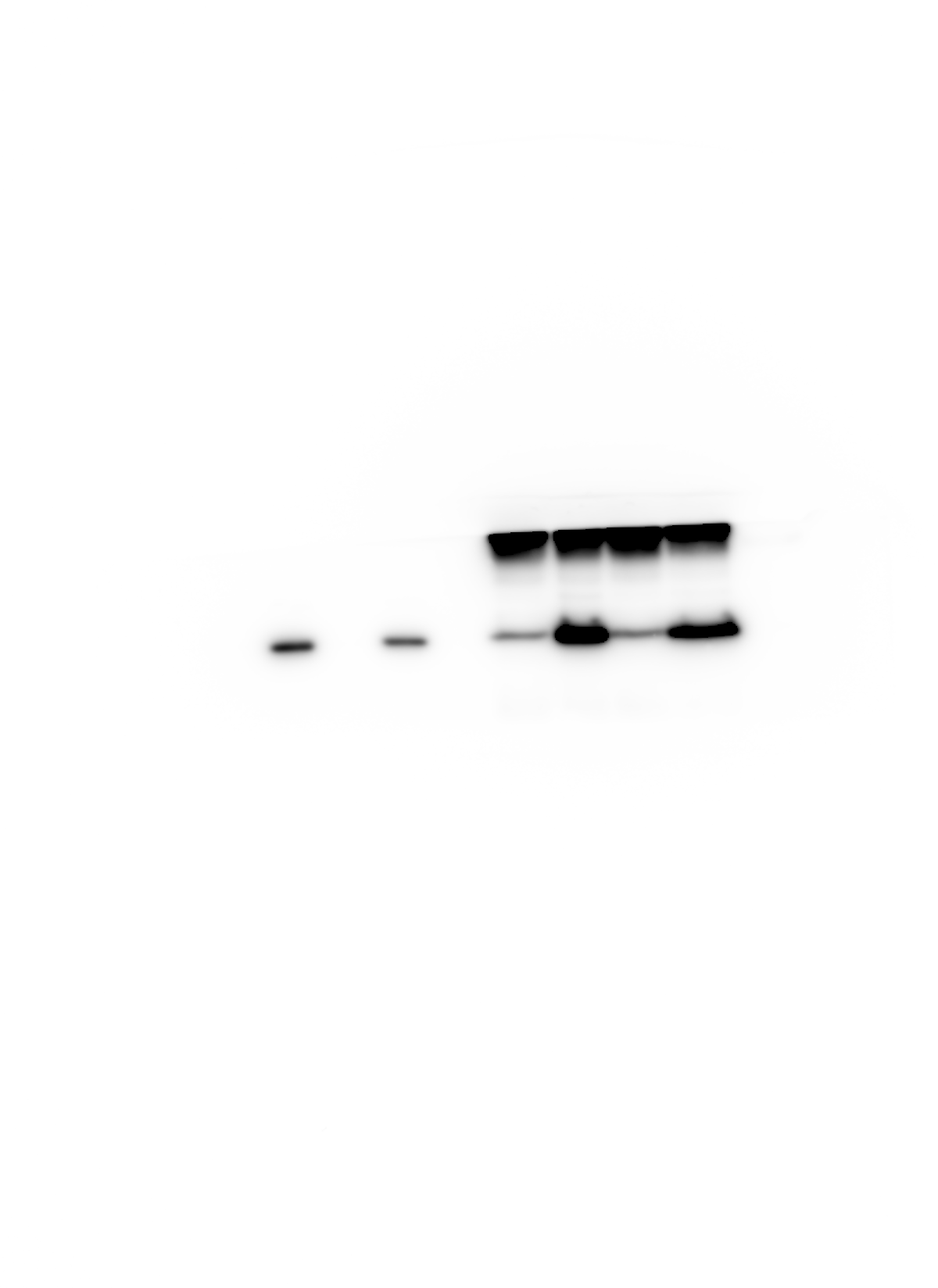

Supplement: Supplementary file 10 — Source data for Expanded View [file 44318_2024_305_MOESM10_ESM.zip › Figure EV1/EV1F/GOLPH3 16bit original 20240313_163740-02_Ch_Chemi.tif]

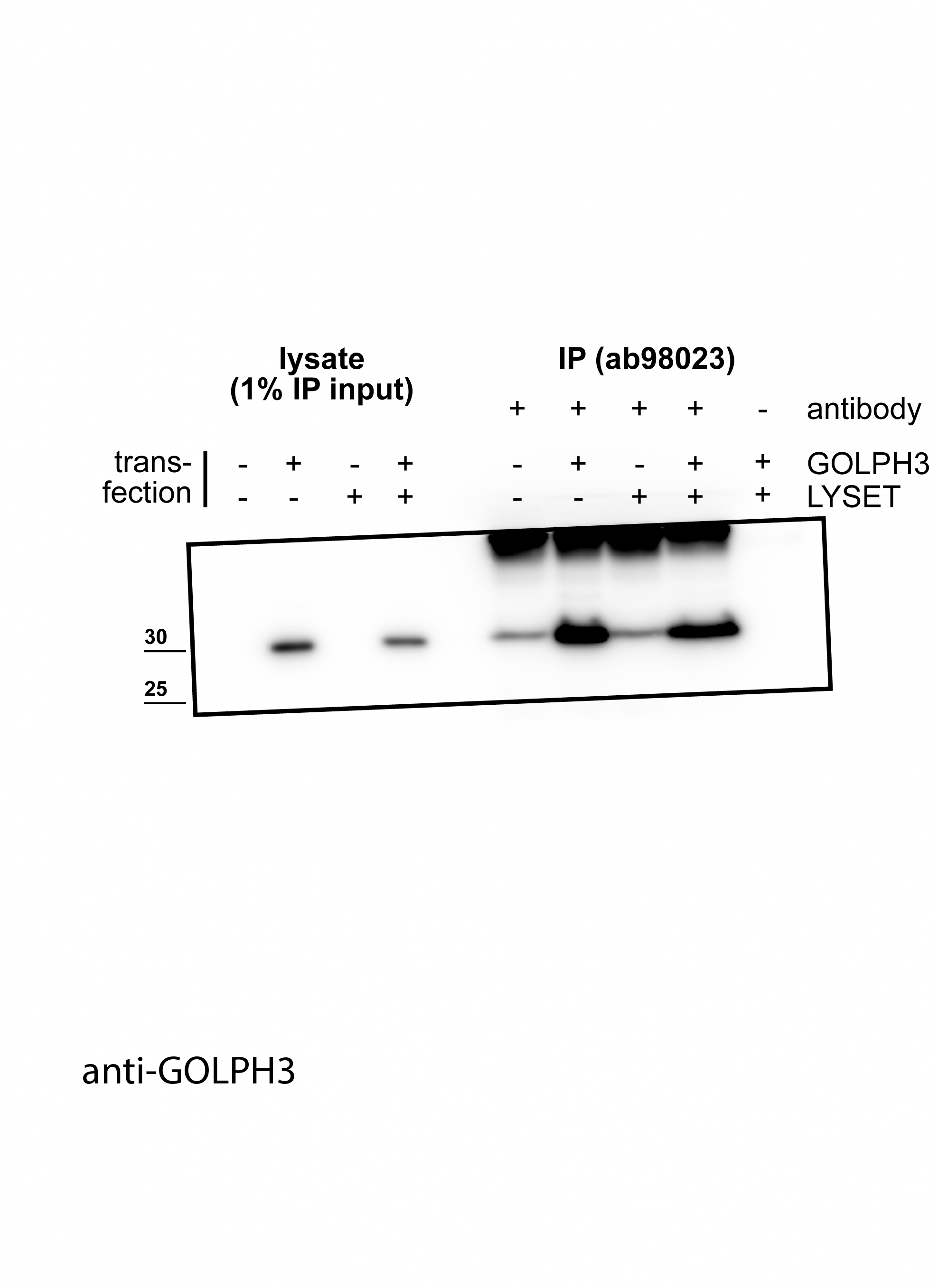

Supplement: Supplementary file 10 — Source data for Expanded View [file 44318_2024_305_MOESM10_ESM.zip › Figure EV1/EV1F/GOLPH3 8bit annotated 20240313_163740-02_Ch_Chemi-01.tif]

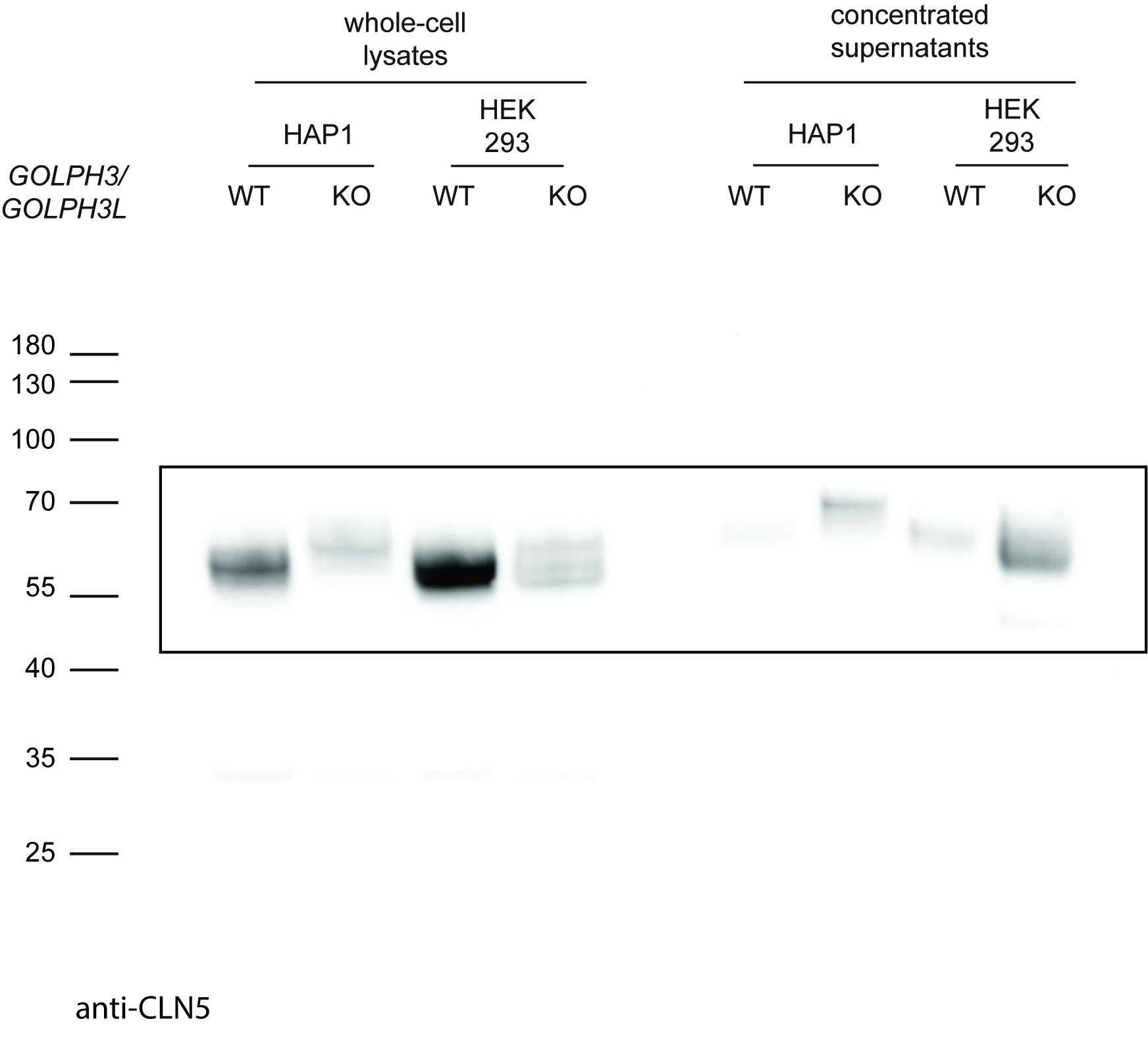

Supplement: Supplementary file 11 — Source data for Appendix [file 44318_2024_305_MOESM11_ESM.zip › Appendix/Appendix Figure S7/S7A/CLN5.tif]

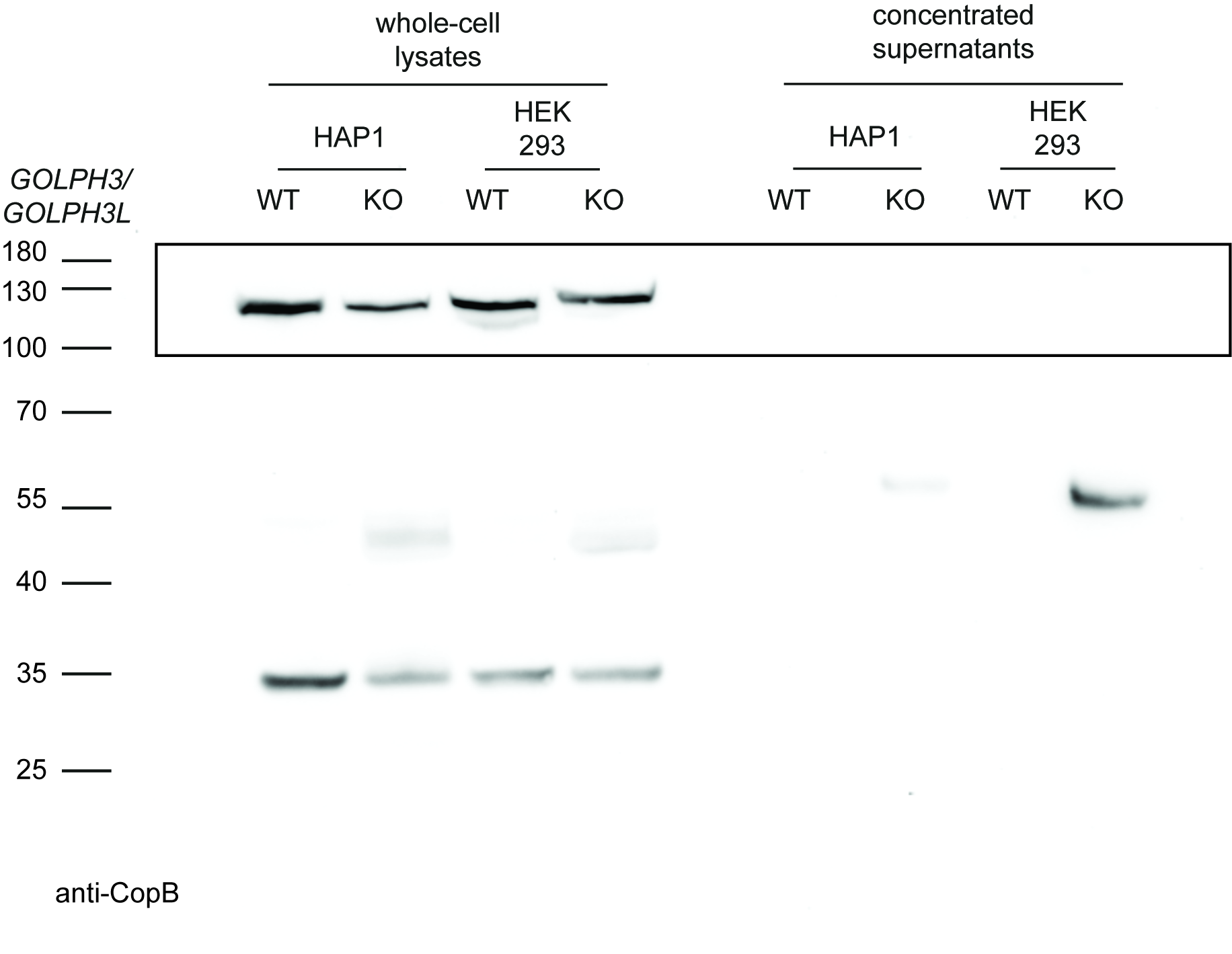

Supplement: Supplementary file 11 — Source data for Appendix [file 44318_2024_305_MOESM11_ESM.zip › Appendix/Appendix Figure S7/S7A/copB.tif]

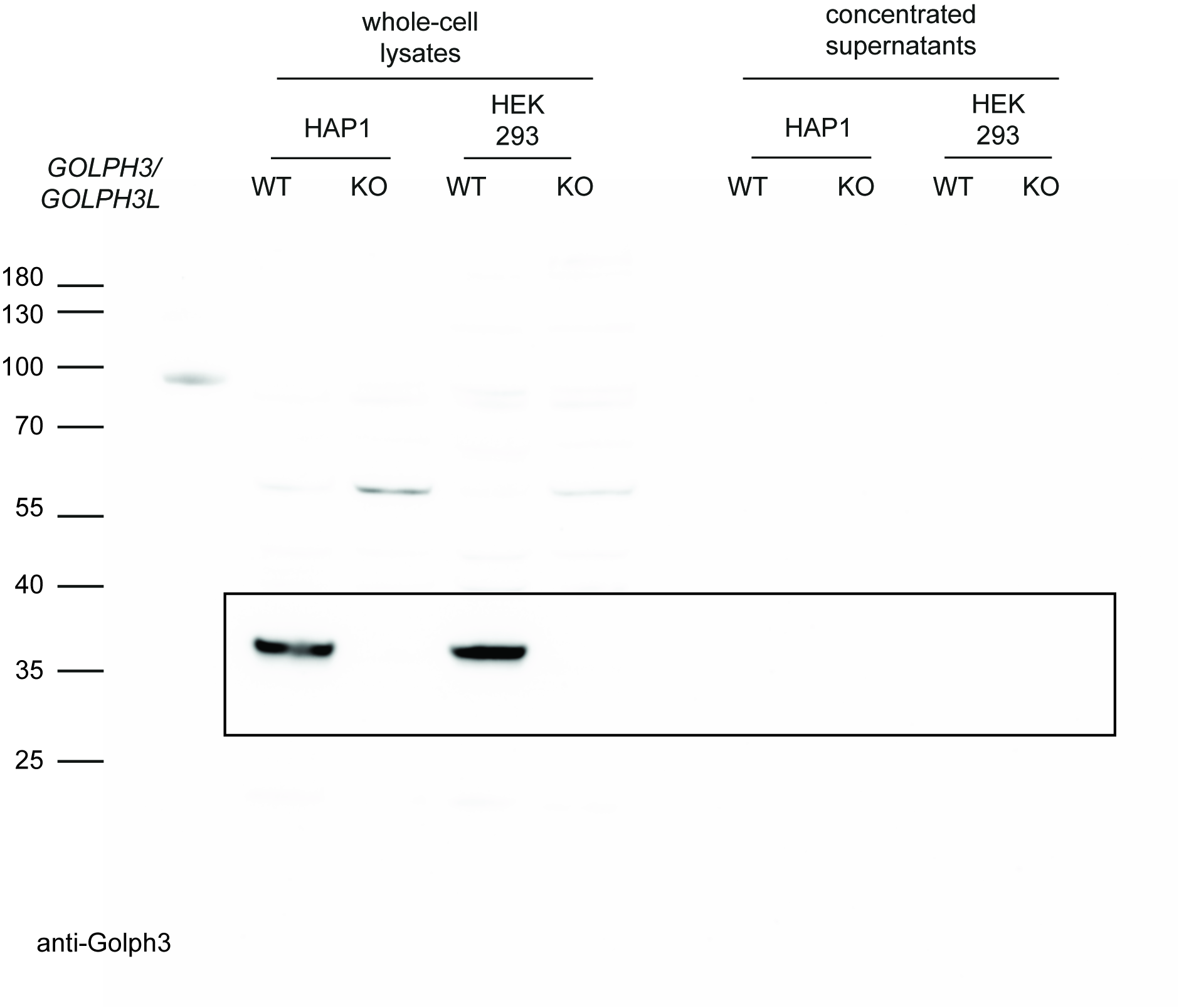

Supplement: Supplementary file 11 — Source data for Appendix [file 44318_2024_305_MOESM11_ESM.zip › Appendix/Appendix Figure S7/S7A/Golph3.tif]

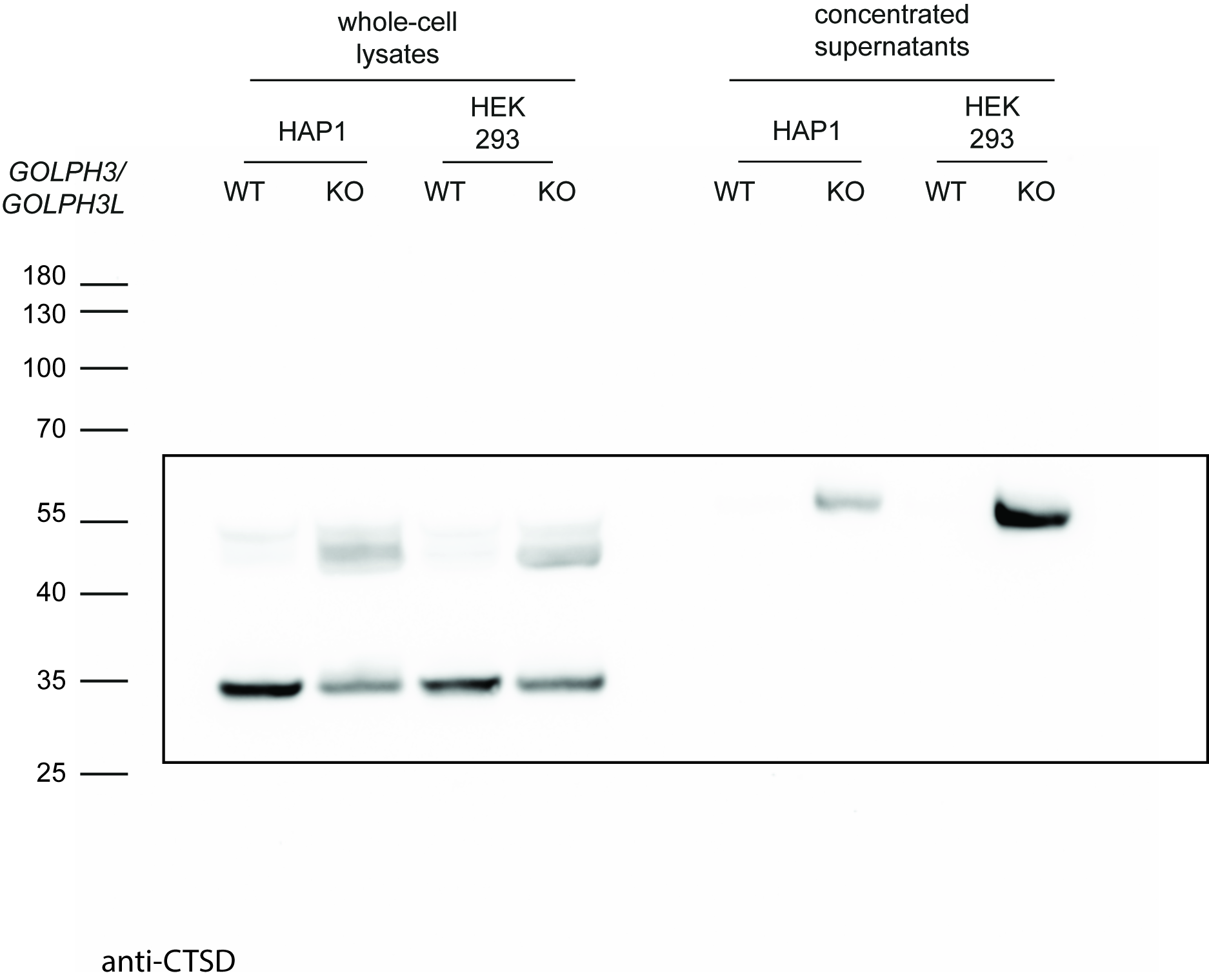

Supplement: Supplementary file 11 — Source data for Appendix [file 44318_2024_305_MOESM11_ESM.zip › Appendix/Appendix Figure S7/S7A/ctsD.tif]

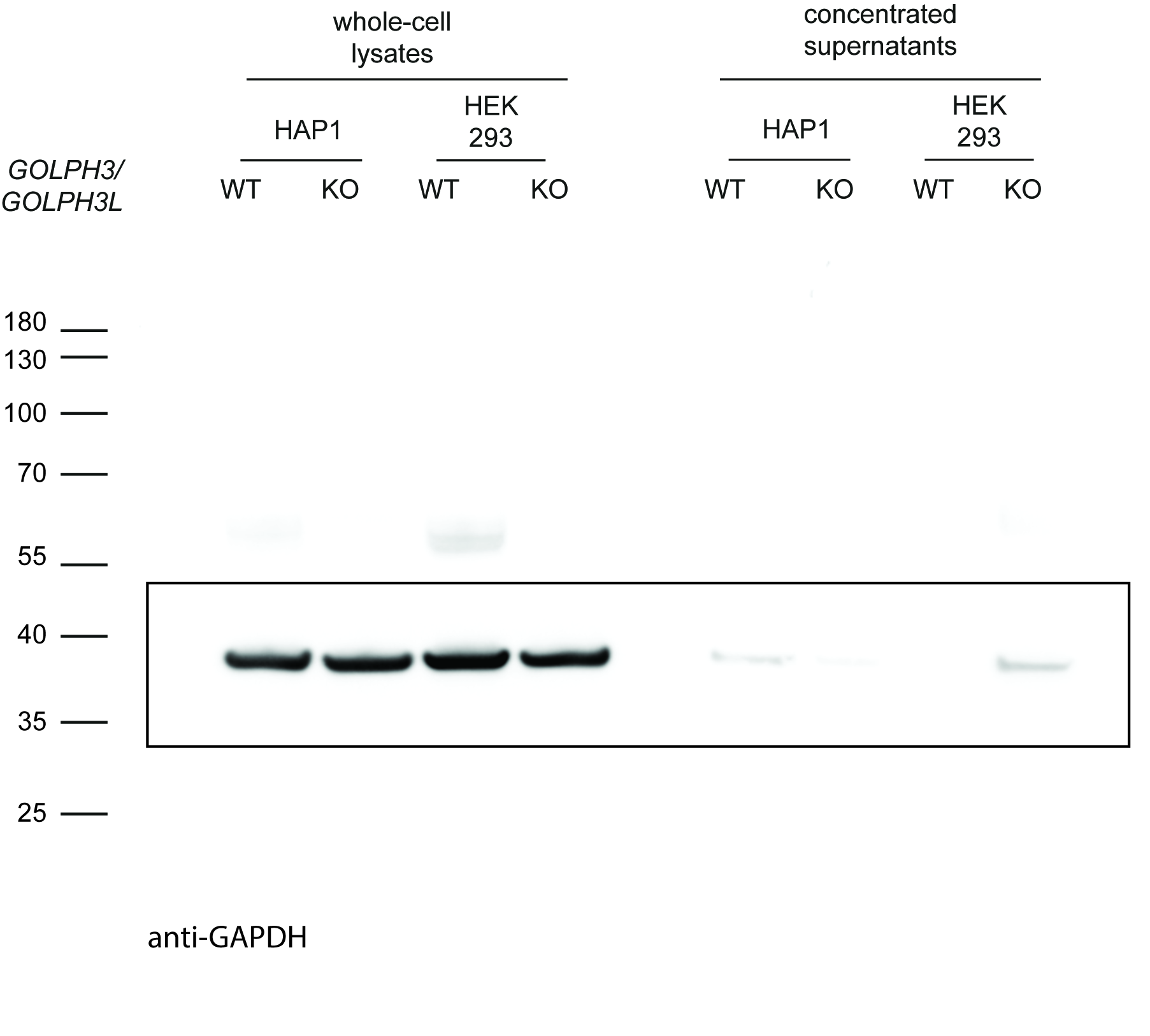

Supplement: Supplementary file 11 — Source data for Appendix [file 44318_2024_305_MOESM11_ESM.zip › Appendix/Appendix Figure S7/S7A/GAPDH.tif]

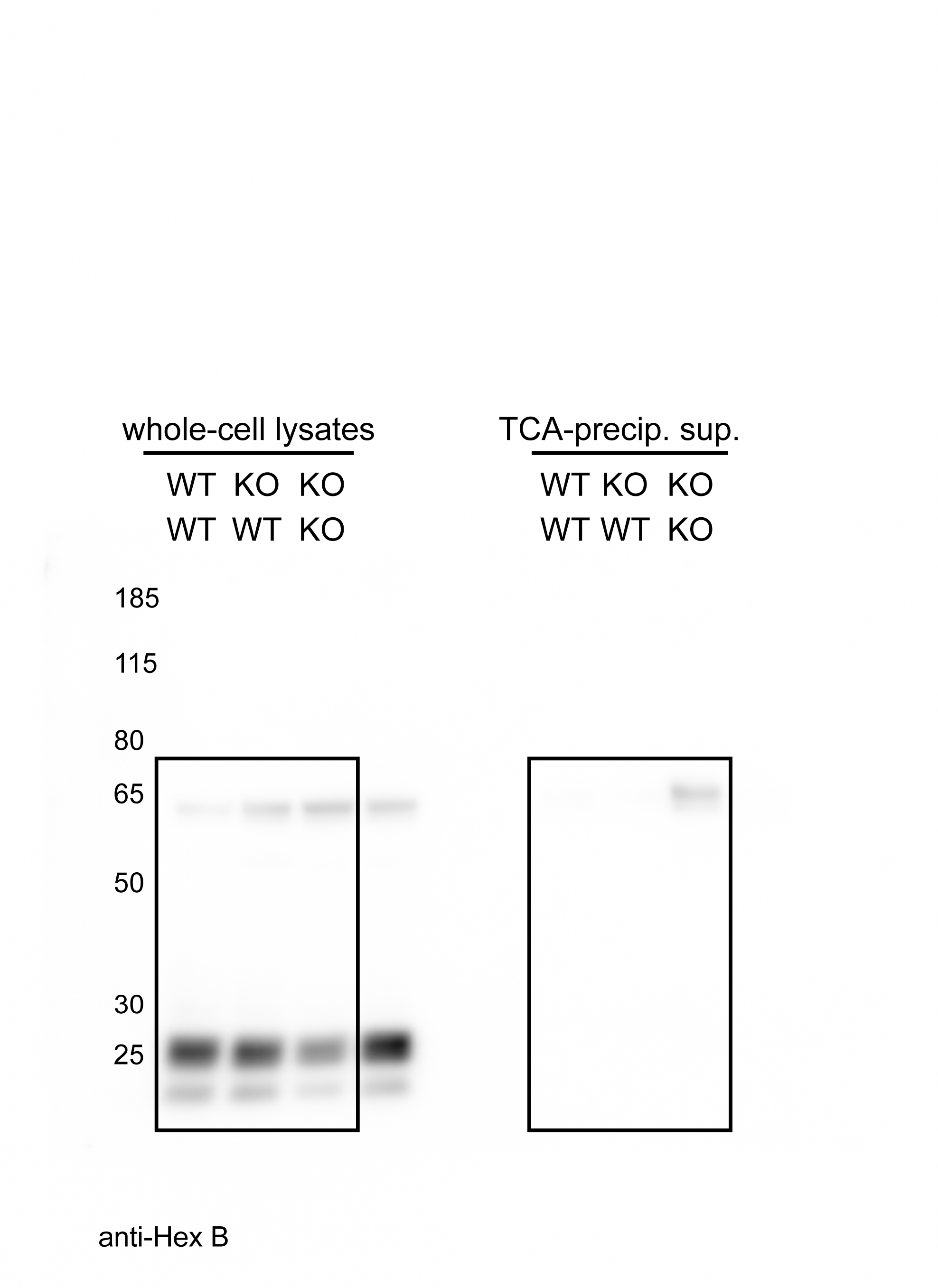

Supplement: Supplementary file 11 — Source data for Appendix [file 44318_2024_305_MOESM11_ESM.zip › Appendix/Appendix Figure S8/S8D/Hex B annotated 8bit 20240306_142708-02_Ch_Chemi-01.tif]

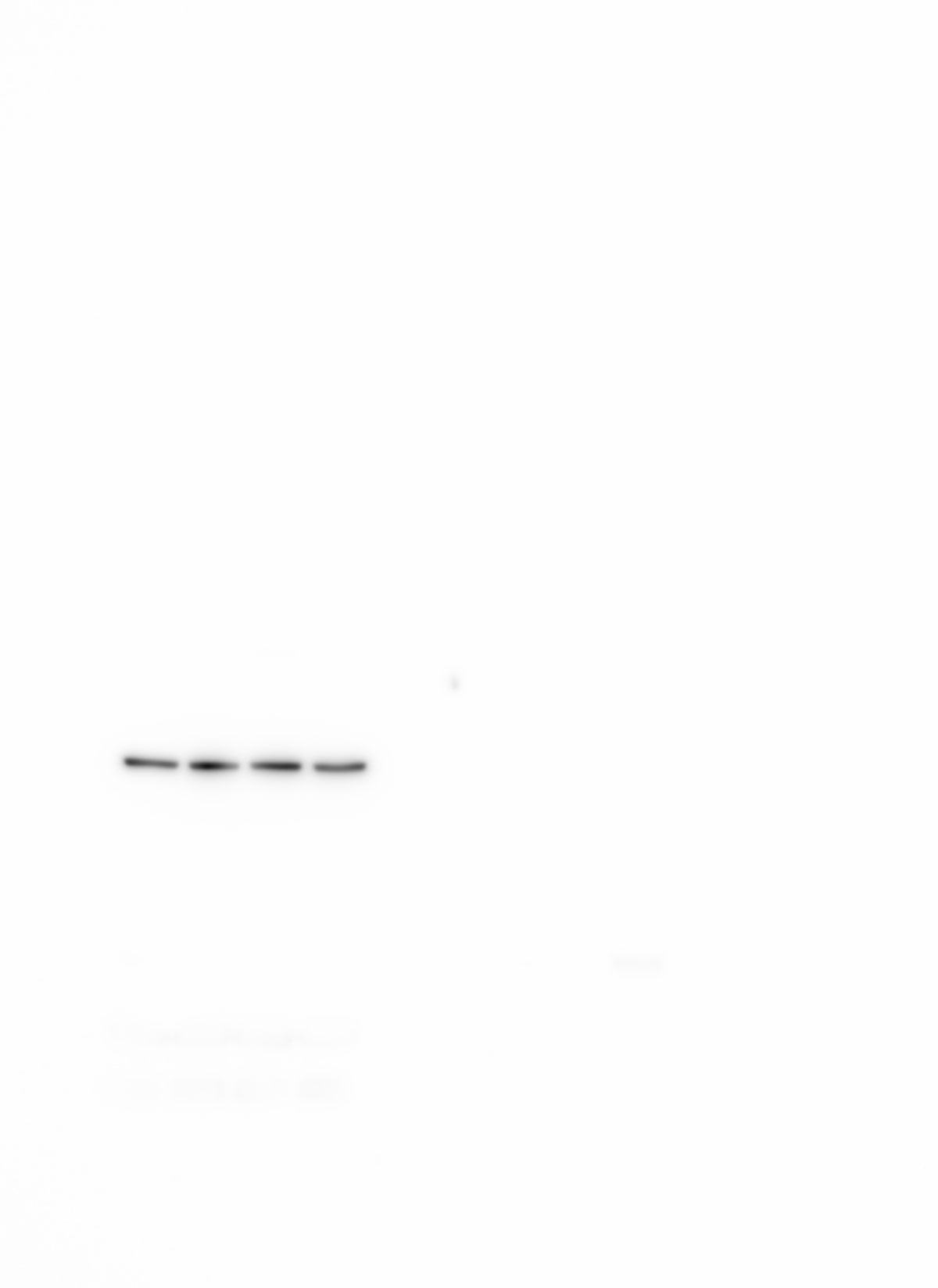

Supplement: Supplementary file 11 — Source data for Appendix [file 44318_2024_305_MOESM11_ESM.zip › Appendix/Appendix Figure S8/S8D/Calnexin for Cathepsin L Cathepsin C 16bit original 20240409_130603-10_Ch_Chemi.tif]

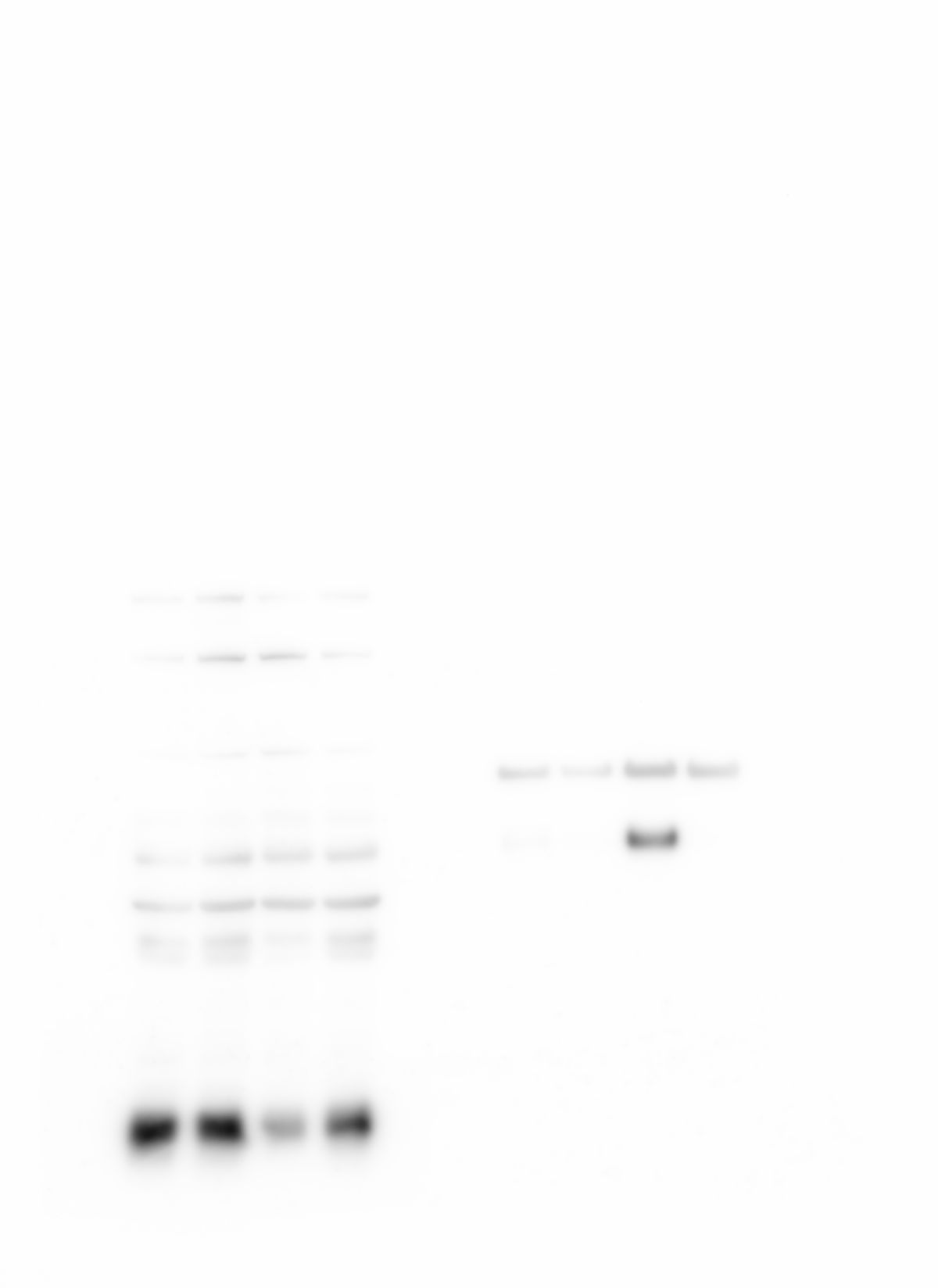

Supplement: Supplementary file 11 — Source data for Appendix [file 44318_2024_305_MOESM11_ESM.zip › Appendix/Appendix Figure S8/S8D/Cathepsin C 16bit original 20240306_143538-07_Ch_Chemi.tif]

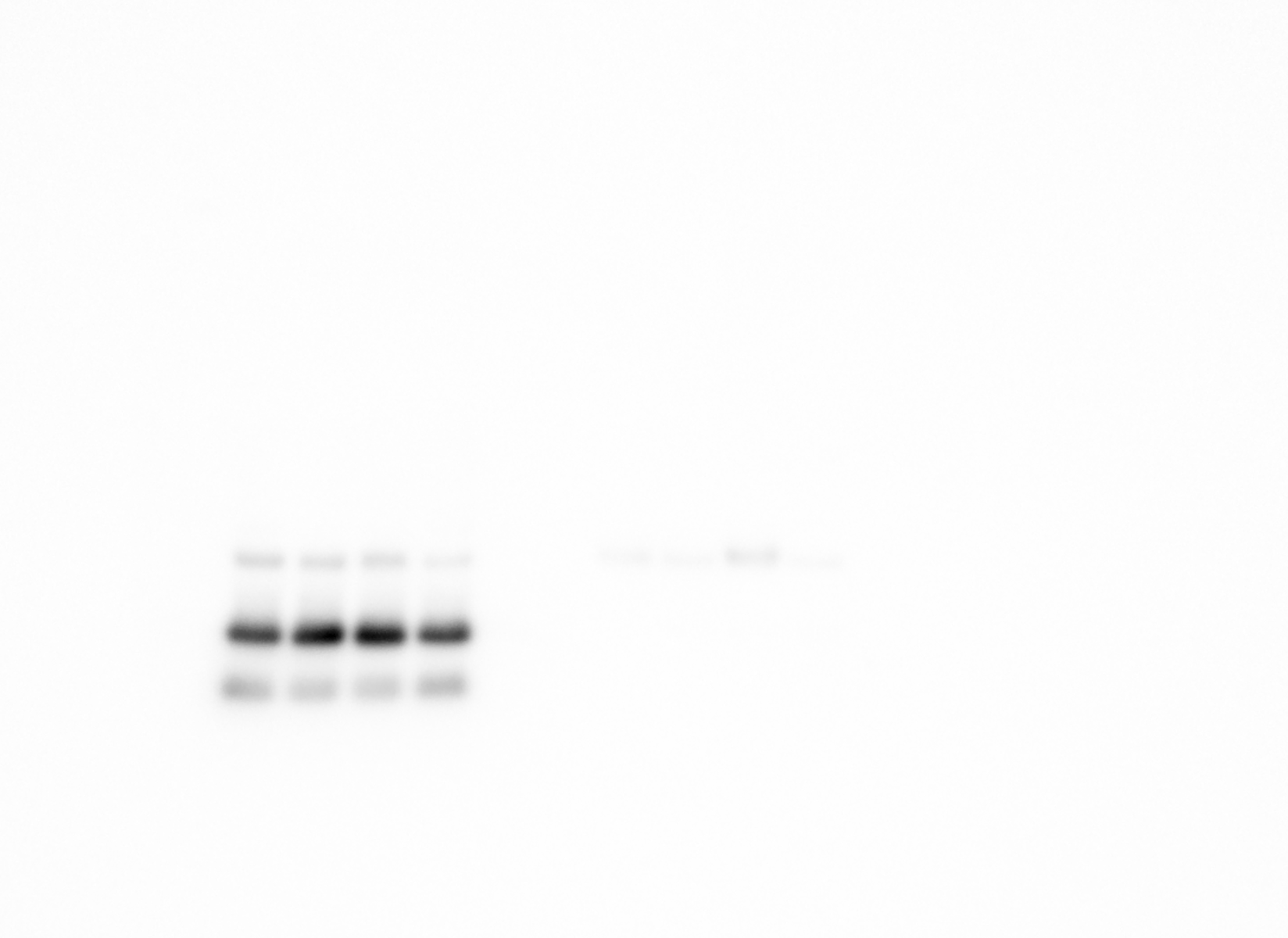

Supplement: Supplementary file 11 — Source data for Appendix [file 44318_2024_305_MOESM11_ESM.zip › Appendix/Appendix Figure S8/S8D/Cathepsin L short exposure 16bit original 20240307_133249_Ch_Chemi.tif]

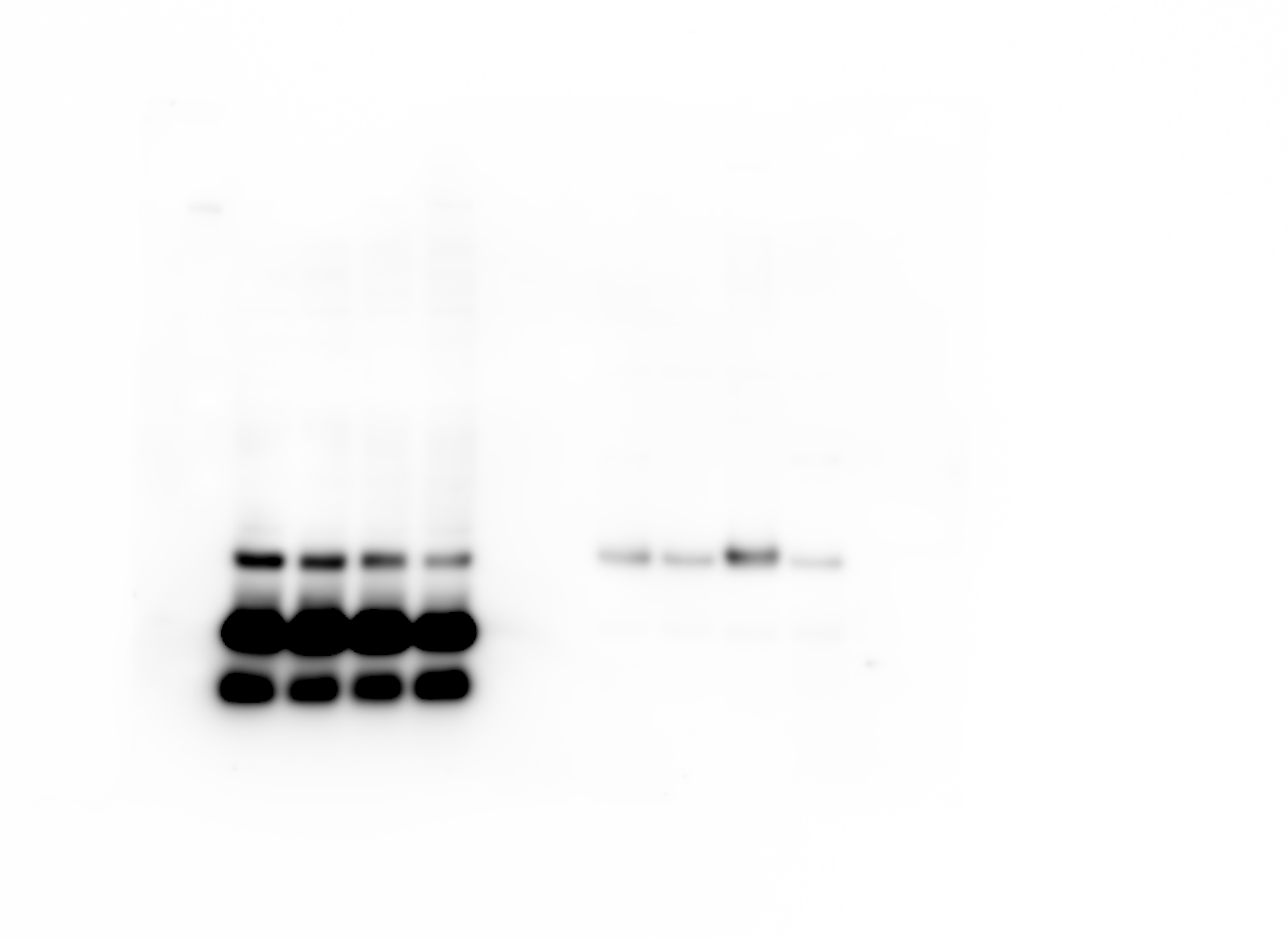

Supplement: Supplementary file 11 — Source data for Appendix [file 44318_2024_305_MOESM11_ESM.zip › Appendix/Appendix Figure S8/S8D/Cathepsin L long exposure 16bit original 20240307_133415-02_Ch_Chemi.tif]

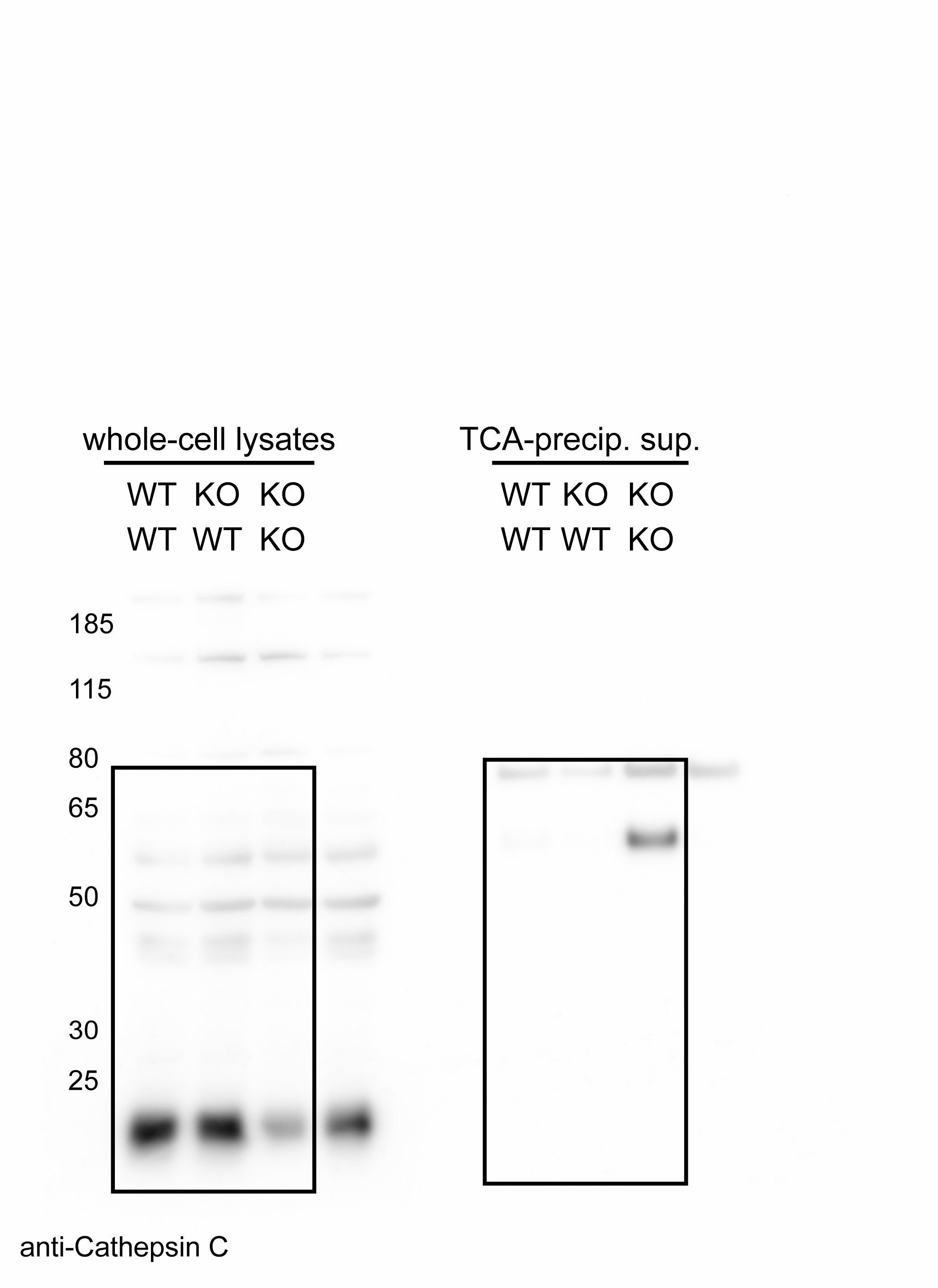

Supplement: Supplementary file 11 — Source data for Appendix [file 44318_2024_305_MOESM11_ESM.zip › Appendix/Appendix Figure S8/S8D/Cathepsin C 8bit annotated 20240306_143538-07_Ch_Chemi-01.tif]

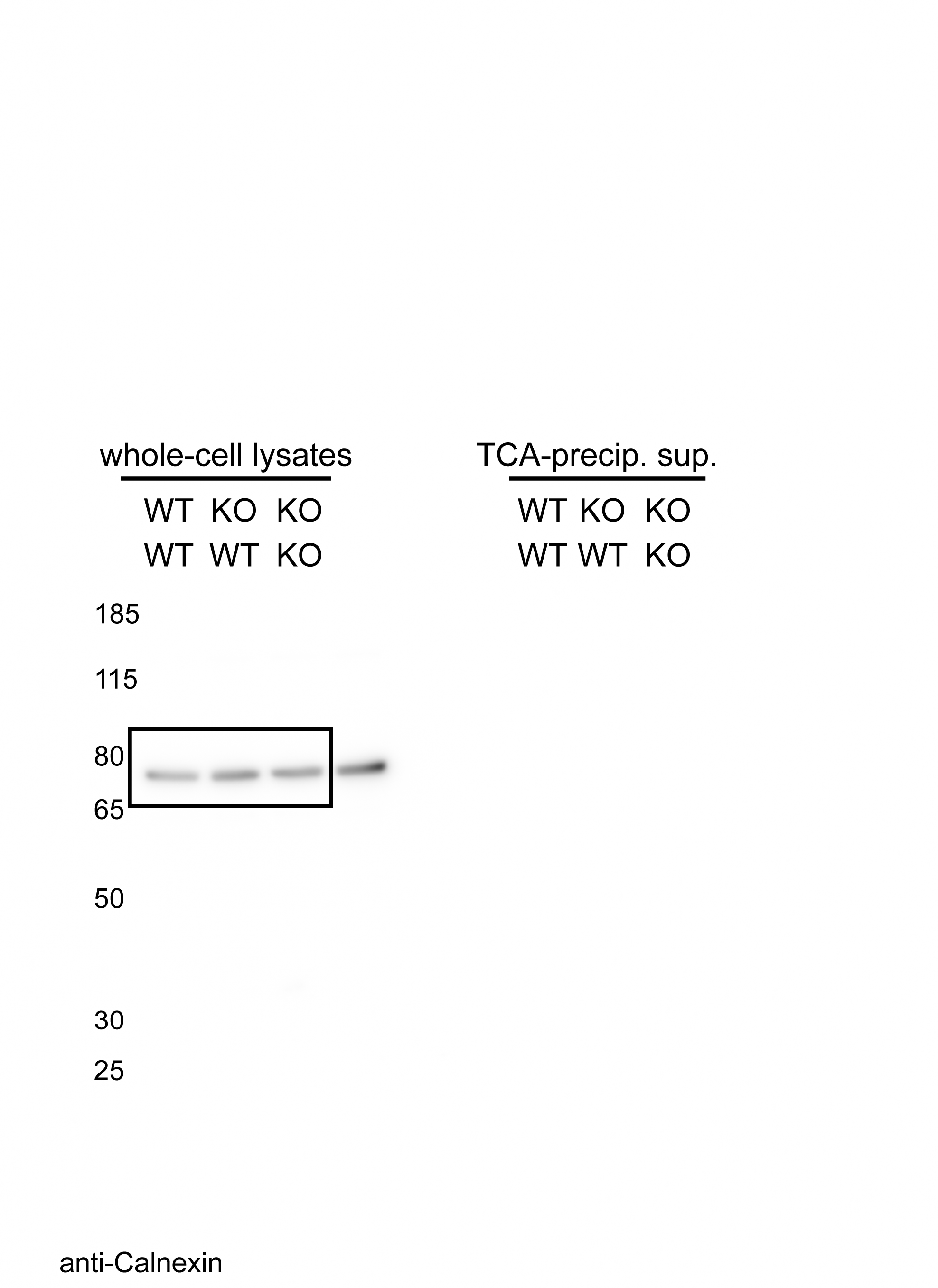

Supplement: Supplementary file 11 — Source data for Appendix [file 44318_2024_305_MOESM11_ESM.zip › Appendix/Appendix Figure S8/S8D/Calnexin for Hex 8bit annotated 20240409_134520-02_Ch_Chemi-01.tif]

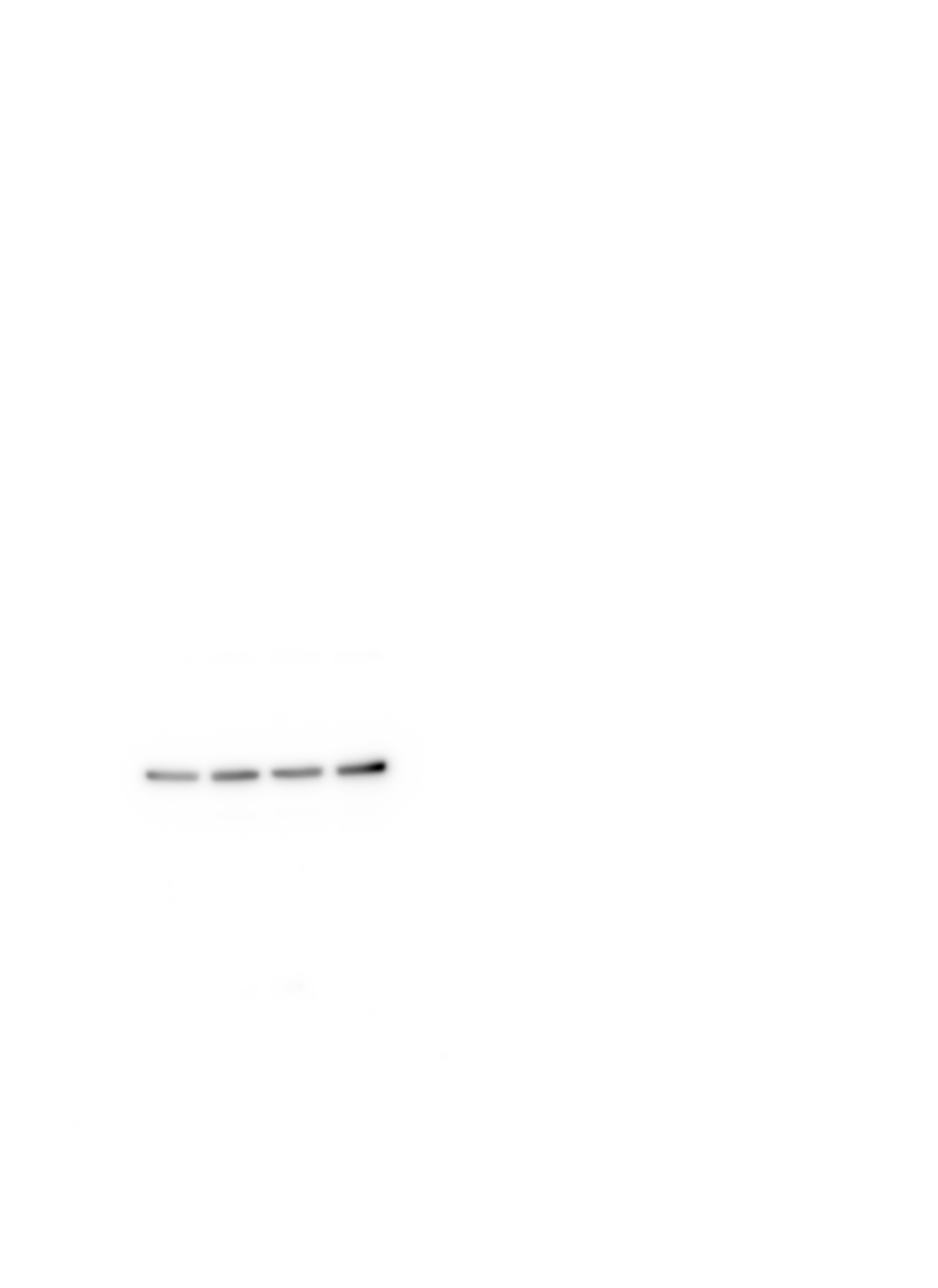

Supplement: Supplementary file 11 — Source data for Appendix [file 44318_2024_305_MOESM11_ESM.zip › Appendix/Appendix Figure S8/S8D/Calnexin for HexB 16bit original 20240409_134520-02_Ch_Chemi.tif]

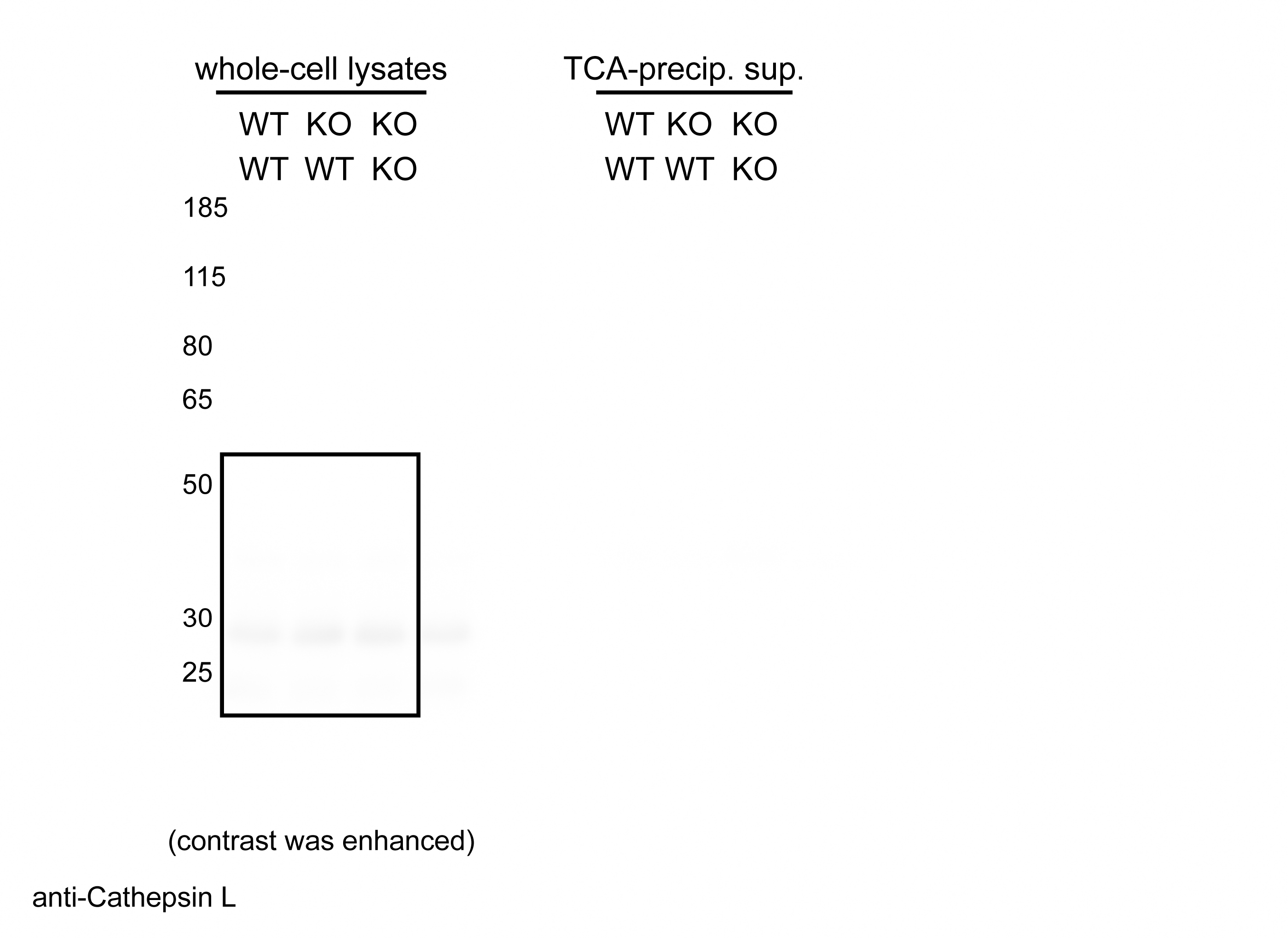

Supplement: Supplementary file 11 — Source data for Appendix [file 44318_2024_305_MOESM11_ESM.zip › Appendix/Appendix Figure S8/S8D/Cathepsin L short exposure 8bit annotated 20240307_133249_Ch_Chemi-01.tif]

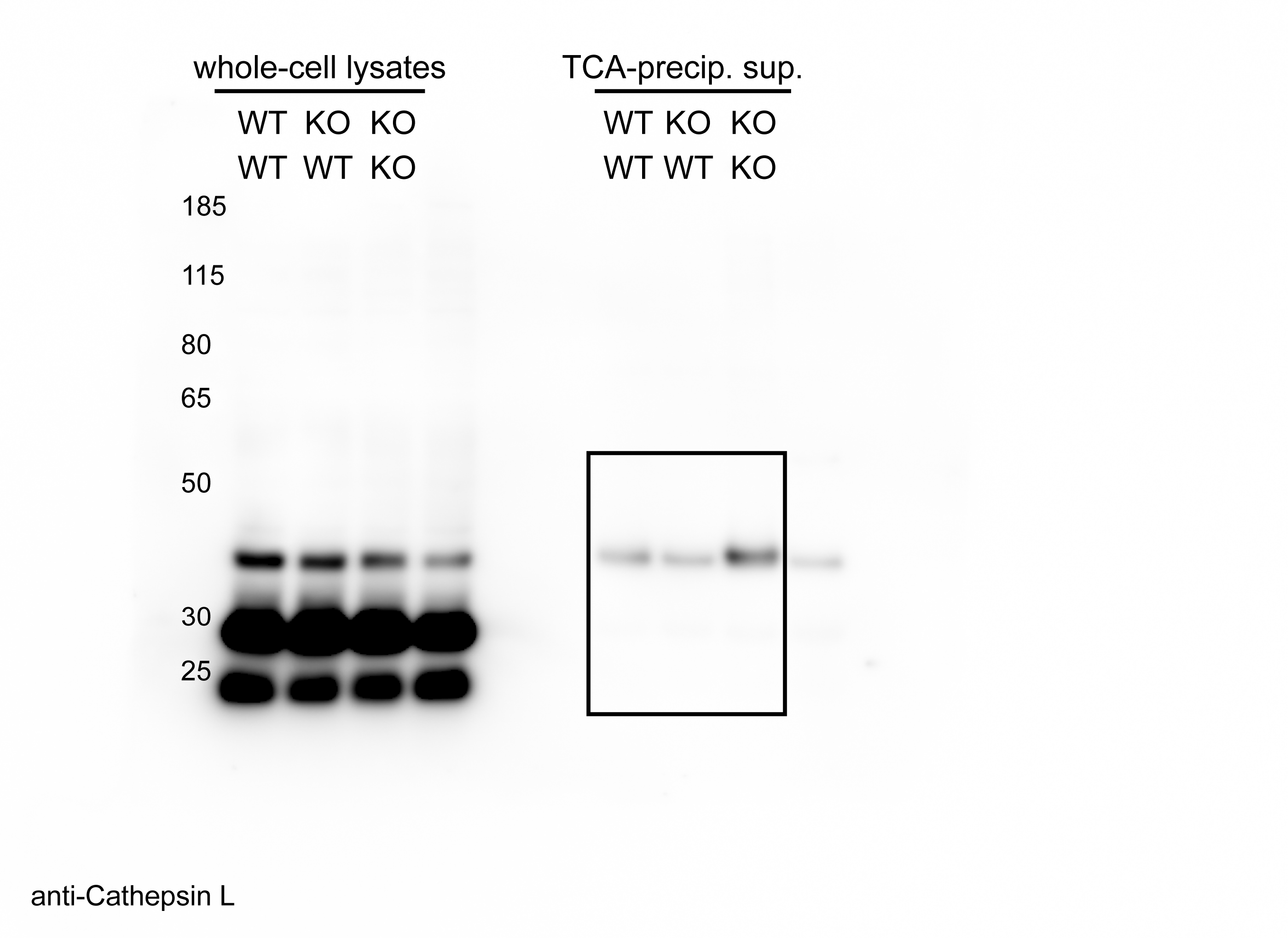

Supplement: Supplementary file 11 — Source data for Appendix [file 44318_2024_305_MOESM11_ESM.zip › Appendix/Appendix Figure S8/S8D/Cathepsin L long exposure 8bit annotated 20240307_133415-02_Ch_Chemi-01.tif]

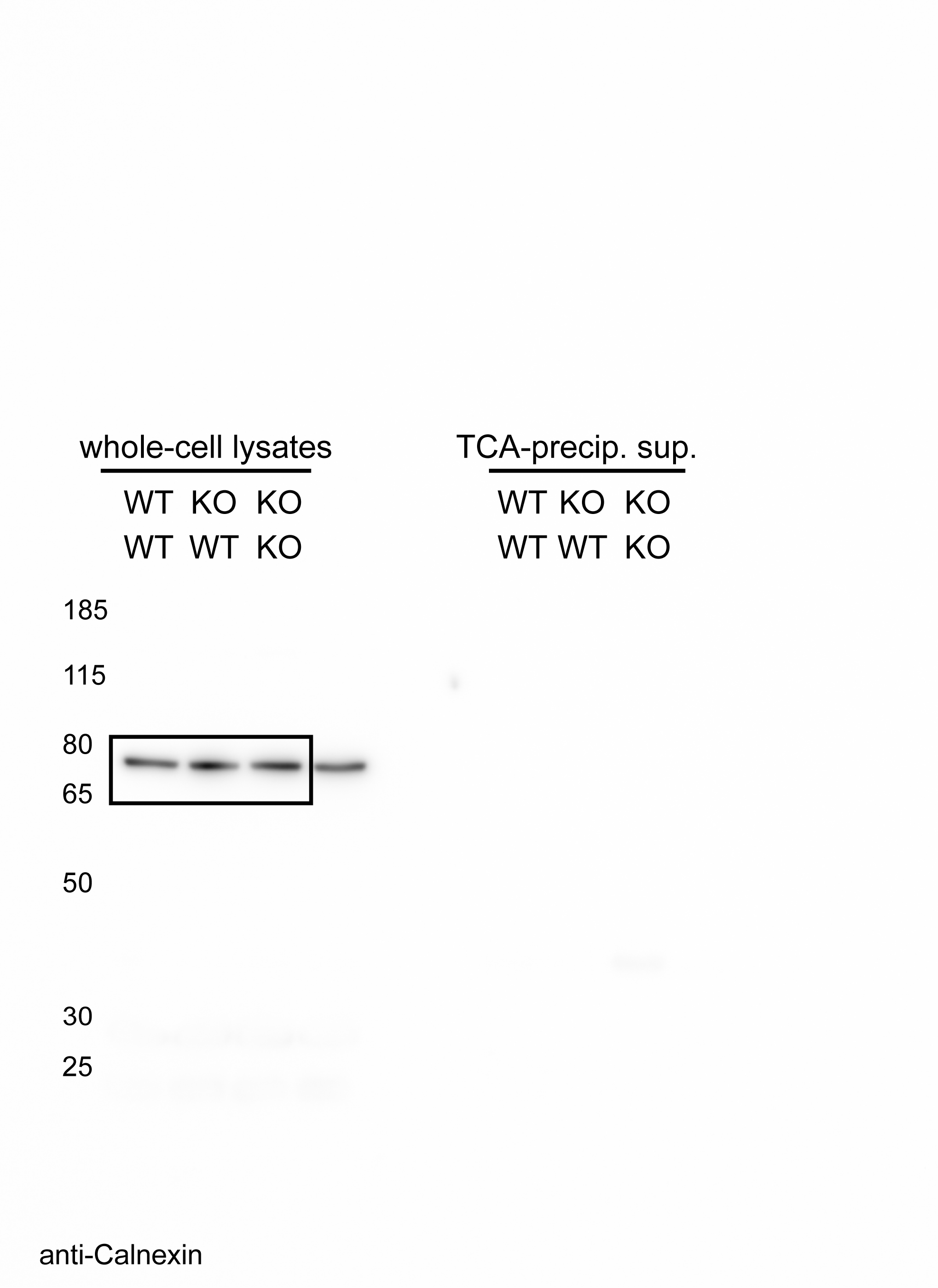

Supplement: Supplementary file 11 — Source data for Appendix [file 44318_2024_305_MOESM11_ESM.zip › Appendix/Appendix Figure S8/S8D/Calnexin for Cathepsin L Cathepsin C 8bit annotated 20240409_130603-10_Ch_Chemi-01.tif]

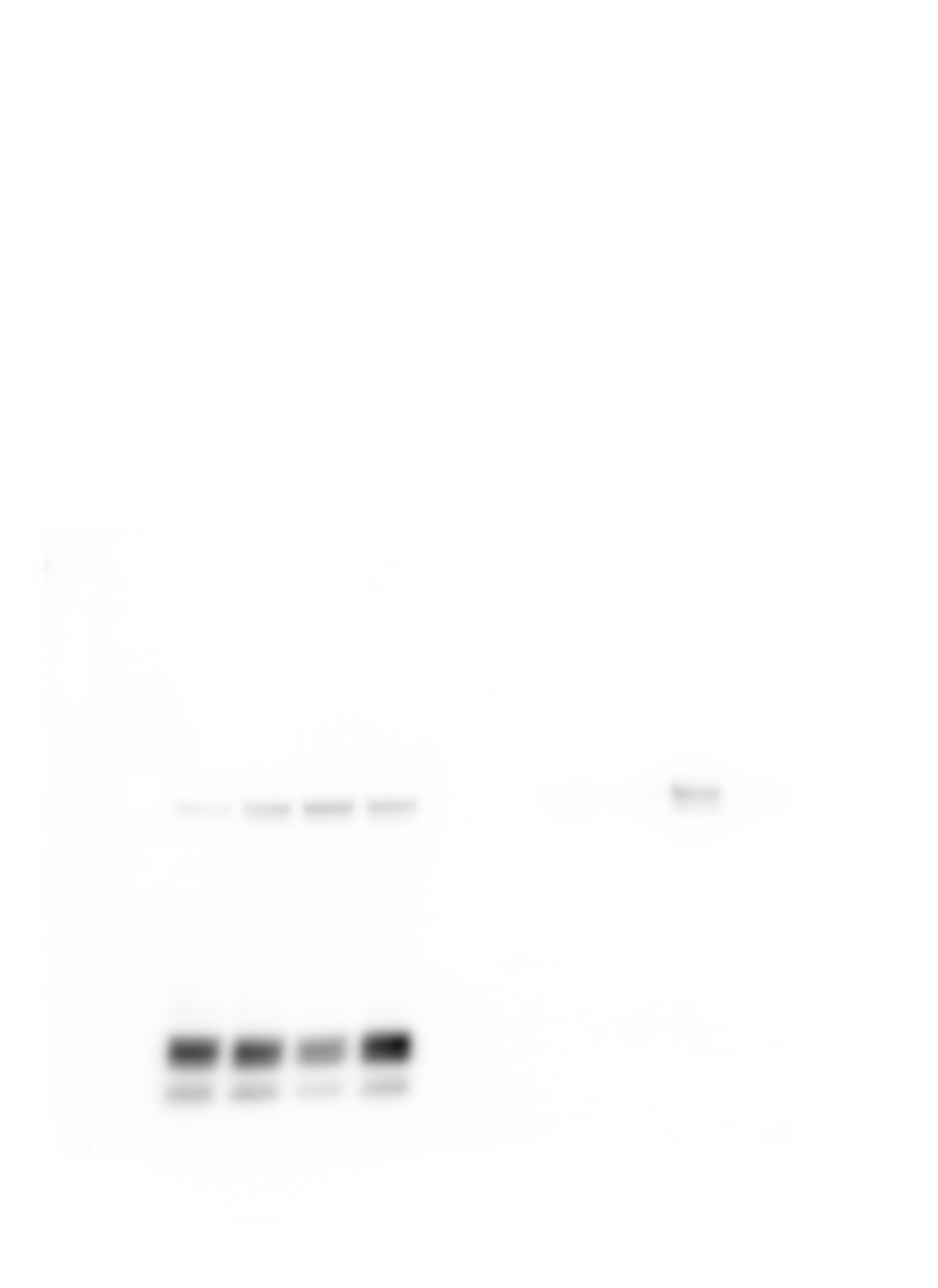

Supplement: Supplementary file 11 — Source data for Appendix [file 44318_2024_305_MOESM11_ESM.zip › Appendix/Appendix Figure S8/S8D/Hex B 16bit original 20240306_142708-02_Ch_Chemi.tif]

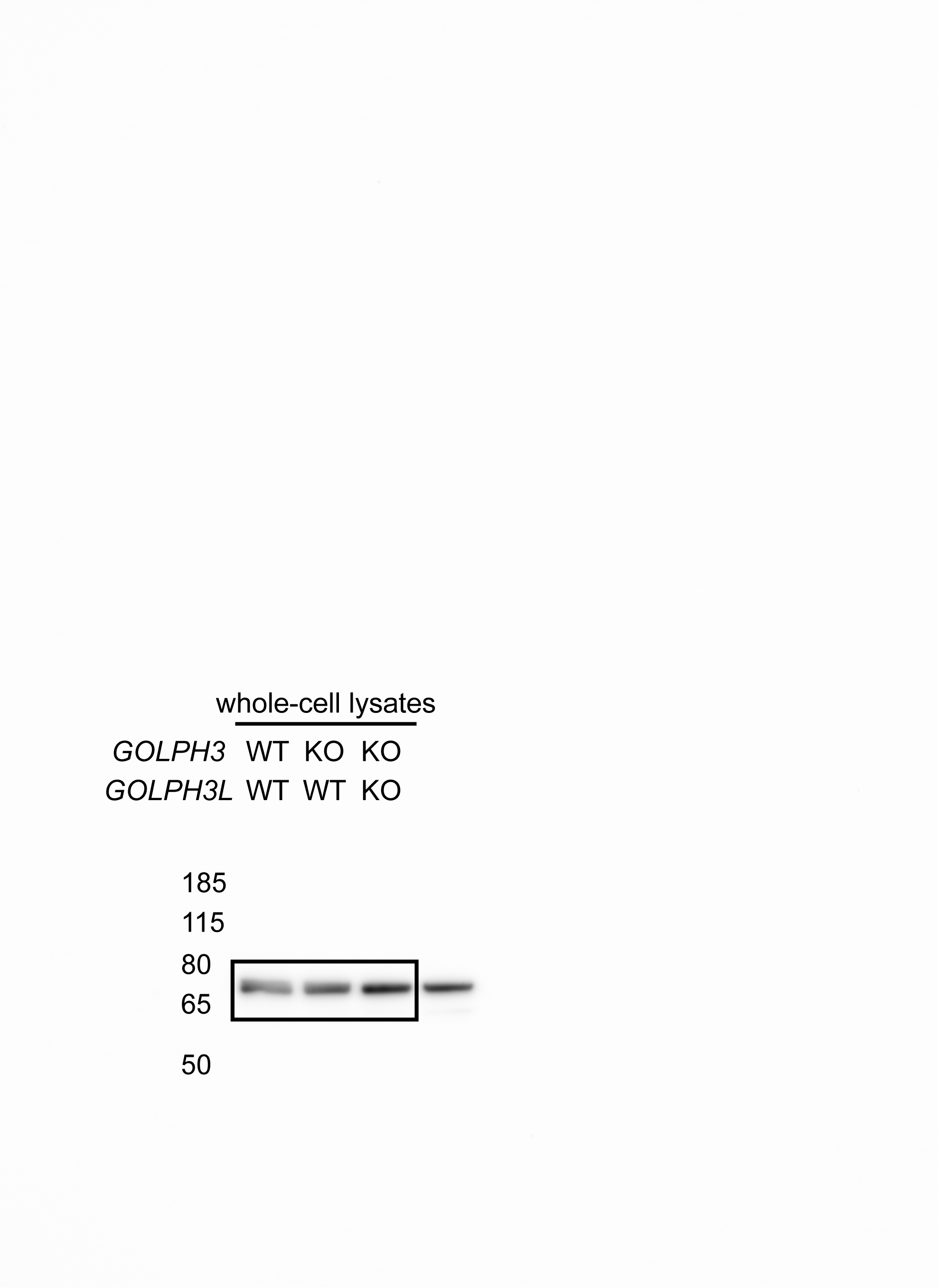

Supplement: Supplementary file 11 — Source data for Appendix [file 44318_2024_305_MOESM11_ESM.zip › Appendix/Appendix Figure S8/S8C/Calnexin for LYSET 8bit annotated 20240312_145536-20_Ch_Chemi-01.tif]

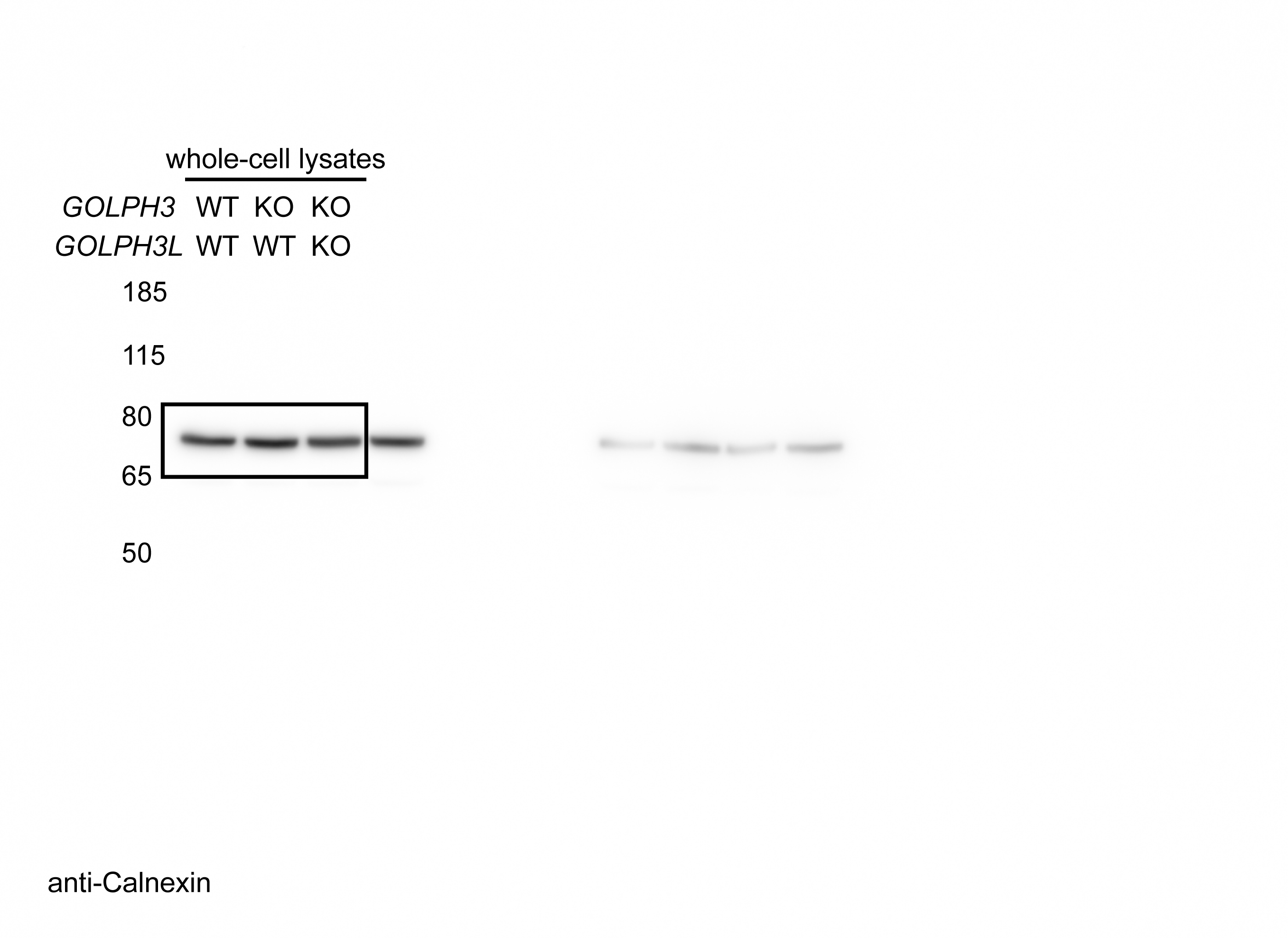

Supplement: Supplementary file 11 — Source data for Appendix [file 44318_2024_305_MOESM11_ESM.zip › Appendix/Appendix Figure S8/S8C/Calnexin for GOLPH3 (Abcam) 8bit annotated 20240306_150859-03_Ch_Chemi-01.tif]

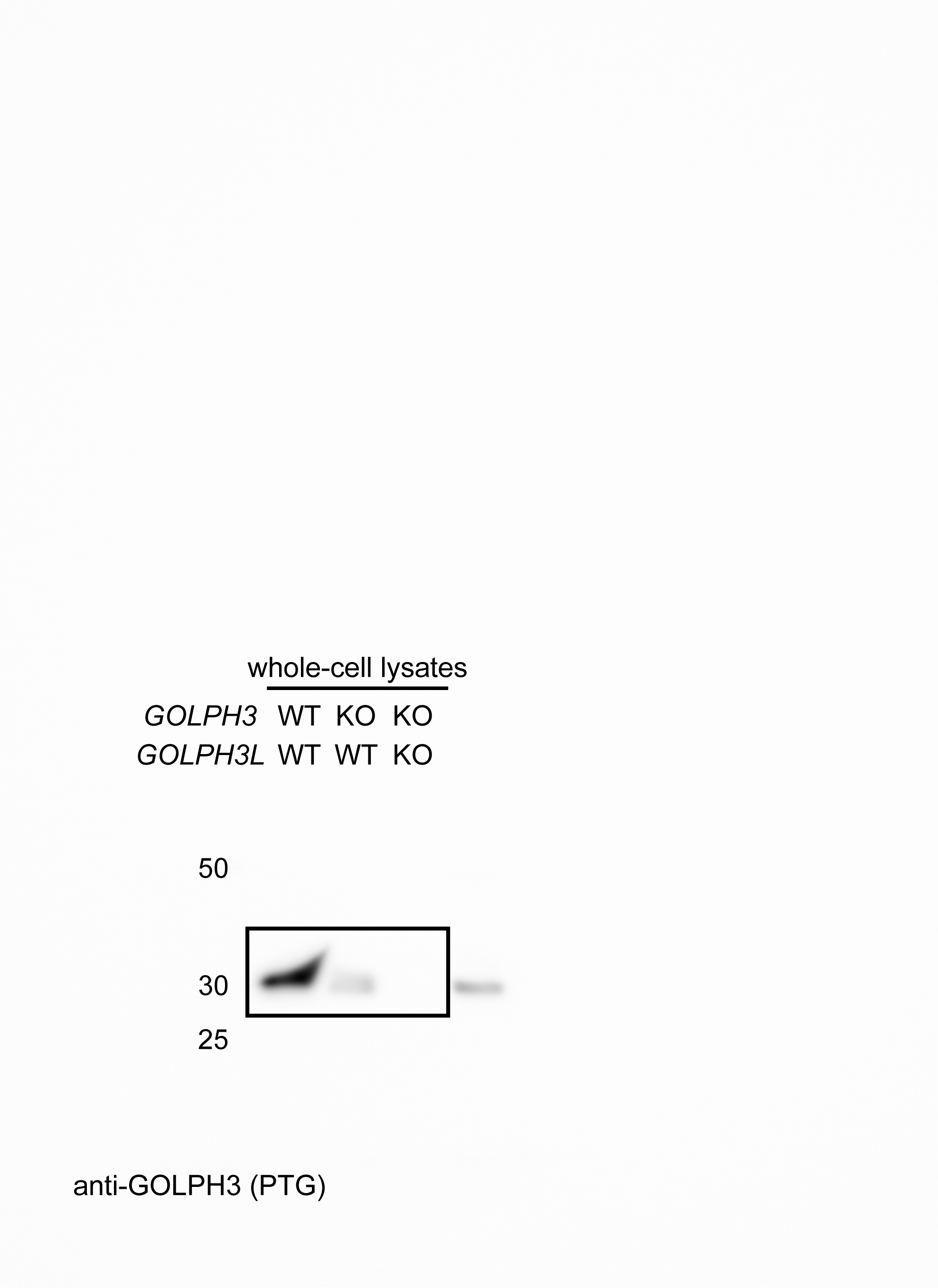

Supplement: Supplementary file 11 — Source data for Appendix [file 44318_2024_305_MOESM11_ESM.zip › Appendix/Appendix Figure S8/S8C/GOLPH3 (PTG) 8bit annotated 20240306_135440-18_Ch_Chemi-01.tif]

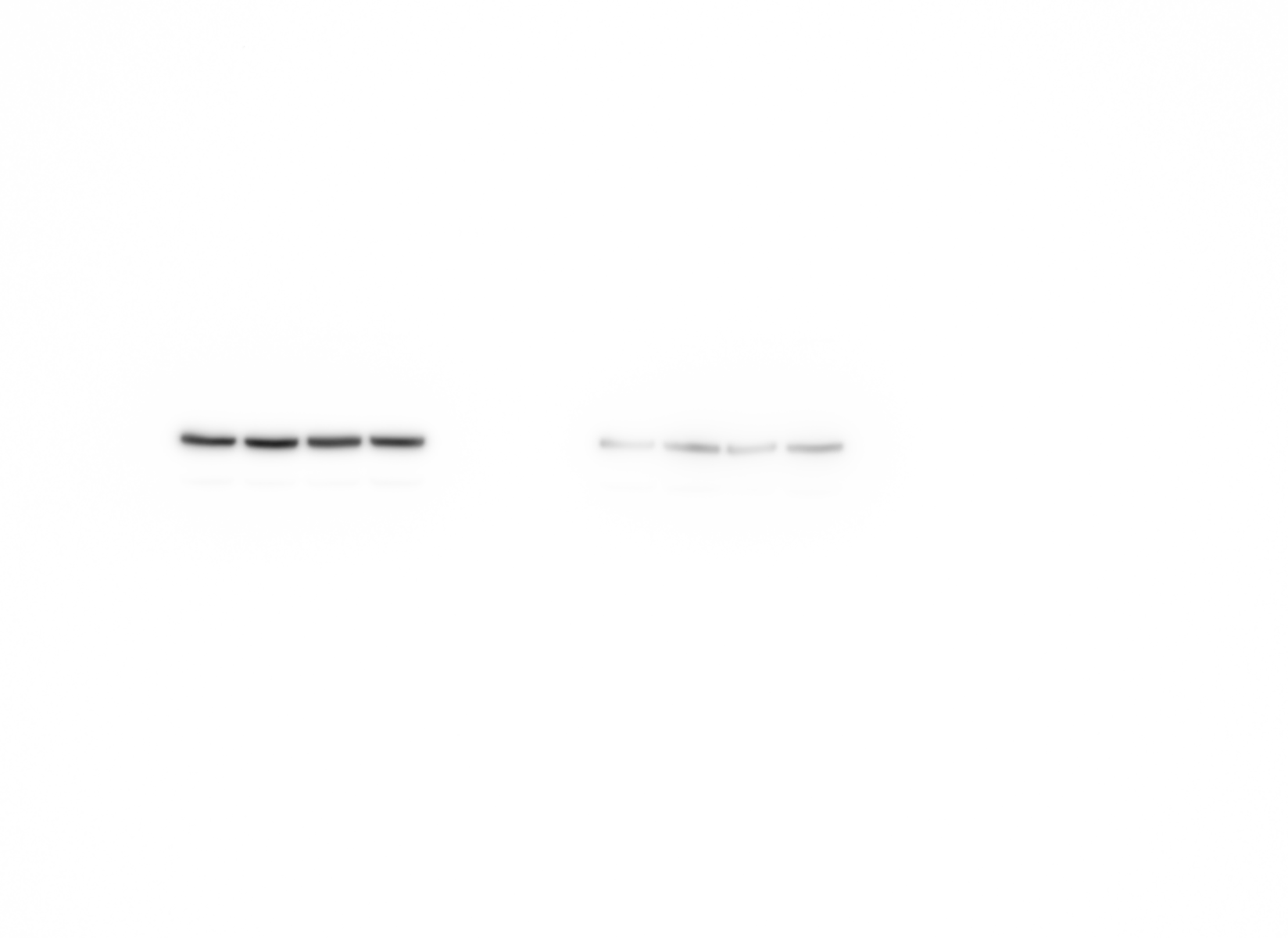

Supplement: Supplementary file 11 — Source data for Appendix [file 44318_2024_305_MOESM11_ESM.zip › Appendix/Appendix Figure S8/S8C/Calnexin for GOLPH3 (Abcam) 16bit original 20240306_150859-03_Ch_Chemi.tif]

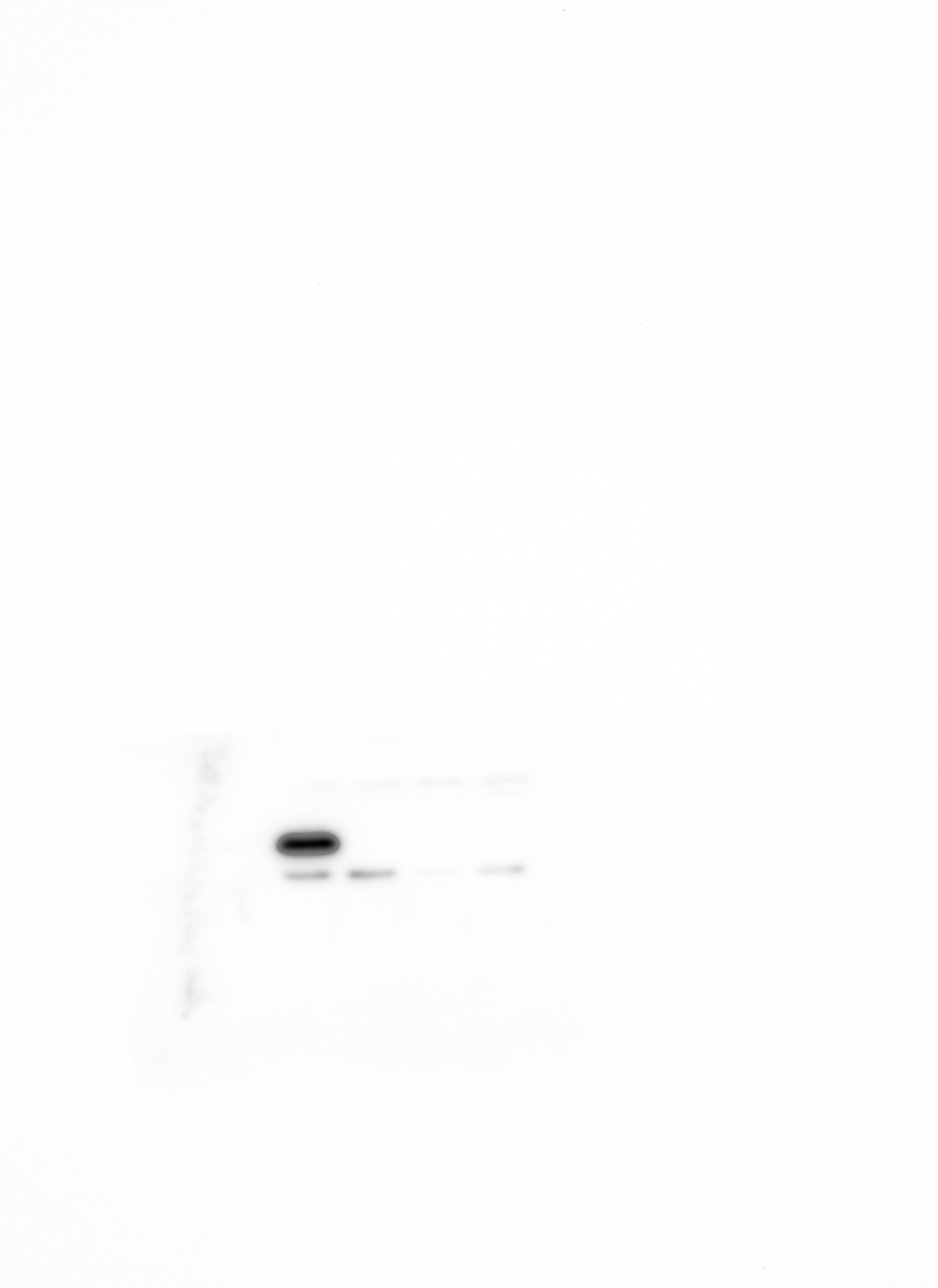

Supplement: Supplementary file 11 — Source data for Appendix [file 44318_2024_305_MOESM11_ESM.zip › Appendix/Appendix Figure S8/S8C/GOLPH3 (Abcam) 16bit original 20240306_144509-07_Ch_Chemi.tif]

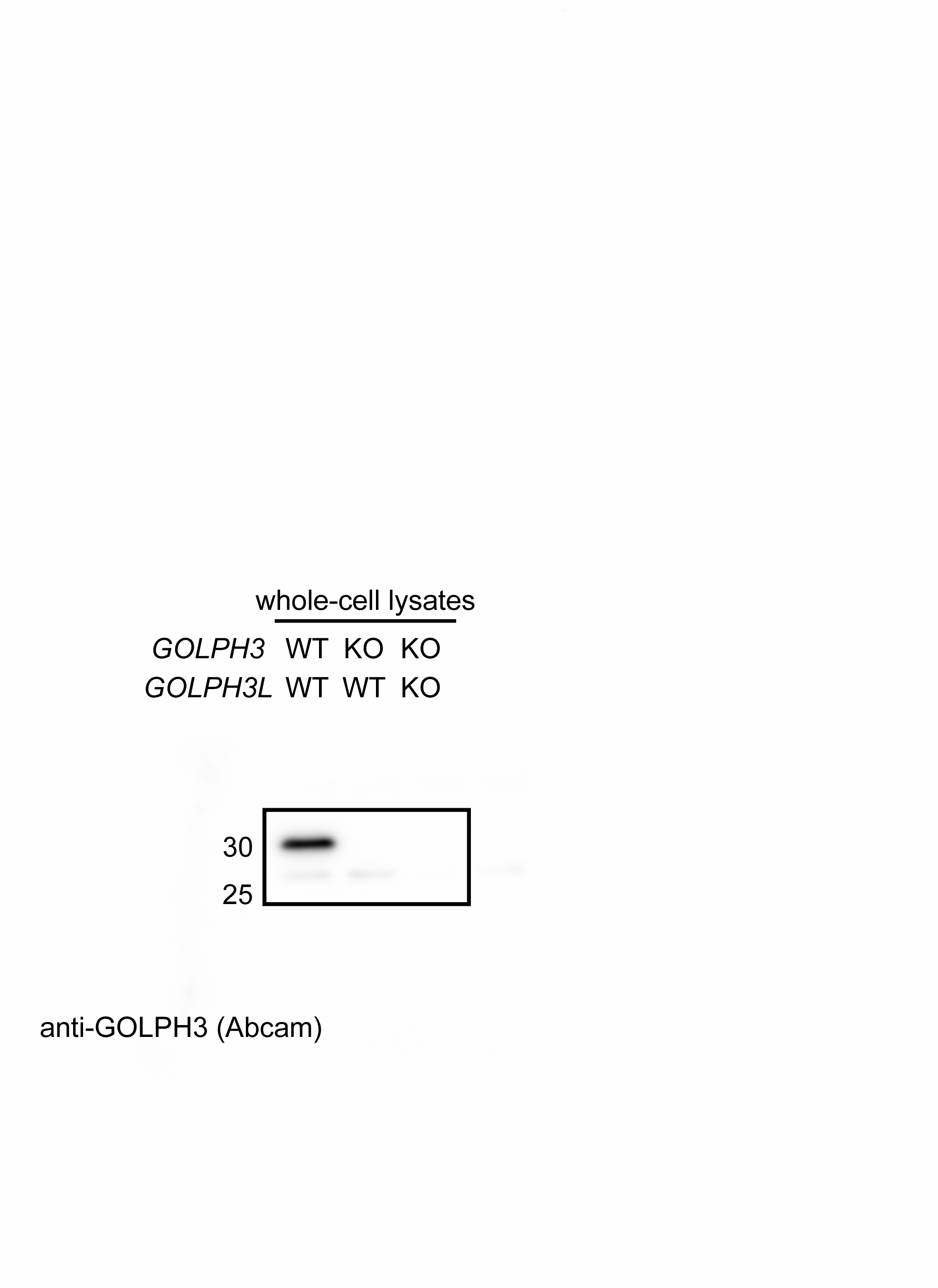

Supplement: Supplementary file 11 — Source data for Appendix [file 44318_2024_305_MOESM11_ESM.zip › Appendix/Appendix Figure S8/S8C/GOLPH3 (Abcam) 8bit annotated 20240306_144509-07_Ch_Chemi-01.tif]

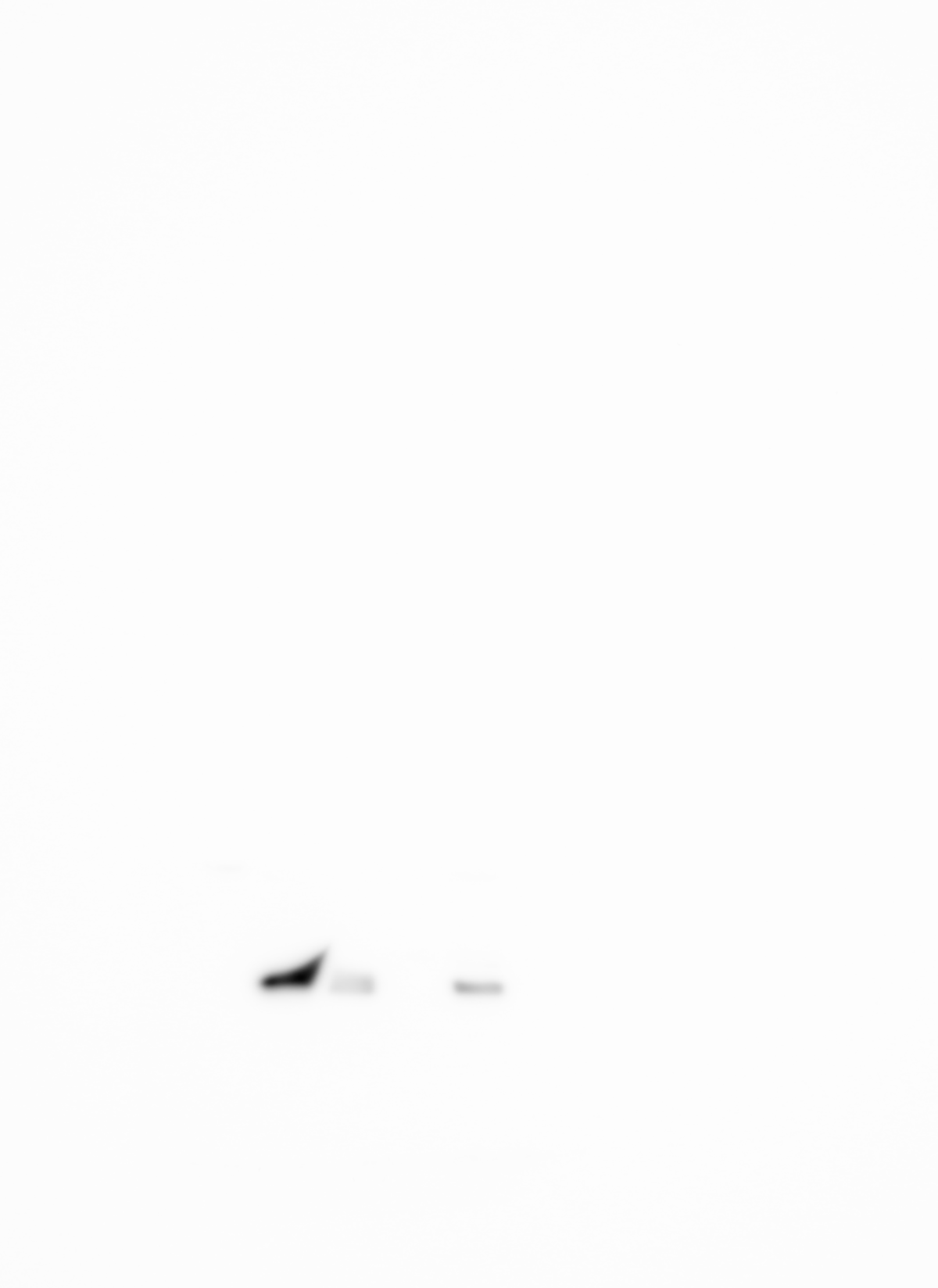

Supplement: Supplementary file 11 — Source data for Appendix [file 44318_2024_305_MOESM11_ESM.zip › Appendix/Appendix Figure S8/S8C/GOLPH3 (PTG) 16bit original 20240306_135440-18_Ch_Chemi.tif]

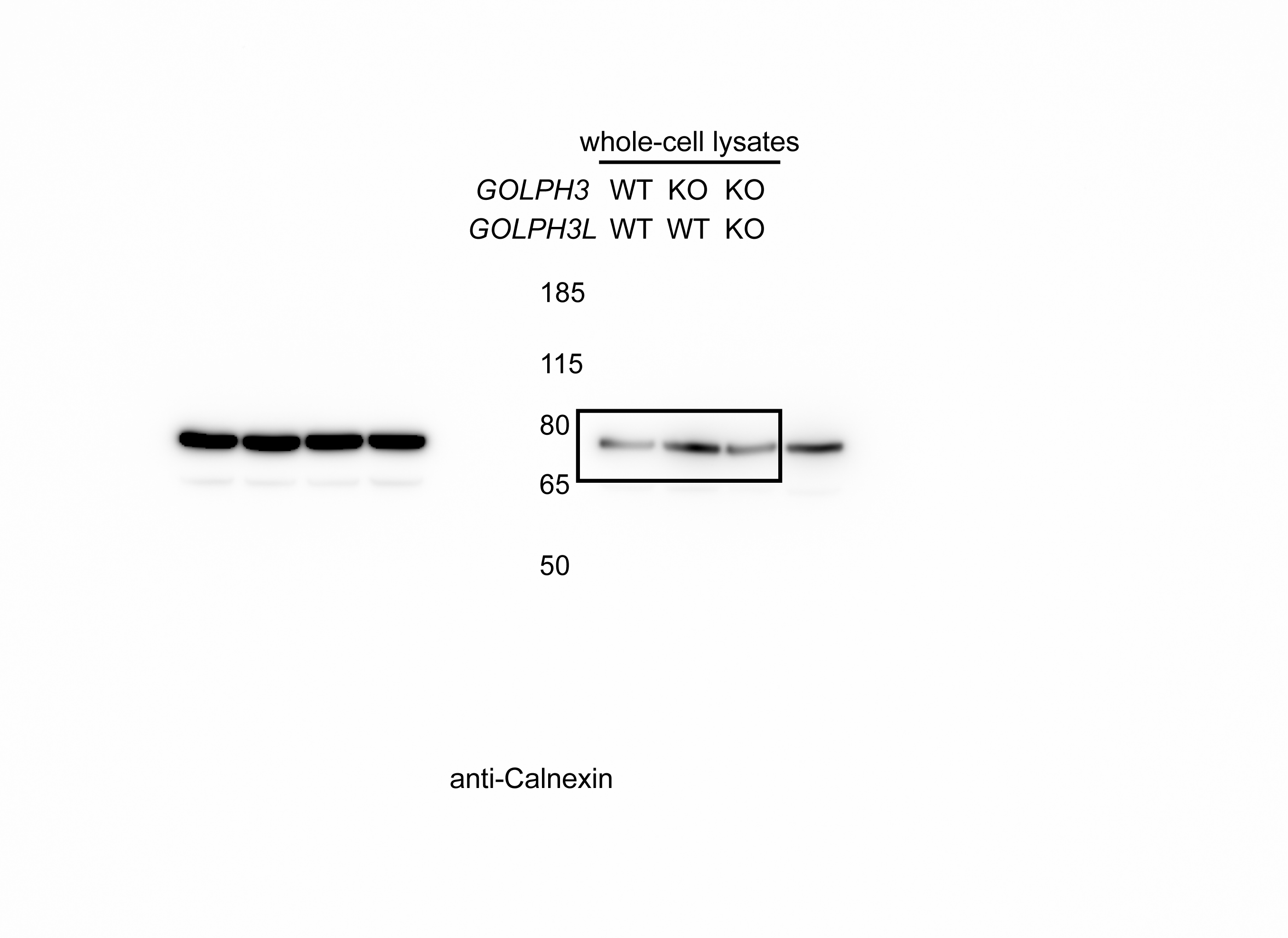

Supplement: Supplementary file 11 — Source data for Appendix [file 44318_2024_305_MOESM11_ESM.zip › Appendix/Appendix Figure S8/S8C/Calnexin for GOLPH3 (PTG) 8bit annotated 20240306_150859-10_Ch_Chemi-01-01.tif]

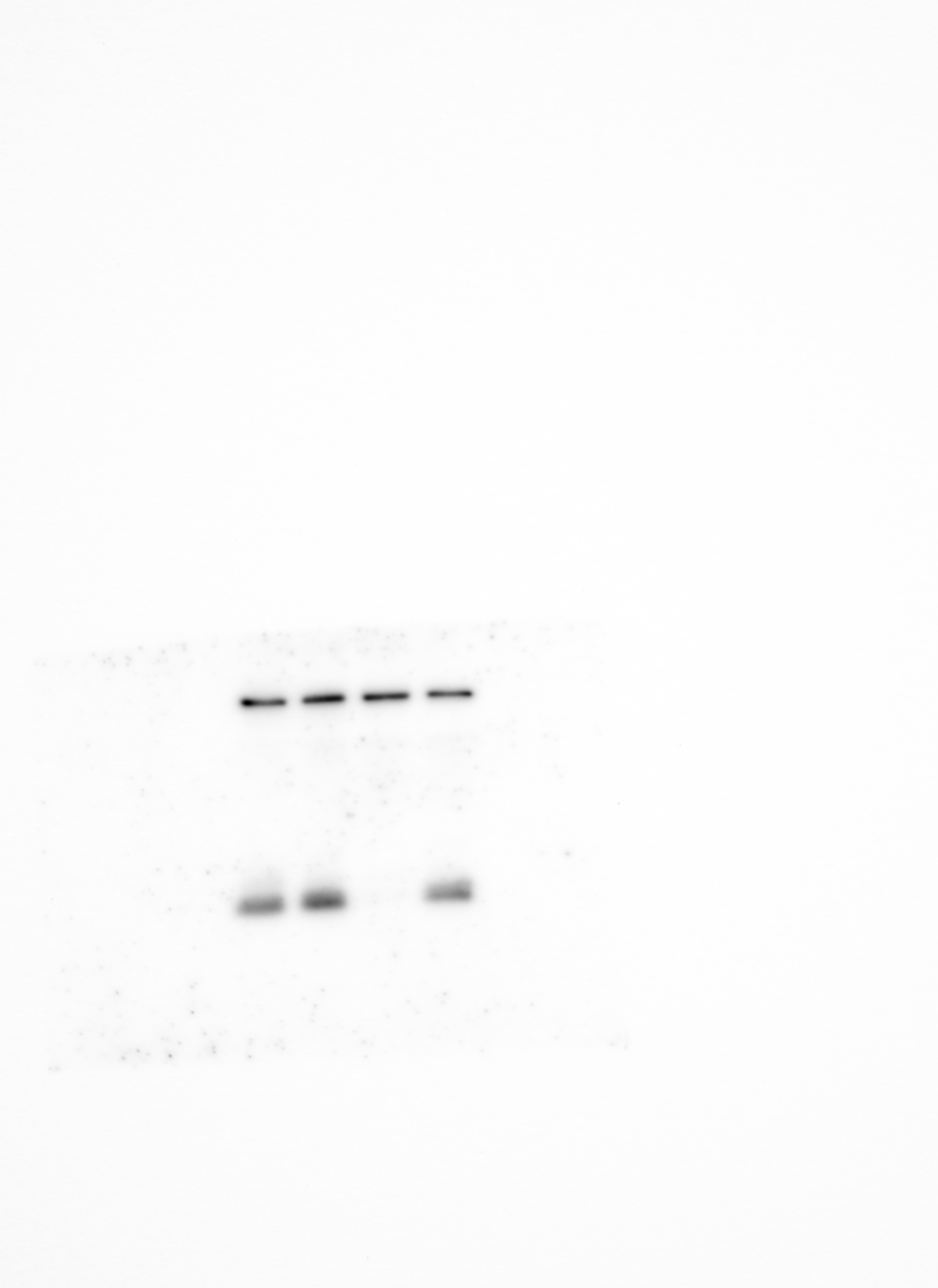

Supplement: Supplementary file 11 — Source data for Appendix [file 44318_2024_305_MOESM11_ESM.zip › Appendix/Appendix Figure S8/S8C/LYSET original 16bit 20240312_143509-50_Ch_Chemi.tif]

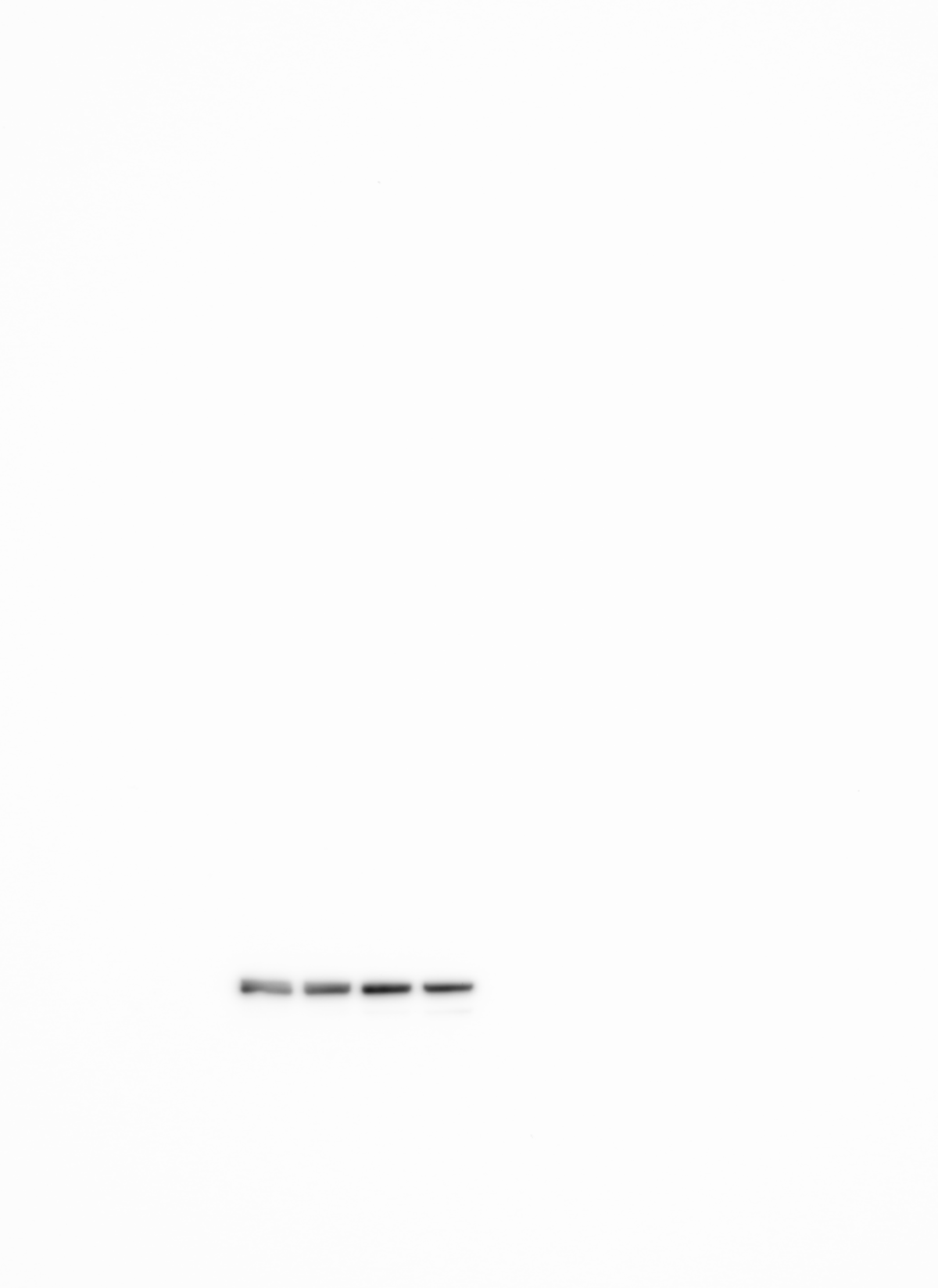

Supplement: Supplementary file 11 — Source data for Appendix [file 44318_2024_305_MOESM11_ESM.zip › Appendix/Appendix Figure S8/S8C/Calnexin for LYSET 16bit original 20240312_145536-20_Ch_Chemi.tif]

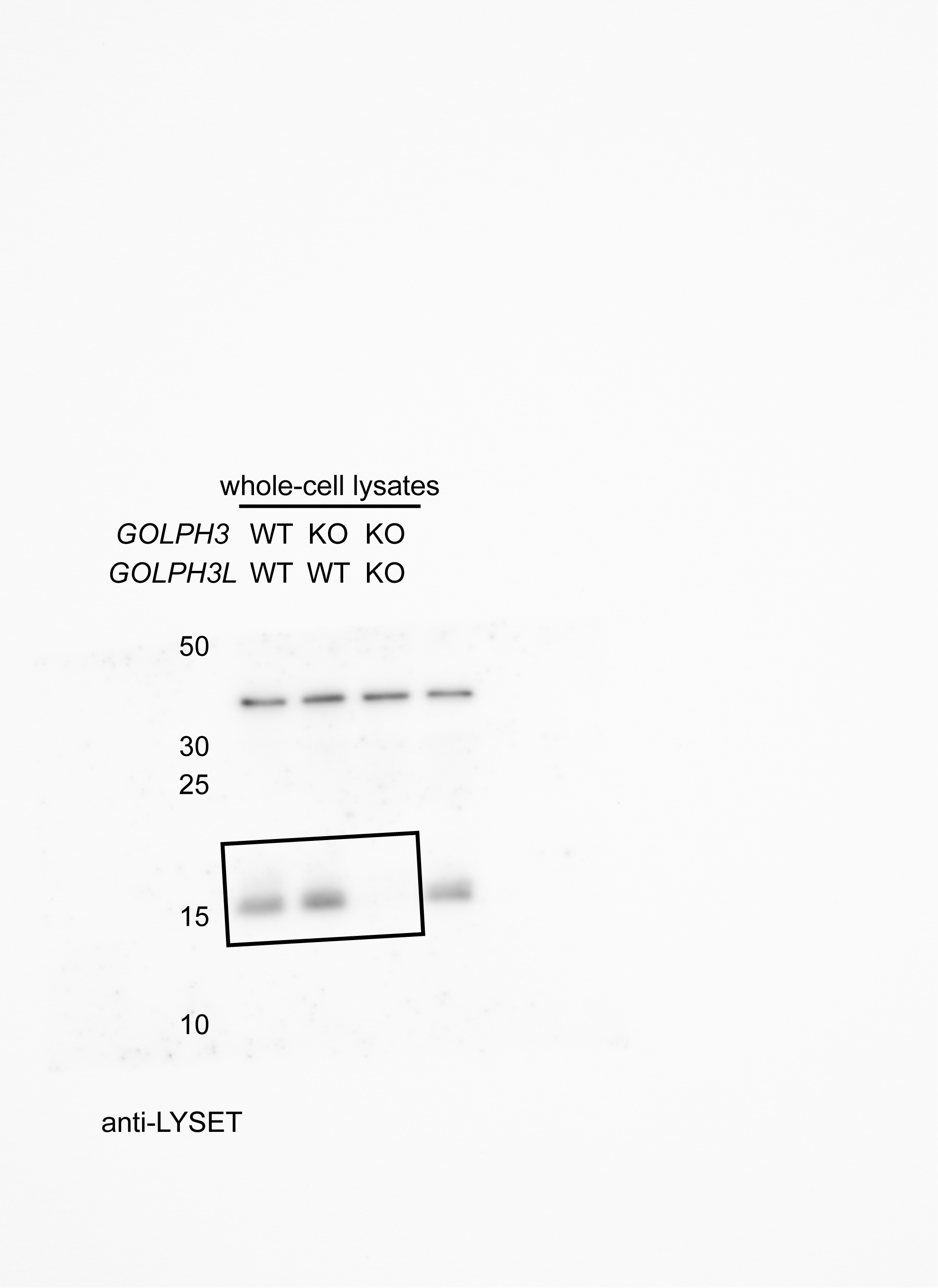

Supplement: Supplementary file 11 — Source data for Appendix [file 44318_2024_305_MOESM11_ESM.zip › Appendix/Appendix Figure S8/S8C/LYSET 8bit annotation 20240312_143509-50_Ch_Chemi-01-01.tif]

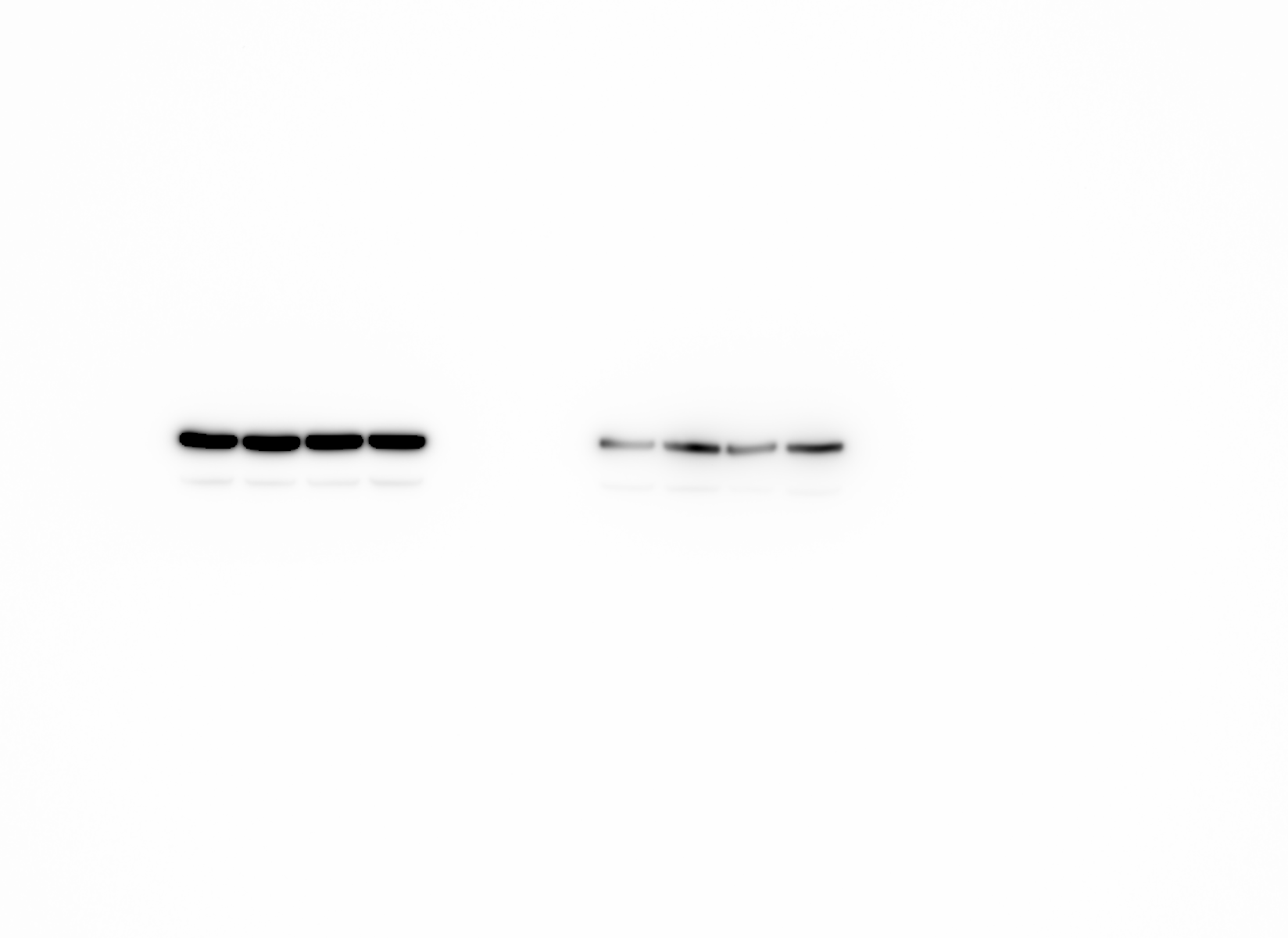

Supplement: Supplementary file 11 — Source data for Appendix [file 44318_2024_305_MOESM11_ESM.zip › Appendix/Appendix Figure S8/S8C/Calnexin for GOLPH3 (PTG) 16bit original 20240306_150859-10_Ch_Chemi.tif]

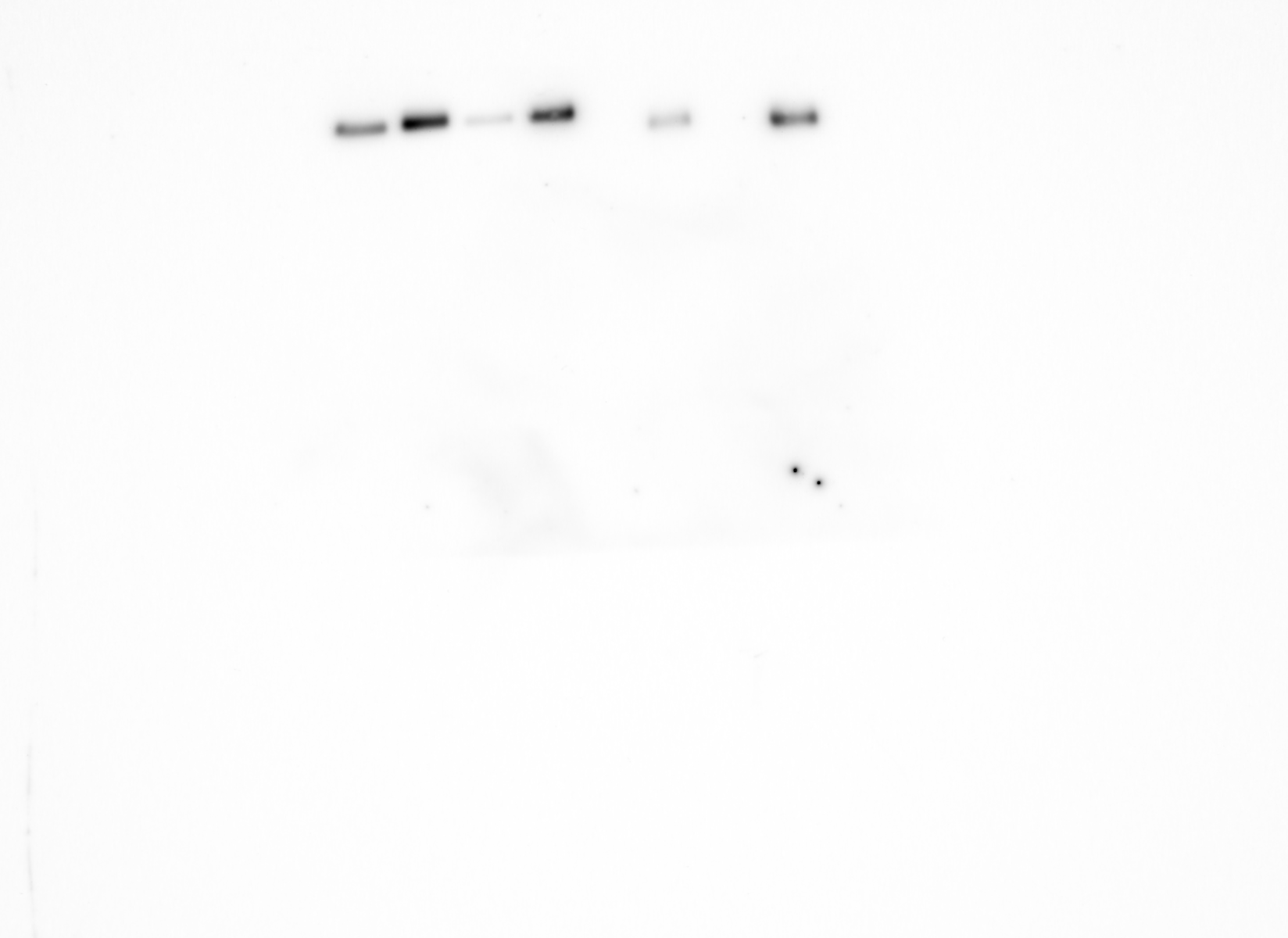

Supplement: Supplementary file 11 — Source data for Appendix [file 44318_2024_305_MOESM11_ESM.zip › Appendix/Appendix Figure S1/S1D/B4GALT1 16bit original 20240206_171041-50_Ch_Chemi.tif]

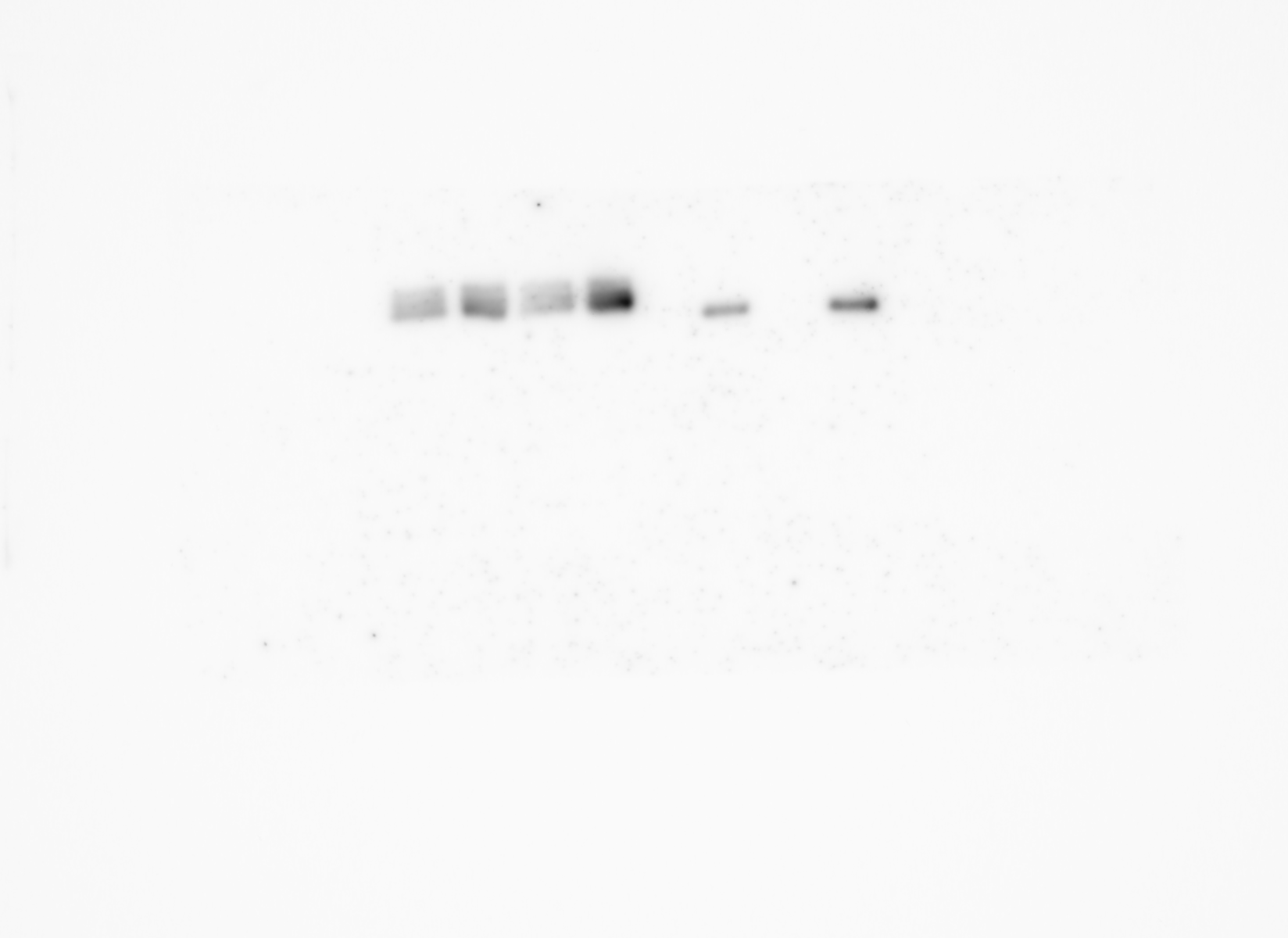

Supplement: Supplementary file 11 — Source data for Appendix [file 44318_2024_305_MOESM11_ESM.zip › Appendix/Appendix Figure S1/S1D/CANT1 16bit original 20240213_161512-48_Ch_Chemi.tif]

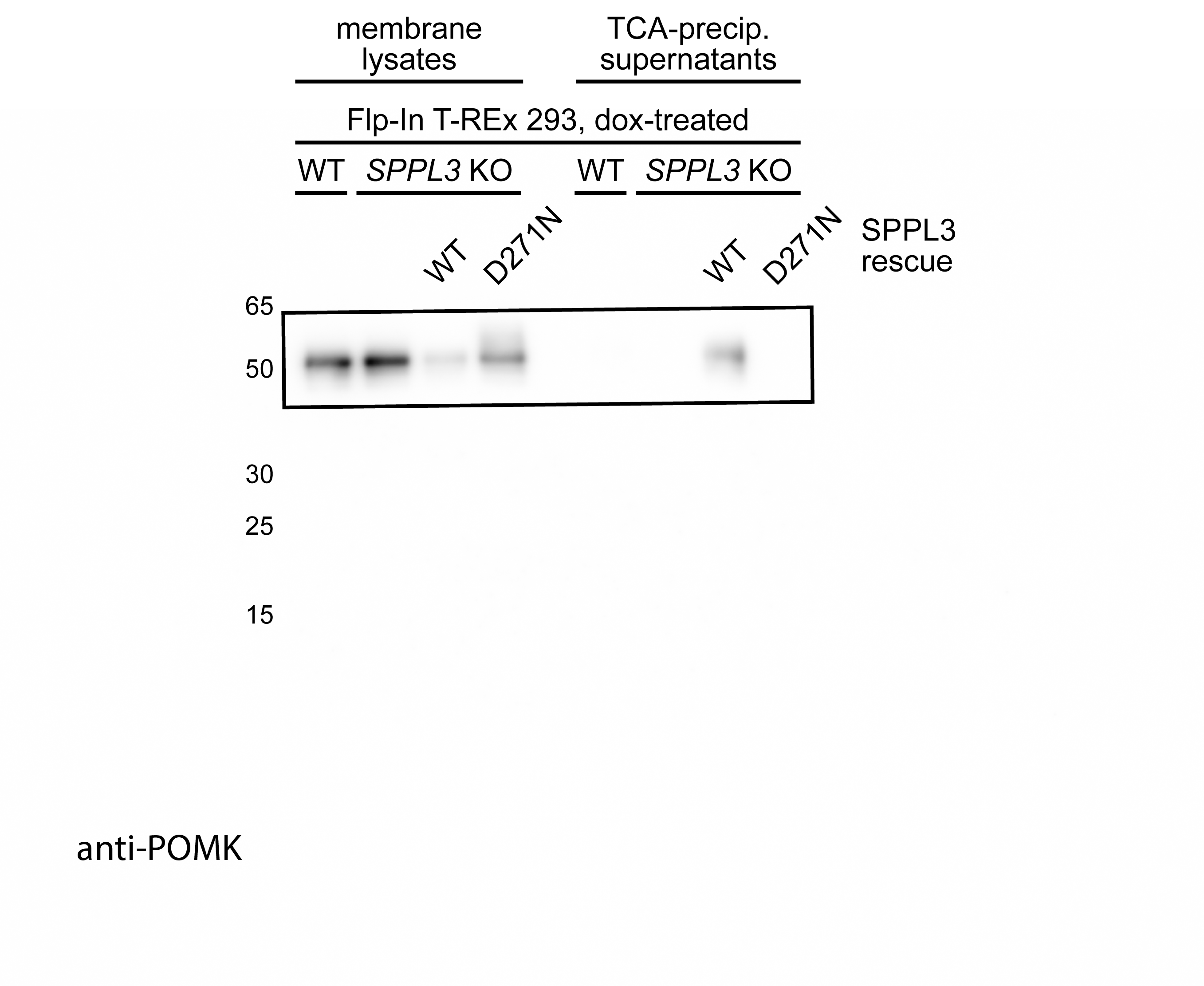

Supplement: Supplementary file 11 — Source data for Appendix [file 44318_2024_305_MOESM11_ESM.zip › Appendix/Appendix Figure S1/S1D/POMK 8bit annotated 20240213_165904-05_Ch_Chemi-01.tif]

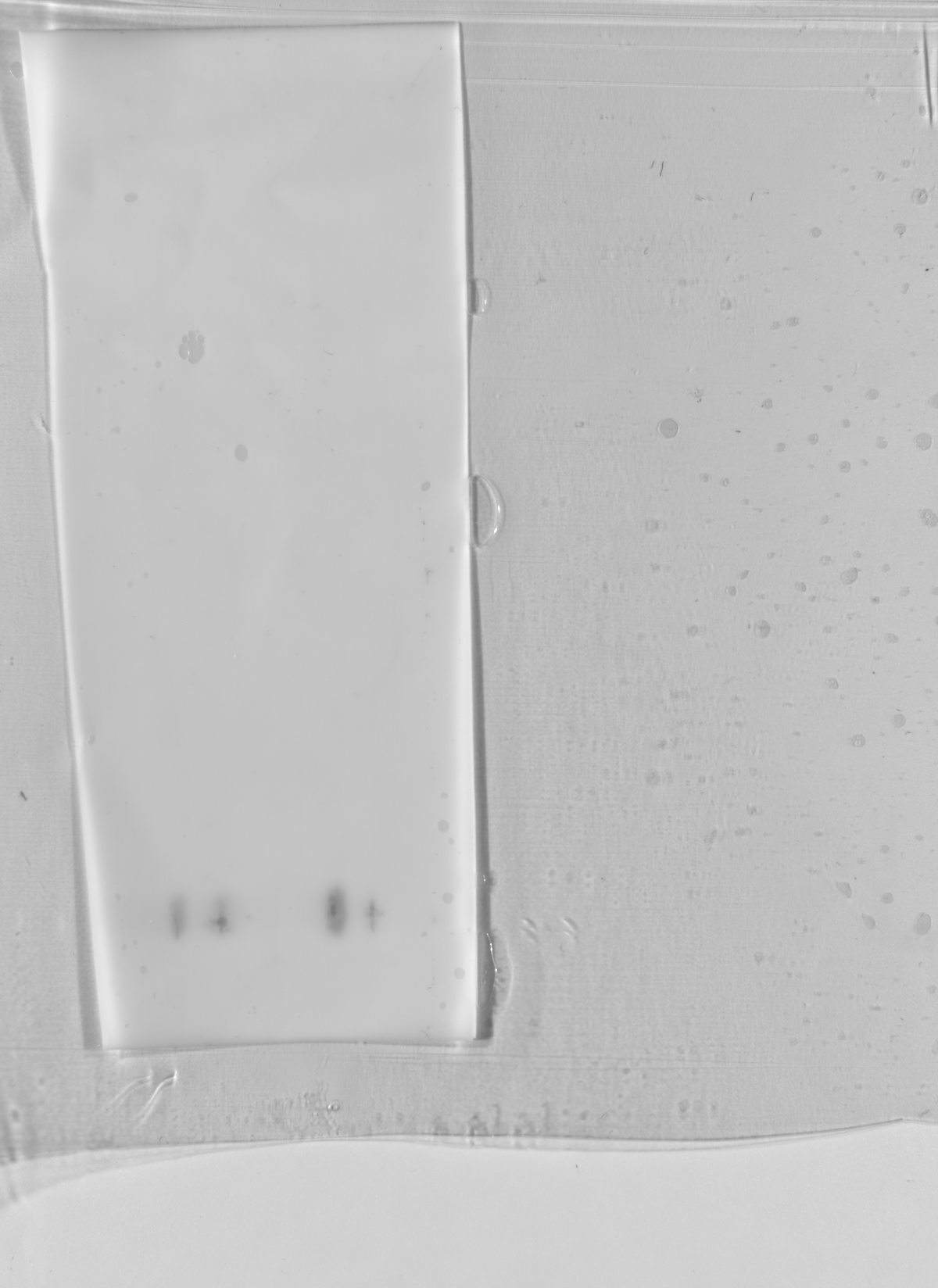

Supplement: Supplementary file 11 — Source data for Appendix [file 44318_2024_305_MOESM11_ESM.zip › Appendix/Appendix Figure S1/S1D/SPPL3 long exposure 16 bit original 20240206_173210-18_Ch-Marker.tif]
