# Supplementary figures and images for: GOLPH3 and GOLPH3L maintain Golgi localization of LYSET and a functional mannose 6-phosphate transport pathway (part 4 of 4)
Source: EMBO J. 2024 Nov 25;43(24):6264–90. doi: 10.1038/s44318-024-00305-z (PMC11649813; doi:10.1038/s44318-024-00305-z)

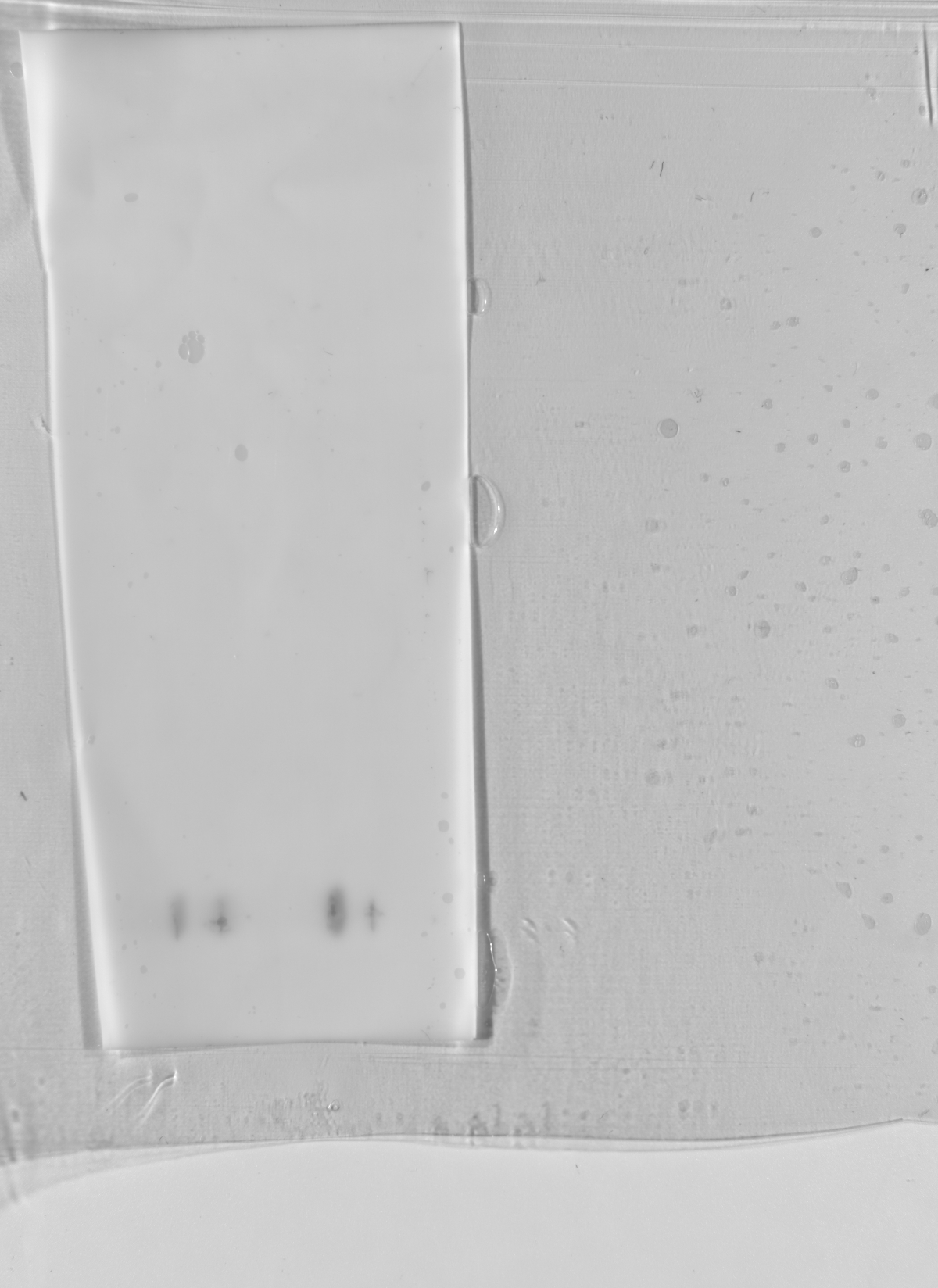

Supplement: Supplementary file 11 — Source data for Appendix [file 44318_2024_305_MOESM11_ESM.zip › Appendix/Appendix Figure S1/S1D/SPPL3 short exposure 16 bit original 20240206_173210-02_Ch-Marker.tif]

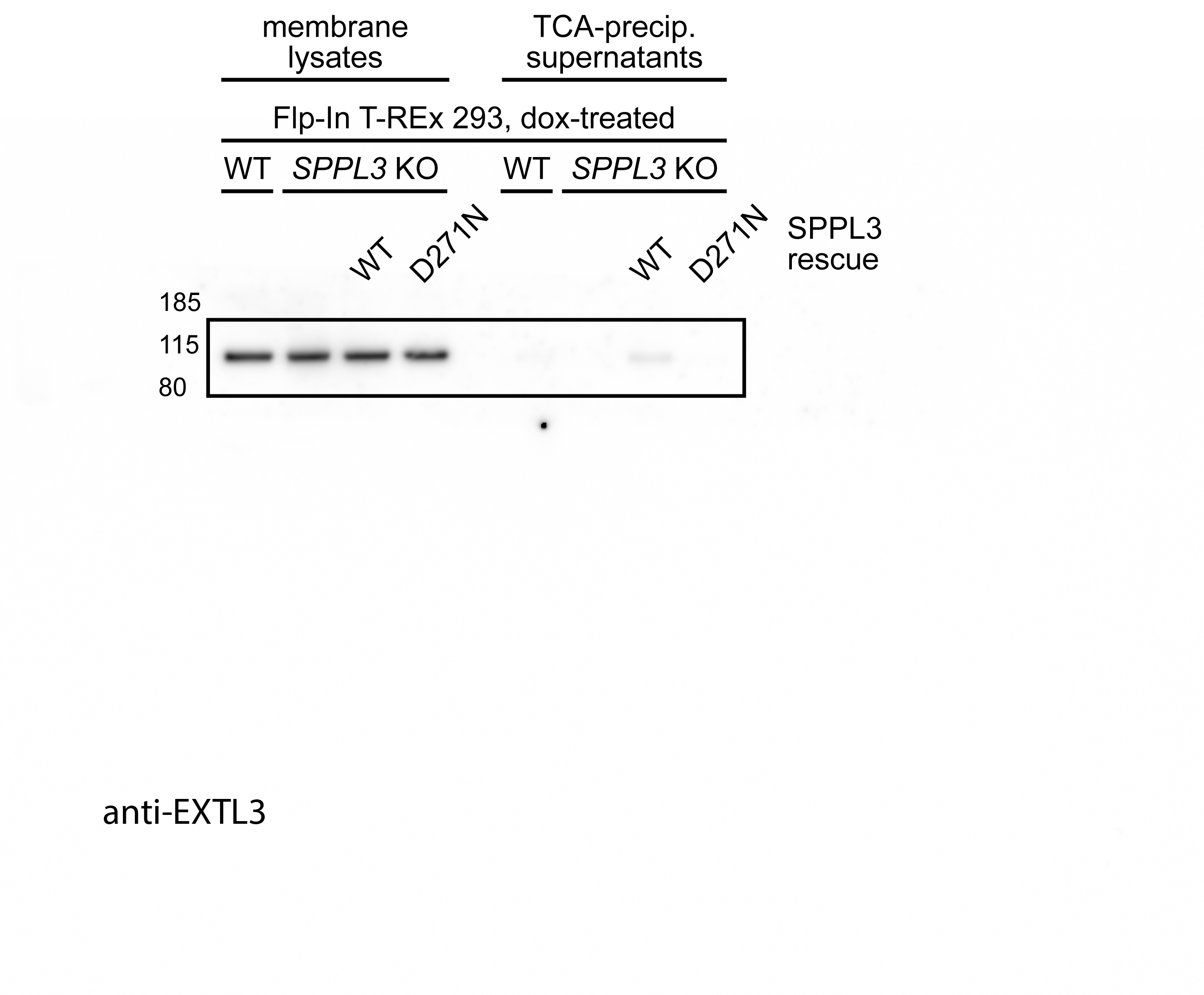

Supplement: Supplementary file 11 — Source data for Appendix [file 44318_2024_305_MOESM11_ESM.zip › Appendix/Appendix Figure S1/S1D/EXTL3 short exposure 8bit annotated 20240215_111115-06_Ch_Chemi-01.tif]

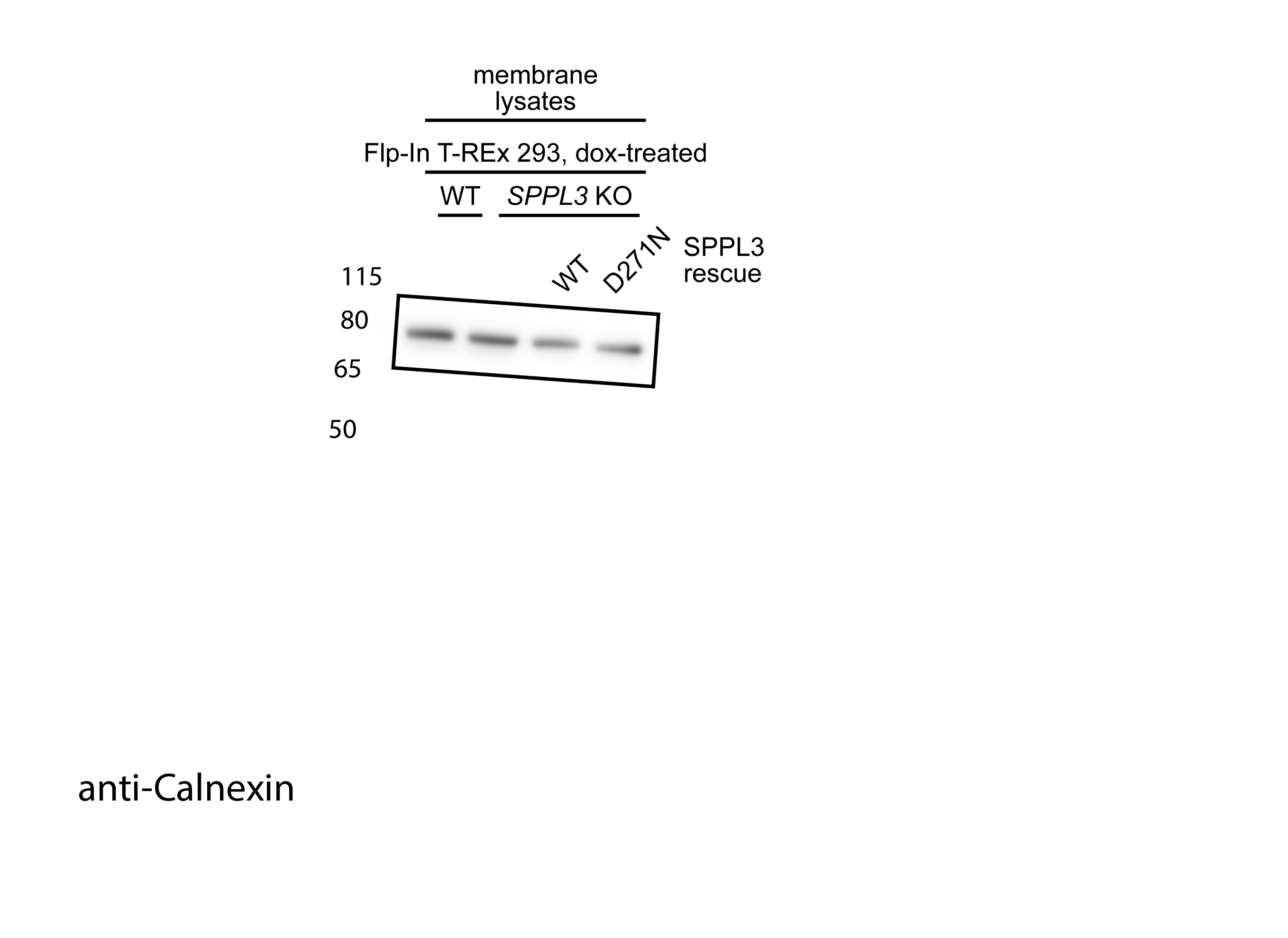

Supplement: Supplementary file 11 — Source data for Appendix [file 44318_2024_305_MOESM11_ESM.zip › Appendix/Appendix Figure S1/S1D/Calnexin 8bit annotated loading control for SPPL3 20240206_165416-01_Ch_Chemi-01.tif]

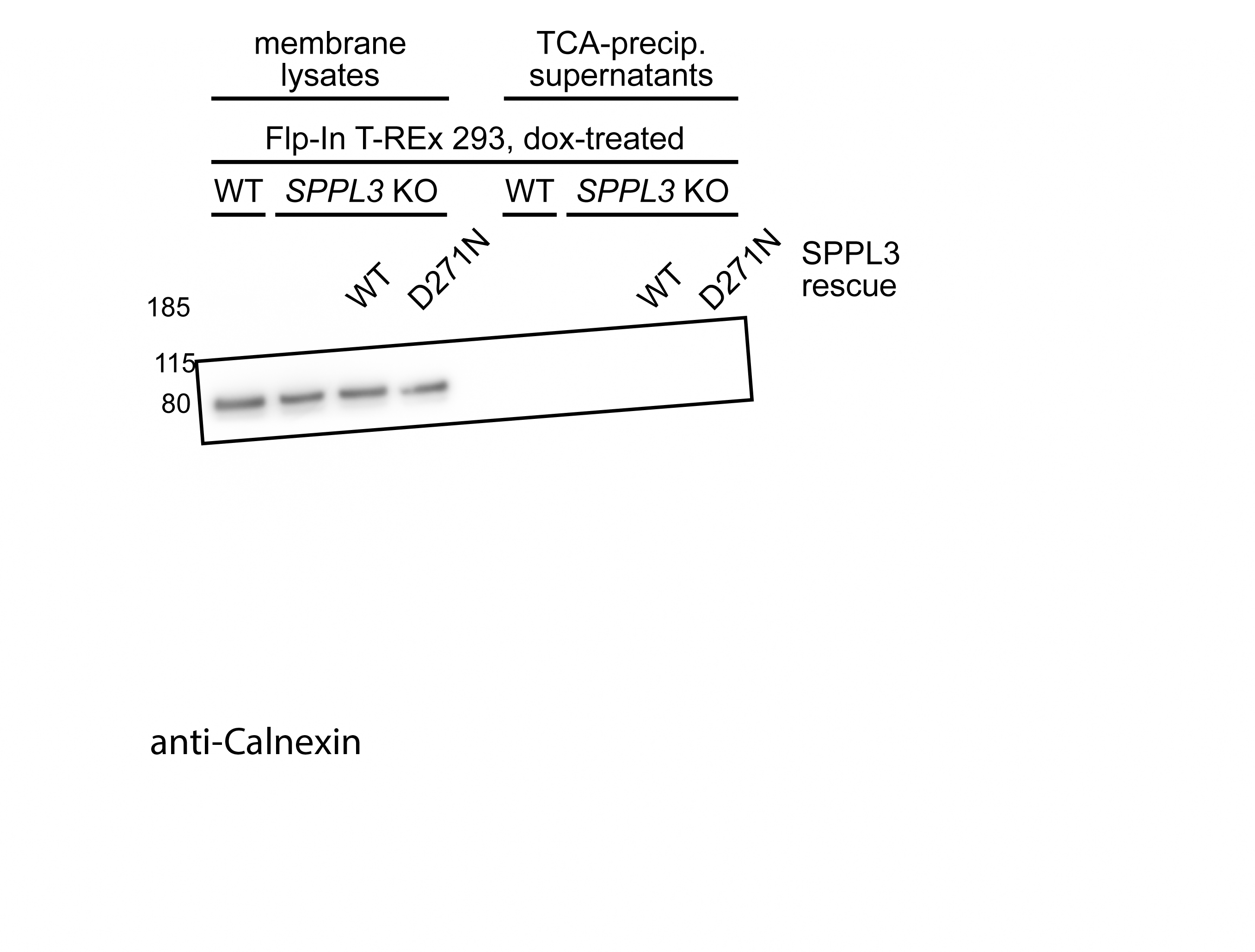

Supplement: Supplementary file 11 — Source data for Appendix [file 44318_2024_305_MOESM11_ESM.zip › Appendix/Appendix Figure S1/S1D/Calnexin 8bit annotated for MGAT5 EXTL3 B4GALT1 CANT1 20240222_112212_Ch_Chemi-01.tif]

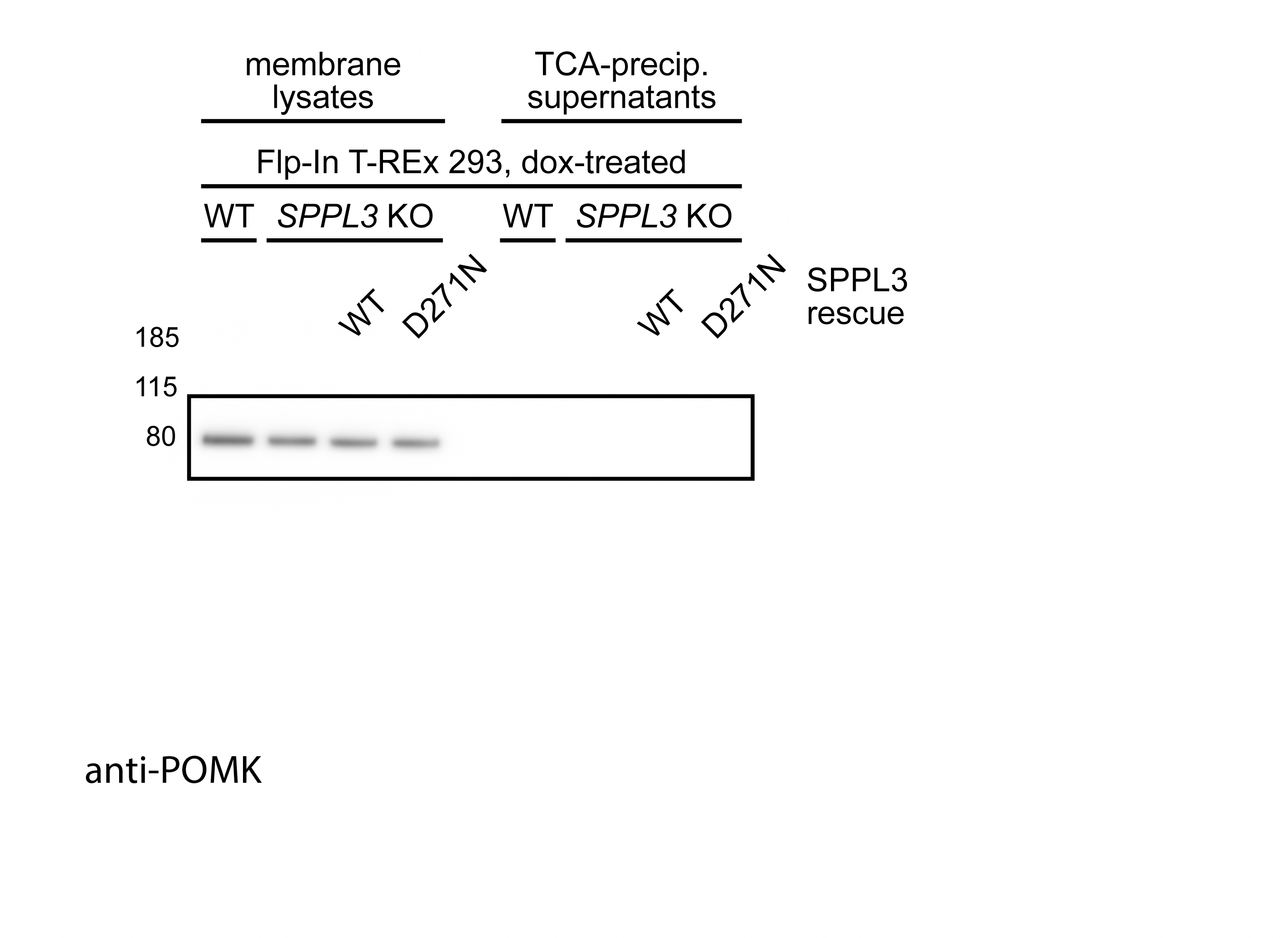

Supplement: Supplementary file 11 — Source data for Appendix [file 44318_2024_305_MOESM11_ESM.zip › Appendix/Appendix Figure S1/S1D/Calnexin 8bit annotated for POMK 20240222_112739_Ch_Chemi-01.tif]

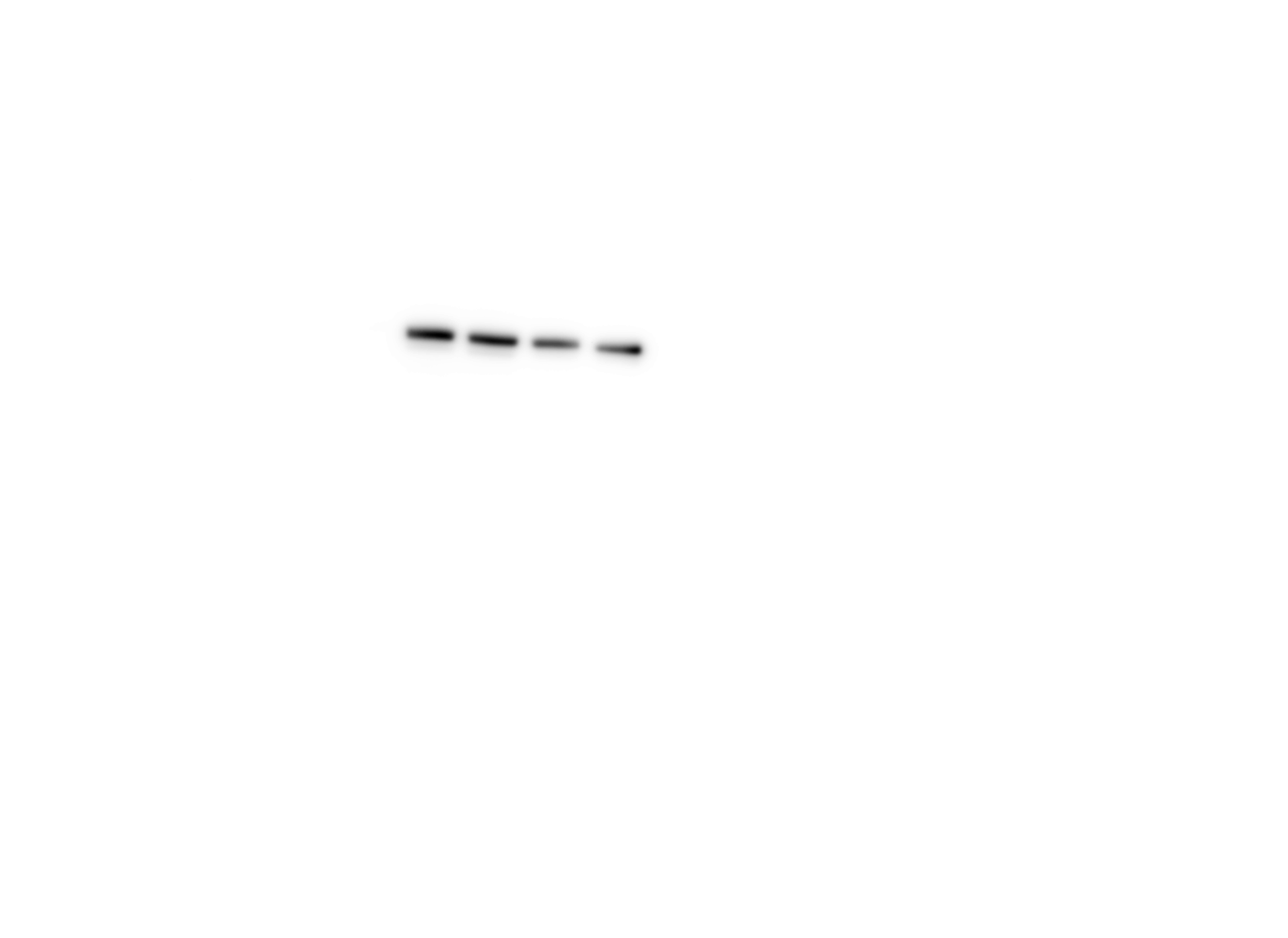

Supplement: Supplementary file 11 — Source data for Appendix [file 44318_2024_305_MOESM11_ESM.zip › Appendix/Appendix Figure S1/S1D/Calnexin 16bit original loading control for SPPL3 20240206_165416-01_Ch_Chemi.tif]

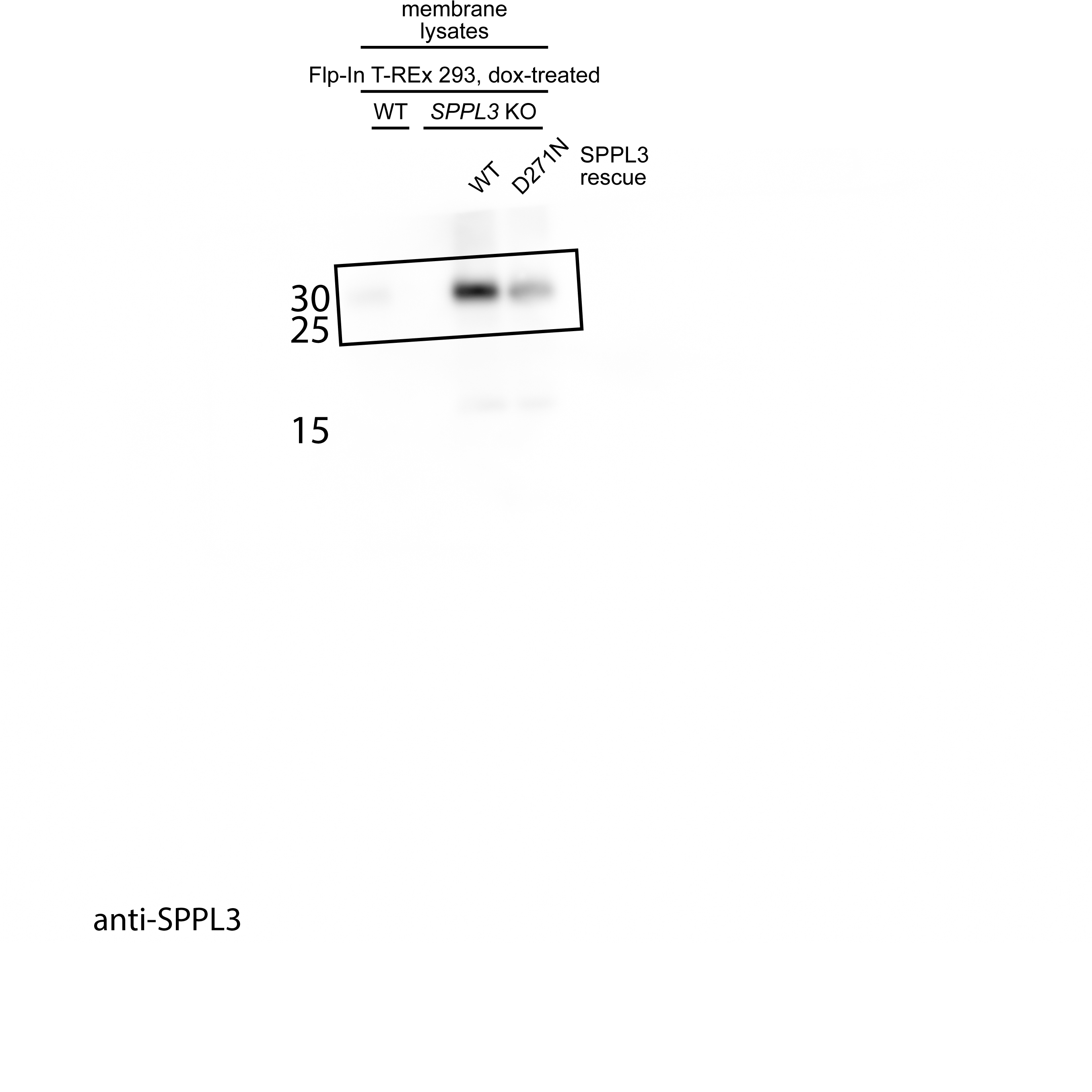

Supplement: Supplementary file 11 — Source data for Appendix [file 44318_2024_305_MOESM11_ESM.zip › Appendix/Appendix Figure S1/S1D/SPPL3 short exposure 8bit annotated 20240206_173210-02_Ch_Chemi-01.tif]

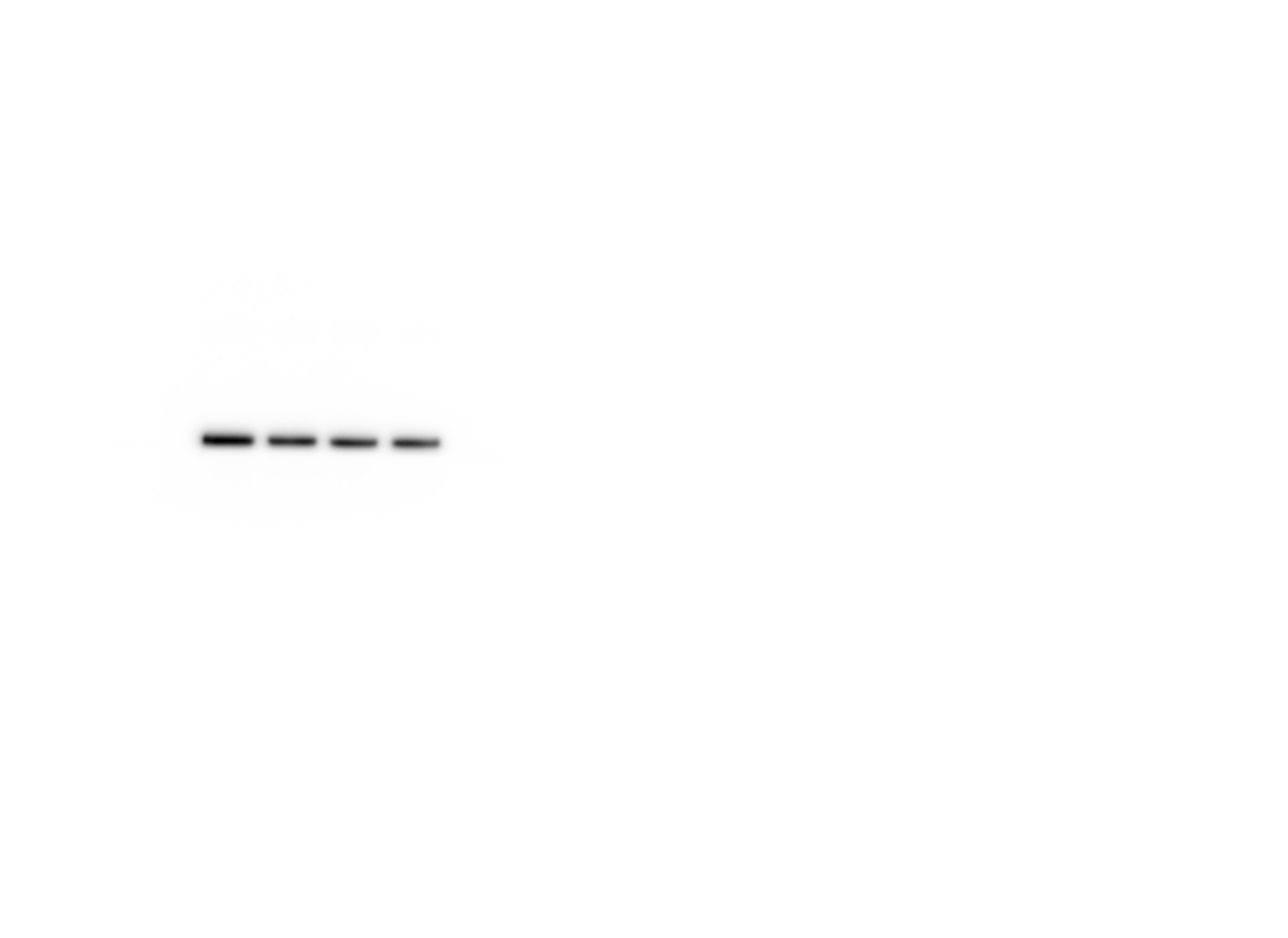

Supplement: Supplementary file 11 — Source data for Appendix [file 44318_2024_305_MOESM11_ESM.zip › Appendix/Appendix Figure S1/S1D/Calnexin16bit original for POMK 20240222_112739_Ch_Chemi.tif]

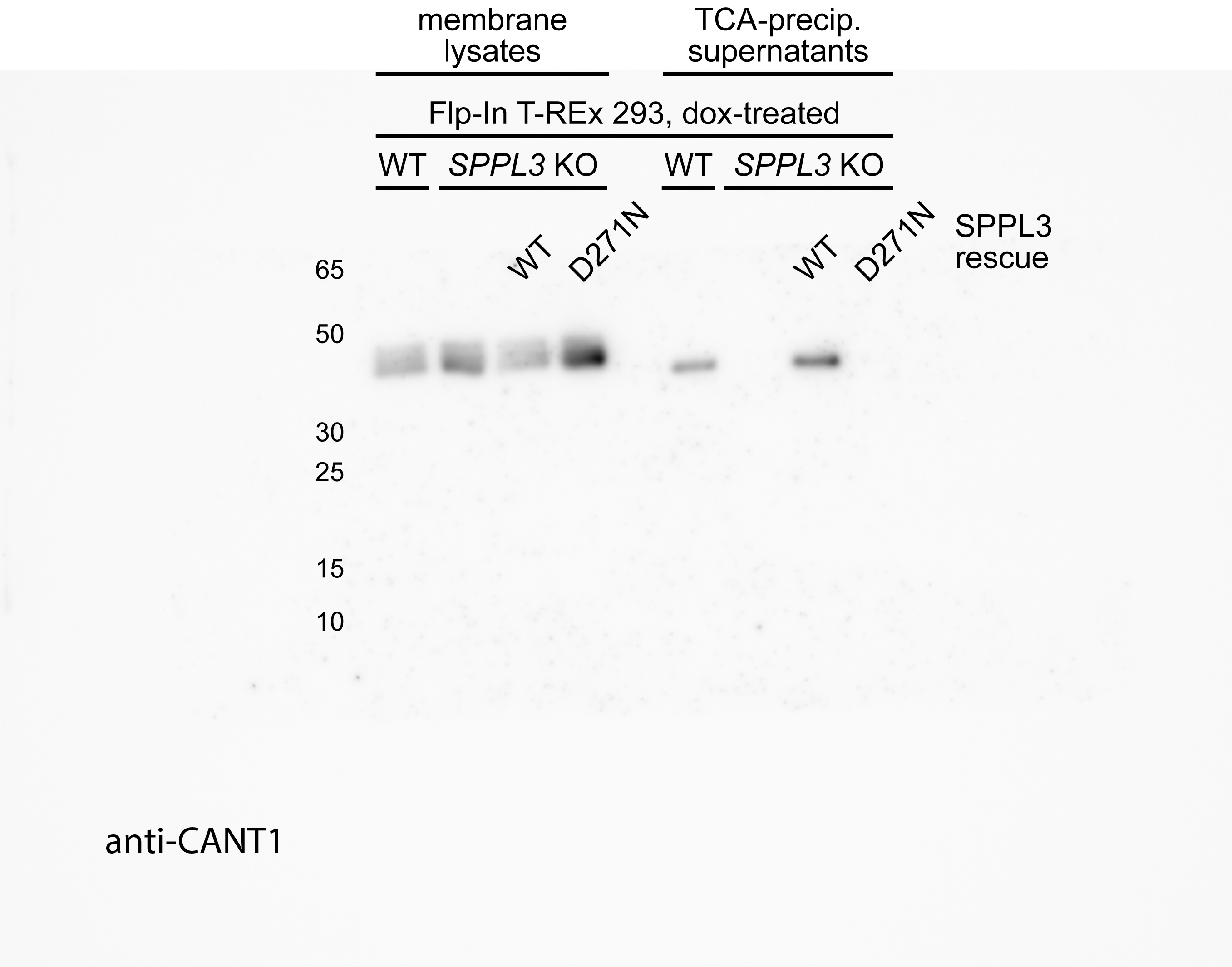

Supplement: Supplementary file 11 — Source data for Appendix [file 44318_2024_305_MOESM11_ESM.zip › Appendix/Appendix Figure S1/S1D/CANT1 8bit annotated 20240213_161512-48_Ch_Chemi-01.tif]

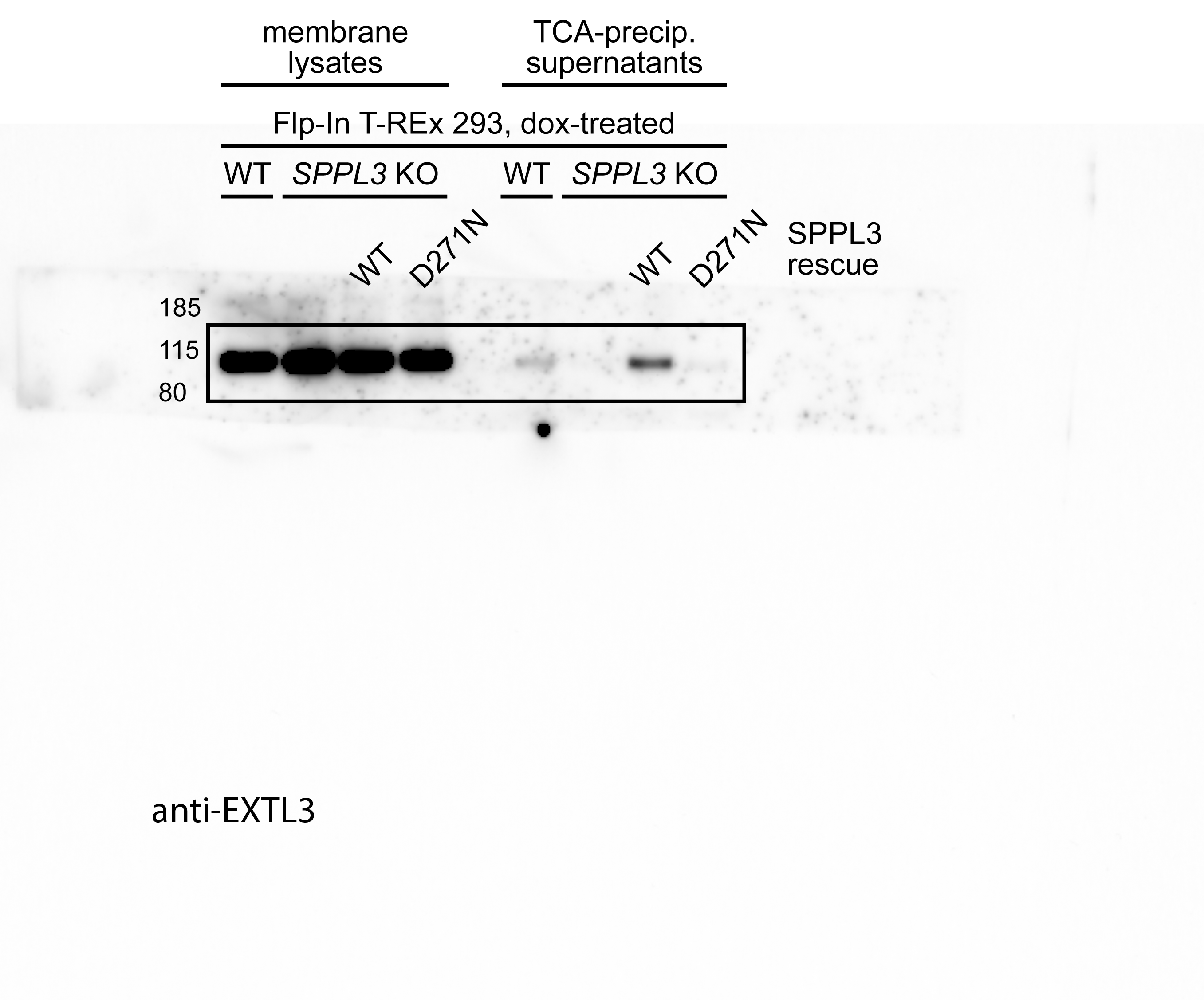

Supplement: Supplementary file 11 — Source data for Appendix [file 44318_2024_305_MOESM11_ESM.zip › Appendix/Appendix Figure S1/S1D/EXTL3 long exposure 8bit annotated 20240215_111115-49_Ch_Chemi-01.tif]

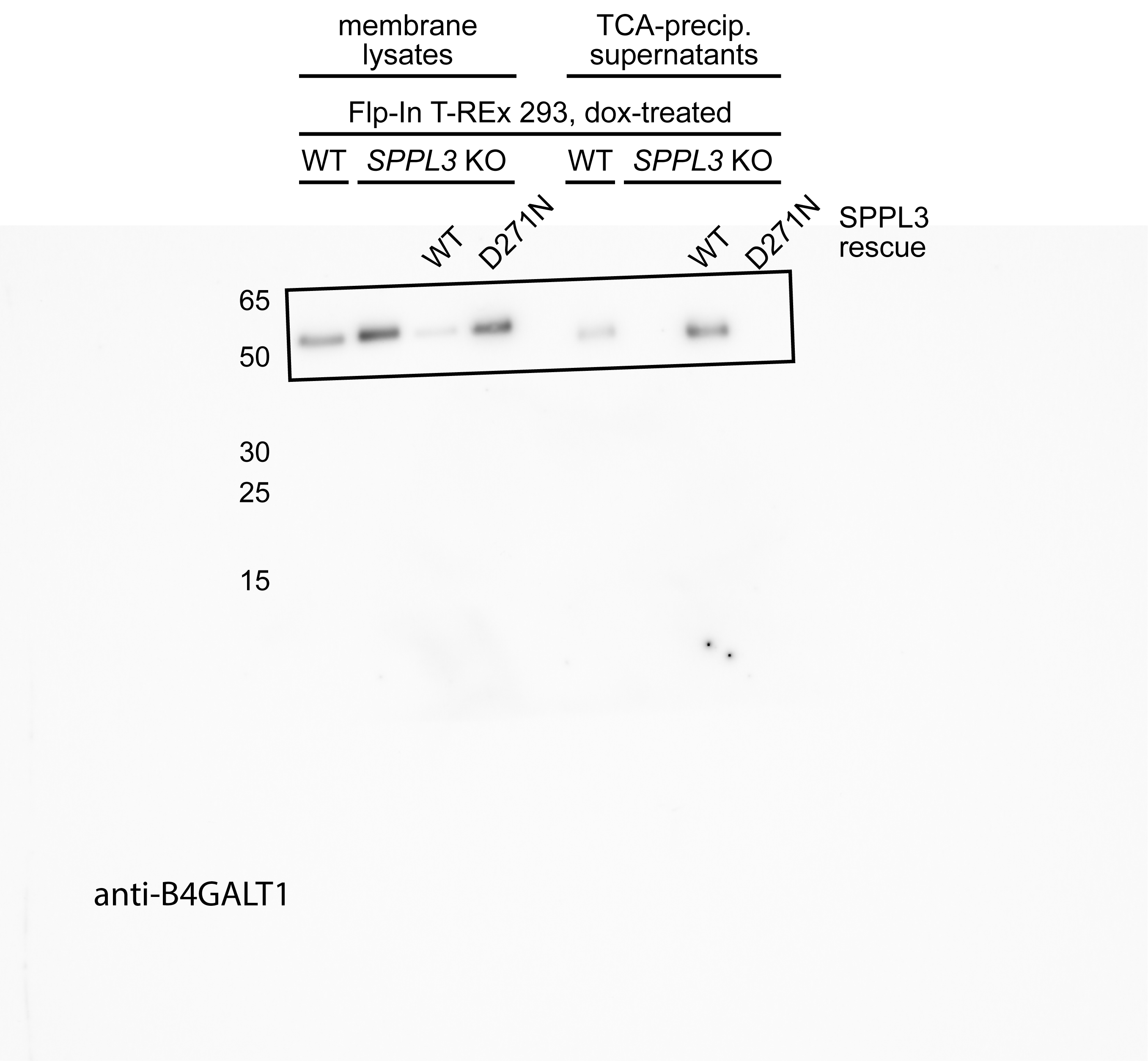

Supplement: Supplementary file 11 — Source data for Appendix [file 44318_2024_305_MOESM11_ESM.zip › Appendix/Appendix Figure S1/S1D/B4GALT1 8bit annotated 20240206_171041-50_Ch_Chemi-01.tif]

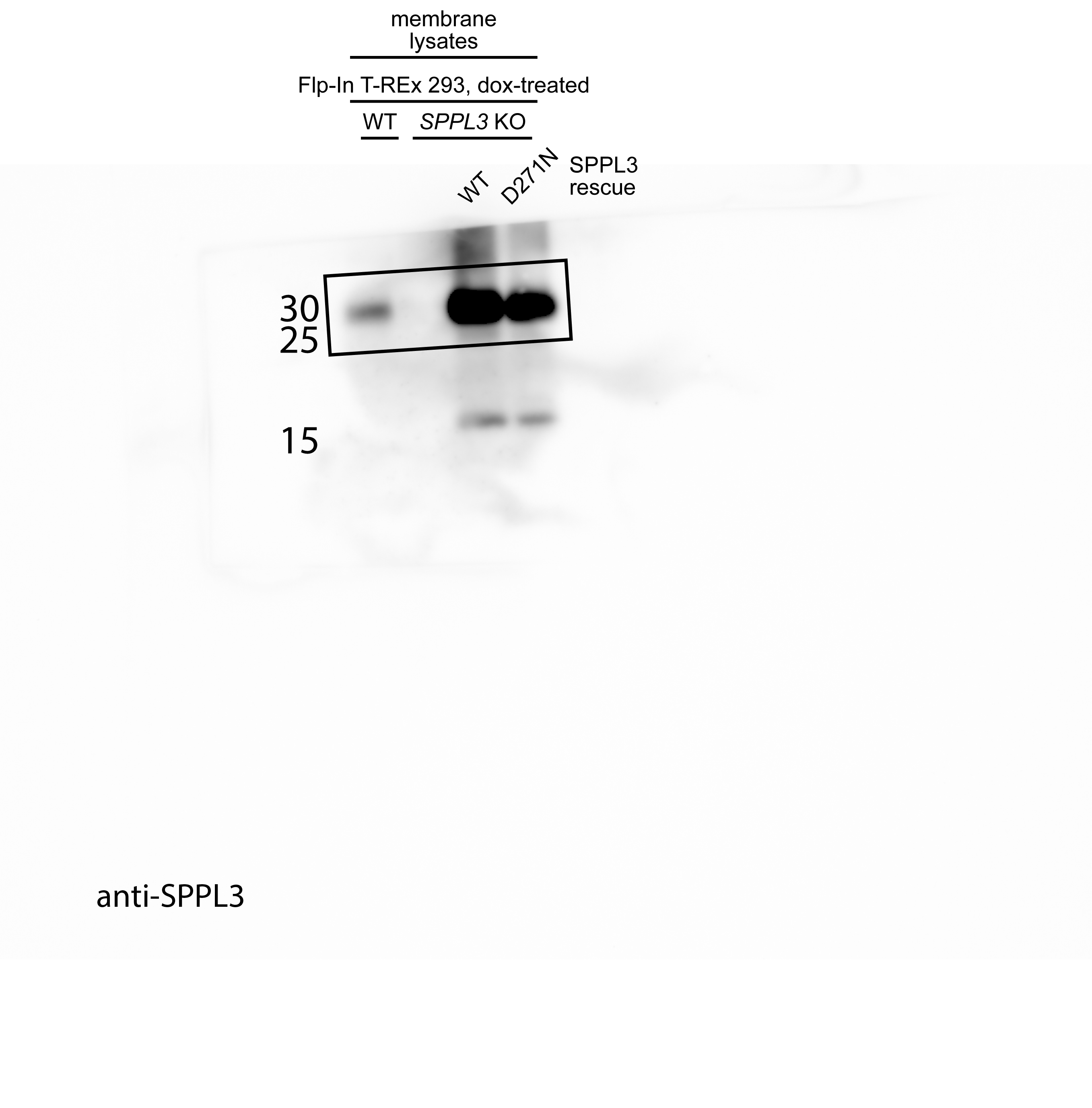

Supplement: Supplementary file 11 — Source data for Appendix [file 44318_2024_305_MOESM11_ESM.zip › Appendix/Appendix Figure S1/S1D/SPPL3 long exposure 8bit annotated 20240206_173210-18_Ch_Chemi-01.tif]

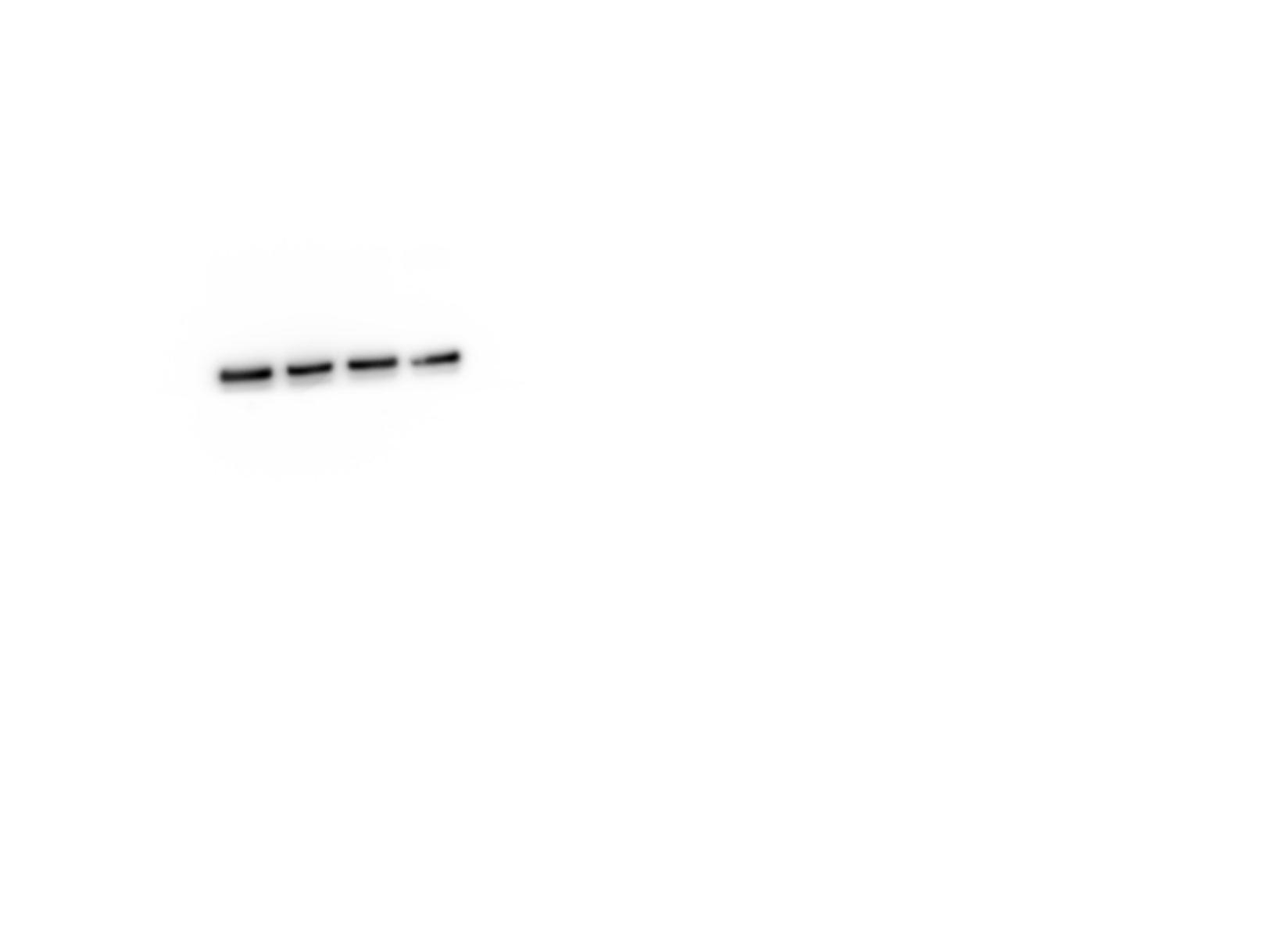

Supplement: Supplementary file 11 — Source data for Appendix [file 44318_2024_305_MOESM11_ESM.zip › Appendix/Appendix Figure S1/S1D/Calnexin 16bit original for MGAT5 EXTL3 B4GALT1 CANT1 20240222_112212_Ch_Chemi.tif]

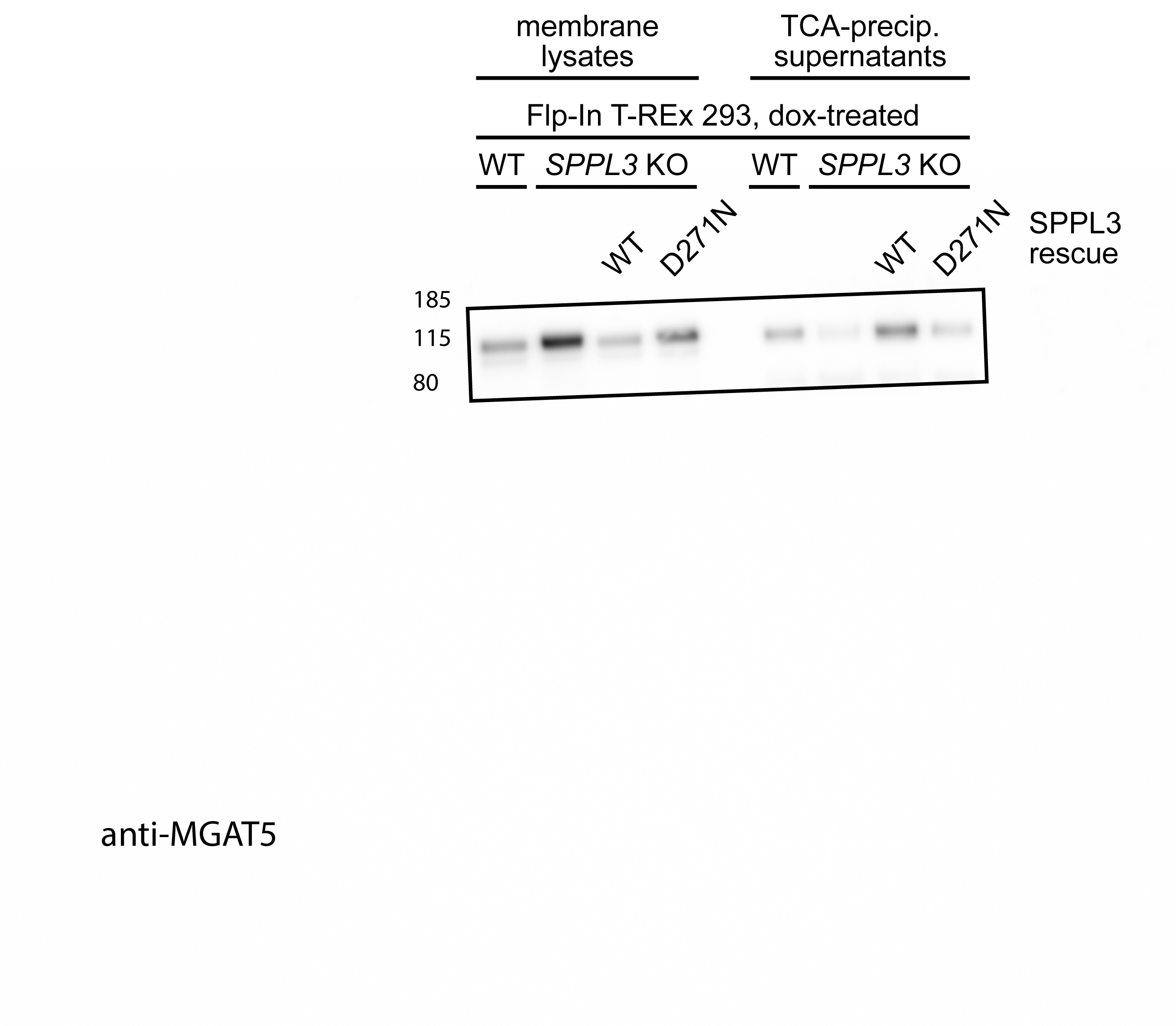

Supplement: Supplementary file 11 — Source data for Appendix [file 44318_2024_305_MOESM11_ESM.zip › Appendix/Appendix Figure S1/S1D/MGAT5 8bit annotated 20240213_155128-05_Ch_Chemi-01.tif]

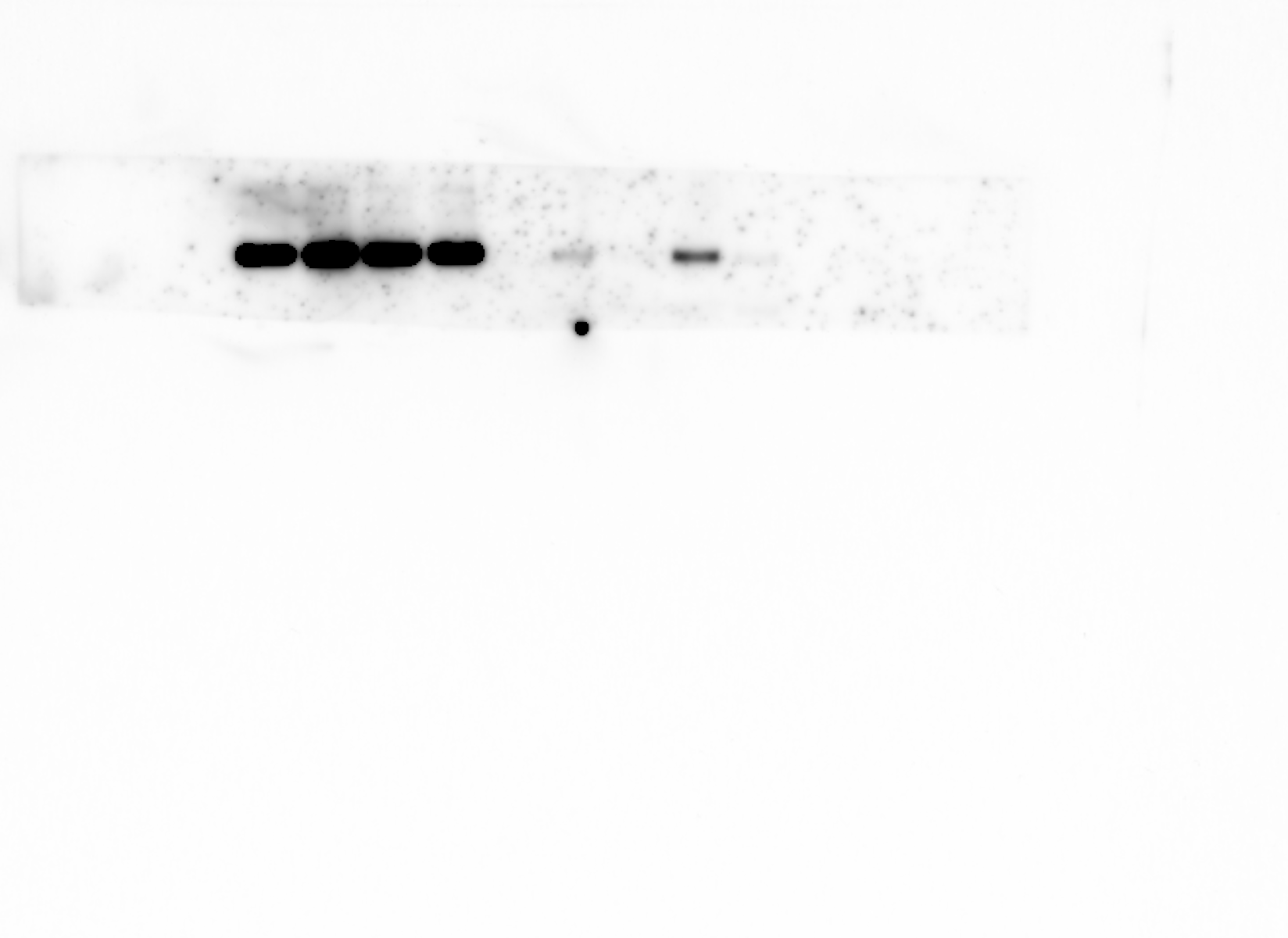

Supplement: Supplementary file 11 — Source data for Appendix [file 44318_2024_305_MOESM11_ESM.zip › Appendix/Appendix Figure S1/S1D/EXTL3 long exposure 16bit original 20240215_111115-49_Ch_Chemi.tif]

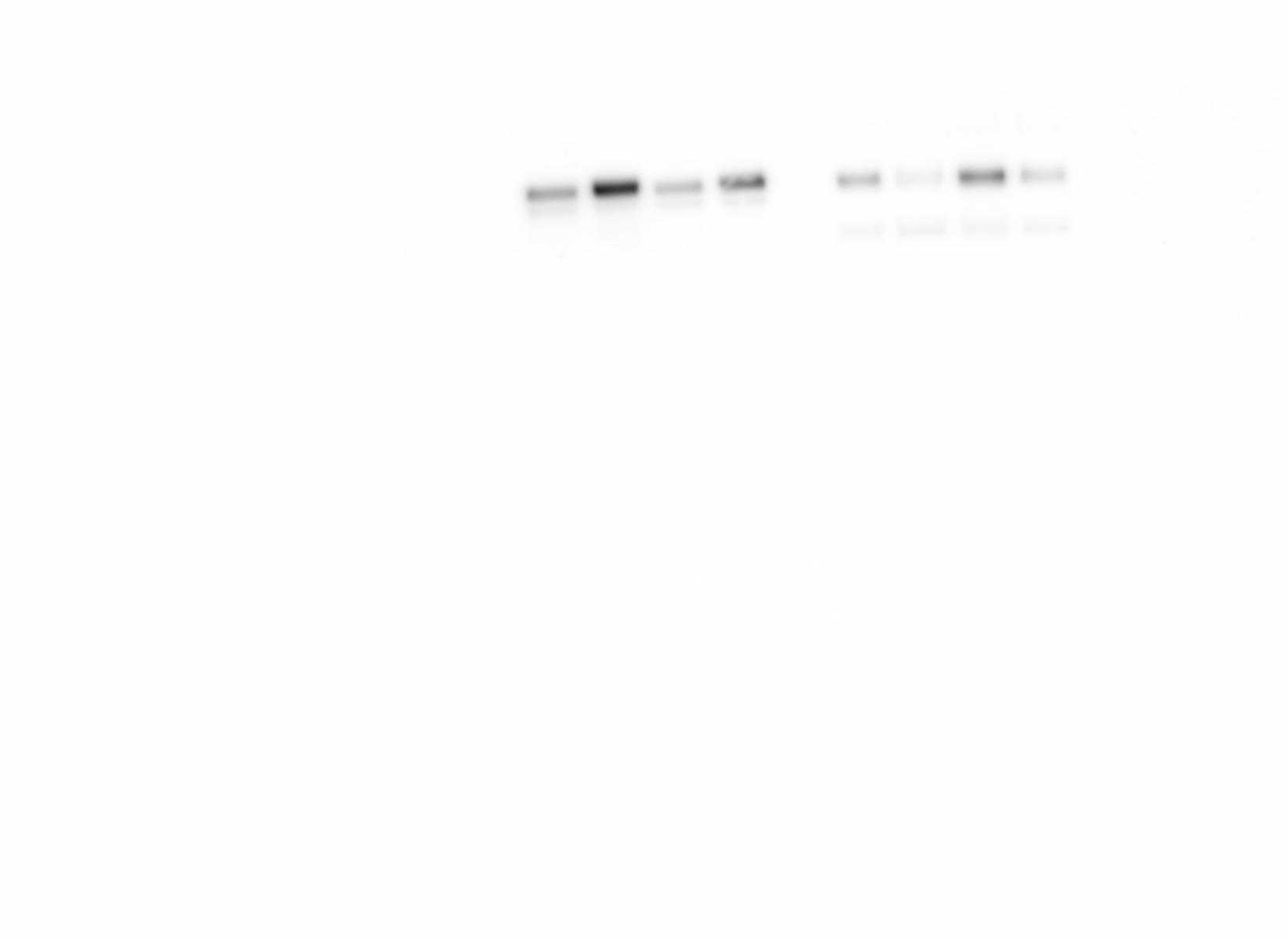

Supplement: Supplementary file 11 — Source data for Appendix [file 44318_2024_305_MOESM11_ESM.zip › Appendix/Appendix Figure S1/S1D/MGAT5 16bit original 20240213_155128-05_Ch_Chemi.tif]

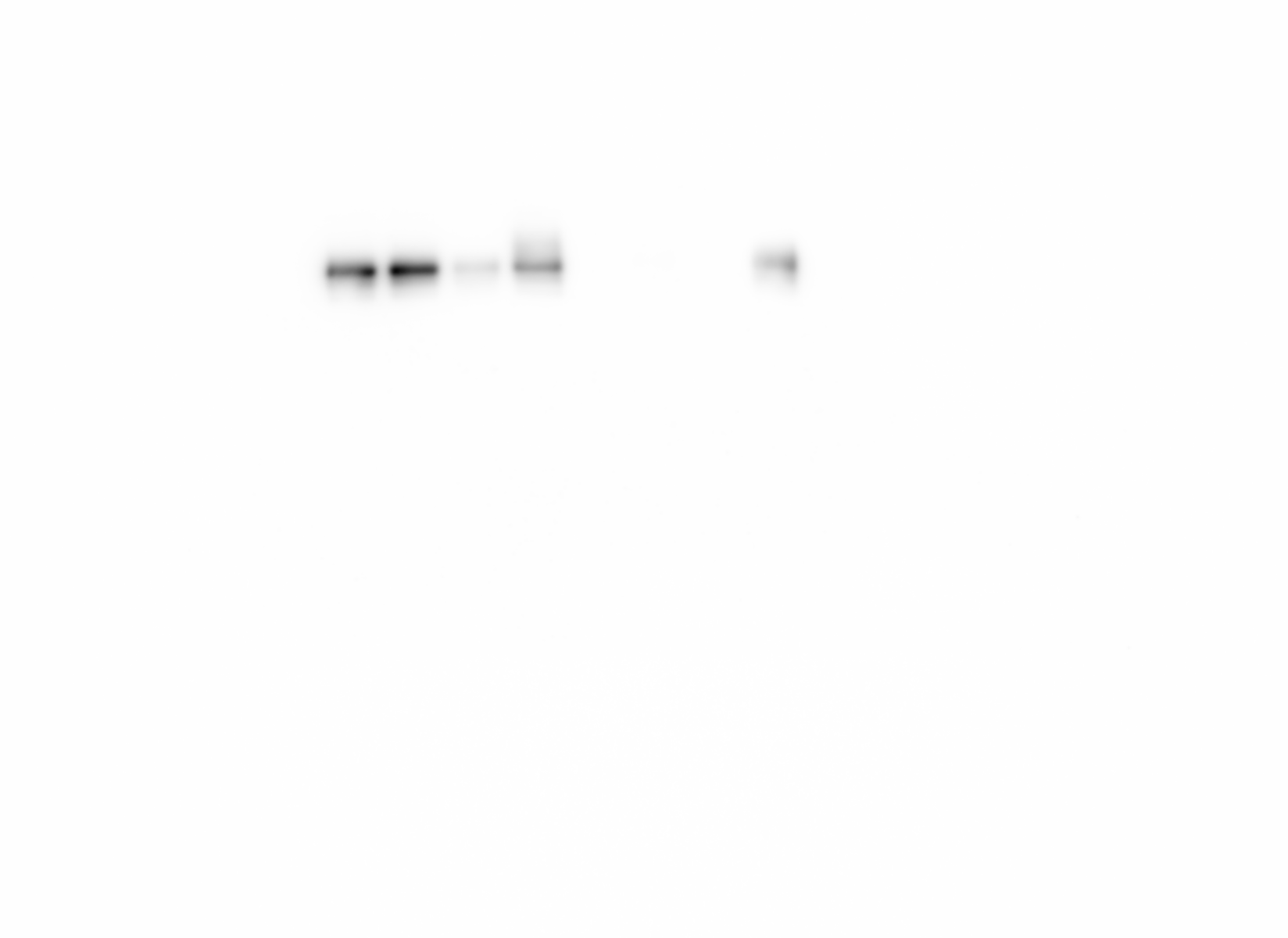

Supplement: Supplementary file 11 — Source data for Appendix [file 44318_2024_305_MOESM11_ESM.zip › Appendix/Appendix Figure S1/S1D/POMK 16bit original 20240213_165904-05_Ch_Chemi.tif]

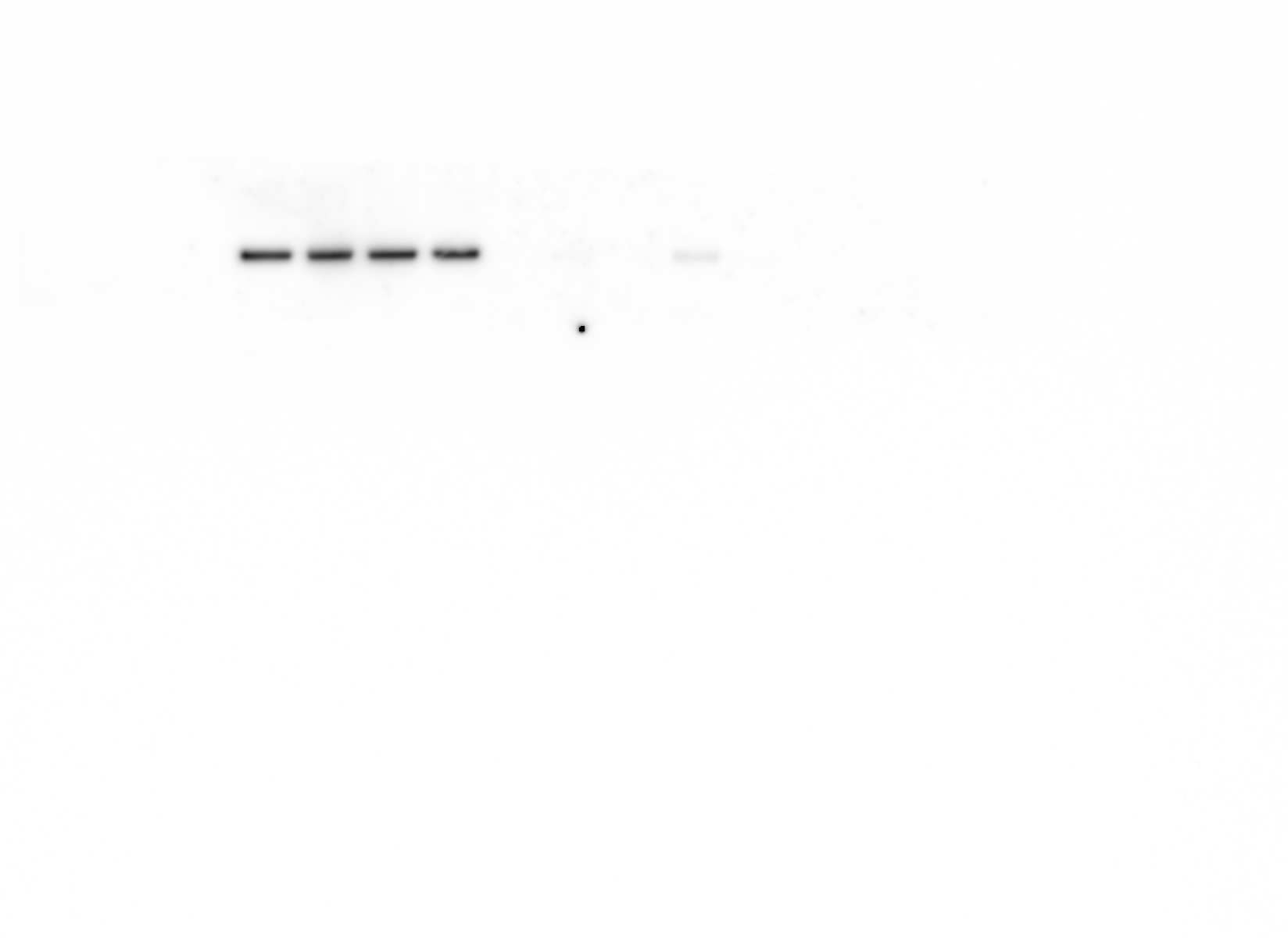

Supplement: Supplementary file 11 — Source data for Appendix [file 44318_2024_305_MOESM11_ESM.zip › Appendix/Appendix Figure S1/S1D/EXTL3 short exposure 16bit original 20240215_111115-06_Ch_Chemi.tif]

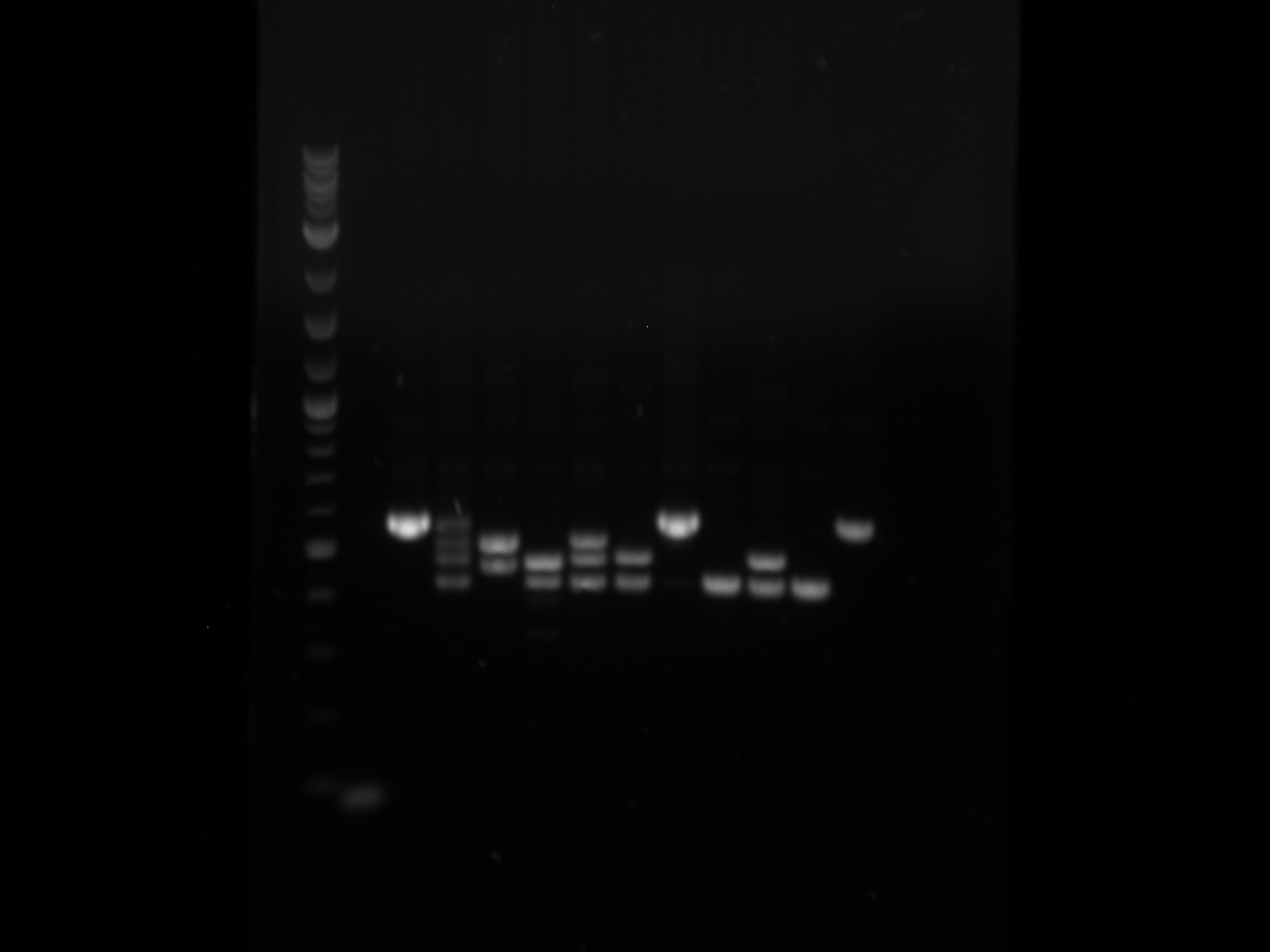

Supplement: Supplementary file 11 — Source data for Appendix [file 44318_2024_305_MOESM11_ESM.zip › Appendix/Appendix Figure S1/S1B/original 200311_flipin_4.tif]

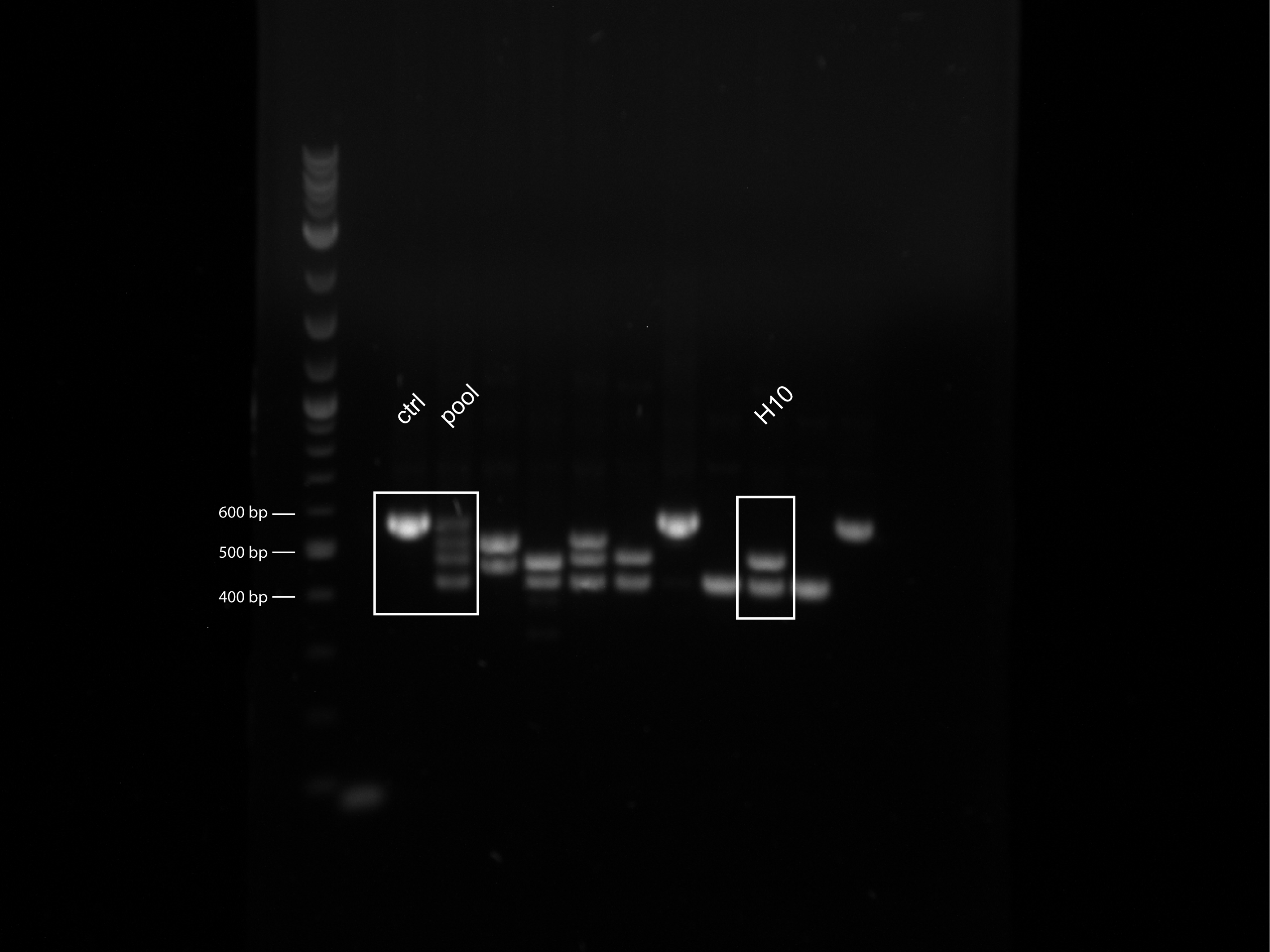

Supplement: Supplementary file 11 — Source data for Appendix [file 44318_2024_305_MOESM11_ESM.zip › Appendix/Appendix Figure S1/S1B/annotated 200311_flipin_4-01.tif]

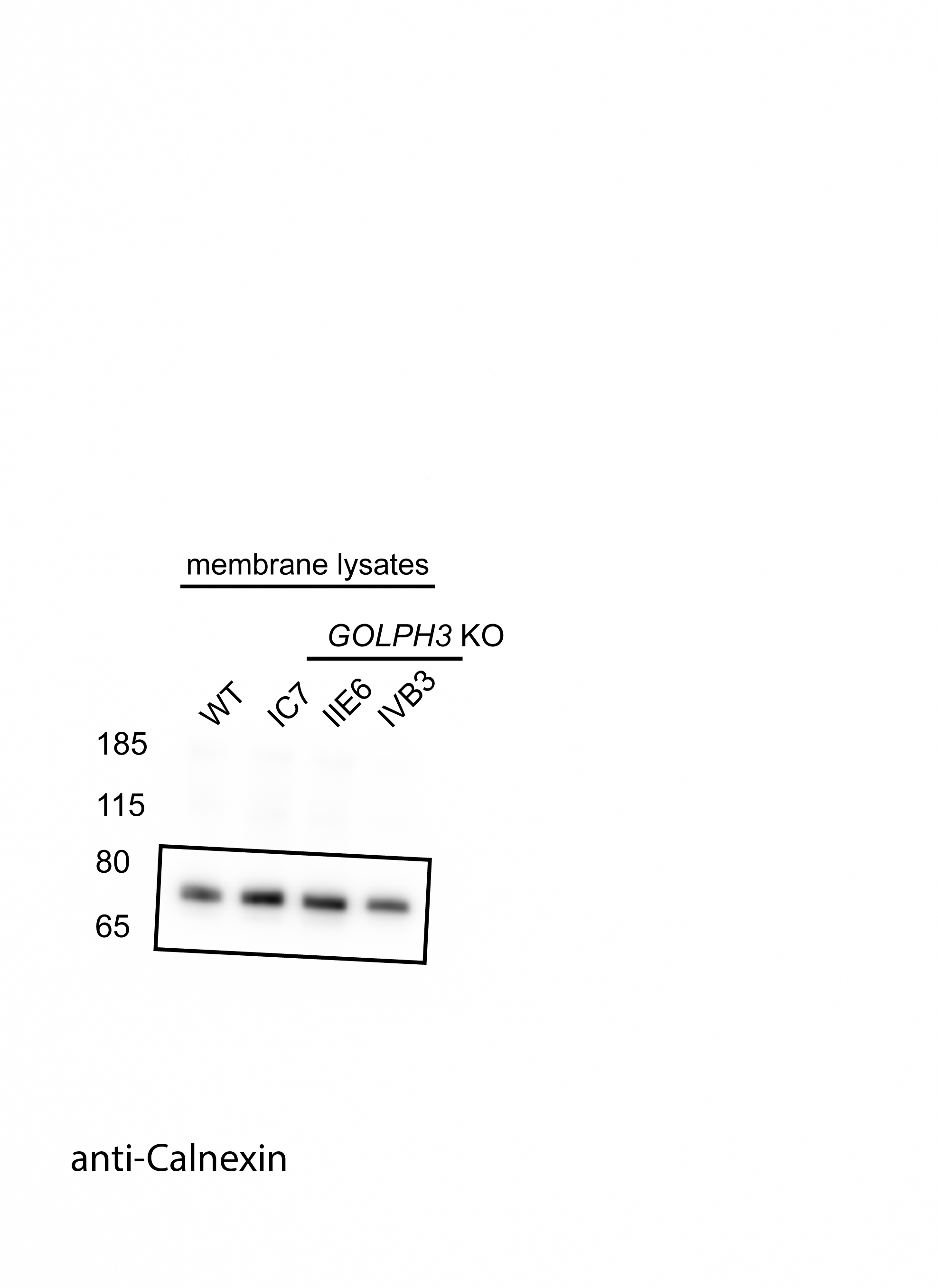

Supplement: Supplementary file 11 — Source data for Appendix [file 44318_2024_305_MOESM11_ESM.zip › Appendix/Appendix Figure S3/S3D/Calnexin 8bit annotated for GALNT7 MAN1B1 CANT1 20240929_172905-04_Ch_Chemi-01.tif]

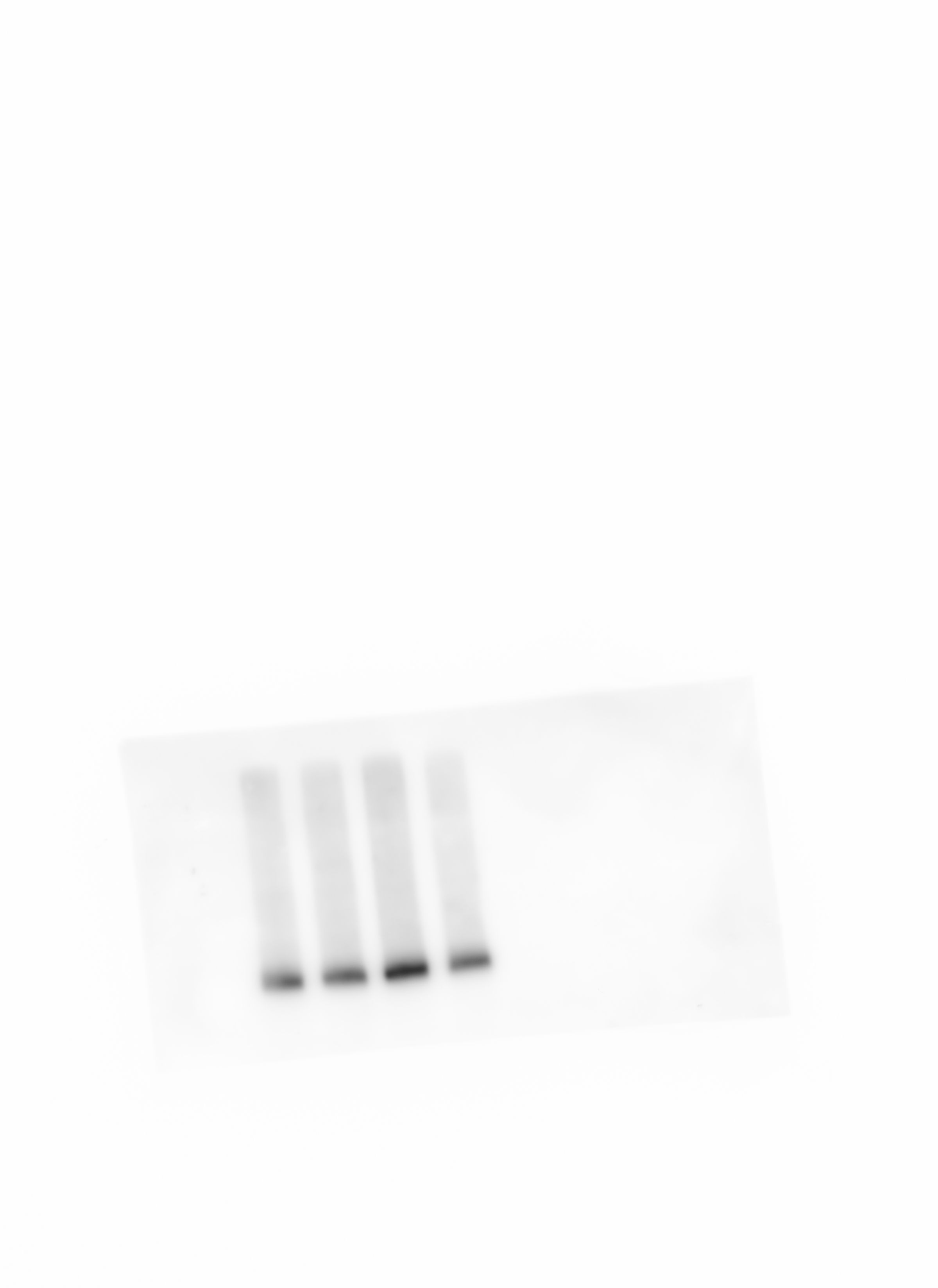

Supplement: Supplementary file 11 — Source data for Appendix [file 44318_2024_305_MOESM11_ESM.zip › Appendix/Appendix Figure S3/S3D/GALNT7 16bit original 20240927_142323-03_Ch_Chemi.tif]

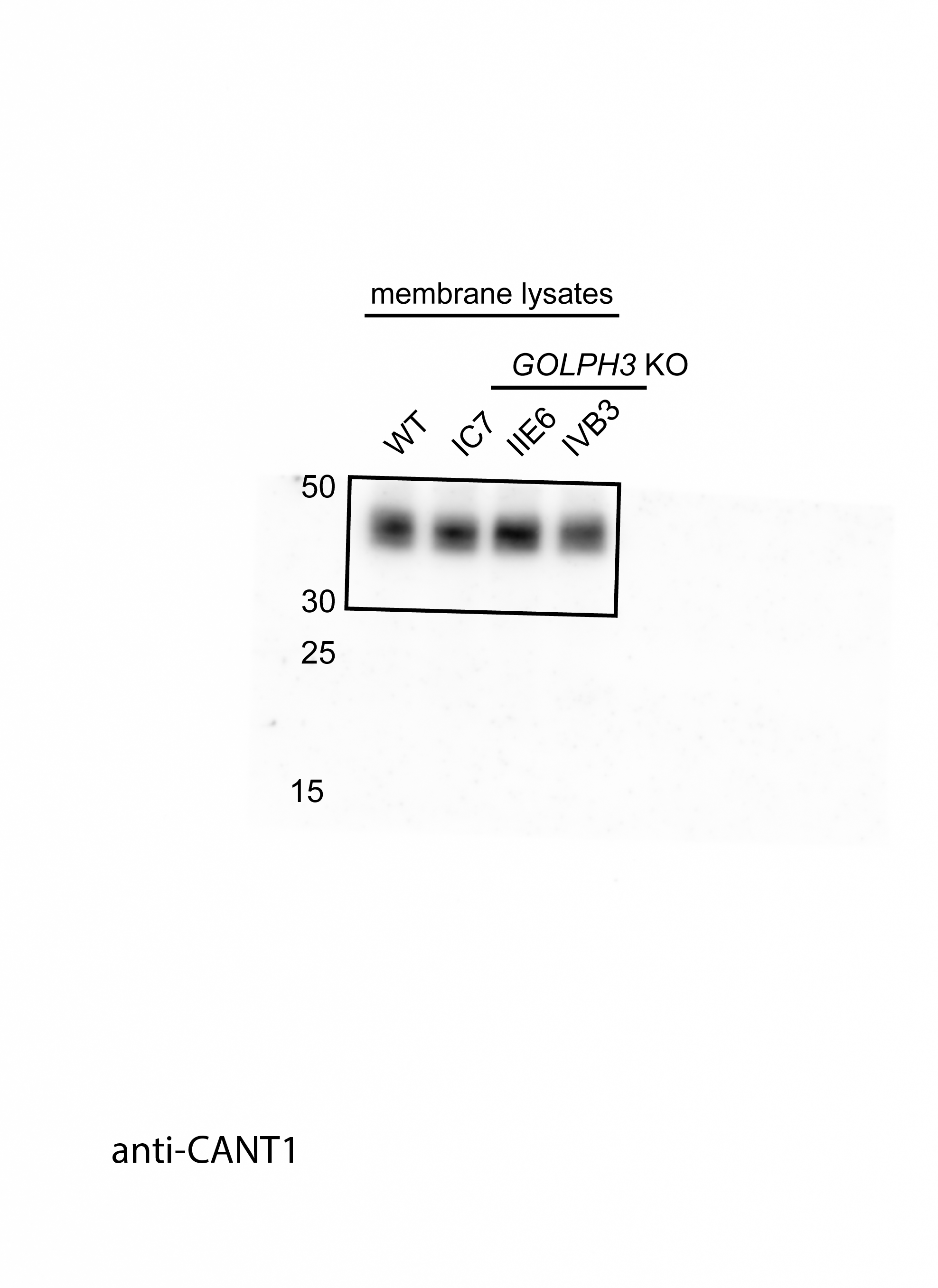

Supplement: Supplementary file 11 — Source data for Appendix [file 44318_2024_305_MOESM11_ESM.zip › Appendix/Appendix Figure S3/S3D/CANT1 8bit annotated 20240926_140623-10_Ch_Chemi-01.tif]

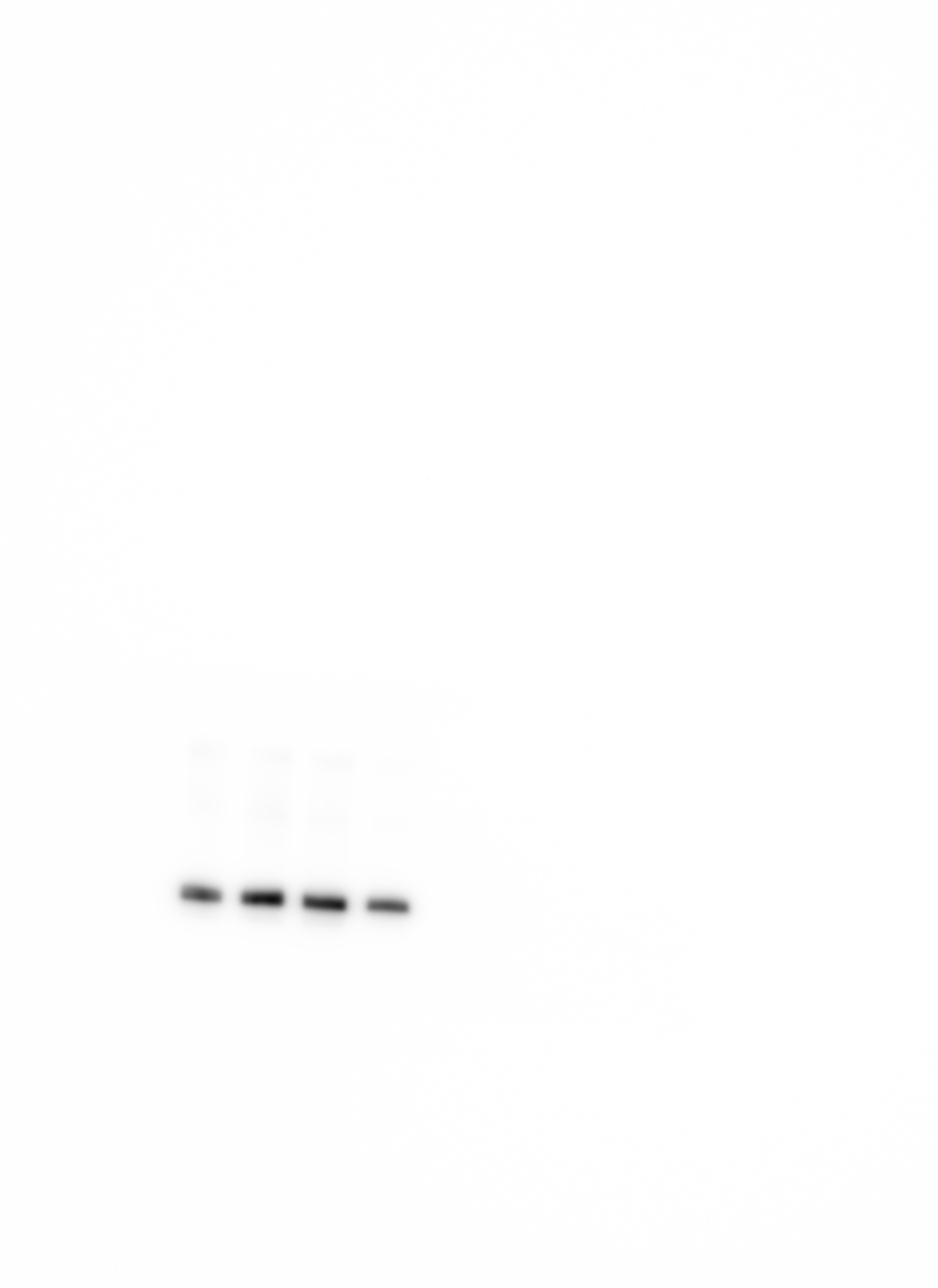

Supplement: Supplementary file 11 — Source data for Appendix [file 44318_2024_305_MOESM11_ESM.zip › Appendix/Appendix Figure S3/S3D/Calnexin 16 bit original for GALNT7 MAN1B1 CANT1 20240929_172905-04_Ch_Chemi.tif]

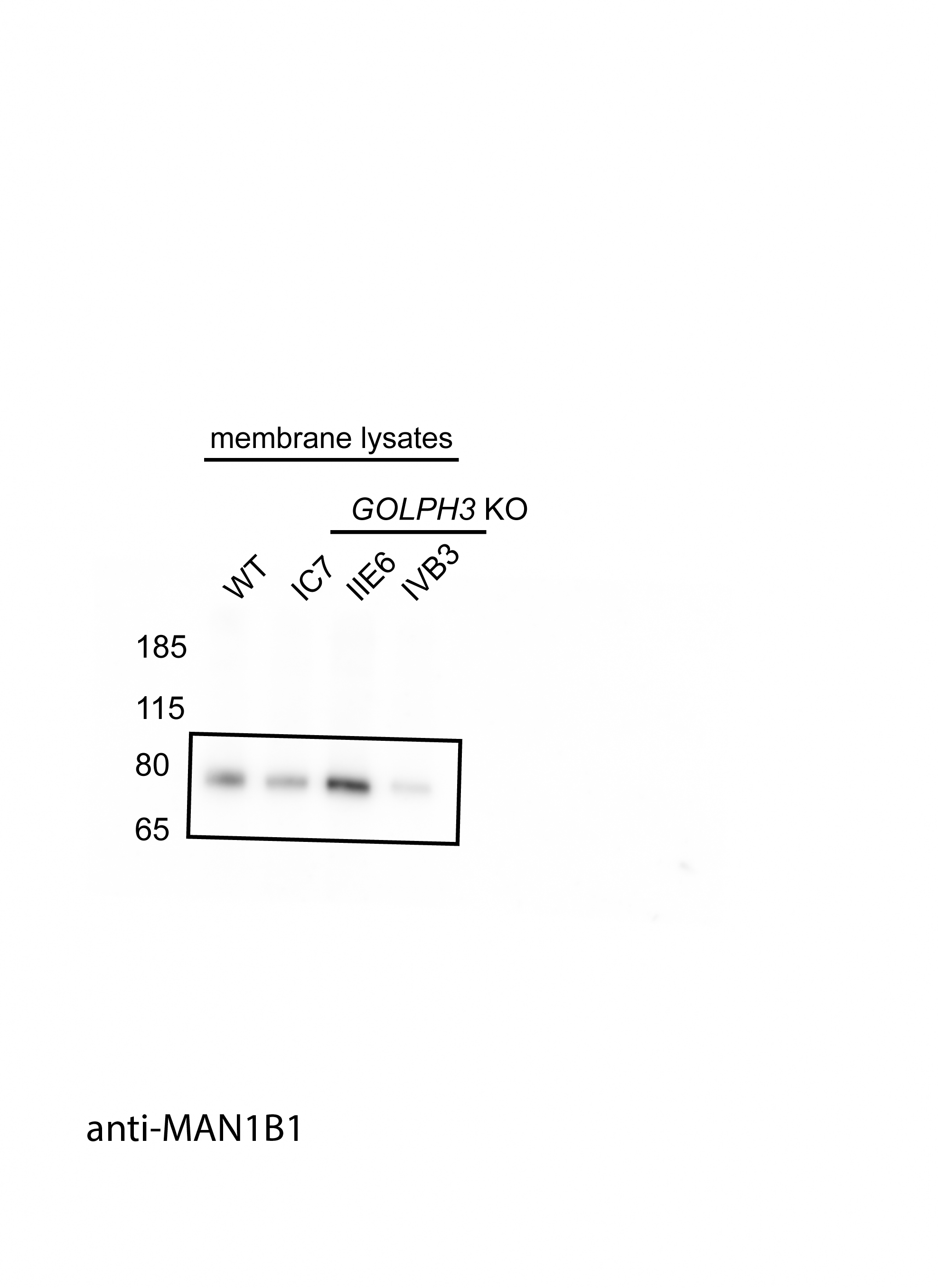

Supplement: Supplementary file 11 — Source data for Appendix [file 44318_2024_305_MOESM11_ESM.zip › Appendix/Appendix Figure S3/S3D/MAN1B1 8bit annotated 20240926_141418-02_Ch_Chemi-01.tif]

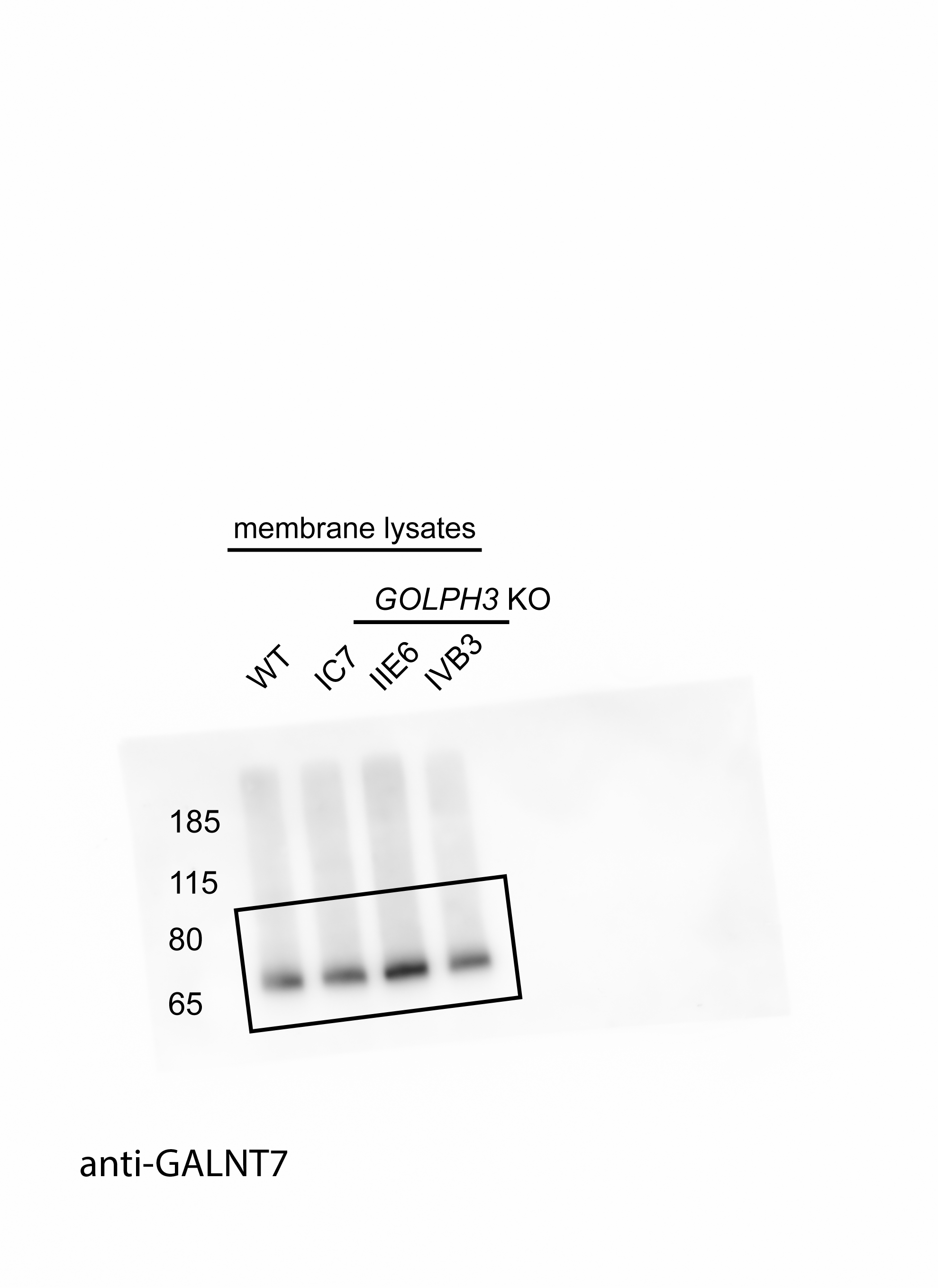

Supplement: Supplementary file 11 — Source data for Appendix [file 44318_2024_305_MOESM11_ESM.zip › Appendix/Appendix Figure S3/S3D/GALNT7 8bit annotated 20240927_142323-03_Ch_Chemi-01.tif]

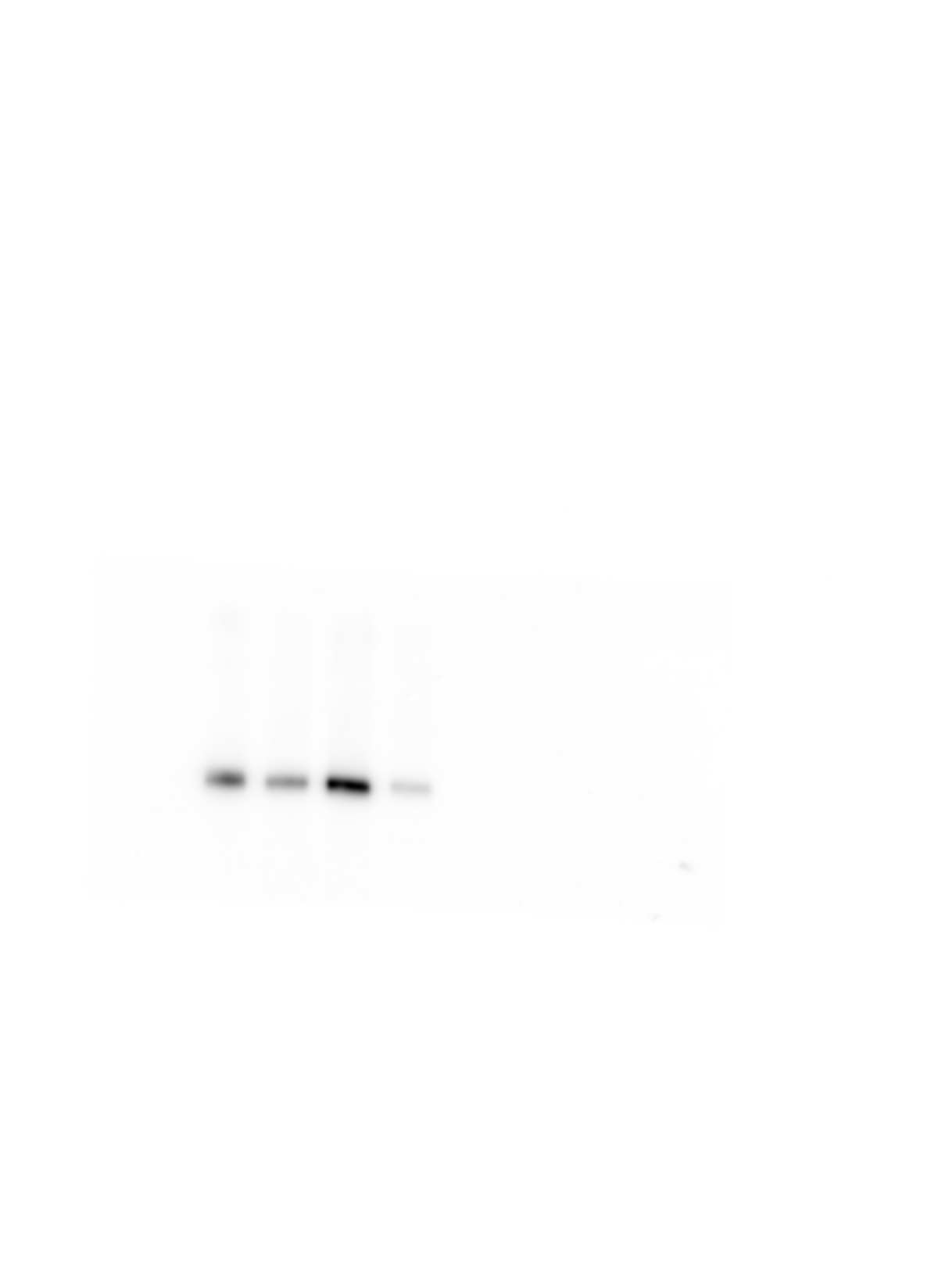

Supplement: Supplementary file 11 — Source data for Appendix [file 44318_2024_305_MOESM11_ESM.zip › Appendix/Appendix Figure S3/S3D/MAN1B1 16bit original 20240926_141418-02_Ch_Chemi.tif]

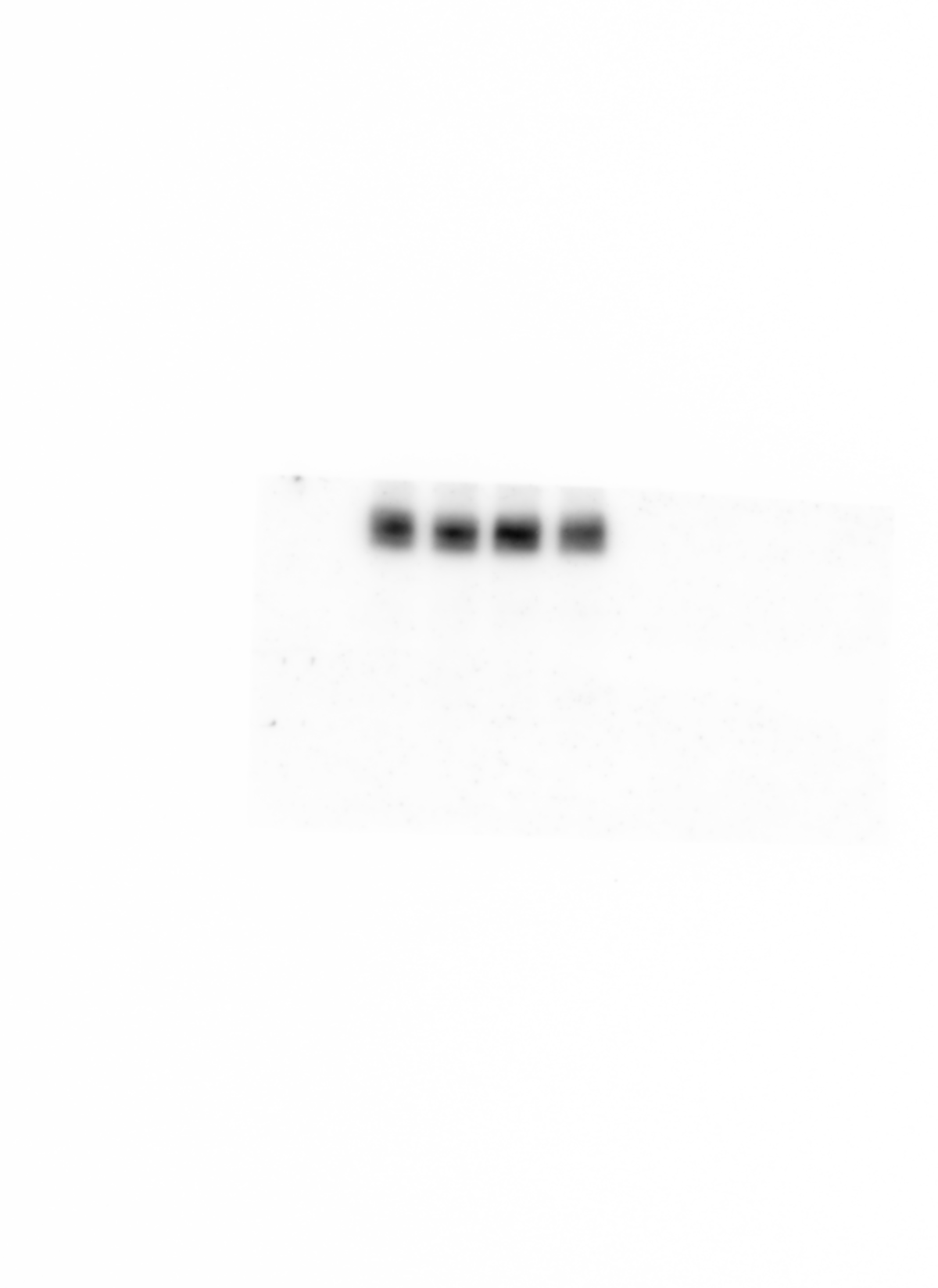

Supplement: Supplementary file 11 — Source data for Appendix [file 44318_2024_305_MOESM11_ESM.zip › Appendix/Appendix Figure S3/S3D/CANT1 16bit original 20240926_140623-10_Ch_Chemi.tif]

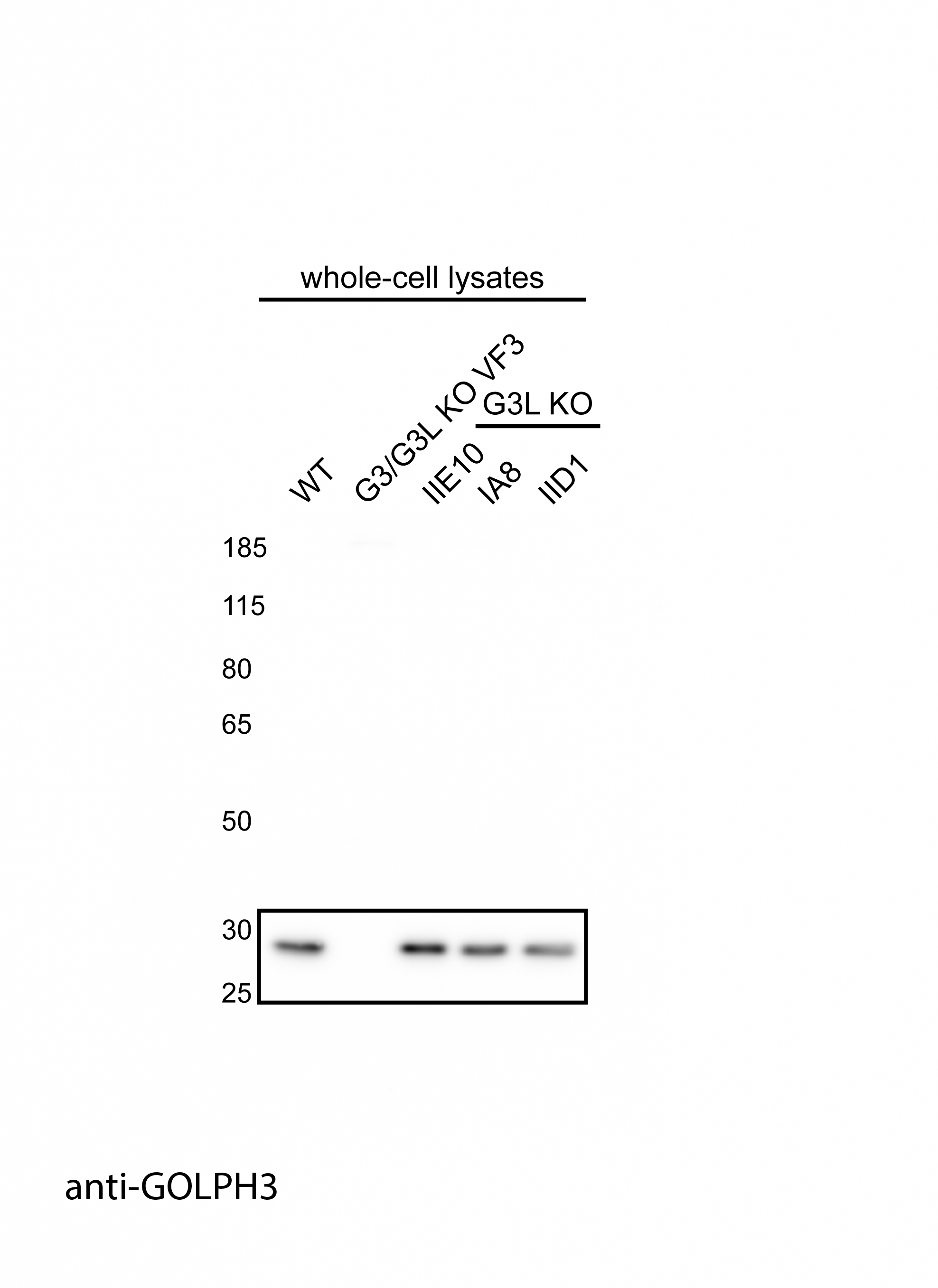

Supplement: Supplementary file 11 — Source data for Appendix [file 44318_2024_305_MOESM11_ESM.zip › Appendix/Appendix Figure S3/S3G/GOLPH3 cell lysate 8bit annotated 20240704_140943-04_Ch_Chemi-01.tif]

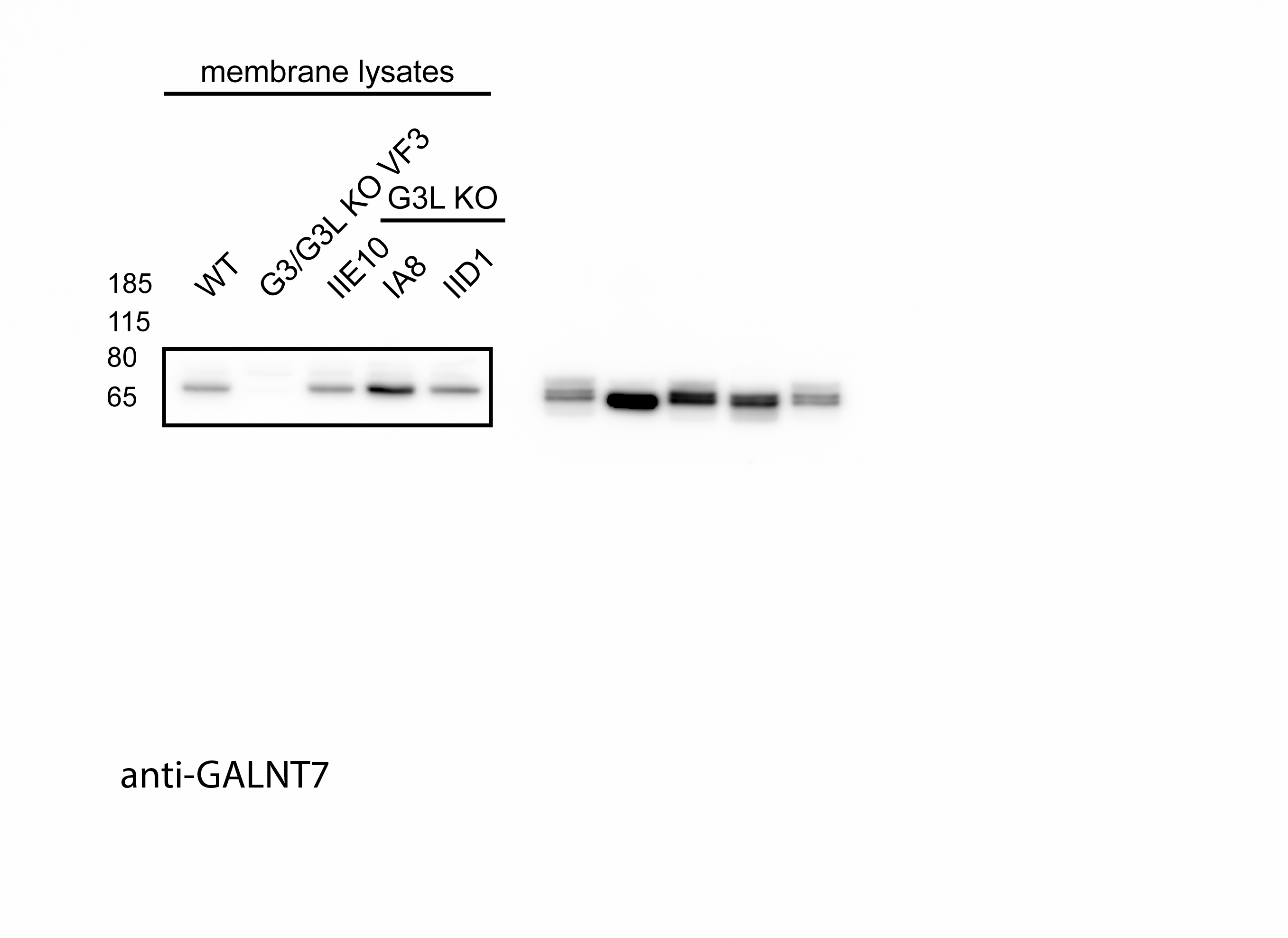

Supplement: Supplementary file 11 — Source data for Appendix [file 44318_2024_305_MOESM11_ESM.zip › Appendix/Appendix Figure S3/S3G/GALNT7 8bit annotated 20240704_133950-13_Ch_Chemi-01.tif]

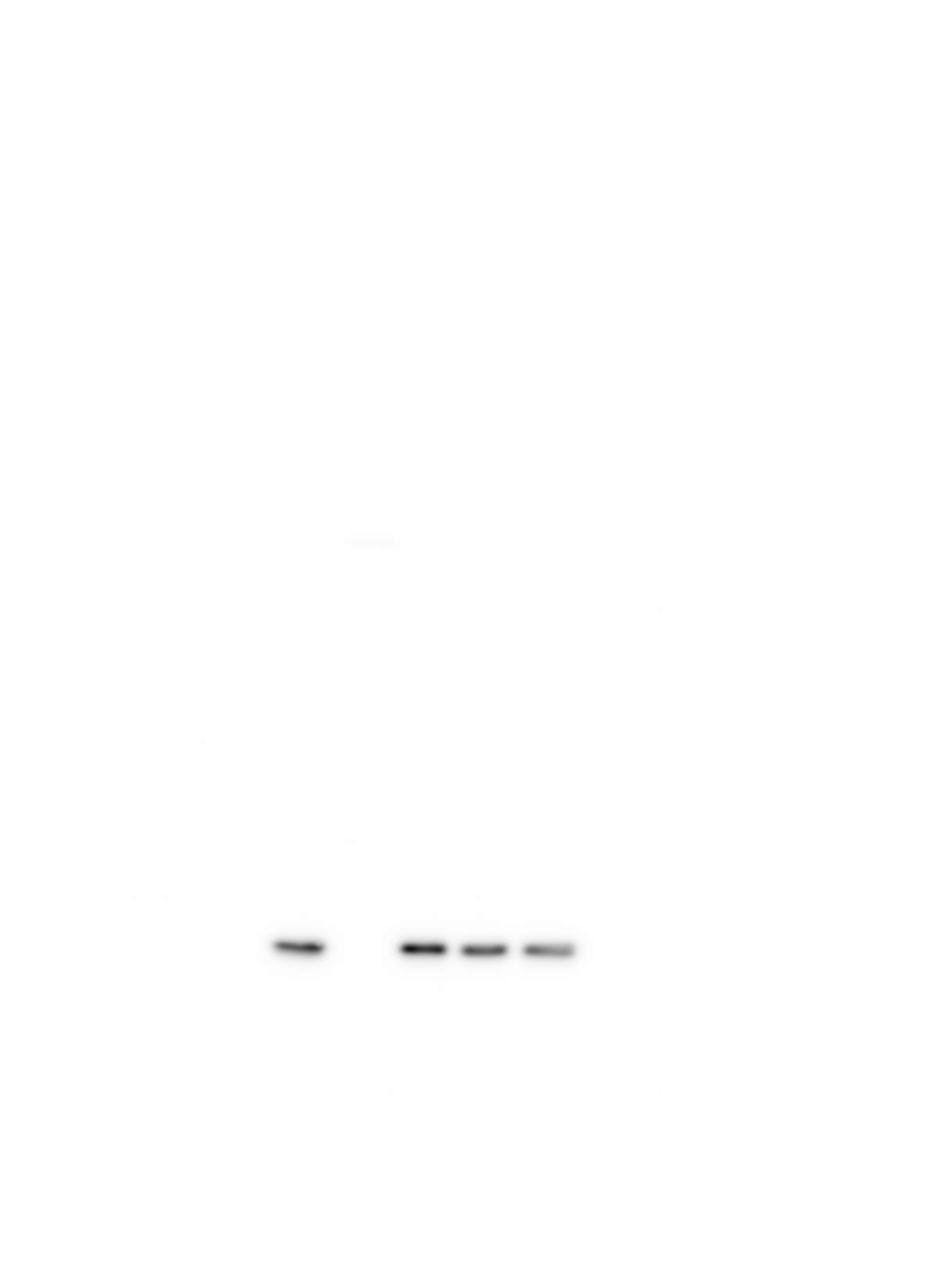

Supplement: Supplementary file 11 — Source data for Appendix [file 44318_2024_305_MOESM11_ESM.zip › Appendix/Appendix Figure S3/S3G/GOLPH3 cell lysate 16bit original 20240704_140943-04_Ch_Chemi.tif]

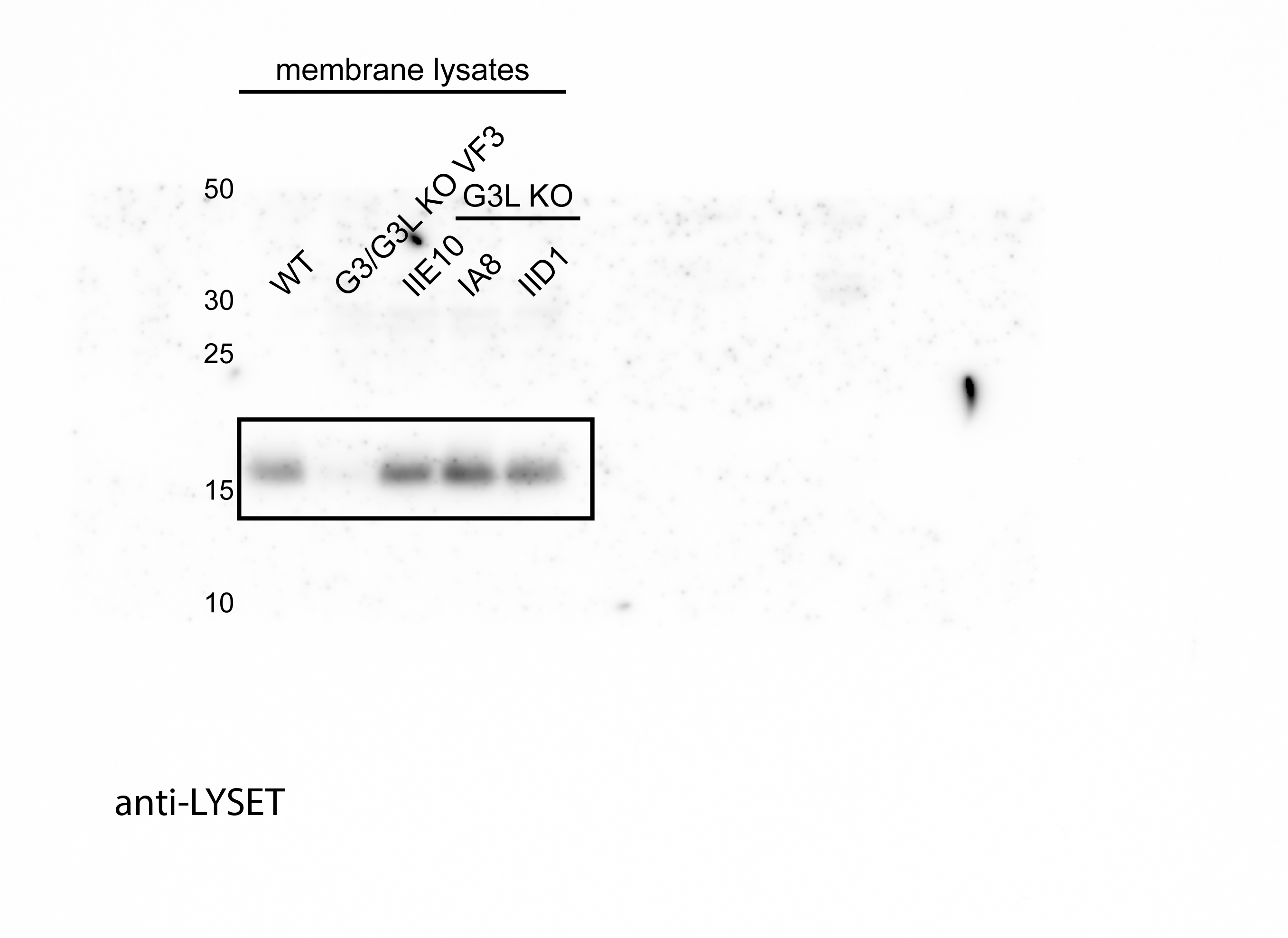

Supplement: Supplementary file 11 — Source data for Appendix [file 44318_2024_305_MOESM11_ESM.zip › Appendix/Appendix Figure S3/S3G/LYSET 8bit annotated20240704_141620-19_Ch_Chemi-01.tif]

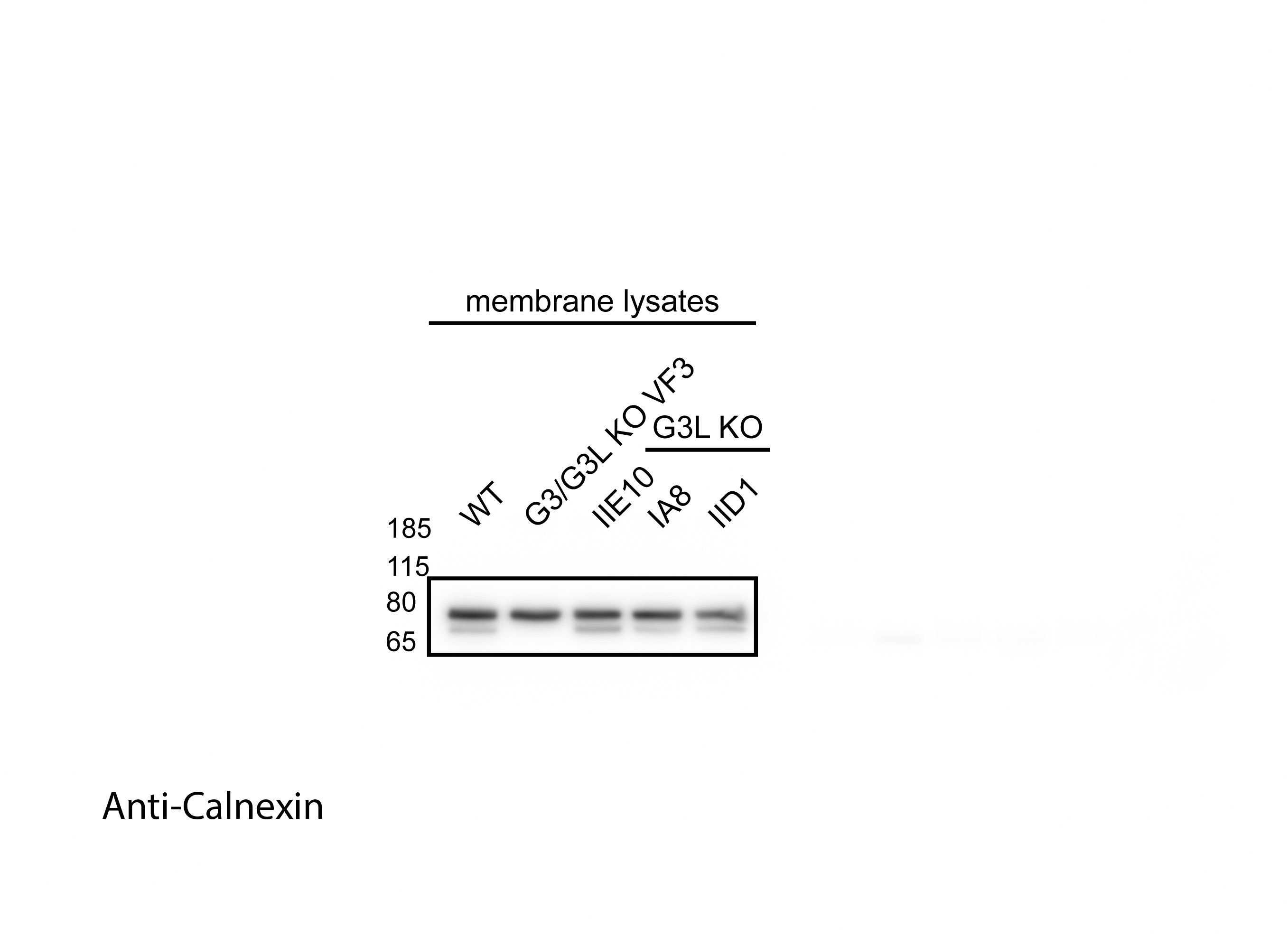

Supplement: Supplementary file 11 — Source data for Appendix [file 44318_2024_305_MOESM11_ESM.zip › Appendix/Appendix Figure S3/S3G/Calnexin membrane lysate for GALNT7 LYSET 8bit annotated 20240709_144024-02_Ch_Chemi-01.tif]

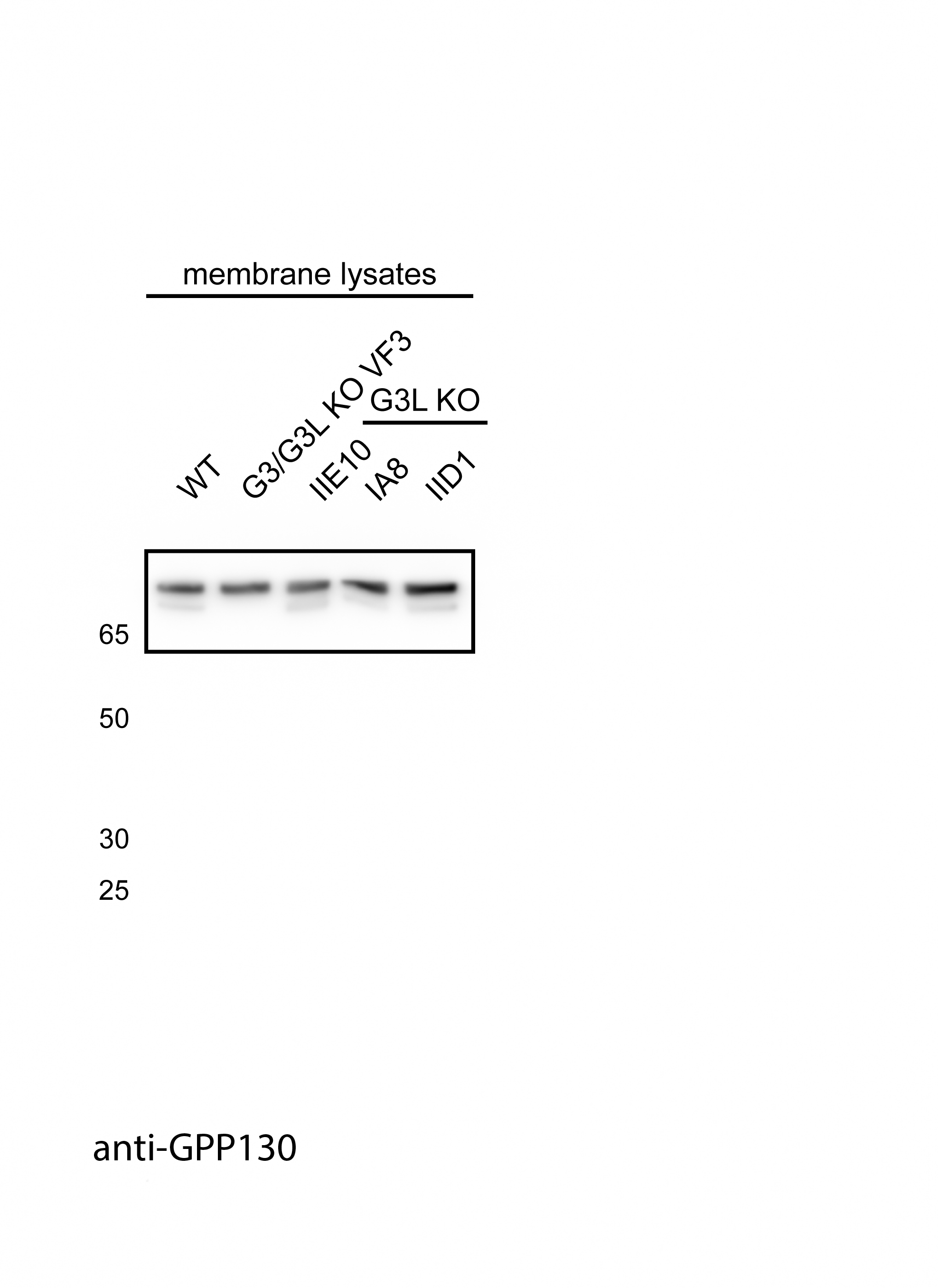

Supplement: Supplementary file 11 — Source data for Appendix [file 44318_2024_305_MOESM11_ESM.zip › Appendix/Appendix Figure S3/S3G/Calnexin for GPP130 8bit annotated 20240709_143157-02_Ch_Chemi-01.tif]

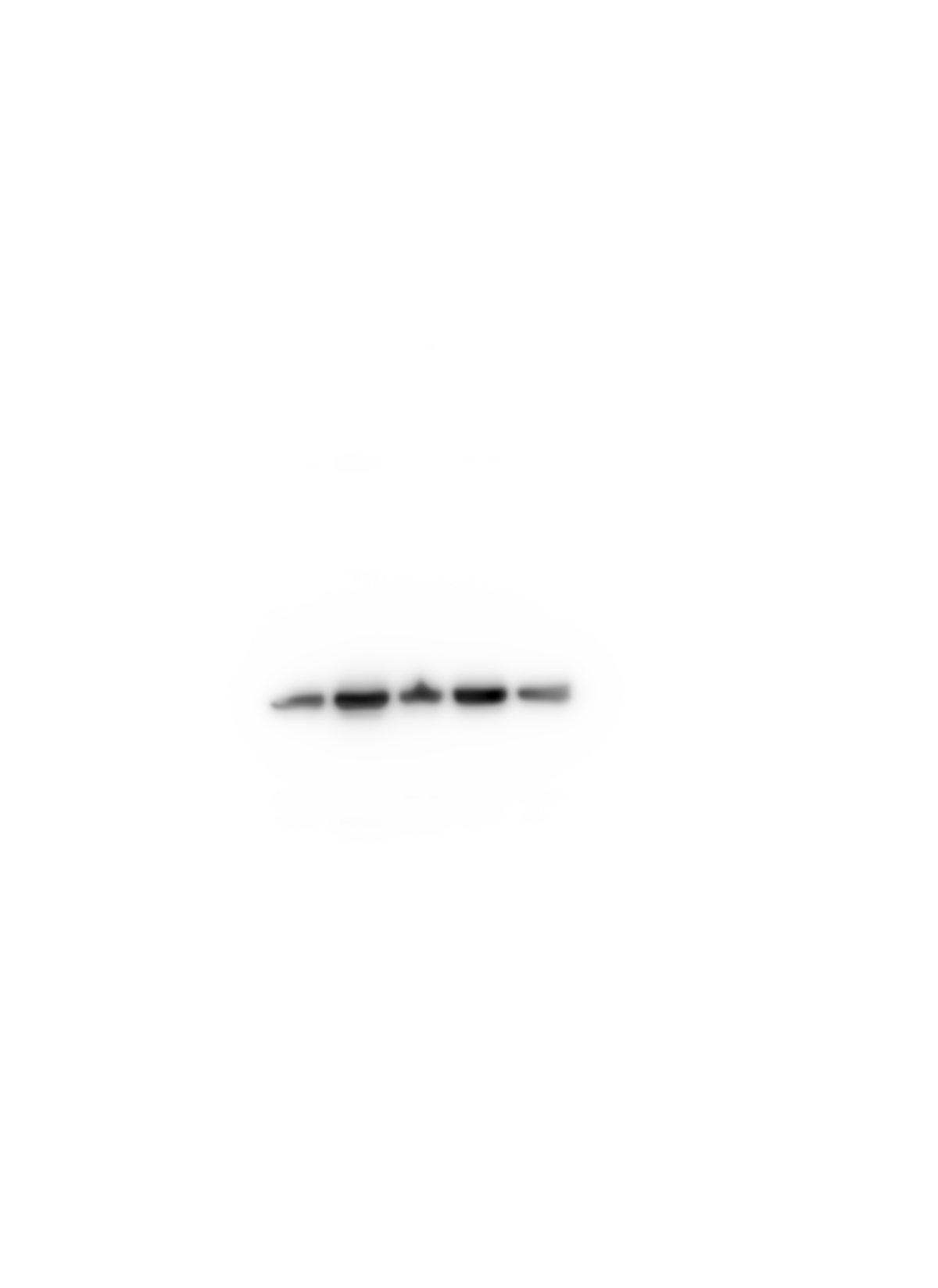

Supplement: Supplementary file 11 — Source data for Appendix [file 44318_2024_305_MOESM11_ESM.zip › Appendix/Appendix Figure S3/S3G/Tubulin cell ysates for GOLPH3 16bit original 20241001_121553-01_Ch_Chemi.tif]

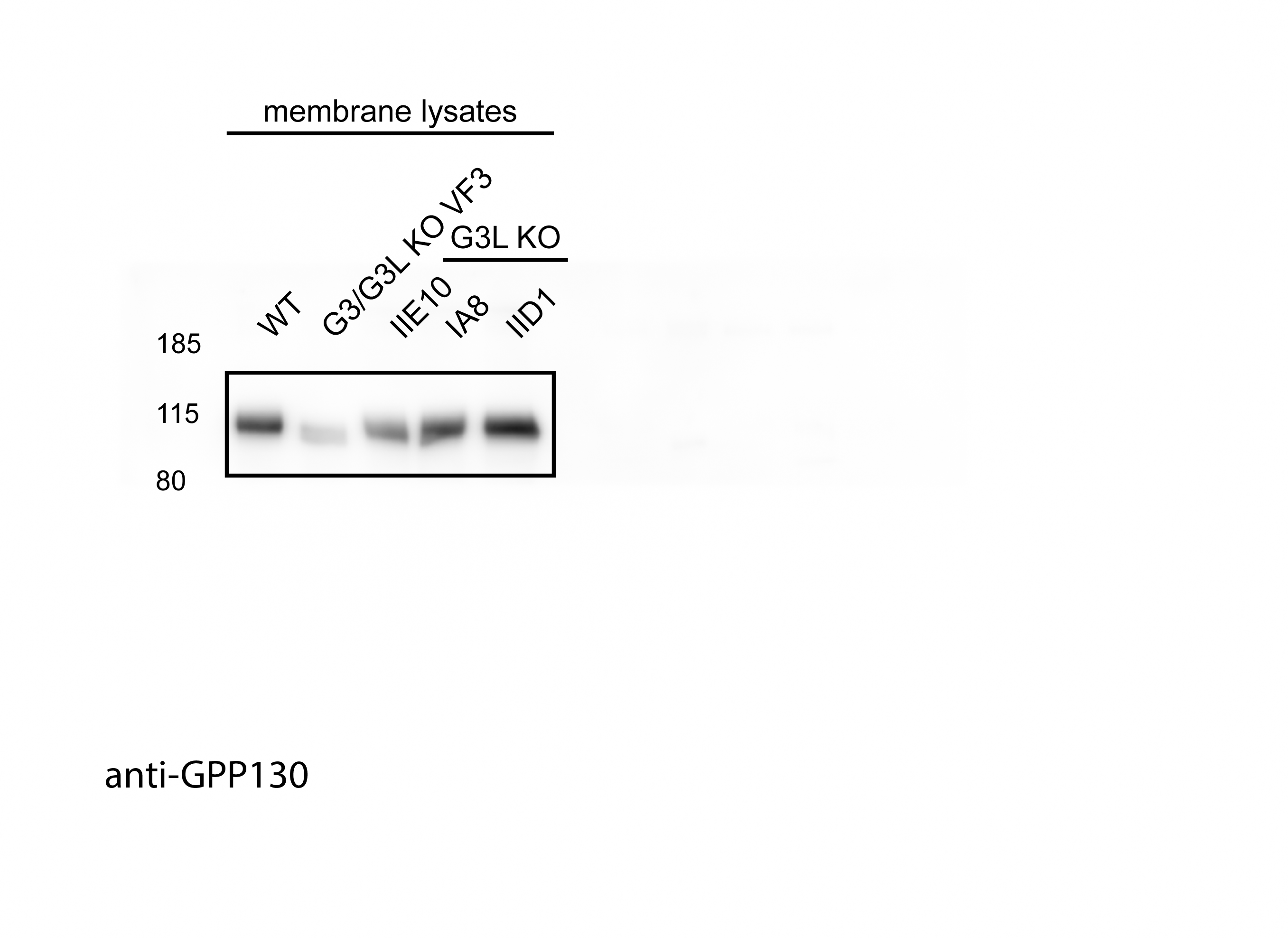

Supplement: Supplementary file 11 — Source data for Appendix [file 44318_2024_305_MOESM11_ESM.zip › Appendix/Appendix Figure S3/S3G/GPP130 8bit annotated 20240704_135037_Ch_Chemi-01.tif]

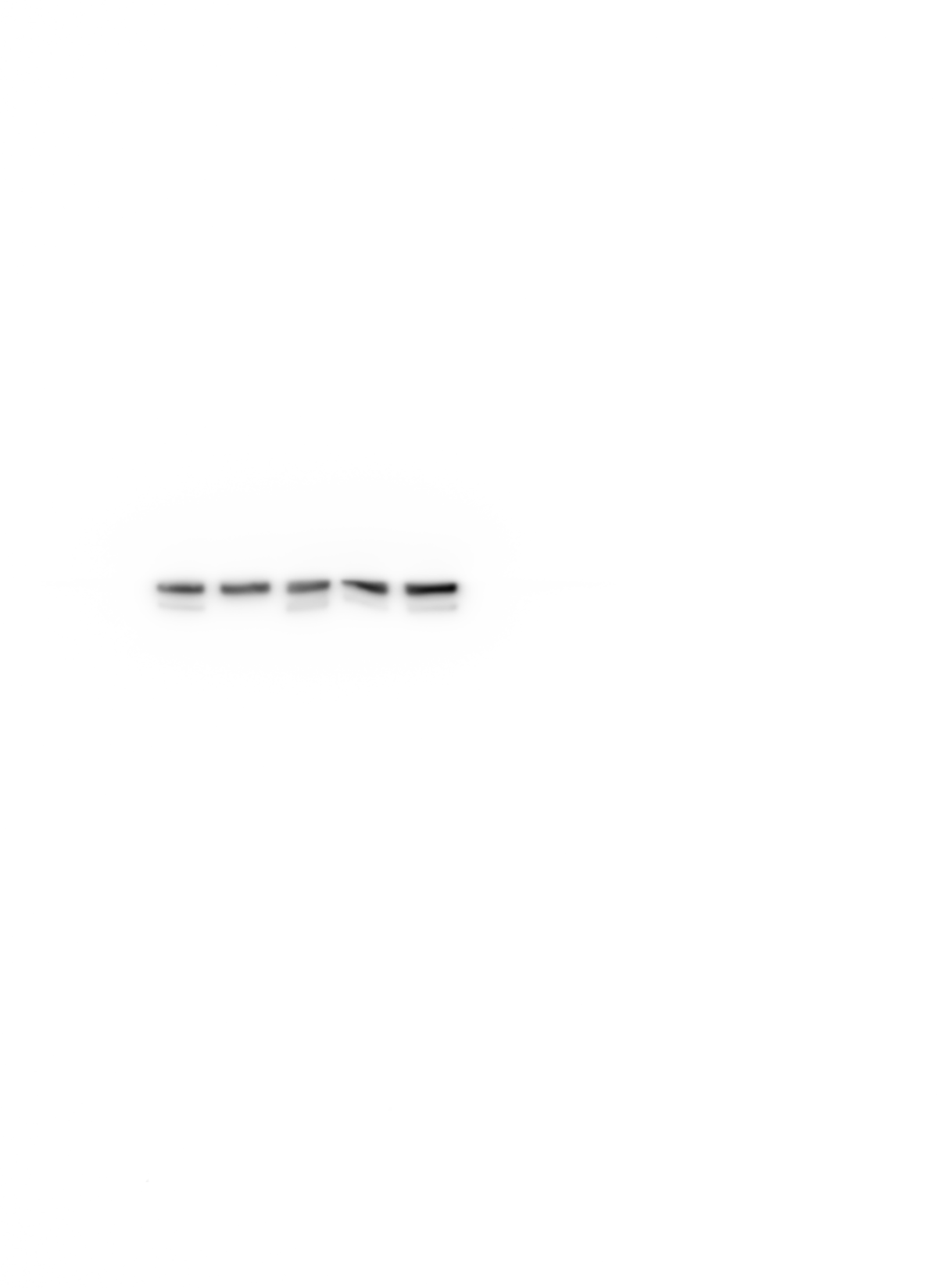

Supplement: Supplementary file 11 — Source data for Appendix [file 44318_2024_305_MOESM11_ESM.zip › Appendix/Appendix Figure S3/S3G/Calnexin for GPP130 16bit original 20240709_143157-02_Ch_Chemi.tif]

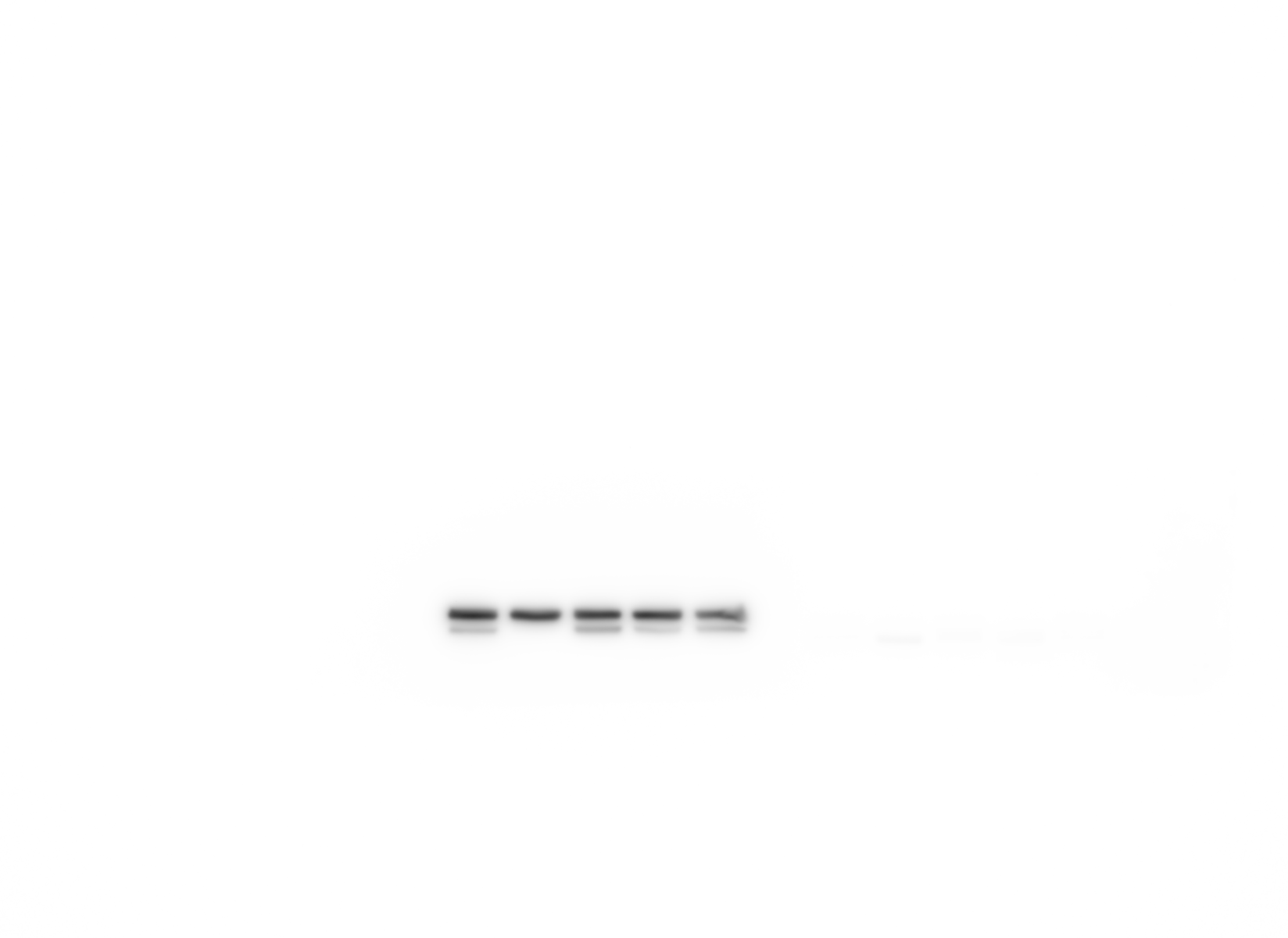

Supplement: Supplementary file 11 — Source data for Appendix [file 44318_2024_305_MOESM11_ESM.zip › Appendix/Appendix Figure S3/S3G/Calnexin membrane lysate 16bit original for GALNT7 LYSET 20240709_144024-02_Ch_Chemi.tif]

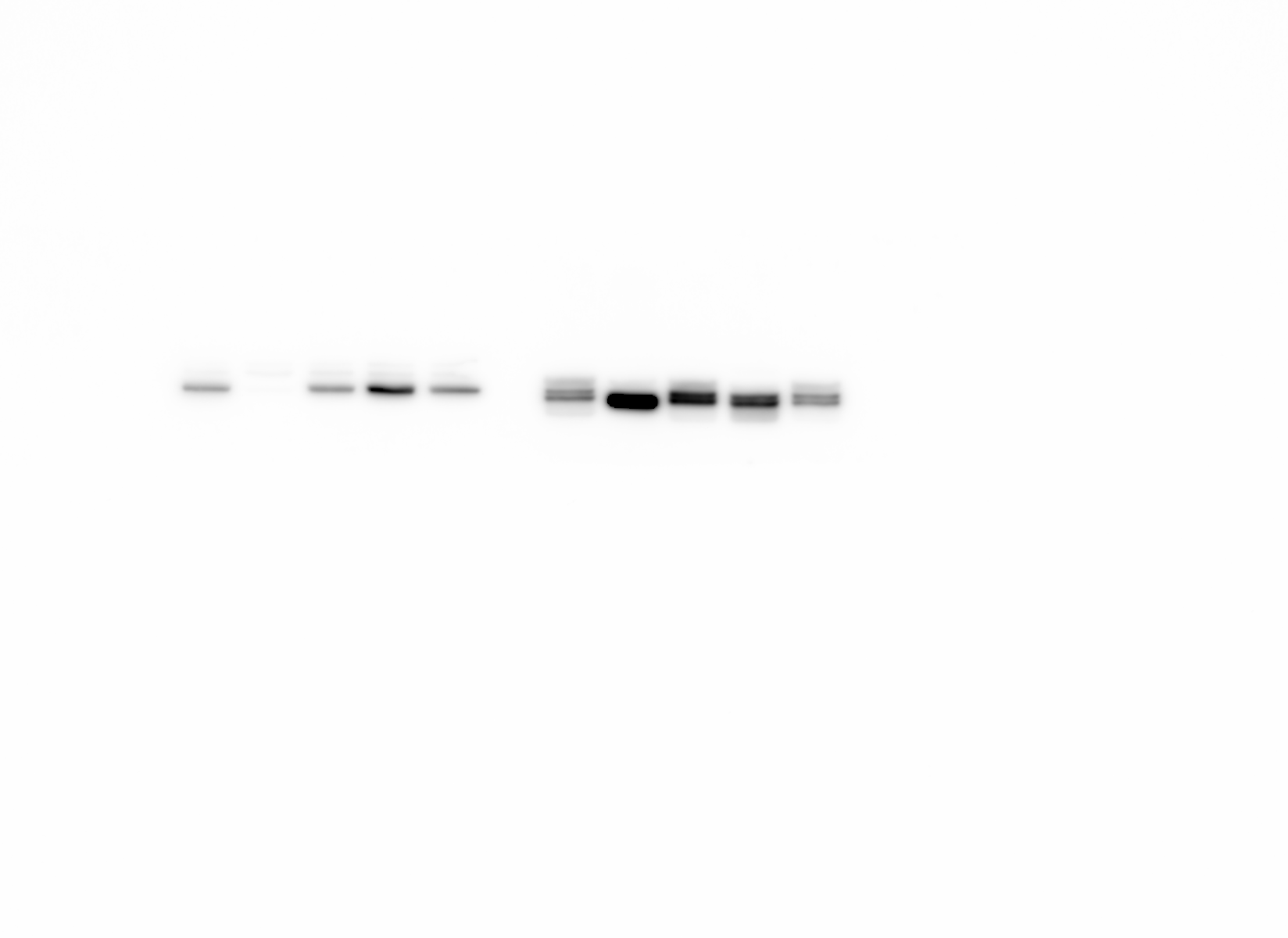

Supplement: Supplementary file 11 — Source data for Appendix [file 44318_2024_305_MOESM11_ESM.zip › Appendix/Appendix Figure S3/S3G/GALNT7 16bit original 20240704_133950-13_Ch_Chemi.tif]

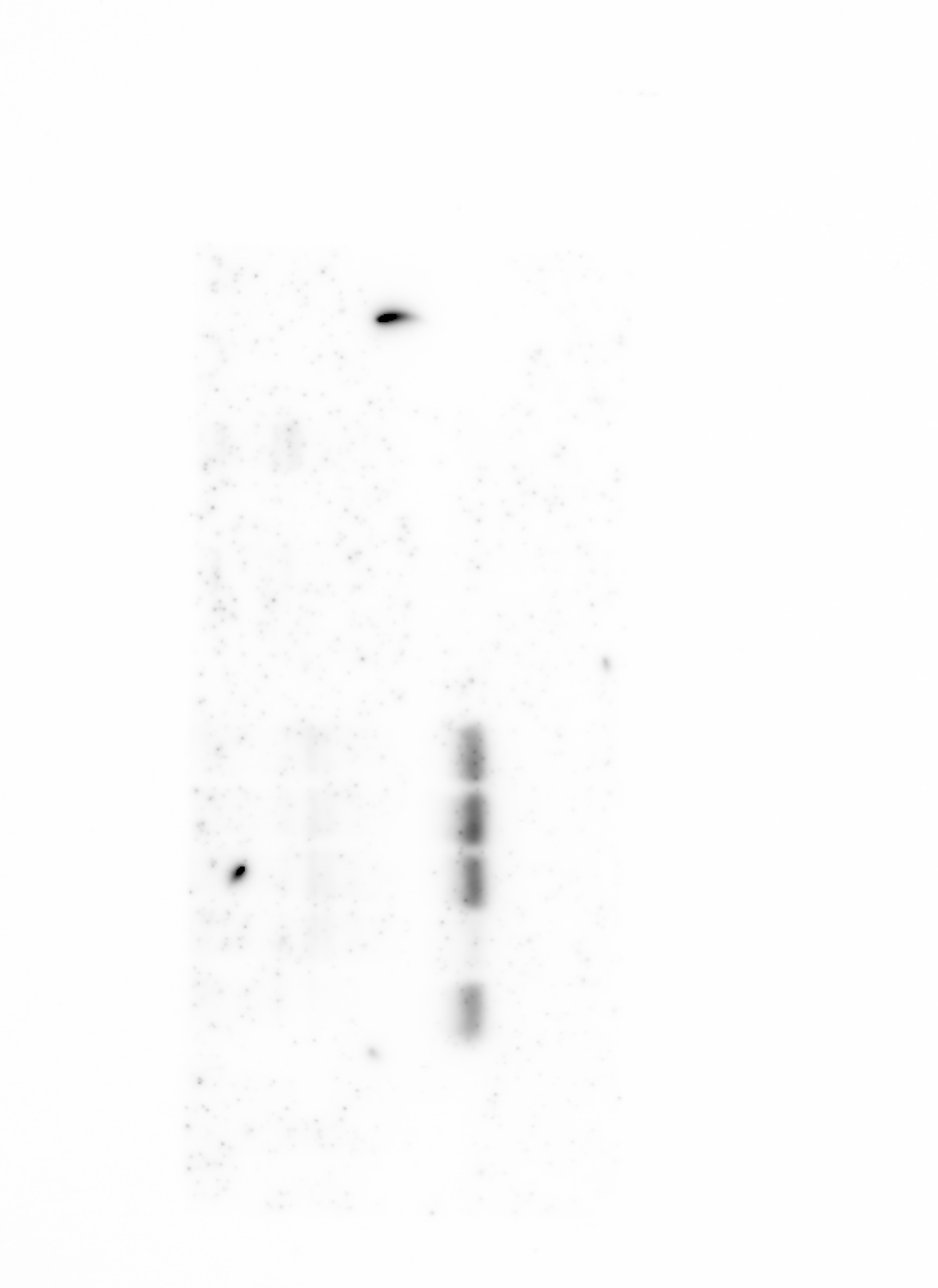

Supplement: Supplementary file 11 — Source data for Appendix [file 44318_2024_305_MOESM11_ESM.zip › Appendix/Appendix Figure S3/S3G/LYSET 16bit original 20240704_141620-19_Ch_Chemi.tif]

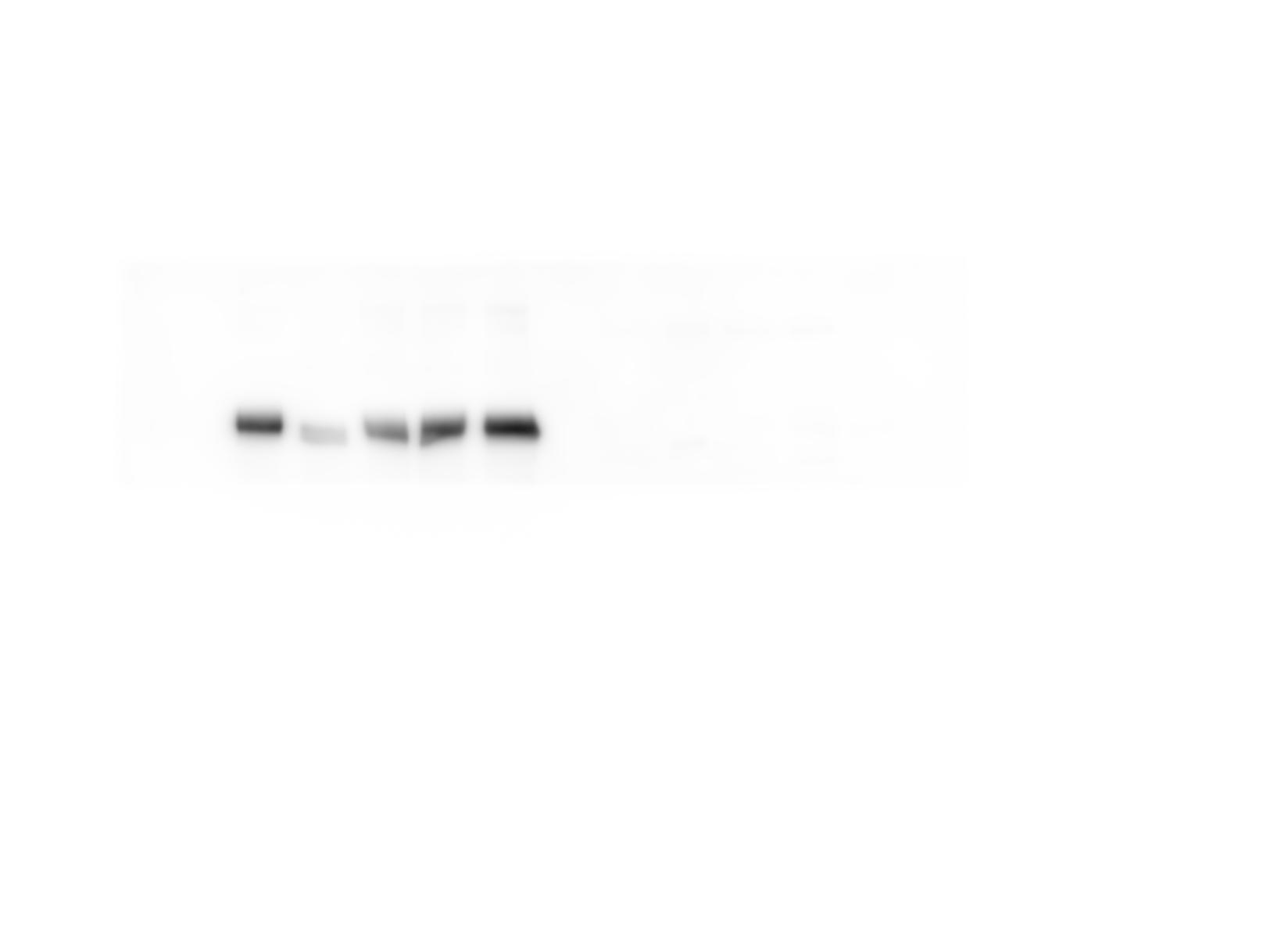

Supplement: Supplementary file 11 — Source data for Appendix [file 44318_2024_305_MOESM11_ESM.zip › Appendix/Appendix Figure S3/S3G/GPP130 16bit original 20240704_135037_Ch_Chemi.tif]

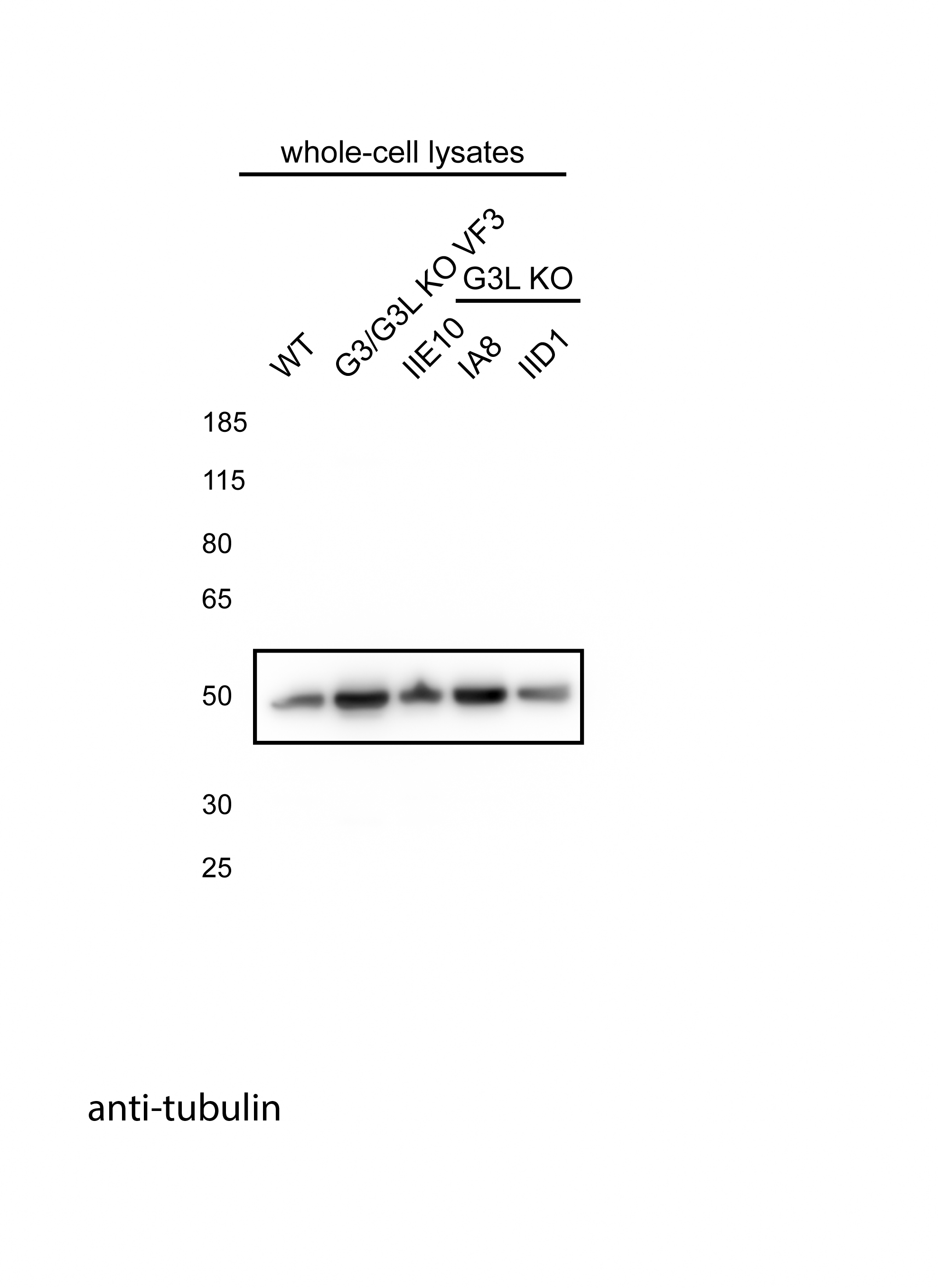

Supplement: Supplementary file 11 — Source data for Appendix [file 44318_2024_305_MOESM11_ESM.zip › Appendix/Appendix Figure S3/S3G/tubulin cell lysate for GOLPH3 8bit annotated 20241001_121553-01_Ch_Chemi-01.tif]

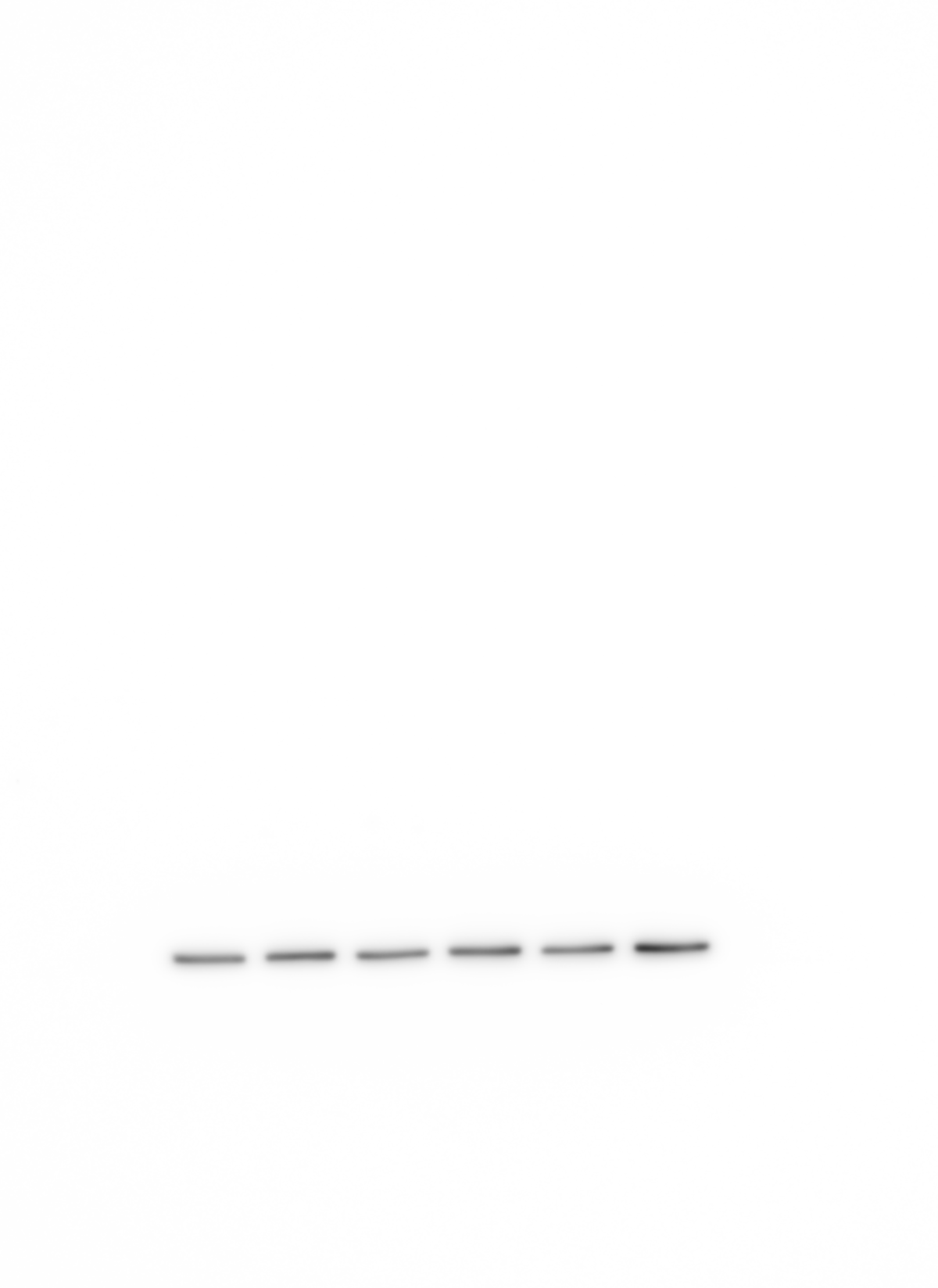

Supplement: Supplementary file 11 — Source data for Appendix [file 44318_2024_305_MOESM11_ESM.zip › Appendix/Appendix Figure S2/S2E/Calnexin lysate for B4GALT1 16bit original 20240319_124524-03_Ch_Chemi.tif]

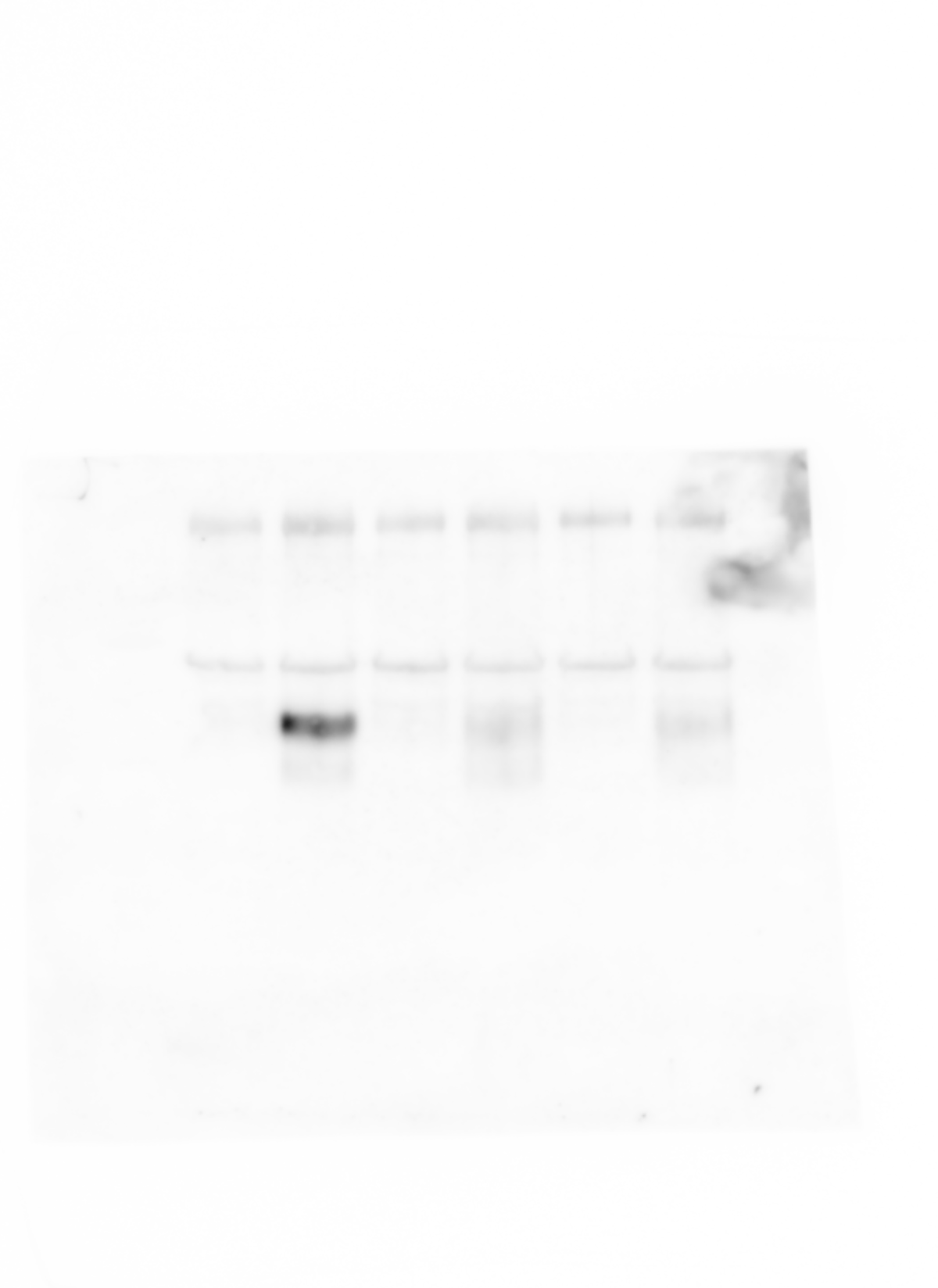

Supplement: Supplementary file 11 — Source data for Appendix [file 44318_2024_305_MOESM11_ESM.zip › Appendix/Appendix Figure S2/S2E/B4GALT5 16bit original 20240215_134424-05_Ch_Chemi.tif]

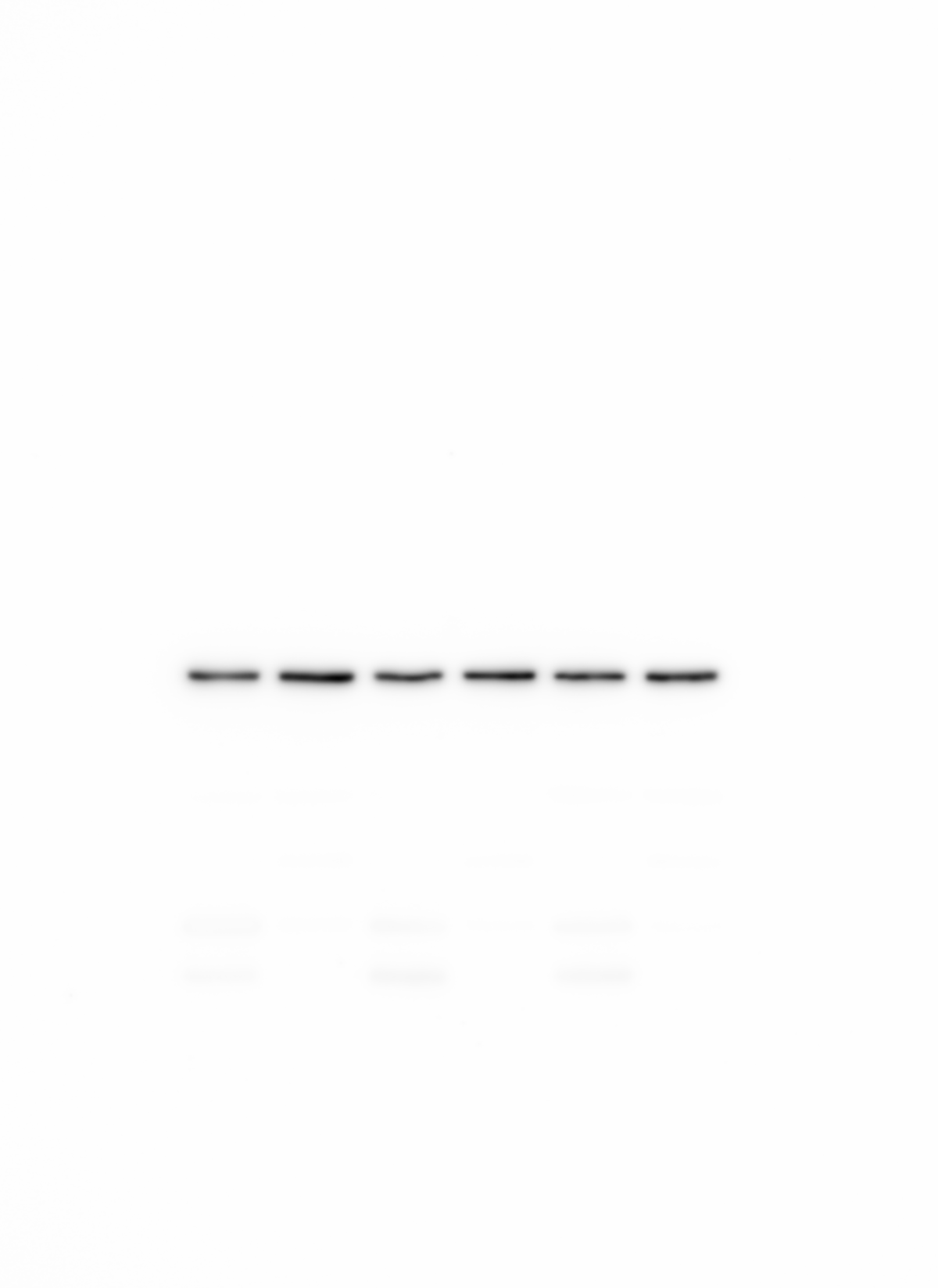

Supplement: Supplementary file 11 — Source data for Appendix [file 44318_2024_305_MOESM11_ESM.zip › Appendix/Appendix Figure S2/S2E/Calnexin for cathepsin L lysate 16bit original 20240319_132738-08_Ch_Chemi.tif]

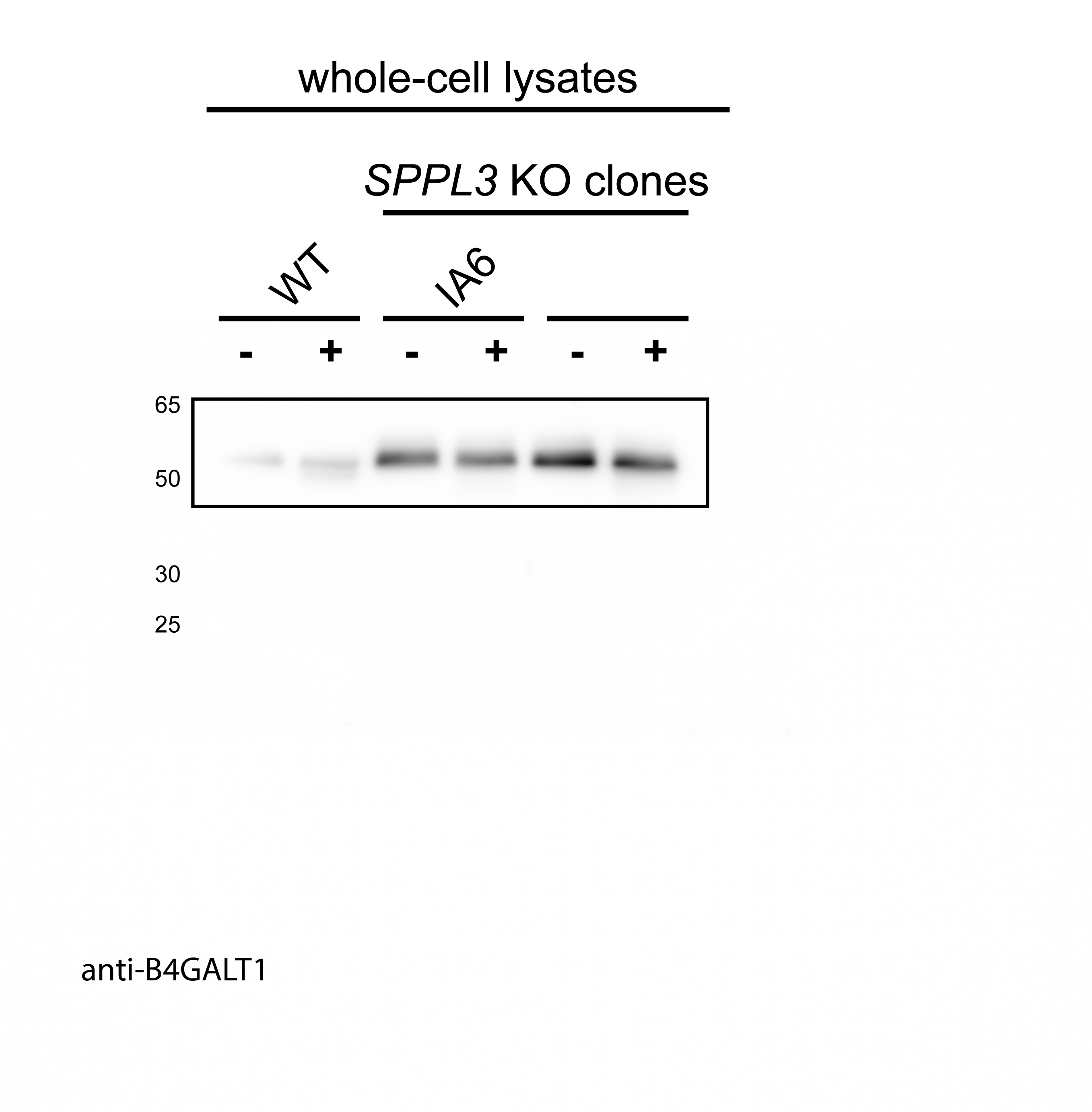

Supplement: Supplementary file 11 — Source data for Appendix [file 44318_2024_305_MOESM11_ESM.zip › Appendix/Appendix Figure S2/S2E/B4GALT1 lysate 8bit annotated 20240227_151818-04_Ch_Chemi-01.tif]

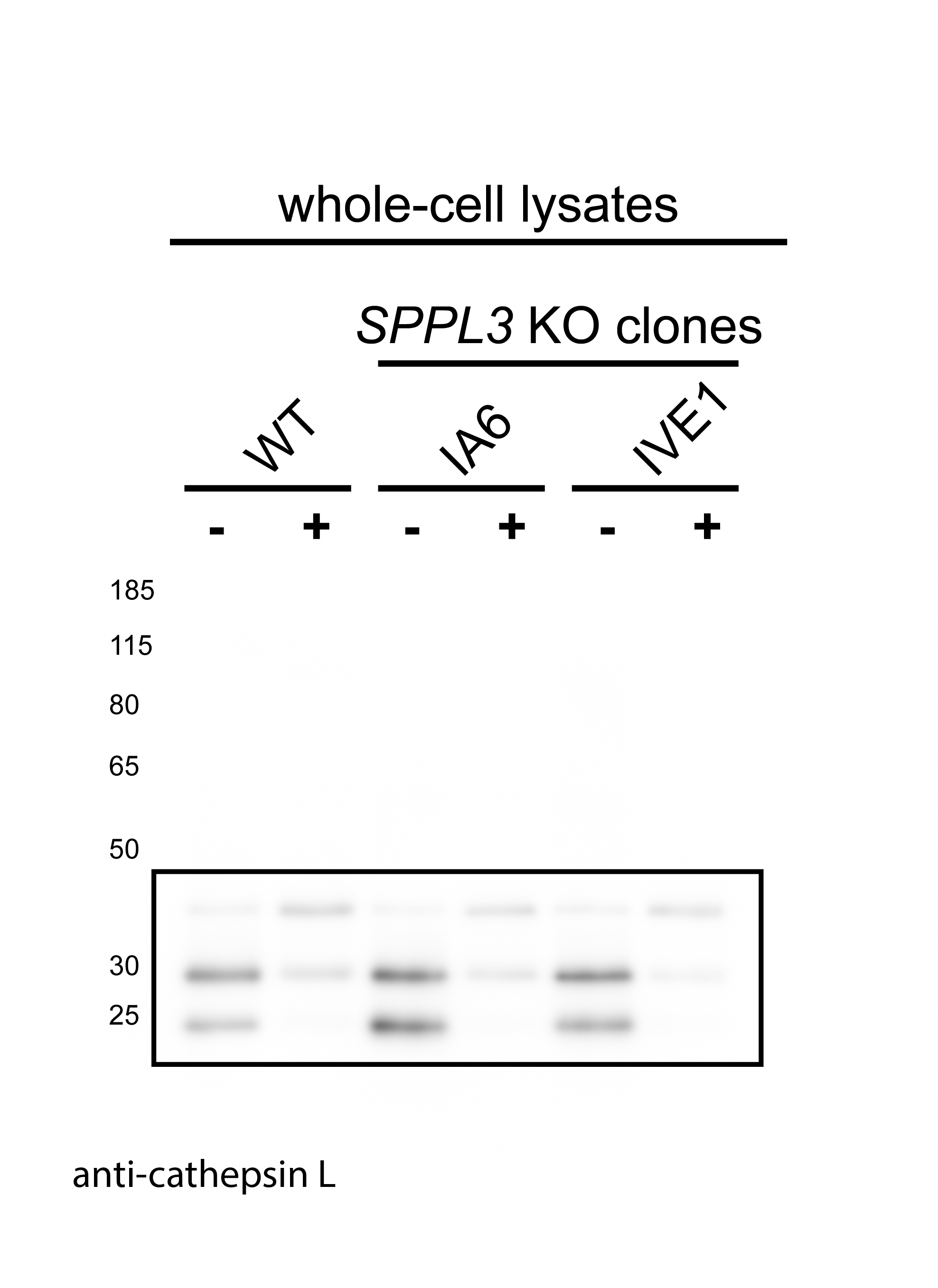

Supplement: Supplementary file 11 — Source data for Appendix [file 44318_2024_305_MOESM11_ESM.zip › Appendix/Appendix Figure S2/S2E/cathepsin L lysates 8bit annoated 20240227_150014_Ch_Chemi-01.tif]

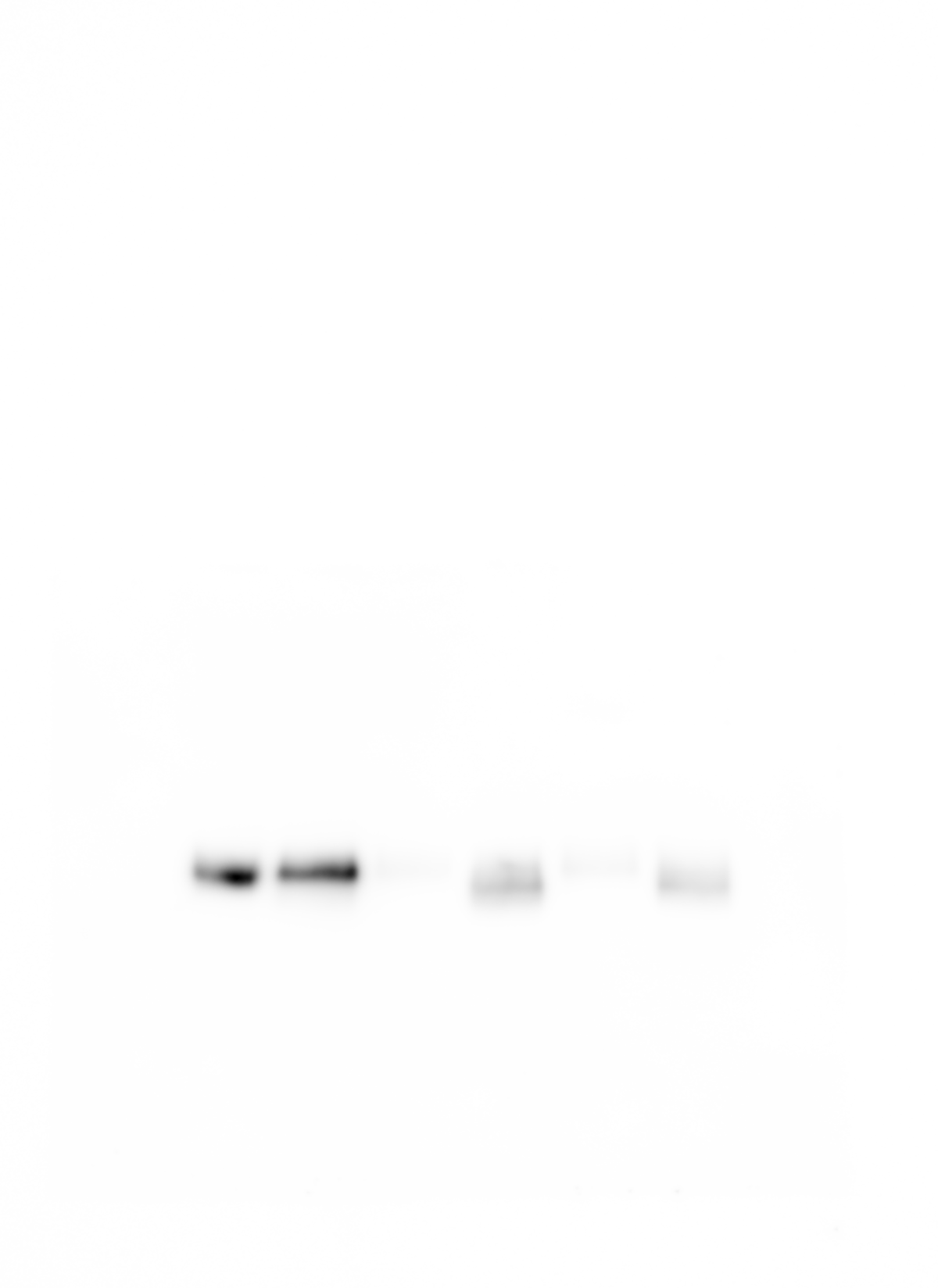

Supplement: Supplementary file 11 — Source data for Appendix [file 44318_2024_305_MOESM11_ESM.zip › Appendix/Appendix Figure S2/S2E/B4GALT1 supernatant 16bit original 20240220_151102-03_Ch_Chemi.tif]

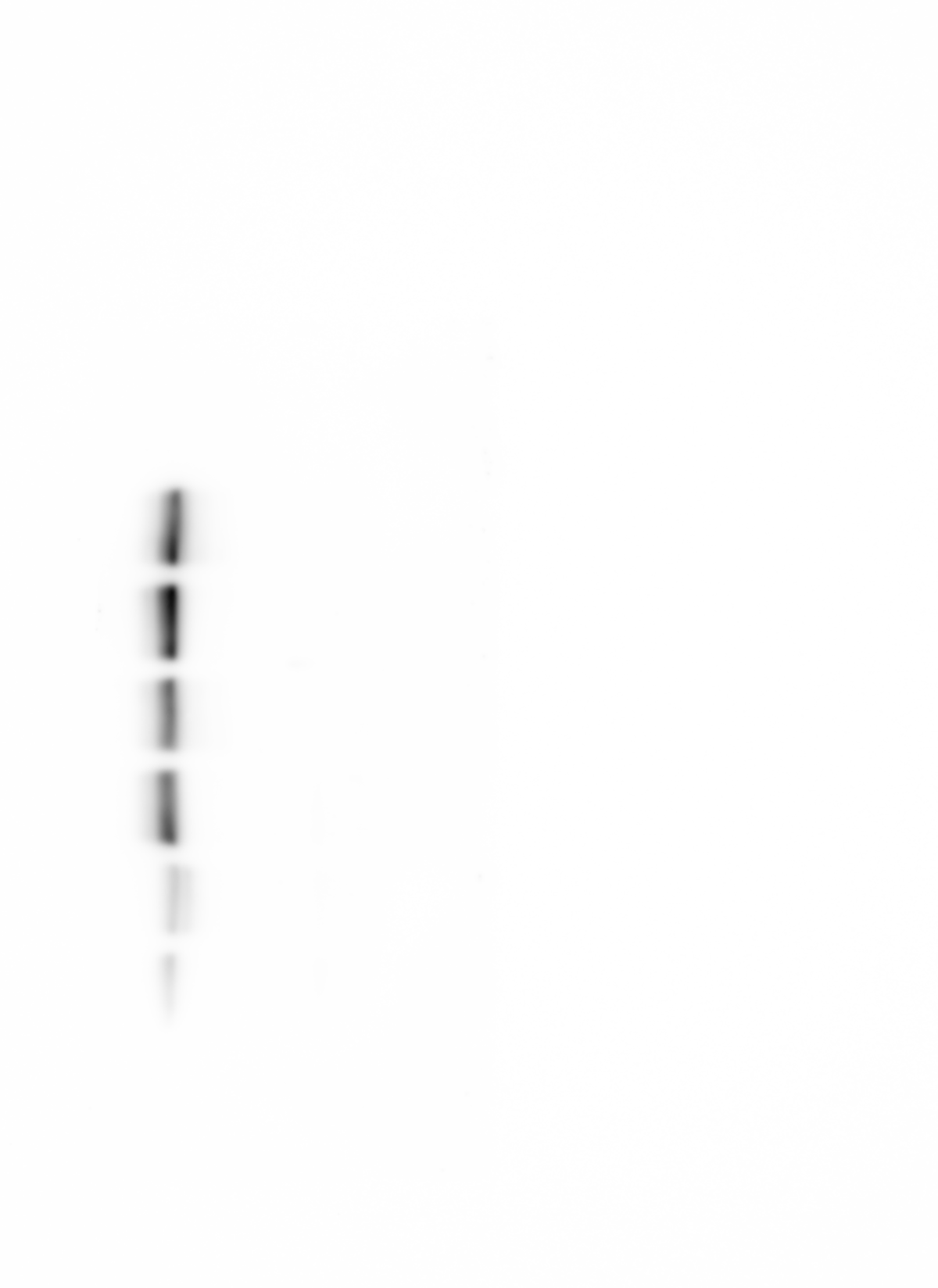

Supplement: Supplementary file 11 — Source data for Appendix [file 44318_2024_305_MOESM11_ESM.zip › Appendix/Appendix Figure S2/S2E/B4GALT1 lysate 16bit original 20240227_151818-04_Ch_Chemi.tif]

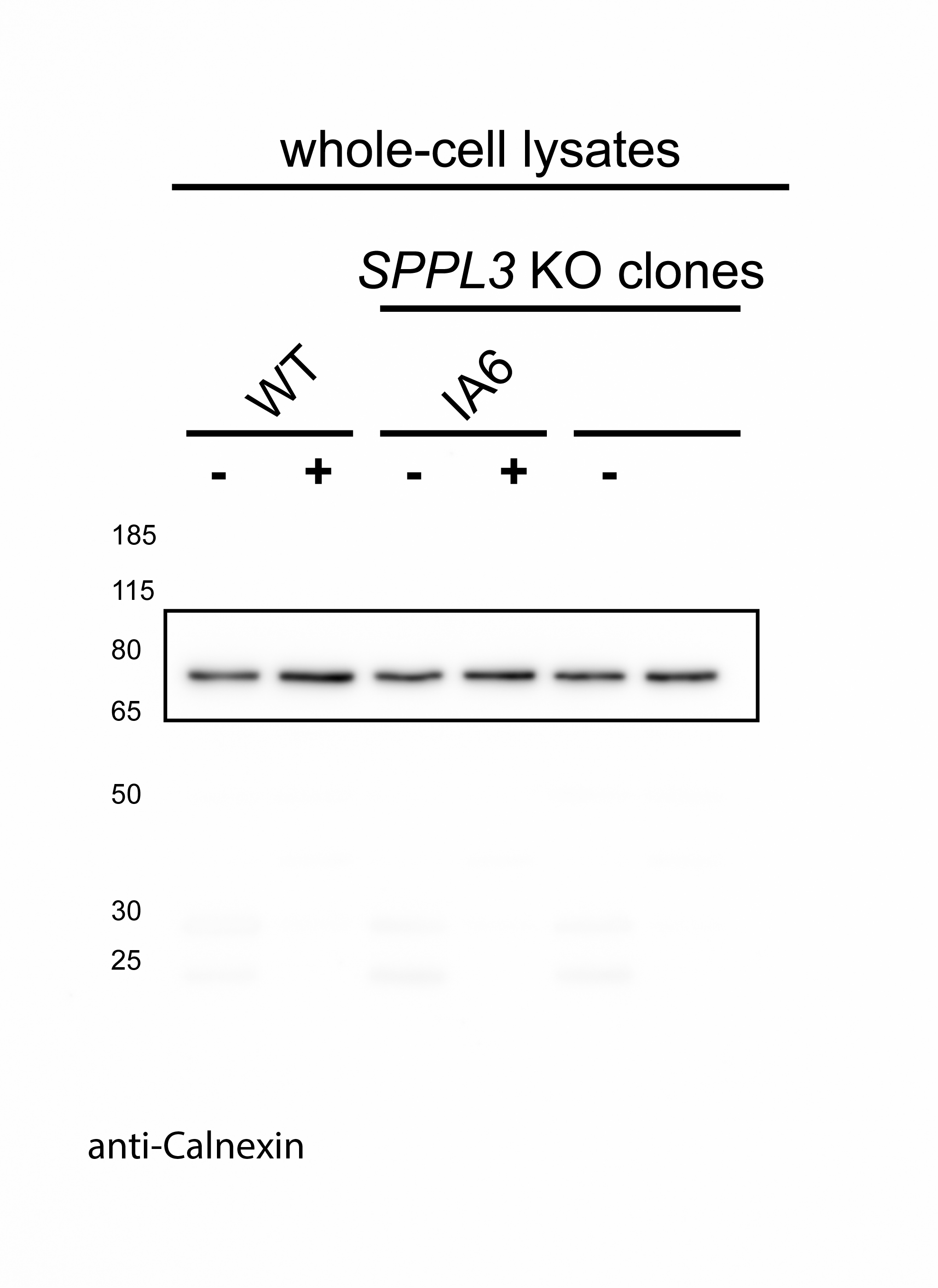

Supplement: Supplementary file 11 — Source data for Appendix [file 44318_2024_305_MOESM11_ESM.zip › Appendix/Appendix Figure S2/S2E/calnexin for cathepsin lysate 8bit annotated 20240319_132738-08_Ch_Chemi-01.tif]

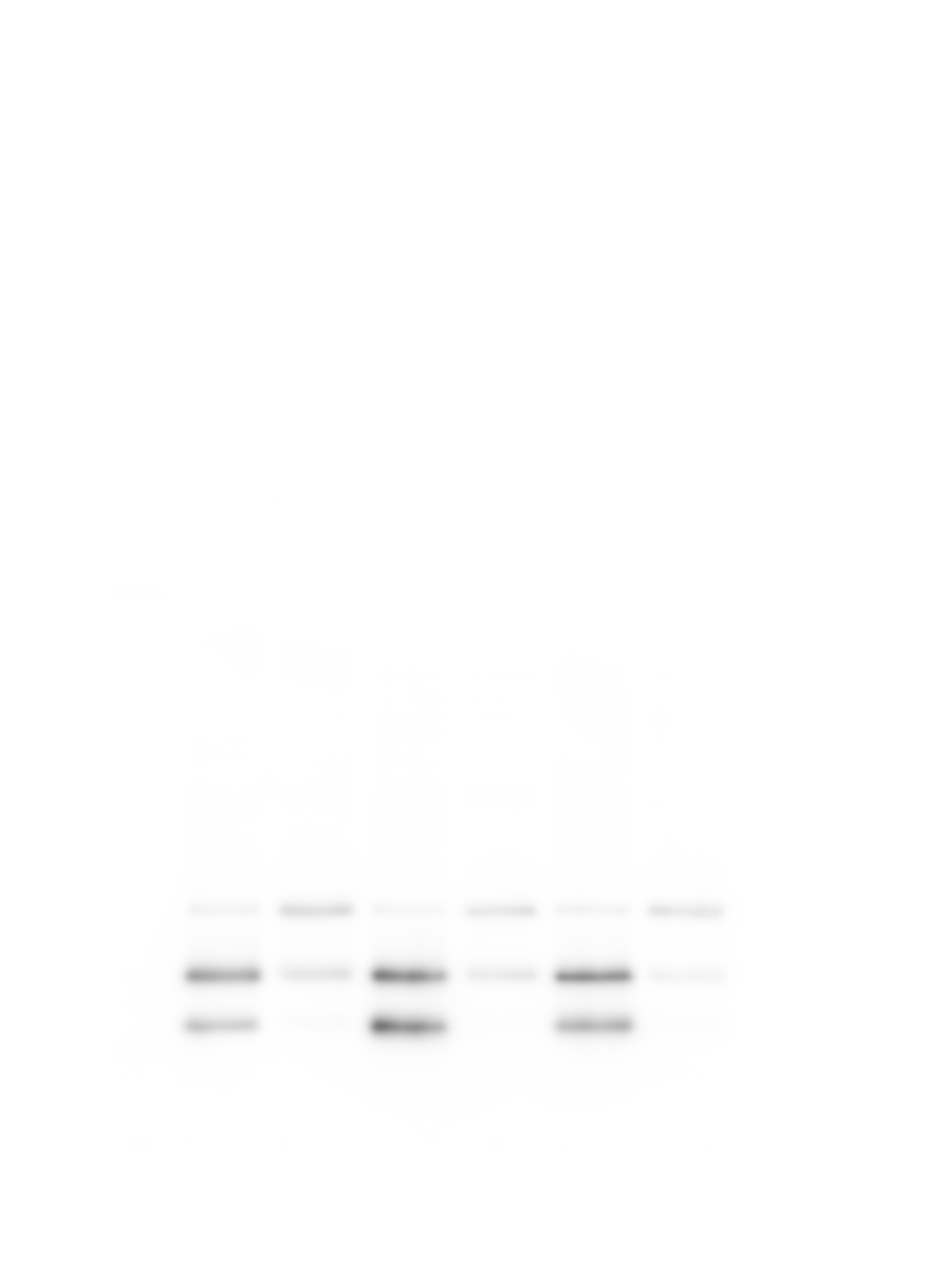

Supplement: Supplementary file 11 — Source data for Appendix [file 44318_2024_305_MOESM11_ESM.zip › Appendix/Appendix Figure S2/S2E/Cathepsin L lysates 16bit original 20240227_150014_Ch_Chemi.tif]

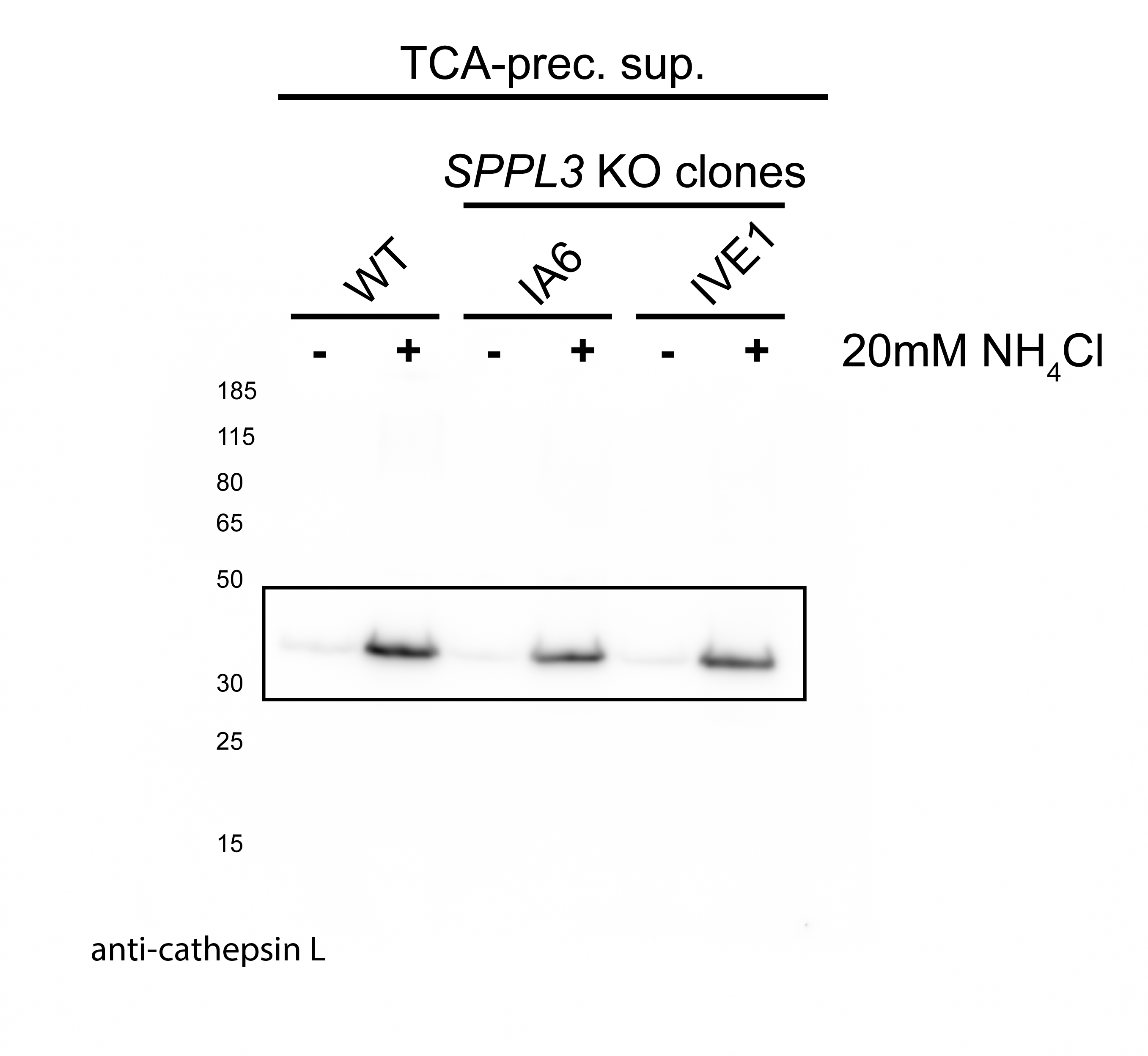

Supplement: Supplementary file 11 — Source data for Appendix [file 44318_2024_305_MOESM11_ESM.zip › Appendix/Appendix Figure S2/S2E/cathepsin L supernatant 8bit annotated 20240216_115922-02_Ch_Chemi-01.tif]

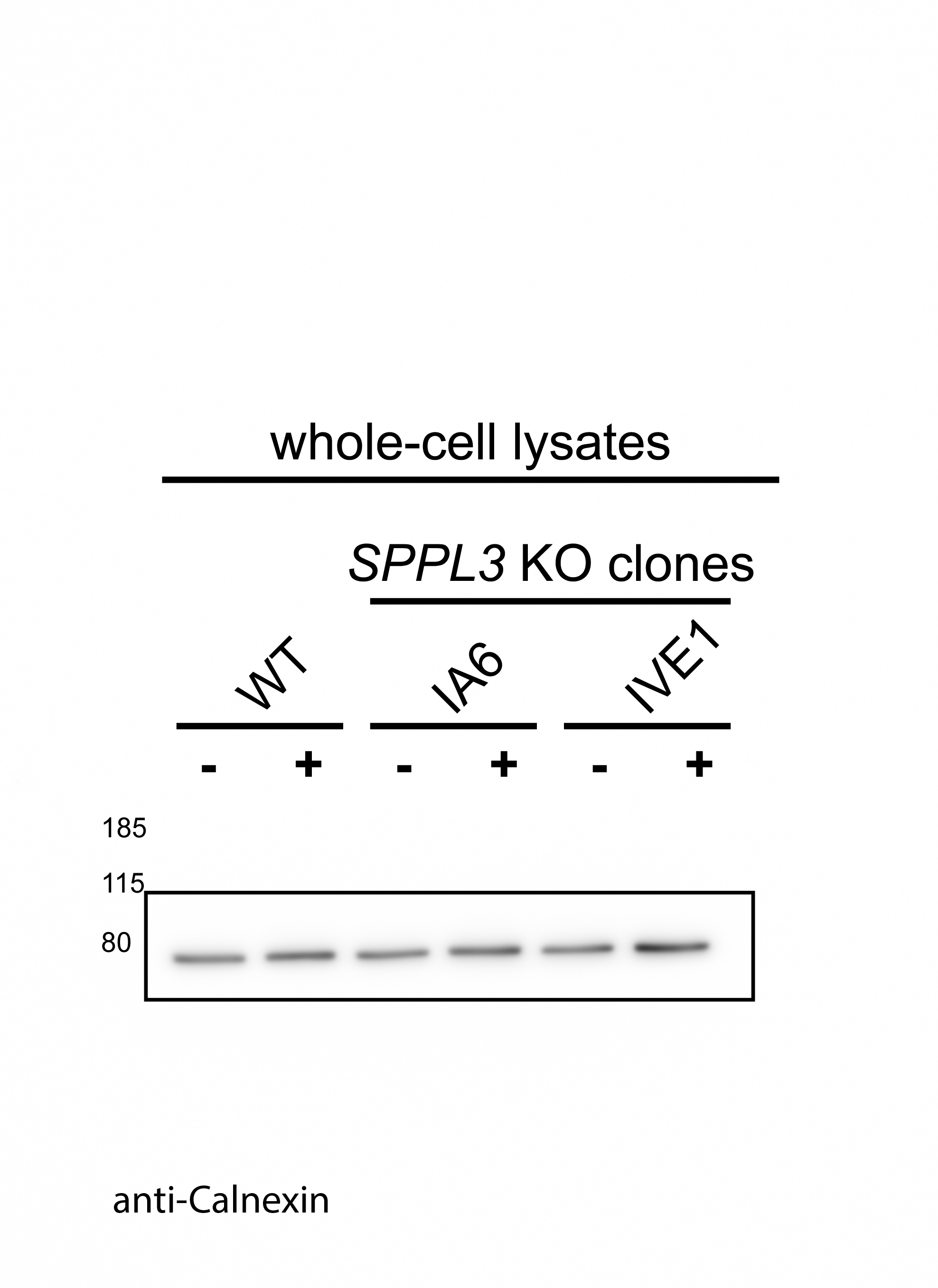

Supplement: Supplementary file 11 — Source data for Appendix [file 44318_2024_305_MOESM11_ESM.zip › Appendix/Appendix Figure S2/S2E/Calnexin lysate for B4GALT1 8bit annotated 20240319_124524-03_Ch_Chemi-01.tif]

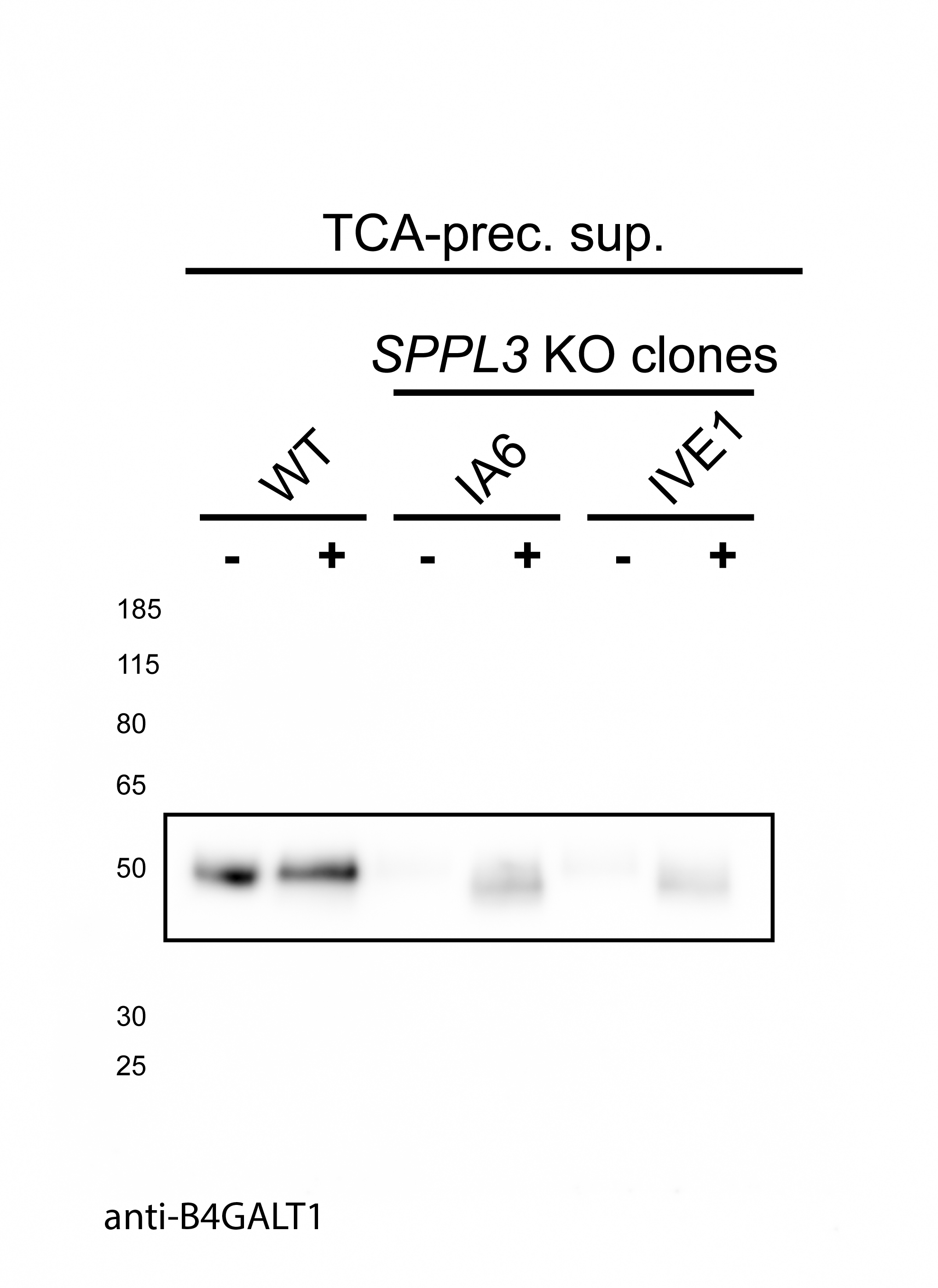

Supplement: Supplementary file 11 — Source data for Appendix [file 44318_2024_305_MOESM11_ESM.zip › Appendix/Appendix Figure S2/S2E/B4GALT1 supernatant 8bit annotated 20240220_151102-03_Ch_Chemi-01.tif]

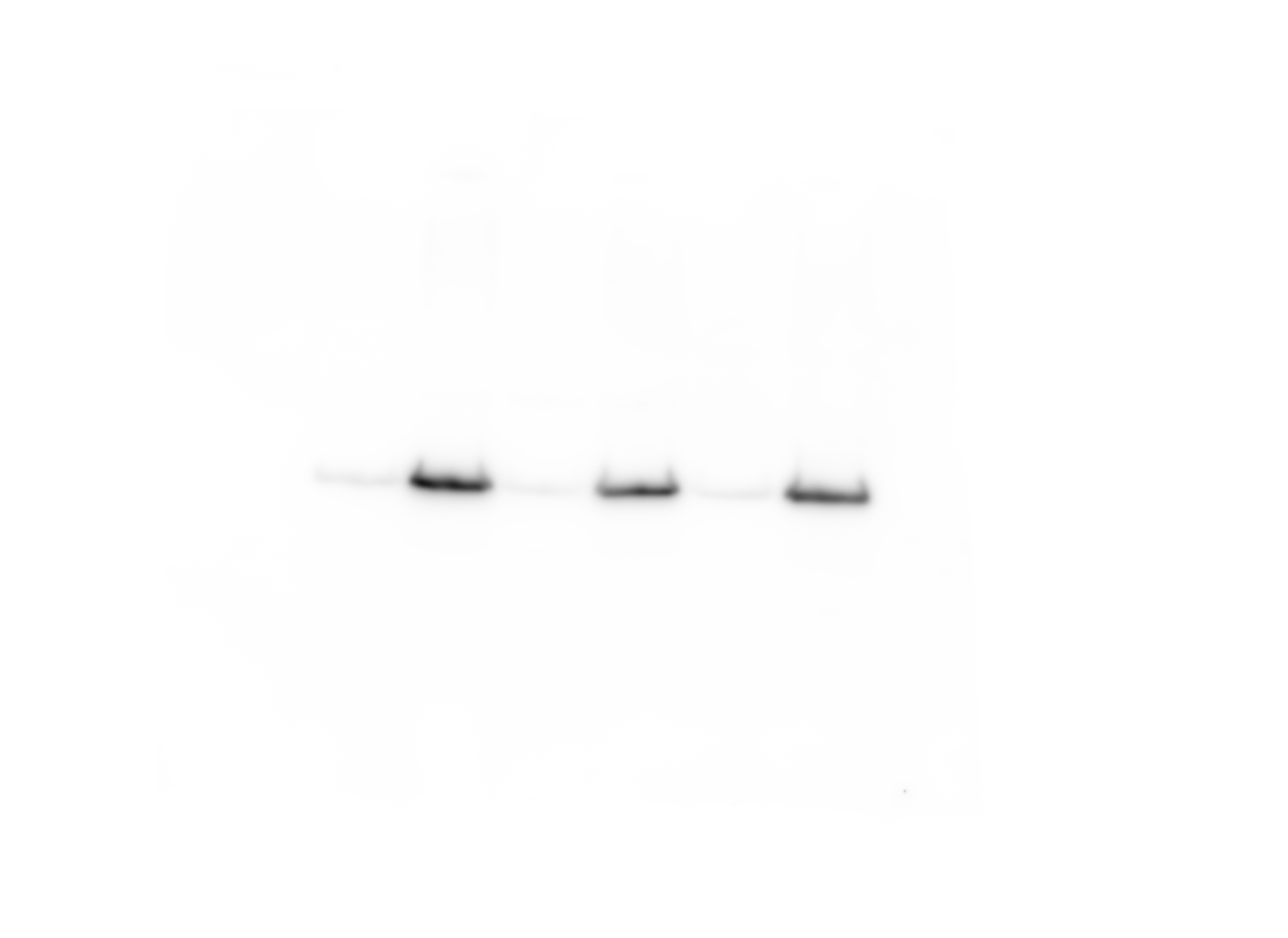

Supplement: Supplementary file 11 — Source data for Appendix [file 44318_2024_305_MOESM11_ESM.zip › Appendix/Appendix Figure S2/S2E/Cathepsin L supernatant 16bit original 20240216_115922-02_Ch_Chemi.tif]

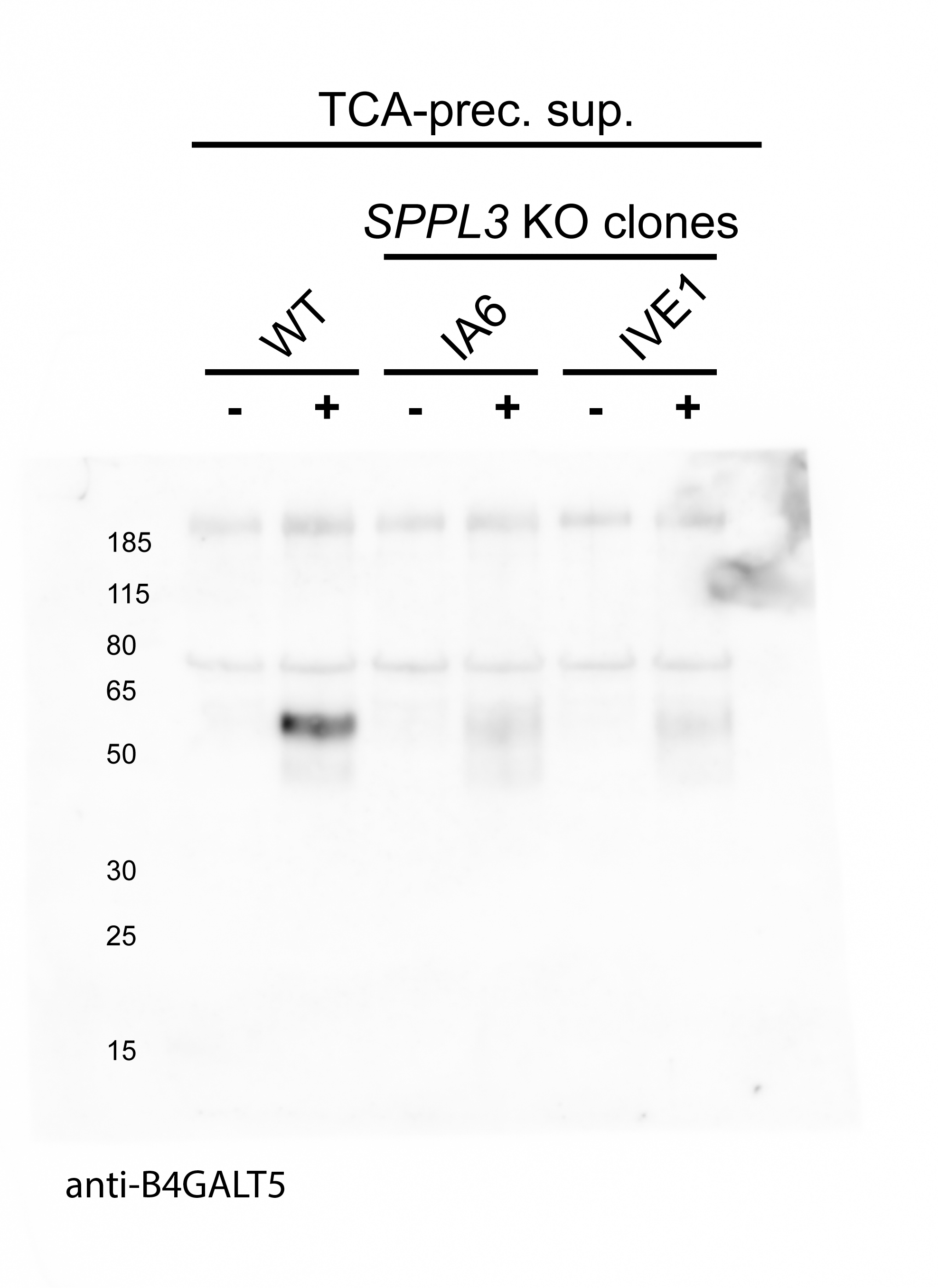

Supplement: Supplementary file 11 — Source data for Appendix [file 44318_2024_305_MOESM11_ESM.zip › Appendix/Appendix Figure S2/S2E/B4GALT5 8bit annotated 20240215_134424-05_Ch_Chemi-01.tif]

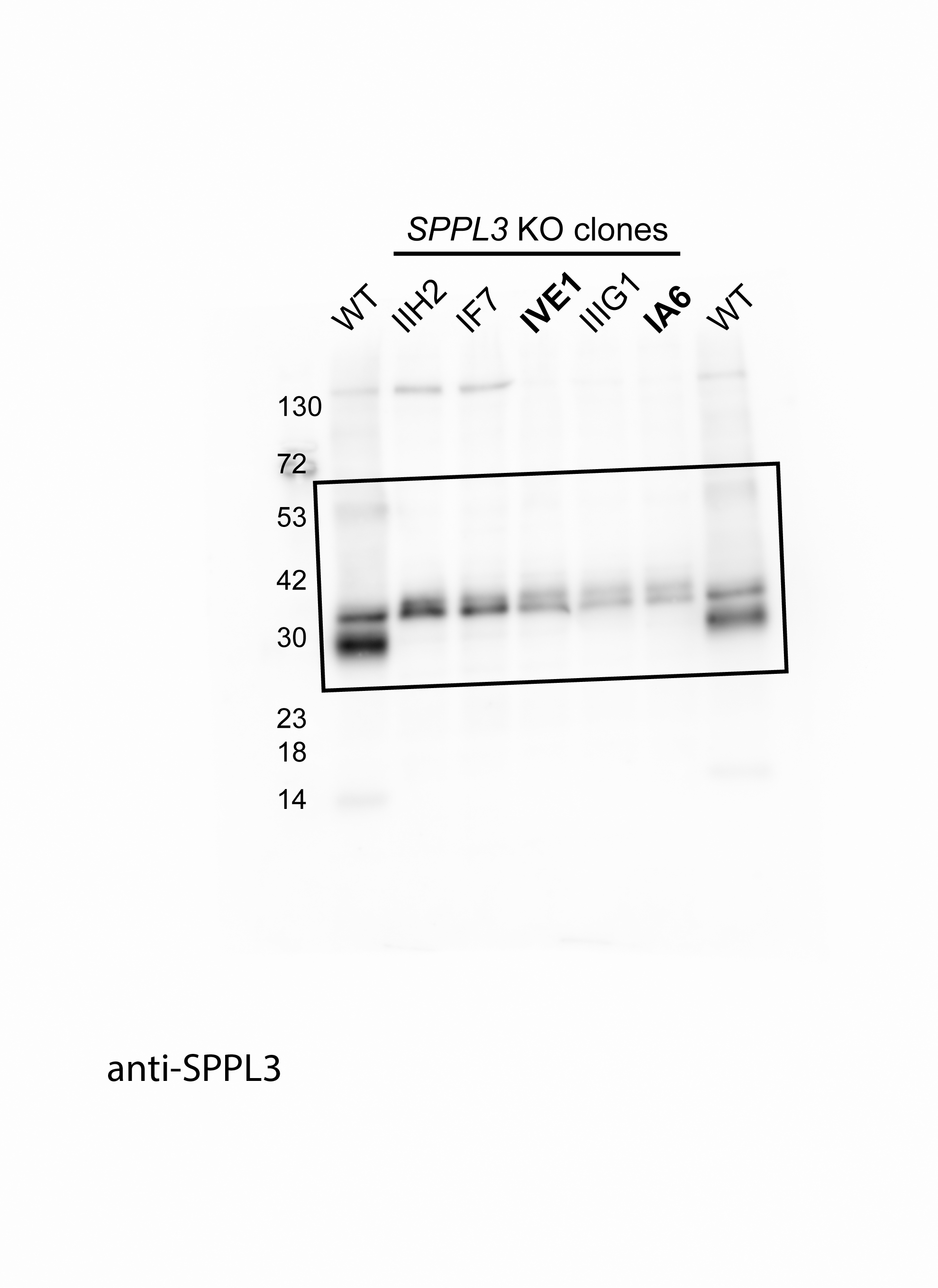

Supplement: Supplementary file 11 — Source data for Appendix [file 44318_2024_305_MOESM11_ESM.zip › Appendix/Appendix Figure S2/S2C/SPPL3 8bit annotated 20230719_124434-07_Ch_Chemi-01.tif]

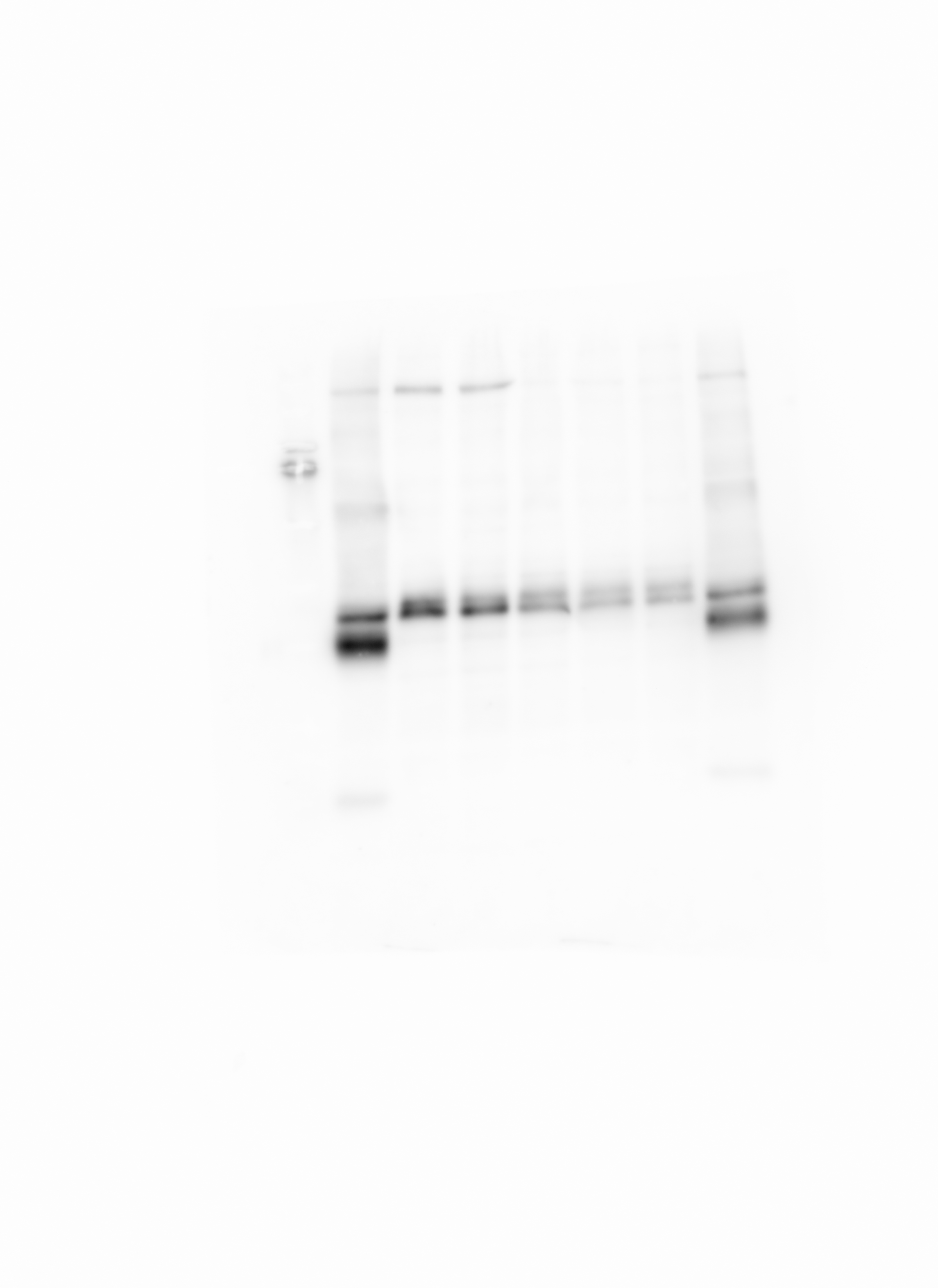

Supplement: Supplementary file 11 — Source data for Appendix [file 44318_2024_305_MOESM11_ESM.zip › Appendix/Appendix Figure S2/S2C/SPPL3 16bit original 20230719_124434-07_Ch_Chemi.tif]

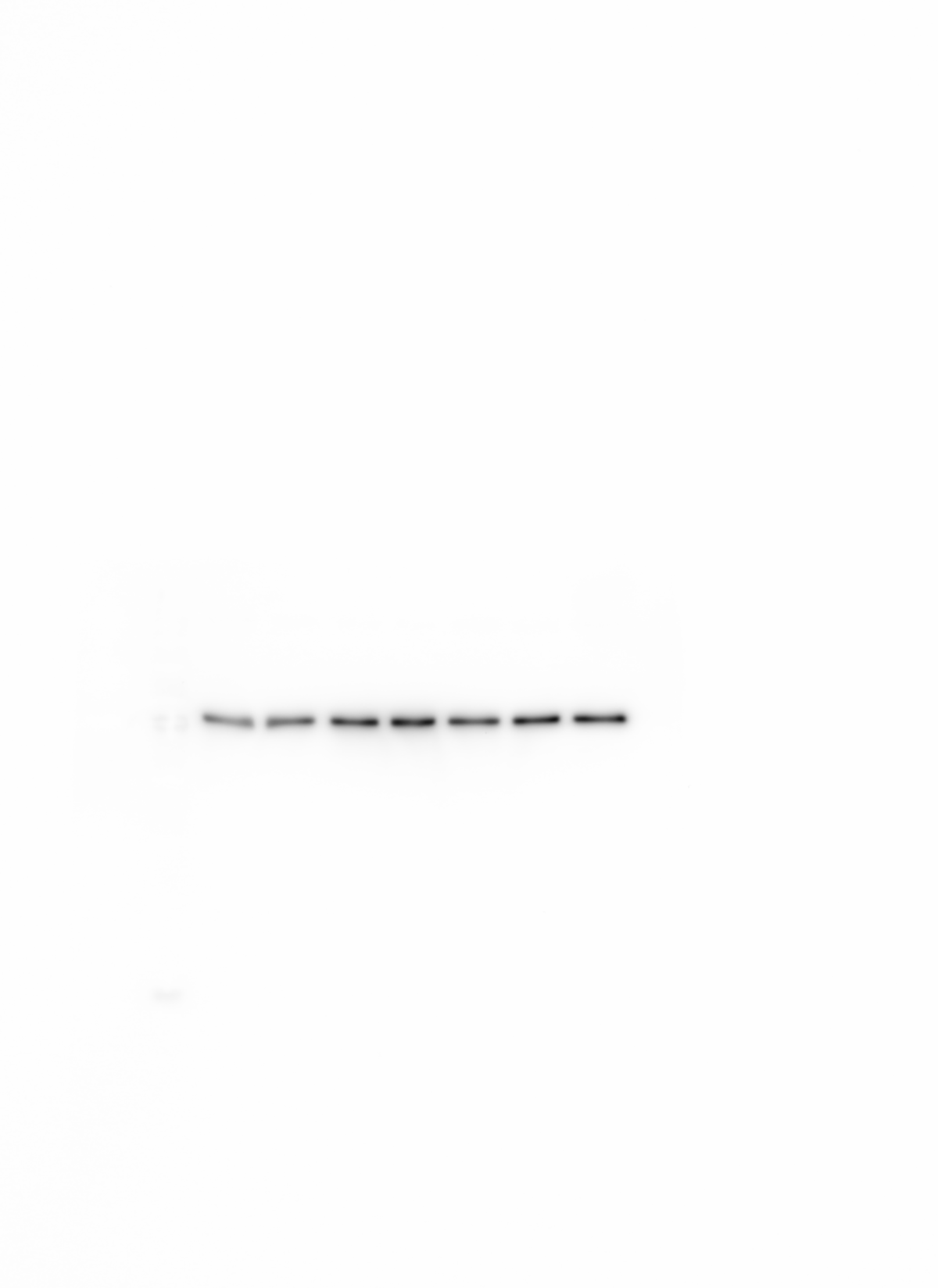

Supplement: Supplementary file 11 — Source data for Appendix [file 44318_2024_305_MOESM11_ESM.zip › Appendix/Appendix Figure S2/S2C/Calnexin 16bit original 20230721_130304-13_Ch_Chemi.tif]

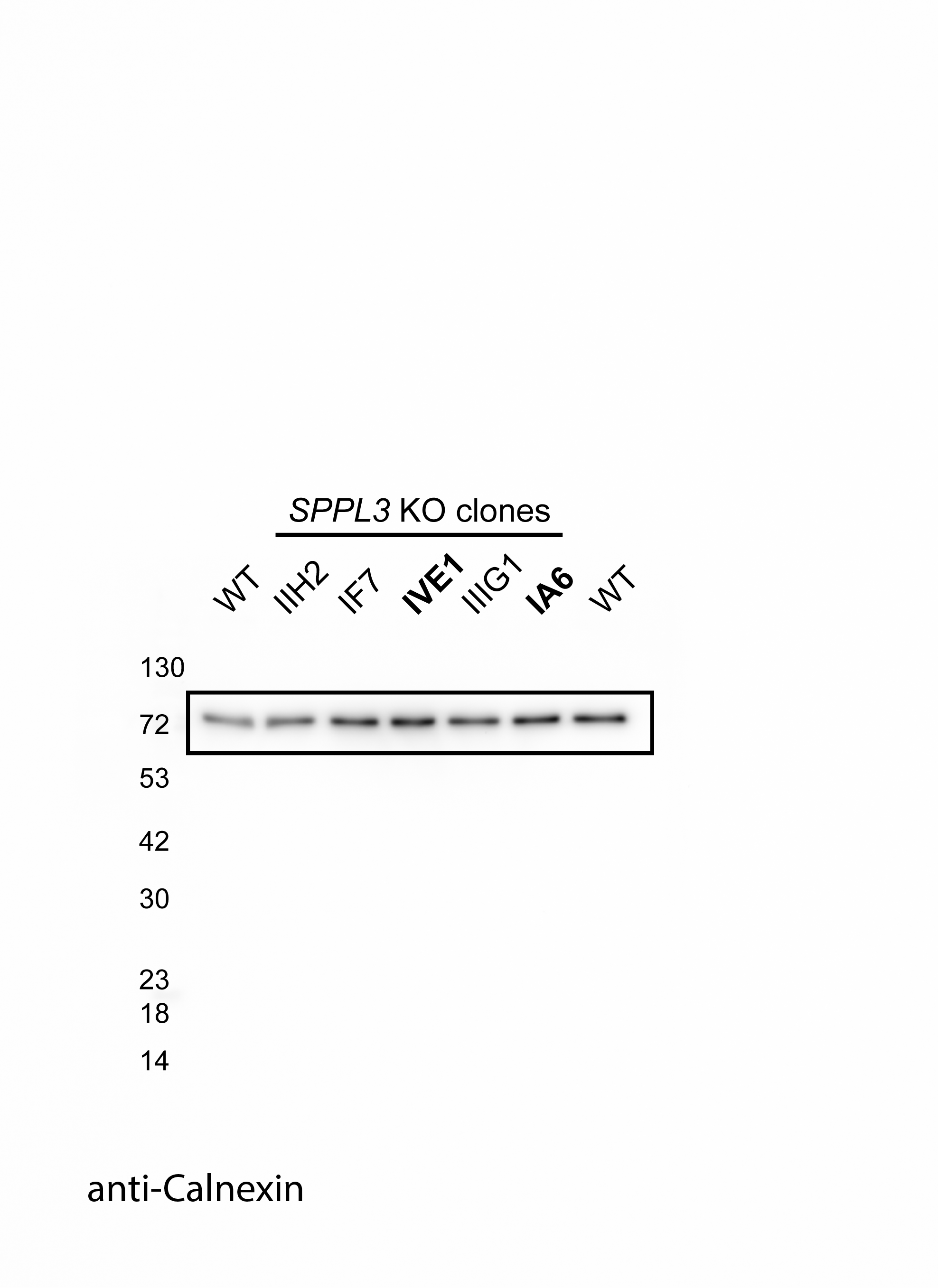

Supplement: Supplementary file 11 — Source data for Appendix [file 44318_2024_305_MOESM11_ESM.zip › Appendix/Appendix Figure S2/S2C/Calnexin 8bit annotated 20230721_130304-13_Ch_Chemi-01.tif]

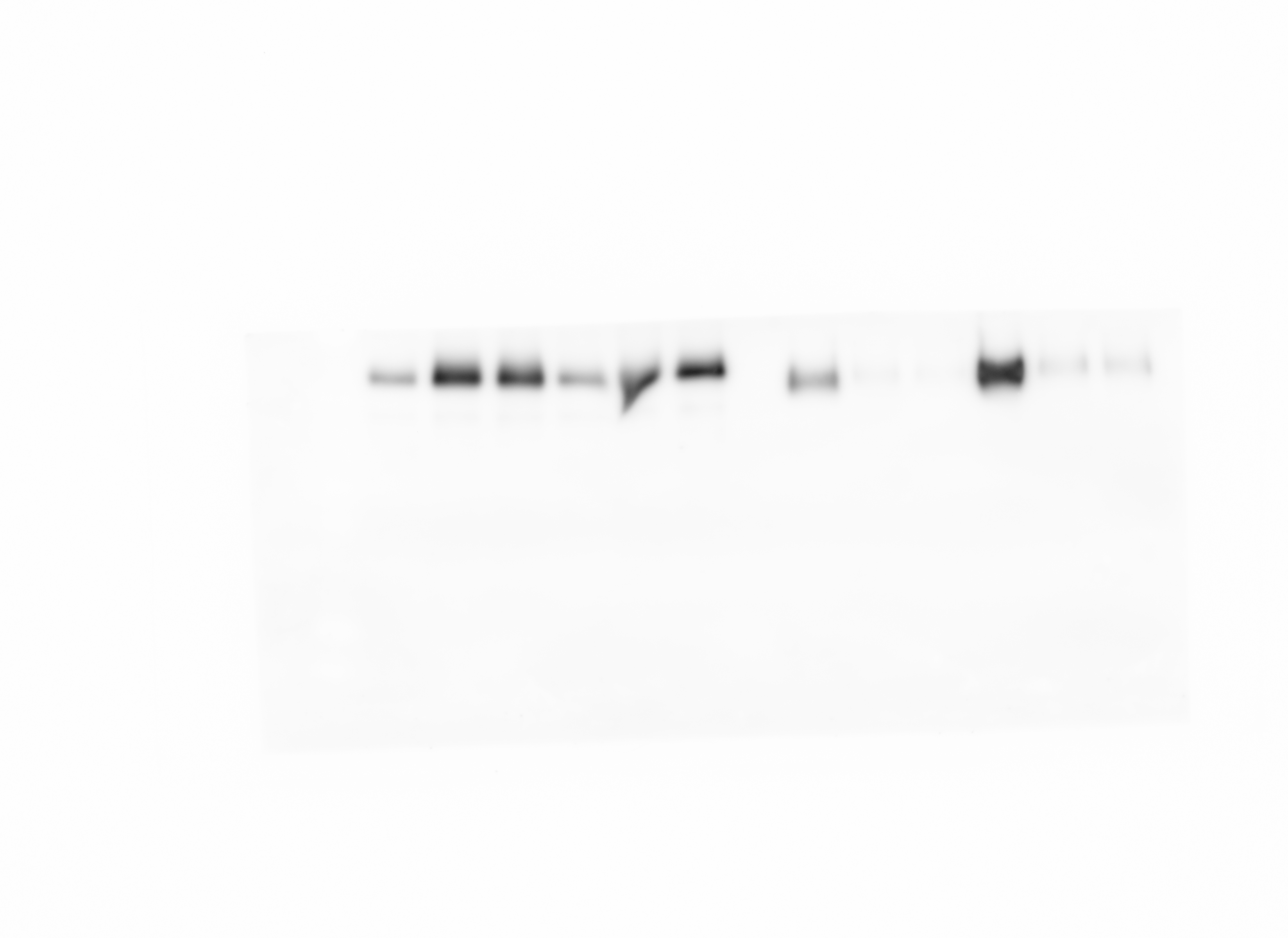

Supplement: Supplementary file 11 — Source data for Appendix [file 44318_2024_305_MOESM11_ESM.zip › Appendix/Appendix Figure S2/S2D/B4GALT1 16bit original 20230914_141347-05_Ch_Chemi.tif]

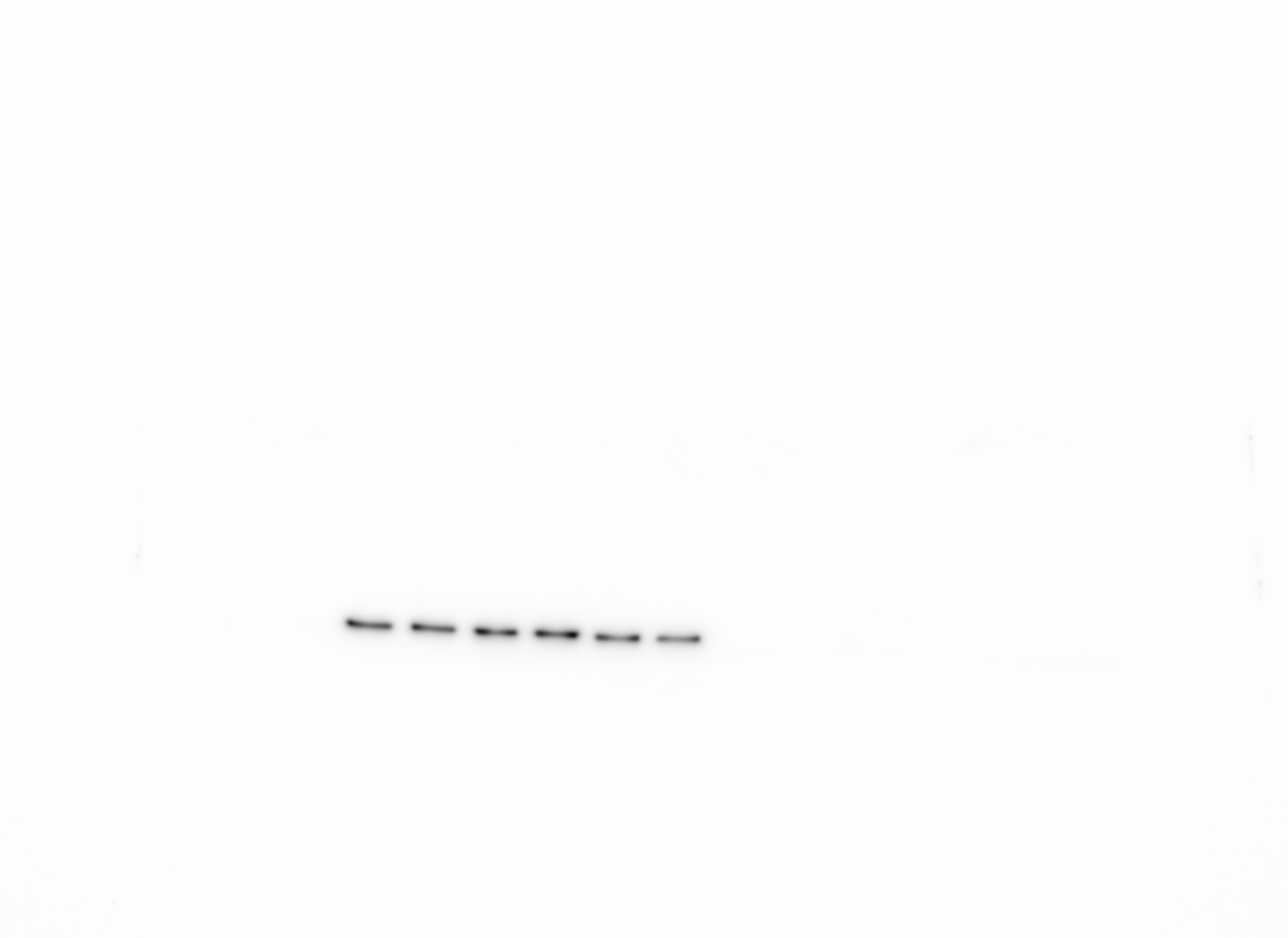

Supplement: Supplementary file 11 — Source data for Appendix [file 44318_2024_305_MOESM11_ESM.zip › Appendix/Appendix Figure S2/S2D/Calnexin 16 bit original for MGAT5 CANT1 B4GALT1 20230914_140230-27_Ch_Chemi.tif]

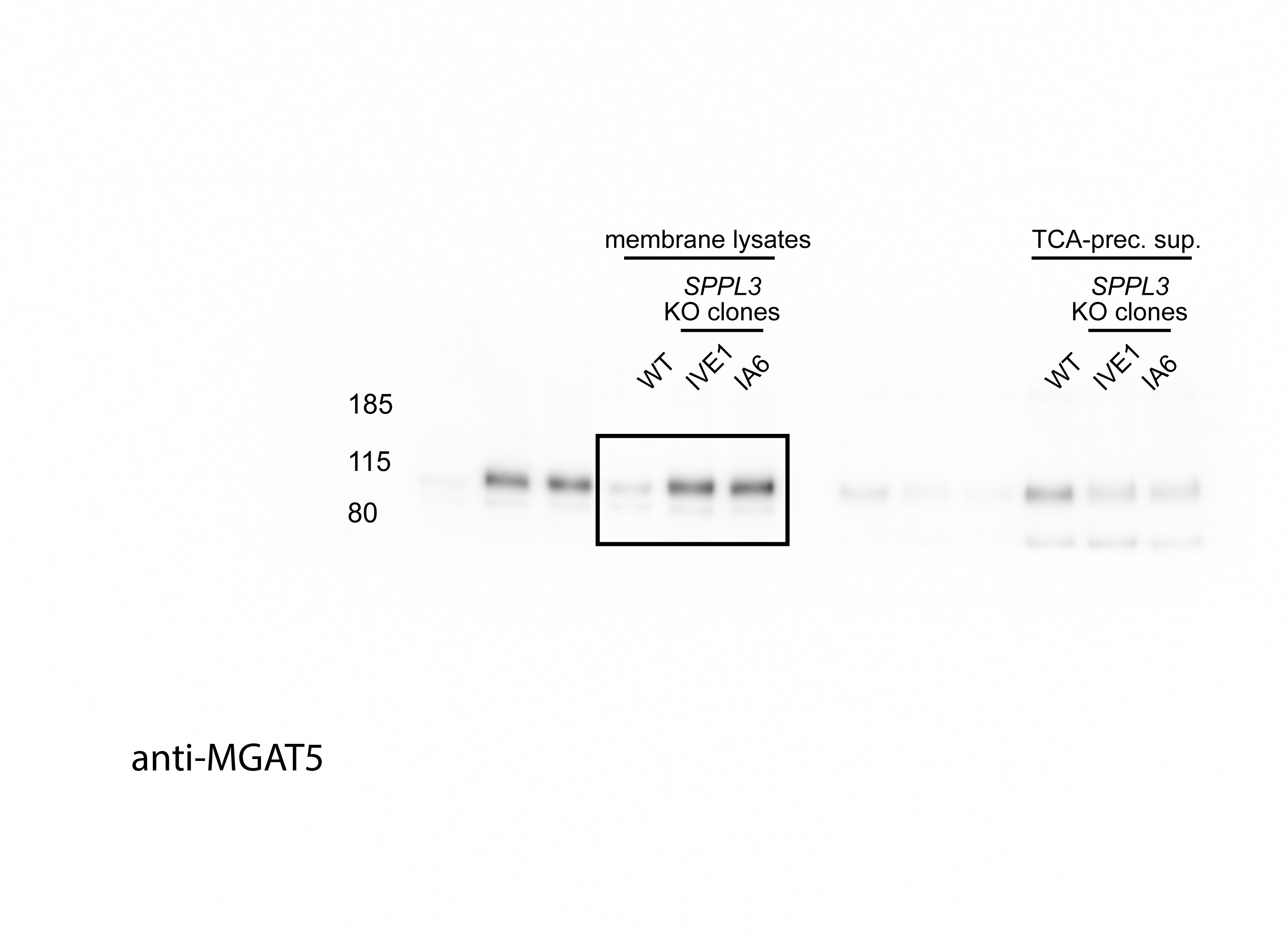

Supplement: Supplementary file 11 — Source data for Appendix [file 44318_2024_305_MOESM11_ESM.zip › Appendix/Appendix Figure S2/S2D/MGAT5 short exposure 8bit annotated 20230908_145635-10_Ch_Chemi-01.tif]

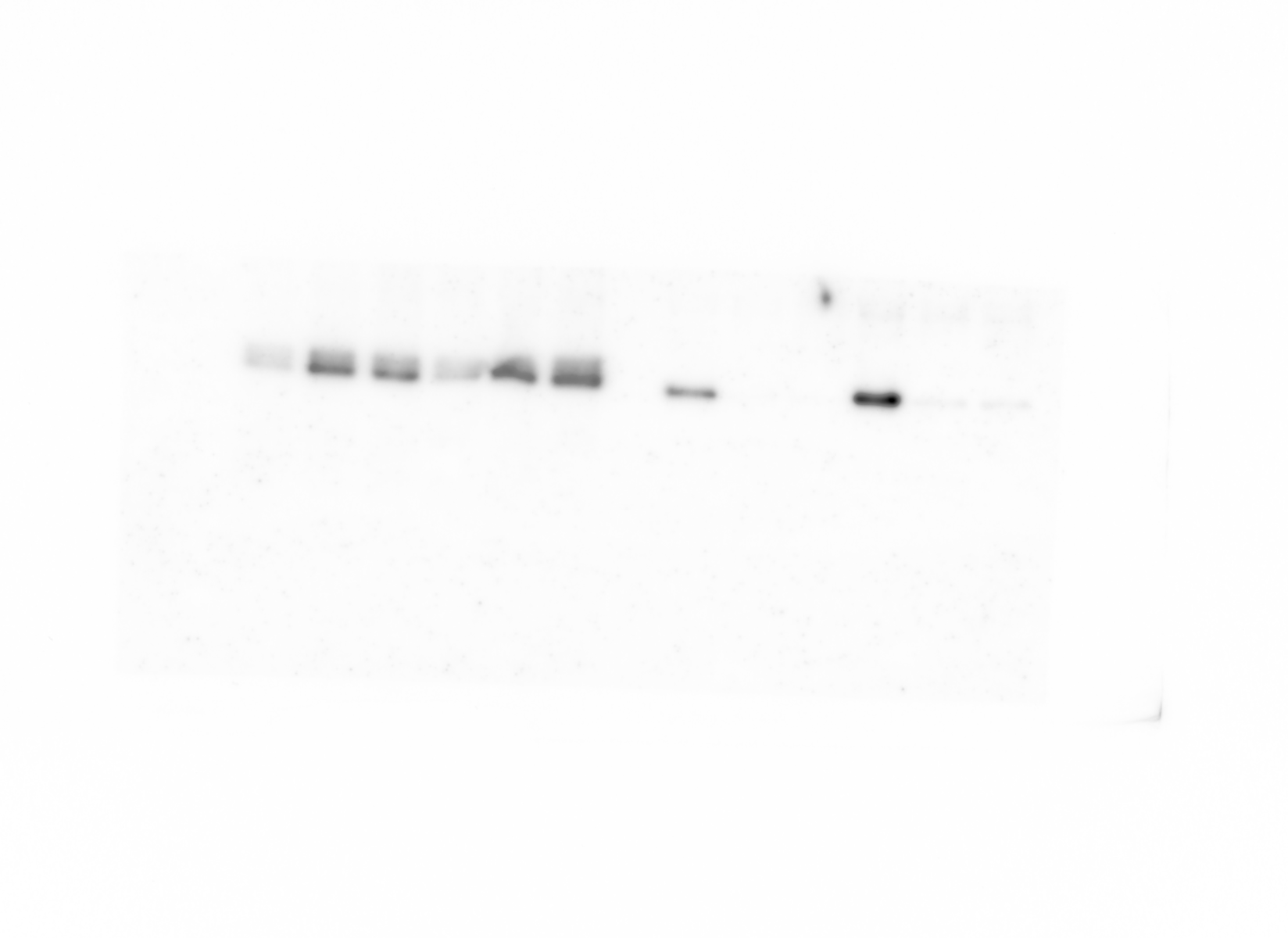

Supplement: Supplementary file 11 — Source data for Appendix [file 44318_2024_305_MOESM11_ESM.zip › Appendix/Appendix Figure S2/S2D/CANT1 16bit original 20230908_150755-16_Ch_Chemi.tif]

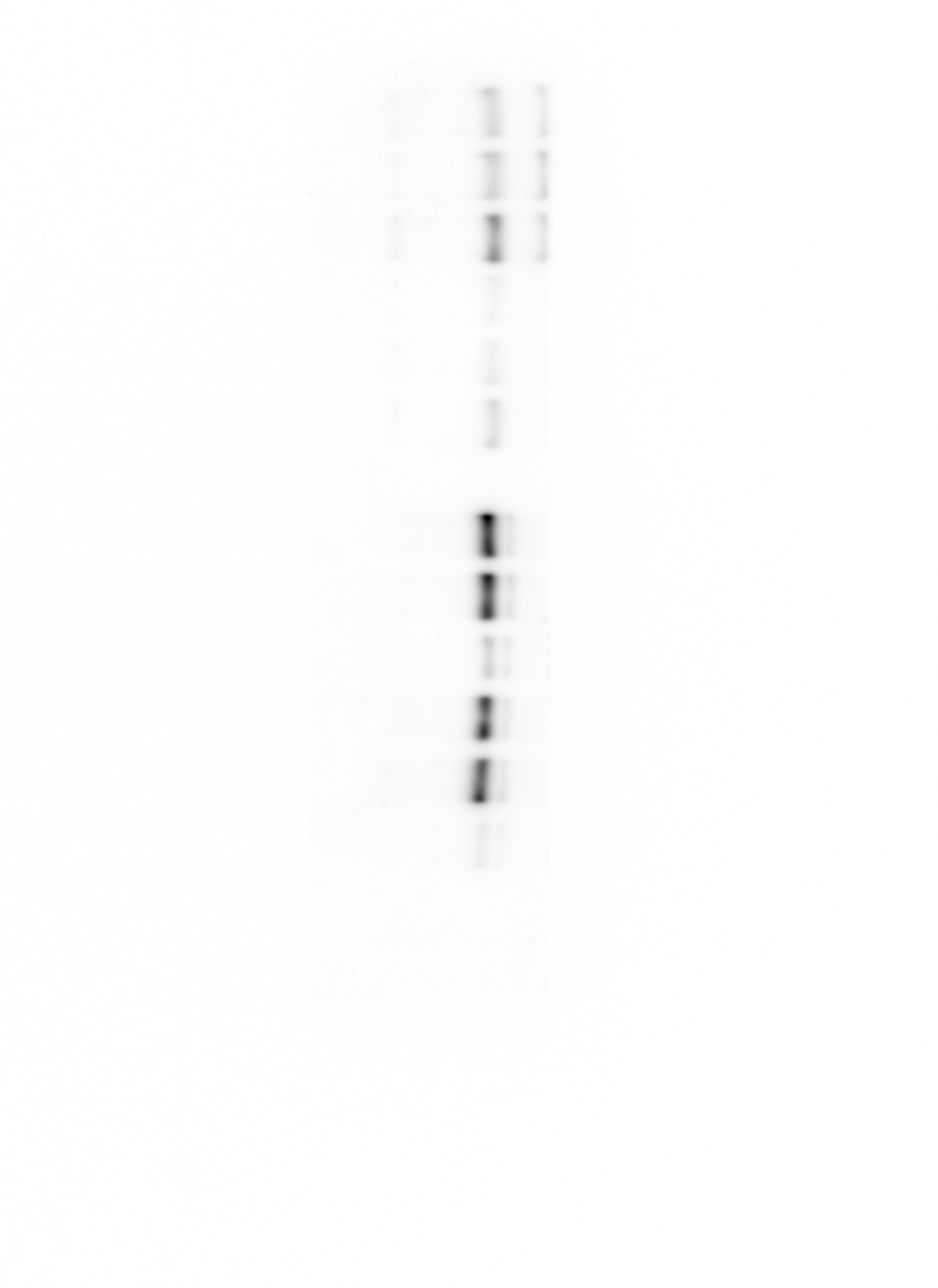

Supplement: Supplementary file 11 — Source data for Appendix [file 44318_2024_305_MOESM11_ESM.zip › Appendix/Appendix Figure S2/S2D/MGAT5 short exposure 16bit original 20230908_145635-10_Ch_Chemi.tif]

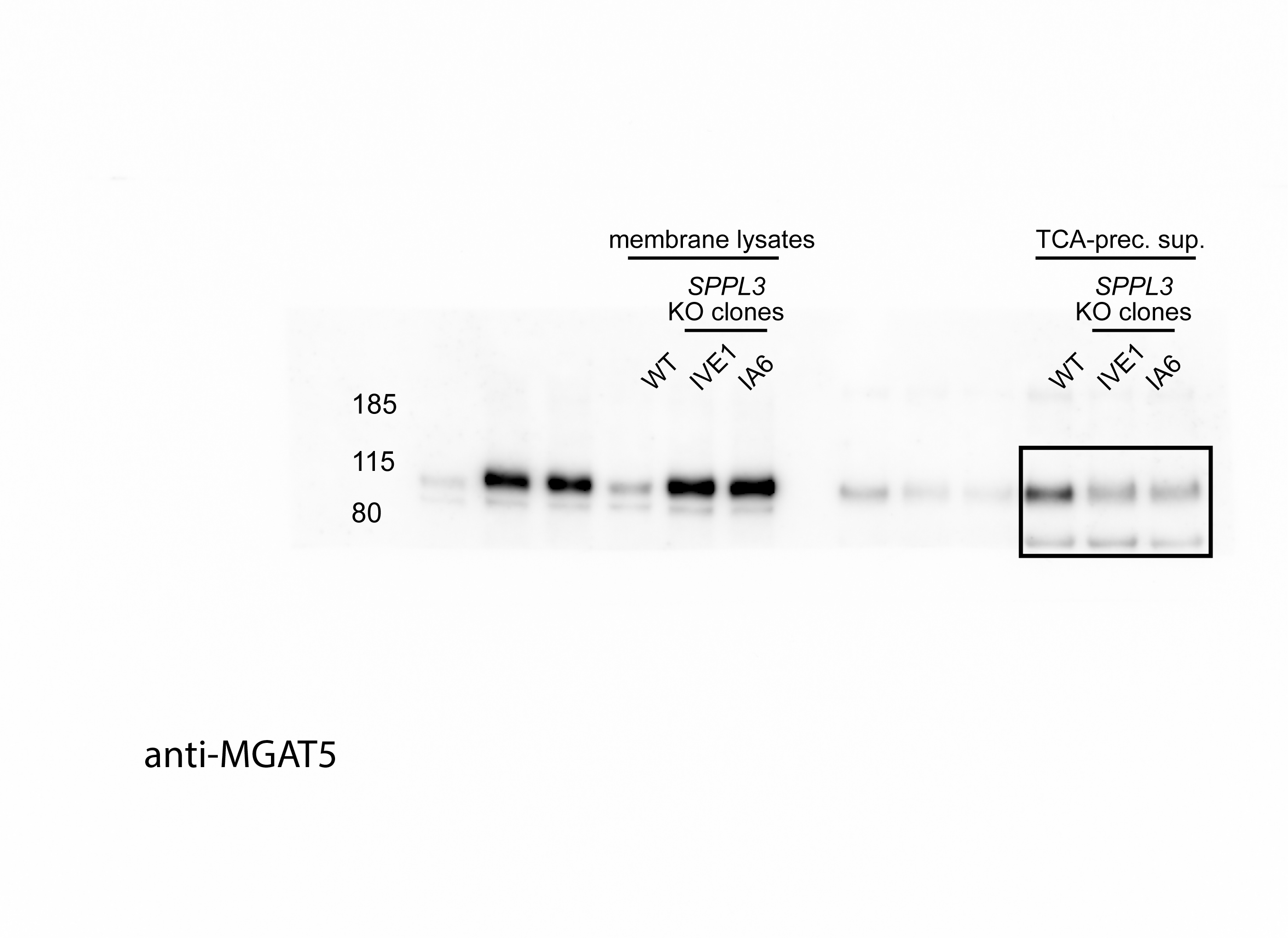

Supplement: Supplementary file 11 — Source data for Appendix [file 44318_2024_305_MOESM11_ESM.zip › Appendix/Appendix Figure S2/S2D/MGAT5 long exposure 8bit annotated 20230908_145635-29_Ch_Chemi-01.tif]

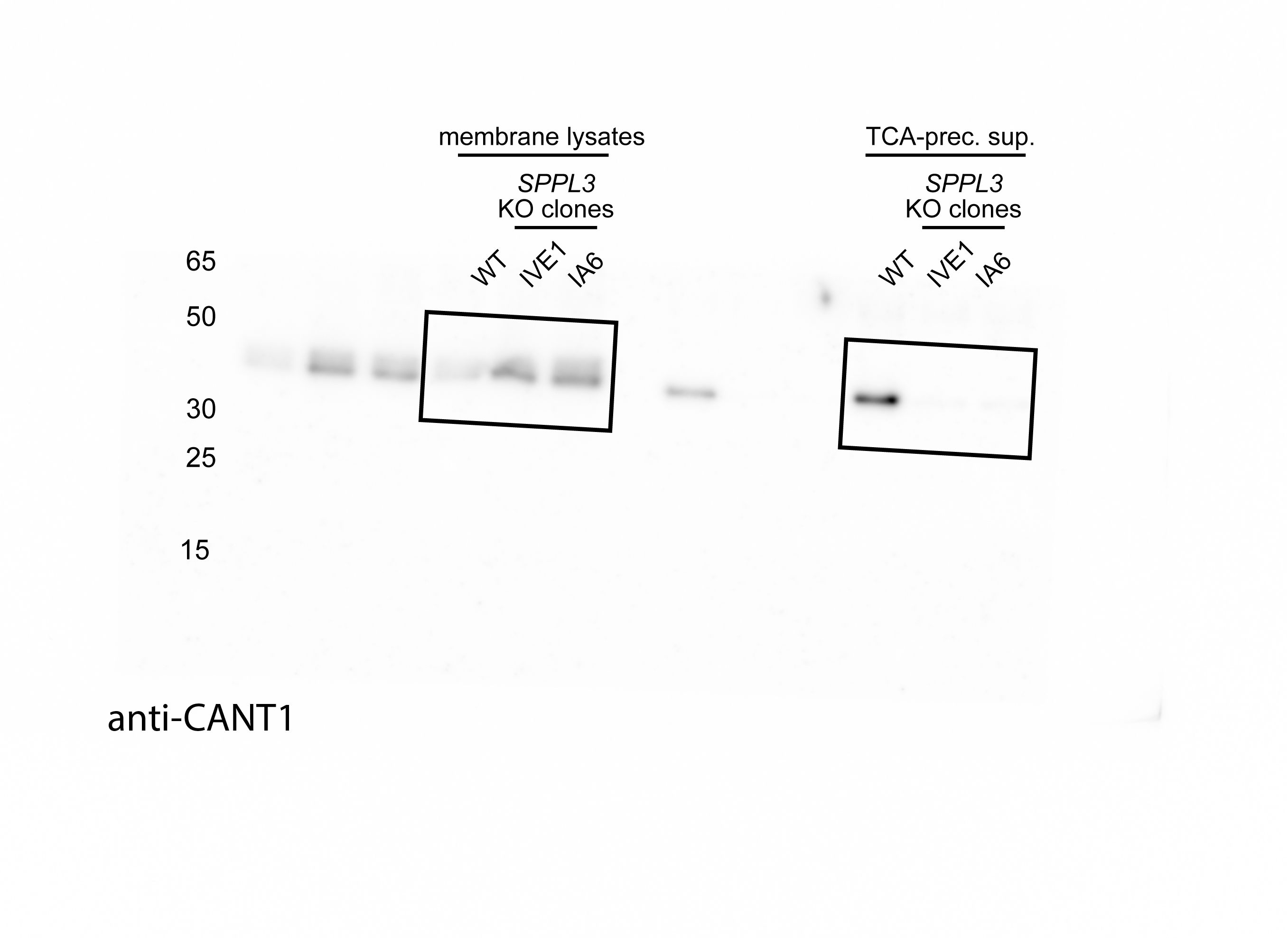

Supplement: Supplementary file 11 — Source data for Appendix [file 44318_2024_305_MOESM11_ESM.zip › Appendix/Appendix Figure S2/S2D/CANT1 8bit annotated 20230908_150755-16_Ch_Chemi-01.tif]

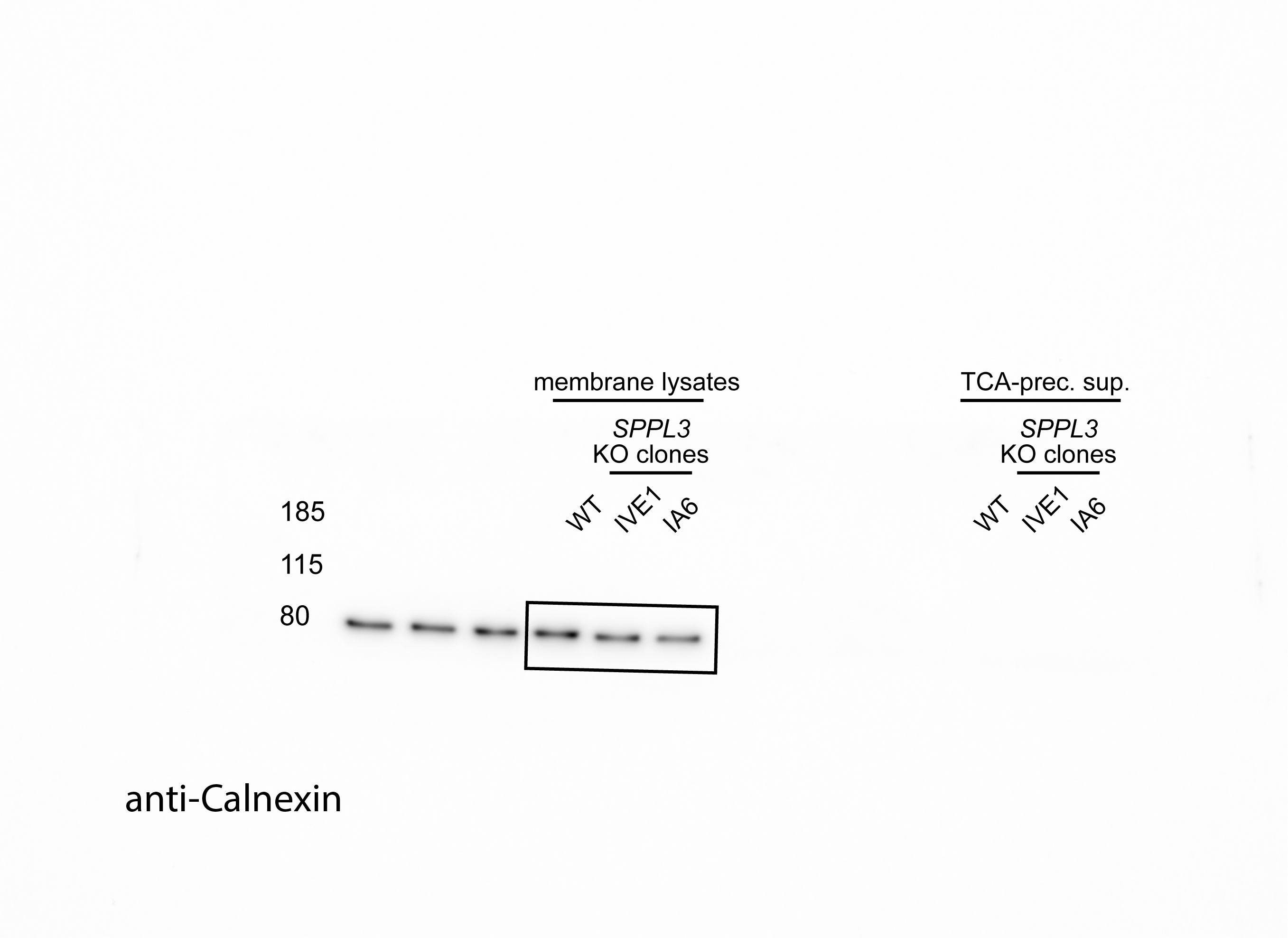

Supplement: Supplementary file 11 — Source data for Appendix [file 44318_2024_305_MOESM11_ESM.zip › Appendix/Appendix Figure S2/S2D/Calnexin 8bit annotated 20230914_140230-27_Ch_Chemi-01.tif]

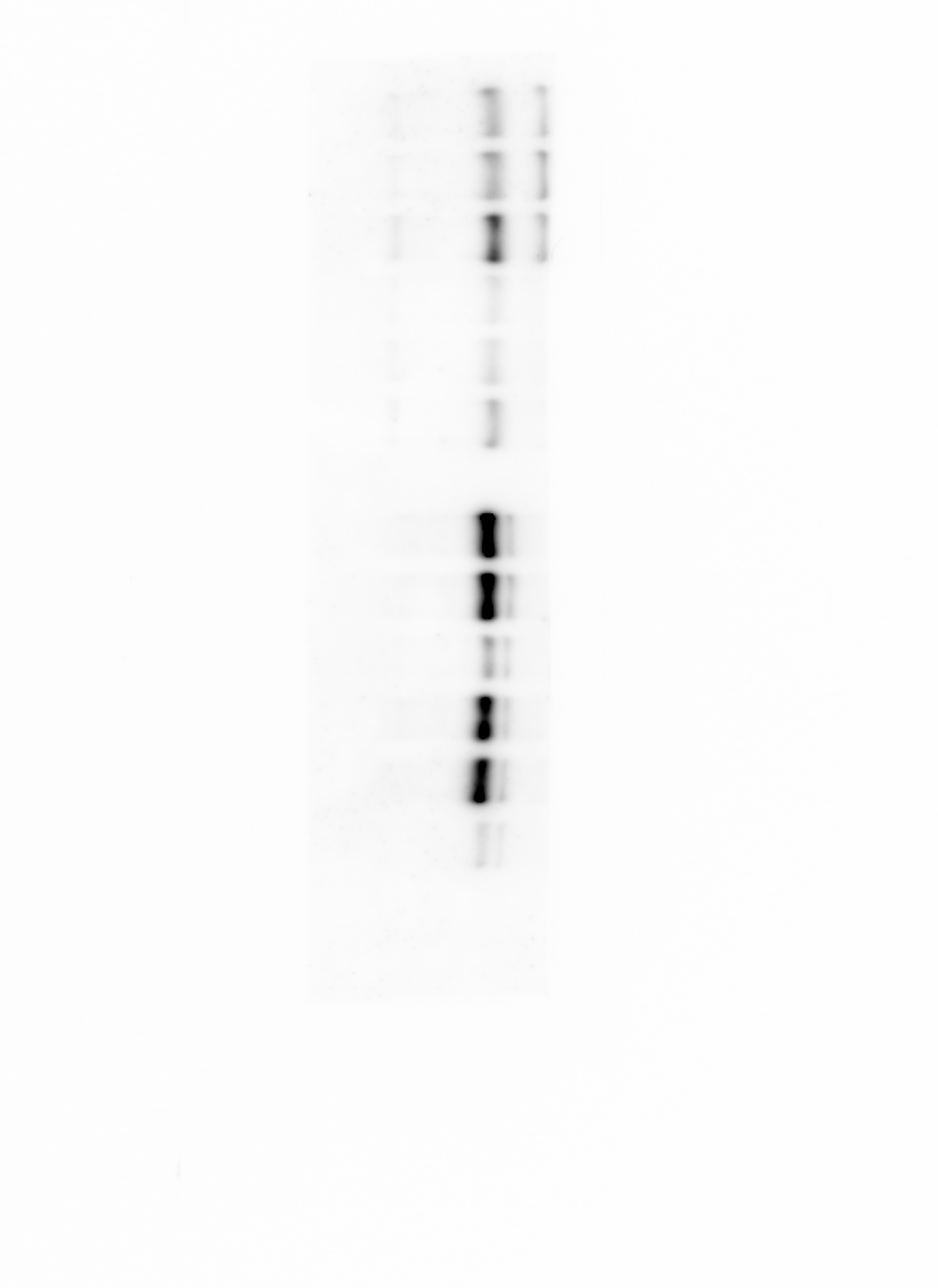

Supplement: Supplementary file 11 — Source data for Appendix [file 44318_2024_305_MOESM11_ESM.zip › Appendix/Appendix Figure S2/S2D/MGAT5 long exposure 16bit original 20230908_145635-29_Ch_Chemi.tif]

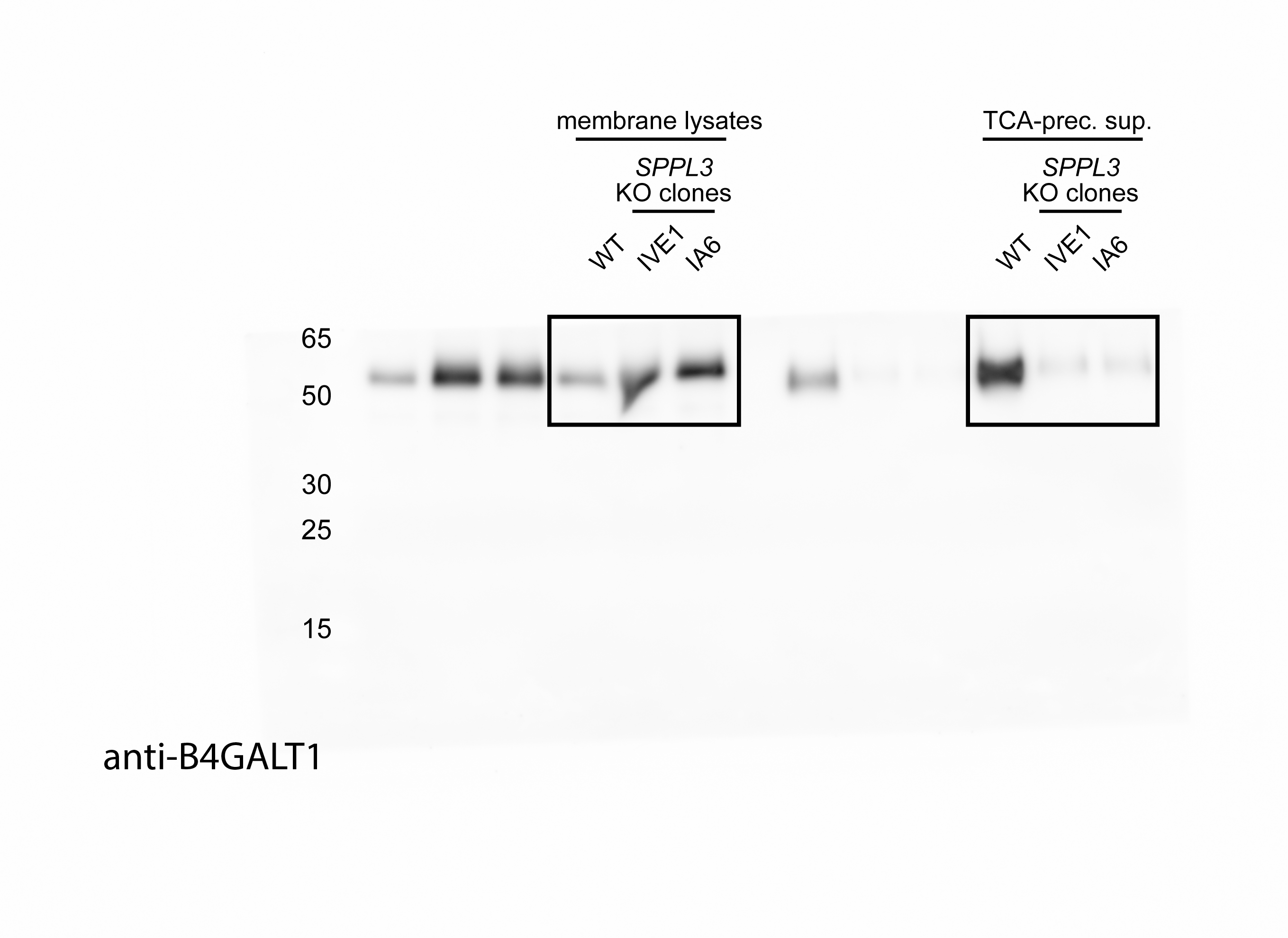

Supplement: Supplementary file 11 — Source data for Appendix [file 44318_2024_305_MOESM11_ESM.zip › Appendix/Appendix Figure S2/S2D/B4GALT1 8bit annotated 20230914_141347-05_Ch_Chemi-01.tif]

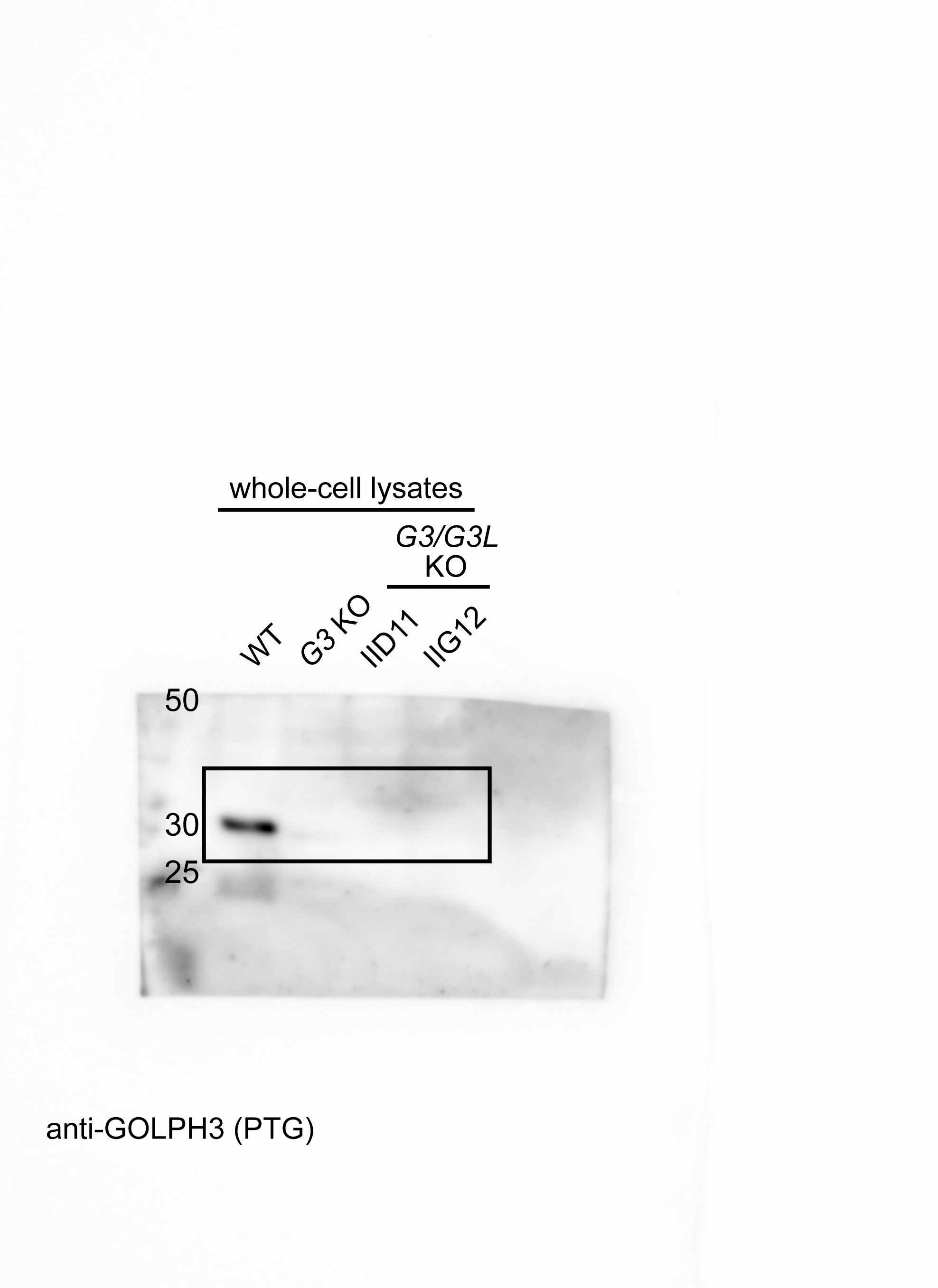

Supplement: Supplementary file 11 — Source data for Appendix [file 44318_2024_305_MOESM11_ESM.zip › Appendix/Appendix Figure S5/S5C/GOLPH3 (PTG) 8bit annotated 20240207_152419-26_Ch_Chemi-01.tif]

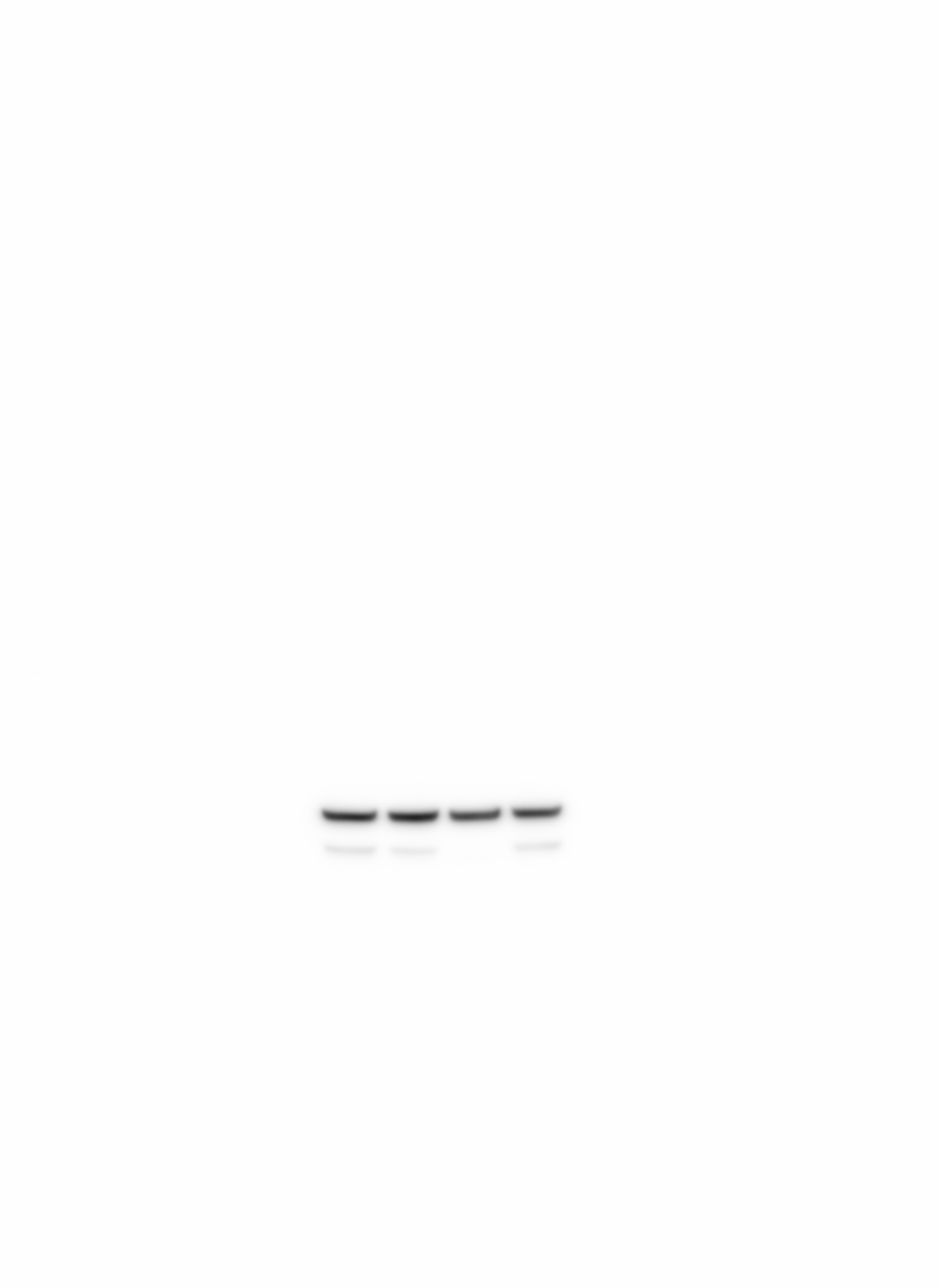

Supplement: Supplementary file 11 — Source data for Appendix [file 44318_2024_305_MOESM11_ESM.zip › Appendix/Appendix Figure S5/S5C/Calenxin for GOLPH3 16bit original 20240207_151235-06_Ch_Chemi.tif]

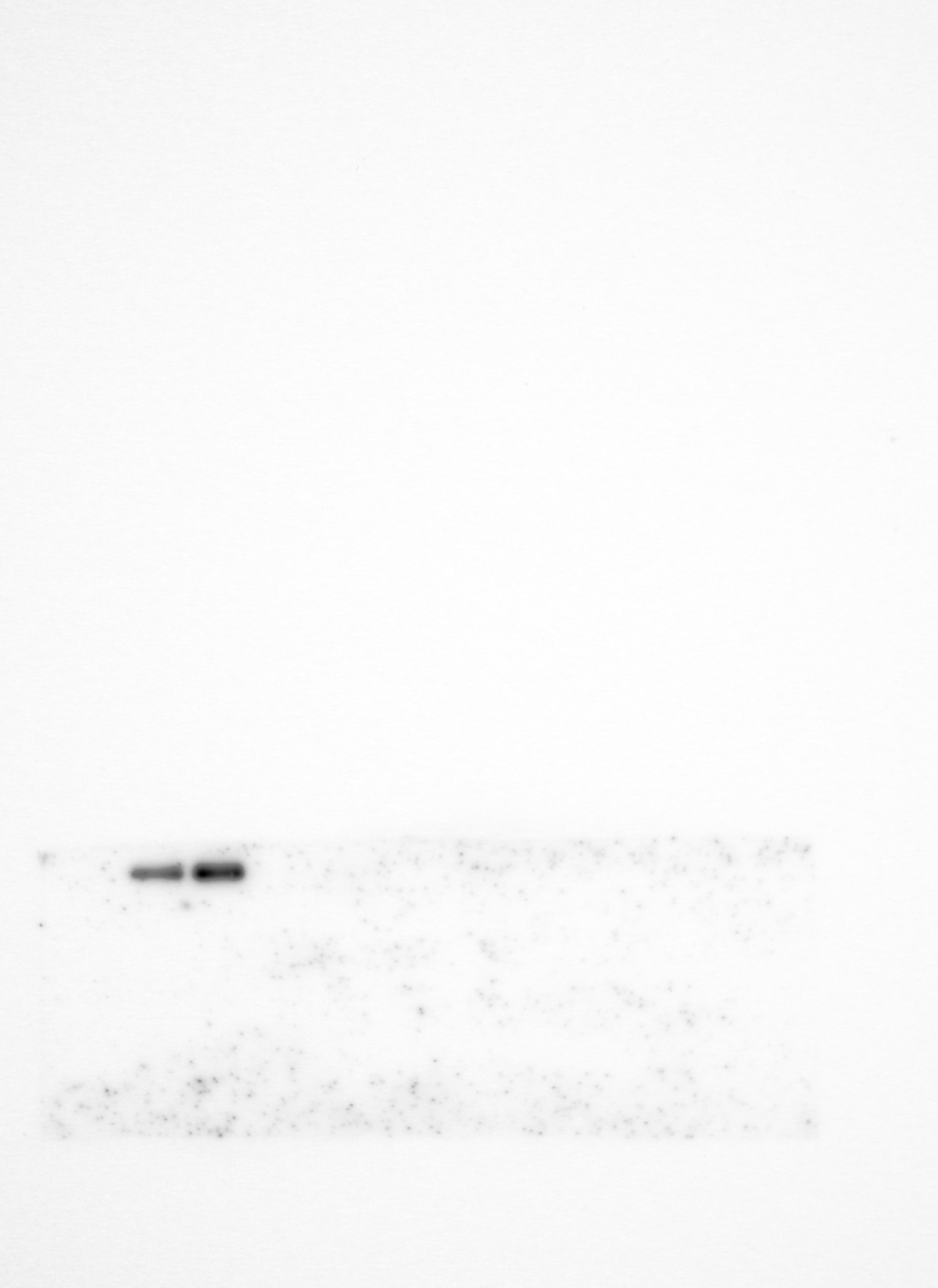

Supplement: Supplementary file 11 — Source data for Appendix [file 44318_2024_305_MOESM11_ESM.zip › Appendix/Appendix Figure S5/S5C/FAM20B 16bit original 20240214_151723-41_Ch_Chemi.tif]

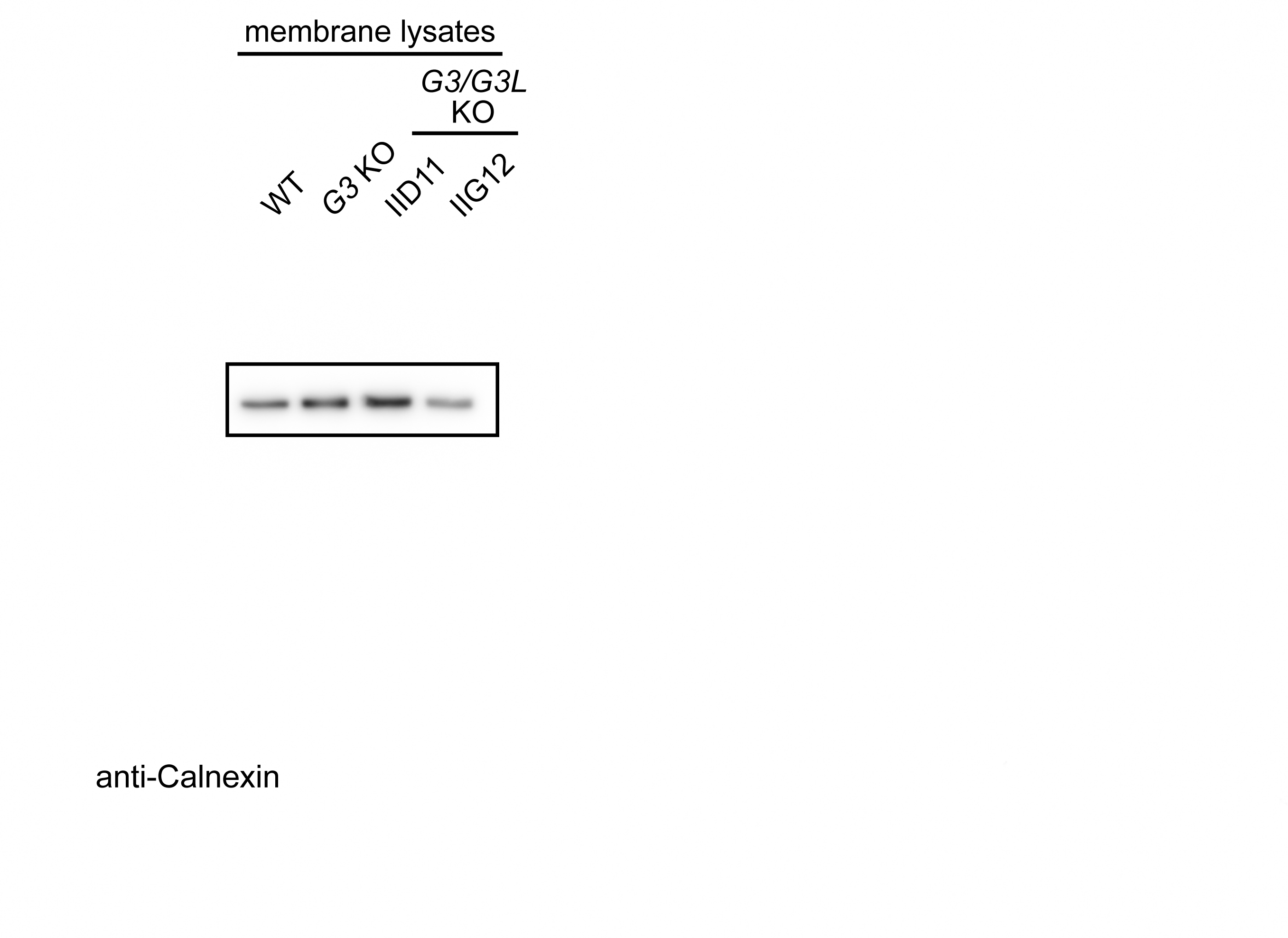

Supplement: Supplementary file 11 — Source data for Appendix [file 44318_2024_305_MOESM11_ESM.zip › Appendix/Appendix Figure S5/S5C/Calnexin for GALNT7 FAM20B 8bit annotated 20240320_144718-02_Ch_Chemi-01.tif]

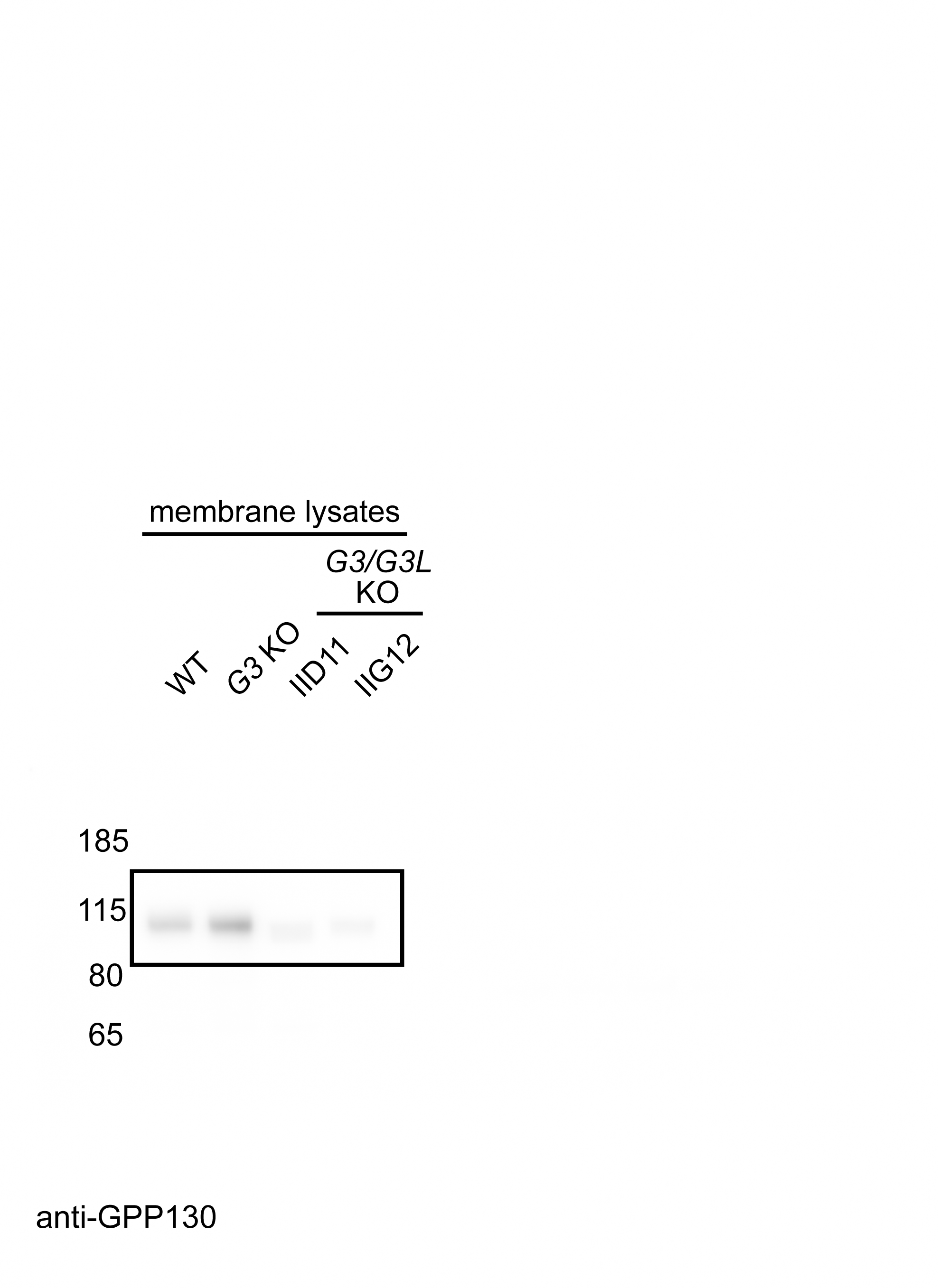

Supplement: Supplementary file 11 — Source data for Appendix [file 44318_2024_305_MOESM11_ESM.zip › Appendix/Appendix Figure S5/S5C/GPP130 8bit annotated 20240213_143725_Ch_Chemi-01.tif]

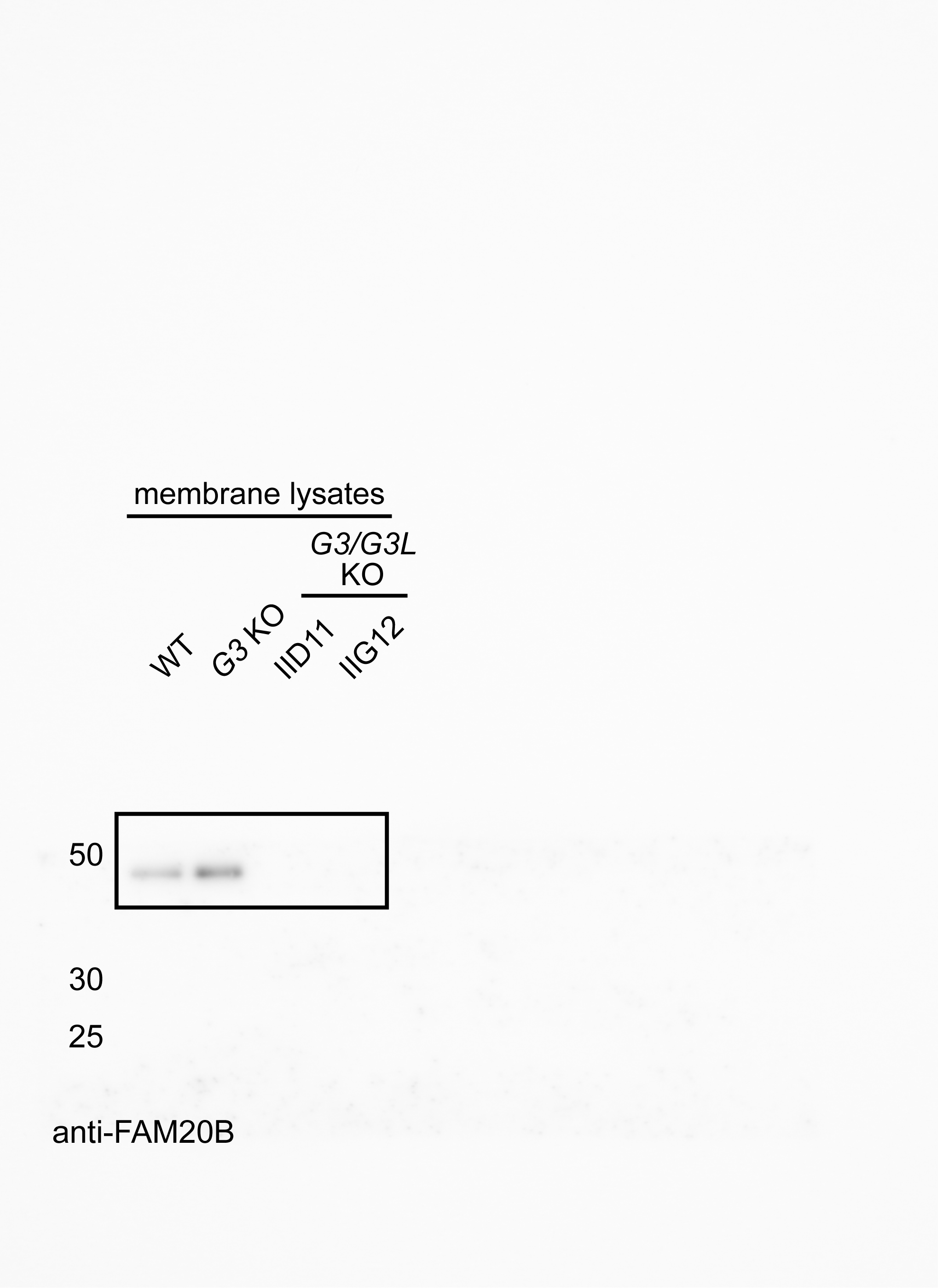

Supplement: Supplementary file 11 — Source data for Appendix [file 44318_2024_305_MOESM11_ESM.zip › Appendix/Appendix Figure S5/S5C/FAM20B 8bit annotated 20240214_151723-41_Ch_Chemi-01.tif]

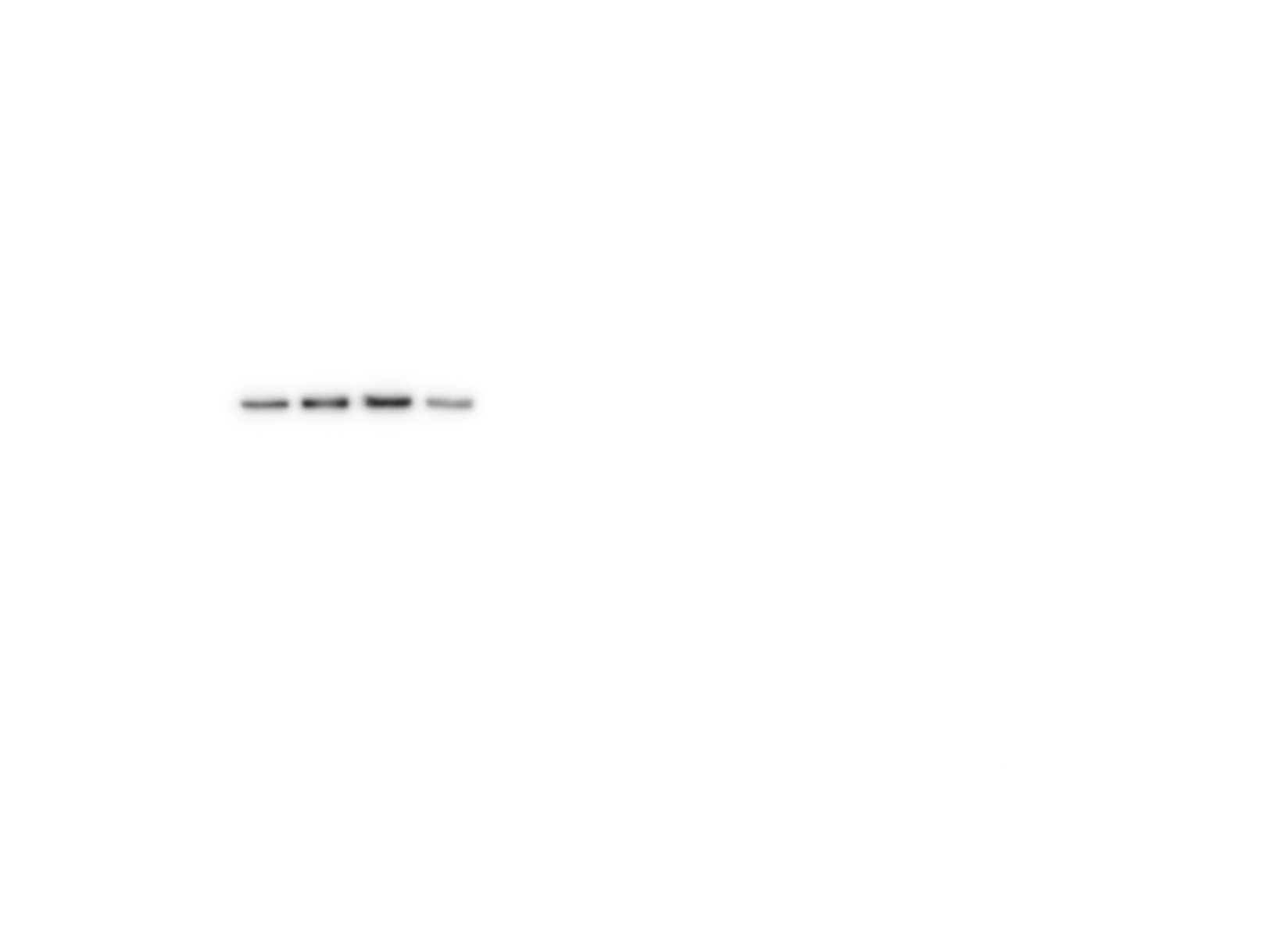

Supplement: Supplementary file 11 — Source data for Appendix [file 44318_2024_305_MOESM11_ESM.zip › Appendix/Appendix Figure S5/S5C/Calnexin for GALNT7 FAM20B 16bit original 20240320_144718-02_Ch_Chemi.tif]

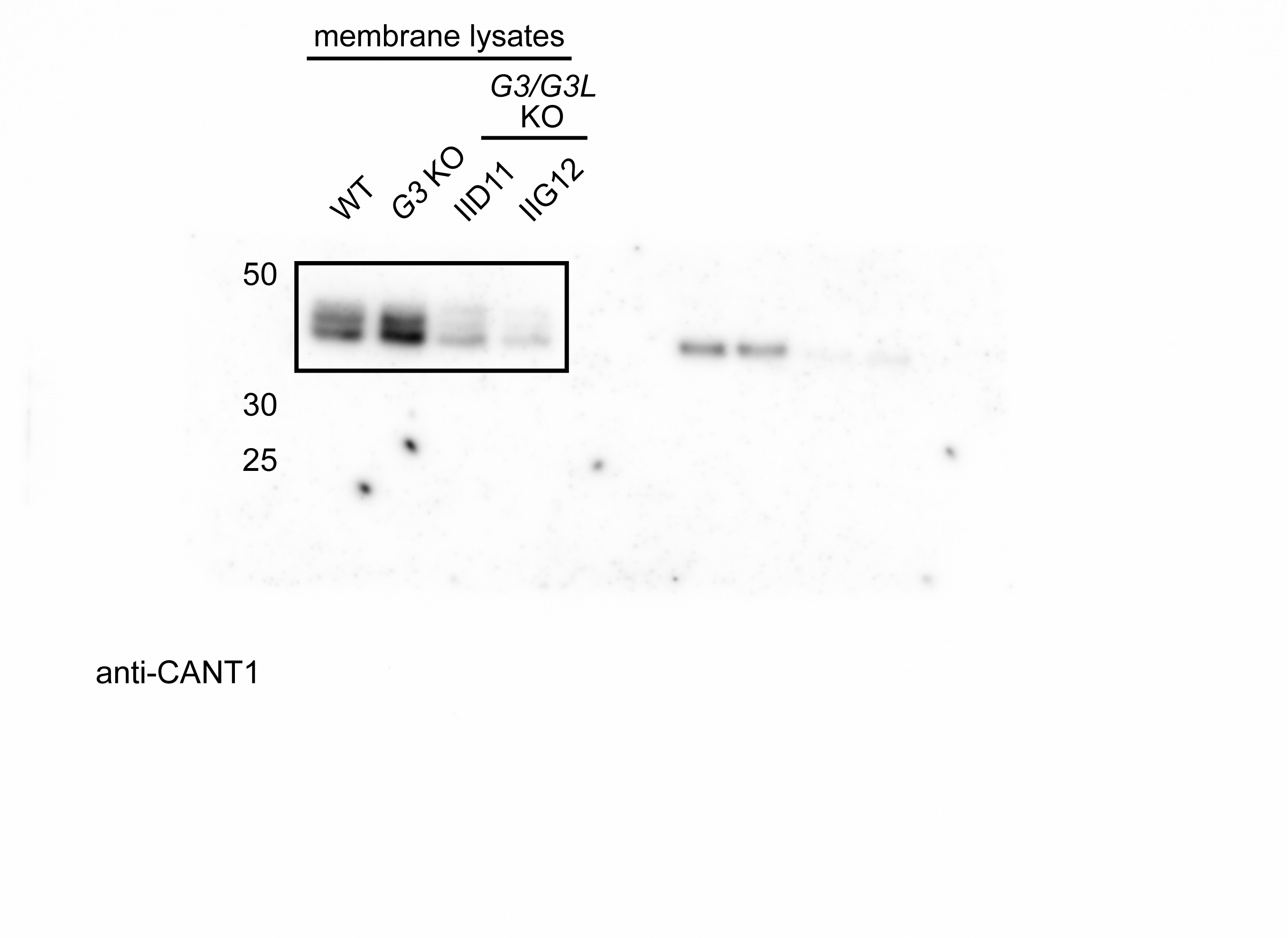

Supplement: Supplementary file 11 — Source data for Appendix [file 44318_2024_305_MOESM11_ESM.zip › Appendix/Appendix Figure S5/S5C/CANT1 8bit annotated 20240208_140211-10_Ch_Chemi-01.tif]

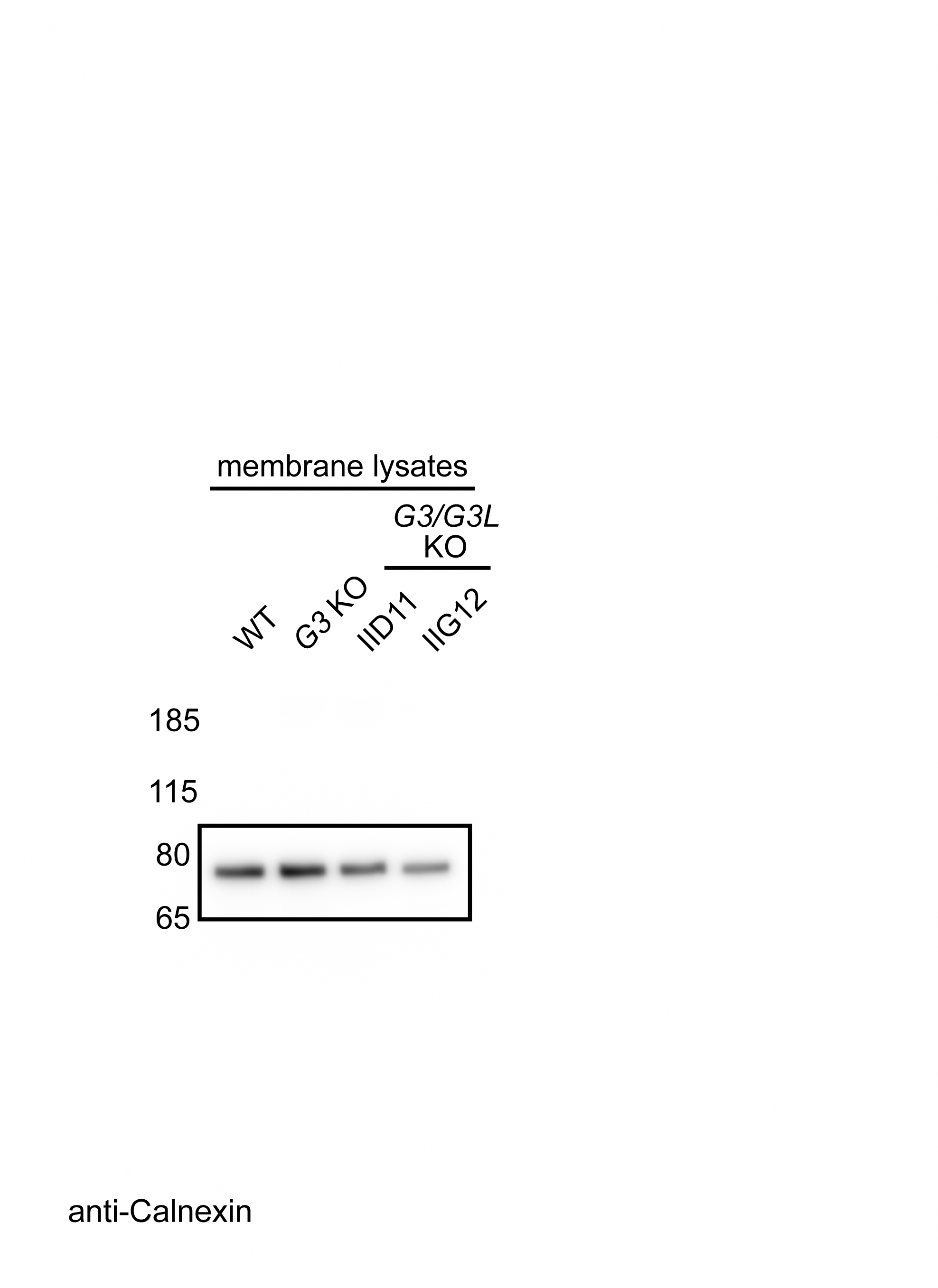

Supplement: Supplementary file 11 — Source data for Appendix [file 44318_2024_305_MOESM11_ESM.zip › Appendix/Appendix Figure S5/S5C/Calnexin for CANT1 8bit annotated 20240312_152948-02_Ch_Chemi-01.tif]

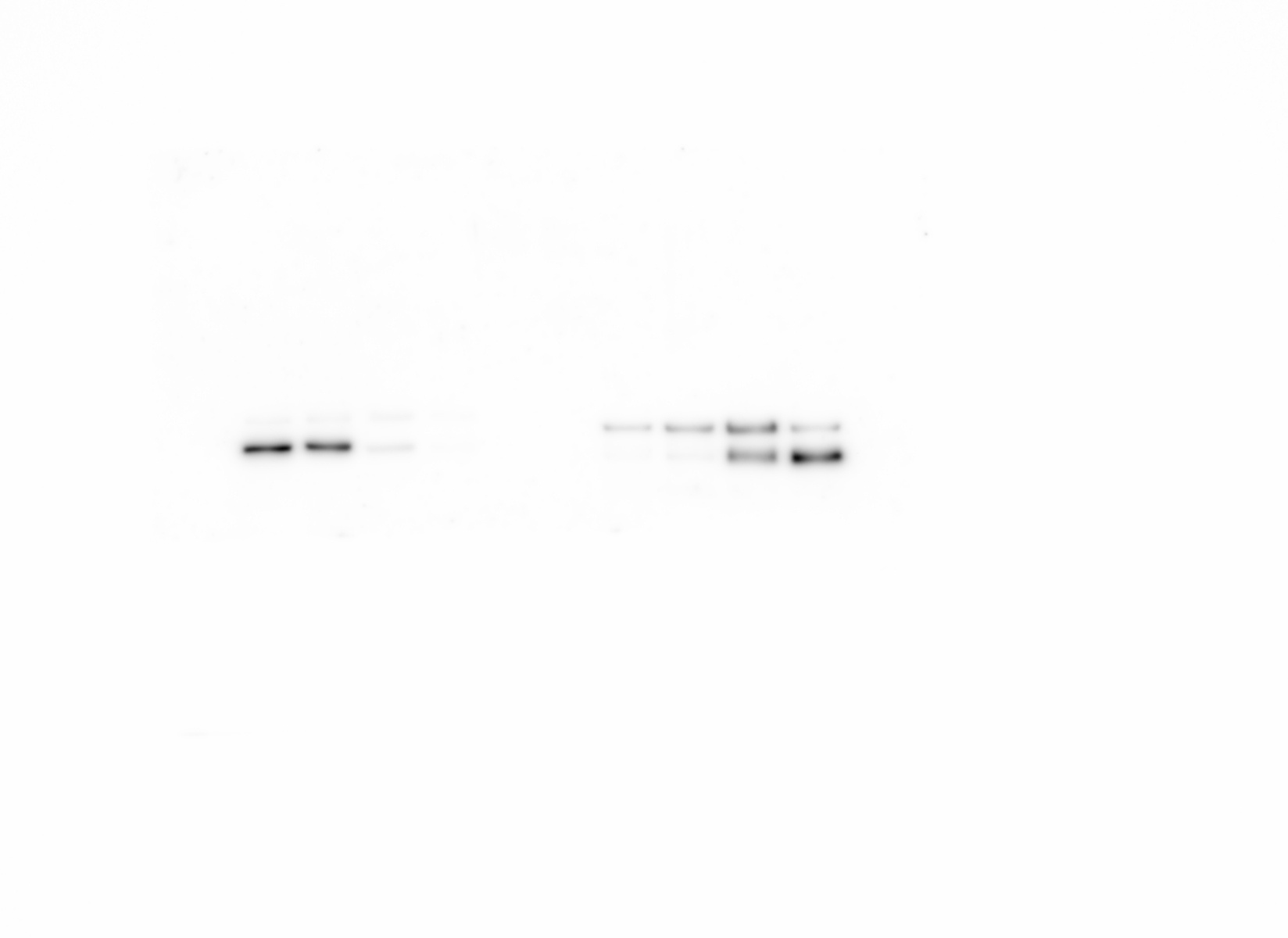

Supplement: Supplementary file 11 — Source data for Appendix [file 44318_2024_305_MOESM11_ESM.zip › Appendix/Appendix Figure S5/S5C/GALNT7 16bit original 20240214_162426-09_Ch_Chemi.tif]

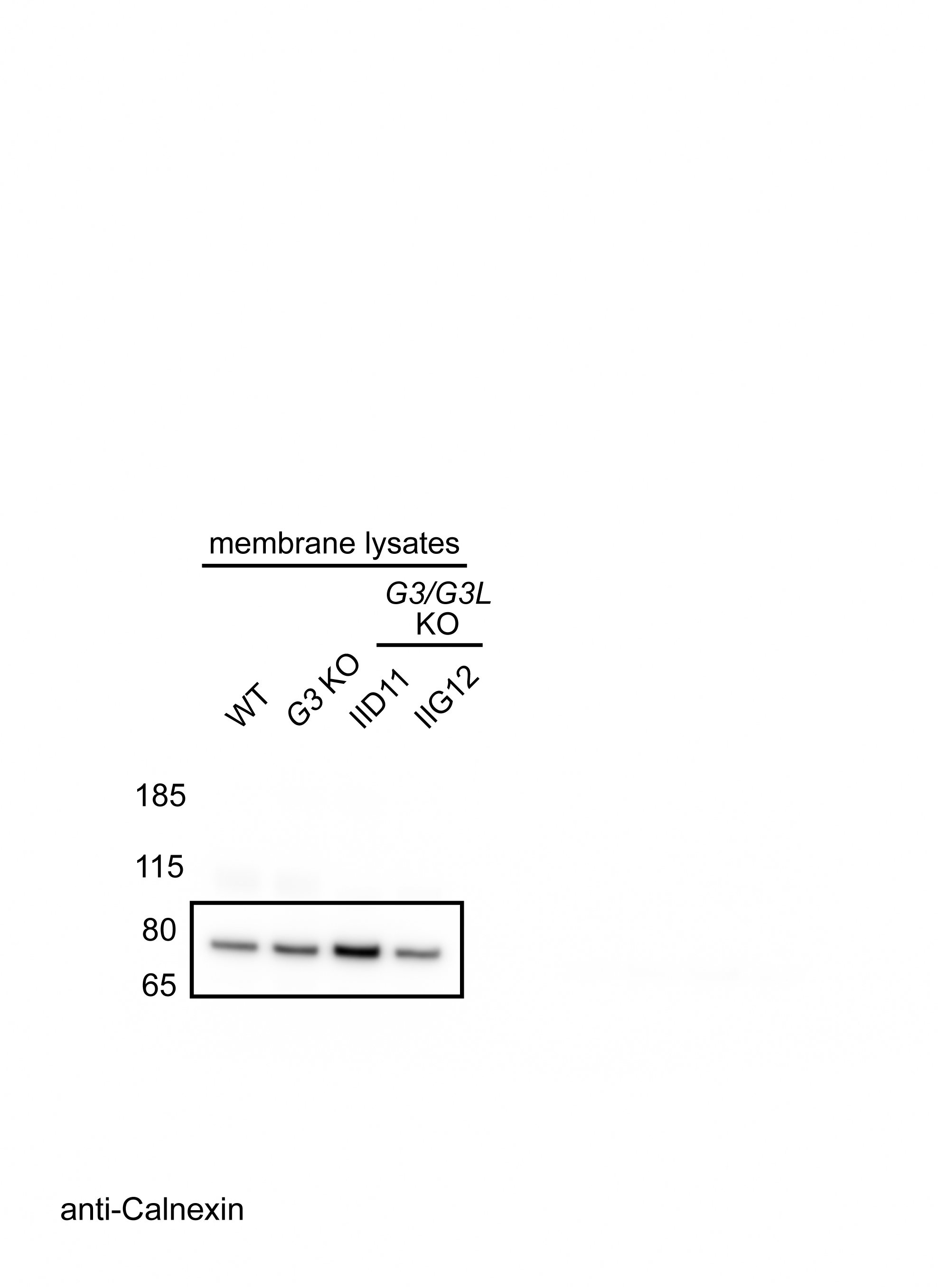

Supplement: Supplementary file 11 — Source data for Appendix [file 44318_2024_305_MOESM11_ESM.zip › Appendix/Appendix Figure S5/S5C/Calenxin for GPP130 8bit annotated 20240312_153722-02_Ch_Chemi-01.tif]

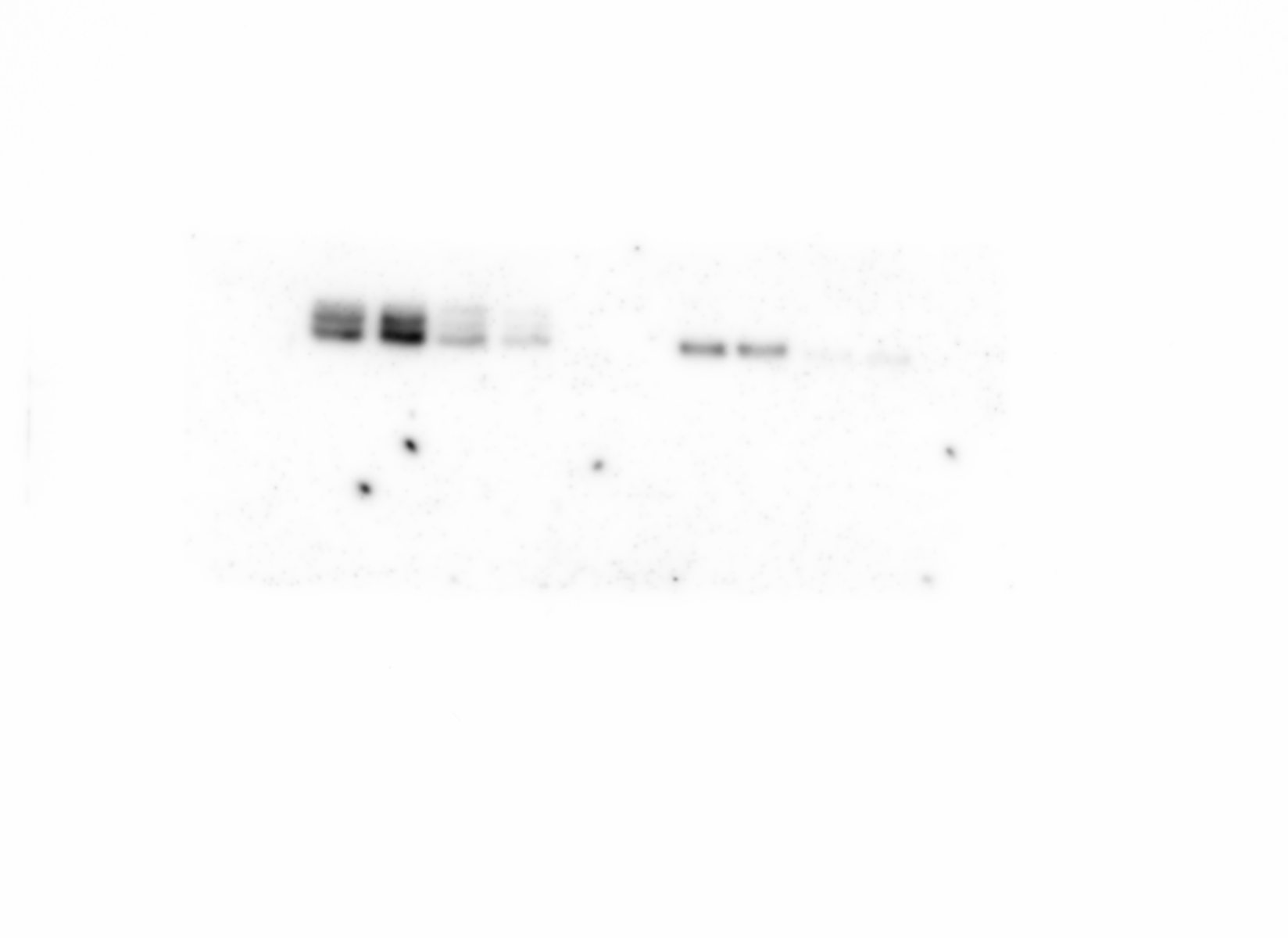

Supplement: Supplementary file 11 — Source data for Appendix [file 44318_2024_305_MOESM11_ESM.zip › Appendix/Appendix Figure S5/S5C/CANT1 16bit original 20240208_140211-10_Ch_Chemi.tif]

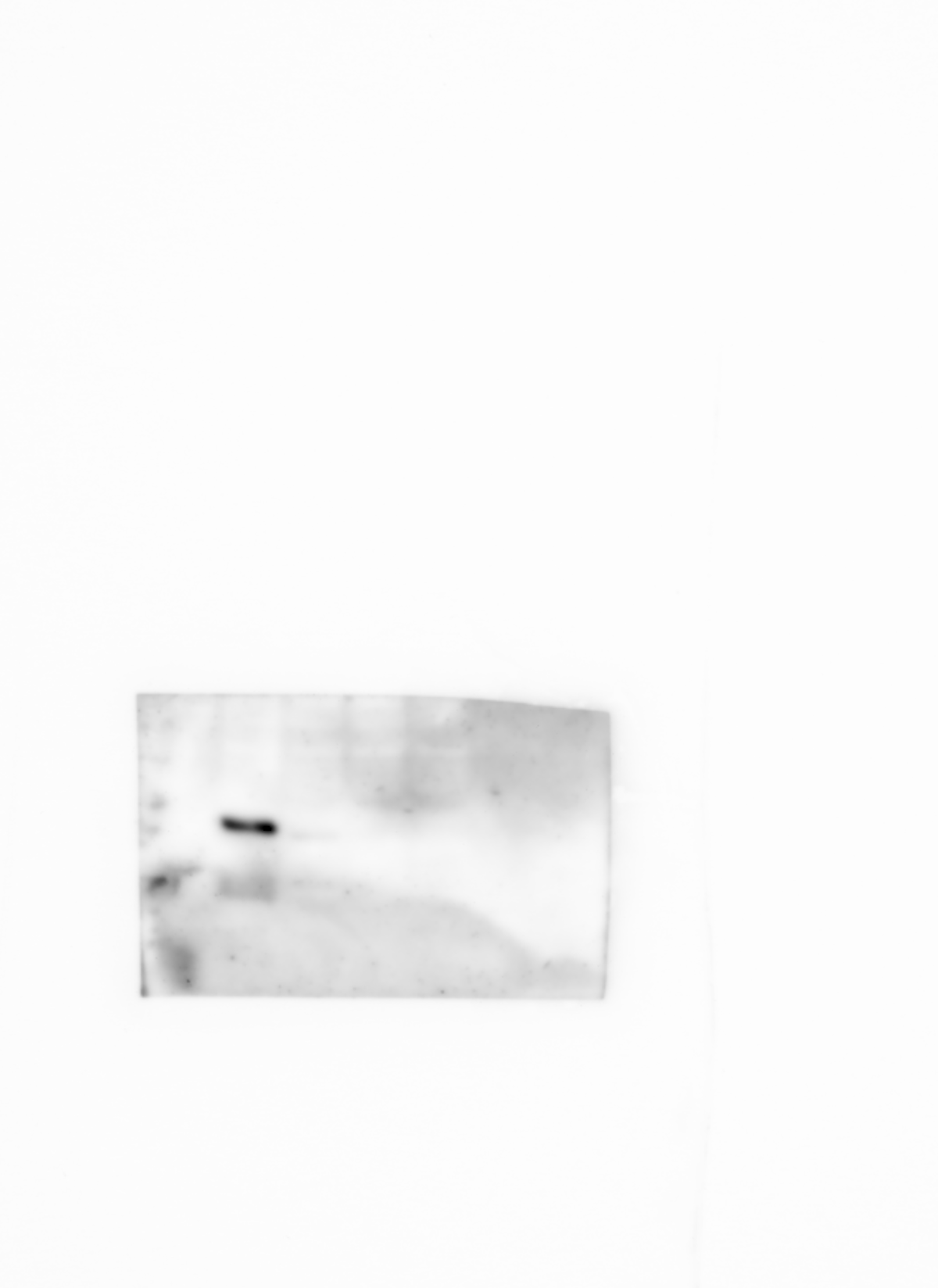

Supplement: Supplementary file 11 — Source data for Appendix [file 44318_2024_305_MOESM11_ESM.zip › Appendix/Appendix Figure S5/S5C/GOLPH3 (PTG) 16bit original 20240207_152419-26_Ch_Chemi.tif]

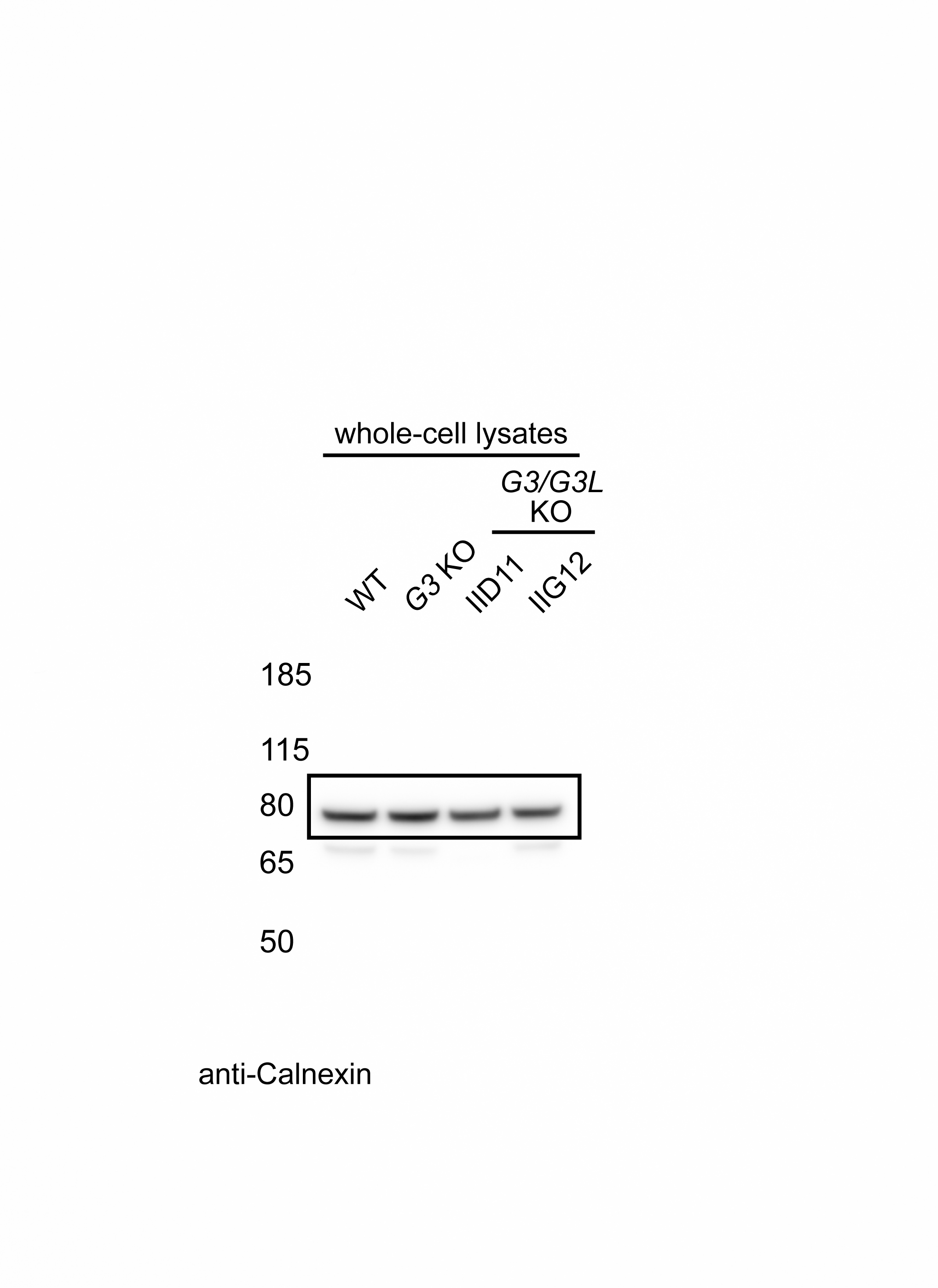

Supplement: Supplementary file 11 — Source data for Appendix [file 44318_2024_305_MOESM11_ESM.zip › Appendix/Appendix Figure S5/S5C/Calnexin for GOLPH3 8bit annotated 20240207_151235-06_Ch_Chemi-01.tif]

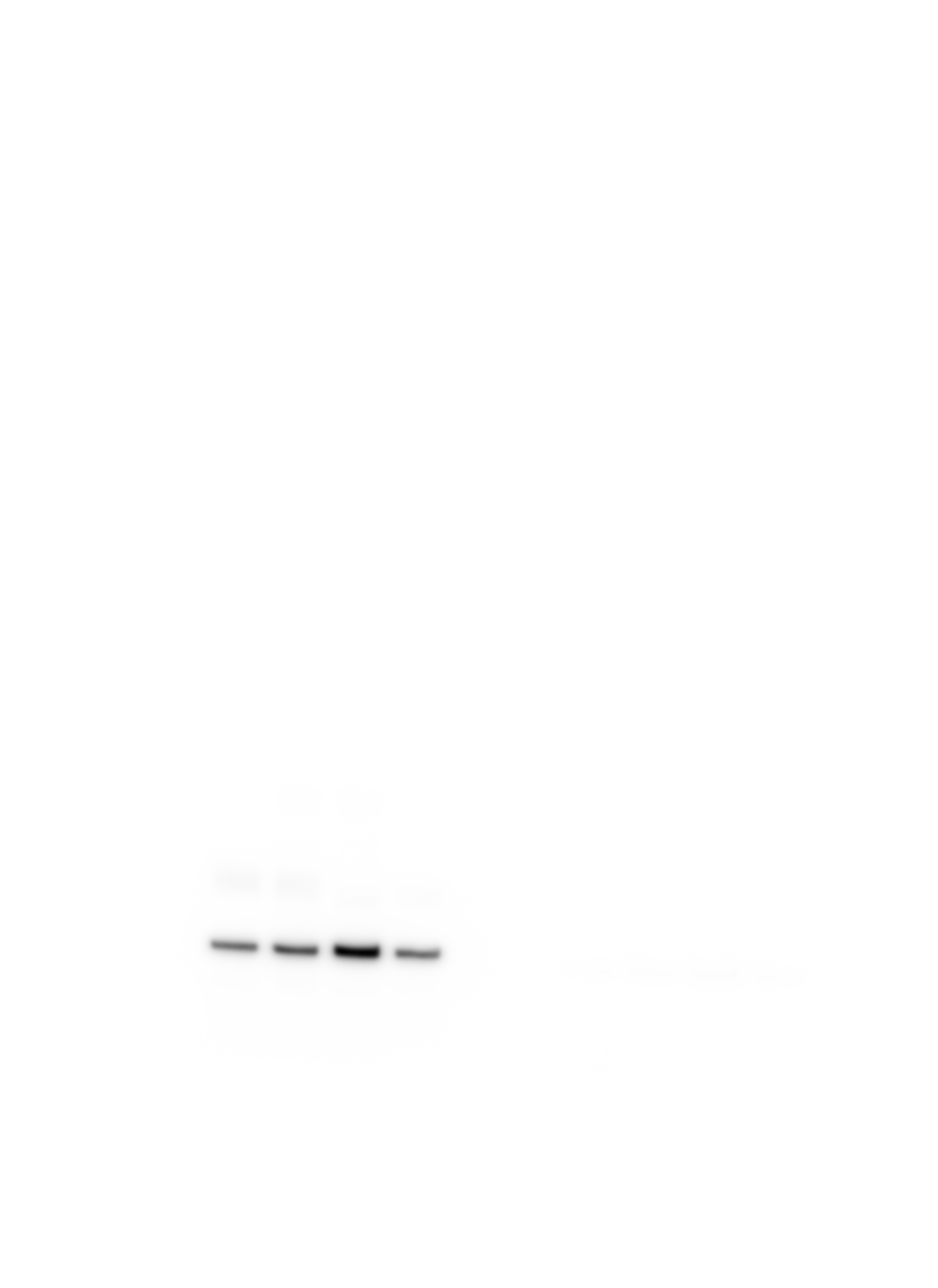

Supplement: Supplementary file 11 — Source data for Appendix [file 44318_2024_305_MOESM11_ESM.zip › Appendix/Appendix Figure S5/S5C/Calnexin for GPP130 16bit 20240312_153722-02_Ch_Chemi.tif]

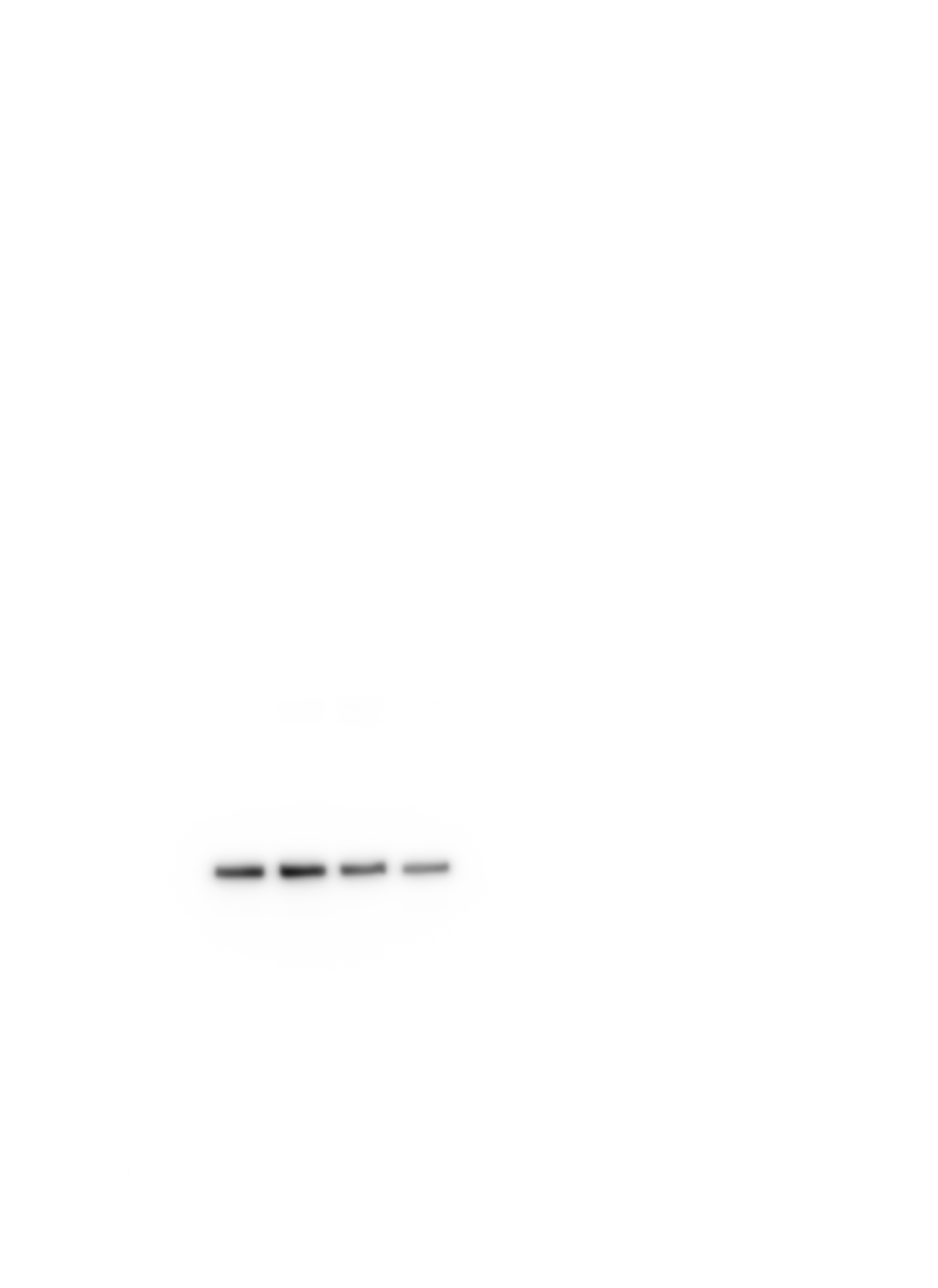

Supplement: Supplementary file 11 — Source data for Appendix [file 44318_2024_305_MOESM11_ESM.zip › Appendix/Appendix Figure S5/S5C/Calnexin for CANT1 16bit original 20240312_152948-02_Ch_Chemi.tif]

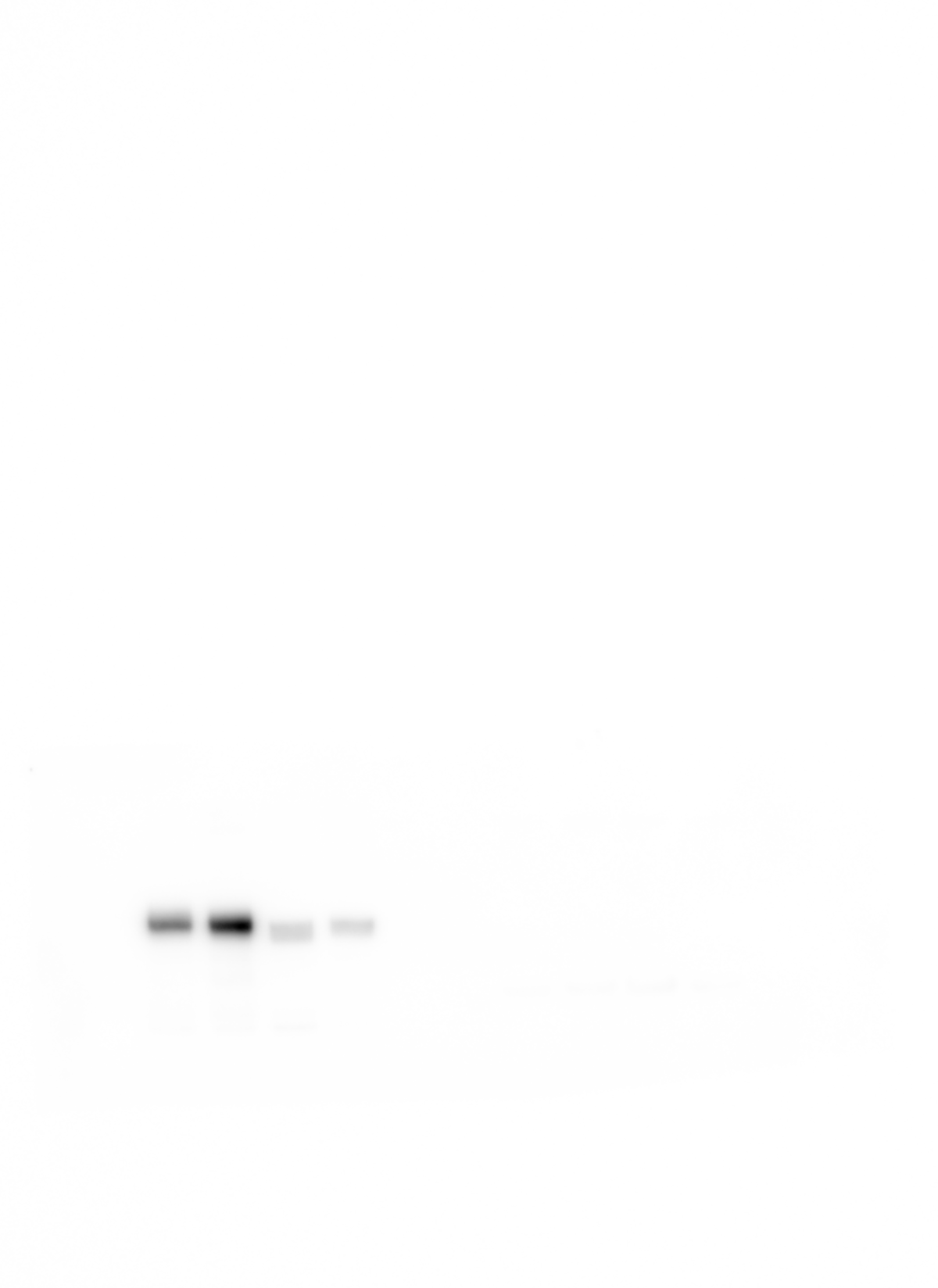

Supplement: Supplementary file 11 — Source data for Appendix [file 44318_2024_305_MOESM11_ESM.zip › Appendix/Appendix Figure S5/S5C/GPP130 16bit original 20240213_143725_Ch_Chemi.tif]

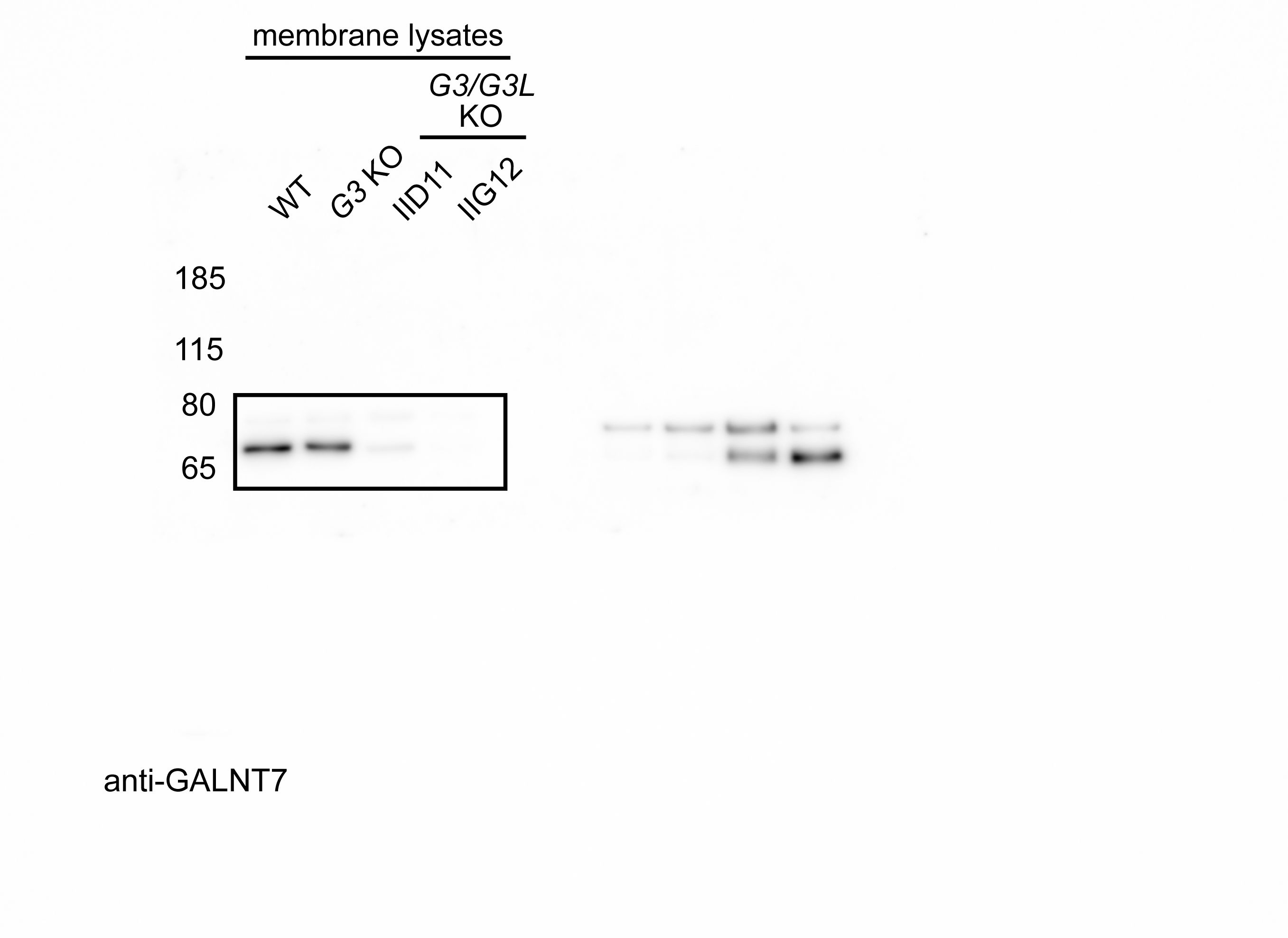

Supplement: Supplementary file 11 — Source data for Appendix [file 44318_2024_305_MOESM11_ESM.zip › Appendix/Appendix Figure S5/S5C/GALNT7 8bit annotated 20240214_162426-09_Ch_Chemi-01.tif]
