# Supplementary material for: GOLPH3 and GOLPH3L maintain Golgi localization of LYSET and a functional mannose 6-phosphate transport pathway
Source: EMBO J. 2024 Nov 25;43(24):6264–90. doi: 10.1038/s44318-024-00305-z (PMC11649813; doi:10.1038/s44318-024-00305-z)
Supplement: Supplementary file 12 — Expanded View Figures [file 44318_2024_305_MOESM12_ESM.pdf]

Expanded View Figures

Figure EV1. Alphafold2 Multimer prediction of a potential interaction of LYSET and GOLPH3.

(**A**) Schematic overview of the topology of LYSET. Based on Uniprot annotations, LYSET is a double-pass membrane protein with cytosolic N- and C-termini. Putative initiator methionines for the long and short isoform are highlighted in magenta and positively charged lysine and arginine residues in green. The sketch was generated using Protter (Omasits et al, 2014). (**B**) Alphafold2 Multimer prediction of a potential interaction of LYSET and GOLPH3 with coloring based on the prediction confidence score. (**C**) LYSET:GOLPH3 complex as depicted in (**B**) with GOLPH3 colored in red and LYSET in blue. Annotated TMD1 and TMD1 along with a putative TMD predicted by DeepTMHMM (Hallgren et al, 2022) in LYSET are colored in yellow. Insert, magnification of the LYSET:GOLPH3 interaction interface with side chains of select positively charged residues in LYSET and negatively charged residues in GOLPH3 shown. (**D**) Face-down view from the membrane plane on the predicted interaction of the LYSET N-terminus (blue) and GOLPH3 (surface charge shown). Side chains of positively charged amino acid residues in the LYSET N-terminus are shown. A similar putative cargo binding site is conserved in GOLPH3L (right). (**E**) Co-immunoprecipitation of LYSET and GOLPH3 in HEK293 transfectants. WT HEK293 cells were transfected as indicated and GOLPH3 was immunoprecipitated with anti-GOLPH3 (ab98023) from obtained cell lysates. Immunoprecipitates were analyzed by immunoblotting. HC, antibody heavy chain. (**F**) Transient overexpression of C-terminally myc-tagged LYSET (isoform 1) variants, that carry point mutations in select arginine residues in the N-terminal cytosolic region, in *LYSET* KO HEK293FT cells. LYSET protein levels were analyzed by immunoblotting with equal proteins amounts loaded and actin serving as a loading control. Source data are available online for this figure.

© The Author(s)

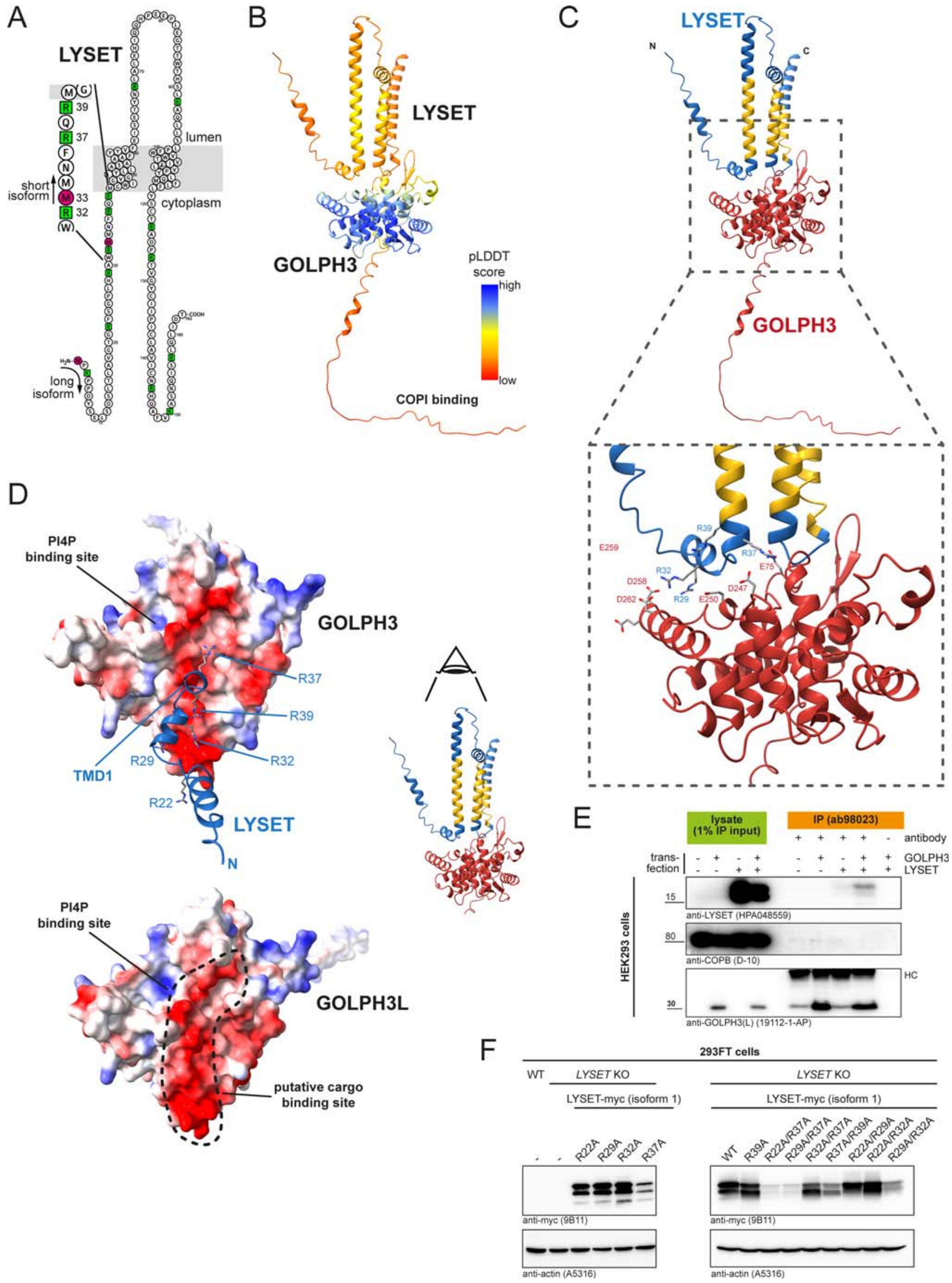
A
LYSET
39
37
short isoform
33
32
lumen
cytoplasm
long isoform
B
LYSET
GOLPH3
pLDDT score
high
low
COPI binding
C
LYSET
N
C
GOLPH3
R39
R37
E259
R32
E75
D258
D247
D262
R29
E250
D
PI4P binding site
GOLPH3
R37
R39
TMD1
R29
R32
R22
LYSET
N
PI4P binding site
GOLPH3L
putative cargo binding site
E
lysate (1% IP input)
IP (ab98023)
antibody
trans-fection
GOLPH3
LYSET
HEK293 cells
15
anti-LYSET (HPA048559)
80
anti-COPB (D-10)
HC
30
anti-GOLPH3(L) (19112-1-AP)
F
293FT cells
WT
LYSET KO
LYSET-myc (isoform 1)
LYSET KO
LYSET-myc (isoform 1)
R22A
R29A
R32A
R37A
WT
R39A
R22A/R37A
R29A/R37A
R32A/R37A
R37A/R39A
R22A/R29A
R22A/R32A
R29A/R32A
anti-myc (9B11)
anti-actin (A5316)
anti-myc (9B11)
anti-actin (A5316)

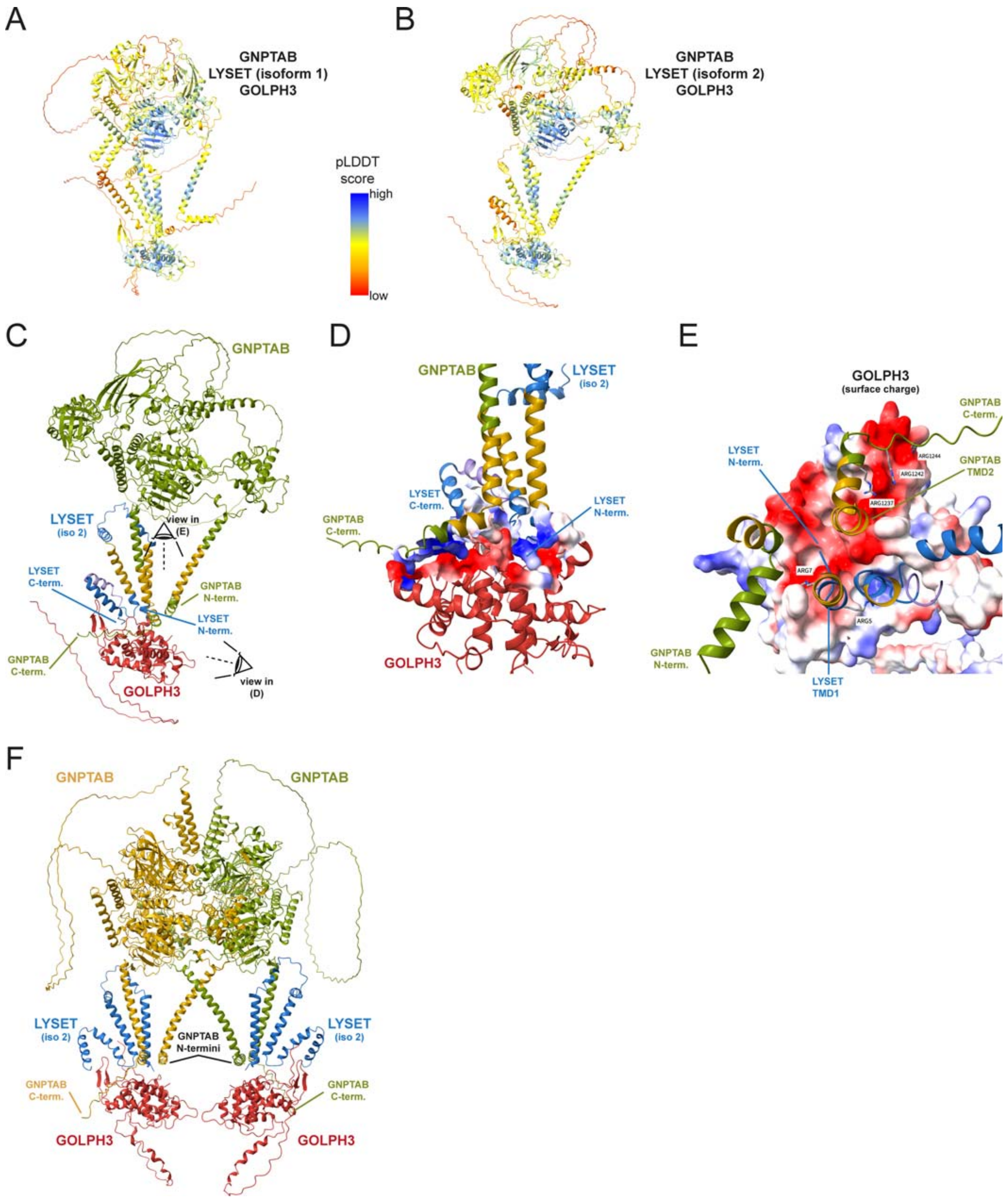
A
GNPTAB
LYSET (isoform 1)
GOLPH3
B
GNPTAB
LYSET (isoform 2)
GOLPH3
pLDDT
score
high
low
C
GNPTAB
LYSET
(iso 2)
view in
(E)
LYSET
C-term.
GNPTAB
N-term.
LYSET
N-term.
GNPTAB
C-term.
GOLPH3
view in
(D)
D
GNPTAB
LYSET
(iso 2)
LYSET
C-term.
LYSET
N-term.
GNPTAB
C-term.
GOLPH3
E
GOLPH3
(surface charge)
GNPTAB
C-term.
LYSET
N-term.
ARG1244
ARG1242
GNPTAB
TMD2
ARG1237
ARG7
ARG5
GNPTAB
N-term.
LYSET
TMD1
F
GNPTAB
GNPTAB
LYSET
(iso 2)
GNPTAB
N-termini
LYSET
(iso 2)
GNPTAB
C-term.
GNPTAB
C-term.
GOLPH3
GOLPH3

 © The Author(s)

Figure EV2. AlphaFold 3 predictions of a ternary interaction of GOLPH3, LYSET and GNPTAB.

(**A**) Structure of the predicted GOLPH3:LYSET (isoform 1):GNPTAB complex shown in Fig. 6A with coloring reflecting the prediction score. (**B**) Structure of the predicted GOLPH3:LYSET (isoform 2):GNPTAB complex with prediction score coloring. (**C**) Structure of the complex shown in (**B**). Individual subunits are given in different colors. Annotated TMD regions are colored in yellow. In case of LYSET, a putative TMD region predicted by DeepTMHMM (Hallgren et al, 2022) is shown in purple. (**D**) Side view the complex containing LYSET isoform 2 shown in (**B**, **C**), highlighting close proximity of GOLPH3 and the LYSET N-terminus as well as the GNPTAB C-terminus. Surface electrostatic potential is shown for selected amino acid side chains. (**E**) Same complex as shown in (**B**, **C**), but top-down view from the membrane plane onto the GOLPH3 surface. Positively charged amino acid side chains in LYSET and GNPTAB are shown as stick representation. (**F**) Alphafold 3 prediction of a complex containing a GNPTAB dimer (yellow and green) as well two LYSET (isoform 2) and two GOLPH3 subunits.

© The Author(s)
